# Detours increase local knowledge—Exploring the hidden benefits of self-control failure

**DOI:** 10.1371/journal.pone.0257717

**Published:** 2021-10-01

**Authors:** Christian Dirk Wiesner, Jennifer Meyer, Christoph Lindner

**Affiliations:** 1 Department of Clinical Psychology and Psychotherapy, Institute of Psychology, Christian-Albrechts-University, Kiel, Germany; 2 Leibniz-Institute for Science and Mathematics Education (IPN), Kiel, Germany; 3 Educational Psychology, Faculty of Education, University of Hamburg (UHH), Hamburg, Germany; Julius-Maximilians-Universität Würzburg, GERMANY

## Abstract

Self-control enables people to override momentary thoughts, emotions, or impulses in order to pursue long-term goals. Good self-control is a predictor for health, success, and subjective well-being, as bad self-control is for the opposite. Therefore, the question arises why evolution has not endowed us with perfect self-control. In this article, we draw some attention to the hidden benefits of self-control failure and present a new experimental paradigm that captures both costs and benefits of self-control failure. In an experiment, participants worked on three consecutive tasks: 1) In a transcription task, we manipulated how much effortful self-control two groups of participants had to exert. 2) In a number-comparison task, participants of both groups were asked to compare numbers and ignore distracting neutral versus reward-related pictures. 3) After a pause for recreation, participants were confronted with an unannounced recognition task measuring whether they had incidentally encoded the distracting pictures during the previous number-comparison task. The results showed that participants who exerted a high amount of effortful self-control during the first task shifted their priorities and attention toward the distractors during the second self-control demanding task: The cost of self-control failure was reflected in worse performance in the number-comparison task. Moreover, the group which had exerted a high amount of self-control during the first task and showed self-control failure during the second task was better in the unannounced third task. The benefit of self-control failure during number comparison was reflected in better performance during the recognition task. However, costs and benefits were not specific for reward-related distractors but also occurred with neutral pictures. We propose that the hidden benefit of self-control failure lies in the exploration of distractors present during goal pursuit, i.e. the collection of information about the environment and the potential discovery of new sources of reward. Detours increase local knowledge.

## 1. Introduction

In this article, we would like to draw some attention to the paradox of self-control and the hidden benefits of self-control failure and introduce a new paradigm to uncover these hidden benefits.

A popular definition of self-control states that self-control enables people to override thoughts, emotions, and behavioral impulses that compete with overarching goals [1], e.g. inhibiting the urge to eat fast food to achieve the goal to become lean and stay healthy. In a broader sense, self-control enables us to bring our responses in line with long-term goals that are derived from ideals, values, morals, or social expectations [1]. Some more general variants of this definition simply focus on advancing one goal over the other [2]. More specific definitions emphasize that self-control enables us to forgo a small reward obtainable immediately or with low effort in favor of a large reward obtainable after a delay or only with high effort [3]. Three aspects are common in most definitions: first, a conflict between goals, second, the questions of what is best for the individual, and third, some kind of cost calculation regarding the delay, probability, or effort.

Moreover, most authors agree on the positive consequences of self-control and the negative consequences of self-control failure. On the positive side, high trait self-control is associated with academic achievements [4, 5], financial and labor market success [6], better social skills and relationship satisfaction [7], physical fitness [8], psychological well-being [9], and life satisfaction [10]. On the negative side, low trait self-control is associated with procrastination [11], financial problems [12], unemployment [13], aggressive and criminal behavior [14], obesity [15], and psychiatric disorders such as mood or anxiety disorders, eating disorders, substance addiction, problem gambling, or attention-deficit hyperactivity disorder [16].

In an opinion piece, Hayden [3] very aptly summarized the paradox of self-control in one question: “Why has evolution not selected for perfect self-control?”. Regarding the consequences of self-control (failure), it seems obvious that most instances of self-control should be adaptive and advantageous for survival and reproduction and vice versa. Therefore, the question arises why evolution has not endowed us with perfect self-control. While the paradox results from a focus on the benefits of self-control and the costs of self-control failure, the answer has to look at the costs of self-control and the benefits of self-control failure. In his article, Hayden proposes that imperfect self-control is the result of an evolutionary compromise between the benefits and drawbacks of automatization on the one side and self-control on the other, i.e. a case of bounded optimality. On the one hand, automatization enables fast, effortless, less variable responses and frees up limited processing capacity for other tasks. On the other hand, automatized processes become less penetrable to modulatory influences, i.e. self- control. Automatization comes in different flavors and with different drawbacks. Hard-wired processes help us to feed and mate, but also make us susceptible to addictive substances, high-caloric food, and internet pornography [17, 18]. Learned habits serve us well and save us processing costs in most cases, but sometimes make self-control especially hard as in the well-known Stroop task [19, 20]. However, apart from pitting automatization against self-control, research has paid too little attention to the real costs of self-control and the hidden benefits of self-control failure. Authors who repeatedly addressed these aspects are Inzlicht, Schmeichel, and colleagues in their process model of self-control [21] and their reward responsivity hypothesis [22].

The process model of self-control addresses the question of why self-control seems but may not be limited [21], at least not as much as many studies suggest [23–25]. The authors propose that effortful self-control is inherently aversive and self-control failure occurs when people switch their task priorities from cognitive labor (self-control demanding tasks) to cognitive leisure (pleasant activities). The switch of task priorities is an expression of the attempt to achieve a balance between these two aspects. The process model then offers explanations on three different levels.

The ultimate explanation is inspired by evolutionary psychology and refers to the idea that organisms strive for an optimal exploitation-exploration trade-off [26]. Humans, like other foraging animals repeatedly face the choice either to exploit a known source of reward or to explore the environment for new potential sources of reward. Both alternatives come with different (opportunity) costs and benefits [27]. The process model uses a definition of exploitation and exploration that originates from research on reward processing [28]. However, these concepts have been studied in numerous disciplines such as vision science, ecology, machine learning, and economics [29]. A common feature of these definitions is that they assume that the individual seeks to maximize some form of utility by finding the right exploitation-exploration trade-off, and this utility need not consist merely of reward, but may also include information, knowledge, or skills [29–32]. Many self-control demanding tasks resemble exploitation and disengagement from these tasks and switch to alternatives resembles exploration [21]. For example, working on a manuscript is like exploiting a known source of reward, and getting distracted by social media is like exploring other stimuli as potential sources of reward. In this sense, one could call exploitation in a self-control demanding task successful self-control and exploration self-control failure. However, labeling exploration as self-control failure is oversimplified and ignores the benefits of exploration, i.e. gaining information about the environment and potential new sources of reward. Moreover, concerning the self-control paradox introduced above, “self-control failure” can be seen as an integral part of an adaptive strategy ensuring an optimal trade-off between exploration and exploitation. In this article, we introduce a new experimental paradigm that uncovers the hidden benefits of self-control failure, especially the benefits of task disengagement and exploration.

The process model of self-control also offers an intermediate and a proximal explanation for apparent self-control failure [21]. The ultimate account of balancing exploitation and exploration translates to the intermediate account of balancing cognitive labor and cognitive leisure, respectively. Effortful self-control during cognitive labor is deemed inherently aversive [33]. According to the model [21], this inherent disutility of effortful self-control accumulates during prolonged or repeated cognitive work and motivates the individual to shift their priorities to cognitive leisure. In the last step, this shift translates to the proximal account, i.e. a shift away from the self-control demanding task toward more pleasant activities. This shift encompasses a shift of motivation, attention, and emotion away from the goals associated with cognitive labor toward the goals associated with cognitive leisure. Hence, the model predicts that people who engage in prolonged or repeated acts of effortful self-control will shift their attention away from stimuli relevant for cognitive labor toward stimuli associated with cognitive leisure. In other words, the individual will exploit less and explore more.

While the process model has a great heuristic and integrative value, it is a bit vague regarding the psychological mechanisms of the shift. However, the authors put forward the reward responsivity hypothesis to fill this gap [22]. The premises are the same as in the process model: self-control is seen as an inherently aversive activity. Moreover, individuals attempt to maintain or restore a positive emotional state. Therefore, prolonged or repeated effortful self-control results in a negative emotional state. As an opponent process, the sensitivity for reward temporally increases, i.e. the degree or intensity of responding to reward-associated stimuli is enhanced [22, 34]. Phenomenologically this is supposed to make the individual feel like they would deserve a reward for their prior effort. On the behavioral level, the individual would have a stronger tendency to approach reward-related stimuli in order to restore a positive emotional state. In their review, the authors present evidence for the reward responsivity hypothesis regarding different types of reward-seeking behavior following effortful self-control, especially food choice, drug addiction, and money-related behaviors like decision-making [22]. In the discussion section of the present article, we review further empirical evidence in favor of and against this hypothesis.

The fine difference between the ultimate account of the process model and the reward responsivity lies in the benefits of self-control failure. Whereas the ultimate account recognizes the benefits of exploration, the reward responsivity hypothesis focuses on the restoration of a positive emotional state. On the one hand, the benefits lie in the acquisition of knowledge about the environment and the potential discovery of new sources of reward. On the other hand, reward-seeking is only a means to the end of a positive emotional state. It is important to note that the two theories are not mutually exclusive. Rather the reward responsivity hypothesis just reaches a little farther than the process model because it is more specific about the psychological processes involved.

The current experiment has two main goals: First, we would like to introduce a new paradigm that captures the costs and benefits of self-control failure as well. We hope that people will find this paradigm interesting and use it to further investigate the mechanism and consequences of self-control failure. Second, we aim to test the predictions of the ultimate account of the process model and the reward responsivity hypothesis. In specific, we test whether previous effortful self-control just increases the tendency to explore task-irrelevant stimuli regardless of valence or whether it only increases the response to positive, reward-associated stimuli. In both cases, we expect that exerting effortful self-control leads to a shift of task priorities and therefore attention from cognitive labor in a self-control demanding task to cognitive leisure, i.e. the exploration of distracting pictures. Note, that the experiment aims to investigate the attention shift hypothesis while the process model and the reward responsivity hypothesis make further assumptions about intervening processes, especially motivation.

The experiment encompassed three steps: In the first step, the amount of necessary effortful self-control was manipulated using a handwriting transcription task. In the experimental condition, a higher amount of effortful self-control was necessary to complete the task as compared to the control condition. In the second step, participants were asked to focus on a self-control demanding number-comparison task while simultaneously ignoring distracting neutral versus reward-related pictures. As previous research has shown, reward-related stimuli tend to “capture the eye” [35] and therefore should be harder to ignore and easier to encode in memory [36]. During a pause, participants were asked to watch a video about abstract painting for relaxation. This was done in order to let participants completely recover from the previous depleting tasks. In the third step, an unannounced recognition task was presented to assess whether participants had incidentally encoded the pictures which were previously presented as distractors during the number-comparison task. Note that the unannounced explicit recognition task in part three of the experiment allowed us to assess the benefits of self-control failure during the second self-control task. If participants explored the task-irrelevant pictures while they were required to focus on the task-relevant numbers, they would show better recognition performance in part three. Moreover, if the exploration of the distractors would be caused by the search for reward—as the reward responsivity hypothesis states—we would expect that reward-related distractors lead to even better encoding and recognition in the experimental group.

In summary, we expected that the experimental group would shift their task priorities from the self-control demanding number-comparison task to the distracting pictures to a greater degree as compared to the control group and hence would need more time to solve the explicit number-comparison tasks and would show better performance in recognizing the implicitly encoded pictures. Furthermore, we expected that this attention shift would be more pronounced for reward-related pictures as compared to neutral pictures.

## 2. Methods

### 2.1 Ethics statement

The study protocol was discussed and approved by the research colloquium of the department of psychology of the University of Kiel. Participants received course credits for their participation. However, participation was optional and all participants gave written informed consent before the beginning of the experiment. This study was carried out in accordance with the Declaration of Helsinki and the ethical guidelines for experimental research with human participants as proposed by the American Psychological Association.

### 2.2 Participants

The required sample size was calculated using g*power (version 3.1.3) [37]. We calculated the required total sample size to detect an interaction effect in an ANOVA with the between-subject factor Group (experimental vs. control) and the within-subject factor Distractor Valence (neutral vs. positive). The assumed effect size of *f* ≥ 0.25 (*d* ≥ 0.5) and the correlation between measurements of *r* ≥ .6 were derived from a previous study of our group where we used the same experimental manipulation and stimulus material [38]. We assumed an alpha of .05 and a minimal power of .95. According to g*power, the required total sample size is N = 44 to obtain a power greater than .95. Therefore, our sample size of 61 should be on the safe side. However, because the dependent measures of the previous and the current study are different, the effect size is only a guess and we additionally report sensitivity analyses in the results section taking into account the observed correlation coefficients of repeated measures.

We included undergraduate psychology students with normal or corrected to normal vision in the sample. Potential participants were informed that they would be asked to transcribe a text and would be working on cognitive tasks encompassing verbal, numerical, and pictorial stimuli presented on a computer screen. As we wanted to measure incidental memory encoding, we omitted any hints suggesting a subsequent recognition task (see details below). After the experiment participants filled out a funnel questionnaire concerning their hypotheses about the experiment and the recognition task. One participant had to be excluded because he or she expected the memory test and intentionally encoded the pictures. Four participants were excluded because they did not match the inclusion criteria (age, vision, study subject). Five participants in each group were excluded due to multiple reaction times shorter than 500 ms (details see section 2.4). The final sample consisted of 61 participants (54 female, 7 male) aged 24.2 ± 4.7 (mean ± SD). The participants were randomly divided into a control (n = 29) and an experimental group (n = 32). The groups did not differ regarding gender, handedness, age, or secondary-school grade point average (GPA). Further details are reported in Table 1.

**Table 1:** Participant characteristics.

Variable	Control	Experimental	Comparison			
	*n*	*n*	*p*			
Gender	27 female / 2 male	27 female / 5 male	.429			
Handedness	3 left / 26 right	3 left / 29 right	≈ 1			
	mean ± SD	mean ± SD	*df*	*t*	*p*	*Cohen’s d*
Age (years)	24.31 ± 4.97	24.00 ± 4.55	59	0.26	.800	0.065
GPA (grade)	1.80 ± 0,66	1.83 ± 0,56	58	-0.16	.874	-0.041

GPA, the grades of the German secondary school grade point averages vary between 1.0 (very good) and 4.0 (satisfactory). Gender and handedness were compared using Fisher’s exact test. Age and GPA were compared using t-tests. The p-values correspond to two-tailed tests.

### 2.3 Manipulation: Transcription task

**Fig 1:**
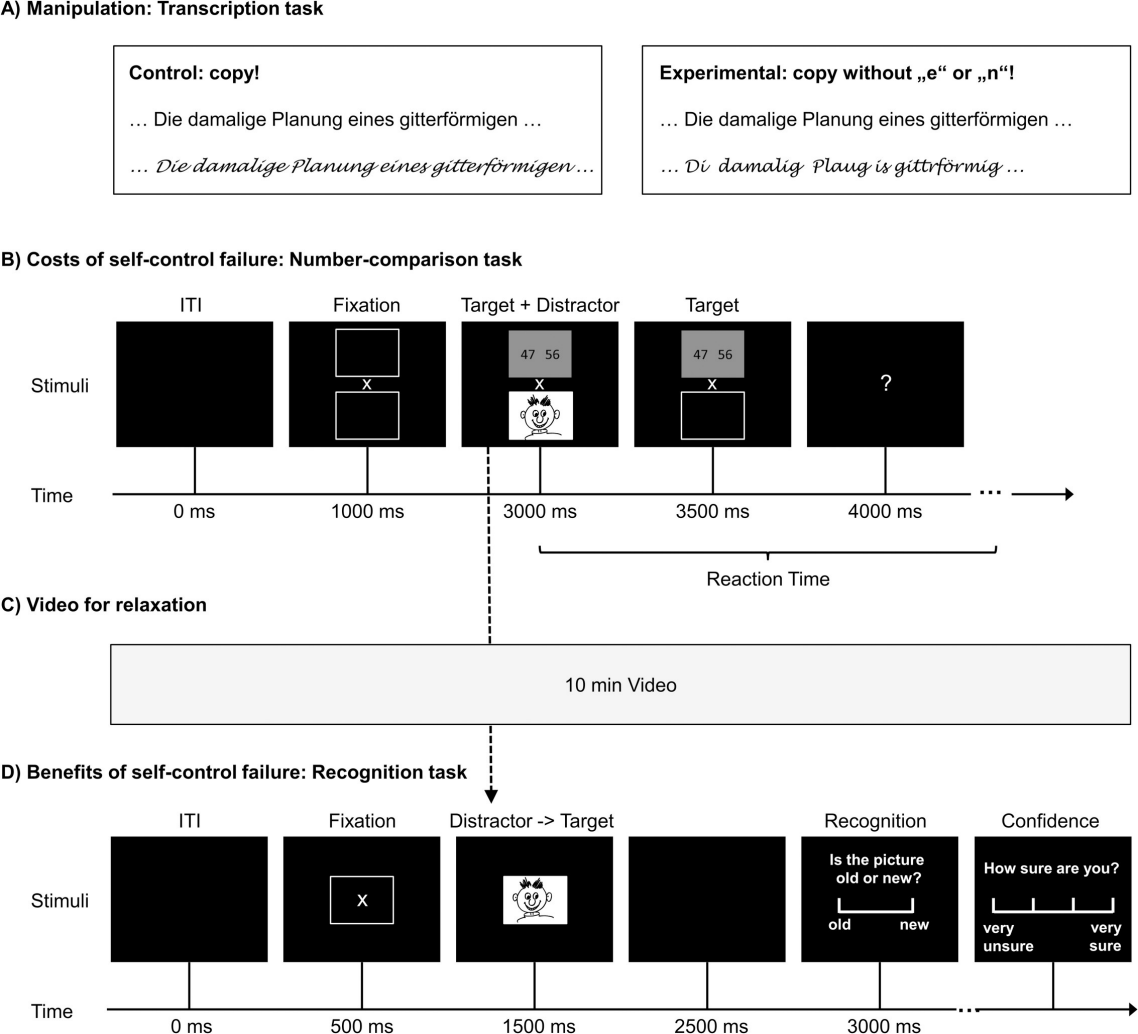
Tasks. A) The first task was used to manipulate the amount of effortful self-control that participants had to exert. In this transcription task, participants were asked to copy a text either omitting the frequent letters “e” and “n” (experimental) or as usual (control). B) The second task was used to measure the costs of self-control failure. Participants of both groups were asked to work on a number-comparison task and to ignore the pictures. Participants had to indicate whether the left or right of the two-digit numbers was bigger. The distracting photographic pictures either showed reward-related stimuli (e.g. chocolate cake) or matched neutral stimuli (e.g. grey bread). C) To introduce a delay between incidental memory encoding and recognition task as well as to allow the participants to recover from the previous effortful tasks, a 10 min long video about abstract painting was shown and the participants were instructed to watch and relax. D) The third task was used to measure the hidden benefits of self-control failure. In an unannounced recognition task, participants were asked whether they had seen the pictures before and how confident they were about their judgment. One-half of the pictures were “distractors” from the second task and the other half new pictures, i.e. former distractors became targets. A shift of the attention from the number-comparison task to the distracting pictures would result in better recognition performance later on.

To manipulate the amount of exerted effortful self-control in step 1 of the experiment we employed a handwriting transcription task which has been used in a similar form in numerous other studies [e.g. 38–47]. Participants were asked to transcribe by hand a text dealing with the history of the city of Mannheim in Germany (see Fig 1A). In the low effortful self-control condition (control) no further instructions were given. In the high effortful self-control condition (experimental) participants were instructed to skip any instance of the letters “e” and “n”, which are the most frequently used letters in the German language. To comply with this rule, participants had to exert effortful self-control, i.e. monitor their well-learned, highly automated writing habits and inhibit pre-potent impulses to write the whole word instead of omitting the forbidden letters. After 8 min the experimenter stopped the transcription task.

As a manipulation check, we used a three-item scale (Cronbach’s *α* = .785) measuring the effortful self-control exerted during the handwriting transcription task [39, 40]. Participants rated effort, difficulty, and how much they needed to suppress their usual writing habits on 7-point Likert scales ranging from 1 “not at all” to 7 “very much so” (e.g. “How much did you suppress your usual writing habits during the copying task?”). To ensure that the manipulation did not induce changes in the participants’ mood, we applied the German version of the Positive and Negative Affect Schedule [48]. The participants rated their positive (ten adjectives; e.g., “attentive”) and negative affect (ten adjectives; e.g., “angry”) on a 5-point Likert scale ranging from 1 “not at all” to 5 “extremely”. Furthermore, at the end of the experiment, post hoc ratings of difficulty and (un-)pleasantness were conducted: We asked the participants to rate how hard (1 “not at all” to 5 “very much so”) and how unpleasant/pleasant (1 “very unpleasant” to 5 “very pleasant”) it was to work on the transcription task. Descriptive statistics of the manipulation and mood check are reported in Table 3 in the results section.

### 2.4 Measurement of the costs of self-control failure: Number-comparison task

To measure whether participants shift their priorities and hence their attention between tasks, we devised a new experimental paradigm with an explicit self-control demanding task (number comparison) and an implicit memory task (recognition of distractor pictures). The task was programmed in PsychoPy 1.83 [49] and can be downloaded as S2 File below. In the number-comparison task, participants were presented with two-digit numbers on the left and right sides of the screen along with irrelevant pictures (see Fig 1B). The participants were instructed to concentrate only on the numbers, to ignore distracting pictures, and to select as fast and accurately as possible the bigger of the two numbers by pressing the left or right mouse button. Note that the task is designed as a speed test as opposed to a power test. Participants only needed basic number literacy to solve the tasks. Nevertheless, self-control was required to compare numbers in working memory while ignoring distracting stimuli. The numbers spanned from 11 to 91 and each pair differed by 1 to 9. The side of the bigger number was pseudorandomized and each side was correct in 50% of the trials. Each of the 50 trials started with two boxes at the top and the bottom of the screen with a fixation cross in the middle. After 2000 ms one box was filled with the number-comparison task and the other with the irrelevant distractor picture. After 500 ms the irrelevant picture disappeared and the number-comparison task remained on the screen for another 500 ms. If the participant had not responded until now a blank screen with a question mark was displayed until the participant answered. The intertrial interval was 1000 ms. The positions (top vs bottom) of the number-comparison task and the distracting picture were pseudorandomized and balanced over sides (left vs right of the bigger number) and the valence (neutral vs positive) of the distractors. This was done to make the task unpredictable and thereby force the participants to refocus their attention at the beginning of each trial. Two dummy trials were added at the beginning to allow for complete counterbalancing and to familiarize the participant with the task timing. In 25 trials the distractor picture was neutral and in 25 trials positive. There were no more than three distractors of equal valence in a row and neutral and positive distractors were equally frequent in the first and second half of the trials to prevent confounding of fatigue and distractor valence. Reaction times and the number of correct answers in the number-comparison task were recorded. Note that a shift of attention from the number-comparison task (cognitive labor) to the distracting pictures (cognitive leisure) would result in longer reaction times and fewer correct answers.

It was critical that the reaction times were at least 500 ms long so that all distractors were presented for the same duration. If a participant responded faster than 500 ms the trial would prematurely end as would the chance to incidentally encode the distractor. Therefore, to keep the presentation times of the distractor pictures constant the number-comparison task was designed to last at least 500 ms. In several pretests, we adjusted the difficulty of the number task accordingly by changing the timing, the constellation of the numbers, and the contrast of background (grey) and digits (black). However, in a few cases, participants managed to give a correct answer in marginally less than 500 ms. We allowed only one reaction time per participant to be shorter than 500 ms to avoid distortion of the recognition task. Participants with multiple faster reaction times were excluded because the incidental encoding of the distractors would have been impossible (see section 2.2).

The distractor pictures were selected from a stimulus set evaluated in a previous study [38]. The positive, reward-related distractor pictures were chosen to allude to the affiliation motive (humans, e.g. „kissing couple“), to the motivation to care (animals, Kindchenschema, e.g. „cute duckling“), to hunger and appetite (food, e.g. „delicious cake“), or to the appreciation of beauty (sceneries, e.g. „tree by a beautiful lake“). Furthermore, for each positive picture we selected a neutral picture with similar content (e.g. humans: “couple in the office”, animals: “adult sparrow”, food: “grey-bread”, scenery: “tree by a parking lot”). An overview is given in Table 2. The high-resolution pictures were standardized to 800*600 pixels resolution and equal luminance. In the previous study, the pictures were rated for valence and arousal using the self-assessment manikin [50]. In short, positive pictures elicited a significantly more positive and more arousing emotional state than neutral pictures [for details see 38]. In other words, looking at the positive pictures made the participants of that study feel good and thus can be considered as rewarding.

**Table 2:** Stimuli in the number-comparison task and the recognition task.

Number-Comparison Task			Recognition Task	
Targets	Distractors		Targets (old)	Distractors (new)
25 number pairs	25 pos. pictures	→	25 pos. pictures	25 pos. pictures
25 number pairs	25 neu. pictures	→	25 neu. pictures	25 neu. pictures

In the number-comparison task, 50 pairs of numbers were presented as targets for the number-comparison task along with 25 neutral and 25 positive pictures as distractors. The distractors from the number-comparison task became the targets in the subsequent incidental memory recognition task which were presented intermixed with new distractors not shown before.

### 2.5 Measurement of the benefits of self-control failure: Unannounced memory recognition task

Before working on the unannounced recognition task, participants of both groups were asked to watch a video about abstract painting and relax (Fig 1C). The duration of the video break was standardized to 10 min. Watching the video had three functions: First, a delay between the incidental memory encoding during the number-comparison task and the recognition task was introduced. Second, participants were kept busy and discouraged from thinking about the stimuli from the previous task. Third, this rather long resting break allowed the participants of both groups to fully recover from the exertion of self-control during the previous tasks. This was required because we did not aim to measure the effects of previous self-control exertion on recognition, but only on incidental encoding during the number-comparison task.

The distractor images of the previously described number comparison task became the targets in the unannounced recognition task (see Table 2 and Fig 1D). For every distracting picture from the number-comparison task, we selected a new distractor picture not shown before which was matched for the content category, valence, resolution, and luminance. However, the distractor pictures of the recognition task were not formally evaluated but the similarity of content and valence were determined by the consensual decision of the authors.

Each of the 100 Trials (50 targets / 50 distractors) began with an empty box on the screen and a fixation cross in the middle. After 1000 ms the stimulus picture was displayed for 1000 ms. After a blank screen for 500 ms, the two-point recognition rating scale was presented asking the participants whether the picture was old (i.e., seen during the initial number-comparison task) or new. The recognition scale remained on the screen until the participant gave her or his answer by clicking one of the alternatives. Lastly, the four-point confidence rating scale was presented asking the participants how confident they were of their old/new rating. The participants answered by clicking one of four alternatives ranging from 1 “very unsure” to 4 “very sure”. The confidence scale remained on the screen until the participant responded. The inter-trial interval was 500 ms long. Ratings and reaction times were recorded.

To determine the recognition performance, trials were sorted according to target valence, receiver operating characteristic (ROC) curves were constructed for each valence category, and the areas under the curve were computed. To obtain fine-grained ROC curves the recognition (“old” vs. “new”) and confidence (1 to 4) ratings were combined to 8 judgments: From old & 4 (“very sure old”) to old & 1 (“very unsure old”) and from new & 1 (“very unsure new”) to new & 4 (“very sure new”). According to the 8 combined ratings 7 pairs of hit rates and false-alarm rates were calculated defining an ROC curve [51]. Good recognition performance was reflected by large areas under the ROC curve and fast reaction times.

### 2.6 Data processing and statistical analysis

To minimize the effect of outliers, the medians of the reaction times of the number task and the recognition task were computed separately for every condition and valence category and entered into the following statistical analyses. To evaluate the effect of the amount of previously exerted effortful self-control on subsequent task priorities, we computed mixed ANOVAs with the between-subject factor Group (control: low effortful self-control versus experimental: high effortful self-control) and the within-subject factor Valence (neutral versus positive). Four ANOVAs were computed using the number of correct answers and median reaction time of the number-comparison task and area under the curve and median reaction times of the recognition task. In the case of significant effects, Bonferroni corrected post hoc contrasts were computed. The manipulation checks were carried out using unpaired t-tests (two-tailed) comparing the control and experimental group. To check for speed-accuracy trade-offs we computed Pearson’s correlation coefficients of reaction times and the number of correct answers in the number-comparison task and correlation coefficients of reaction times and the area under the curve in the recognition task. All computations were carried out using IBM SPSS Statistics for Windows (2012, Version 21.0. Armonk, NY: IBM Corp.).

## 3. Results

### 3.1 Manipulation check

The manipulation check confirmed that the experimental group exerted more effortful self-control in the transcription task than the control group (*t*_*58*_ = -6.53, *p*< .001, also see Table 3). Still, the groups did not differ regarding positive or negative affect after the manipulation (positive affect scale: *t*_*58*_ = -0.98, *p* = .330; negative affect scale: *t*_*58*_ = -0.95, *p* = .348). The post hoc ratings (assessed at the end of the experiment) revealed that the groups differed regarding the perceived difficulty (*t*_*59*_ = -5.21, *p*< .001) but not regarding the affective evaluation (*t*_*59*_ = -0.03, *p* = .977) of the transcription task: the experimental group rated the task as more difficult compared to the control group.

**Table 3:** Manipulation and mood check.

Variable	Control	Experimental	Comparison			
	*n* = 29	*n* = 32				
	mean ± SD	mean ± SD	*df*	*t*	*p*	*Cohen’s d*
Manipulation check	2.71 ± 1.22	4.65 ± 1.07	58	-6.53	< .001	-1.688
Positive affect scale	27.00 ± 5.55	28.45 ± 5.87	58	-0.98	.330	-0.254
Negative affect scale	12.31 ± 2.85	13.26 ± 4.63	58	-0.95	.348	-0.244
Post hoc difficulty	1.72 ± 0.80	3.00 ± 1.08	59	-5.21	< .001	-1.336
Post hoc un-/pleasantness	2.93 ± 0.80	2.94 ± 0.95	59	-0.03	.977	-0.007

Manipulation check, sum score of the 3-item effort scale ranging from 1 (“low exerted self-control”) to 3 (“high exerted self-control”); positive affect scale of the PANAS ranging from 10 (“low positive affect”) to 50 (“high positive affect”); negative affect scale of the PANAS ranging from 10 (“low negative affect”) to 50 (“high negative affect”); difficulty rating ranging from 1 (“not at all”) to 5 (“very much so”); un-/pleasentness rating ranging from 1 (“very unpleasant”) to 5 (“very pleasant”) assessed at the end of the experiment. Groups were compared using unpaired t-tests (two-tailed).

### 3.2 Costs of self-control failure: Slower number comparison

**Fig 2:**
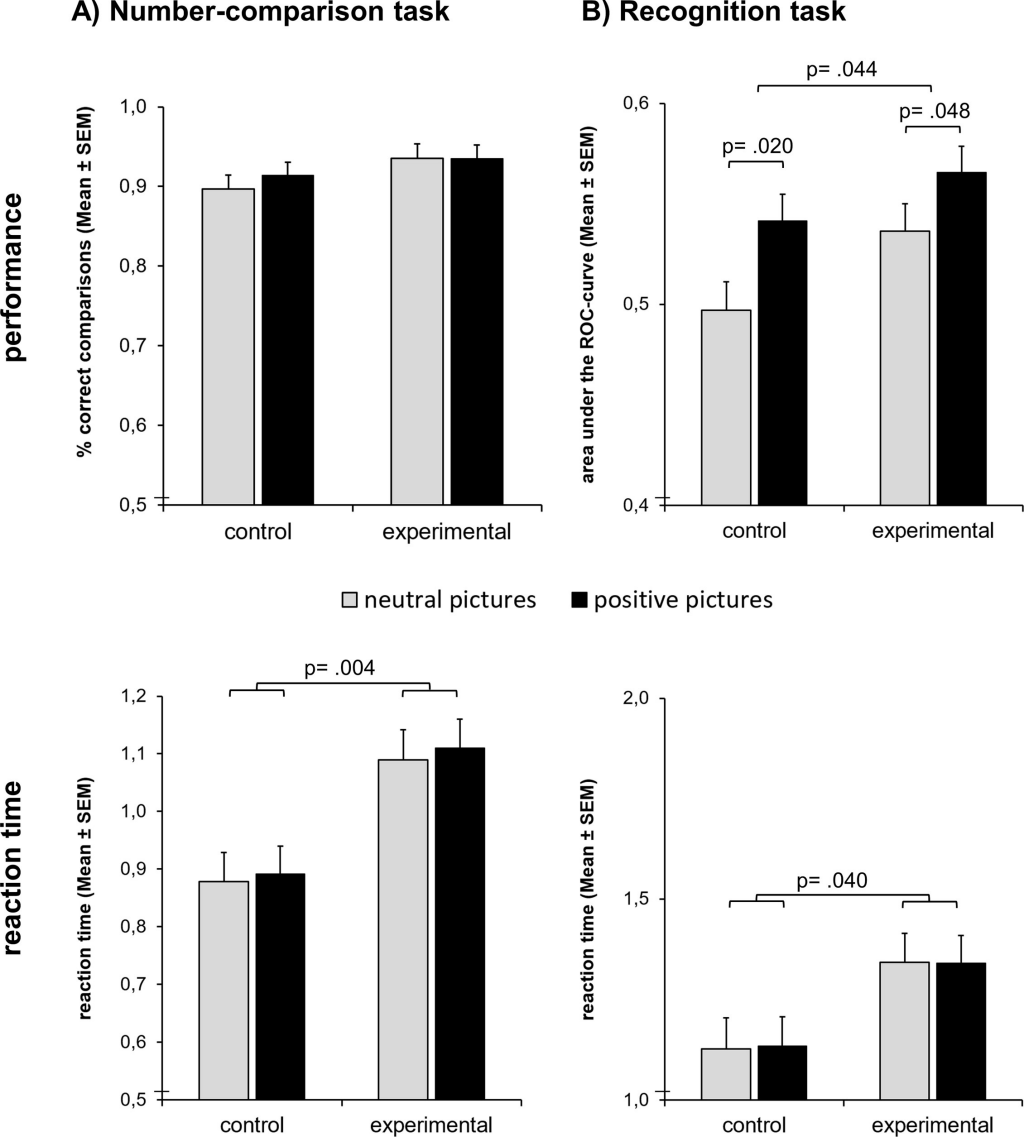
Results. A) The cost of self-control failure is reflected in an attention shift away from the number-comparison task: Participants of the experimental group who worked on consecutive self-control demanding tasks were slower in the number-comparison task than participants of the control group who had previously worked on the easy control task. B) The benefit of self-control failure is reflected in an attention shift toward the distracting pictures presented during the number-comparison task: After the delay, the experimental group was better at recognizing the incidentally encoded pictures than the control group. Reward-related pictures distracted both groups more than neutral pictures during the number-comparison task and were also better recognized later on. However, we did not find an interaction effect. Therefore, it remains unclear whether the exertion of self-control caused a motivation to specifically attend to reward-related distractors.

The hypothesis was that the exertion of effortful self-control would lead participants to shift their subsequent task priorities from cognitive labor to cognitive leisure, i.e. from exploitation to exploration. Therefore, we expected worse performance and longer reaction times in the number-comparison task in the experimental group. The ANOVA of the percentage of correct answers revealed neither a significant main effect of Group (*F*_*1;59*_ = 1.88, *p* = .176, *η*^*2*^ = .031), nor Distractor Valence (*F*_*1;59*_ = 0.63, *p* = .430, *η*^*2*^ = .011), nor an interaction of Group by Distractor Valence (*F*_*1;59*_ = 0.66, *p* = .420, *η*^*2*^ = .011, see Fig 2A). To enable better classification of the results, we have also calculated a sensitivity analysis for the ANOVA with the between-subject factor Group (control vs. experimental) and the within-subject factor Distractor Valence using g*power [37]. Given a targeted power of .95, α = .05, N = 61, and the empirical correlation of repeated measures of r = .617, the analysis would reveal medium effects of the within factor and the interaction (*f* = 0.205; *η*2 = 0.041) and large effects of the between factor (*f* = 0.422; *η*2 = 0.151). This fits the design principle of the task as a speed test, which means that effects should not show up in the performance but in the processing speed.

As expected the experimental group needed significantly longer to solve the tasks than the control group (main effect of Group: *F*_*1;59*_ = 9.24, *p* = .004, *η*^*2*^ = .135) indicating that they were distracted by the irrelevant pictures. Furthermore, by trend participants needed a little longer to solve the number-comparison task when positive as compared to neutral distractors were present (main effect of Distractor Valence: *F*_*1;59*_ = 3.12, *p* = .082, *η*^*2*^ = .050). There was no significant interaction of Group by Distractor Valence (*F*_*1;59*_ = 0.15, *p* = .703, *η*^*2*^ = .002). The sensitivity analysis showed that the ANOVA would reveal even small effects of the within-subject factor and small interaction effects (*r* = .969; *f* = 0.058; *η*^2^ = 0.003) but only large effects of the between-subject factor (*f* = 0.466; *η*^2^ = 0.178). The analysis suggests that an interaction effect would have been detectable even with the small sample size. There were no significant correlations between the number of correct answers and reaction times indicating that participants did not use different speed-accuracy trade-offs while dividing their attention between the number-comparison task and the distracting pictures.

### 3.3 Benefits of self-control failure: Better recognition of distractors

The hypothesis was that exploration of distracting pictures during the number-comparison task would result in better incidental encoding and subsequent recognition of these pictures. In fact, the experimental group showed better recognition performance than the control group as reflected in the ANOVA of the area under the ROC curve (main effect of Group: *F*_*1;59*_ = 4.25, *p* = .044, *η*^*2*^ = .067; see Fig 2B). Moreover, positive, reward-related pictures were significantly better encoded than neutral ones (main effect of Target Valence: *F*_*1;59*_ = 10.51, *p* = .002, *η*^*2*^ = .151). There was no significant interaction effect of Group by Target Valence (*F*_*1;59*_ = 0.44, *p* = .509, *η*^*2*^ = .007). The sensitivity analysis showed that the ANOVA would reveal medium effects of the within-subject factor and small interaction effects (*r* = .322; *f* = 0.273; *η*^2^ = 0.070) but only large effects of the between-subject factor (*f* = 0.382; *η*^2^ = 0.127). Since there was no indication of an interaction effect while at the same time the sensitivity of the analysis was quite high, it seems unlikely that the analysis missed a substantial effect due to a lack of power. The ANOVA of the reaction times showed that the experimental group needed significantly longer for the recognition decision than the control group (main effect of Group: *F*_*1;59*_ = 4.39, *p* = .040, *η*^*2*^ = .069). There was neither a significant main effect of Target Valence (*F*_*1;59*_ = 0.01, *p* = .907, *η*^*2*^< .001) nor an interaction of Group by Target Valence (*F*_*1;59*_ = 0.03, *p* = .863, *η*^*2*^ = .001). The sensitivity analysis showed that the ANOVA would reveal even small effects of the within-subject factor and small interaction effects (*r* = .911; *f* = 0.099; *η*^2^ = 0.010) but only large effects of the between-subject factor (*f* = 0.459; *η*^2^ = 0.174). There were no significant correlations between the area under the curve and reaction times indicating that participants did not use different strategies in the recognition task.

To check whether subjects who were slower during number comparison were subsequently better at recognizing the distracting pictures, we computed the correlation between the reaction times in the number-comparison task and the performance (area under the curve) in the recognition task. Indeed, there was a significant positive correlation indicating that participants who responded slower to the number-comparison task—and incidentally encoded the distractor pictures–showed better recognition performance in the unannounced recognition test (*r* = .352, *n* = 61, *p* = .005). Although the correlation does not prove causality, it is compatible with the interpretation that the participants shifted their attention from the numbers to the distractors.

### 3.4 Time-on-task

Previous research has shown that short active or passive breaks can improve the performance of self-control demanding tasks [52, 53]. Therefore, the question arises whether the participants of both groups had the same opportunity to rest and recover and whether this would affect the results of our experiment. In specific, one could suspect that the participants of the experimental group might have managed to sneak in unobserved breaks to rest.

As shown in Table 4, we compared time-on-tasks between the experimental and control group for each task in our experiment to ensure that the effects in our main analyses are not due to excessive resting times in previous tasks. We standardized the durations of the transcription task, the manipulation check and the affect ratings as well as for the relaxation video by using a stopwatch or computer respectively. After the transcription task (manipulation), the groups did not significantly differ in their invested time for reading the instruction and working on the demo trials preparing them for the following number-comparison task (mean difference = 5.9 s, *t*_59_ = -1.67, *p* = .100, *d* = 0.429). As expected, the experimental group needed significantly longer than the control group to actually complete the number comparison task (mean difference = 10.9 s, *t*_59_ = -3.01, *p* = .004, *d* = 0.747) confirming the analysis of the reaction times reported in section 3.2. This means that the effect of the manipulation mainly occurred in the actual task and not during the preceding instructions and demo trials.

**Table 4:** Time on task.

Task	Control	Experimental	Comparison			
	*n* = 29	*n* = 32				
	mean ± SD	mean ± SD	*df*	*t*	*p*	*Cohen’s d*
Transcription task	480 ± 0	480 ± 0	standardized duration			
Manipulation check	180 ± 0	180 ± 0	standardized duration			
Number comparison instr.	73.9 ± 13.6	79.8 ± 13.8	59	-1.67	.100	0.429
Number comparison task	203.0 ± 8.3	213.9 ± 18.6	59	-3.01	.004	0.747
Video instruction	8.2 ± 2.4	10.4 ± 4.6	59	-2.26	.027	0.580
Video watching	600 ± 0	600 ± 0	standardized duration			
Recognition instruction	104.9 ± 29.2	104.2 ± 22.8	59	0.12	.907	-0.030
Recognition task	349.9 ± 69.7	377.9 ± 77.7	59	-1.48	.145	0.379

The duration of the transcription task (manipulation), the manipulation check, and the video for relaxation were standardized and controlled by the experimenters using a stopwatch or computer respectively. All other durations were computed from the log files of PsychoPy. Durations are reported as mean and standard deviations in seconds.

After the number-comparison task, participants of both groups watched a 10 min long video to completely recover from the exertion of self-control. However, we also checked the time-on-task to look for residual effects of the manipulation. The participants of the experimental group needed significantly longer to read the instructions for the upcoming video (mean difference = 2.2 s, *t*_59_ = -2.26, *p* = .027, *d* = 0.580) although the difference between the group means was only 2.2 seconds. Moreover, the duration of the video was standardized to 10 min (600 s) and should have been long enough to allow for complete recovery in both groups.

The groups did not differ significantly regarding the time needed to read the instructions for the recognition task and working on the demo trials (mean difference = 0.7 s, *t*_59_ = -0.12, *p* = .907, *d* = -0.030) nor regarding the overall time to perform the recognition task (mean difference = 28 s, *t*_59_ = -1.48, *p* = .145, *d* = 0.379). This indicates that the video pause was sufficient to allow for recovery. However, as the analysis reported in section 3.3 revealed, the participants of the experimental group invested significantly more time in the old-new judgments and showed a significantly better recognition performance.

## 4. Discussion

### 4.1 Aim and summary

The present study aimed to shed some light on “the other side” of self-control failure, i.e. the benefits of self-control failure [3], and to investigate the assumptions of the ultimate account of the process model [21] and the reward responsivity hypothesis [22]. We developed a new experimental paradigm to test whether previous effortful self-control results in a shift of task priorities and attention away from a number-comparison task (cognitive labor) toward distracting pictures (cognitive leisure). In terms of the ultimate account, this would be a shift from exploitation to exploration. Moreover, we looked at the mechanisms behind this assumed shift of task priorities. In specific, we tested whether previous effortful self-control exertion just increases the tendency to explore task-irrelevant stimuli regardless of valence (ultimate account) or whether it only increases the response to positive, reward-associated stimuli (reward responsivity).

Our results indicate an attention shift away from the number-comparison task (cognitive labor): Participants of the experimental group answered slower in the number-comparison task than participants of the control group. Moreover, we found evidence for an attention shift toward the distracting pictures (cognitive leisure) presented during the number-comparison task: After the delay, the experimental group was better at recognizing the incidentally encoded pictures than the control group. Appetitive, reward-related pictures distracted both groups more than neutral pictures during the number-comparison task and were also better recognized later on. The lack of any interaction-effect highlights that the previous exertion of effortful self-control led people to shift their task priorities and attention to distracting stimuli, independently of whether the stimuli have a neutral or positive valence. This is indicative of exploration.

### 4.2 No proof for the reward responsivity hypothesis

The first assumption of the reward responsivity hypothesis is that effortful self-control is inherently aversive and results in a negative affect [22]. However, our manipulation check using the PANAS [48] did not show higher negative affect or lower positive affect in the experimental group as compared to the control group. Furthermore, in the post hoc rating, the participants of both groups rated working on the transcription task as equally unpleasant. In fact, the mean ratings almost hit the “neutral” scale center. Note that the manipulation check measuring the subjective effort and the post hoc rating measuring the subjective difficulty revealed large and highly significant differences between the conditions in the expected directions. Therefore, there is no indication that effortful self-control during the manipulation (transcription task) led to negative affect although the task was perceived as difficult and performing as effortful. This matches the results of two of our previous studies where we used the same manipulation and manipulation check and did not find higher negative affect after effortful self-control either [38, 46]. According to a meta-analysis [54], prior exertion of effortful self-control has at best a small effect on negative affect (*d*^*+*^ = 0.14, CI95 [0.06, 0.22], Cochran’s Q-test not significant) and no effect on positive affect. Moreover, numerous newer studies did not find any effect of effortful self-control tasks on negative affect either [e.g. 39–41, 43, 44, 55–58]. In summary, the premise of the reward responsivity hypothesis that exerting effortful self-control induces negative affect cannot be confirmed. Although the absence of evidence is not evidence of absence, the sheer number of studies that did not find more negative affect after the exertion of self-control puts a question mark behind this hypothesis. But it may be too early to completely shelve the hypothesis. Looking at the time course, it is noticeable that negative affect often occurs before tasks that require self-control and that a failure of emotion regulation can then lead to procrastination [11, 59]. The negative affect could thus be a consequence of anticipating self-control exertion rather than the consequence of the exertion of self-control itself. As the example of procrastination shows, some people regulate their negative affect by performing pleasurable, rewarding activities instead of the task requiring self-control. Further studies should investigate the time course of affect before, during, and after the exertion of self-control preferably using non-reactive measurement methods, e.g. facial electromyography, skin-conductance responses, or electroencephalography.

The second assumption of the reward responsivity hypothesis is that exerting effortful self-control increases the tendency to respond to reward-associated stimuli, proposedly to ameliorate the negative affect [22]. In the current experiment, we did not find any proof for this assumption, neither in the number-comparison task (cognitive labor) nor in the recognition of distracting pictures (cognitive leisure). This was reflected in the lack of an interaction effect of group and target valence in all analyses although the sensitivity of the analyses was sufficient. Moreover, a closer look at the data shows a tendency in the opposite direction: the participants of the control condition showed a slightly larger advantage for the recognition of reward-associated distractors relative to neutral distractors as compared to the experimental group. This cannot be due to a ceiling effect for reward-associated distractors in the experimental group because overall recognition performance was low and there would have been plenty of room for heightened reward responsivity to do its thing. This matches the results of one of our previous studies where exertion of effortful self-control did not amplify the appreciation of appetitive pictures [38]. Note, that in the current experiment and the previous experiment the stimuli elicited significant main effects of stimulus valence, either in recognition performance or in affective evaluation, respectively. This means that we could show that the reward-related stimuli work, but that the effects are independent of whether self-control was exercised beforehand.

In their comprehensive review, the authors of the reward responsivity hypothesis present an impressive amount of studies that by and large support their hypothesis [22]. In short, they highlight the effects of prior exertion of self-control on such different behaviors as consumption of unhealthy food, addictive substances, or gambling. Moreover, they report effects in the domain of economic decision-making and risk-taking as well as responses to positive stimuli or rewards. However, we are not aware of any study proving the whole causal chain of events, in specific proving that exerting effortful self-control causes a shift of task priorities and attention toward a pleasant task comprised of reward-associated stimuli. In the literature, we identified three challenges for the empirical test and lastly the falsifiability of the reward responsivity hypothesis: the distinction between self-control versus negative stimulation/frustration on the manipulation side, the measurement of subjective effort versus negative affect as intervening variables, and reward responsivity versus impulsivity on the dependent side.

The reward responsivity hypothesis is based on the assumption that exerting self-control is aversive and causes negative affect. However, in the literature, there are two types of manipulations that may be aversive for other reasons than exerting self-control. First, some studies used negative stimuli in the manipulation which are likely to cause negative affect irrespective of the instruction to inhibit the expression of affect. For example, one study used negative pictures from the International Affective Picture System [study 1 from 60]. Similar manipulations have been used in many studies [54]. Another type of manipulation of self-control encompasses an element of frustration which may also cause negative affect according to the classic frustration-aggression theory [61]. For example, two studies [e.g. 2b and 3 in 60] used a writing task very similar to the one we used in the present study except the participants were not required to copy a neutral text but write about a personal story. This manipulation creates a situation where the individual pursues a personal goal and is confronted with frustrating obstacles which—one might argue—seem very arbitrary and kind of mean. In our study, the participants were also confronted with a frustrating task (omit “e” and “n” while writing) but the task had less personal relevance. Moreover, the manipulation check revealed no effect of the manipulation on negative or positive affect despite significant effects on the self-control and difficulty ratings.

The reward responsivity hypothesis focuses on negative affect as an intervening variable between the exertion of self-control and the increase in reward responsivity. Many earlier studies which manipulated the exertion of self-control in a task also used measures of negative affect in the manipulation check. This was specifically done to exclude the explanation that effects are due to negative affect and not due to the high amount of self-control exerted [62]. However, recent studies which may be regarded as support for the reward responsivity hypothesis did not measure whether the manipulation of effortful self-control caused negative affect [e.g. studies in 60]. In our study, we measured negative and positive affect using a valid and reliable tool [48] but did not find any effect of the exertion of self-control on affect.

Finally, there are some ambiguities with dependent measures. The reward responsivity hypothesis proposes that people respond more strongly or more frequently to reward after exerting effortful self-control. However, in most studies, the participants do not actually get a reward but are merely confronted with stimuli associated with reward [60, 63], fake money [60], points [64], or hypothetical rewards. In our study, participants were more distracted by positive pictures in the number-comparison task but this was independent of the amount of previously exerted self-control. Moreover, our participants recognized reward-related pictures better than neutral ones but again, this effect was independent of the amount of previously exerted self-control. However, participants who exerted more effortful self-control showed a greater tendency to explore and encode task-irrelevant pictures irrespective of valence. This is supported by several other studies investigating the effect of effortful self-control on subsequent economic decision-making [65–67]. Although these studies used hypothetical monetary rewards, the results do not indicate an increase in reward responsivity after self-control exertion but increased delay discounting or impulsivity. To our best knowledge, there is one study that not only measured an attention shift to reward-associated stimuli but also actual reward (food) consumption [68]. The authors investigated restrained and unrestrained hungry eaters who either performed the self-control demanding or control variant of the e-crossing task and subsequently worked on a dot-probe task with reward or self-control associated distractors and finally had the chance to eat sweets ad libitum in a degustation task. The results showed no effect: neither the diet status (restrained/unrestrained) nor the self-control manipulation had any impact on the attentional bias for rewards nor the actual consumption of the rewarding sweets. In summary, the empirical evidence in the literature in favor of the reward responsivity hypothesis maybe not so overwhelming as it seems at first sight. Moreover, some studies yielded results contradicting or at least not confirming the hypothesis [38, 68–73].

### 4.3 Confirmation of the ultimate account of the process model

The process model of self-control [21] is tied in with the observation that humans like many other foraging animals prefer an optimal trade-off between exploitation and exploration. Our experiment supports the idea that repeatedly exerting effortful self-control increases the tendency to stop exploiting the number comparison task (cognitive labor) and to begin exploring the distracting stimuli (cognitive leisure). This behavior was reflected in the worse performance of the experimental group in the number-comparison task and better performance in the recognition task as compared to the control group. However, the effect was not specific for reward-associated distractors but also occurred with neutral distractors. Therefore the question arises, which form of exploitation and exploration took place. The process model adopts a definition of exploration and exploitation that has its origin in reinforcement learning, foraging, and decision-making research [28]. In this context, the individual has to decide between exploiting a known source of reward or exploring other potential sources of reward. However, exploration and exploitation have been investigated in multiple disciplines like vision science, ecology, machine learning, economics, and management [29]. As a result, forms of utility other than reward have been incorporated in the definition of exploration and exploitation: A person can keep on exploiting information from a stimulus or decide to explore other stimuli to gather knowledge. Or a person can keep on optimizing performance in one task or explore other tasks or actions to acquire skills. Both knowledge and skills have an inherent utility for the person just like a proper reward. In the context of the current experiment, working on the number comparison task could be considered a form of reward exploitation: The participants worked on the task to comply with the instructions of the experimenters, to obtain course credit, and to make a good impression in comparison with fellow participants, i.e. they exploited a known source of reward. On the other hand, disengaging from the number-comparison task and attending to the distracting pictures could be considered a form of reward exploration: The participants searched for new sources of reward by exploring the task-irrelevant distractors. In other words, they changed their priorities from exploiting cognitive labor to exploring cognitive leisure. However, the participants were rewarded for their participation independent of their performance in the number comparison task. Moreover, the increased tendency to explore the distracting pictures after repeated exertion of self-control was not specific for reward-associated pictures but also occurred with neutral pictures. If the assumption of the ultimate account of the process model and the reward responsivity hypothesis were correct, we would have expected a reward-specific effect, i.e. the participants of the experimental group should have shown a greater tendency to specifically explore reward-associated distracting pictures. On the other hand, it is important to remember that exploration is not the same as attending to a reward or even approaching a reward. That would be exploitation, as a reward in sight is a known source of reward and the reward can simply be collected. Rather, exploration is the search for unknown or potential sources of reward and this can mean attending to neutral as well as reward-associated distractors alike. Moreover, activities of cognitive leisure like looking at pictures on social media sites usually have a rewarding characteristic [74]. More importantly, the participants not only explored potential sources of reward but also gathered knowledge about the environment, i.e. about stimuli that were irrelevant at the time of encoding but turned out to be useful in the unannounced recognition test. The knowledge acquired during exploration had an inherent utility. This might be an important hidden benefit of self-control failure.

In the light of our results, the paradox of self-control becomes a little bit less paradoxical and the question of why evolution has not endowed us with perfect self-control might be a little more clarified: Self-control during task engagement–exploitation–bears as much opportunity costs as self-control failure–exploration–bears the chance to discover new sources of reward [27, 32]. Moreover, self-control failure–exploration–enables the individual to learn and to collect information about the environment [32]. There is a German proverb that nicely captures this idea: "Detours increase local knowledge.". Exerting self-control to focus on a task or cognitive labor is like staring through a narrow tube. The goal is clearly visible but the surrounding world is not. Self-control failure broadens the view and helps us to be aware of things in the environment that may become relevant later. This benefit of self-control failure is not very obvious but very adaptive. This benefit of self-control failure might be relevant for a vast amount of behaviors as the review “exploration versus exploitation in space, mind, and society” by Hills et al. [32] illustrates.

An open question is, how the mind implements exploration. In the literature two types of exploration are discussed: directed and random exploration [75]. In the current experiment directed exploration would be the targeted search for reward-associated stimuli or at least stronger responses to reward-associated stimuli as the process model and the reward responsivity hypothesis assume. However, the participants in the experimental condition who repeatedly exerted self-control showed an increased tendency to explore distracting pictures independent of the valence or reward-relatedness of the pictures. Therefore, our results are evidence for the second type of exploration, i.e. random exploration. Random exploration does not depend on a specific motivation to seek reward. Instead, there are alternative mechanisms that might even rely on less processing capacity, namely a down-regulation of attention and an up-regulation of impulsivity. There is evidence for both mechanisms. First, Englert et al. [76] investigated the effect of anxiety and prior self-control exertion on a perceptual-motor task that required selective attention. They found that dart-throwers under pressure who had previously engaged in a self-control demanding task were less able to fixate on the dart target and hit the bull’s eye. Similar results were found with basket-ball throwers [44]. Moreover, Garrison et al. [77] found in a very large sample that exercising self-control caused worse attention performance in subsequent Stroop or Attention Network Tests. This result was supported by an eye-tracking study by Englert et al. [78] who found a similar effect using an attention control video. Impulsivity as an alternative mechanism is evident in another set of studies. For example, Lin et al. [79] also investigated the effect of prior effortful self-control on the performance in the Stroop test. However, using a drift-diffusion model the authors showed that the worse performance was due to reduced response caution (i.e. impulsivity) rather than inhibitory control. Moreover, studies on economic decision-making show increased choice impulsivity after exerting effortful self-control [65–67]. In summary, these studies show that exerting effortful self-control reduces attentional control and increases impulsivity thereby fostering task disengagement and explorative behavior. However, further studies are needed to decide which mechanism the mind uses to foster exploration after prolonged or repeated exertion of self-control.

### 4.4 Why does self-control failure exist?

The topic of self-control failure has attracted many researchers from multiple disciplines. Although this shows how relevant the topic is in different areas like education, work, or health behavior, it comes with the drawback that there are multiple alternative theories that can not all be tested by a single experiment. In general, two perspectives can be taken to answer the question of why self-control failure exists: A teleological perspective looks at self-control failure from the end and asks for the advantageous and disadvantageous consequences of self-control failure, i.e. “What is it good for?”. A mechanistic perspective looks at self-control failure from the beginning and asks for the cognitive, affective, and motivational mechanisms of self-control failure, i.e. “What is the cause?”. The current experiment mainly adds to the first perspective. The teleological perspective is somewhat agnostic about the causes of self-control failure but focuses on the consequences, i.e. the hidden benefits of self-control failure. Our experiment showed that a benefit of self-control failure is the collection of information about the environment and the potential discovery of new sources of reward. However, the process model and the reward responsivity hypothesis make specific assumptions from the mechanistic perspective. Since we have found no evidence that the exercise of self-control leads to negative affect or higher reward sensitivity, the question arises as to what mechanisms then cause self-control failure. There are three classes of theories about the causes of self-control failure assuming that, first, people can not control themselves, second, people just think they can not control themselves, or third, people can but do not want to control themselves.

The first class of theories borrows from the physiology of performance and fatigue. In the first version of their theory, Baumeister and colleagues assume that self-control works like a muscle [1, 62]. Accordingly, one needs a limited, exhaustible, domain-spanning resource—often called willpower—to exercise self-control. Repeated or prolonged exercise of self-control leads to a temporary depletion of this resource—“ego depletion”—so that the person can no longer exercise self-control. On the one hand, it was questioned from an empirical perspective whether the claimed "ego-depletion effect" exists at all [for an overview see 23], and on the other hand, the assumption of an exhaustible resource was criticized from a theoretical perspective, e.g. by the Inzlicht and colleagues in their process model [21, 80].

The second class of theories can be summarized in a quote often attributed to Henry Ford: “Whether you think you can, or you think you can’t—you’re right.”. Job and colleagues assume that what matters is not whether or not the capacity for self-control is depleted, but what intuitive theory the person has about self-control [81]. Their experiments [82, 83] showed that individuals who believe that self-control is a limited resource will behave that way, and their self-control will fail sooner or later the longer they exercise self-control. On the other hand, individuals who believe that self-control is unlimited will behave that way and continue to successfully perform tasks requiring self-control. These data are incompatible with the first class of theories that see the capacity for self-control as limited. Another theory from the second class of theories is the schema activation model proposed by Bertrams and colleagues [84]: A fatigued/decreased vitality schema could lead the individual to disengage from a task and explore stimuli associated with recreation and restoration of vitality. In our experiment, effortful self-control in the transcription task could have triggered a fatigue schema causing task disengagement in the number-comparison task and exploration of the distracting pictures. However, we neither measured intuitive theories about self-control nor schema activation.

The third class of theories assumes that the person could control him/herself, but with the continued or repeated exercise of self-control is less and less motivated to continue exercising self-control. The process model and the reward responsivity hypothesis discussed above are examples of this class of theories. The reward responsivity hypothesis does predict that the exertion of self-control leads people to be motivated to seek reward and to shift their attention. But ultimately, we only found evidence of an attention shift, and this shift was independent of whether the distractors were related to reward. A significant interaction effect of Group and Distractor Valence would have supported the interpretation that approach motivation (reward-seeking) is the intervening process. But the actual results and the sensitivity analysis cast some doubt on this. Further studies should clarify whether exercising self-control only leads to a shift of attention away from tasks associated with cognitive labor or also toward alternative pleasant activities, as in procrastination. Another theory of this class is the regulatory focus theory [85] in combination with recent experiments on boredom as a cause of self-control failure [86, 87]: The idea is that participation in the experiment, especially in the experimental condition, might have been boring or aversive and therefore caused a regulatory prevention focus, i.e. a tendency to avoid further boredom or negative affect. In our experiment, this could have caused an attentional disengagement from the number-comparison task rather than reward-seeking. Although we cannot completely rule out this explanation, the affect ratings after the manipulation and the post hoc affect rating showed no differences between the experimental and control group. Therefore, the tendency to avoid negative states should have been the same in both groups and could hardly explain the main effects. Yet another theory of this class is the motivational control theory of cognitive fatigue as proposed by Hockey [88]. Interestingly, this theory stems from another research area, namely the investigation of mental fatigue [89], but it has some striking similarities to the process model which originates from the field of ego depletion research [21]. Both theories assume that prolonged exertion of self-control can result in a motivation to shift task priorities from labor to leisure (process model) or from current activated goals to alternative goals for the control of action (motivational control theory). However, the motivational control theory also specifies when and how motivation can help to compensate for fatigue to keep performance high: In two studies Herlambang and colleagues show that extrinsic or intrinsic motivation can prevent a decline in performance in cognitively demanding or self-control demanding tasks. In the first study [90], the authors confronted the participants with 14 blocks of a working memory task which lasted 2.5 hrs in total and were accompanied by distracting video clips. In an alternating pattern, half of the blocks were rewarded to foster extrinsic motivation. The results showed, that the participants reported becoming increasingly fatigued but performance dropped only in the non-rewarded blocks. In other words, when motivation was high, the participants were able to compensate for subjective feelings of fatigue and keep performance high. In the second study [91], the authors used a similar experimental setting but manipulated intrinsic motivation. The participants were working on 14 blocks of Sudoku puzzles which could either be solved until completion (high motivation) or were constantly changed so that participants had to start from scratch multiple times (low motivation). Again the results showed, that the participants became fatigued over time but high intrinsic motivation prevented a drop in performance. Moreover, the authors measured higher mental effort and lower distraction in the high motivation blocks which might explain how motivation can help to compensate for subjective fatigue. However, one rather philosophical question remains: Is it justified to speak of self-control “failure” when the reason is “not wanting”, i.e. a lack of motivation?

### 4.5 Strength and limitations

The main strength of the current experiment is the new experimental paradigm that can capture both the attention shift away from a self-control demanding task as well as an attention shift toward distracting stimuli. In addition, the paradigm also captures the benefits of self-control failure which were neglected by previous studies. Another strength of the experiment is the manipulation. In the current and the previous [38, 46] experiments, we used a proven manipulation task that reliably produced aftereffects of self-control in many studies [39–45, 92]. Moreover, we conducted comprehensive manipulation checks encompassing repeated measurements of subjective effort and positive and negative affect as well. In addition, we employed multiple, rigorous experimental control procedures of nuisance variables, e.g. standardized stimuli and balancing of presentation locations. Finally, we standardized the timing of the manipulation, manipulation checks, affect ratings, and the video pause and logged the time-on-task in all other tasks including the instructions and demo trials. The analysis of the times-on-task confirms the main analyses, in specific the effect of the exertion of self-control in the experimental group as well as the sufficiency of the video pause to undo the effect before the recognition task. Furthermore, there was no indication that participants in the experimental group may have taken “unofficial” rest breaks to recover prematurely from exercising self-control before the “official” video break began.

During the manipulation, we used a self-control demanding variant of the transcription task in the experimental group that can possibly create frustration and negative affect. Although we found no significant differences in positive or negative affect between the experimental and control groups, further studies are needed to investigate the role of affect as a mediating variable. For example, affect could be explicitly manipulated as in the studies by Bertrams and Englert [41, 43].

A possible limitation could be that the number-comparison task was rather easy. On the one hand, one might criticize that this resulted in a ceiling effect regarding the performance, as the rate of correct answers was about 90%. On the other hand, this helped to ensure that the interesting effect could only be reflected in the response times and not in different speed-accuracy trade-offs. Moreover, the task was not designed to require much working memory as in classical dual-task paradigms but to capture the attention shift between the number-comparison task and the distractor pictures. The participants had to exert self-control to focus attention on the numbers and resist the temptation to explore the distracting pictures.

We acknowledge that exploration of the distracting pictures is a very reduced, “lab-like” form of exploration. The task was inspired by visual foraging tasks (visual search) where participants also have to decide where to look for targets. This form of visual exploration has been shown to be guided by rewards associated with targets [93]. In the number-comparison task, both stimuli–number pairs and distractor picture–were presented simultaneously for 500 ms. Afterward, the distractor disappeared and only the number pair remained on the monitor until the participant responded. This confronted the participants with the necessity to choose where to focus their attention first, the task or the distractor. Attending to the picture first, elaborating its semantic and affective content, and encoding it in memory are considered some kind of exploration. This resembles an everyday situation in which we need self-control: Working intently on a task at the computer without being distracted by social media, notifications, and potentially interesting content. In future studies, the paradigm could be enriched to give participants more options to actively explore distracting stimuli like videos or notification lights. Furthermore, eye-tracking could be used to measure visual exploration behavior.

Like most studies, we used reward-associated stimuli instead of actual rewards. On the one hand, we have previously shown that looking at our positive stimuli creates emotional responses with positive valence [38], and therefore looking at these pictures (cognitive leisure) instead of comparing numbers (cognitive labor) might be considered rewarding. On the other hand, a picture of a reward is not the reward itself and might not be sufficient to elicit approach motivation or increase reward responsivity. Therefore, future studies should compare conditions employing reward signals versus actual rewards. For example, it would be interesting to manipulate the amount of reward for working on the self-control demanding task and the amount of reward that is coupled with the distractors. This way it would be possible to better distinguish between a motivation to shift away from the self-control demanding task and a motivation to shift toward alternative stimuli or actions.

The main purpose of this article is not to amass empirical evidence to enforce a final decision on the process model or the reward responsivity hypothesis. Rather, we regard this article as an opportunity to offer a new experimental paradigm to the research community and stimulate further research and discussion. Therefore, we adhere to open science principles and provide our data as well as the paradigm for download (see supporting information). We hope that this will inspire others to repeat and refine our experiment.

### 4.6 Take-home message

Although self-control and willpower have been proclaimed a superpower [94], there are also hidden benefits of self-control failure. Our experiment showed that prior exertion of effortful self-control causes a shift of task priorities from the self-control demanding task (cognitive labor) to distracting stimuli (cognitive leisure), i.e. a tendency to exploit less and explore more. This was reflected in worse performance in the self-control demanding number-comparison task and better performance in the later recognition of the distracting pictures. The hidden benefit lies in the exploration of the distractors during goal pursuit, i.e. the collection of information about the environment and the potential discovery of new sources of reward. Detours increase local knowledge.

## Supporting information

S1 File Data. (ZIP)

S2 File Software. (ZIP)
