# Supplementary material for: Detours increase local knowledge—Exploring the hidden benefits of self-control failure
Source: PLoS One. 2021 Oct 1;16(10):e0257717. doi: 10.1371/journal.pone.0257717 (PMC8486128; doi:10.1371/journal.pone.0257717)

## Slide 1
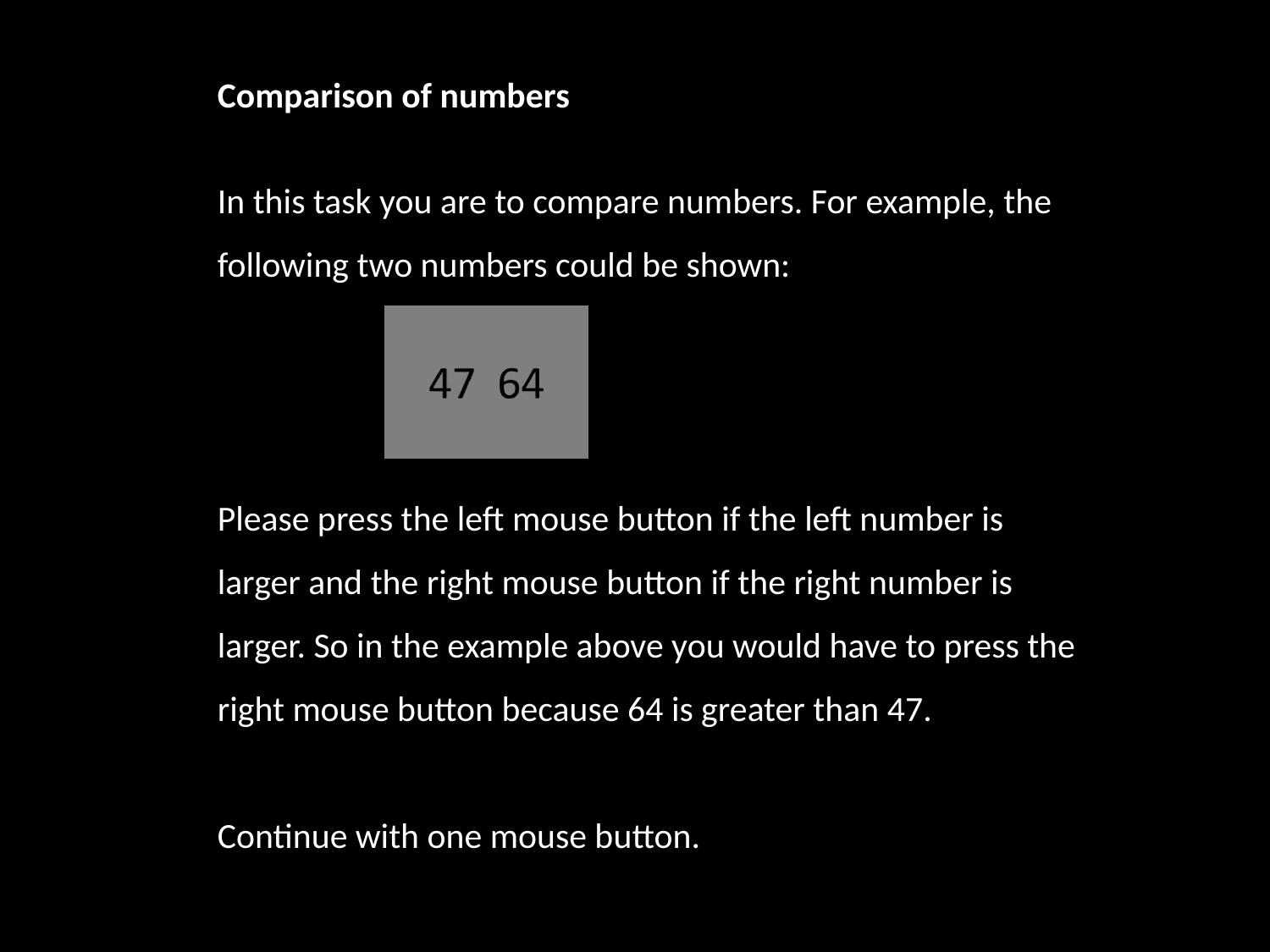

Comparison of numbers
In this task you are to compare numbers. For example, the following two numbers could be shown:
Please press the left mouse button if the left number is larger and the right mouse button if the right number is larger. So in the example above you would have to press the right mouse button because 64 is greater than 47.
Continue with one mouse button.

## Slide 2
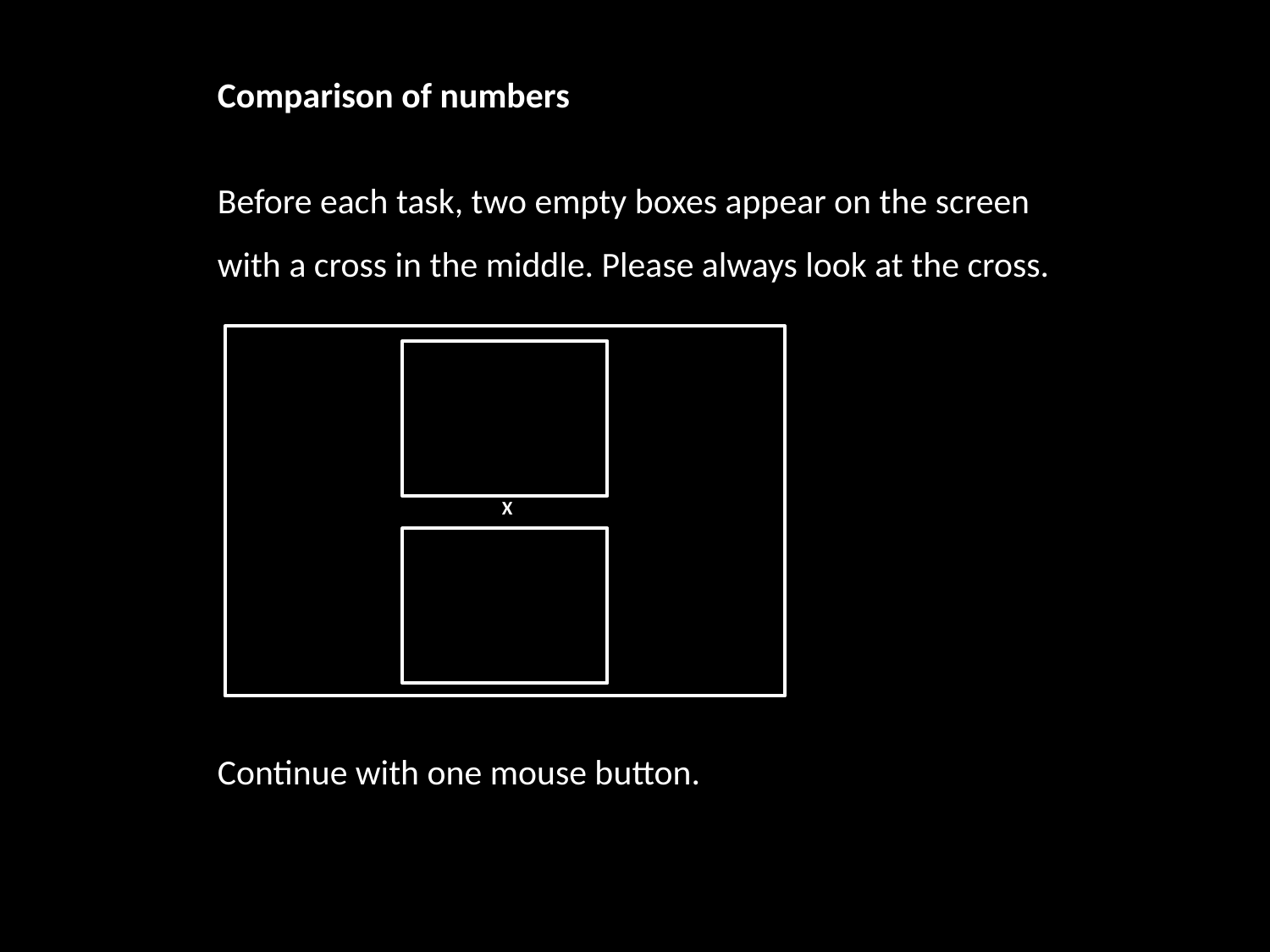

Comparison of numbers
Before each task, two empty boxes appear on the screen with a cross in the middle. Please always look at the cross.
Continue with one mouse button.
X

## Slide 3
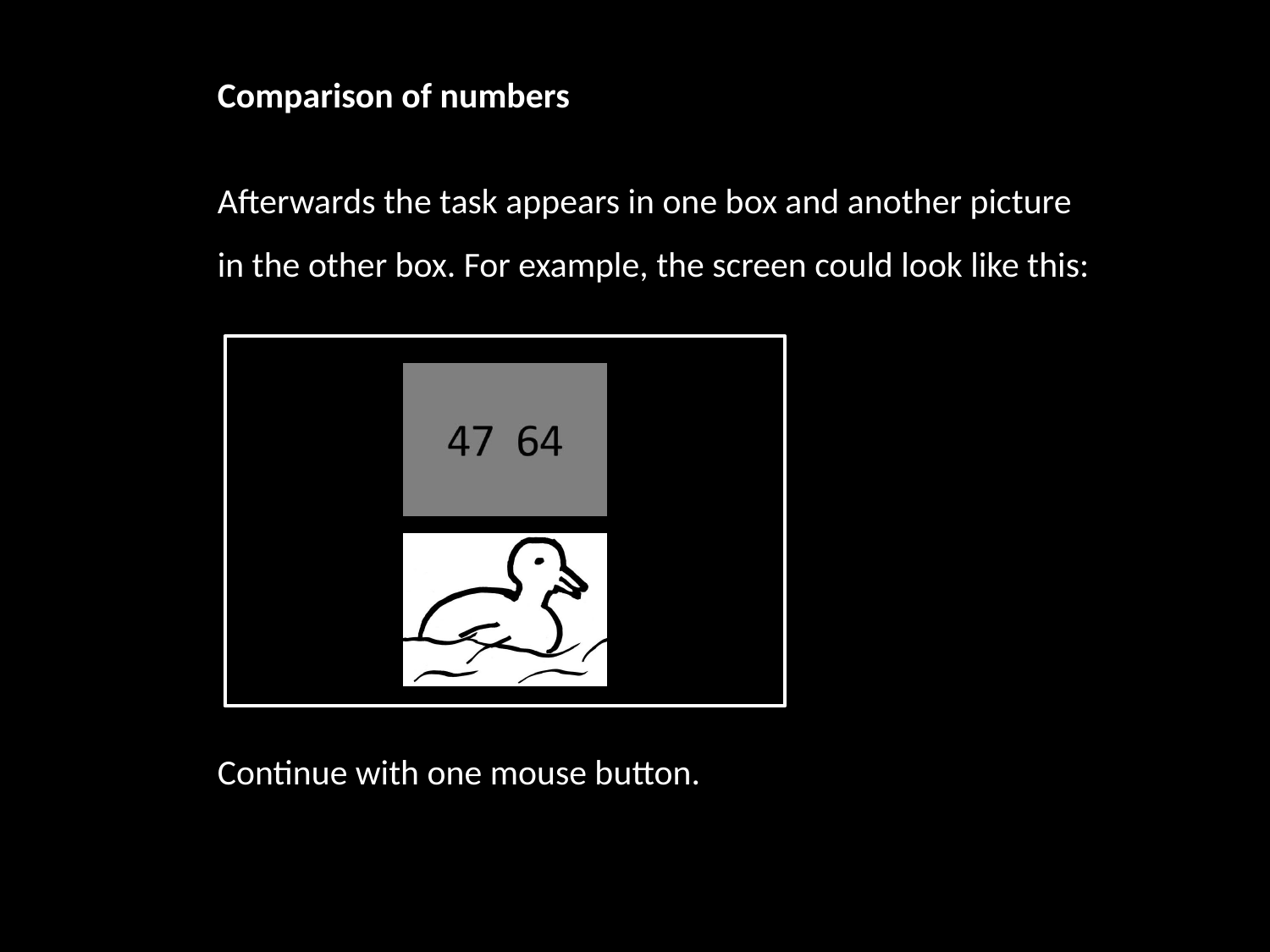

Comparison of numbers
Afterwards the task appears in one box and another picture in the other box. For example, the screen could look like this:
Continue with one mouse button.

## Slide 4
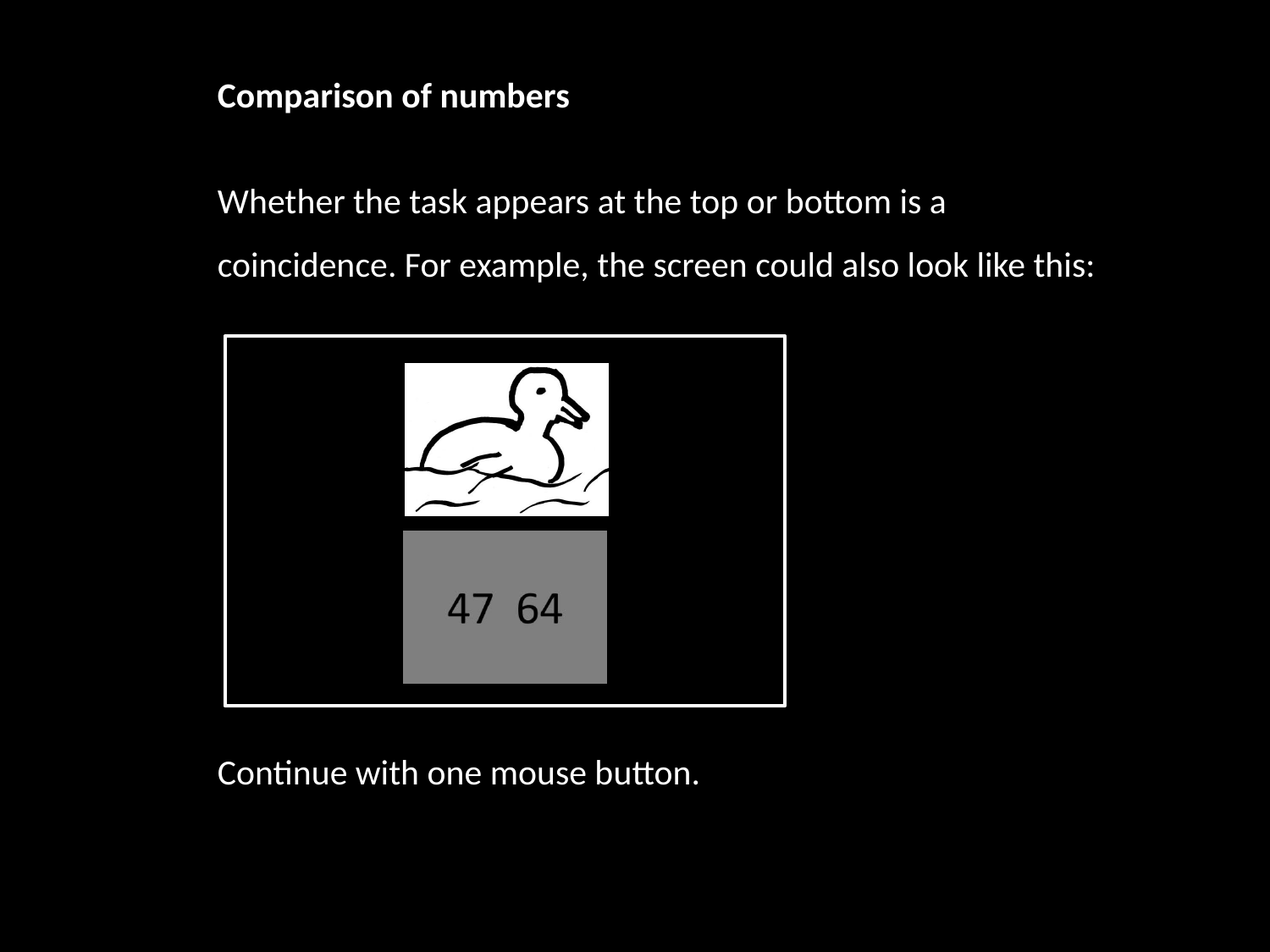

Comparison of numbers
Whether the task appears at the top or bottom is a coincidence. For example, the screen could also look like this:
Continue with one mouse button.

## Slide 5
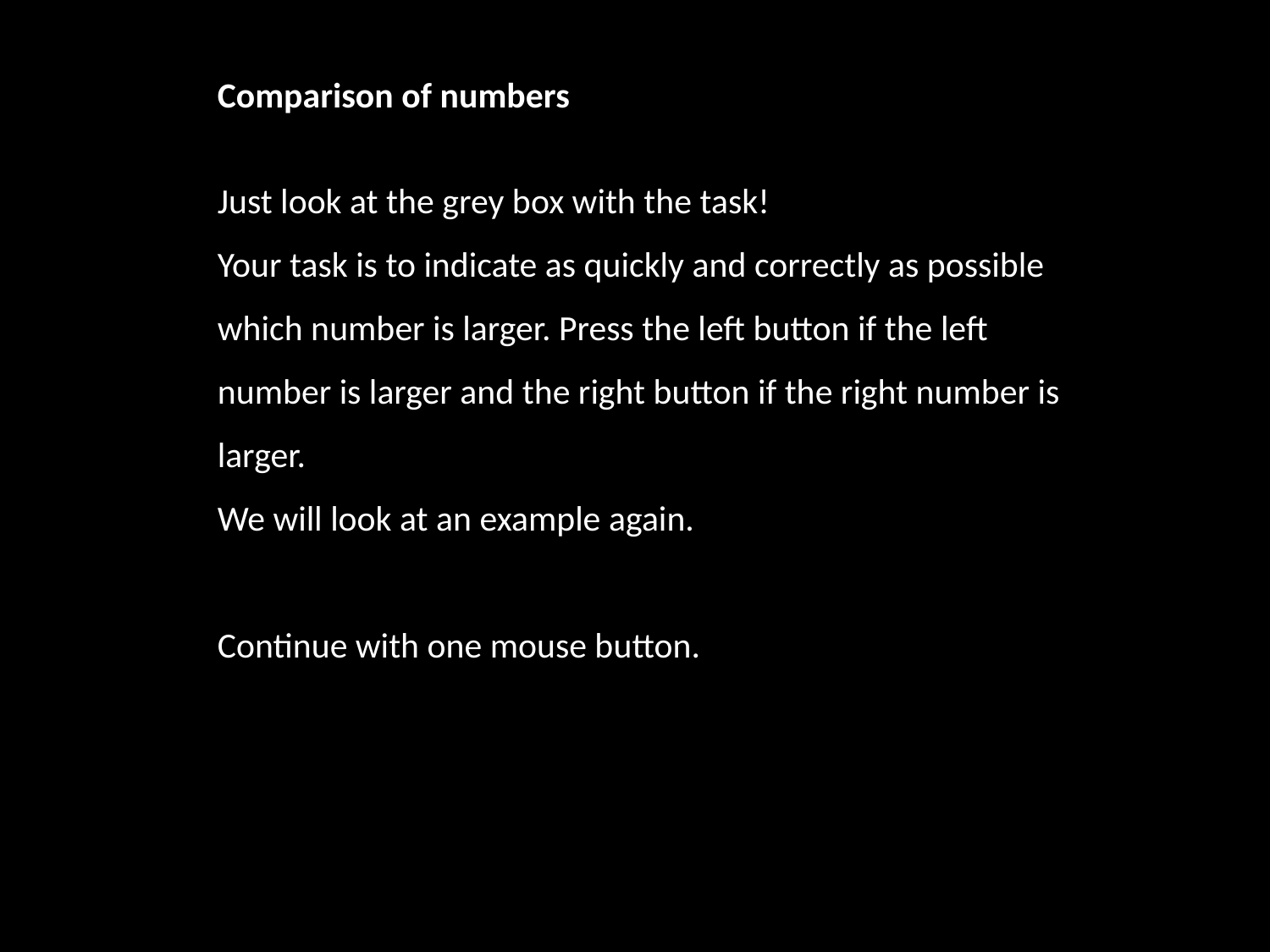

Comparison of numbers
Just look at the grey box with the task!
Your task is to indicate as quickly and correctly as possible which number is larger. Press the left button if the left number is larger and the right button if the right number is larger.
We will look at an example again.
Continue with one mouse button.

## Slide 6
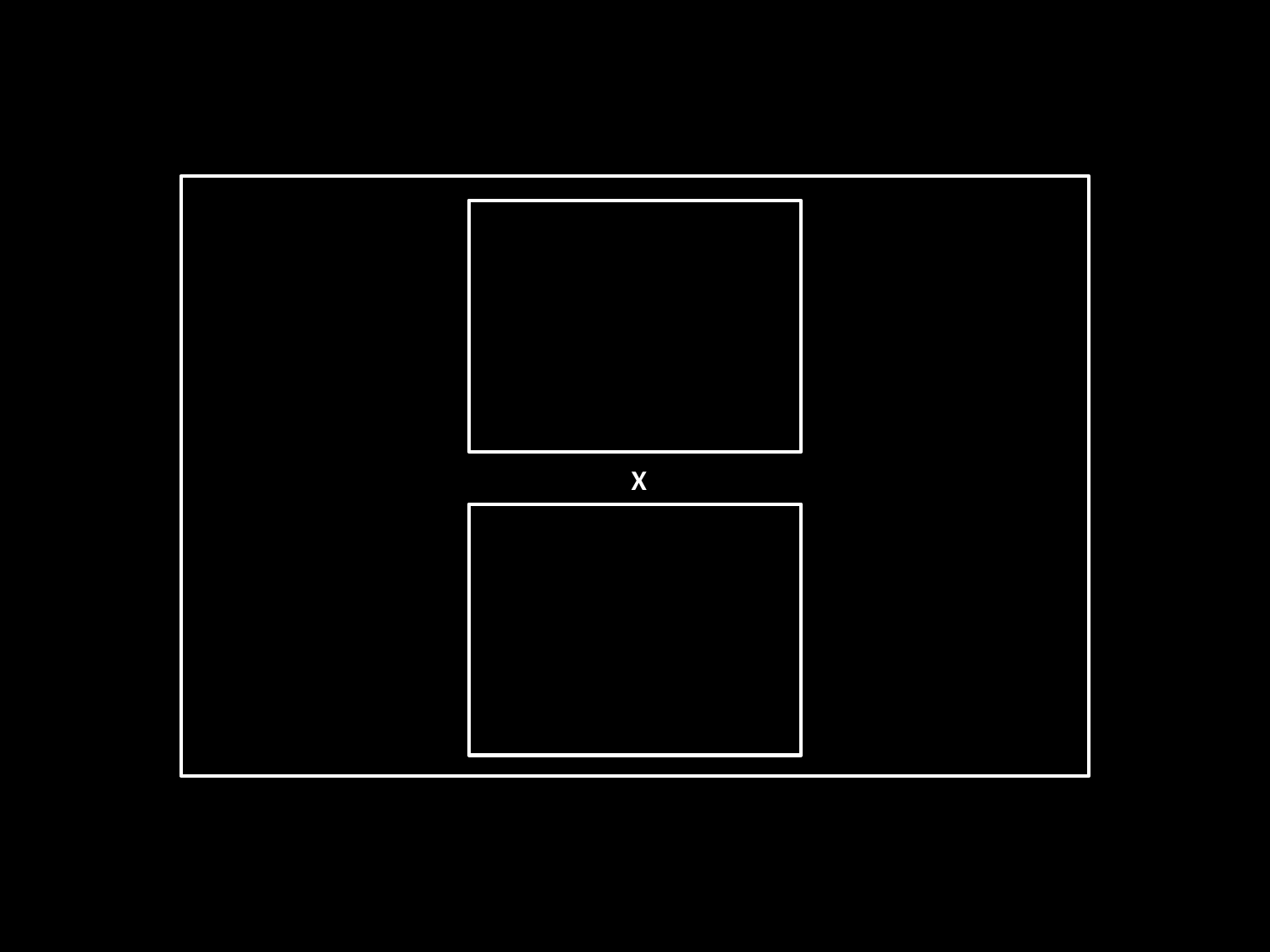

X

## Slide 7
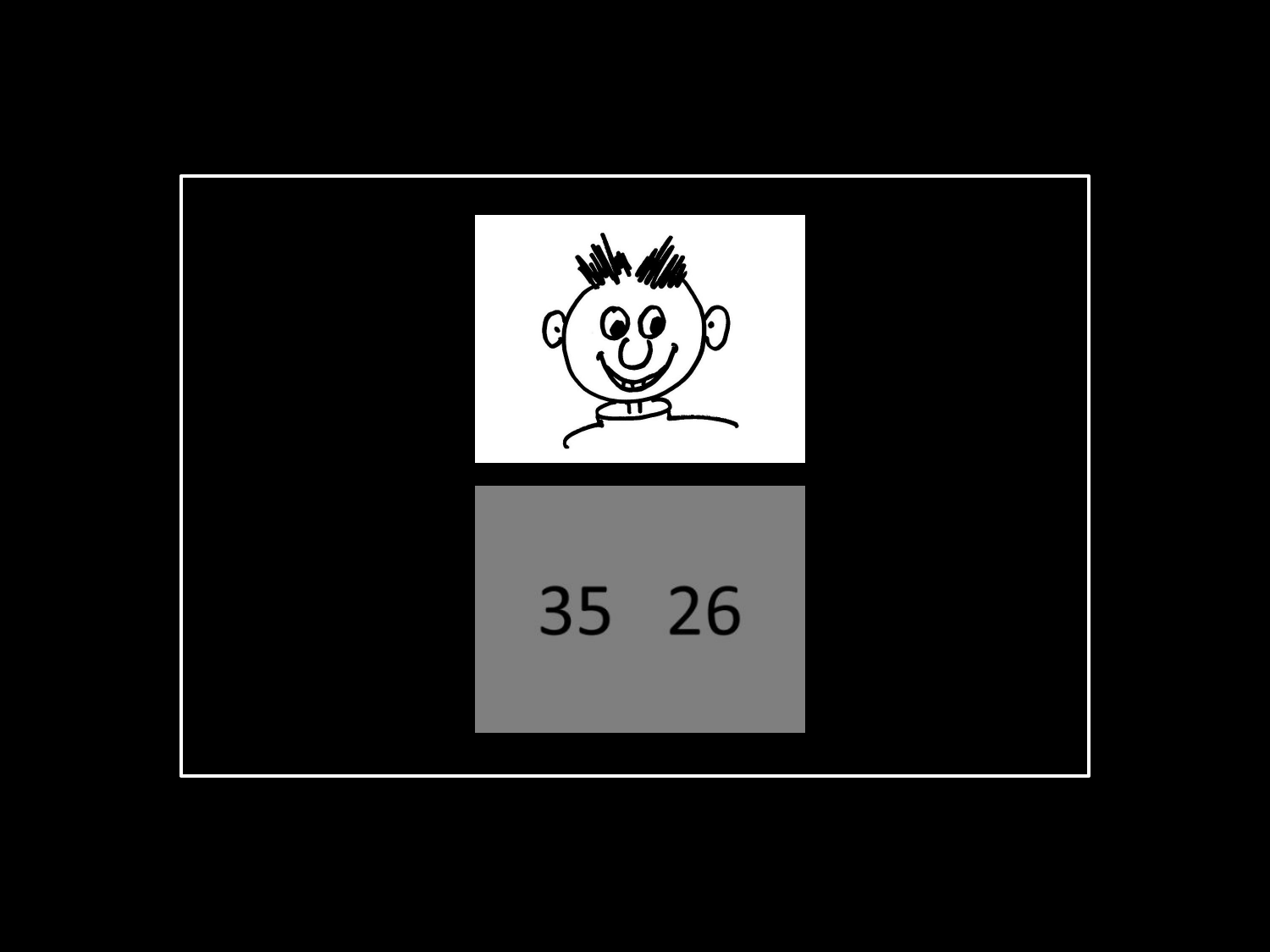

X

## Slide 8
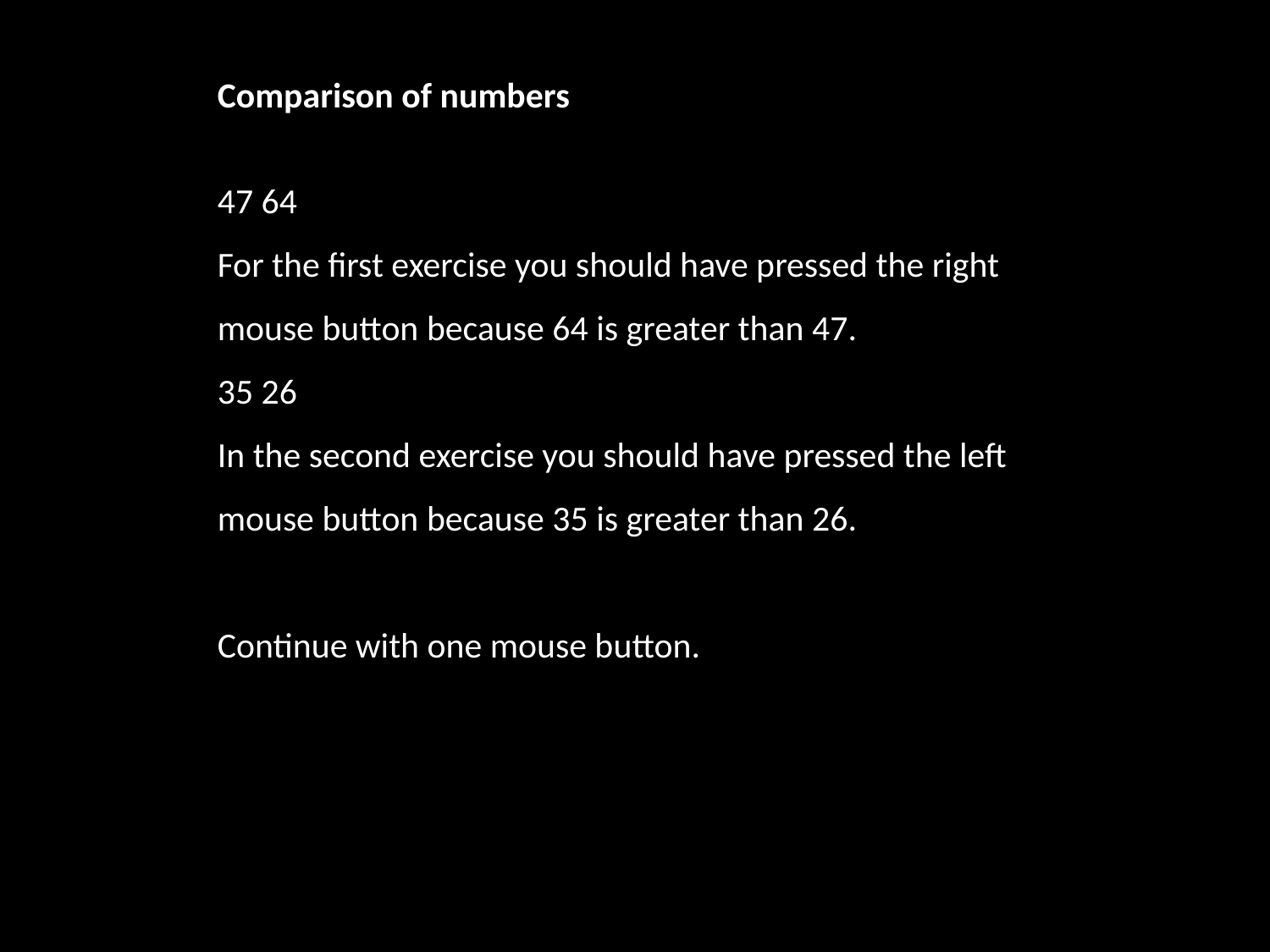

Comparison of numbers
47 64
For the first exercise you should have pressed the right mouse button because 64 is greater than 47.
35 26
In the second exercise you should have pressed the left mouse button because 35 is greater than 26.
Continue with one mouse button.

## Slide 9
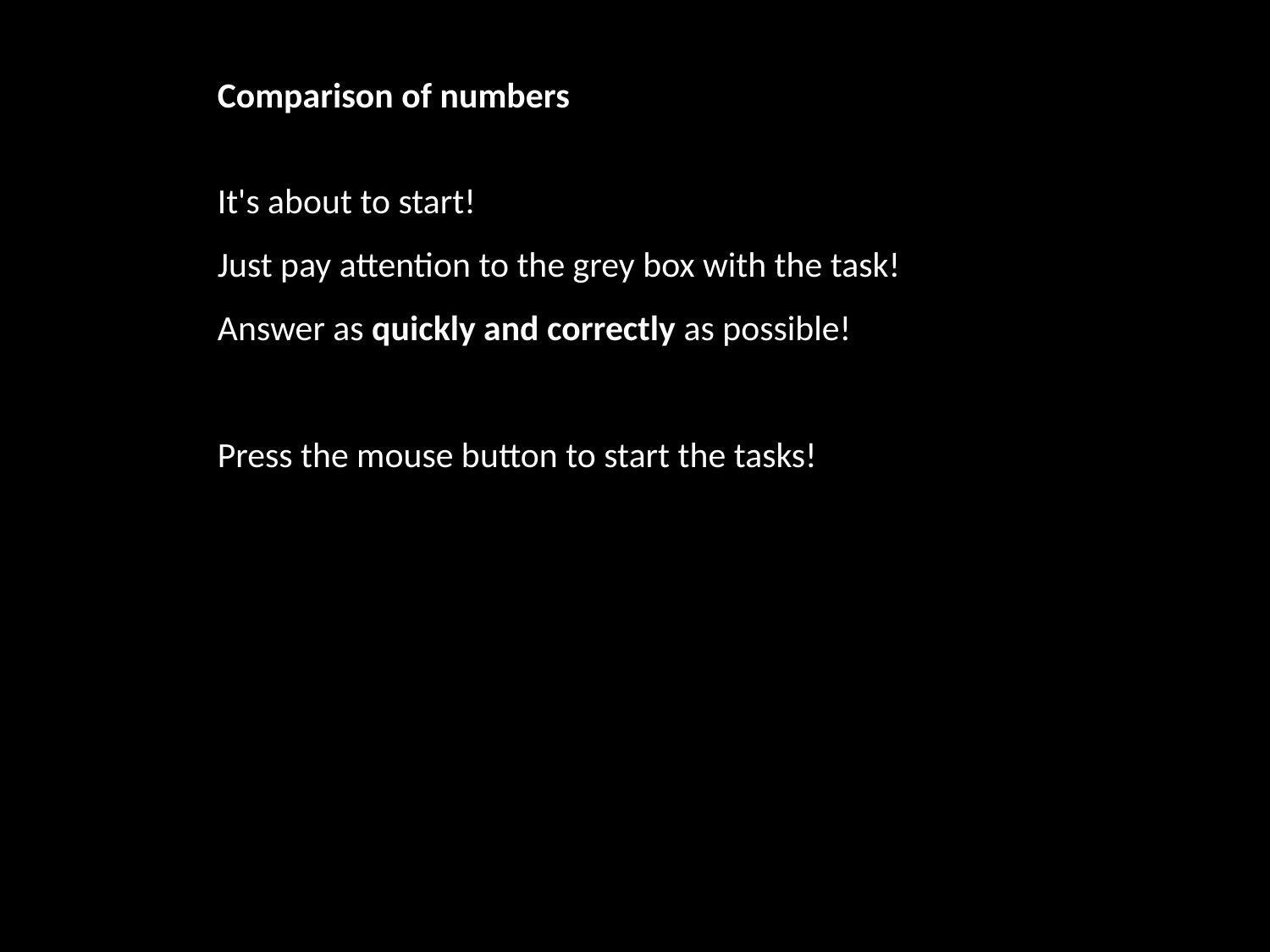

Comparison of numbers
It's about to start!
Just pay attention to the grey box with the task!
Answer as quickly and correctly as possible!
Press the mouse button to start the tasks!

## Slide 10
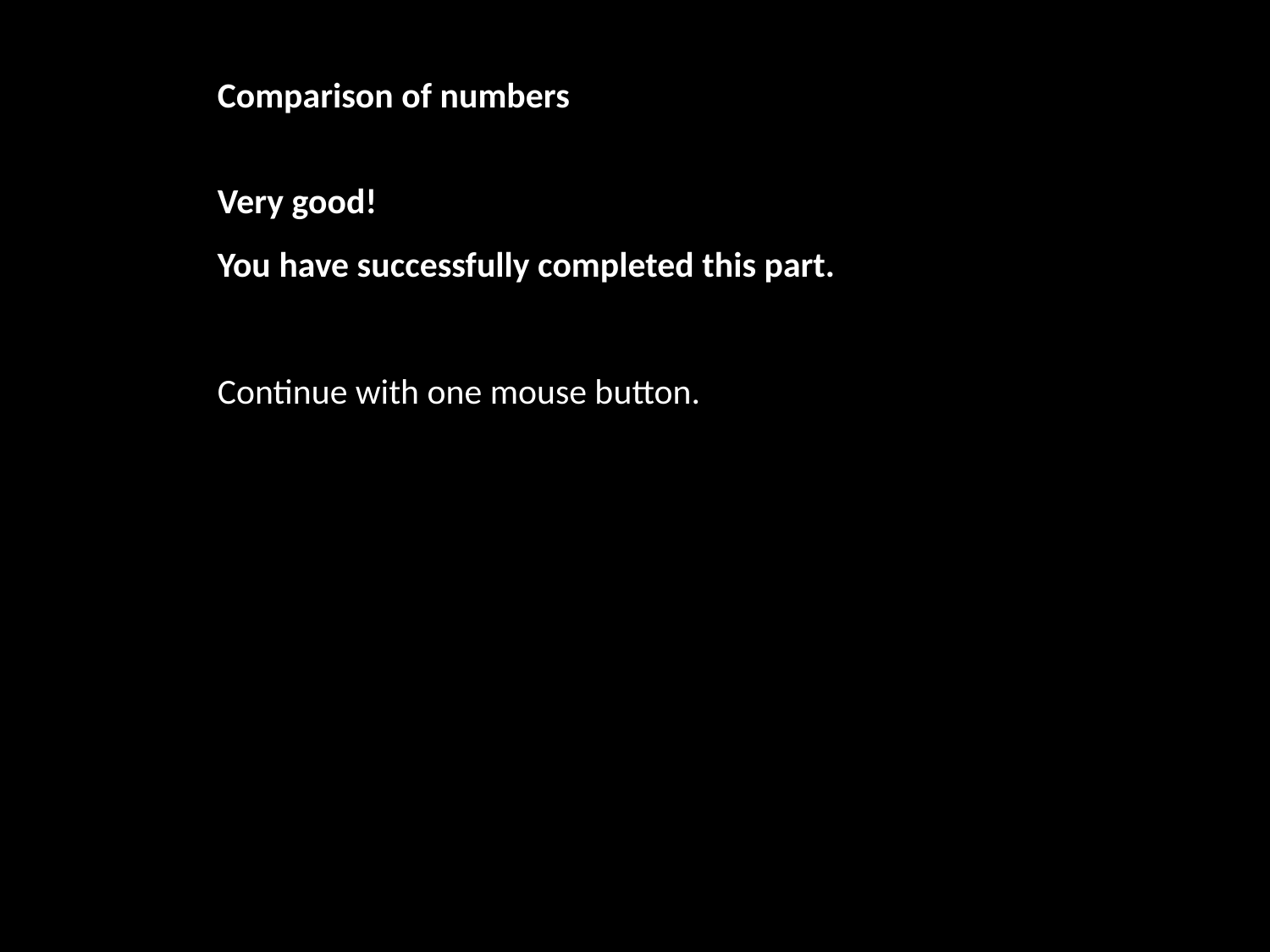

Comparison of numbers
Very good!
You have successfully completed this part.
Continue with one mouse button.

## Slide 11
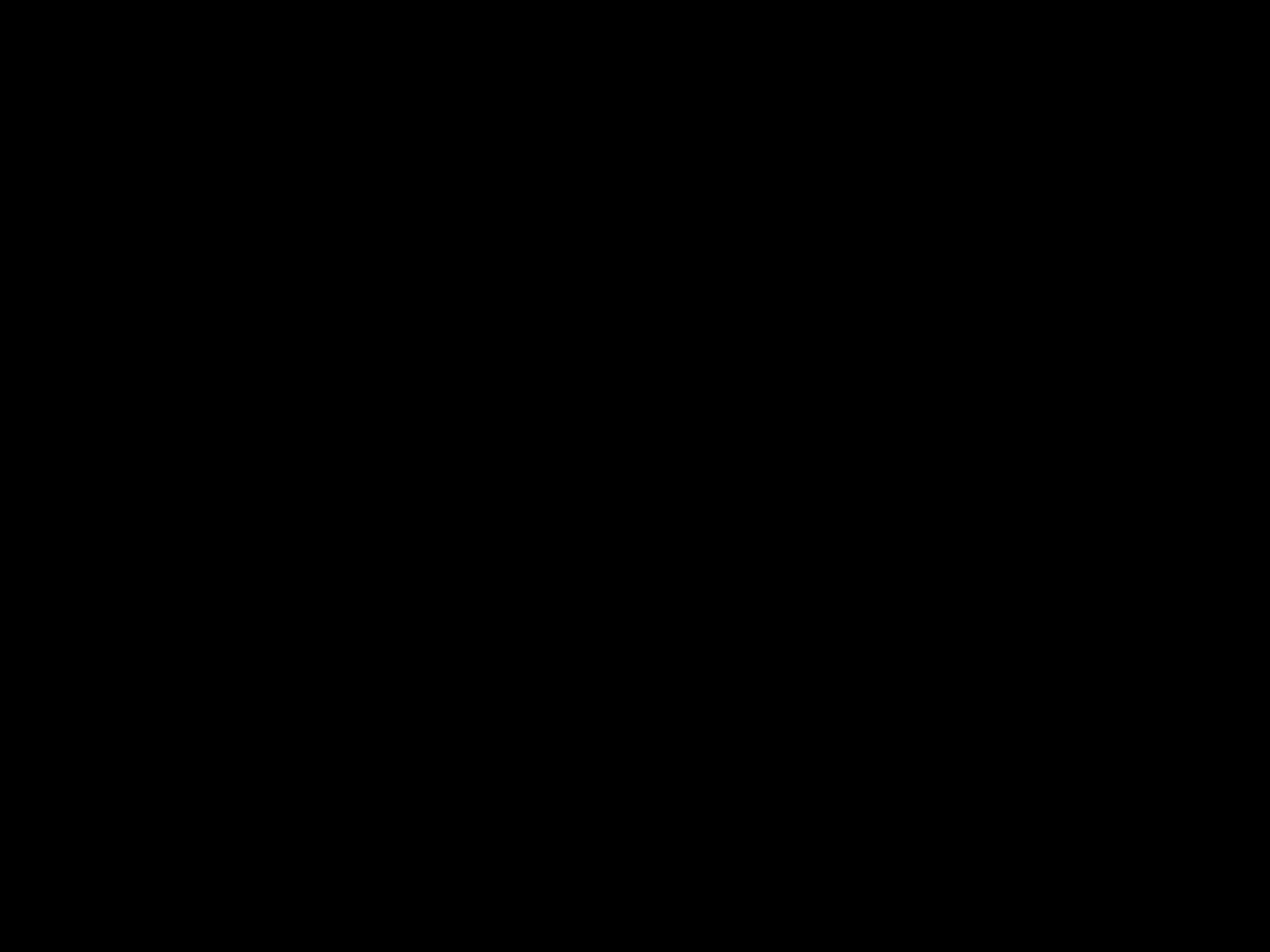

Supplement: S2 File — (ZIP) [file pone.0257717.s002.zip › software/material/InstructionHaveTo.pptx]
