# Supplementary material for: Detours increase local knowledge—Exploring the hidden benefits of self-control failure
Source: PLoS One. 2021 Oct 1;16(10):e0257717. doi: 10.1371/journal.pone.0257717 (PMC8486128; doi:10.1371/journal.pone.0257717)
Supplement: S2 File — (ZIP) [file pone.0257717.s002.zip › software/material/InstructionProcedure.pptx]

## Slide 1
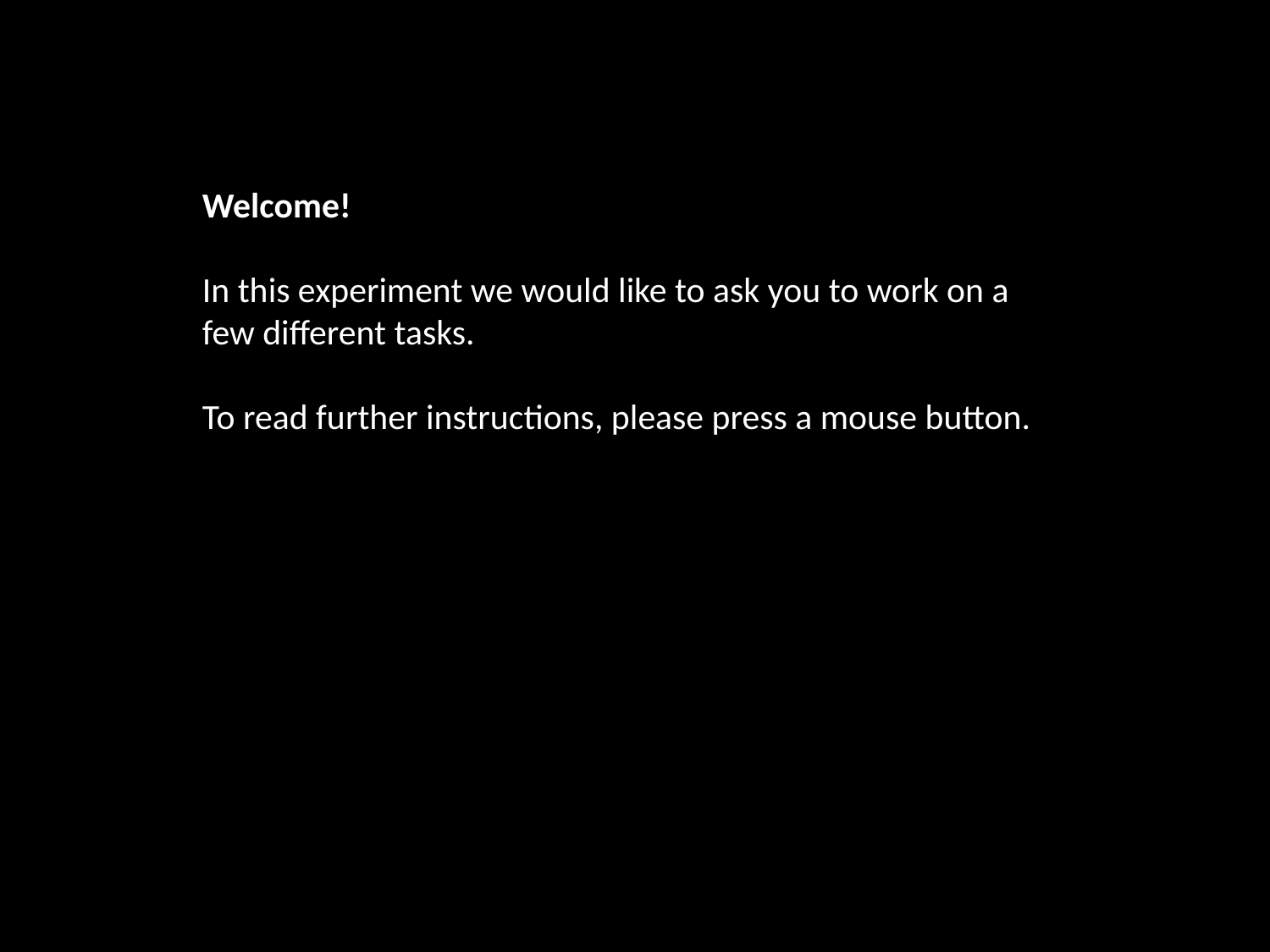

Welcome!
In this experiment we would like to ask you to work on a few different tasks.
To read further instructions, please press a mouse button.

## Slide 2
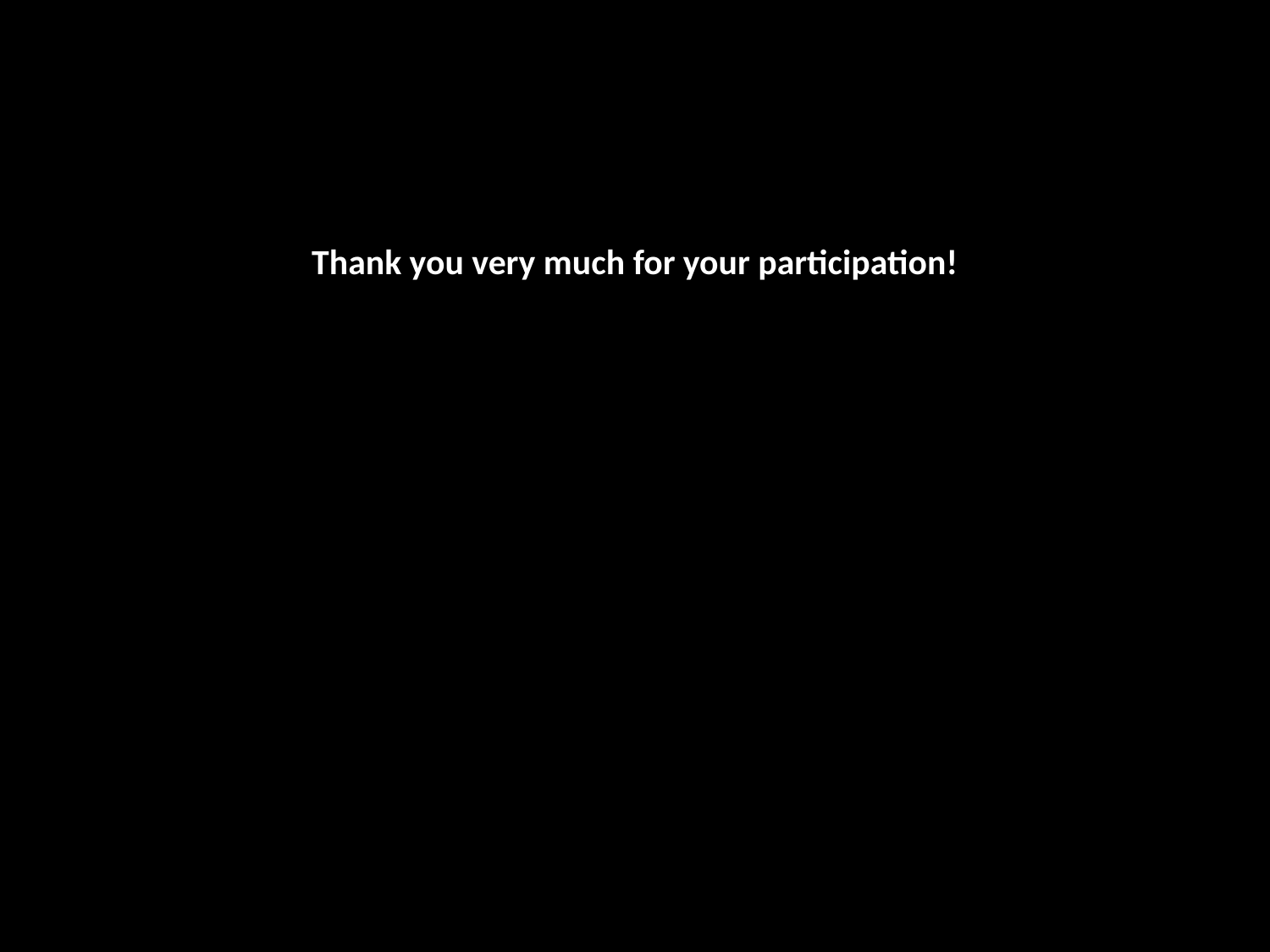

Thank you very much for your participation!
