# Supplementary material for: Detours increase local knowledge—Exploring the hidden benefits of self-control failure
Source: PLoS One. 2021 Oct 1;16(10):e0257717. doi: 10.1371/journal.pone.0257717 (PMC8486128; doi:10.1371/journal.pone.0257717)
Supplement: S2 File — (ZIP) [file pone.0257717.s002.zip › software/material/InstructionVideo.pptx]

## Slide 1
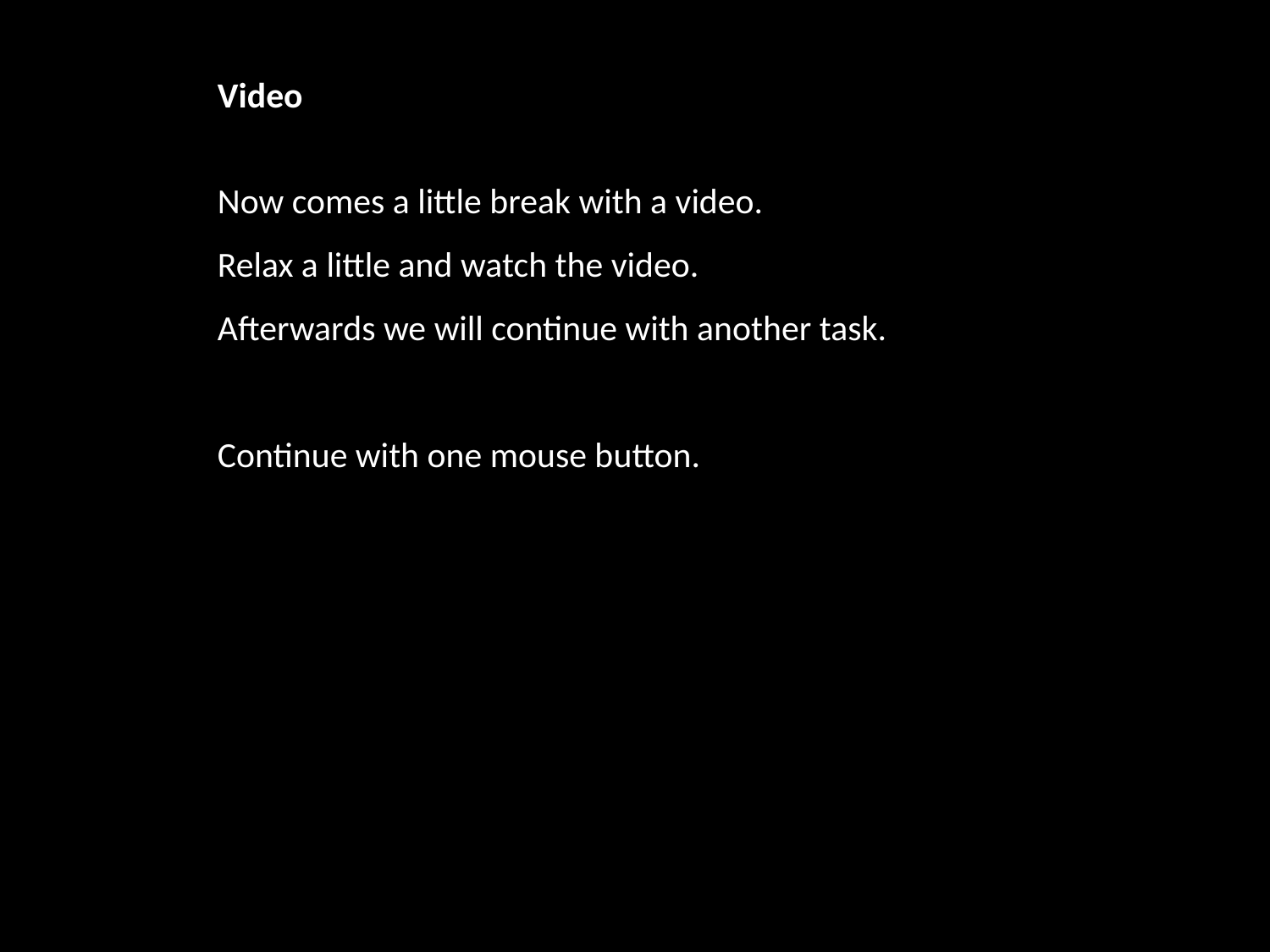

Video
Now comes a little break with a video.
Relax a little and watch the video.
Afterwards we will continue with another task.
Continue with one mouse button.

## Slide 2
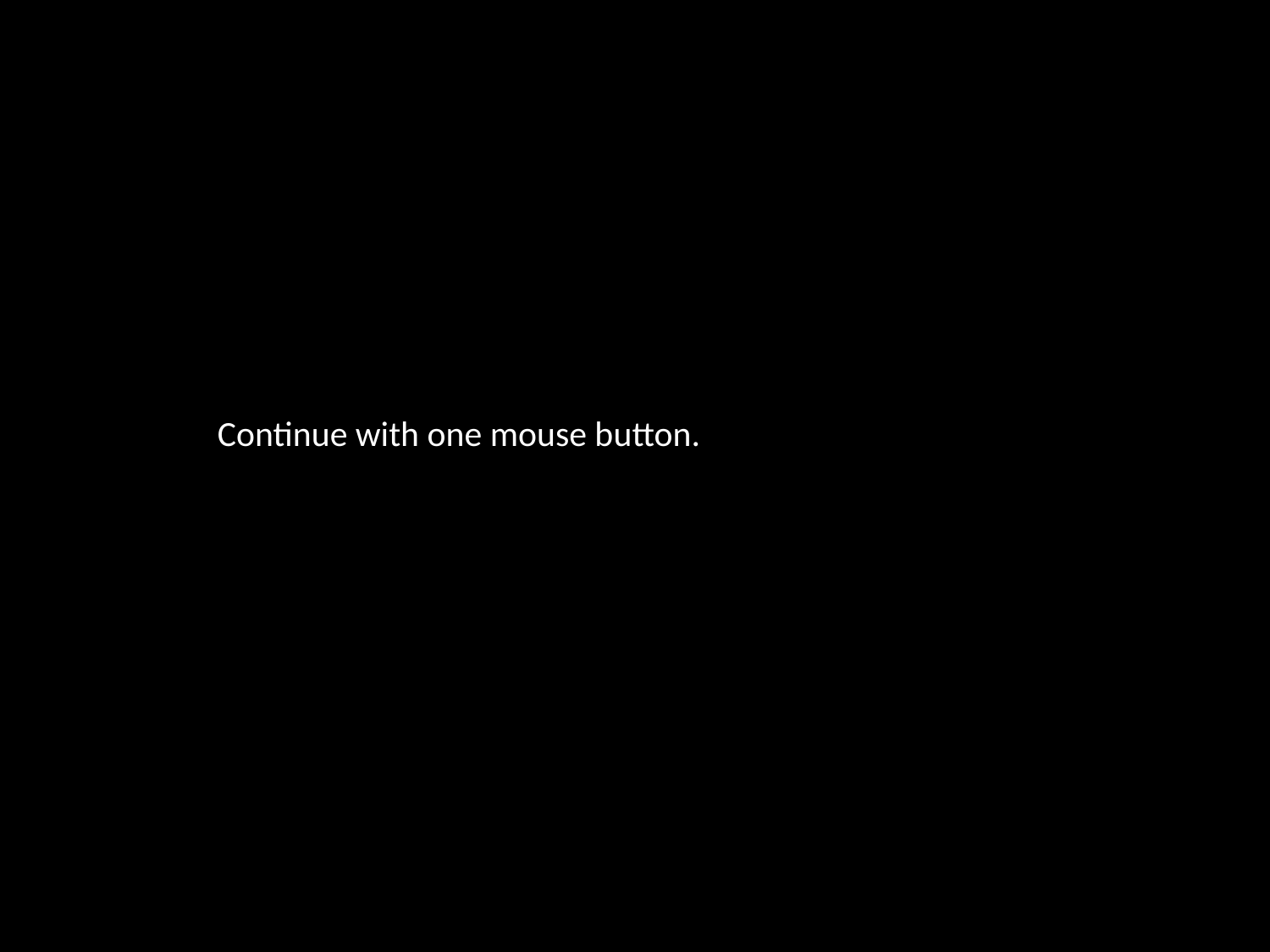

Continue with one mouse button.
