# Supplementary material for: Detours increase local knowledge—Exploring the hidden benefits of self-control failure
Source: PLoS One. 2021 Oct 1;16(10):e0257717. doi: 10.1371/journal.pone.0257717 (PMC8486128; doi:10.1371/journal.pone.0257717)

## Slide 1
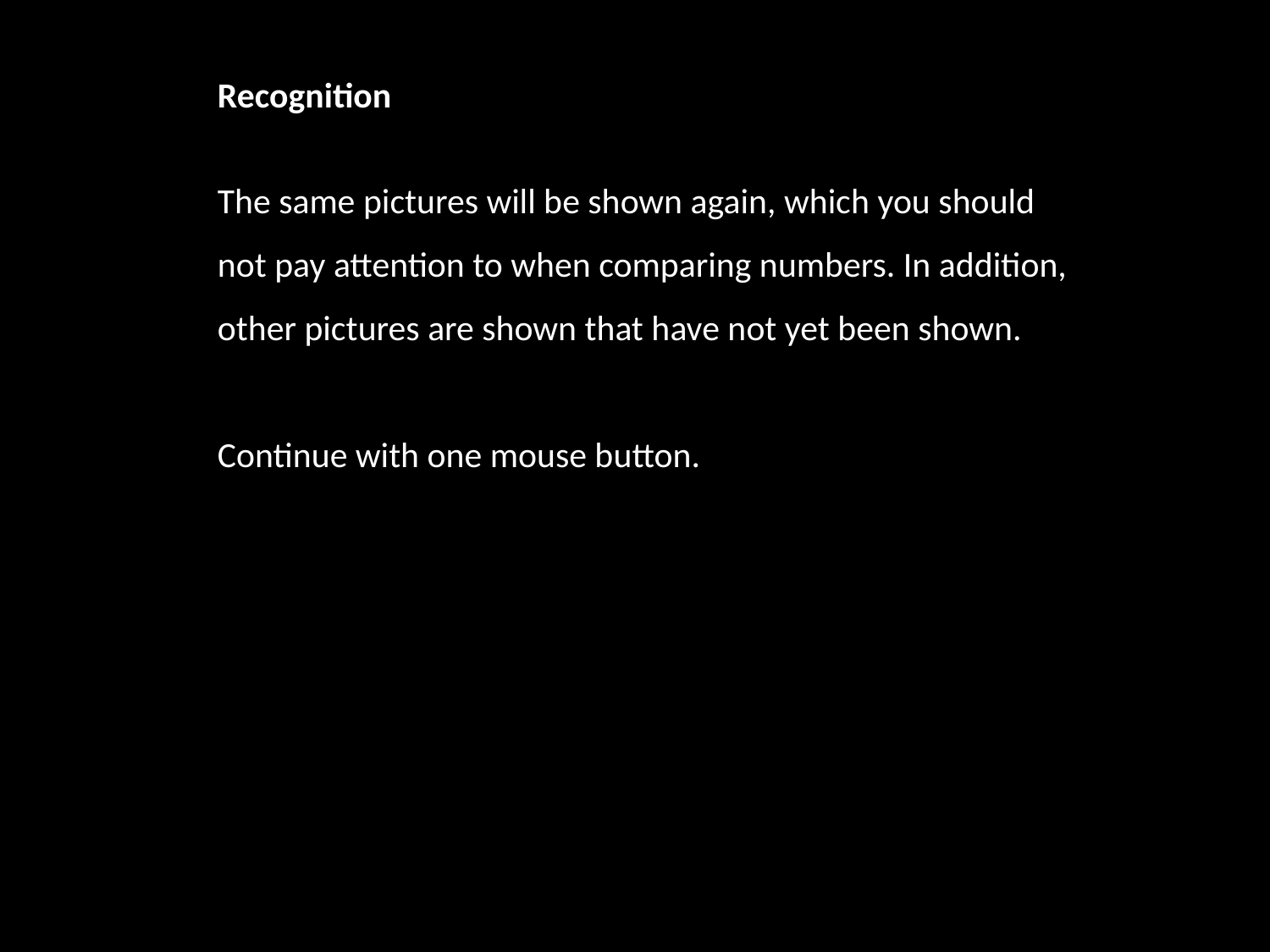

Recognition
The same pictures will be shown again, which you should not pay attention to when comparing numbers. In addition, other pictures are shown that have not yet been shown.
Continue with one mouse button.

## Slide 2
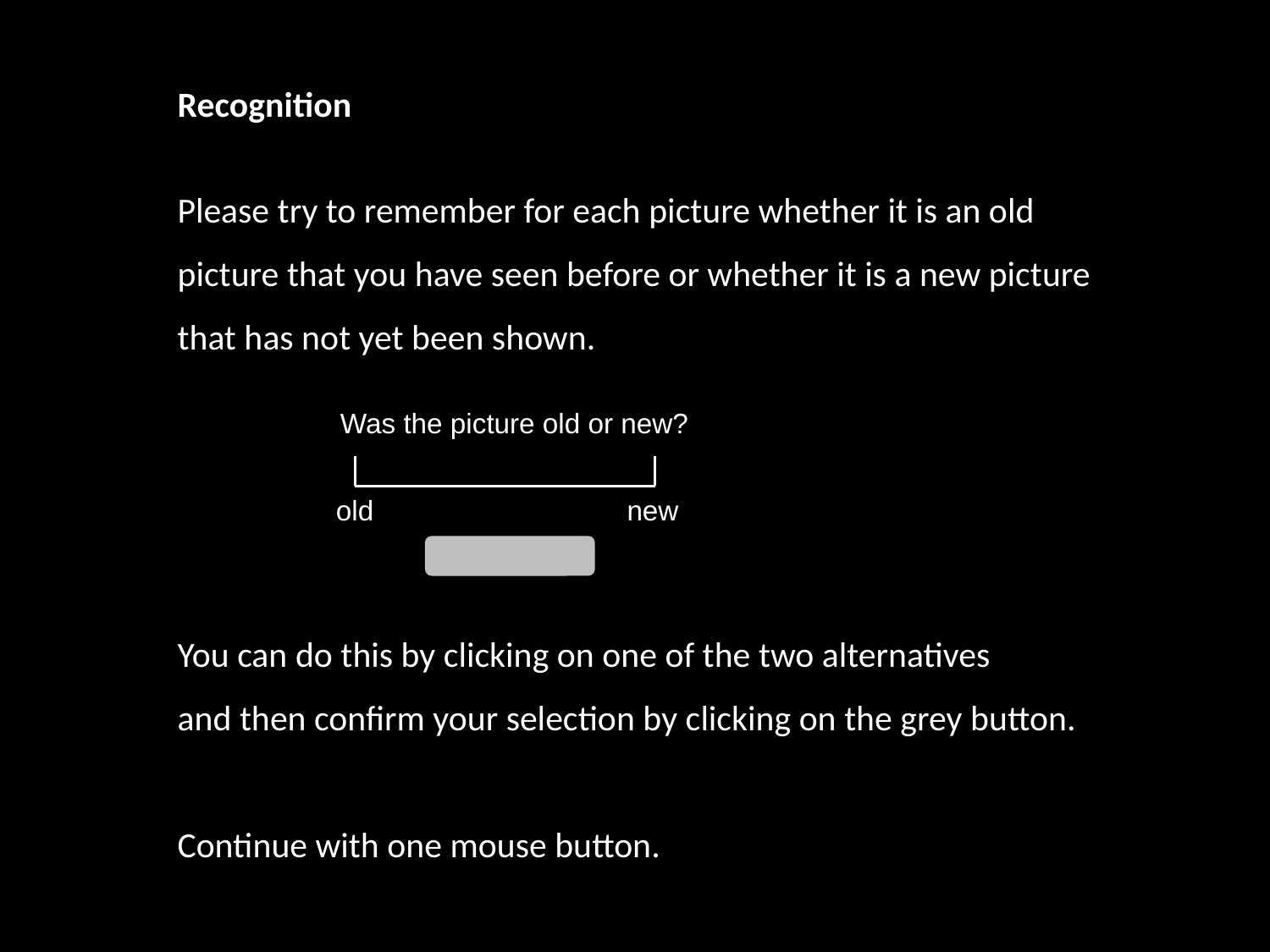

Recognition
Please try to remember for each picture whether it is an old picture that you have seen before or whether it is a new picture that has not yet been shown.
You can do this by clicking on one of the two alternatives
and then confirm your selection by clicking on the grey button.
Continue with one mouse button.
Was the picture old or new?
old
new

## Slide 3
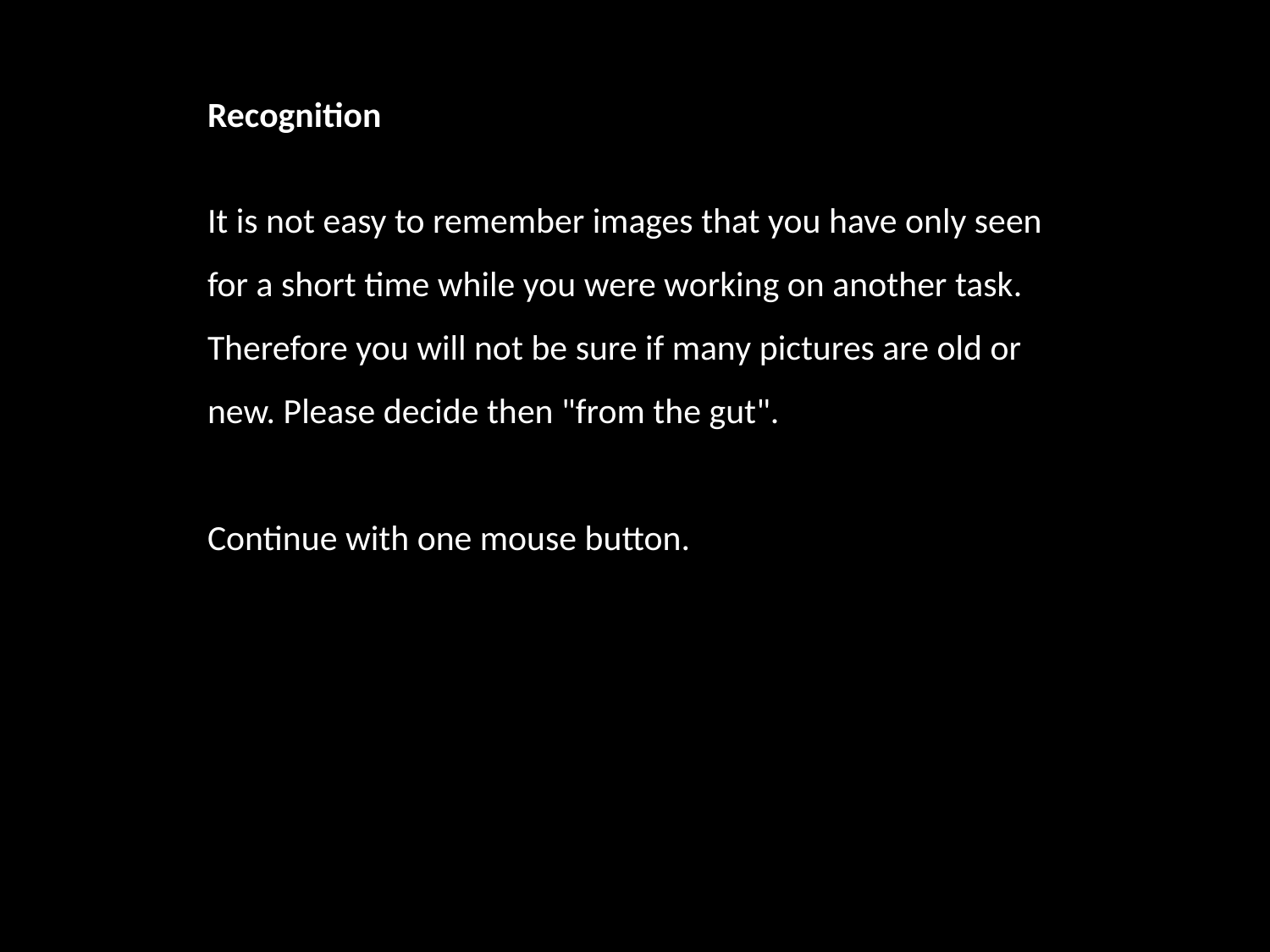

Recognition
It is not easy to remember images that you have only seen for a short time while you were working on another task. Therefore you will not be sure if many pictures are old or new. Please decide then "from the gut".
Continue with one mouse button.

## Slide 4
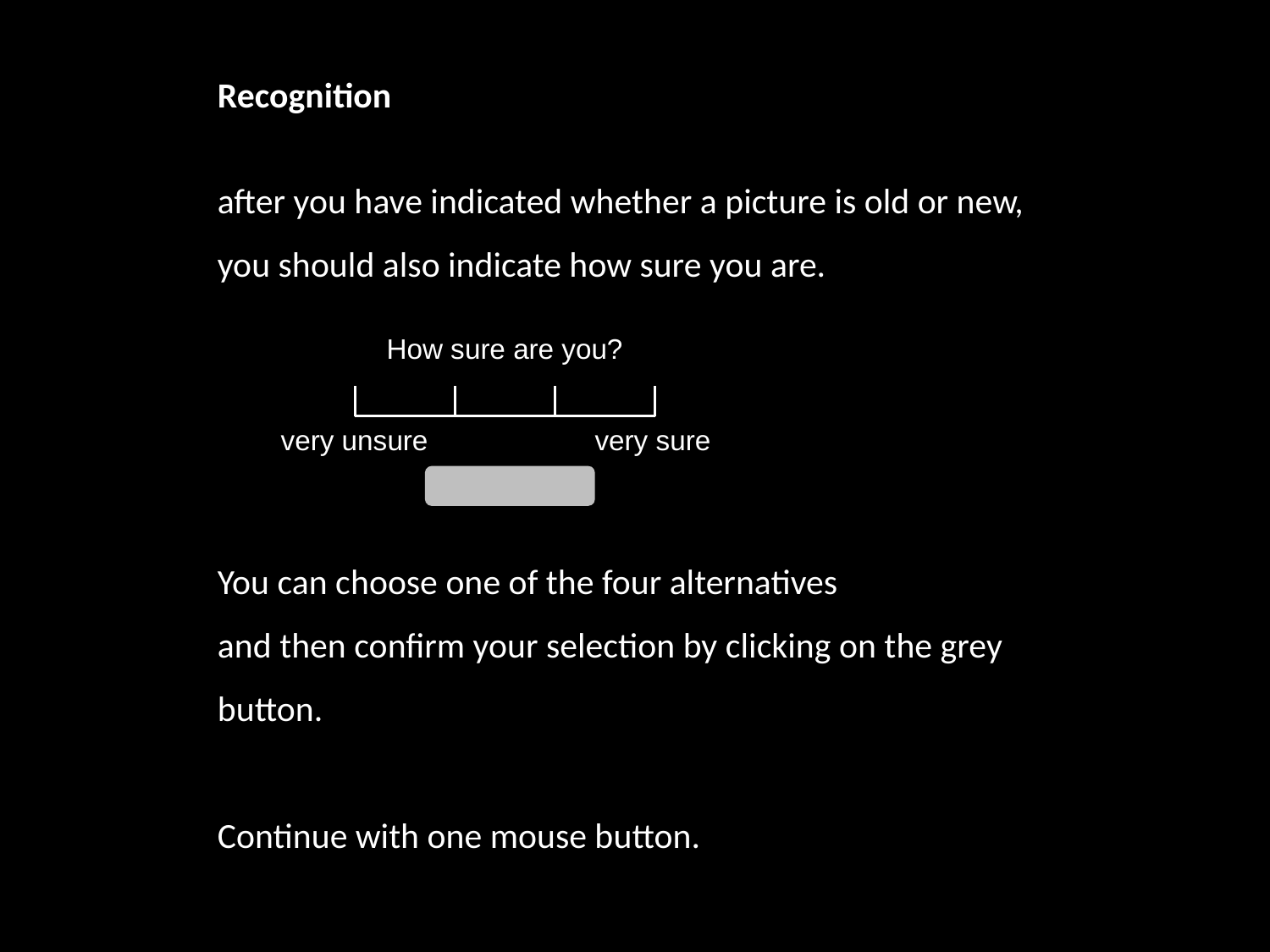

Recognition
after you have indicated whether a picture is old or new, you should also indicate how sure you are.
You can choose one of the four alternatives
and then confirm your selection by clicking on the grey button.
Continue with one mouse button.
How sure are you?
very unsure
very sure

## Slide 5
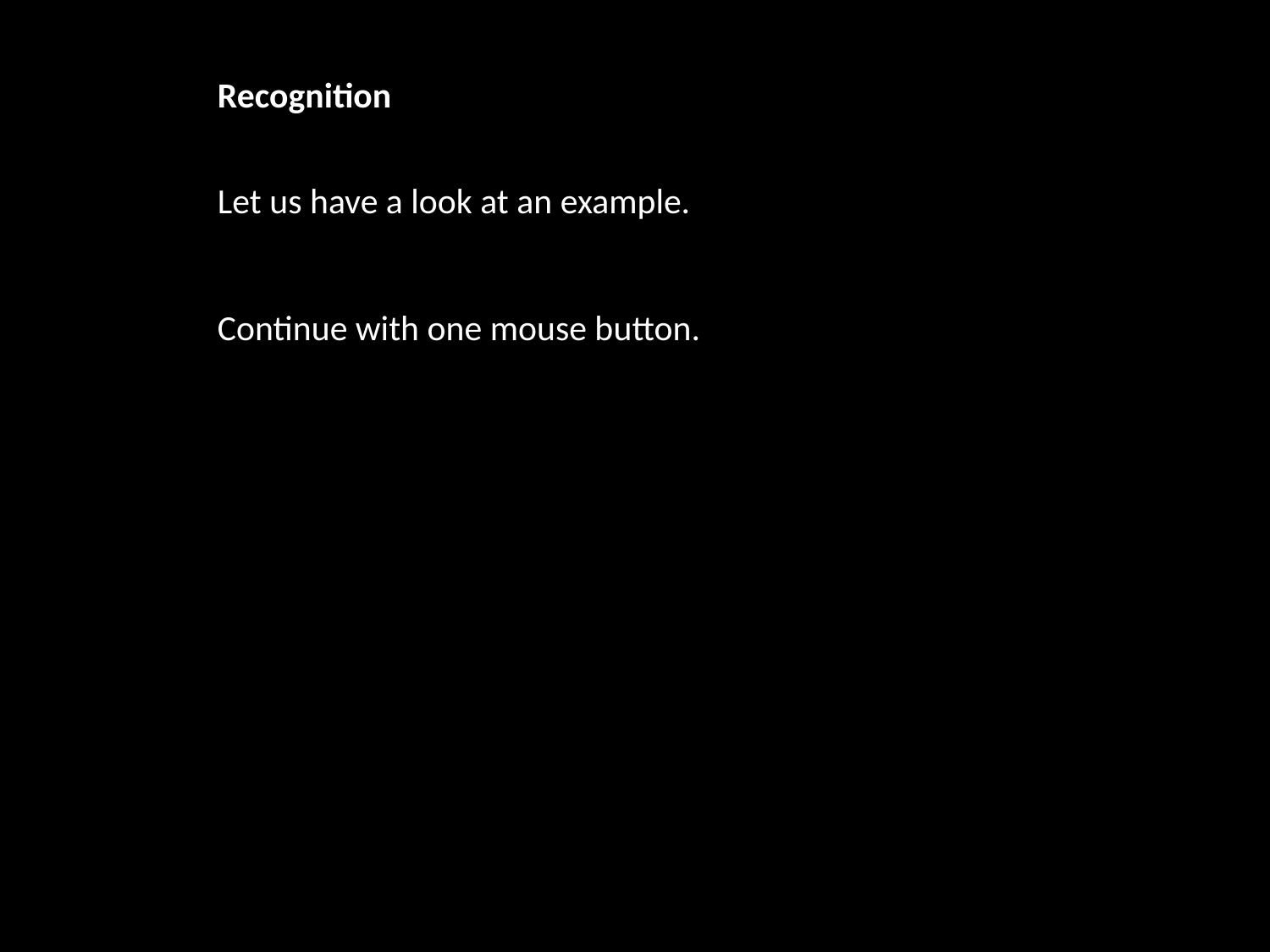

Recognition
Let us have a look at an example.
Continue with one mouse button.

## Slide 6
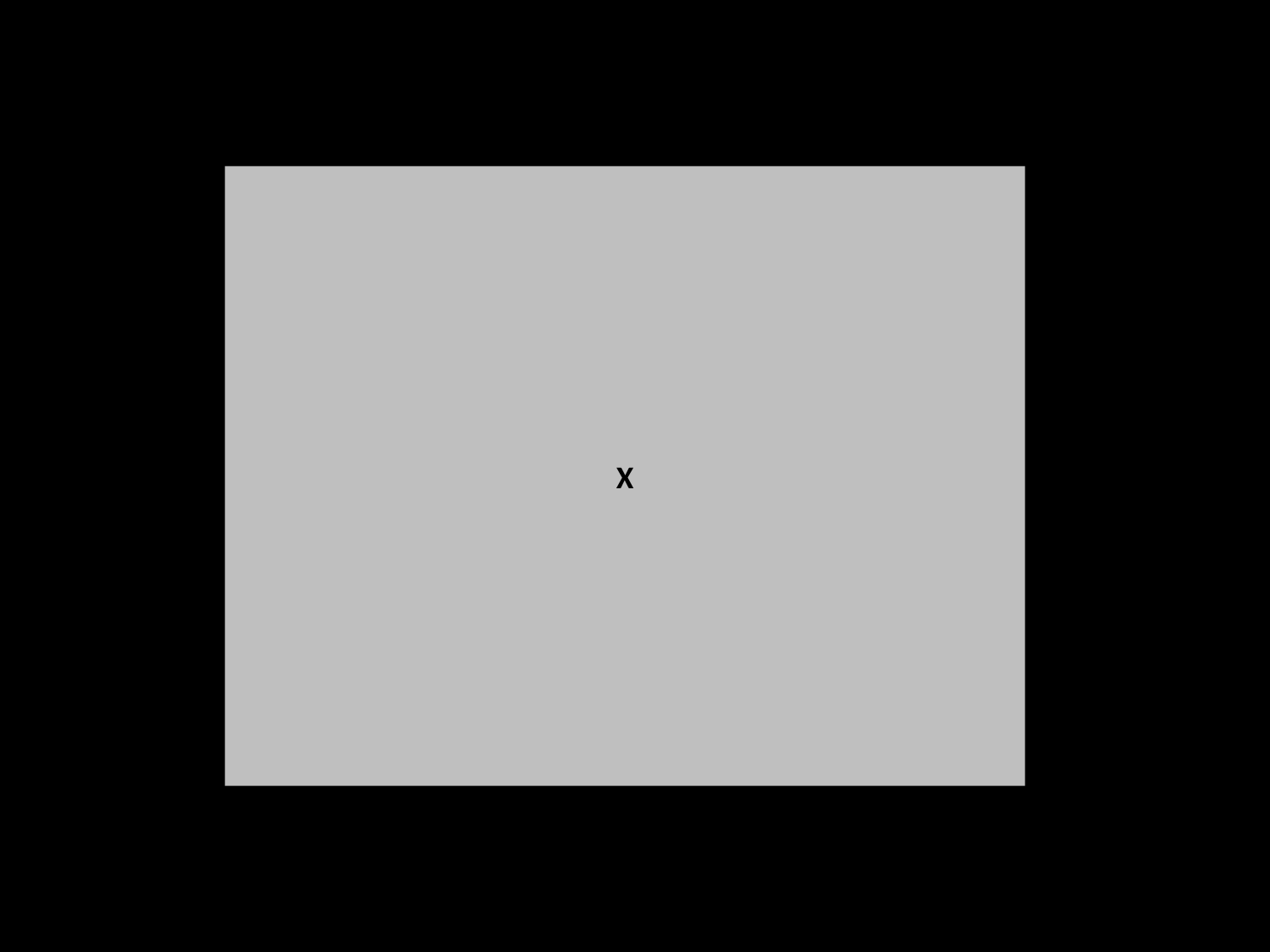

X

## Slide 7
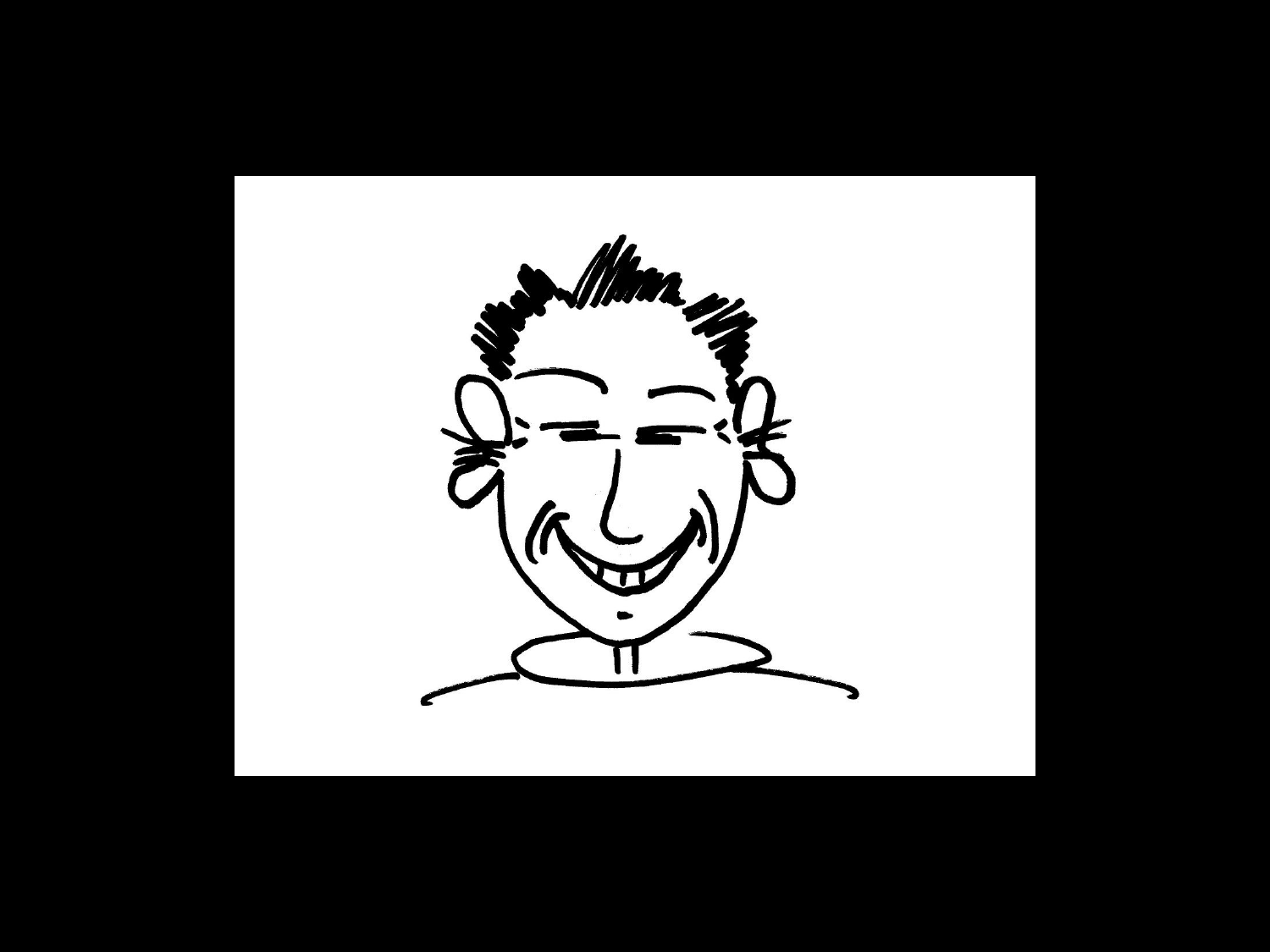

## Slide 8
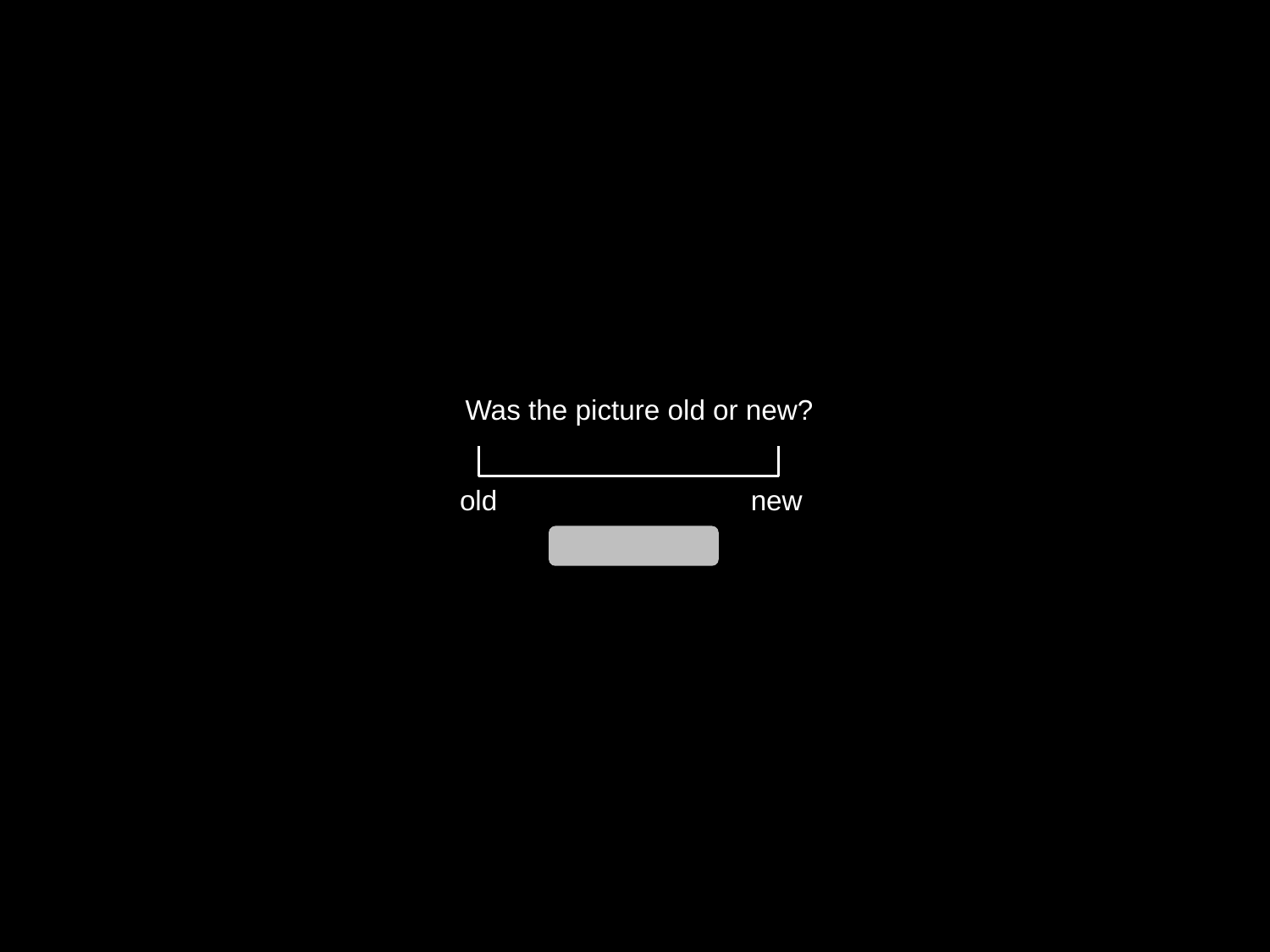

Was the picture old or new?
old
new

## Slide 9
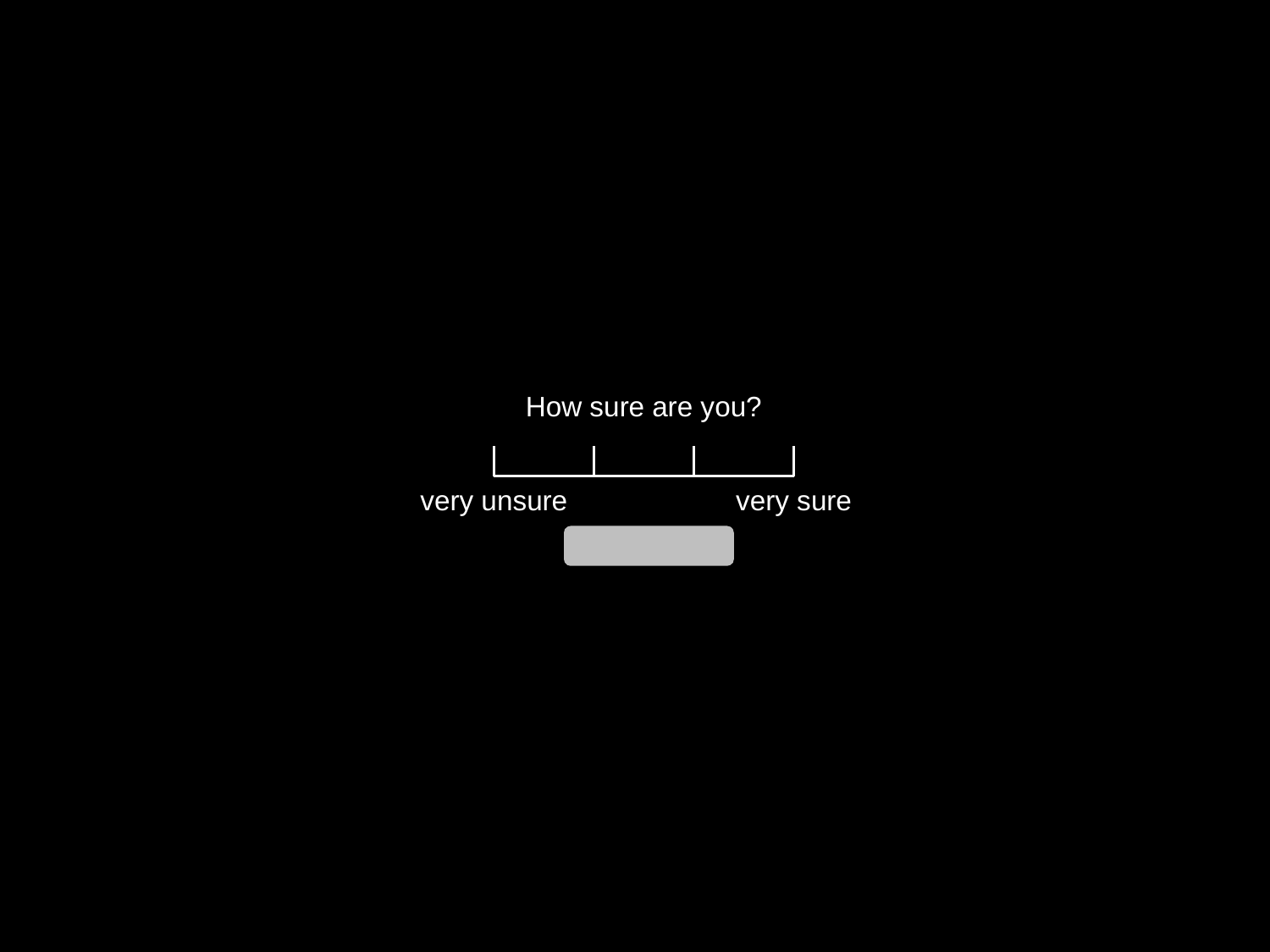

How sure are you?
very unsure
very sure

## Slide 10
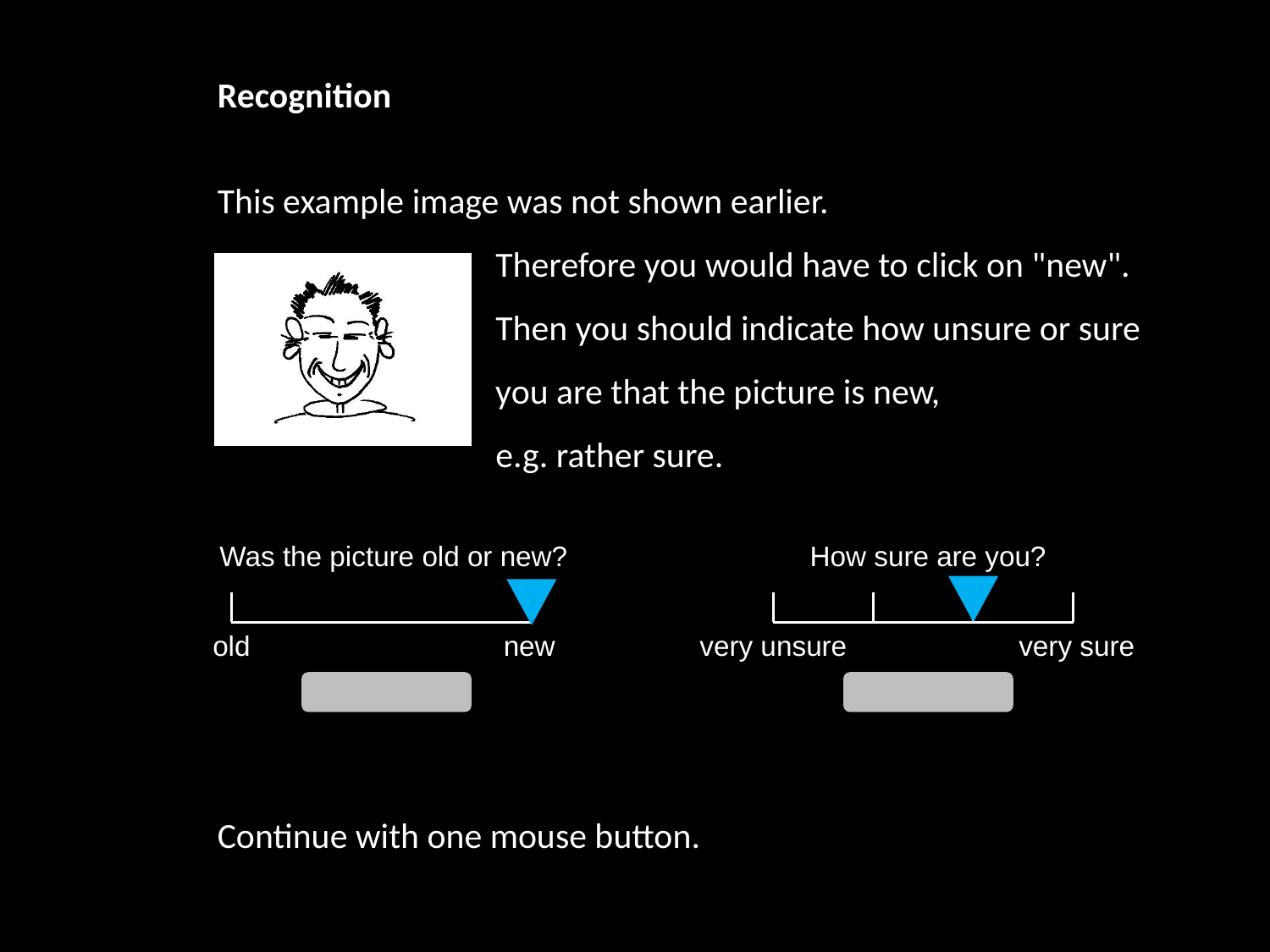

Recognition
This example image was not shown earlier.		 			 Therefore you would have to click on "new".
		 Then you should indicate how unsure or sure 		 you are that the picture is new,
		 e.g. rather sure.
Continue with one mouse button.
Was the picture old or new?
old
new
How sure are you?
very sure
very unsure

## Slide 11
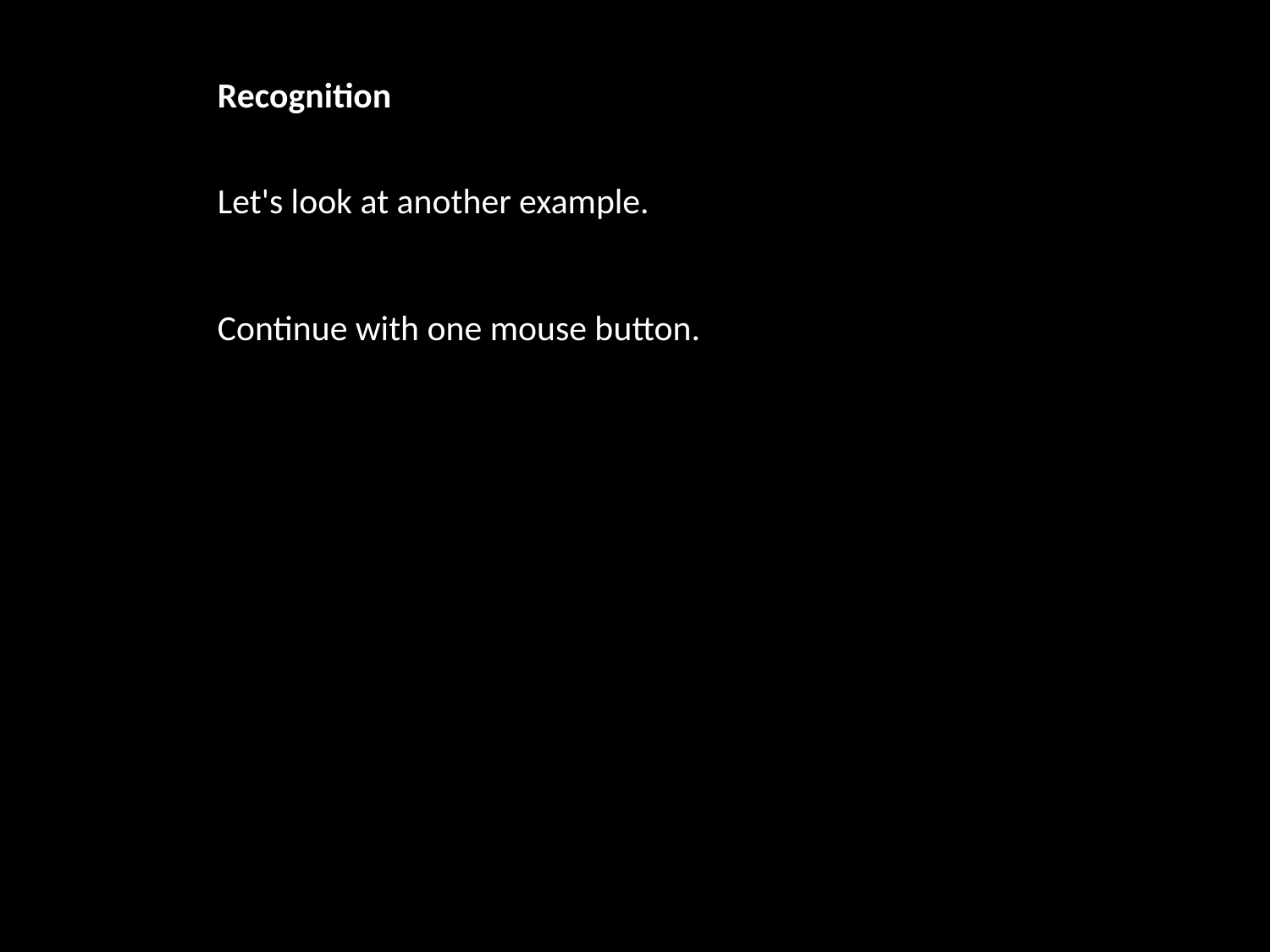

Recognition
Let's look at another example.
Continue with one mouse button.

## Slide 12
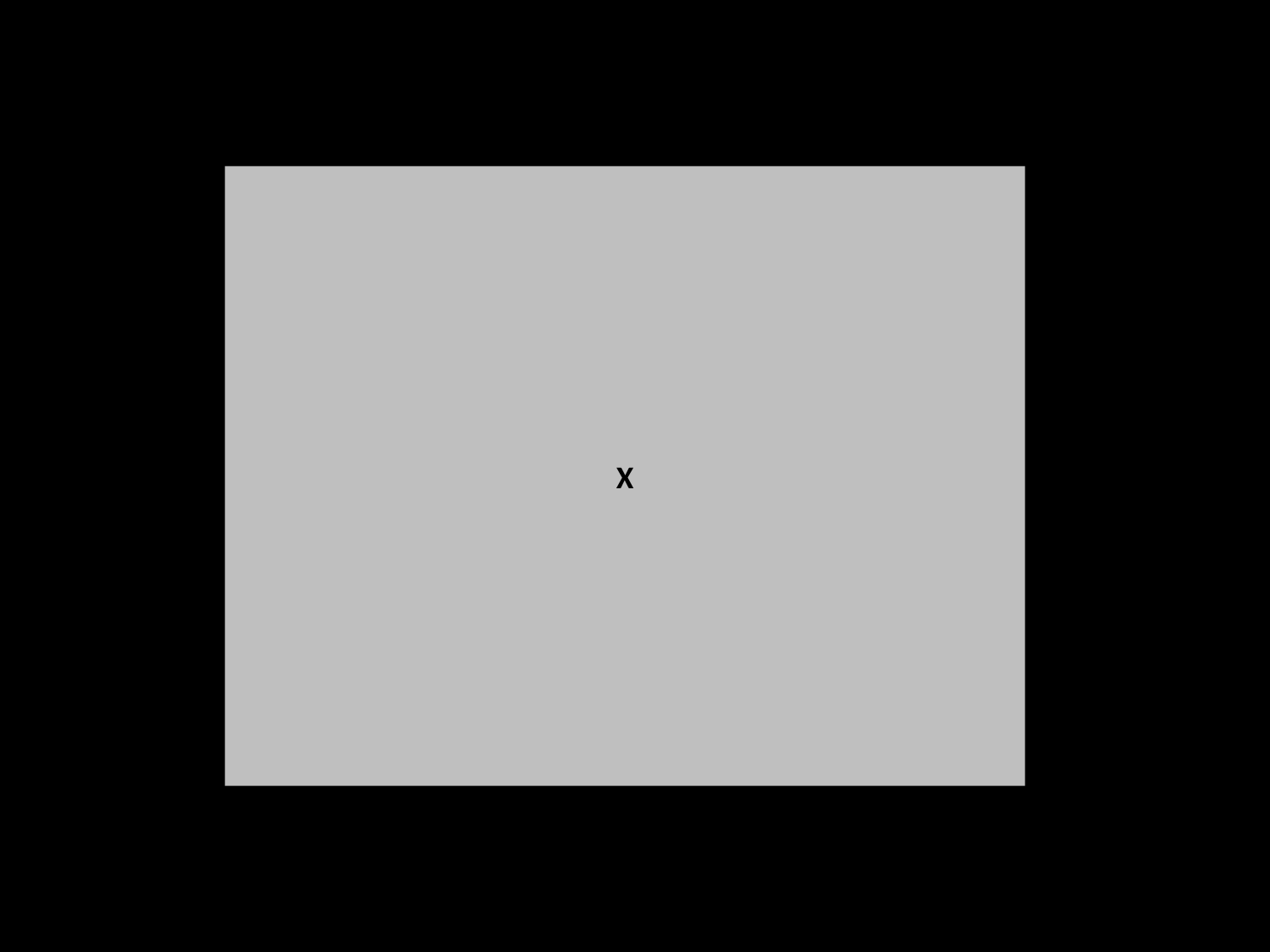

X

## Slide 13
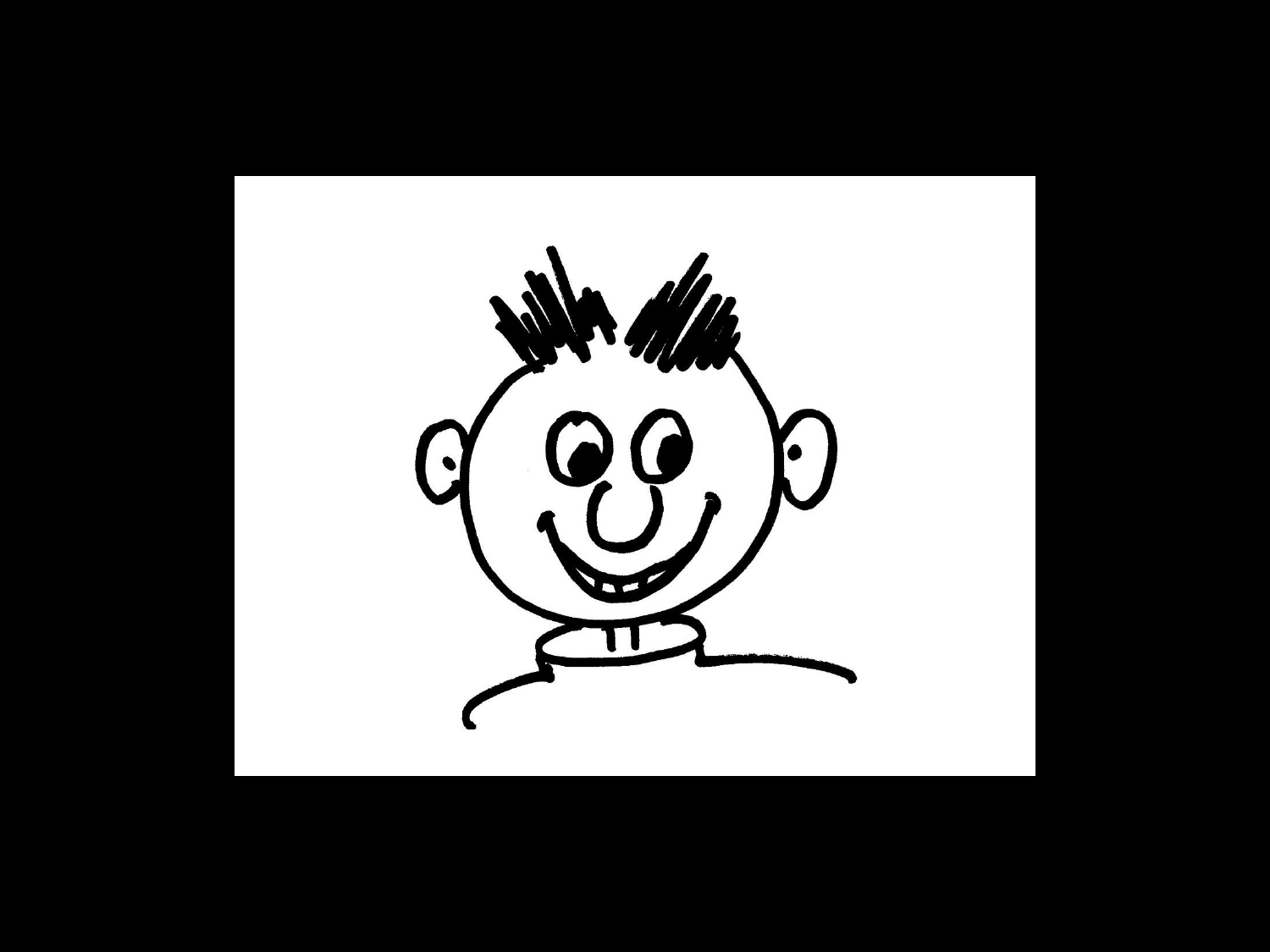

## Slide 14
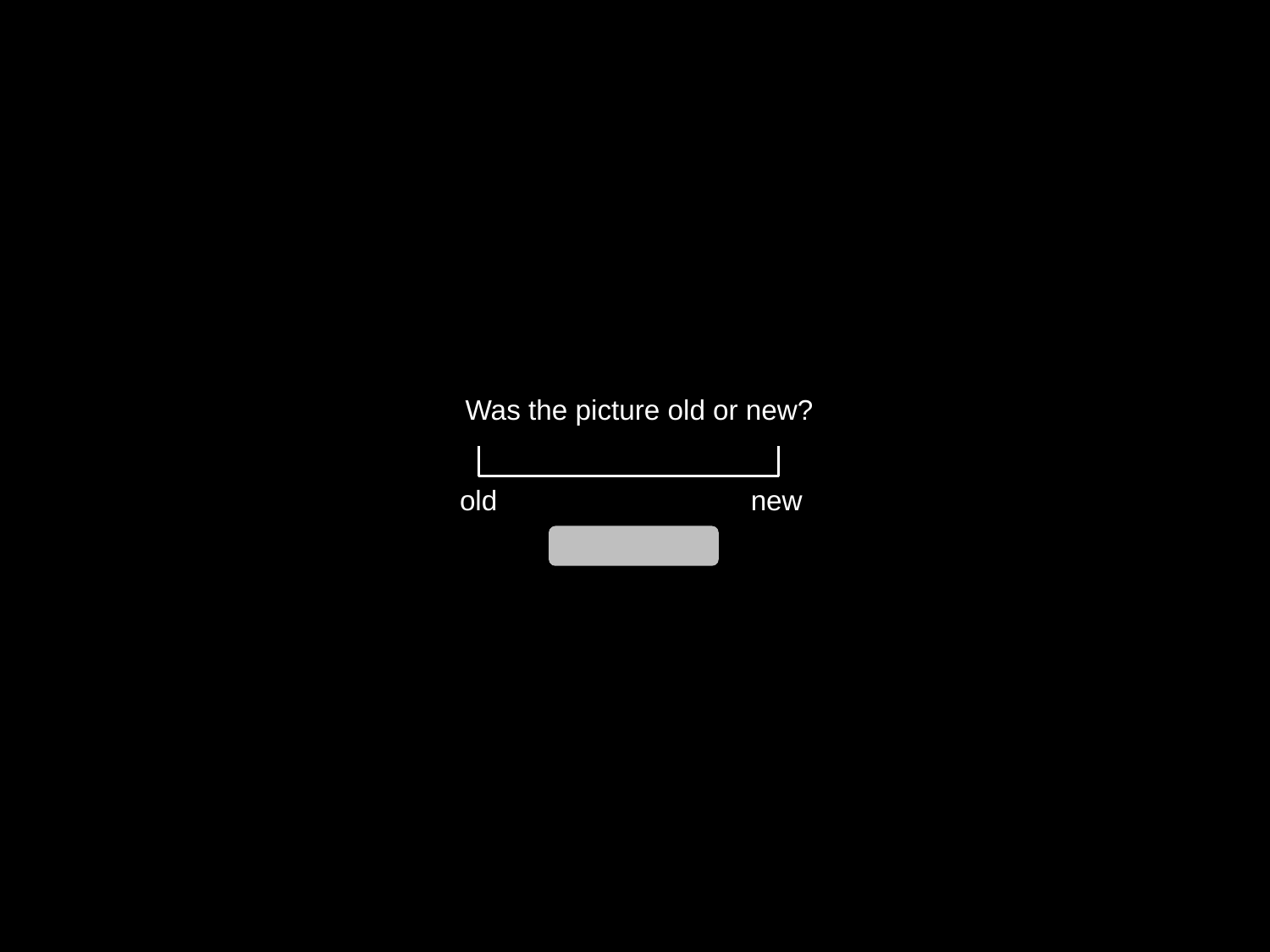

Was the picture old or new?
old
new

## Slide 15
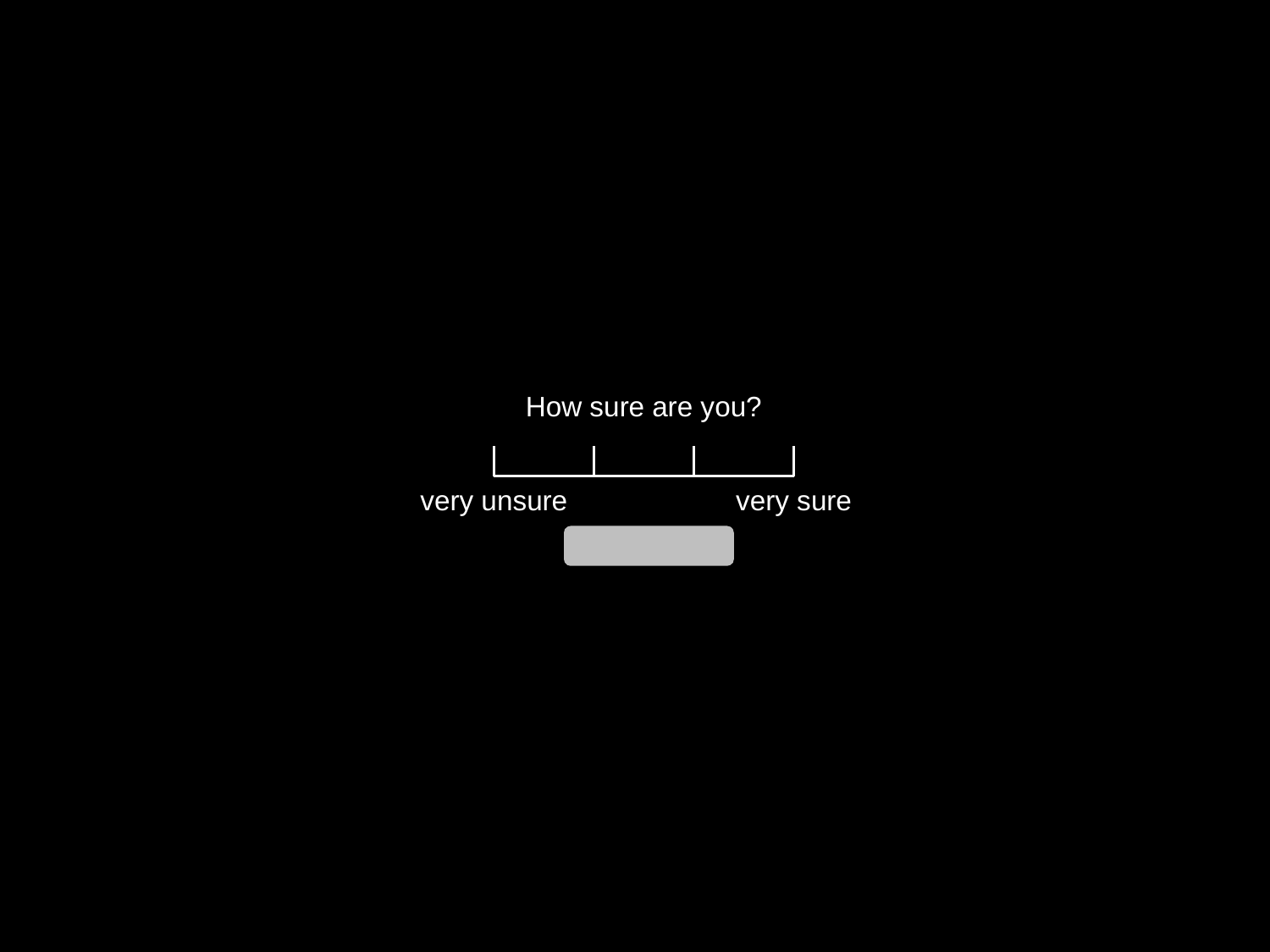

How sure are you?
very unsure
very sure

## Slide 16
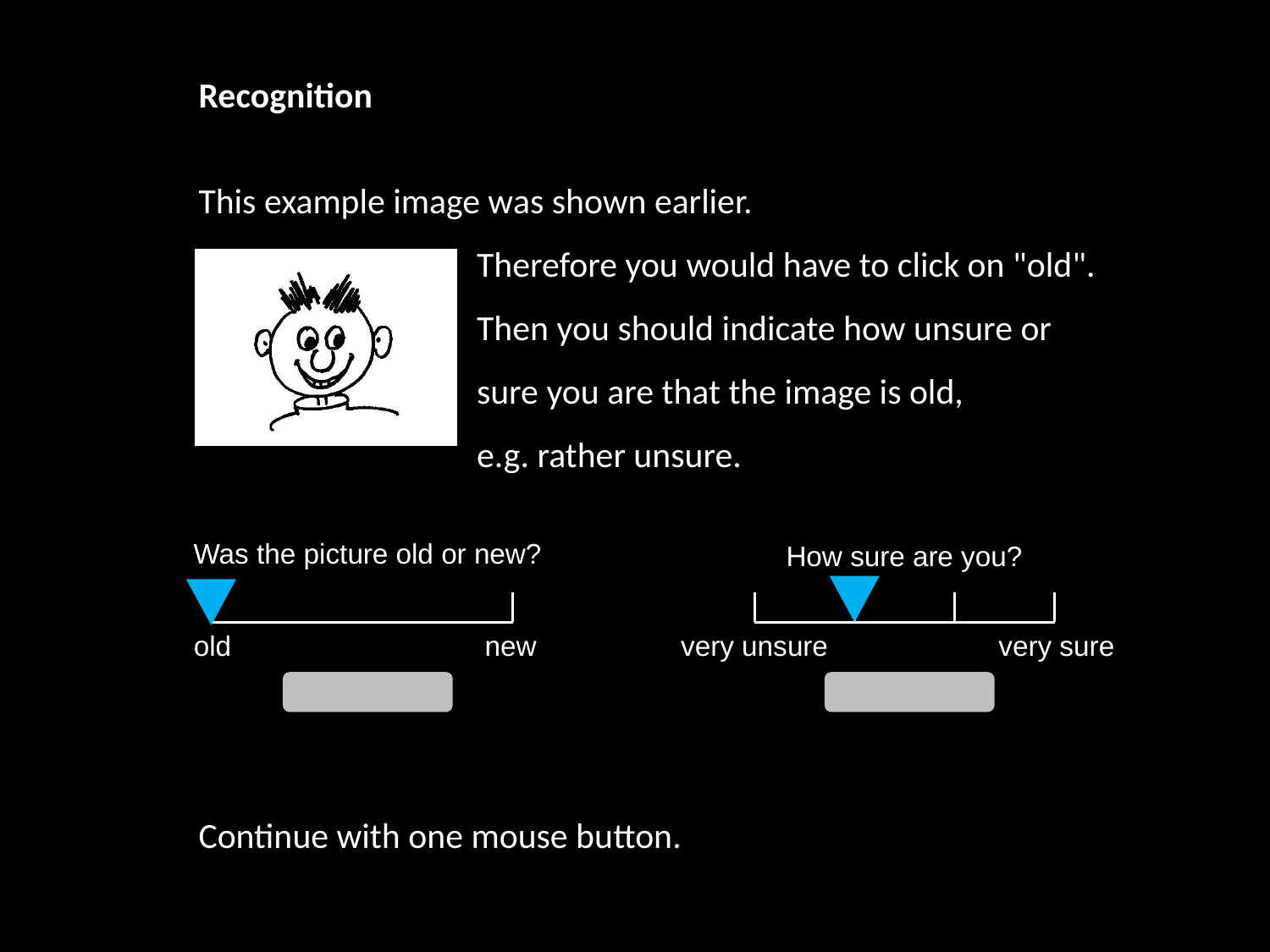

Recognition
This example image was shown earlier.
		 Therefore you would have to click on "old".
		 Then you should indicate how unsure or 			 sure you are that the image is old,
		 e.g. rather unsure.
Continue with one mouse button.
Was the picture old or new?
old
new
How sure are you?
very sure
very unsure

## Slide 17
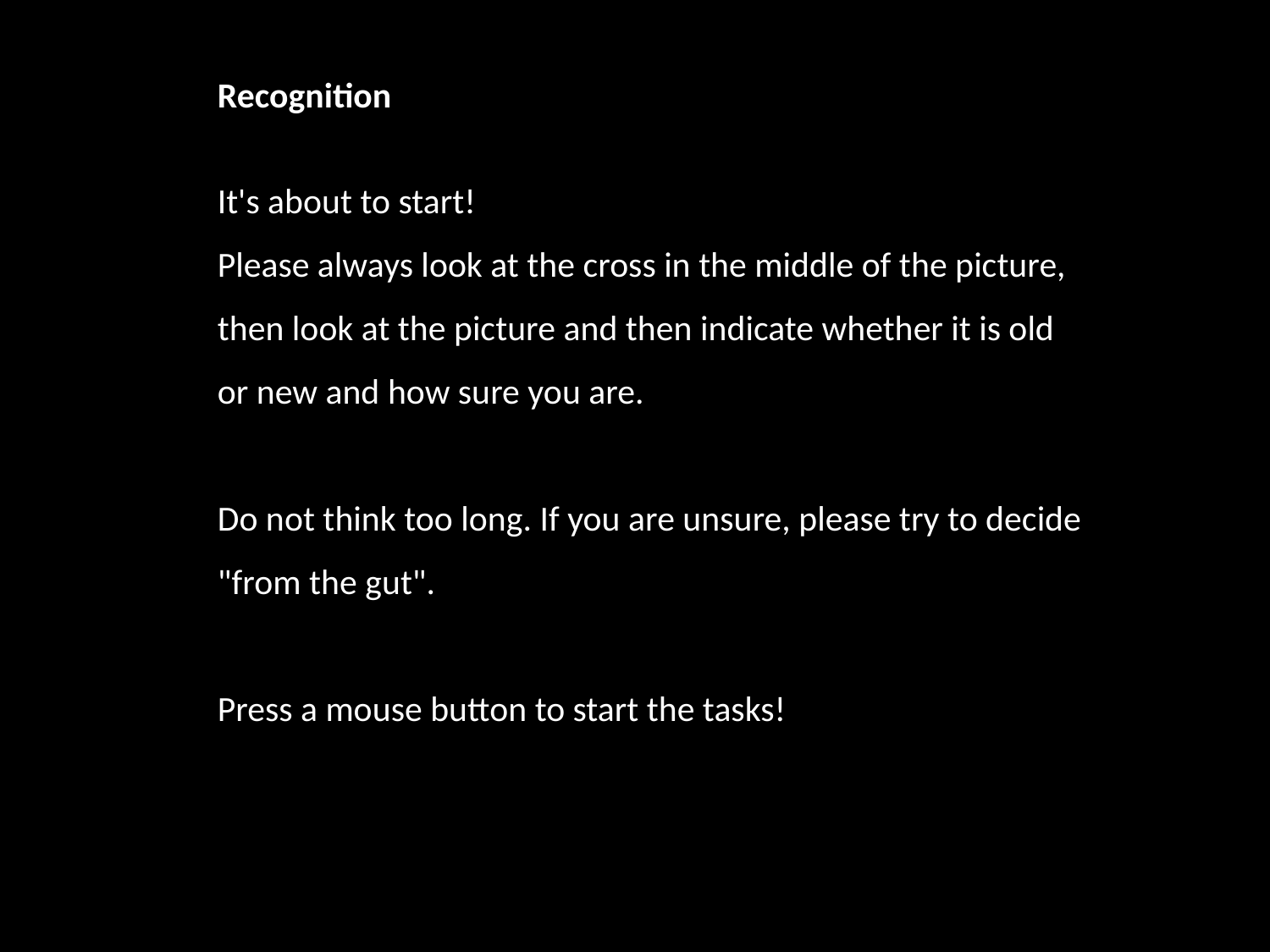

Recognition
It's about to start!
Please always look at the cross in the middle of the picture,
then look at the picture and then indicate whether it is old or new and how sure you are.
Do not think too long. If you are unsure, please try to decide "from the gut".
Press a mouse button to start the tasks!

## Slide 18
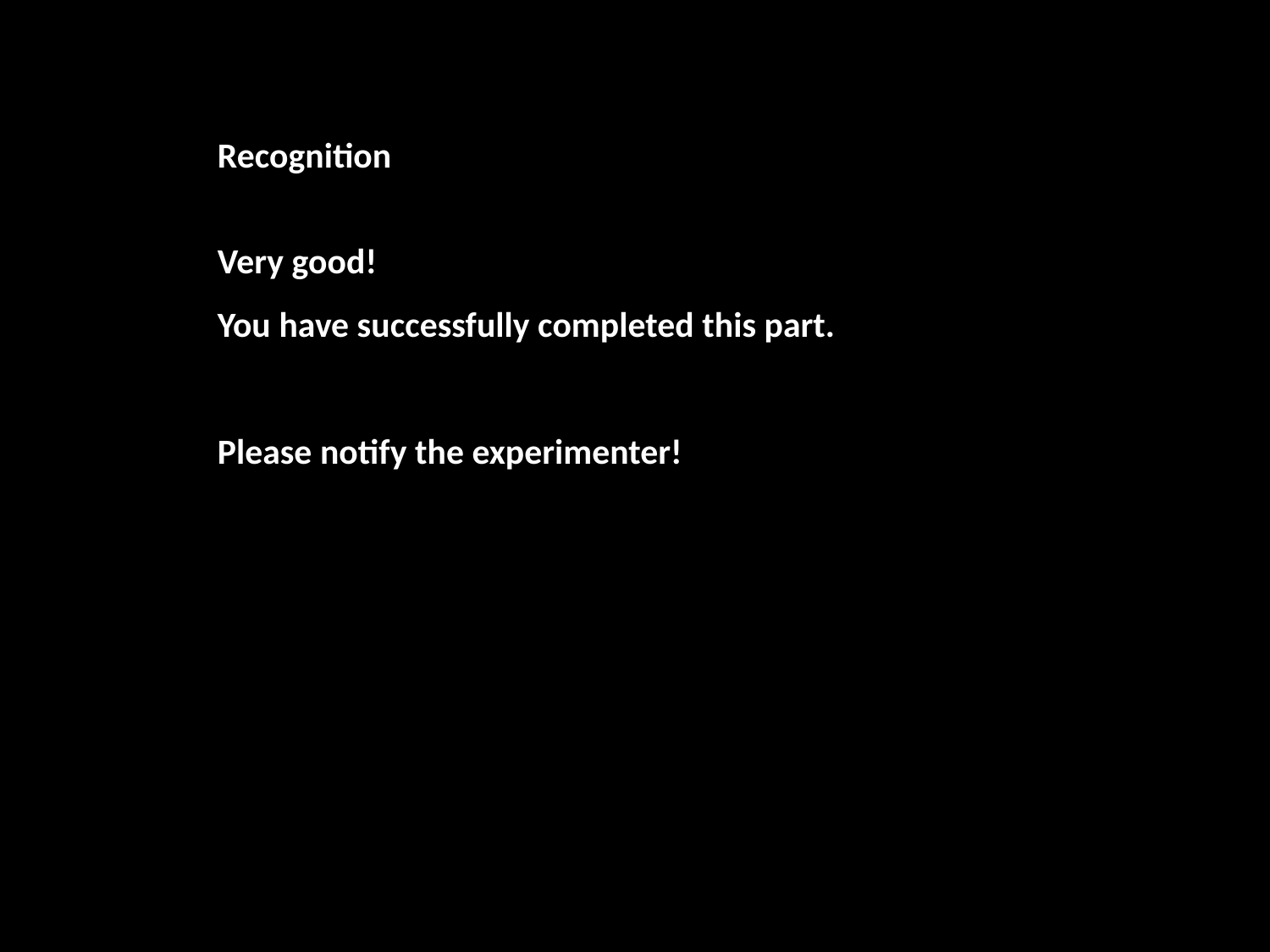

Recognition
Very good!
You have successfully completed this part.
Please notify the experimenter!

## Slide 19
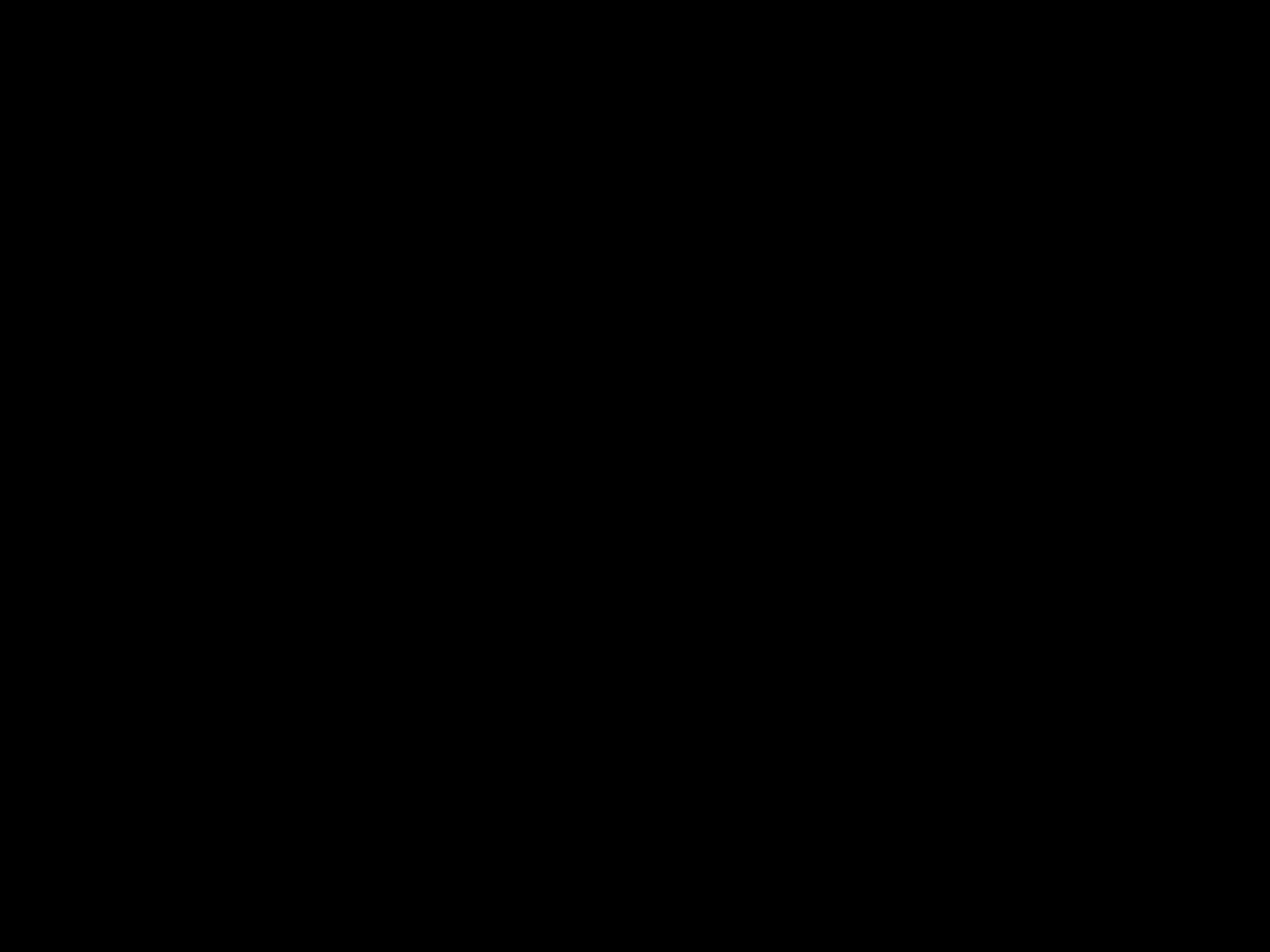

Supplement: S2 File — (ZIP) [file pone.0257717.s002.zip › software/material/InstructionWantTo.pptx]
