# Supplementary material for: Detours increase local knowledge—Exploring the hidden benefits of self-control failure
Source: PLoS One. 2021 Oct 1;16(10):e0257717. doi: 10.1371/journal.pone.0257717 (PMC8486128; doi:10.1371/journal.pone.0257717)

## Slide 1
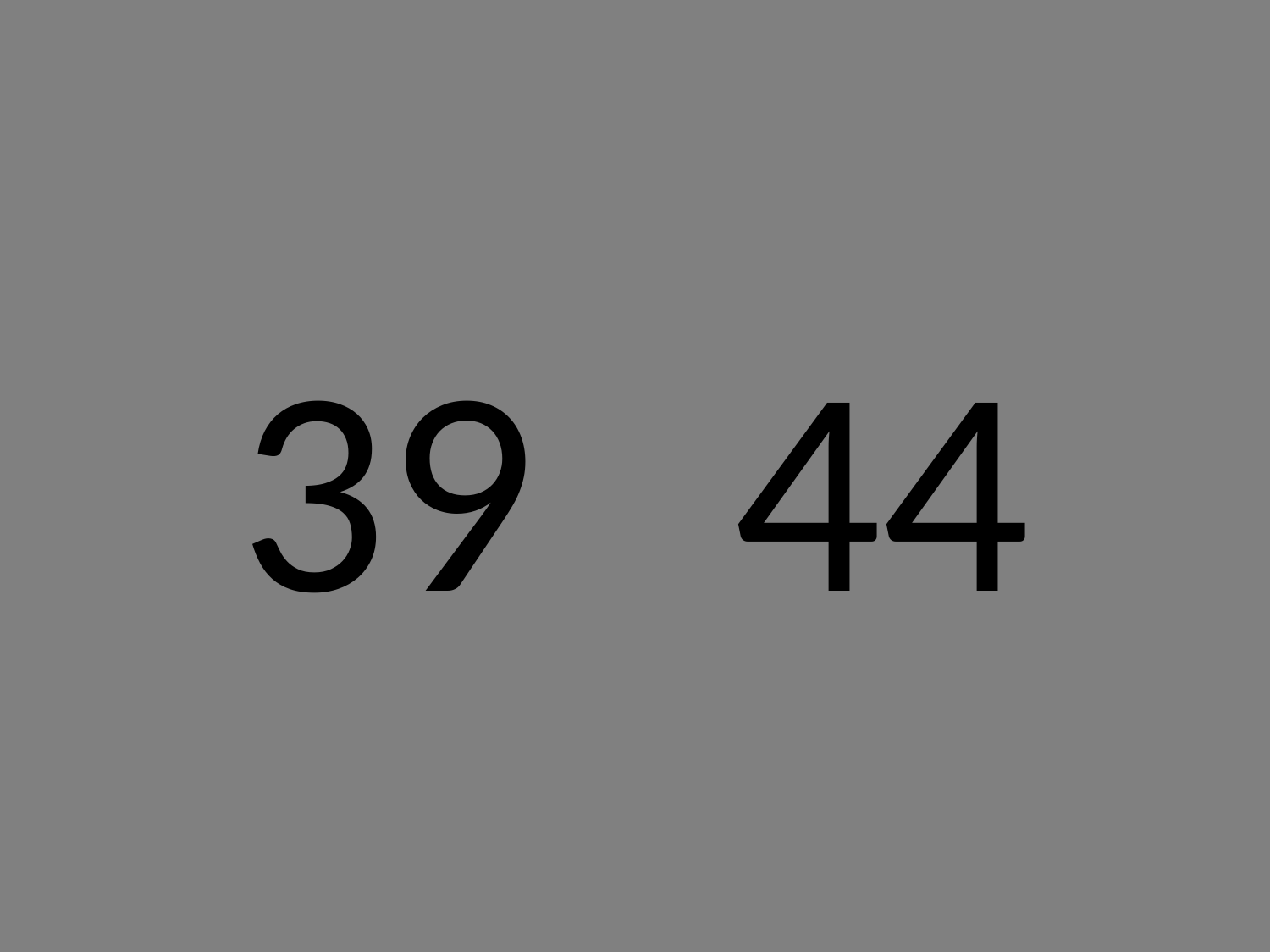

39 44

## Slide 2
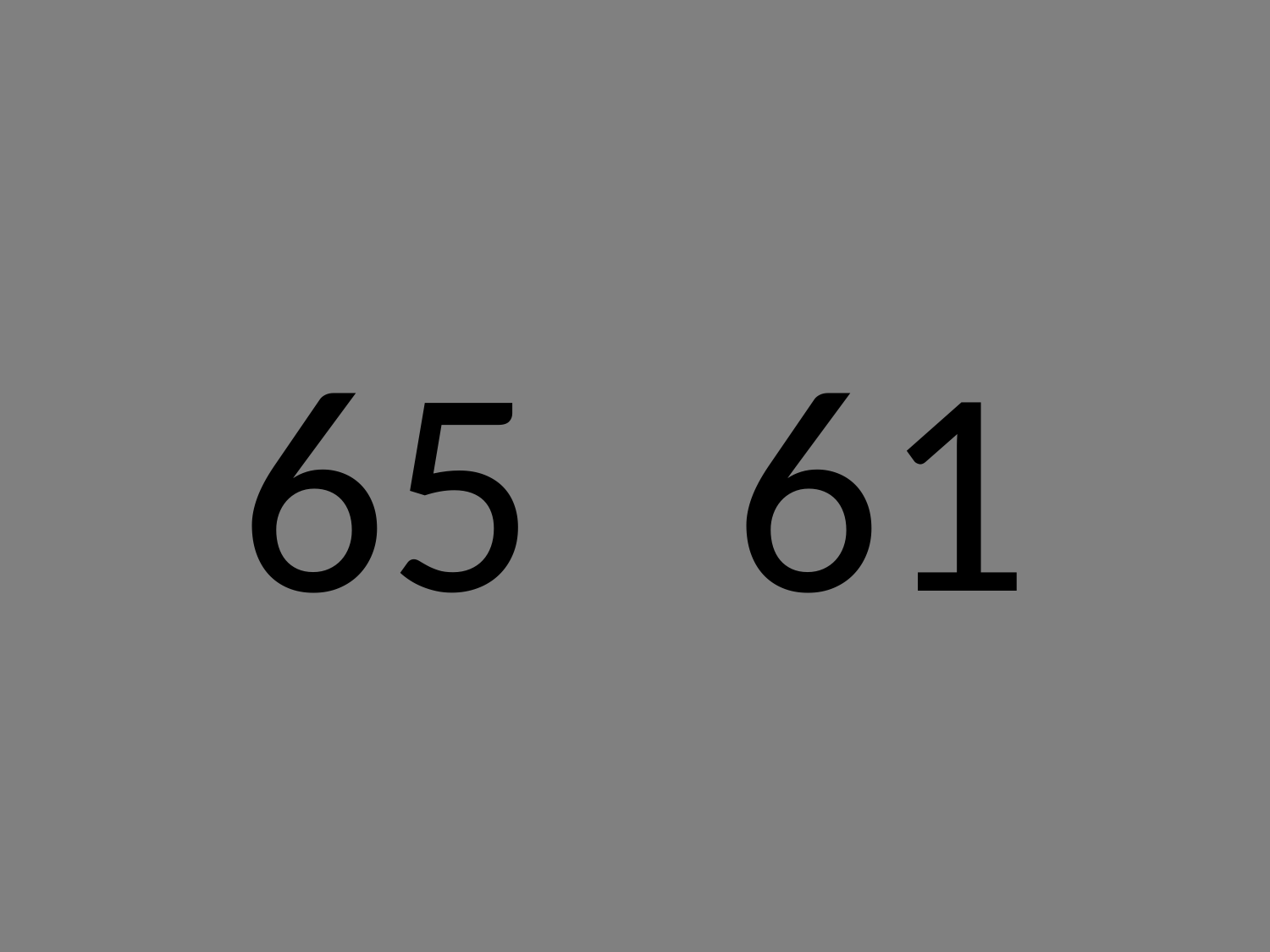

65 61

## Slide 3
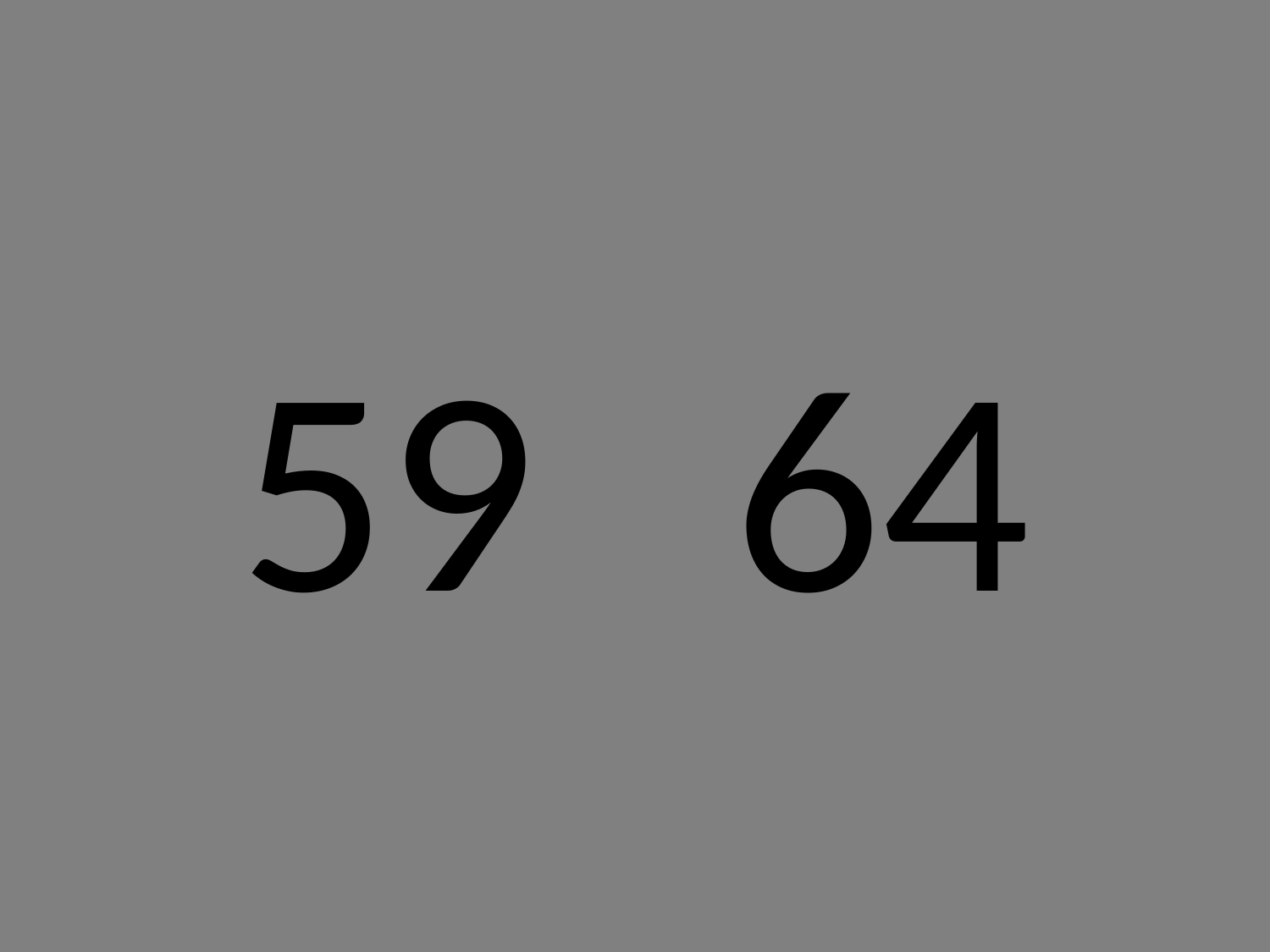

59 64

## Slide 4
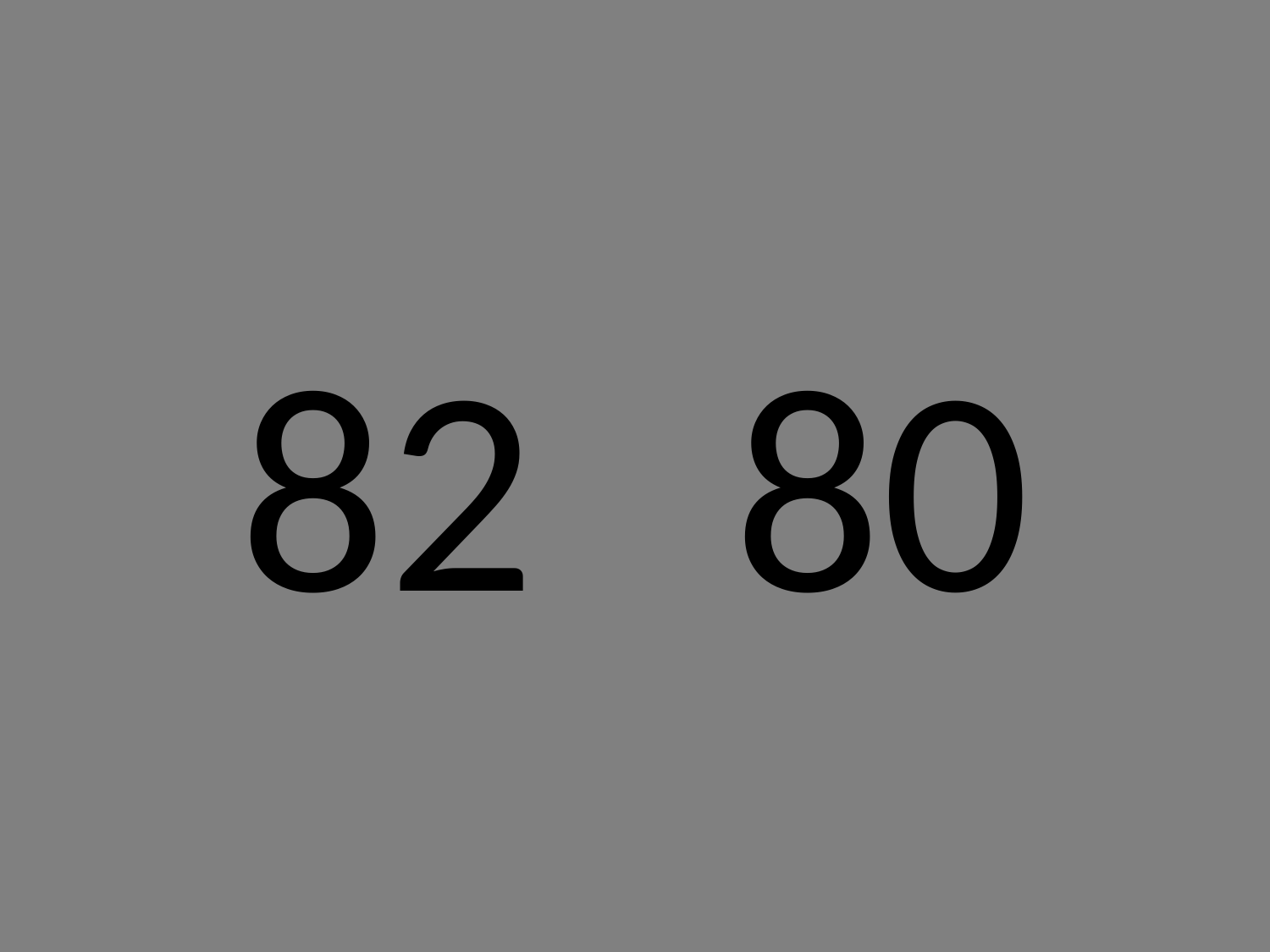

82 80

## Slide 5
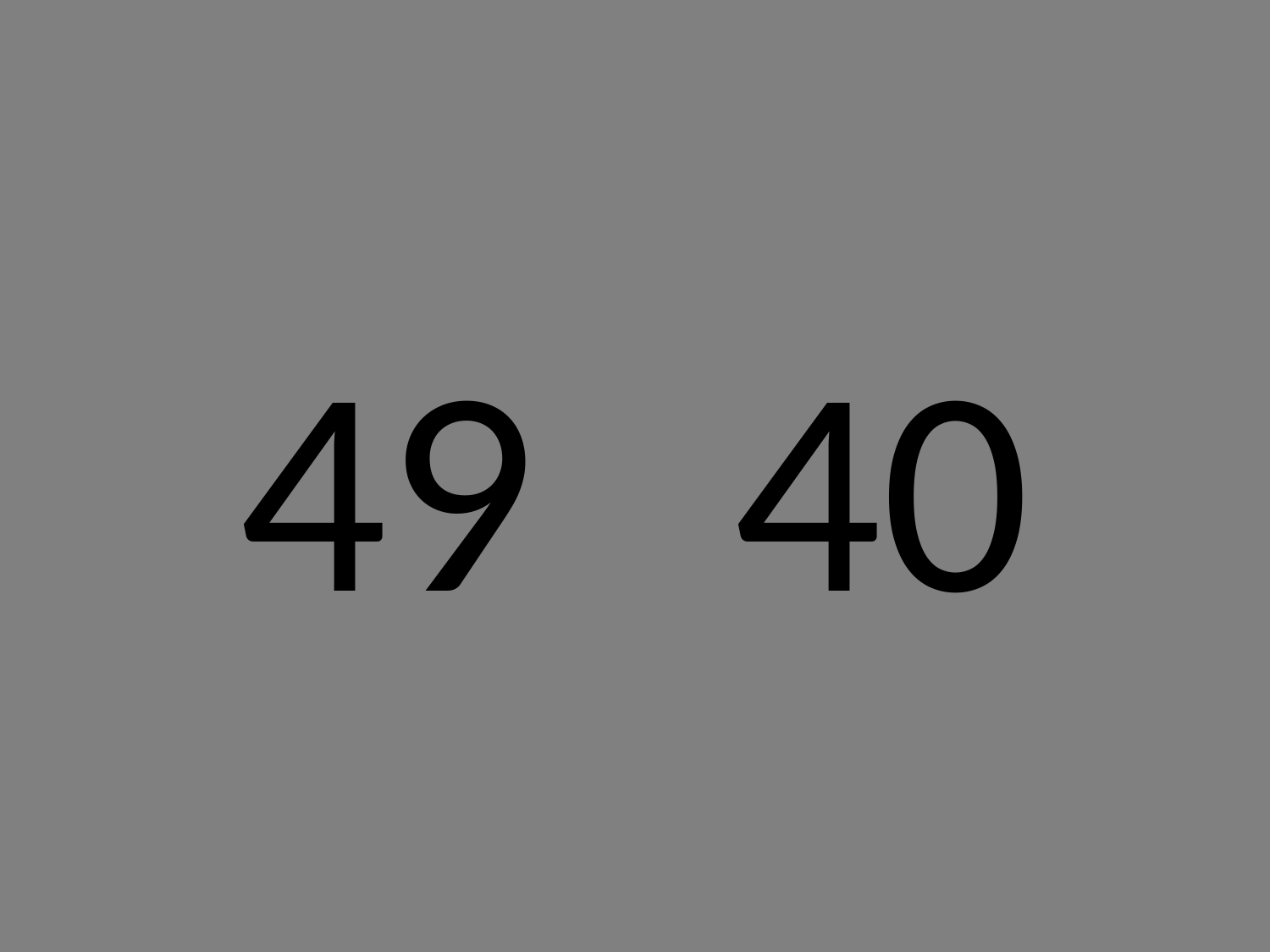

49 40

## Slide 6
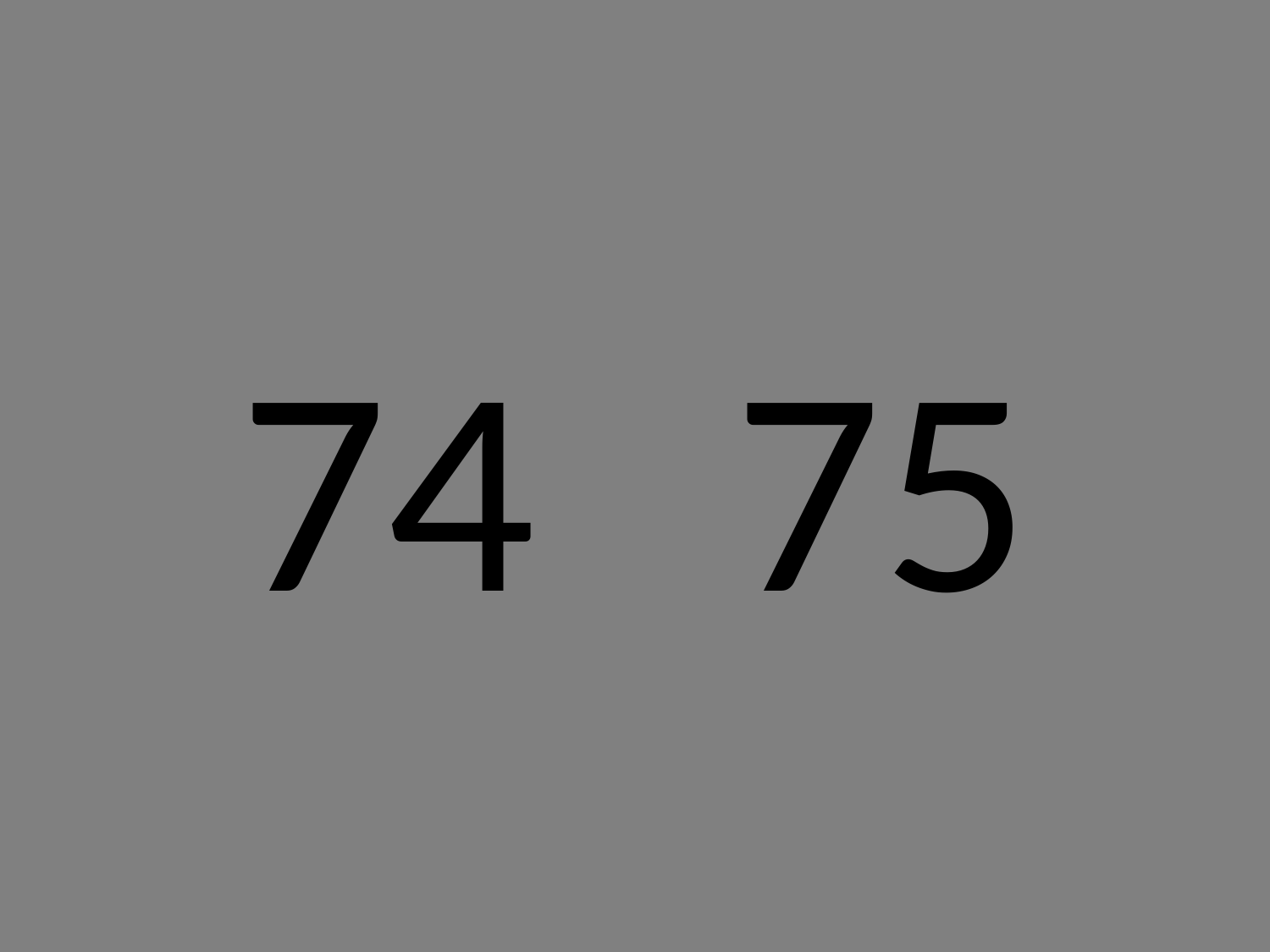

74 75

## Slide 7
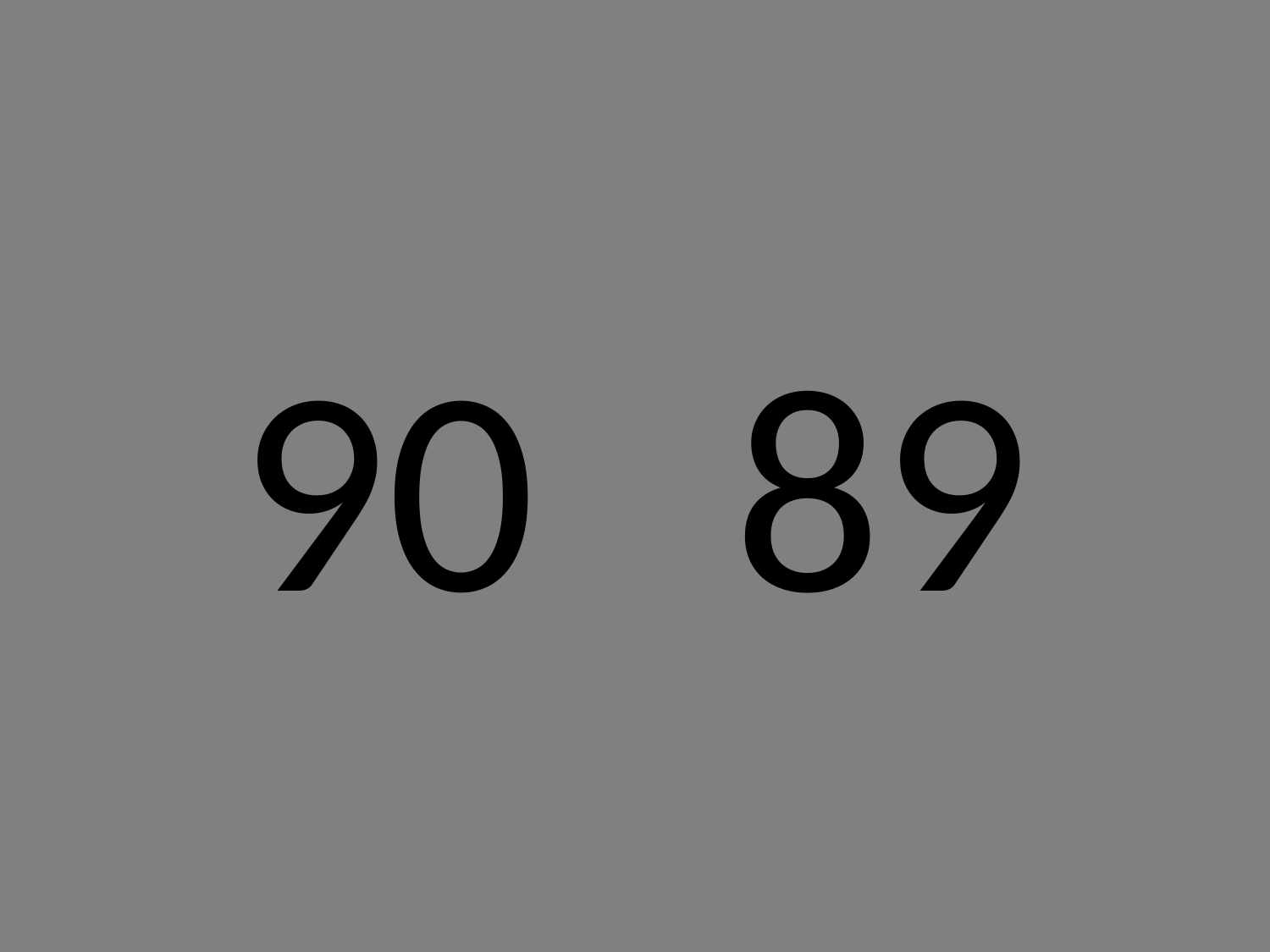

90 89

## Slide 8
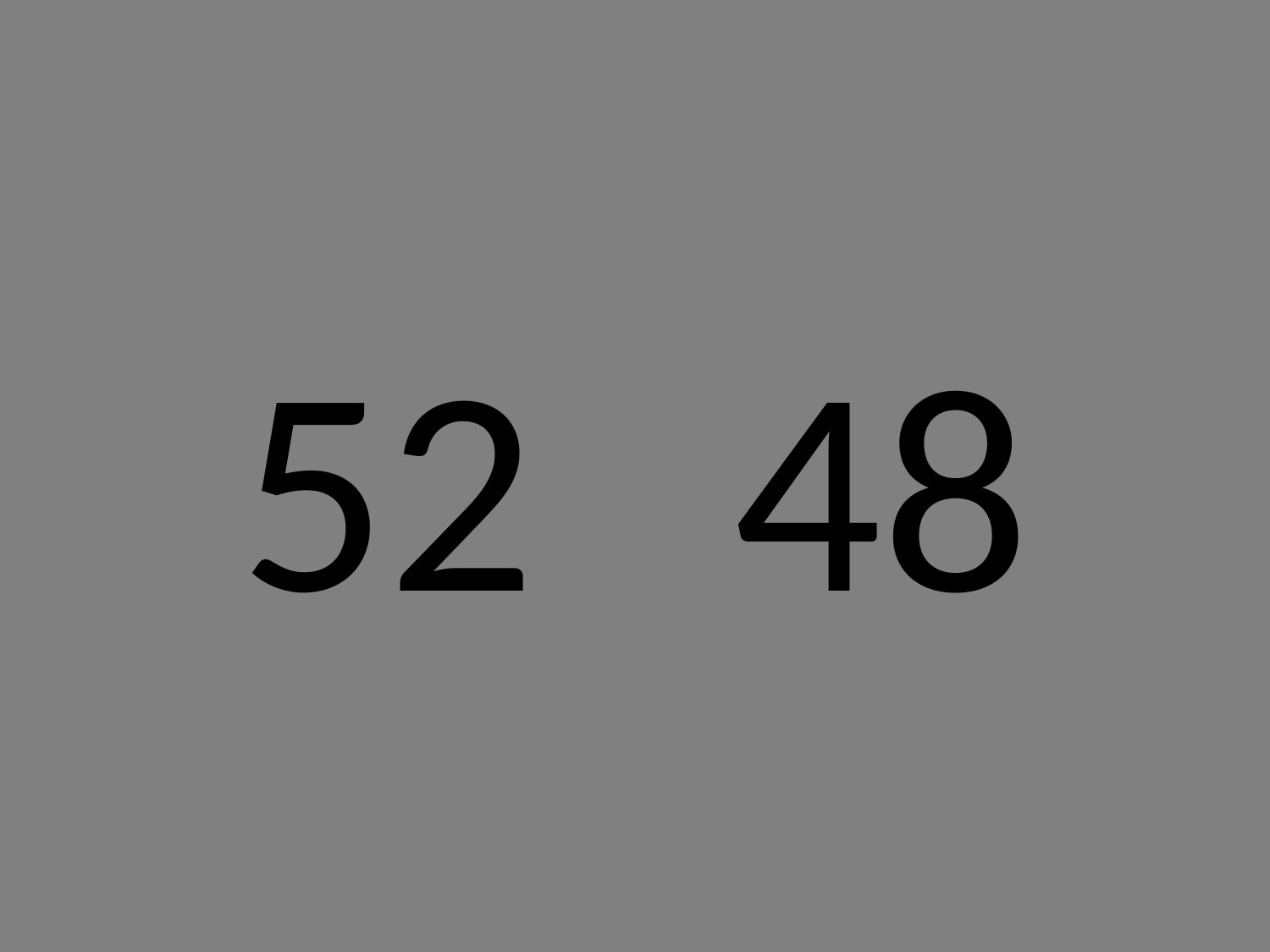

52 48

## Slide 9
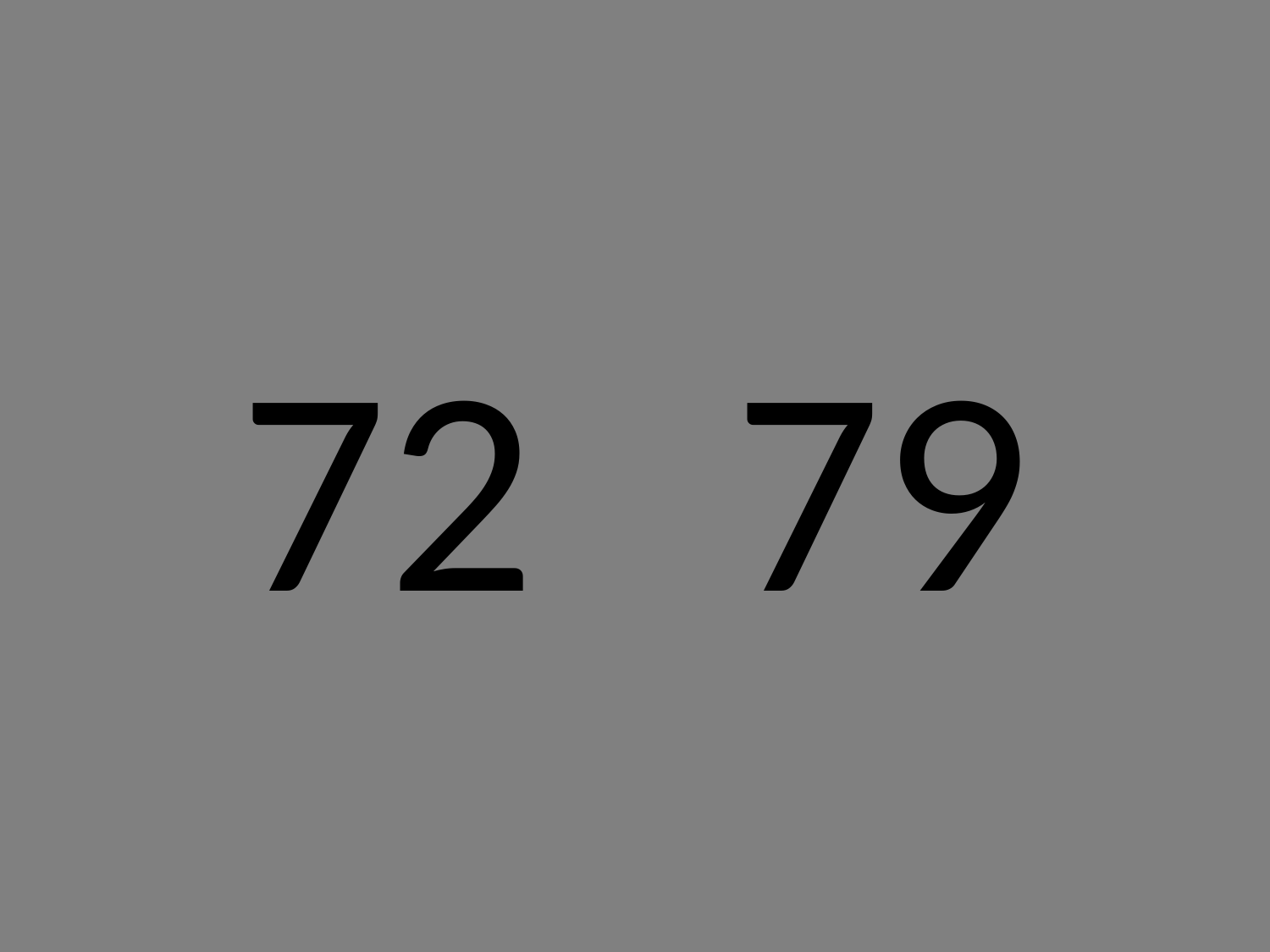

72 79

## Slide 10
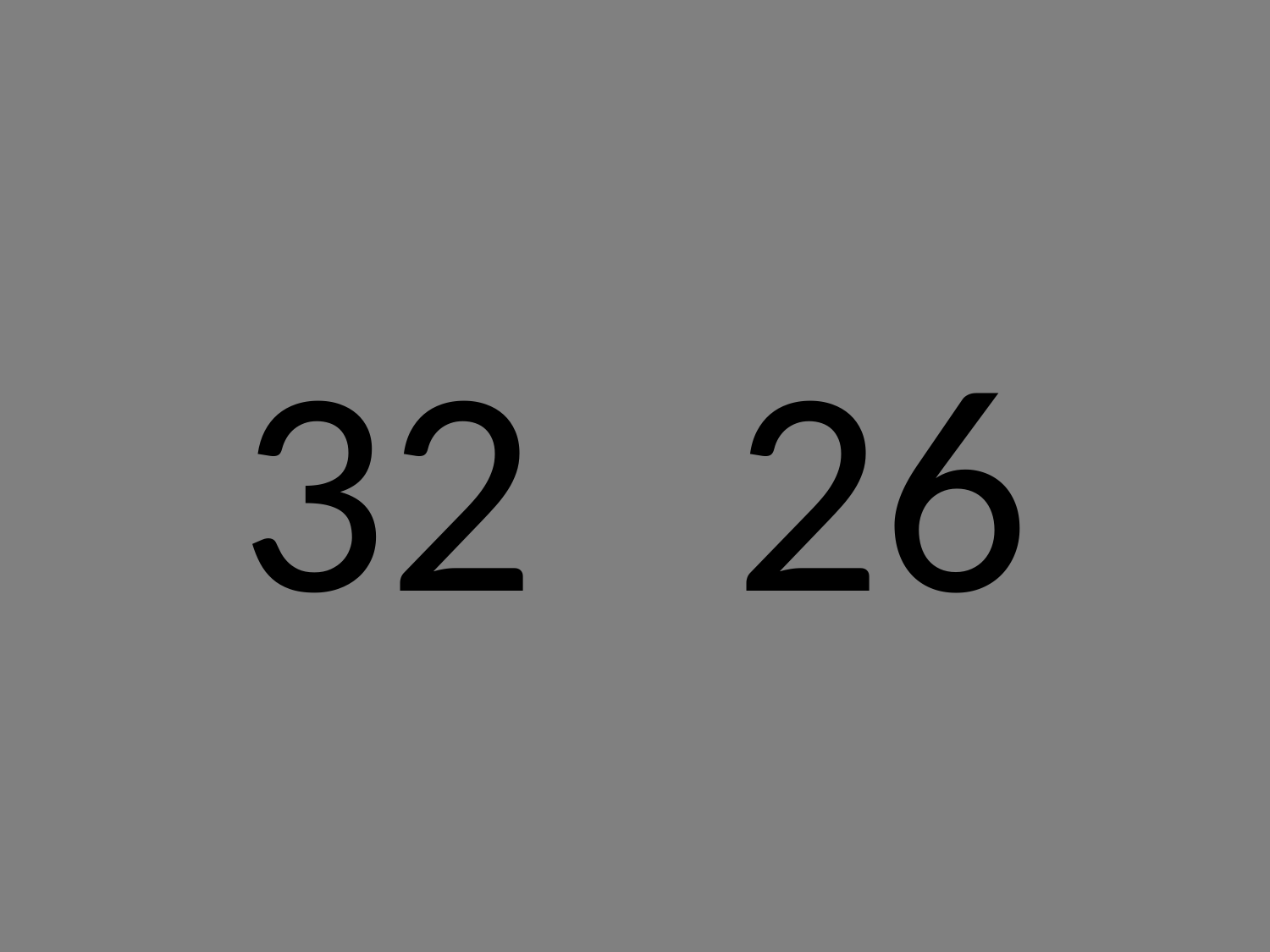

32 26

## Slide 11
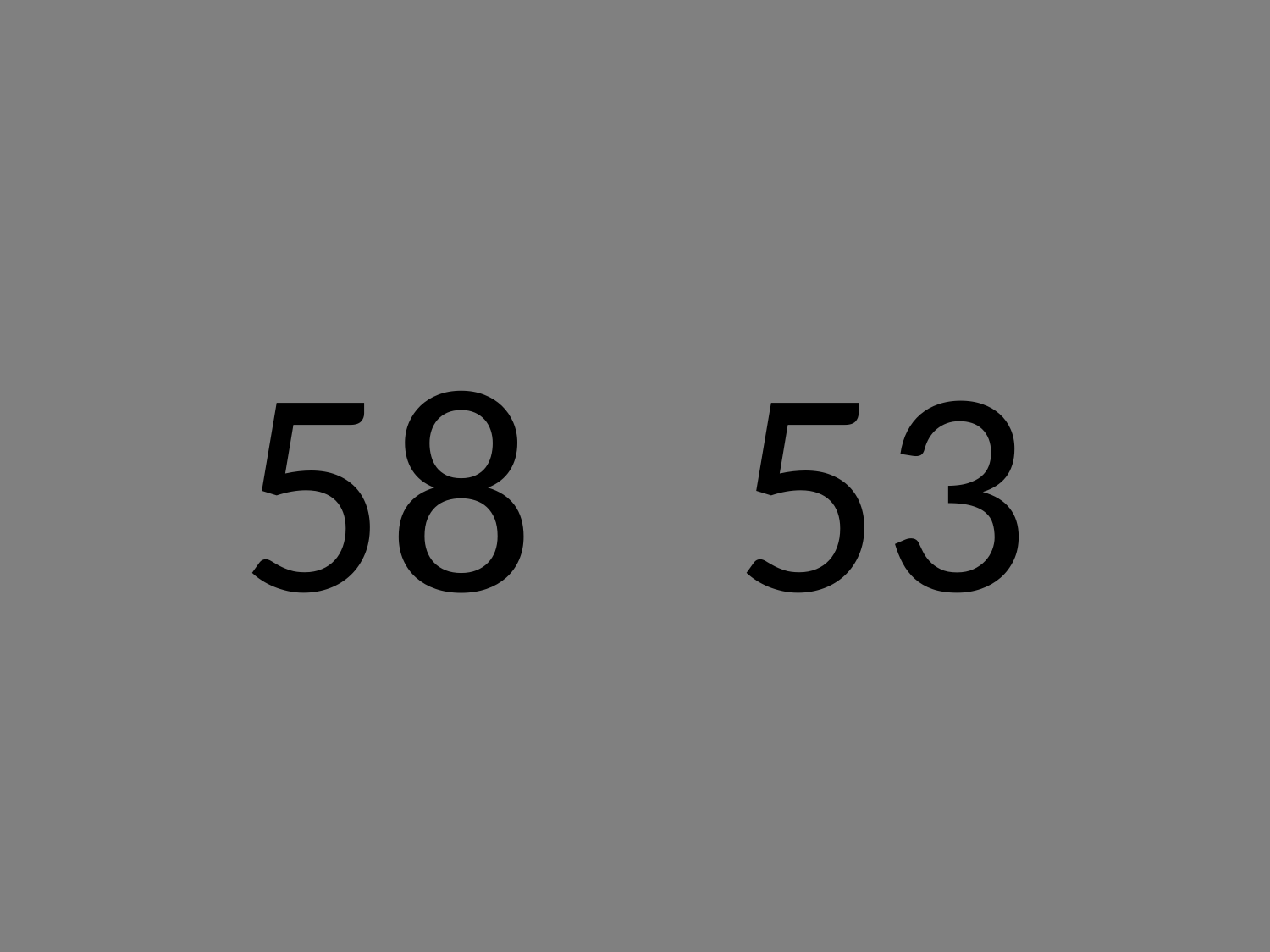

58 53

## Slide 12
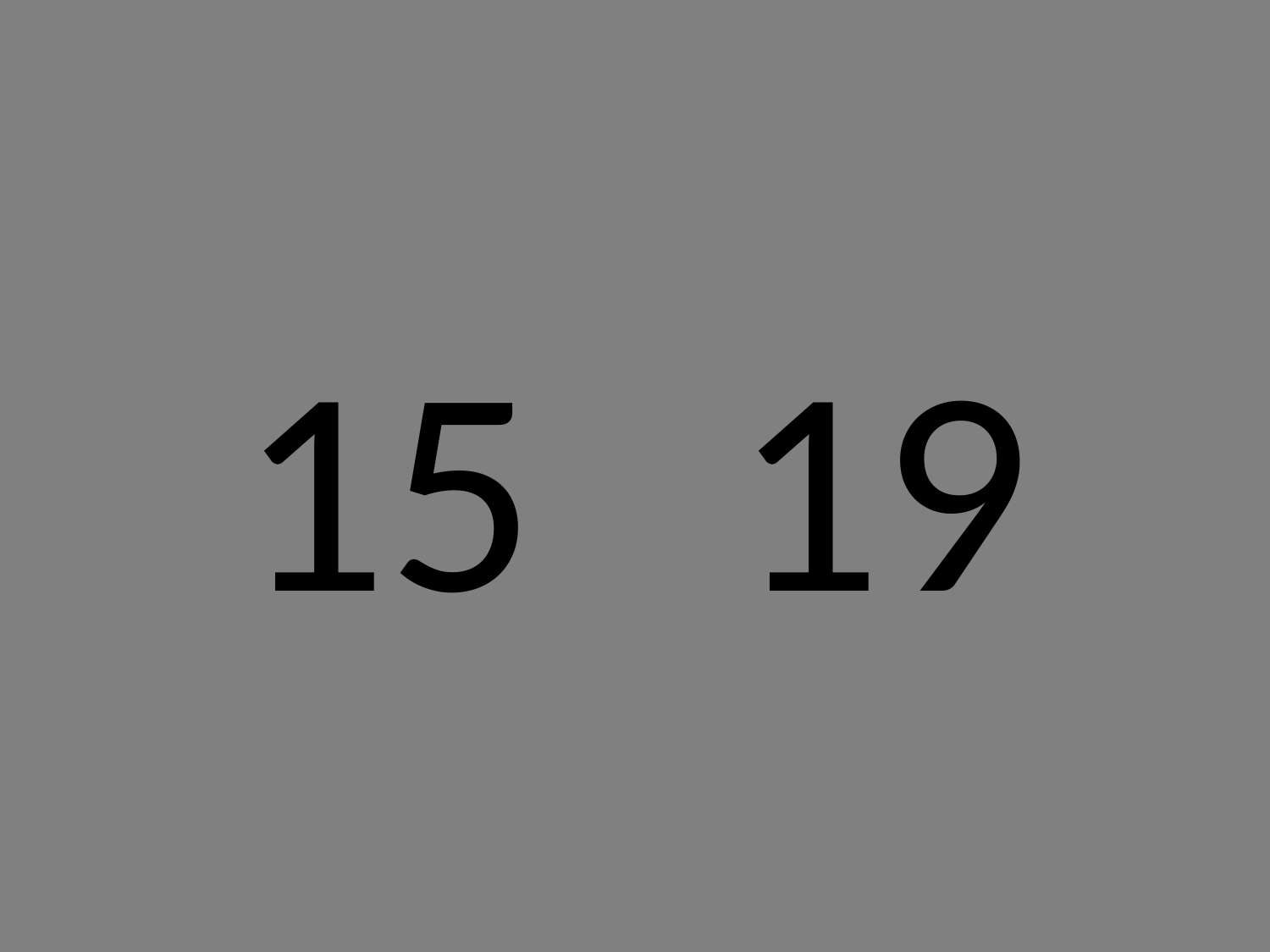

15 19

## Slide 13
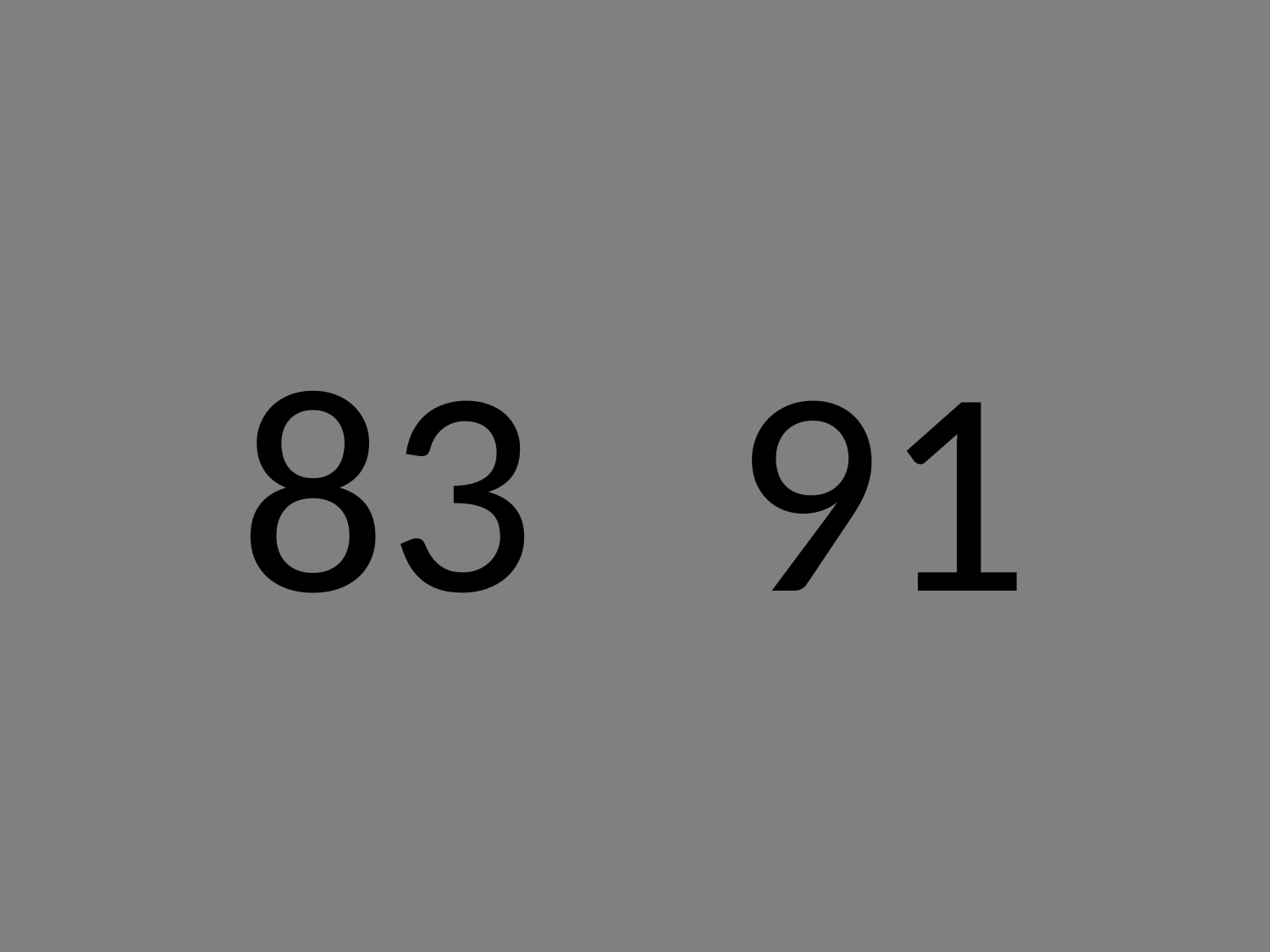

83 91

## Slide 14
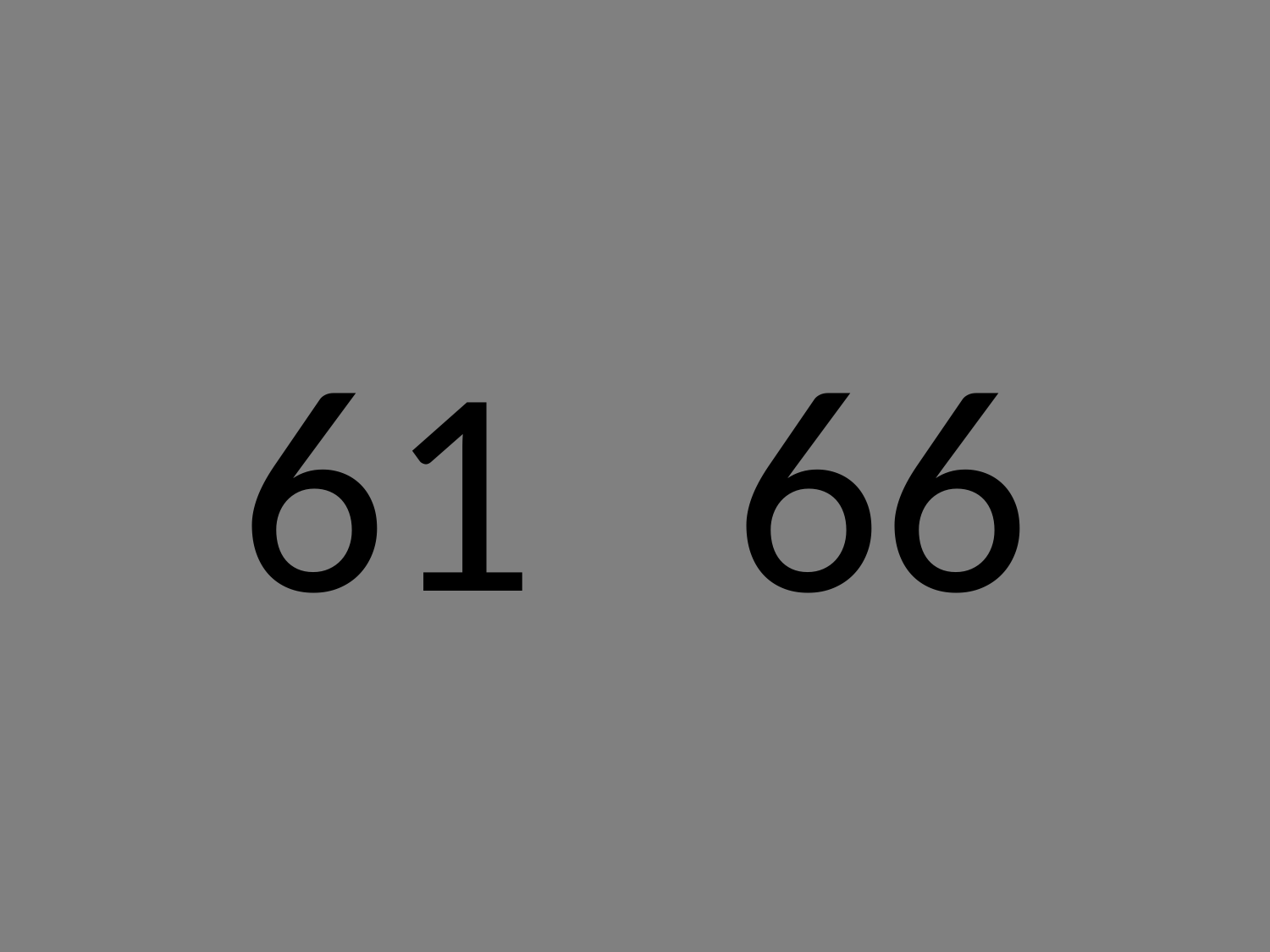

61 66

## Slide 15
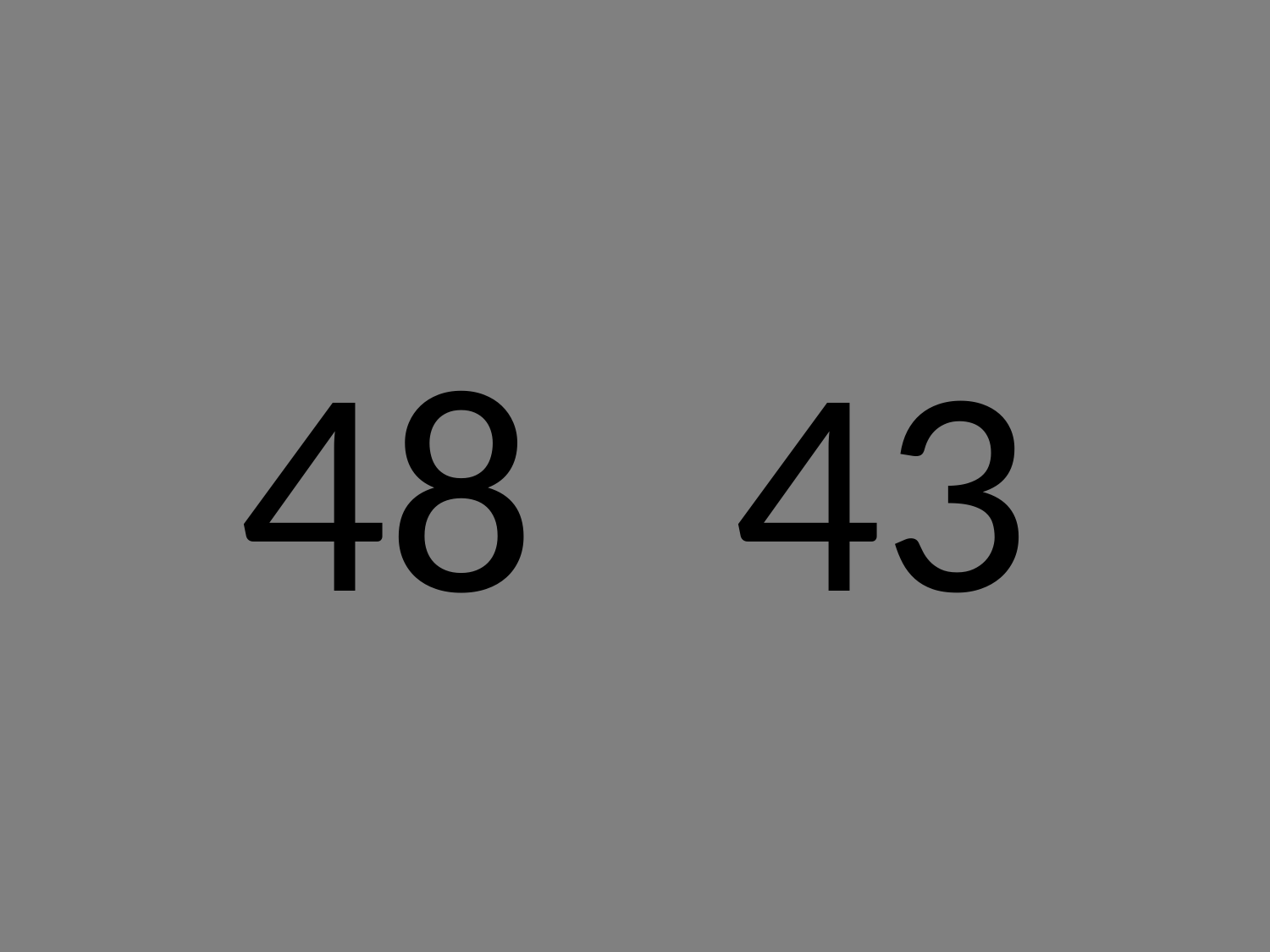

48 43

## Slide 16
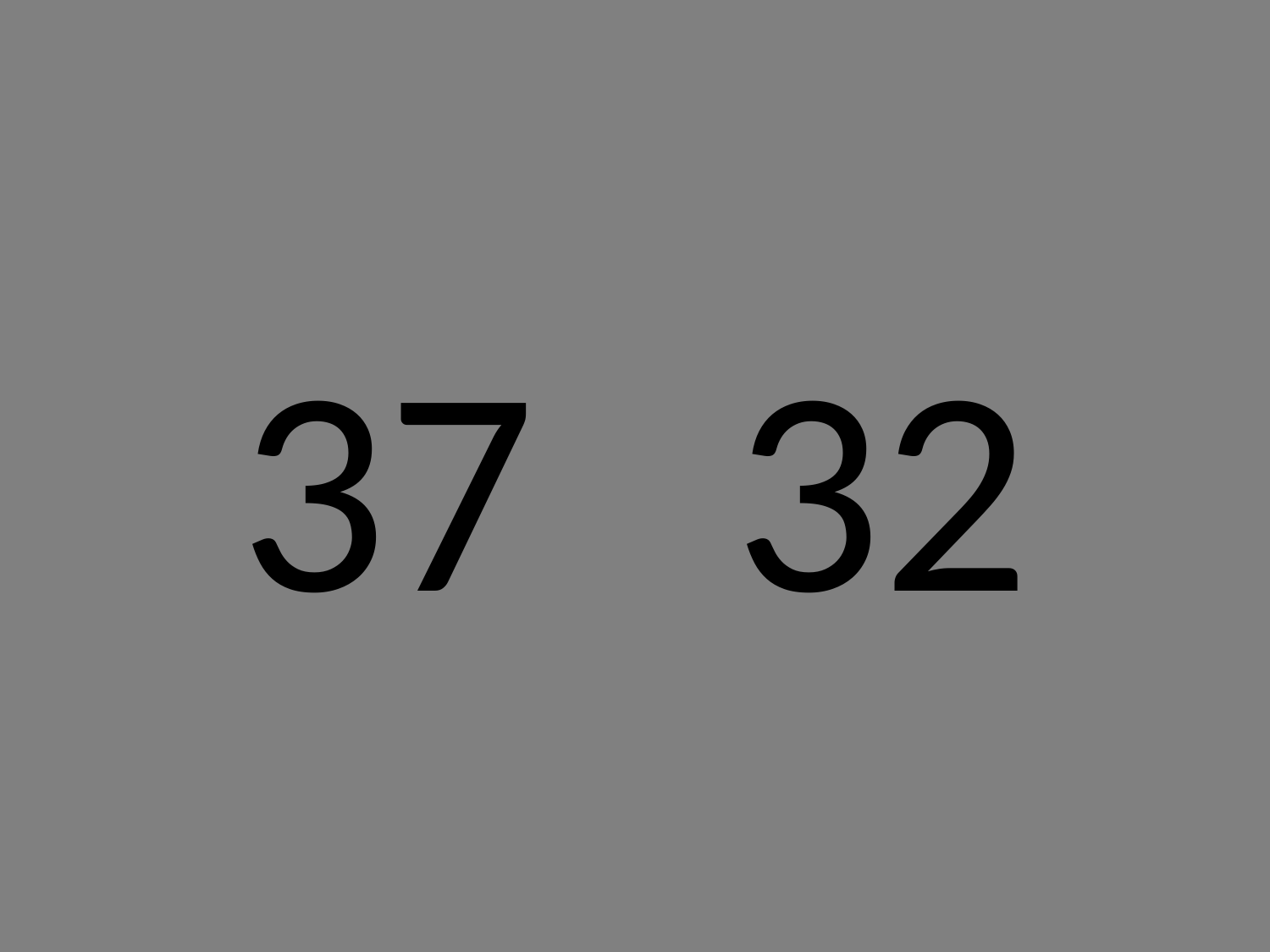

37 32

## Slide 17
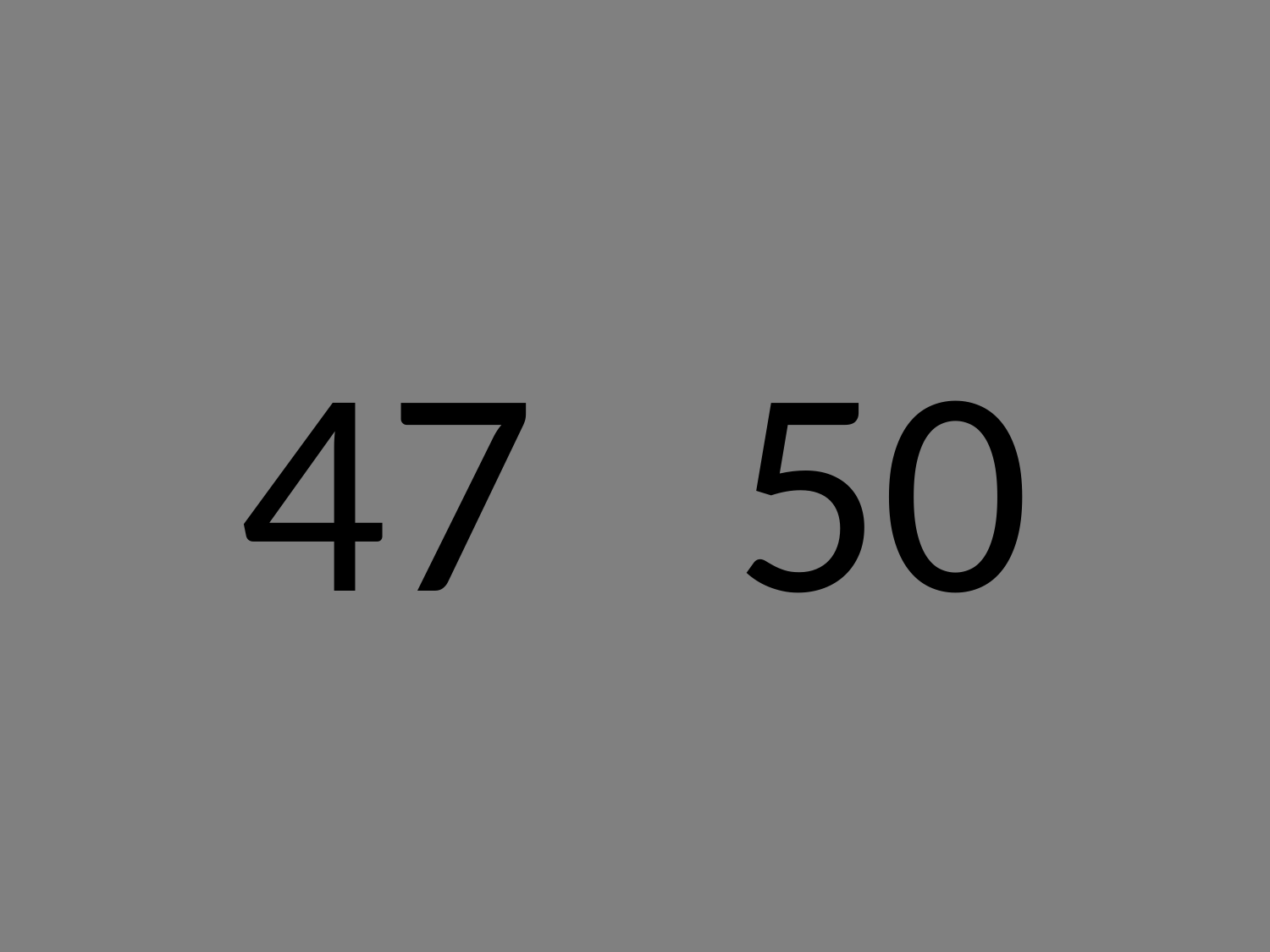

47 50

## Slide 18
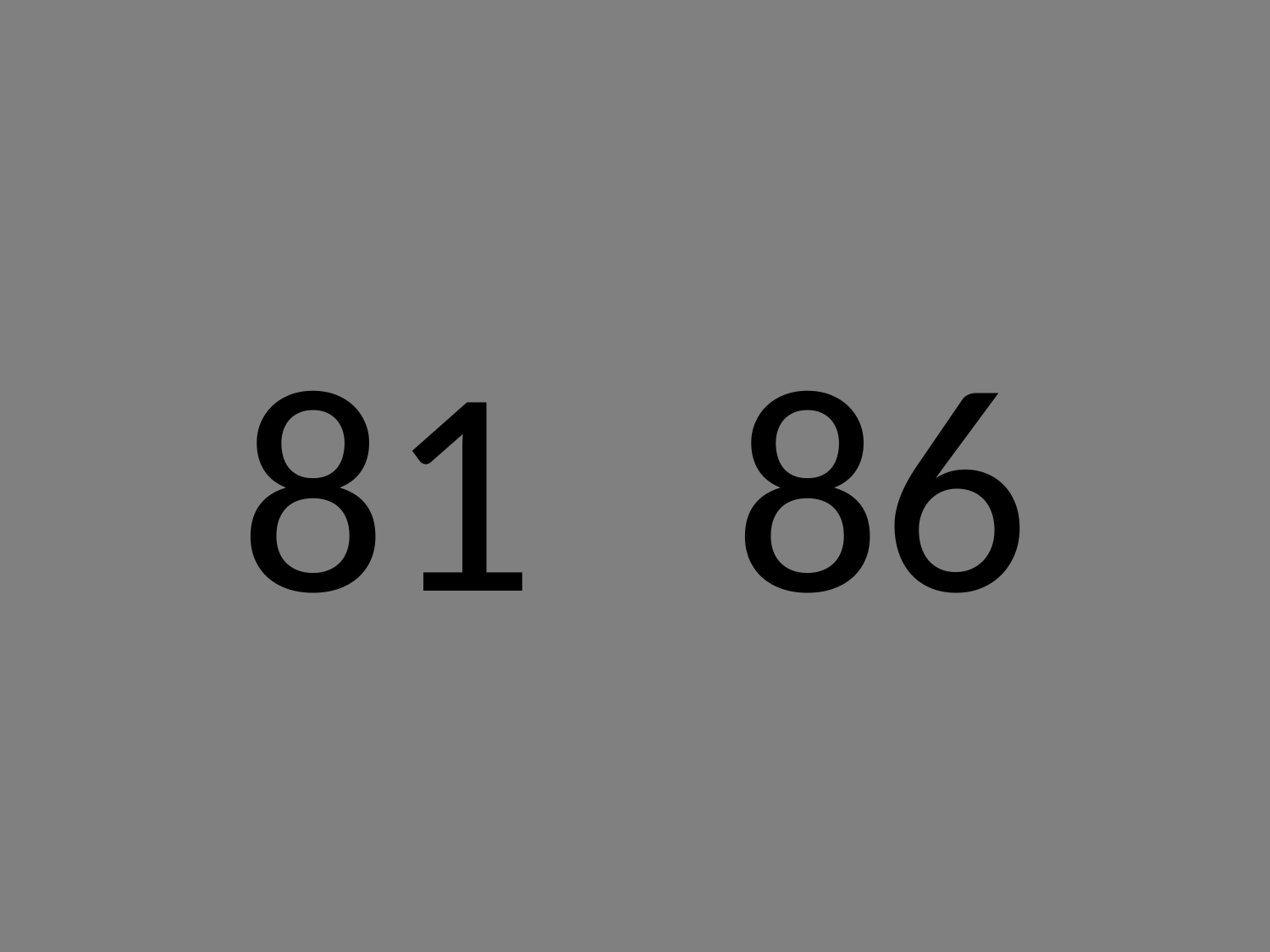

81 86

## Slide 19
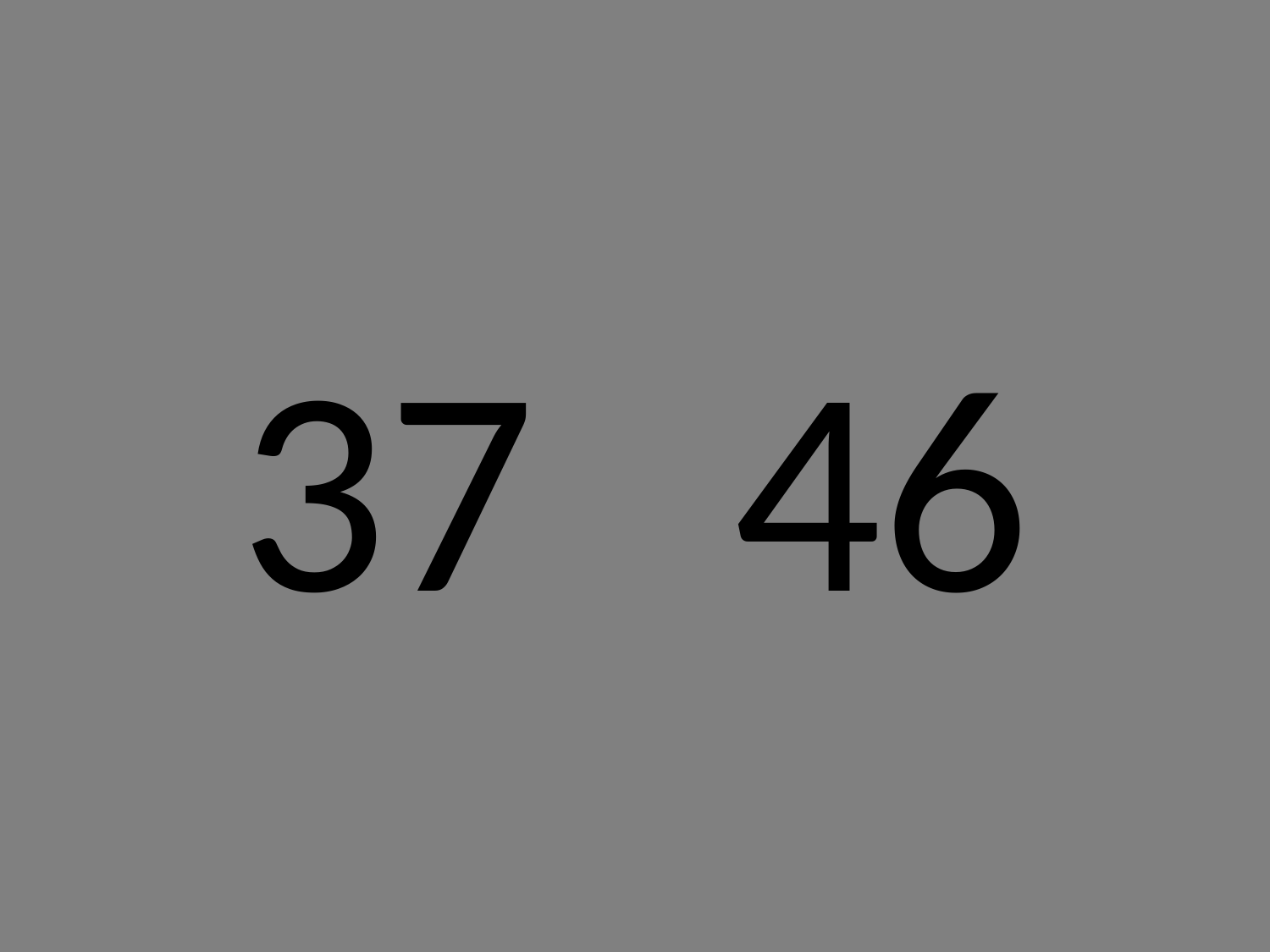

37 46

## Slide 20
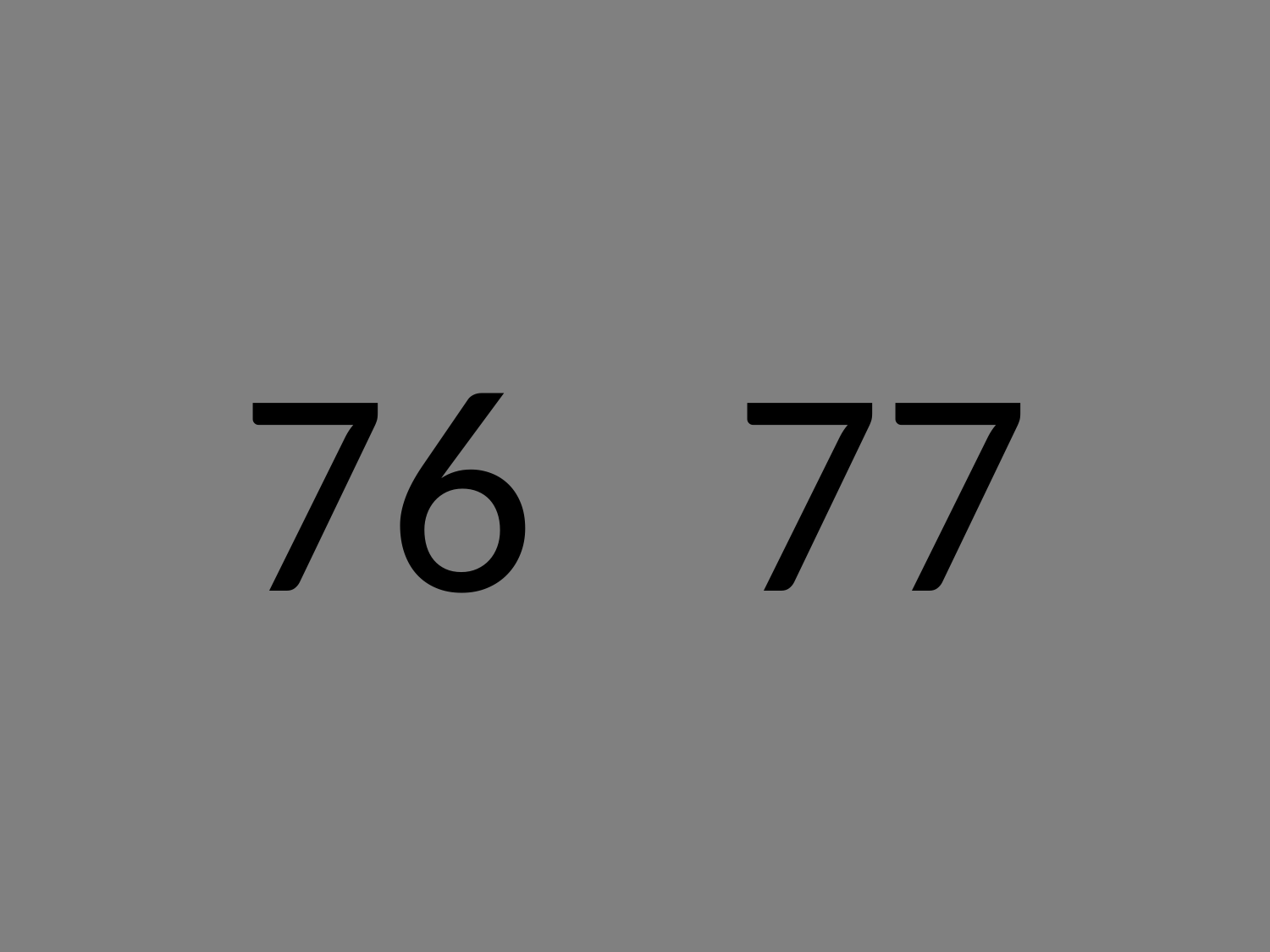

76 77

## Slide 21
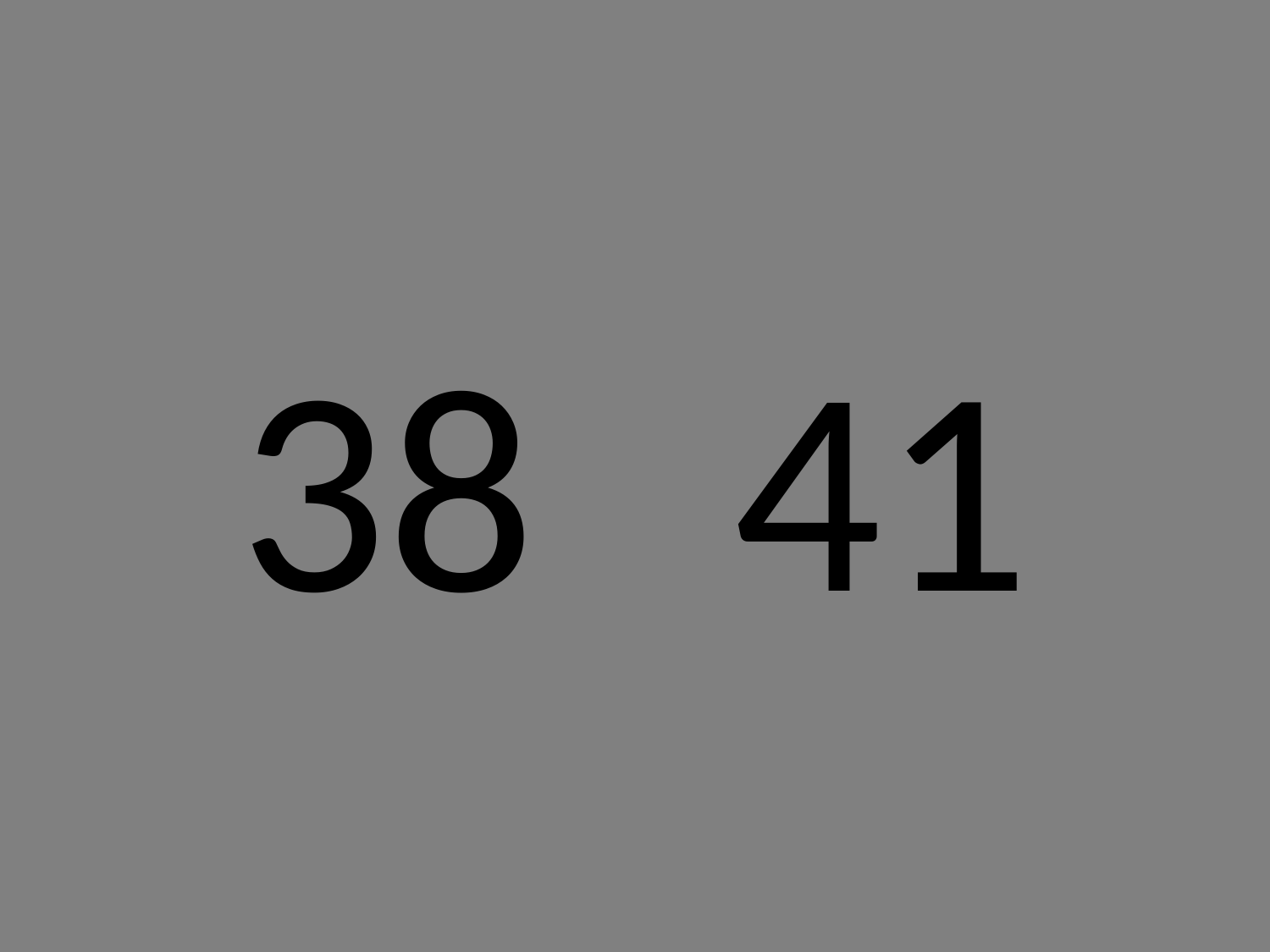

38 41

## Slide 22
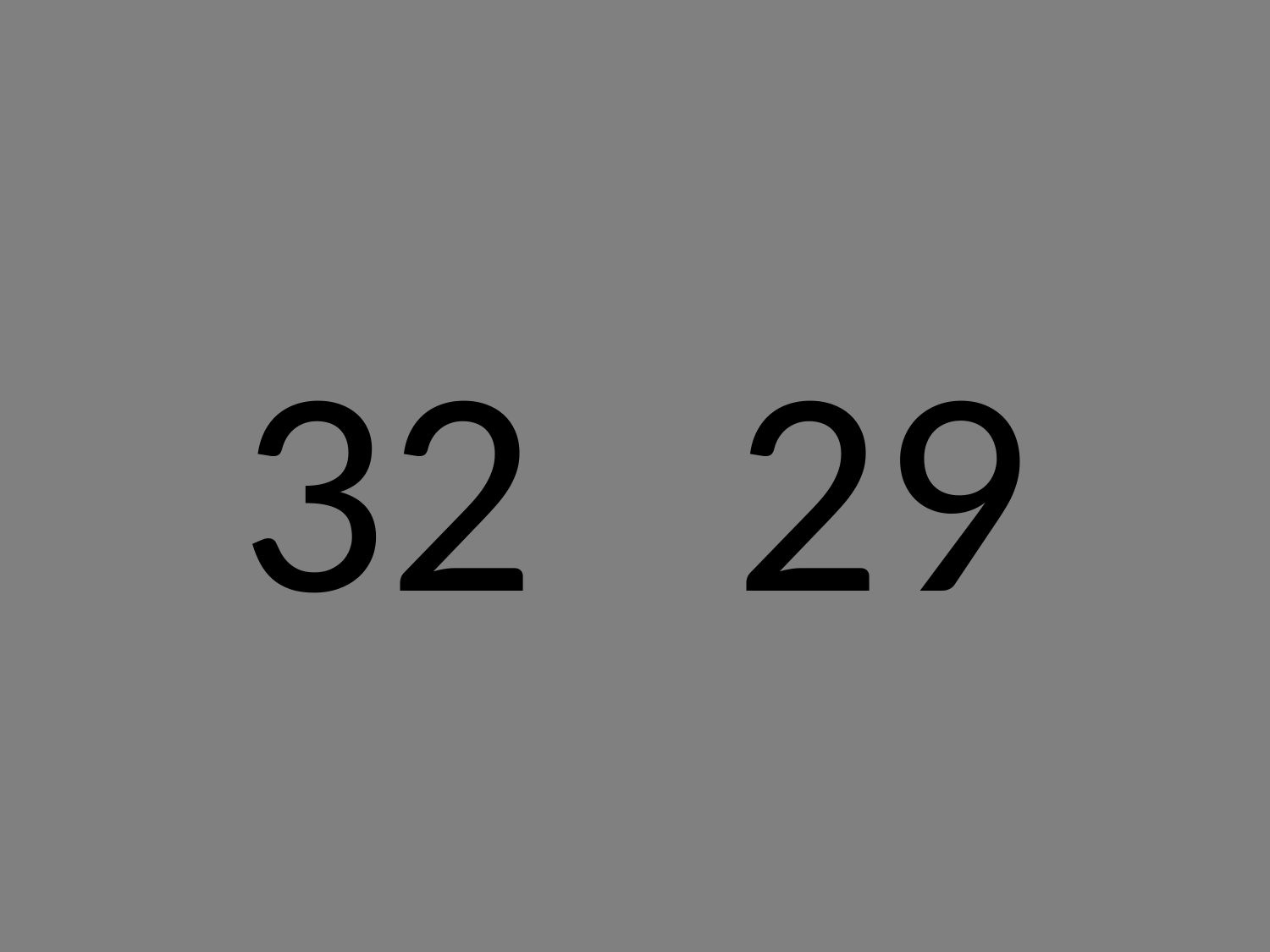

32 29

## Slide 23
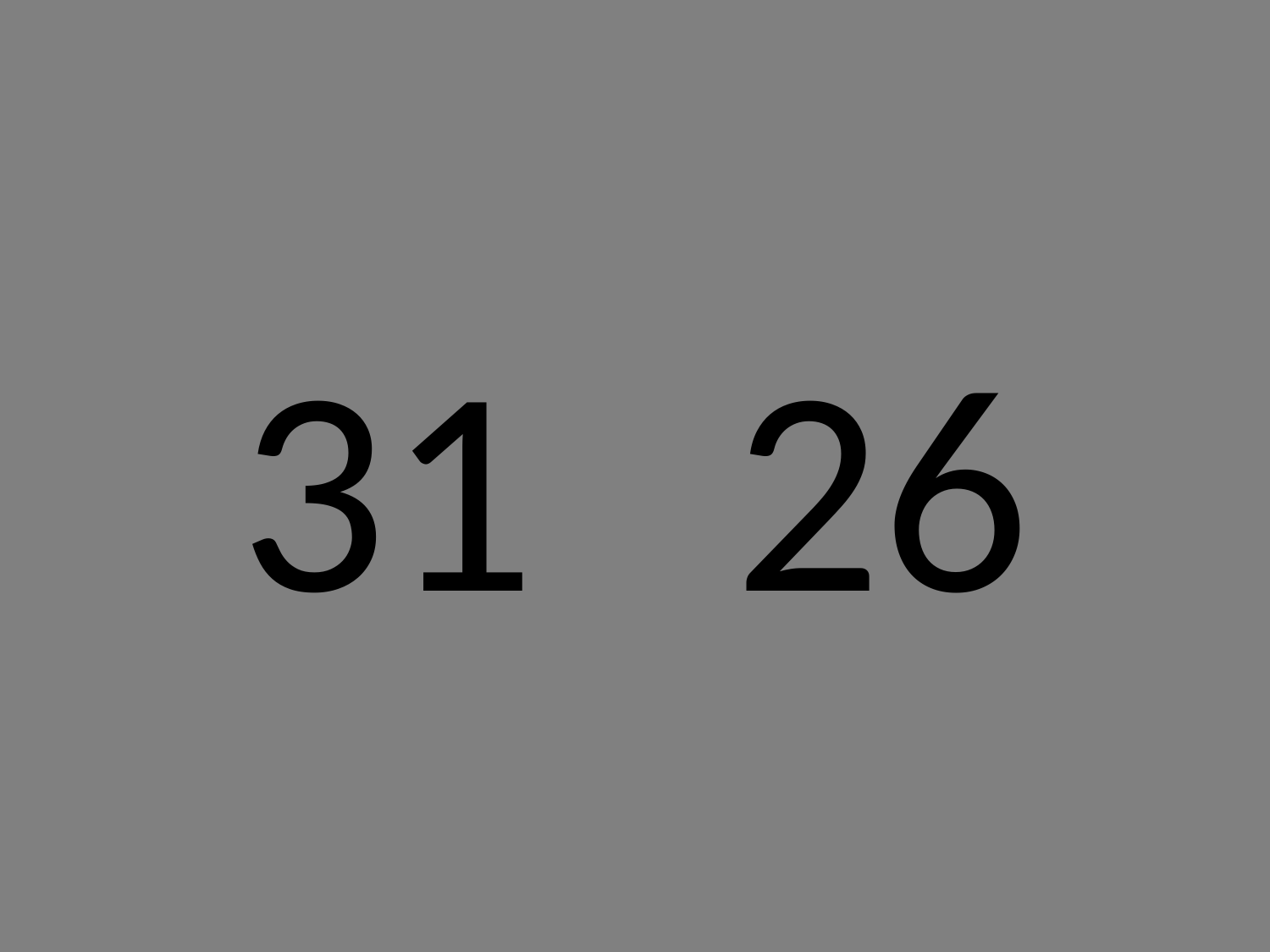

31 26

## Slide 24
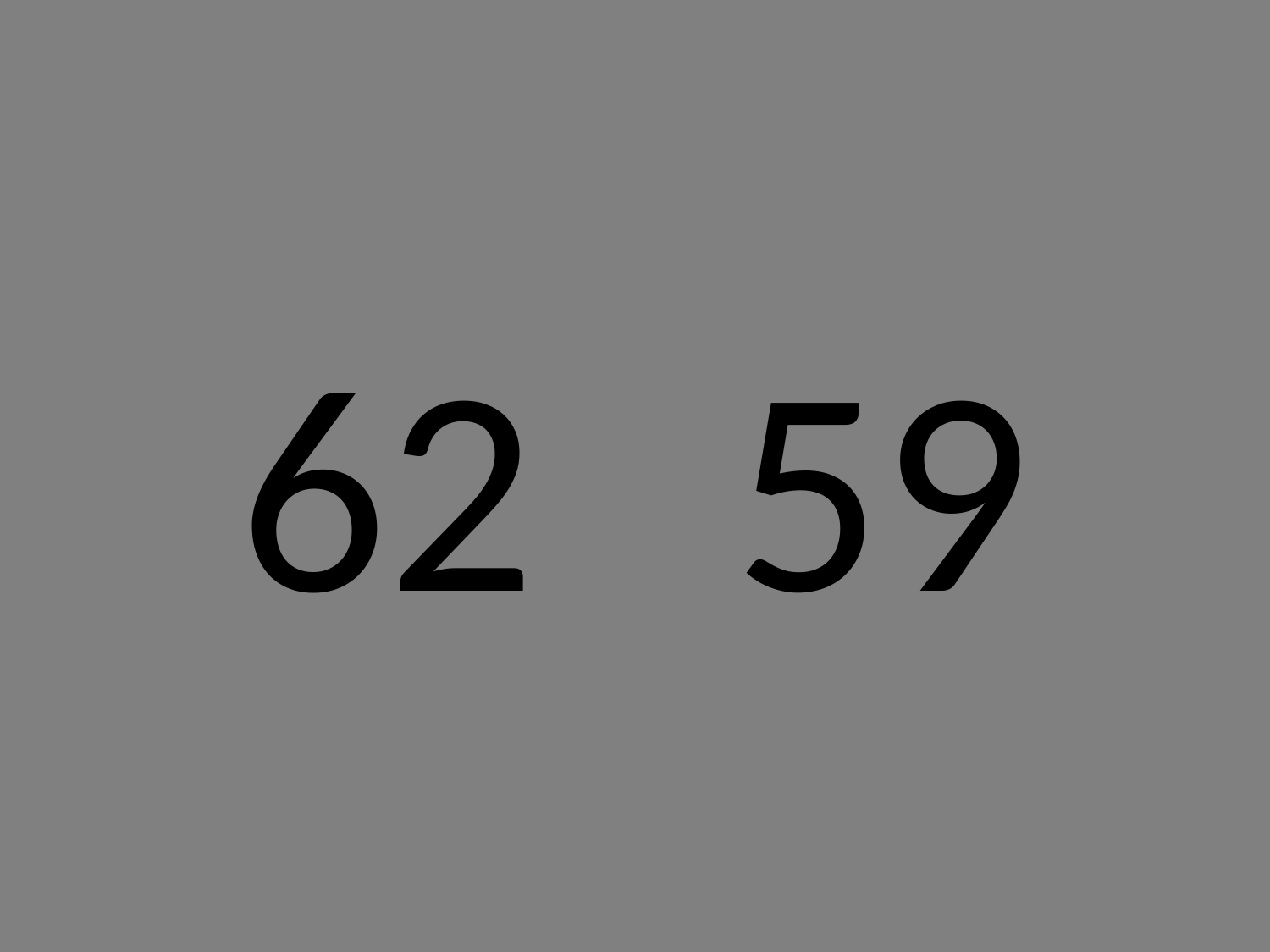

62 59

## Slide 25
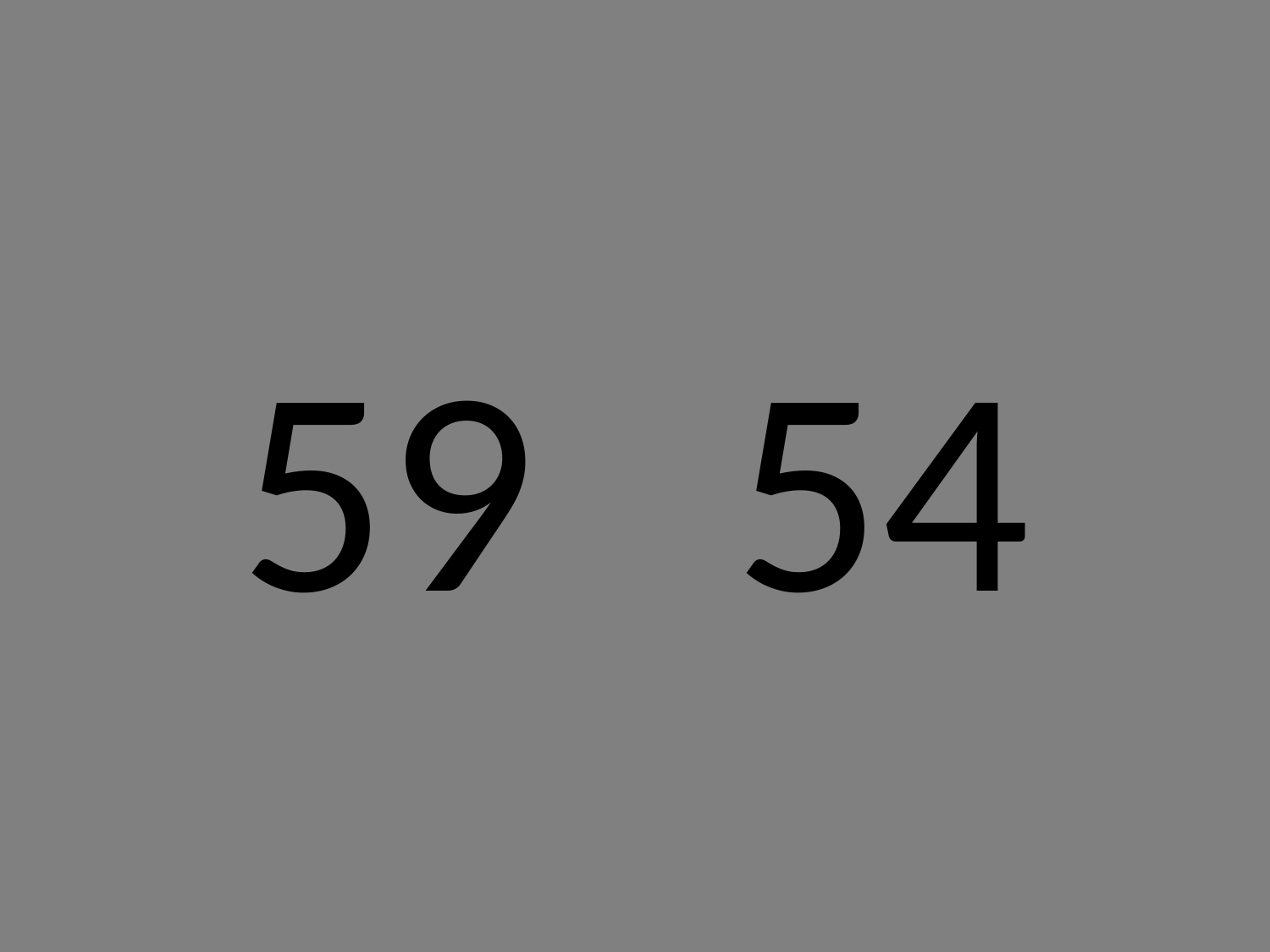

59 54

## Slide 26
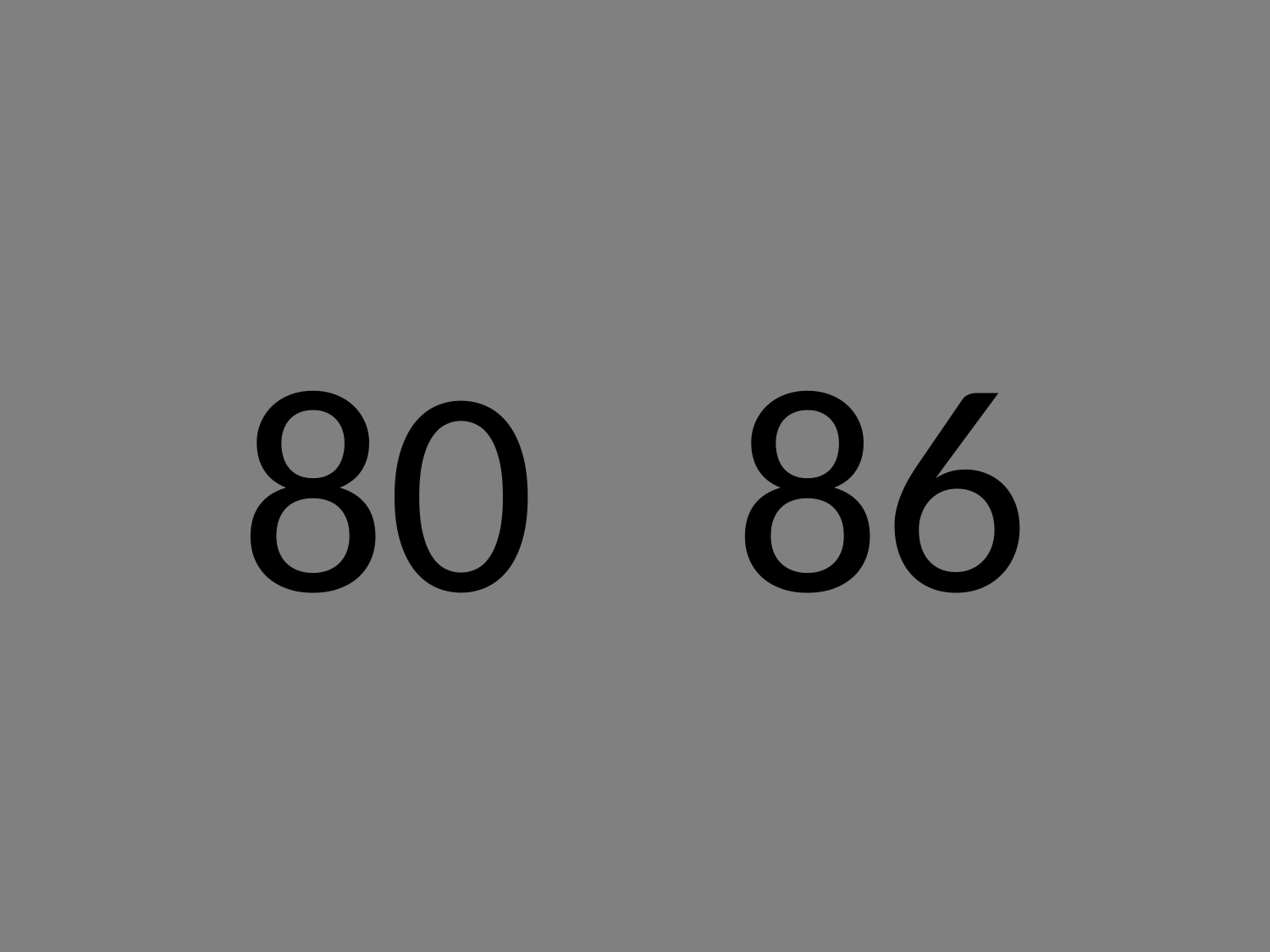

80 86

## Slide 27
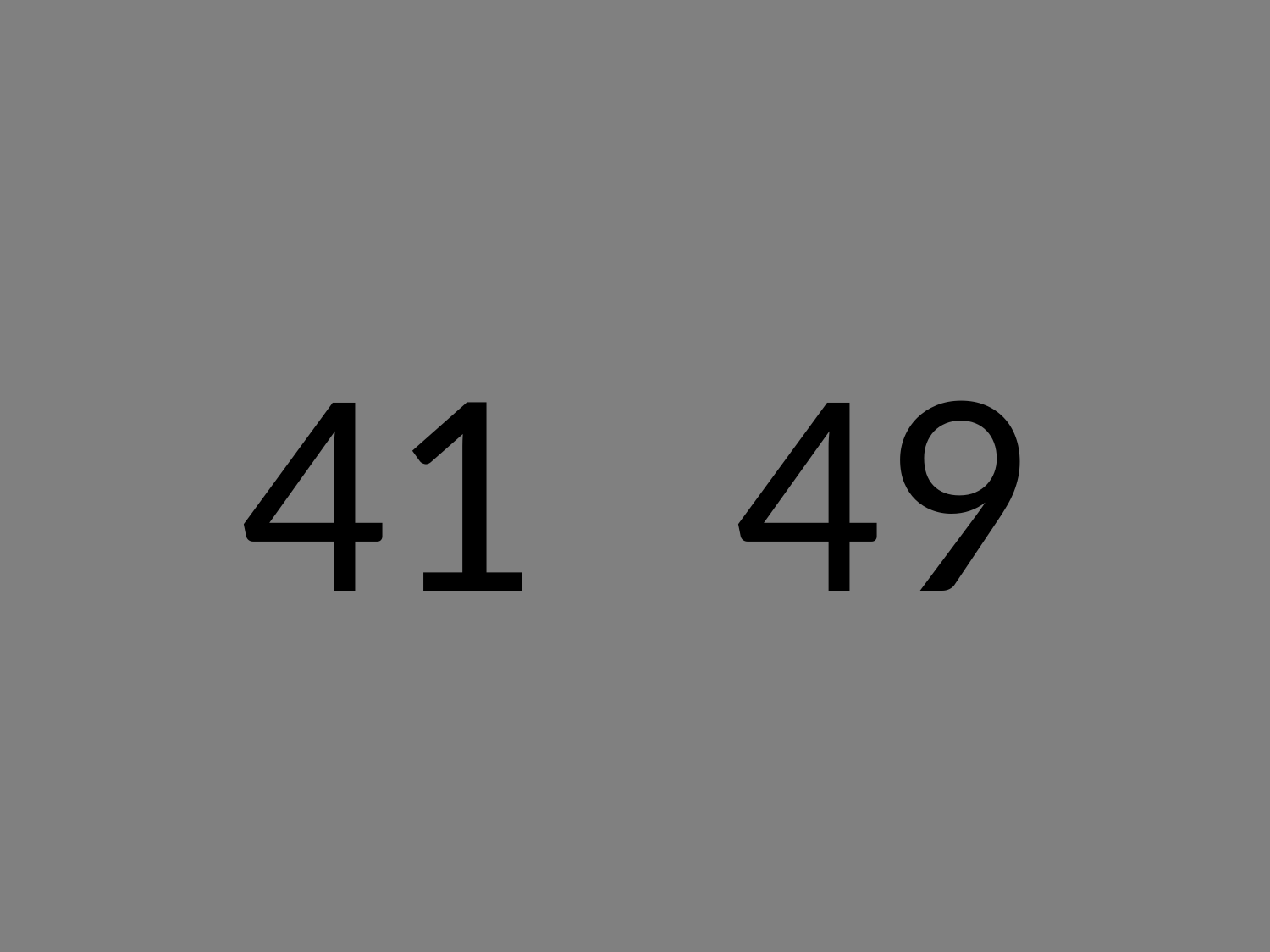

41 49

## Slide 28
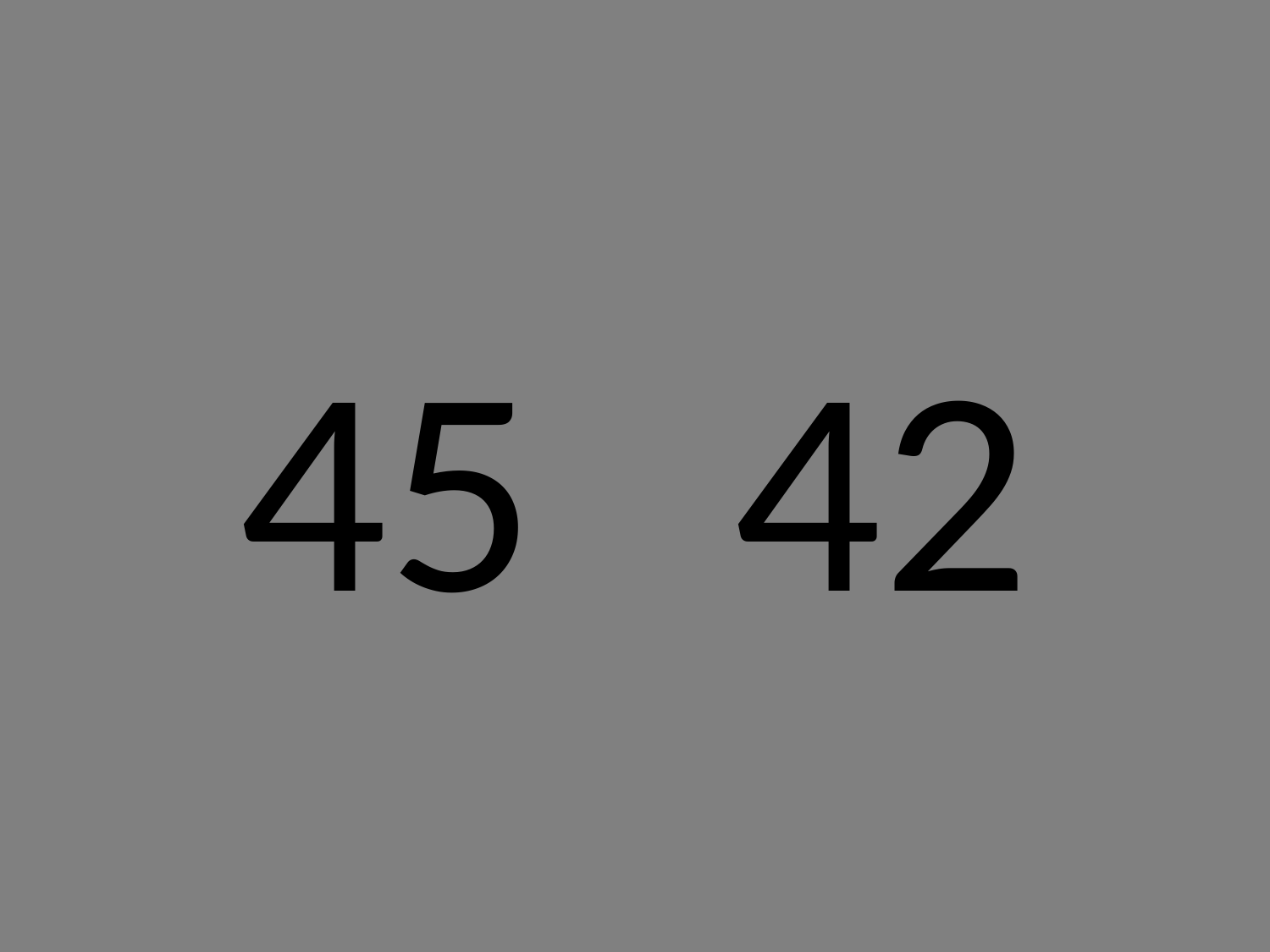

45 42

## Slide 29
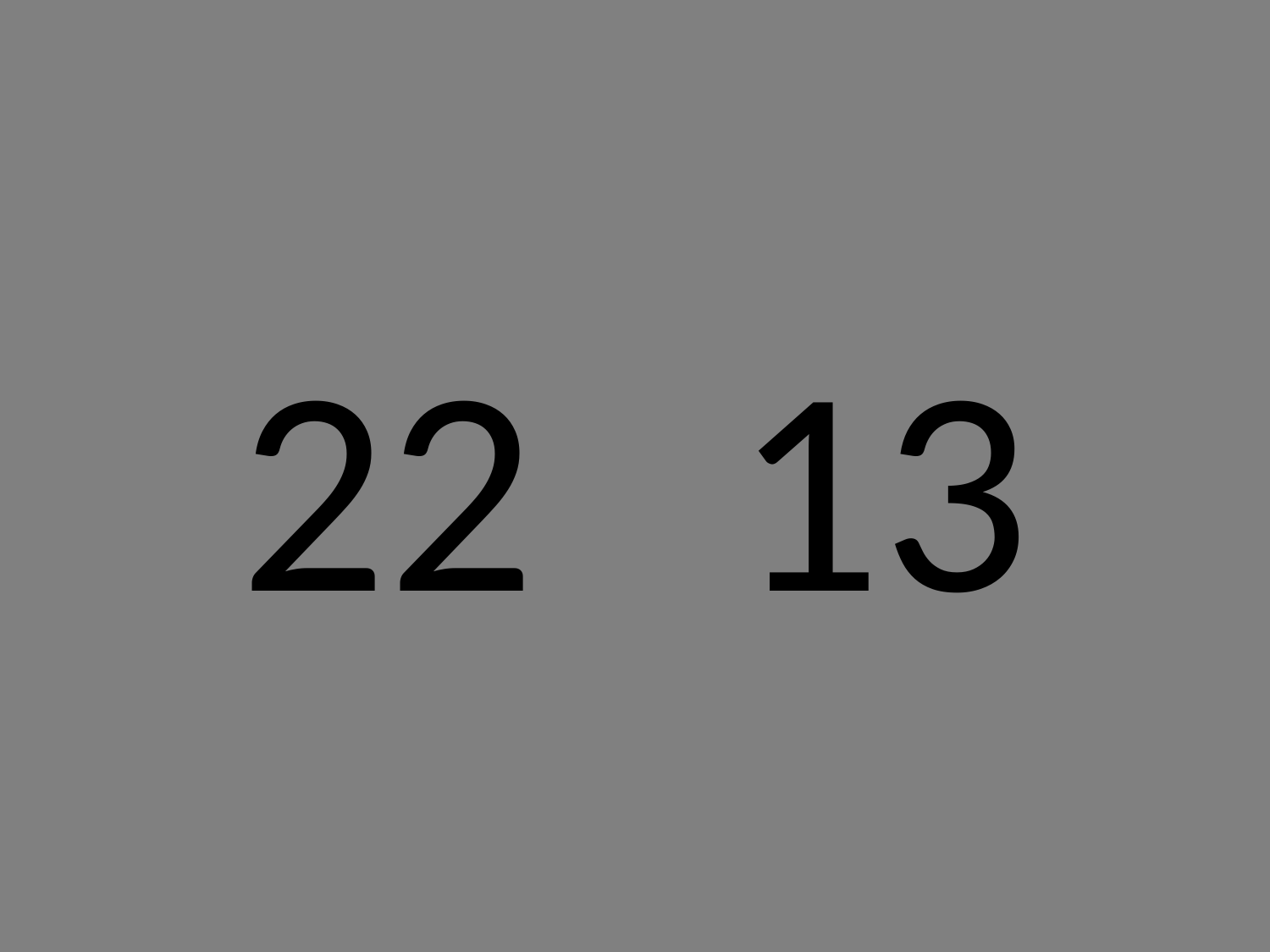

22 13

## Slide 30
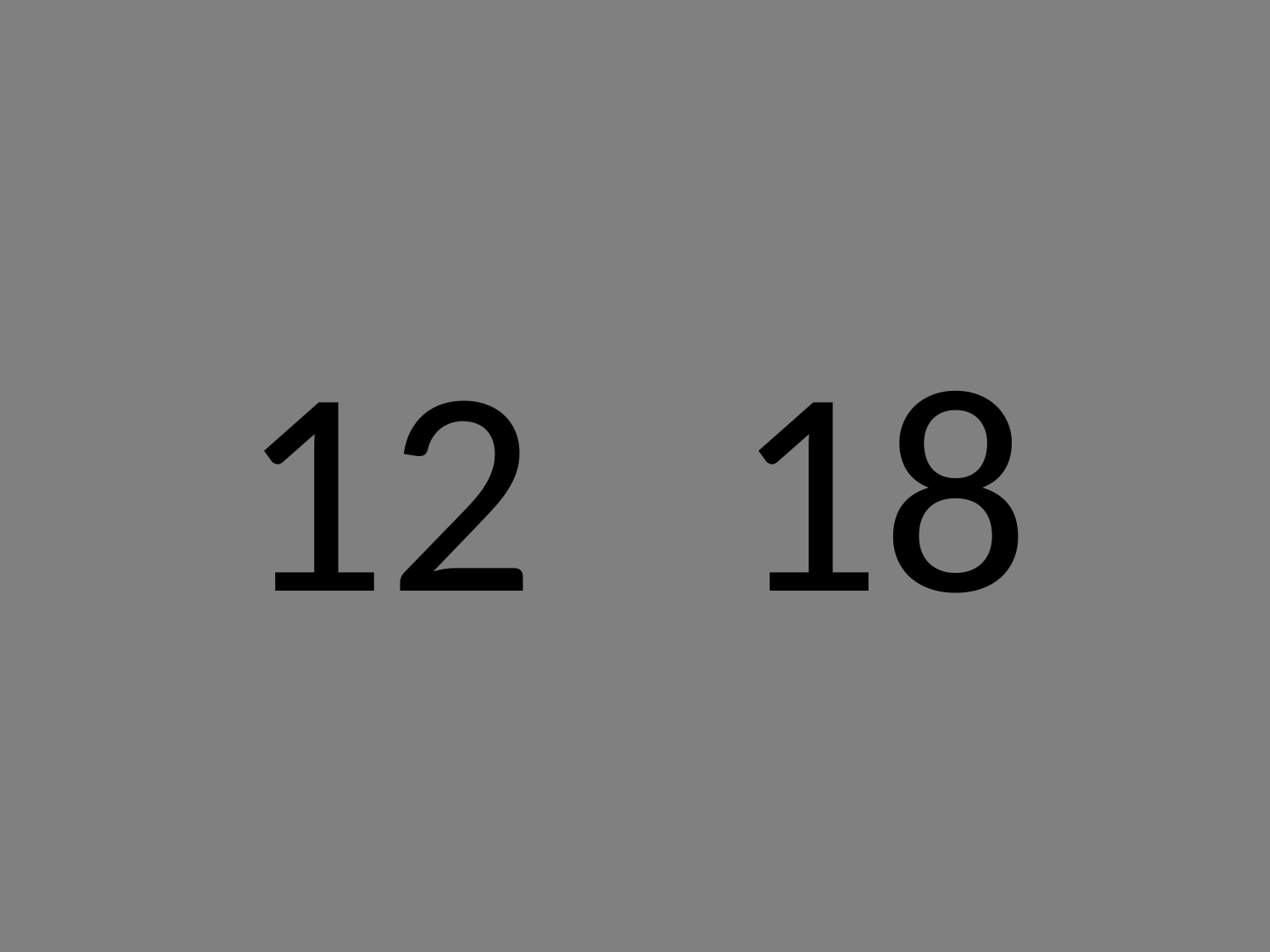

12 18

## Slide 31
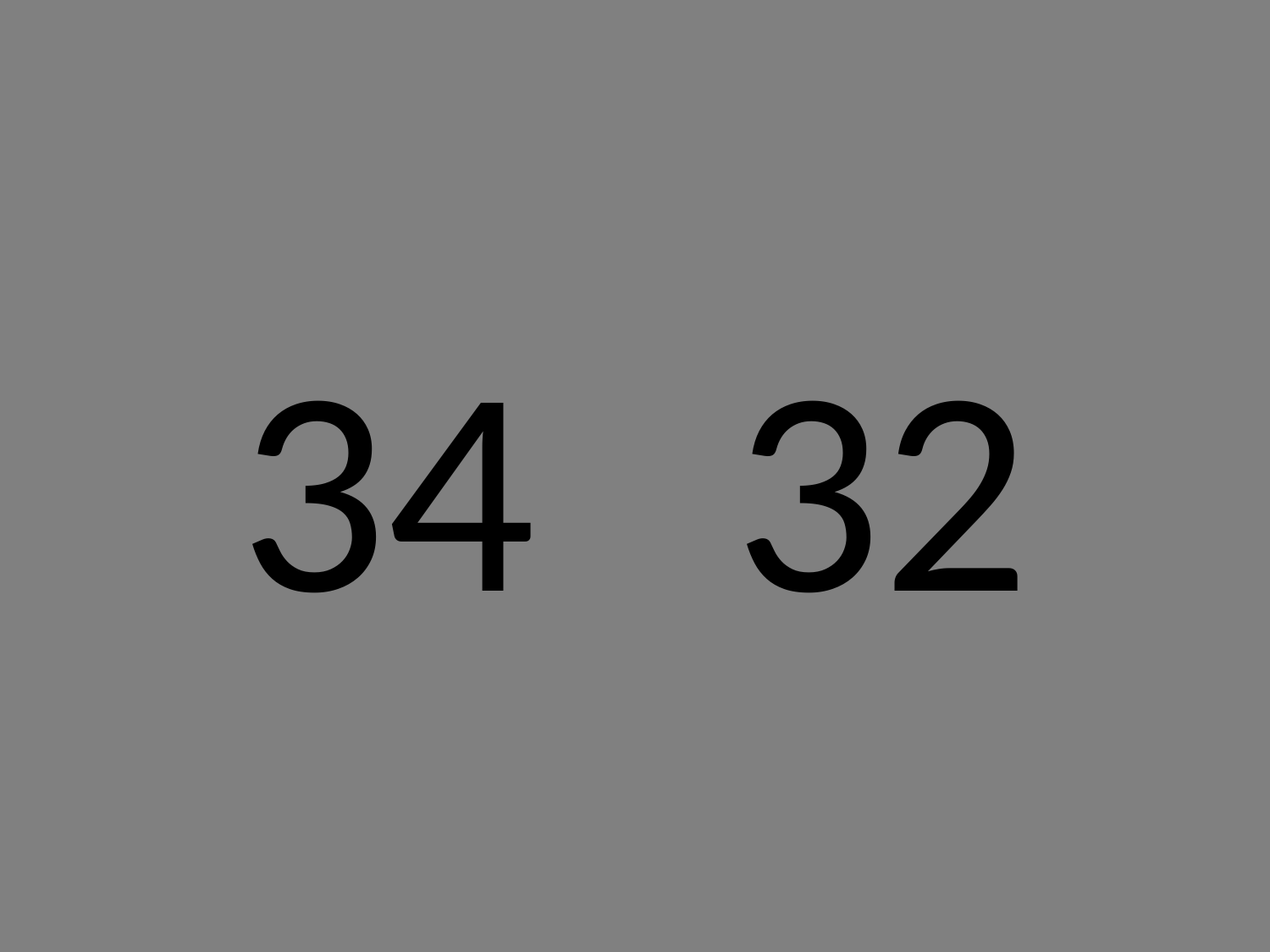

34 32

## Slide 32
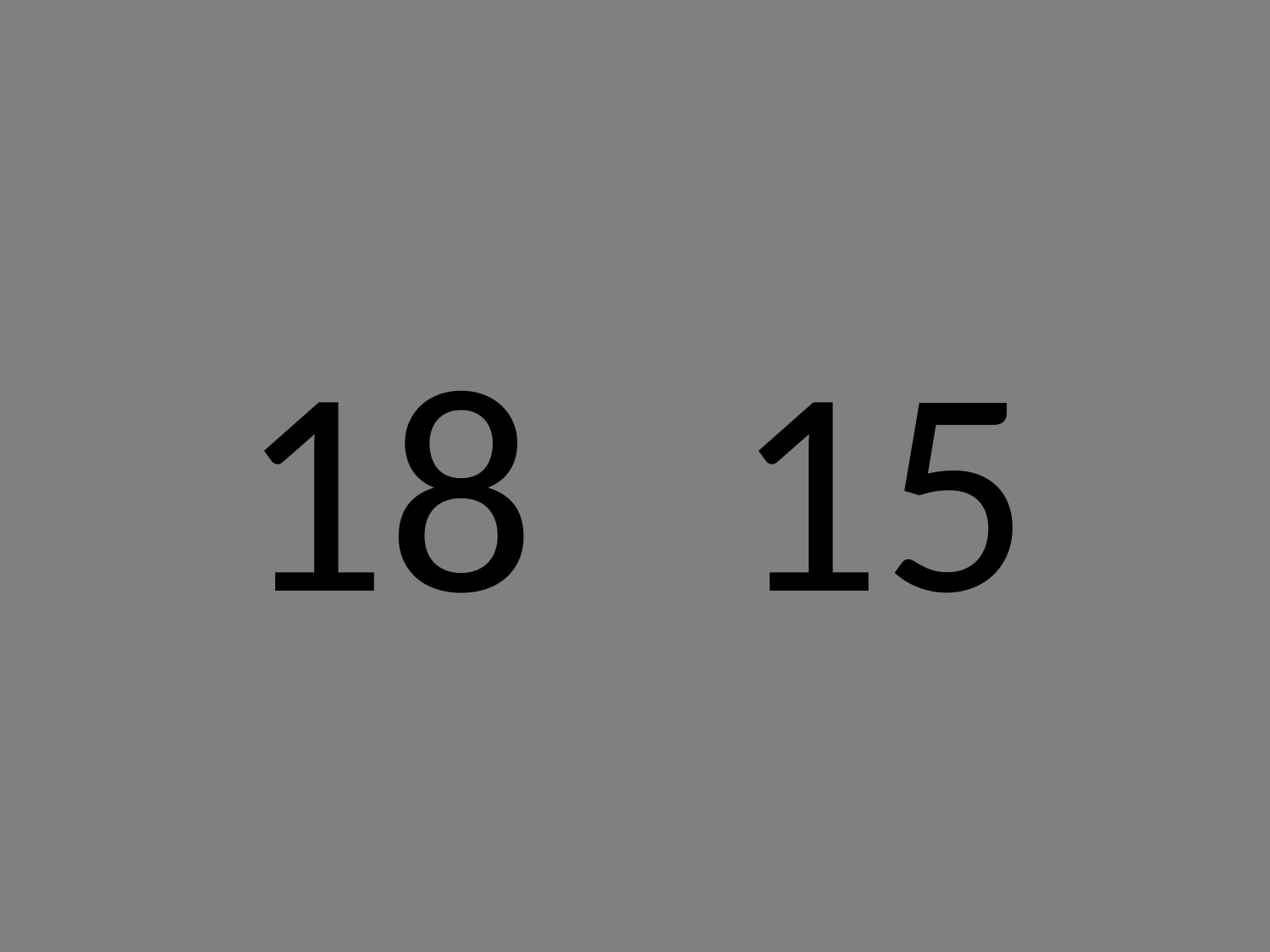

18 15

## Slide 33
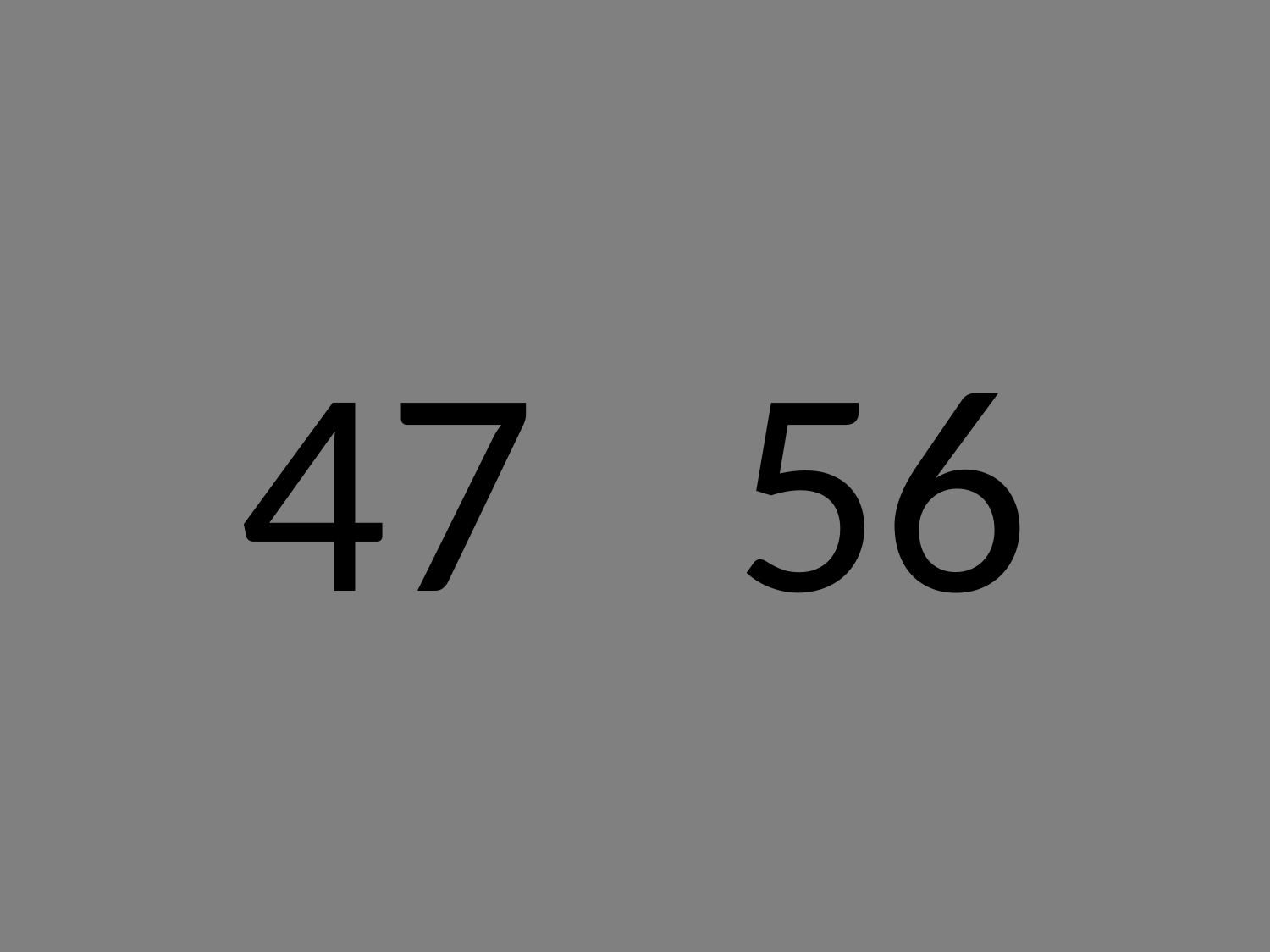

47 56

## Slide 34
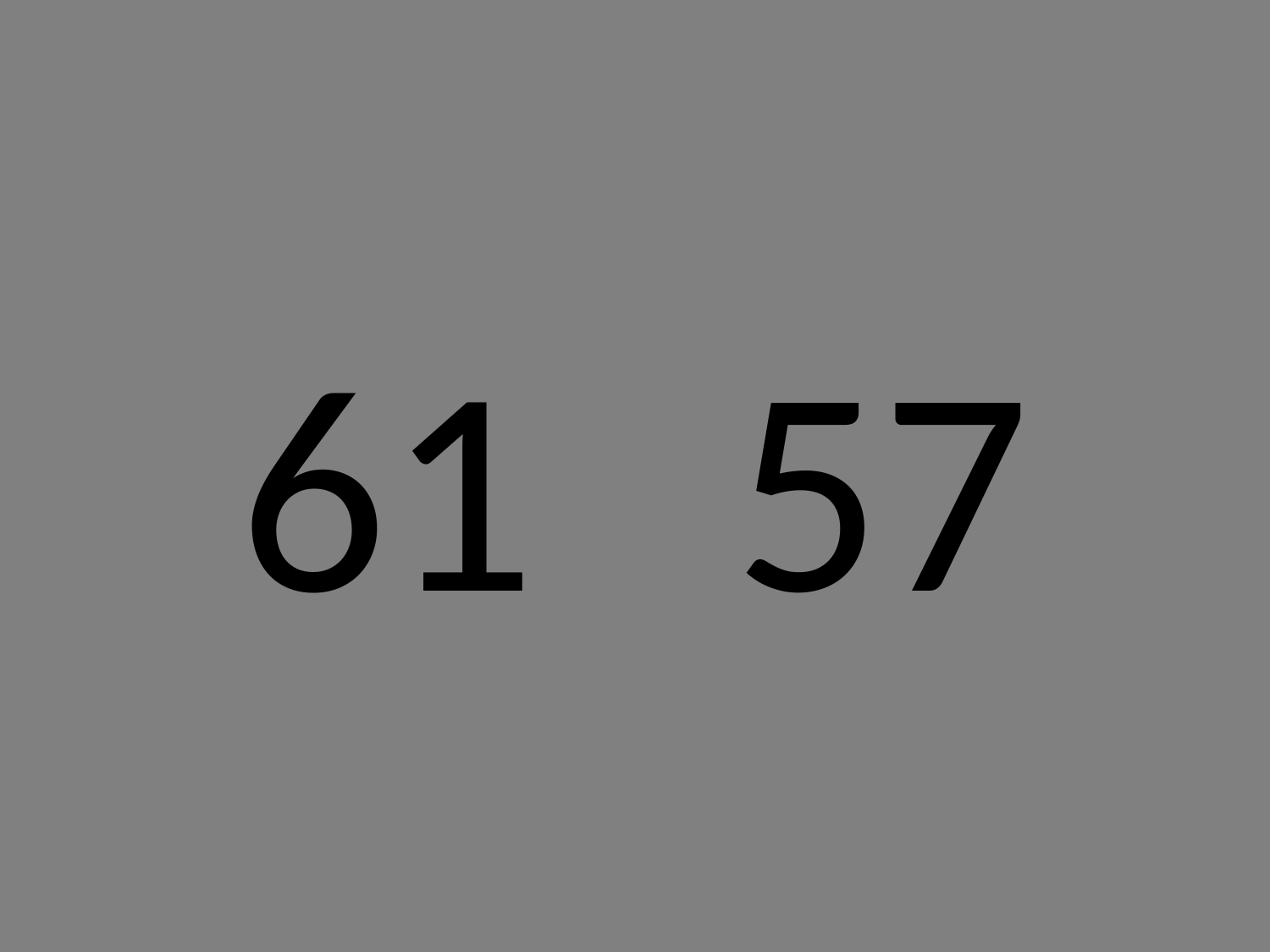

61 57

## Slide 35
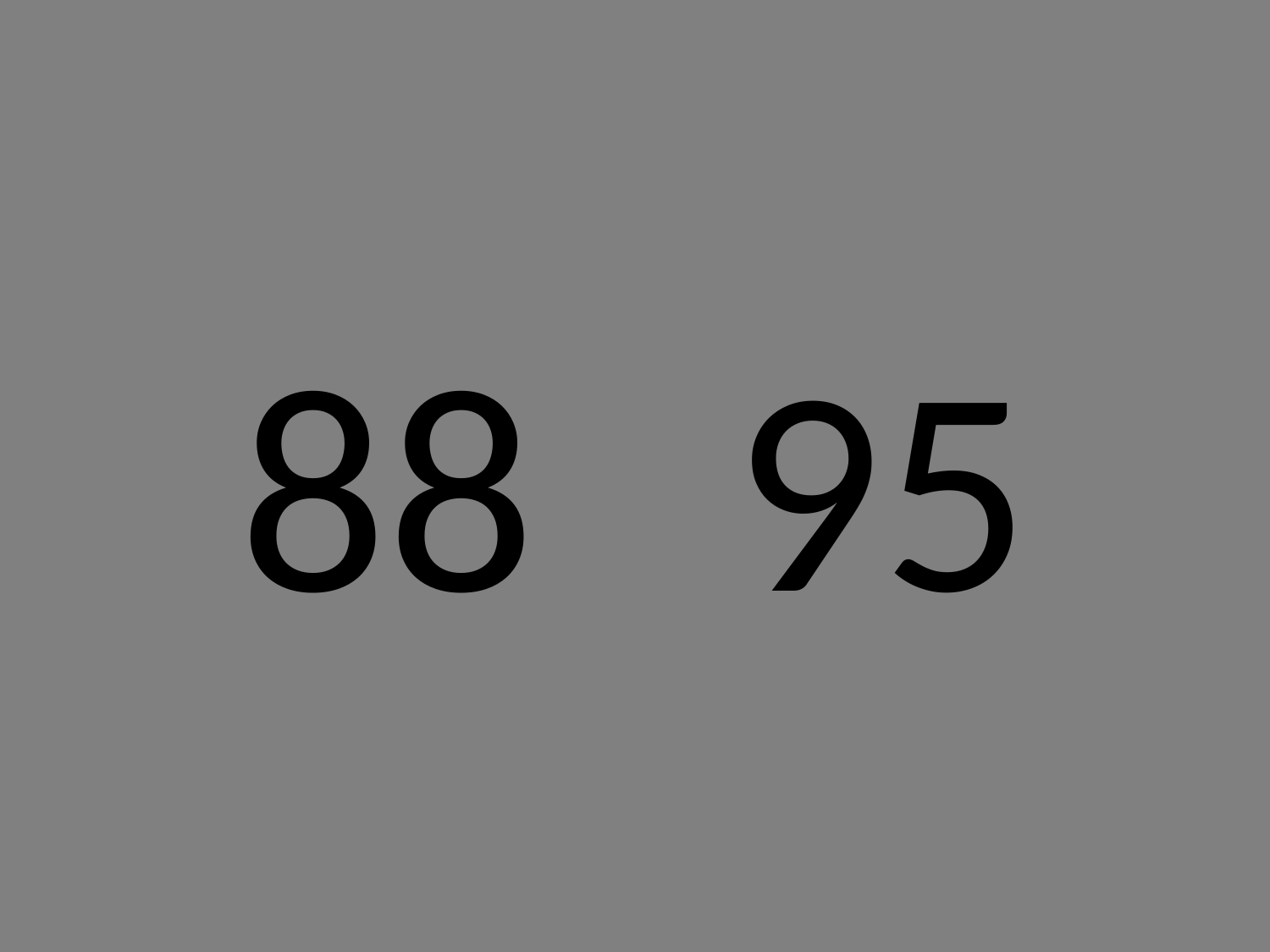

88 95

## Slide 36
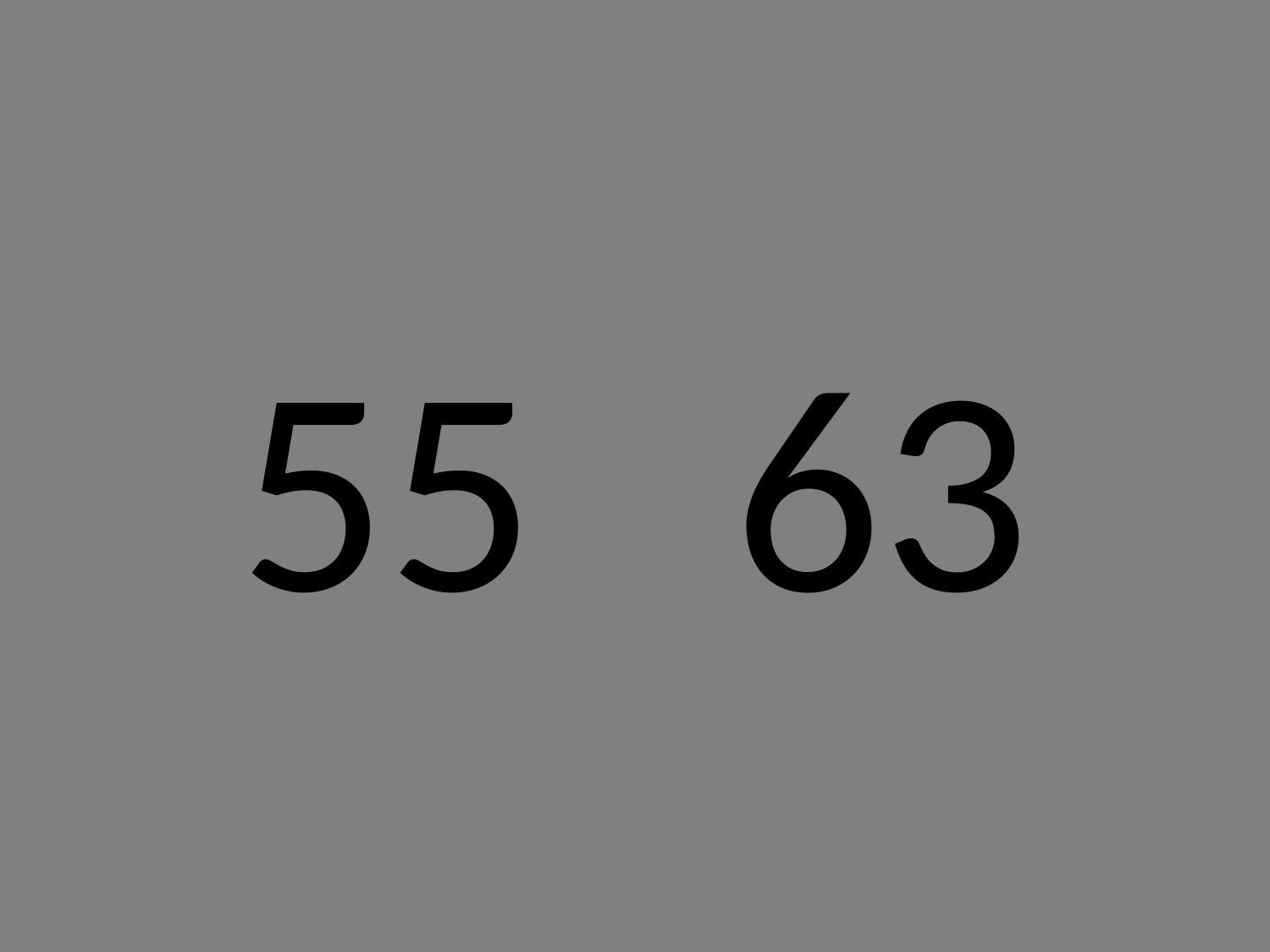

55 63

## Slide 37
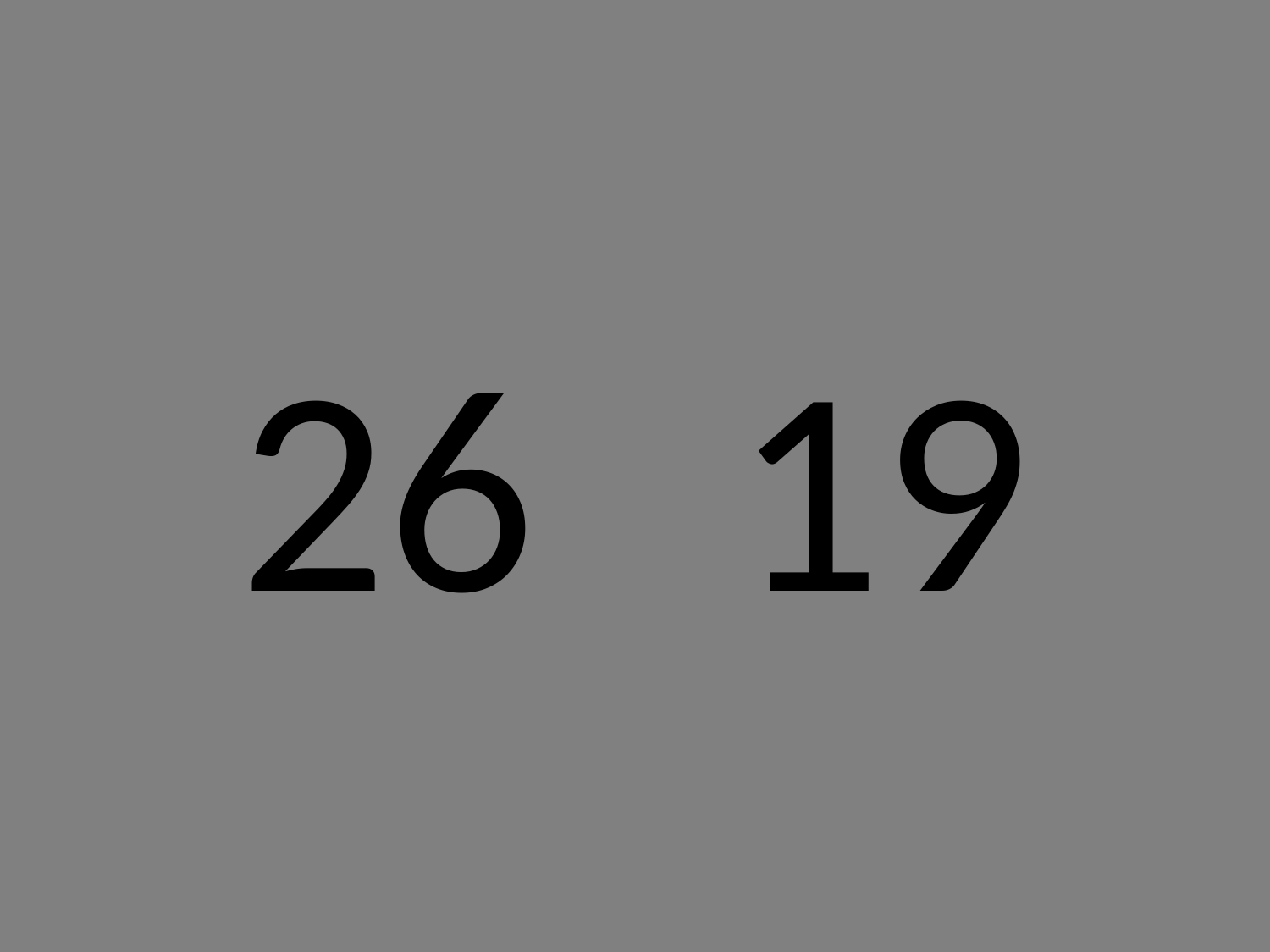

26 19

## Slide 38
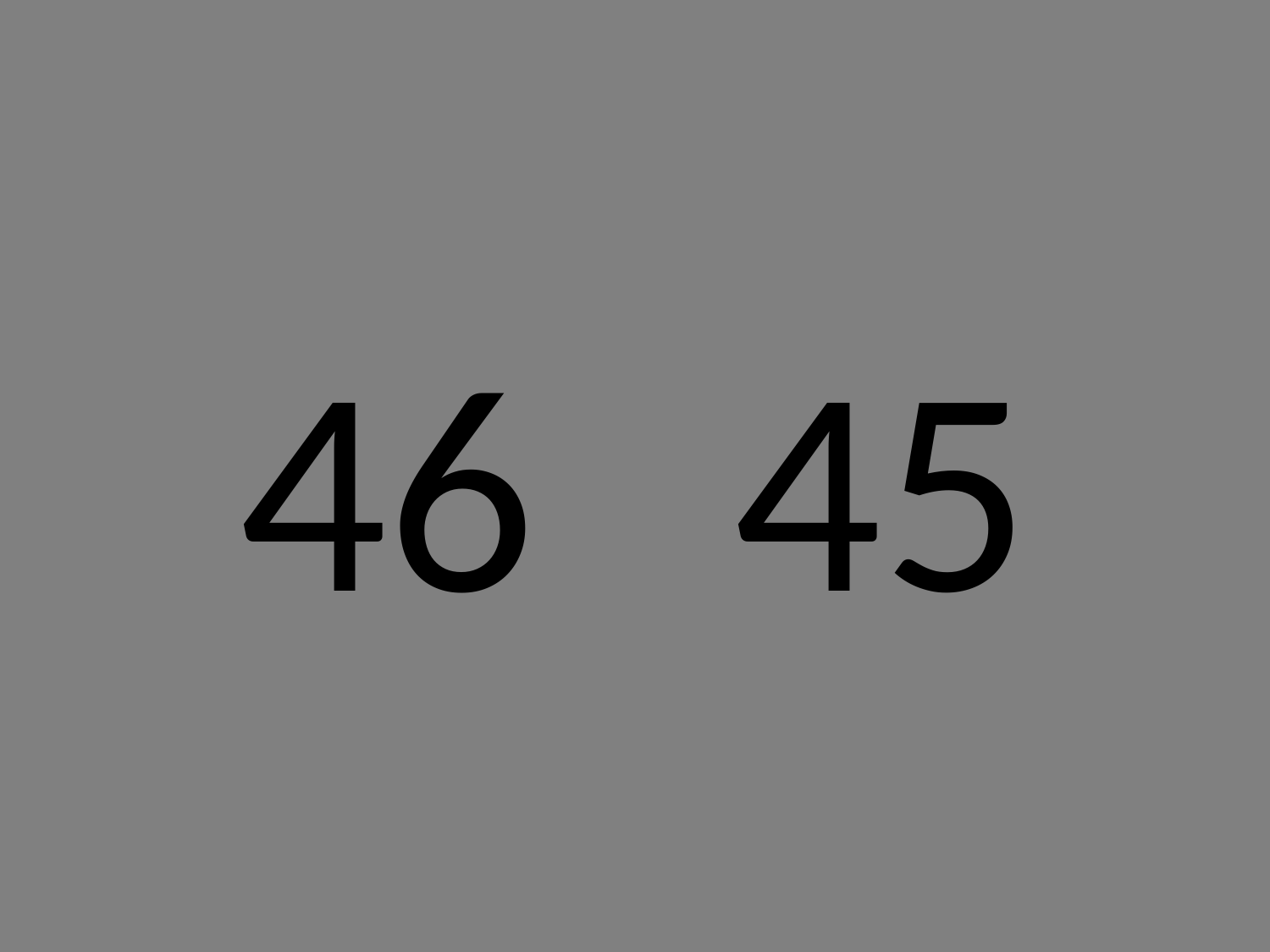

46 45

## Slide 39
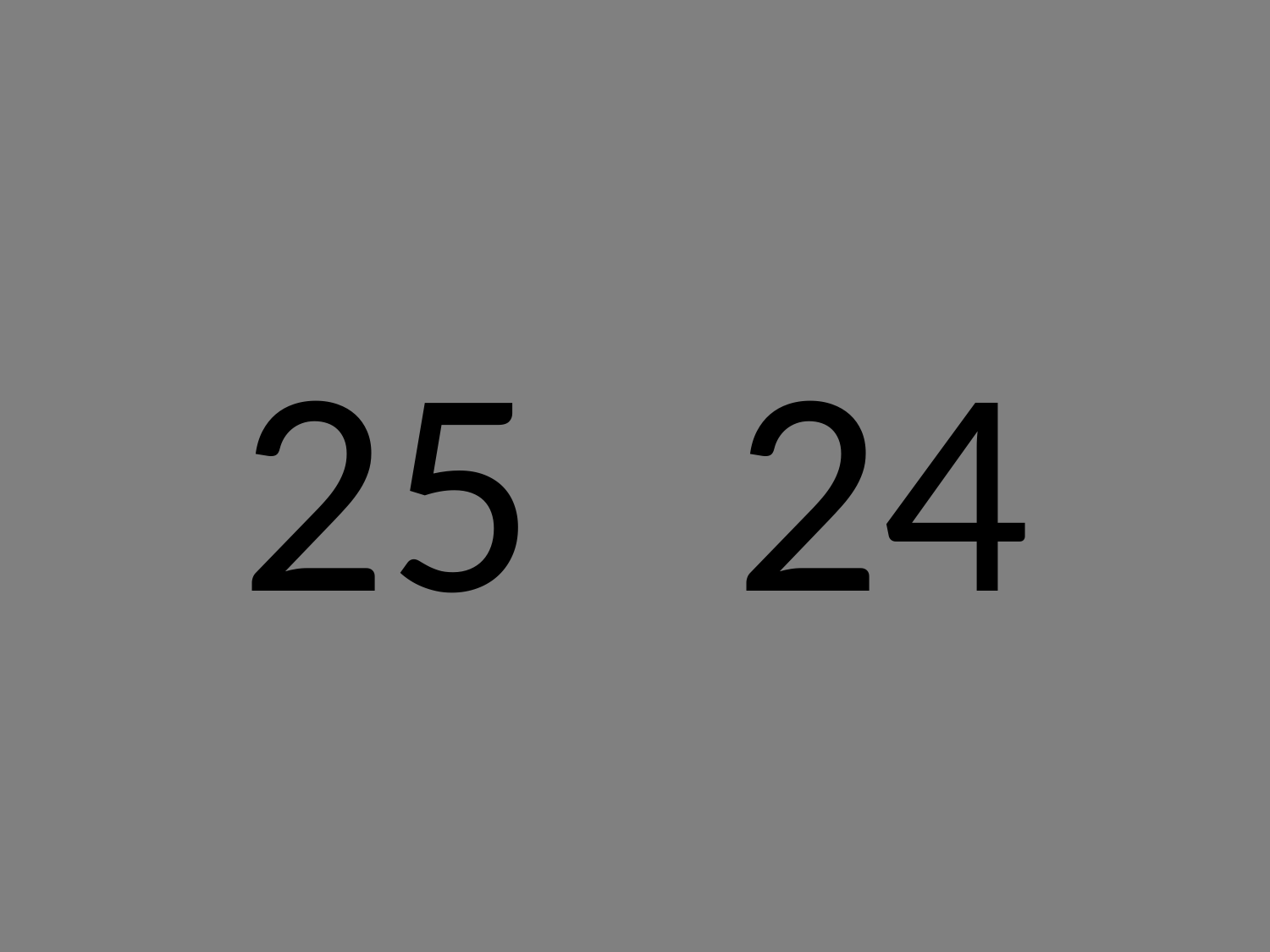

25 24

## Slide 40
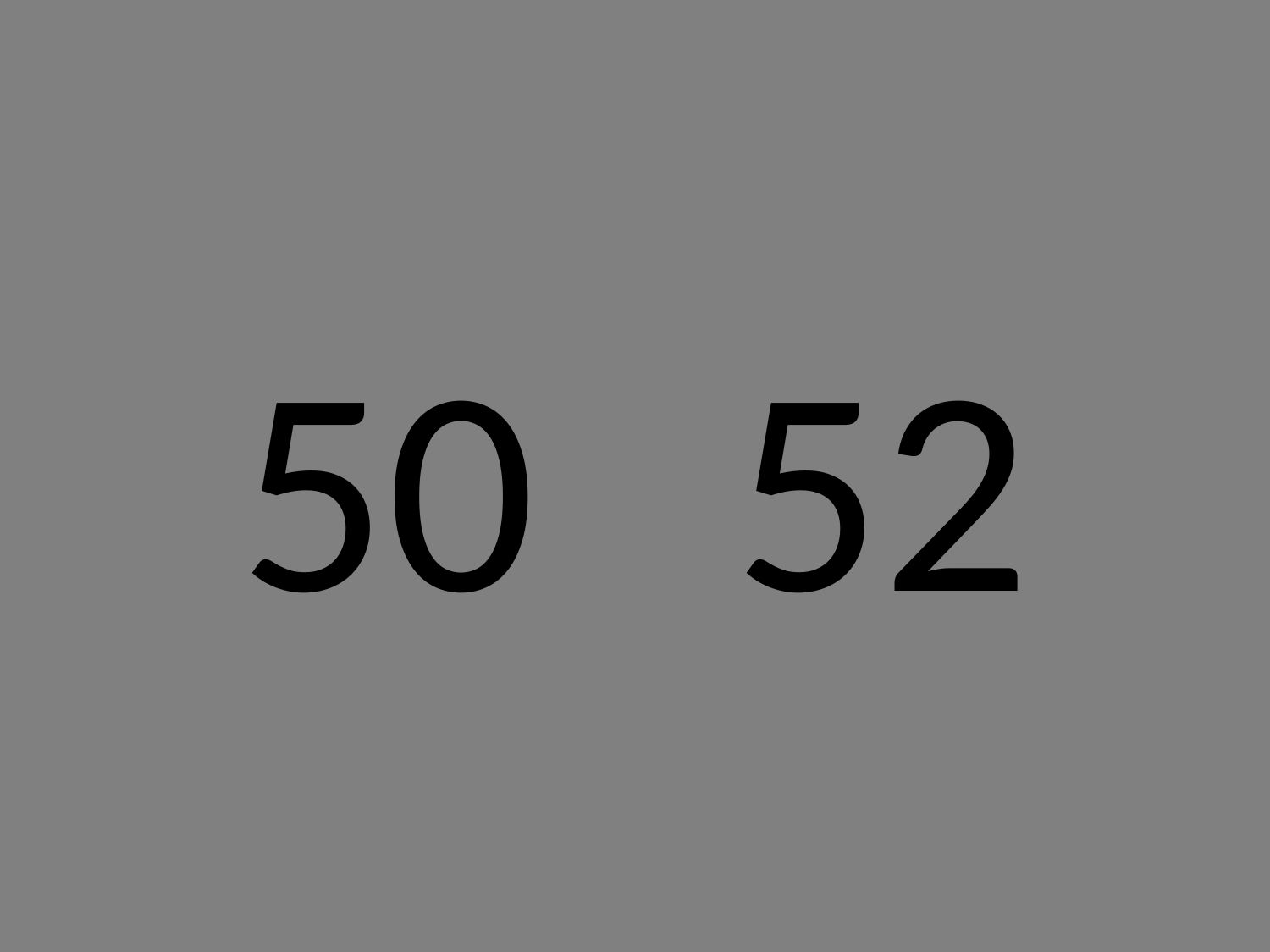

50 52

## Slide 41
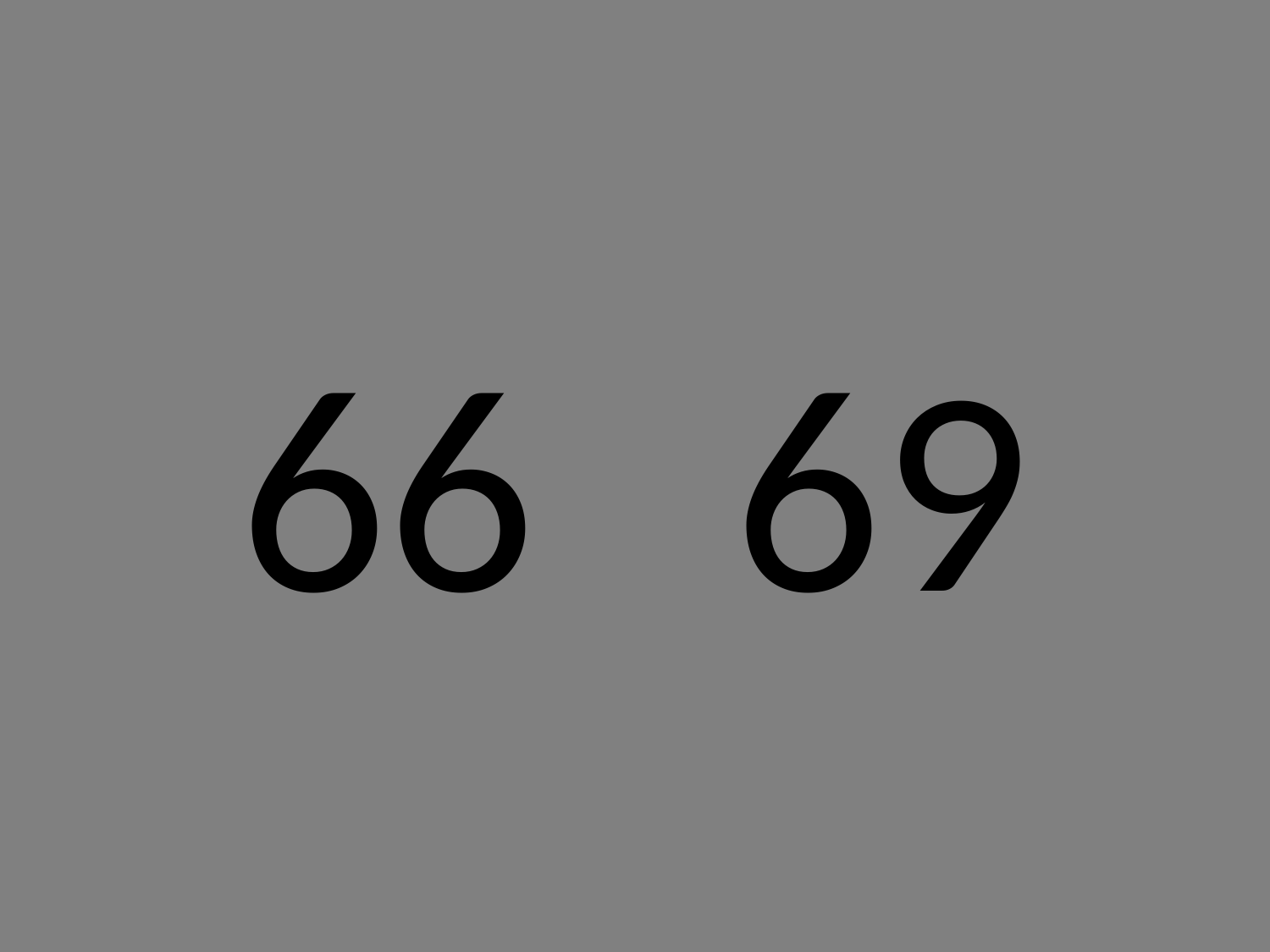

66 69

## Slide 42
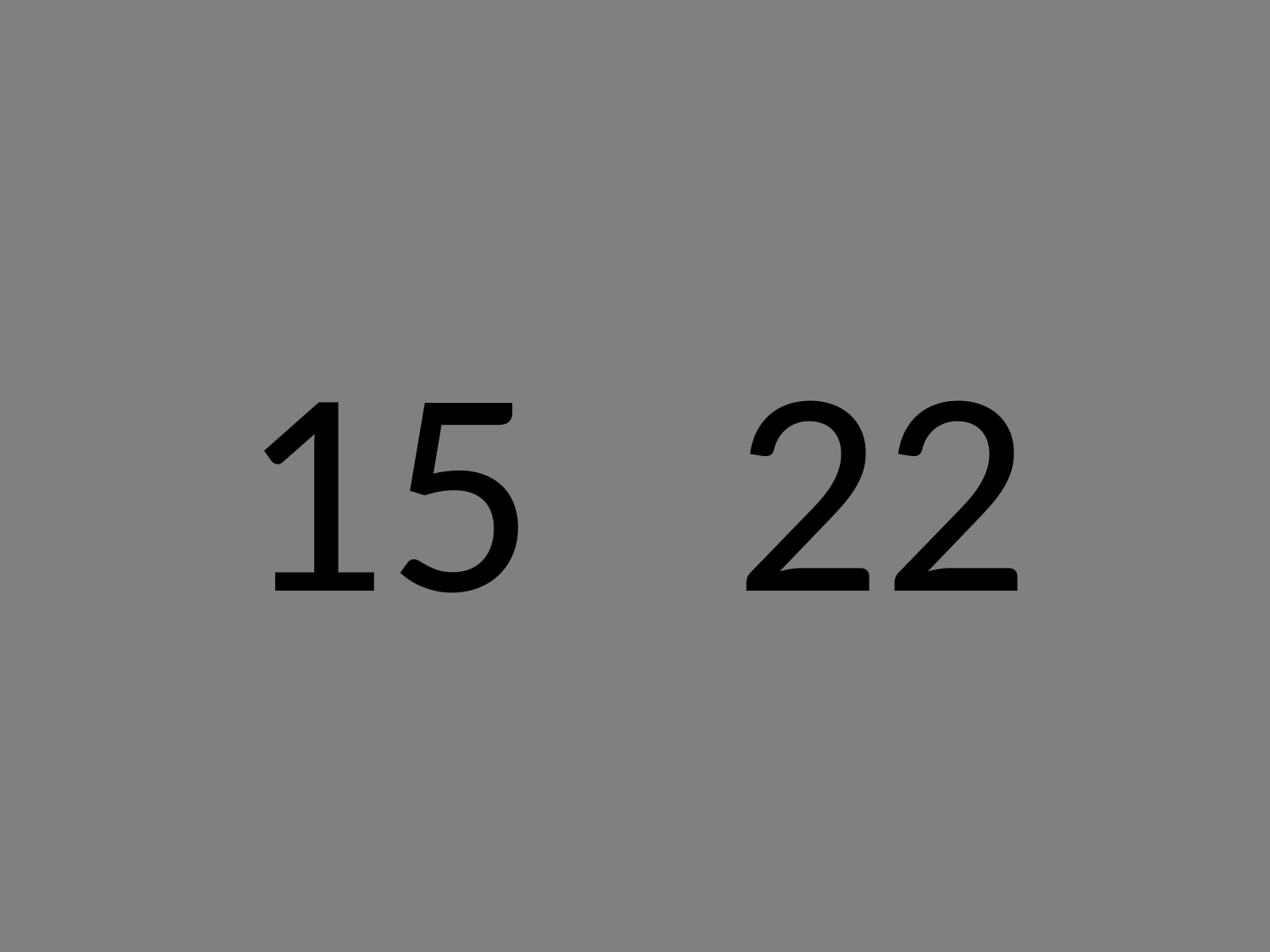

15 22

## Slide 43
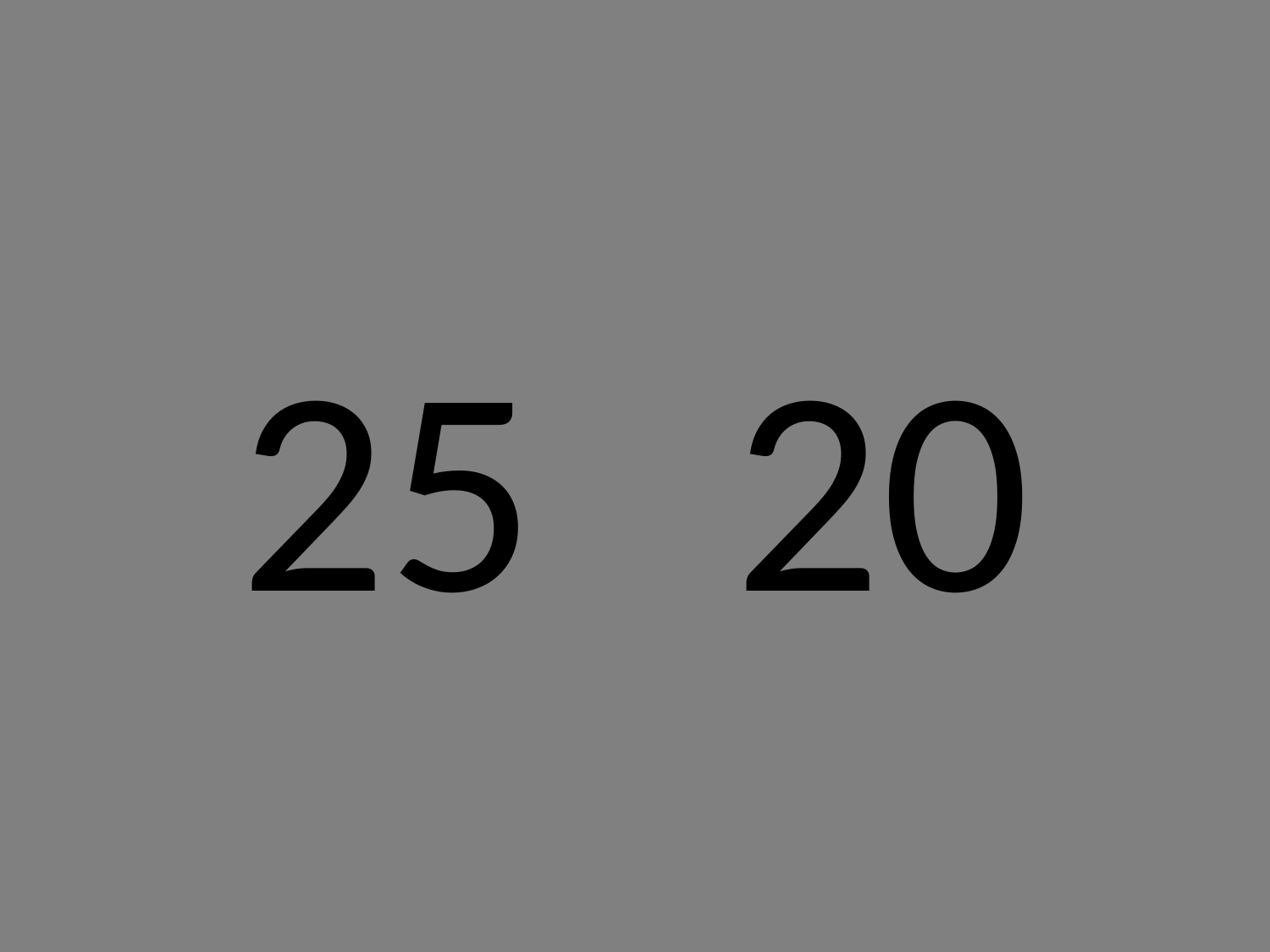

25 20

## Slide 44
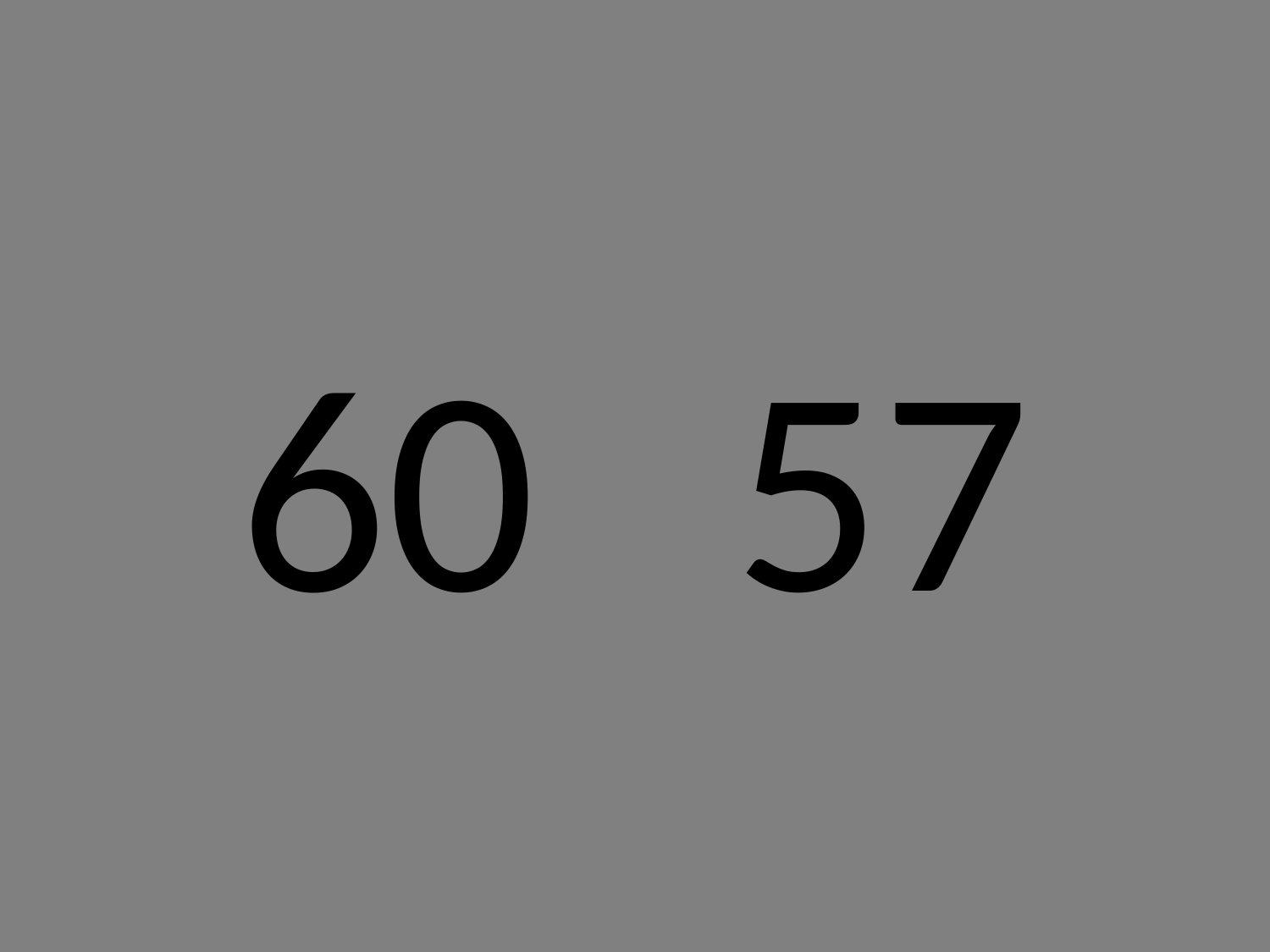

60 57

## Slide 45
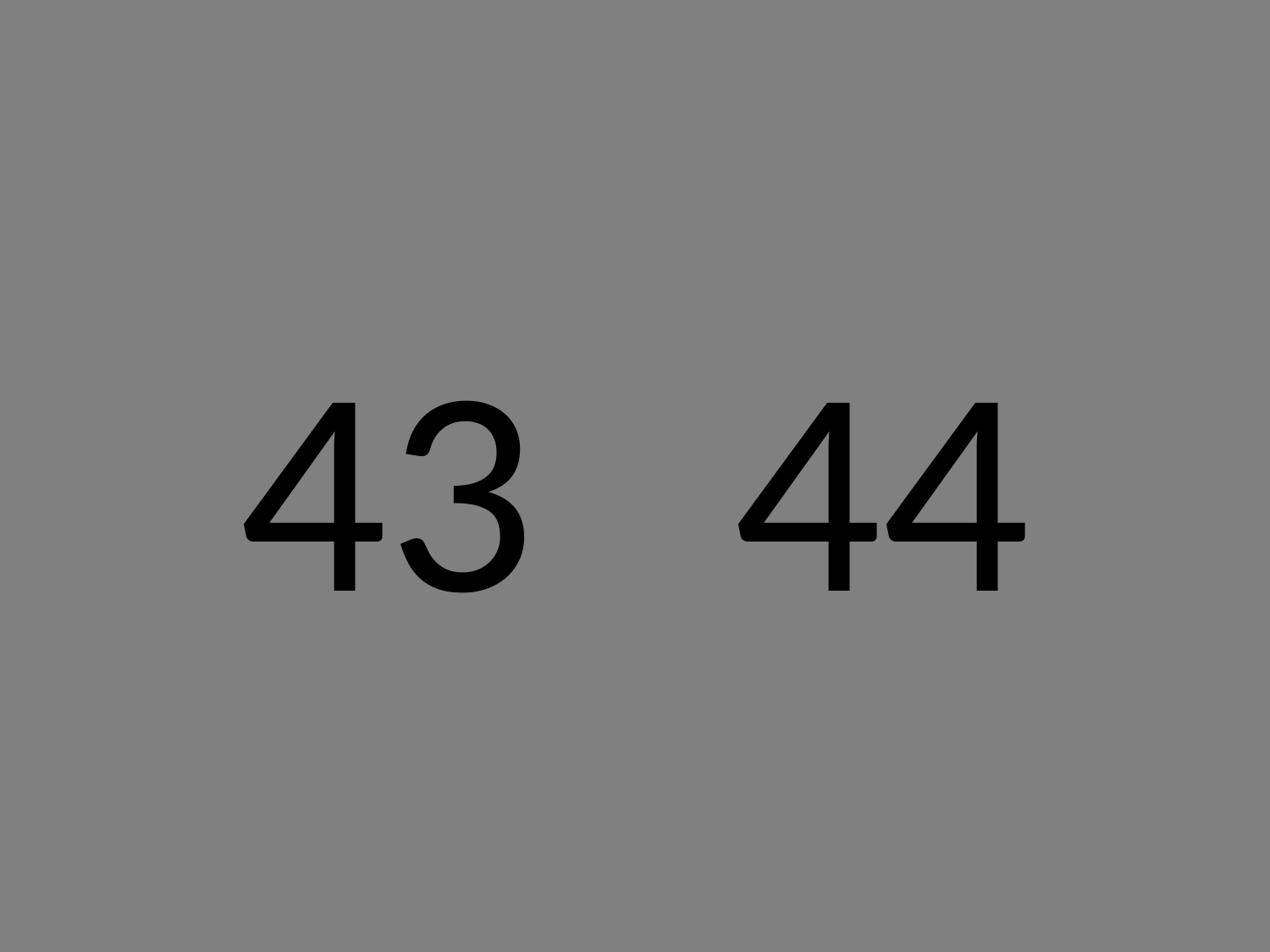

43 44

## Slide 46
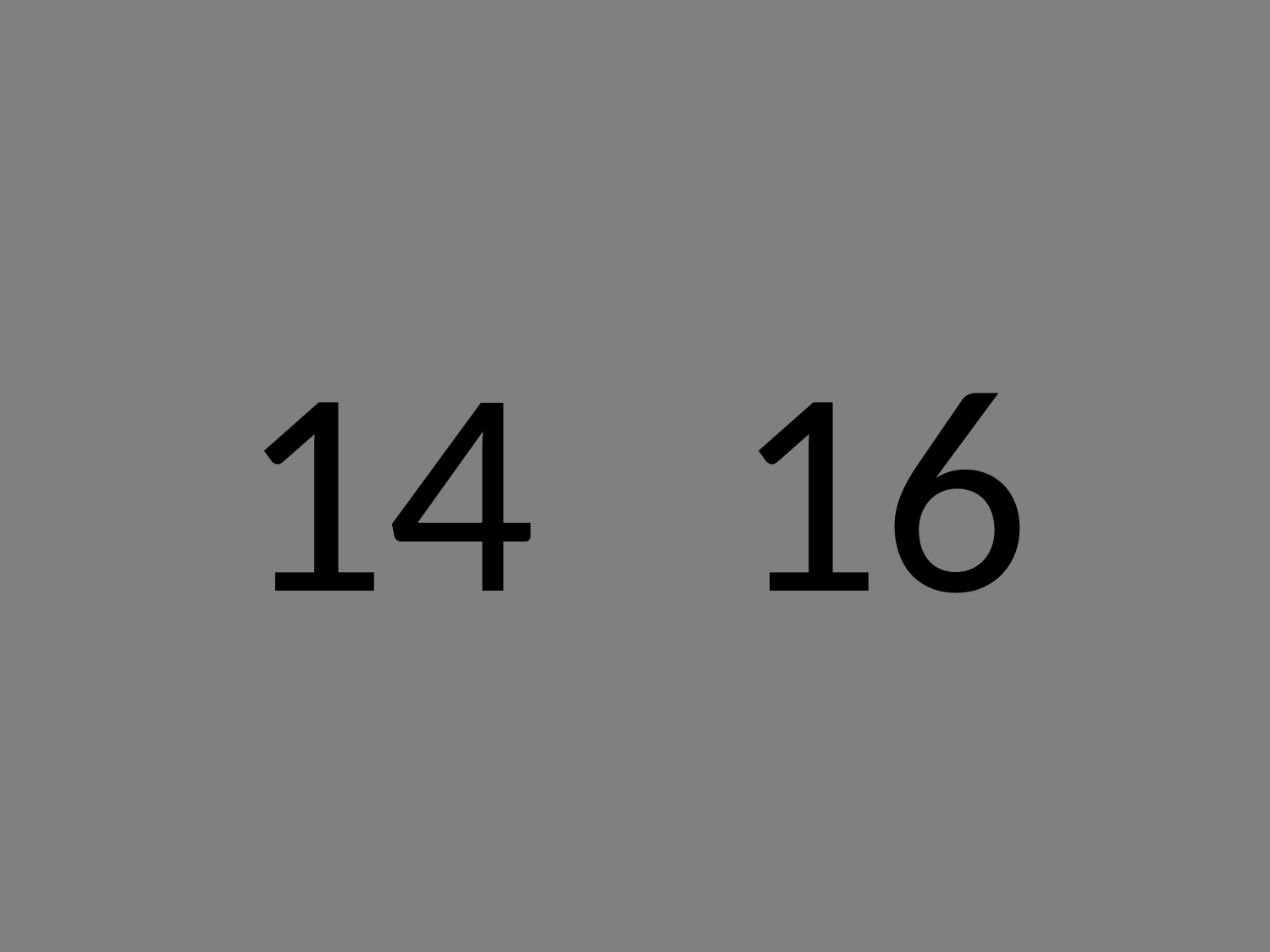

14 16

## Slide 47
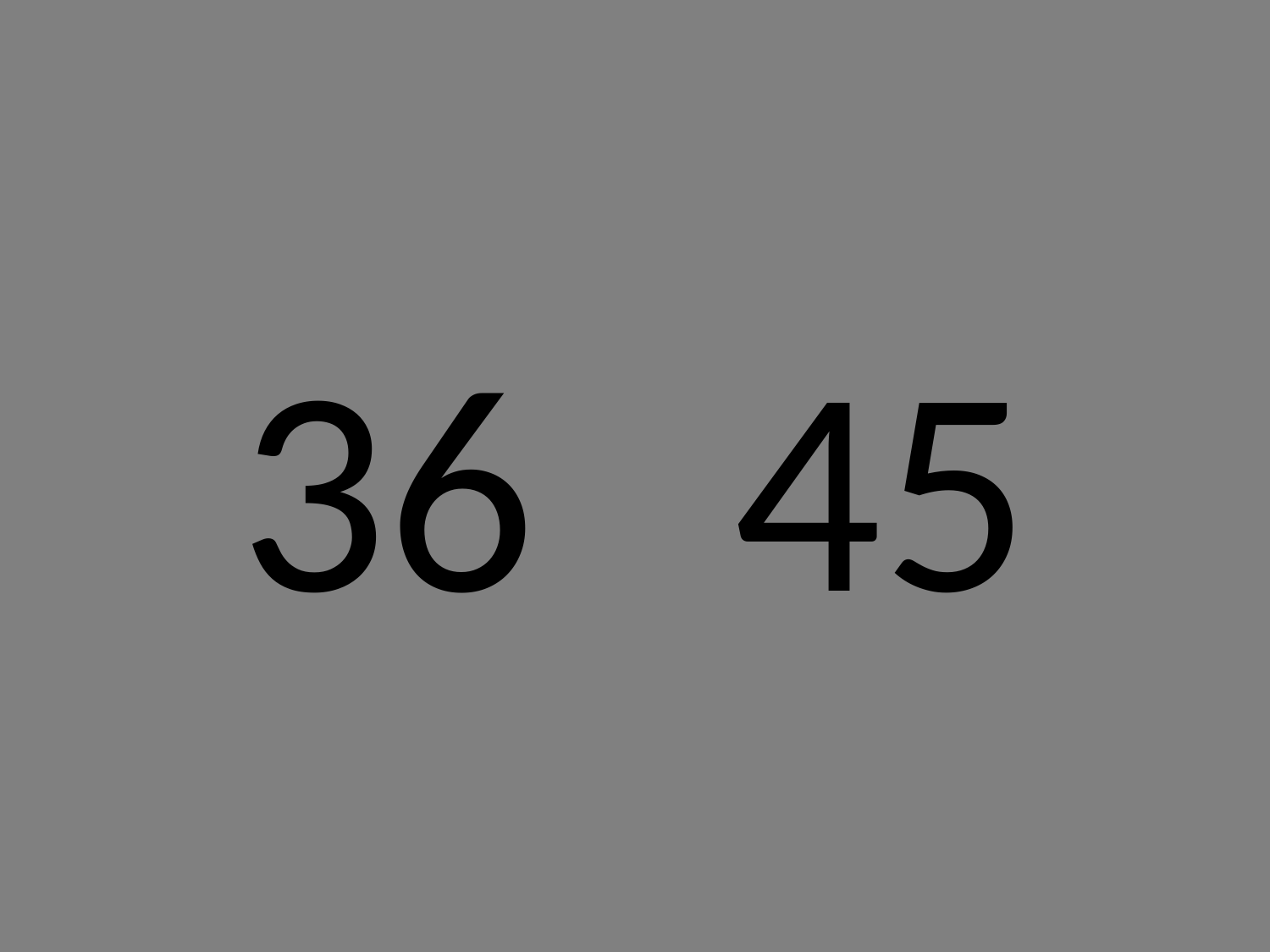

36 45

## Slide 48
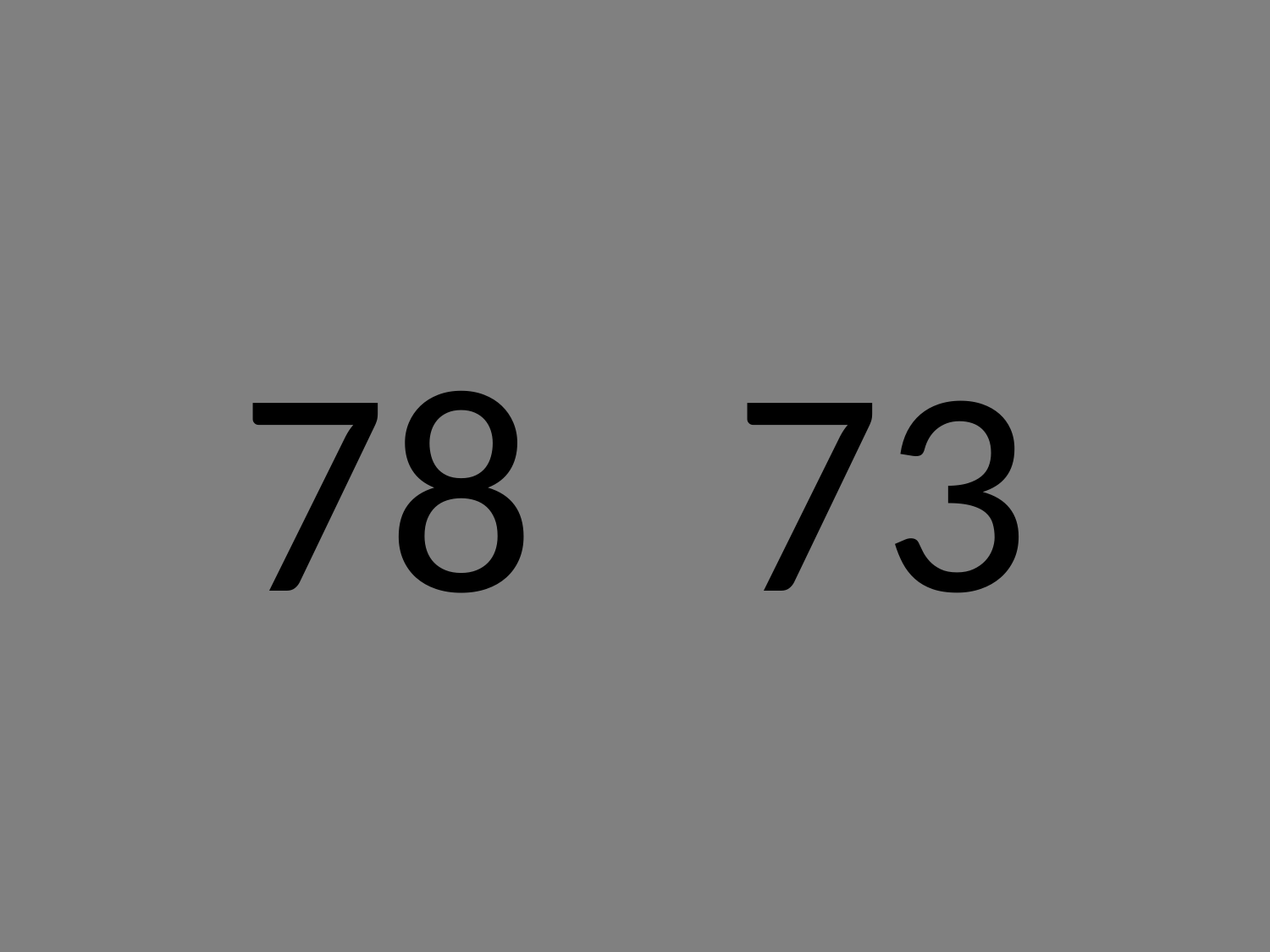

78 73

## Slide 49
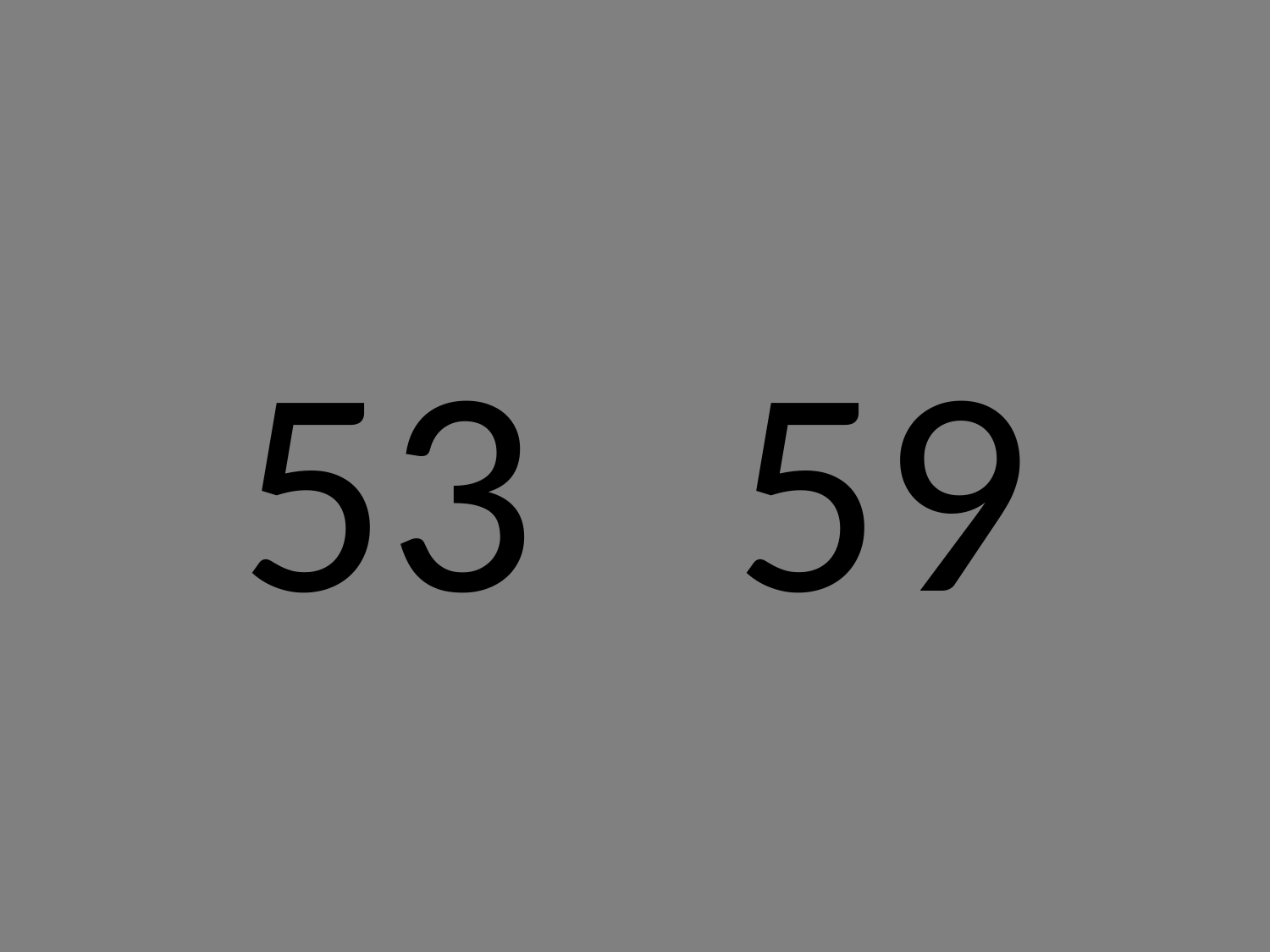

53 59

## Slide 50
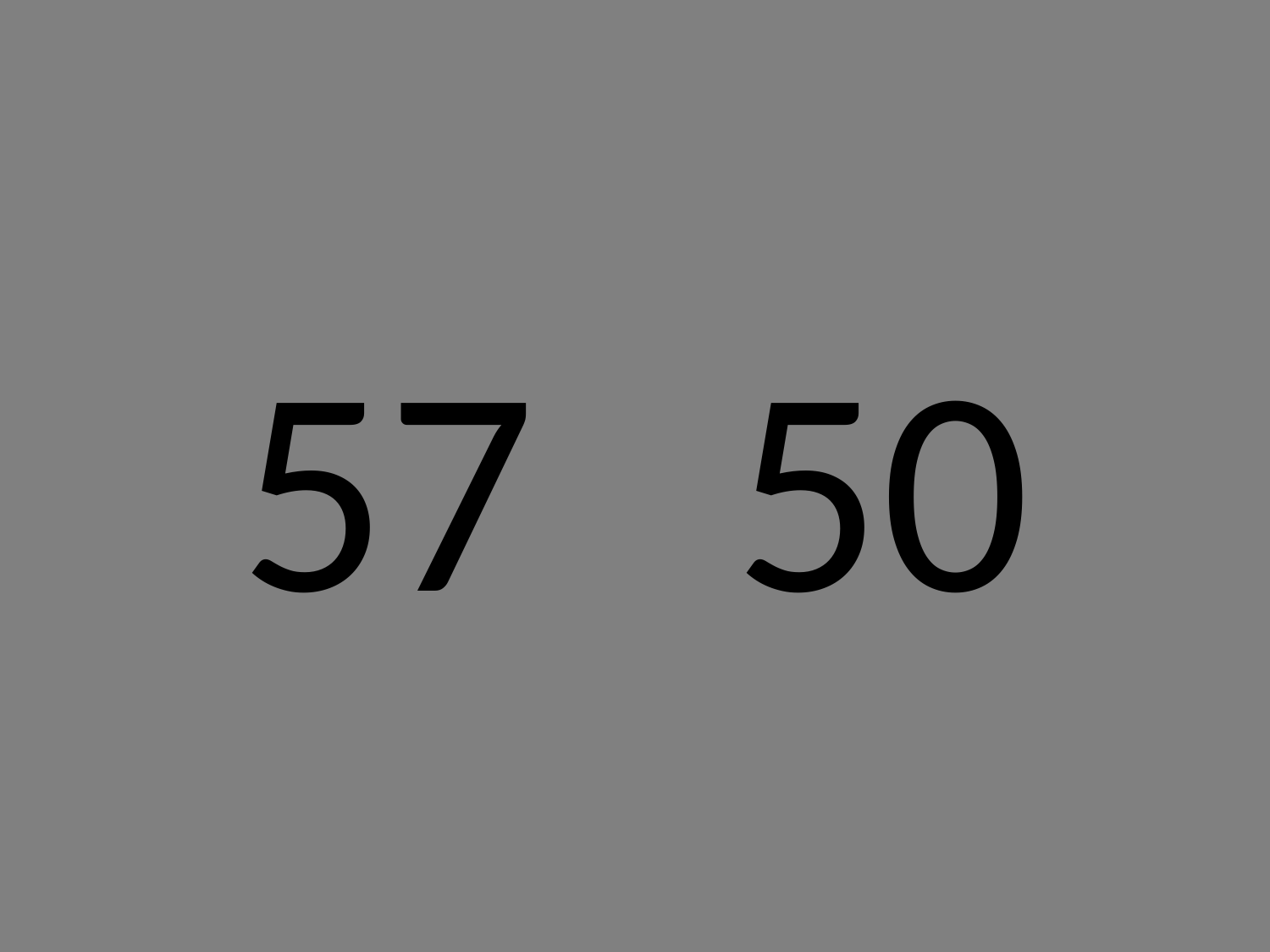

57 50

## Slide 51
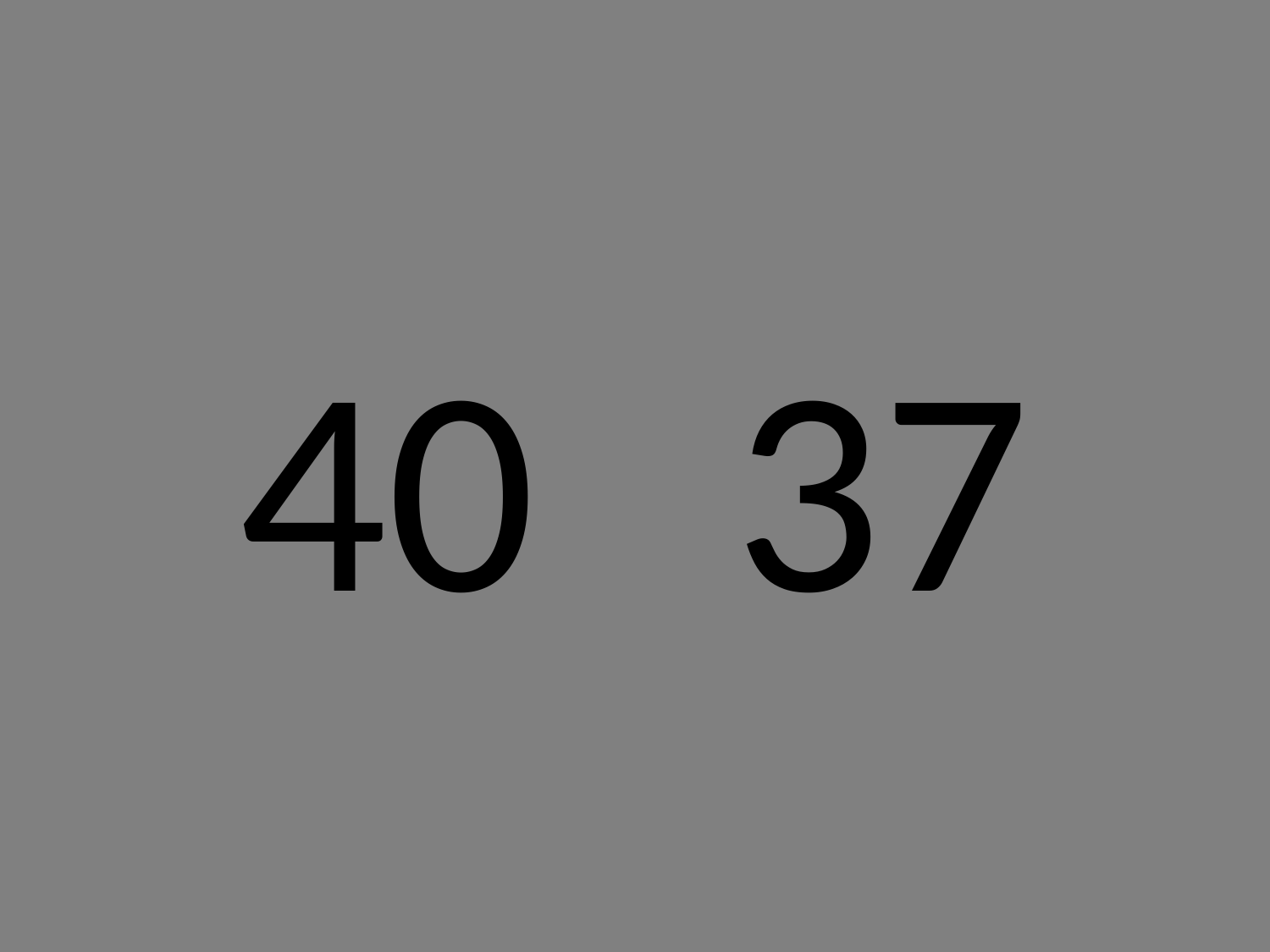

40 37

## Slide 52
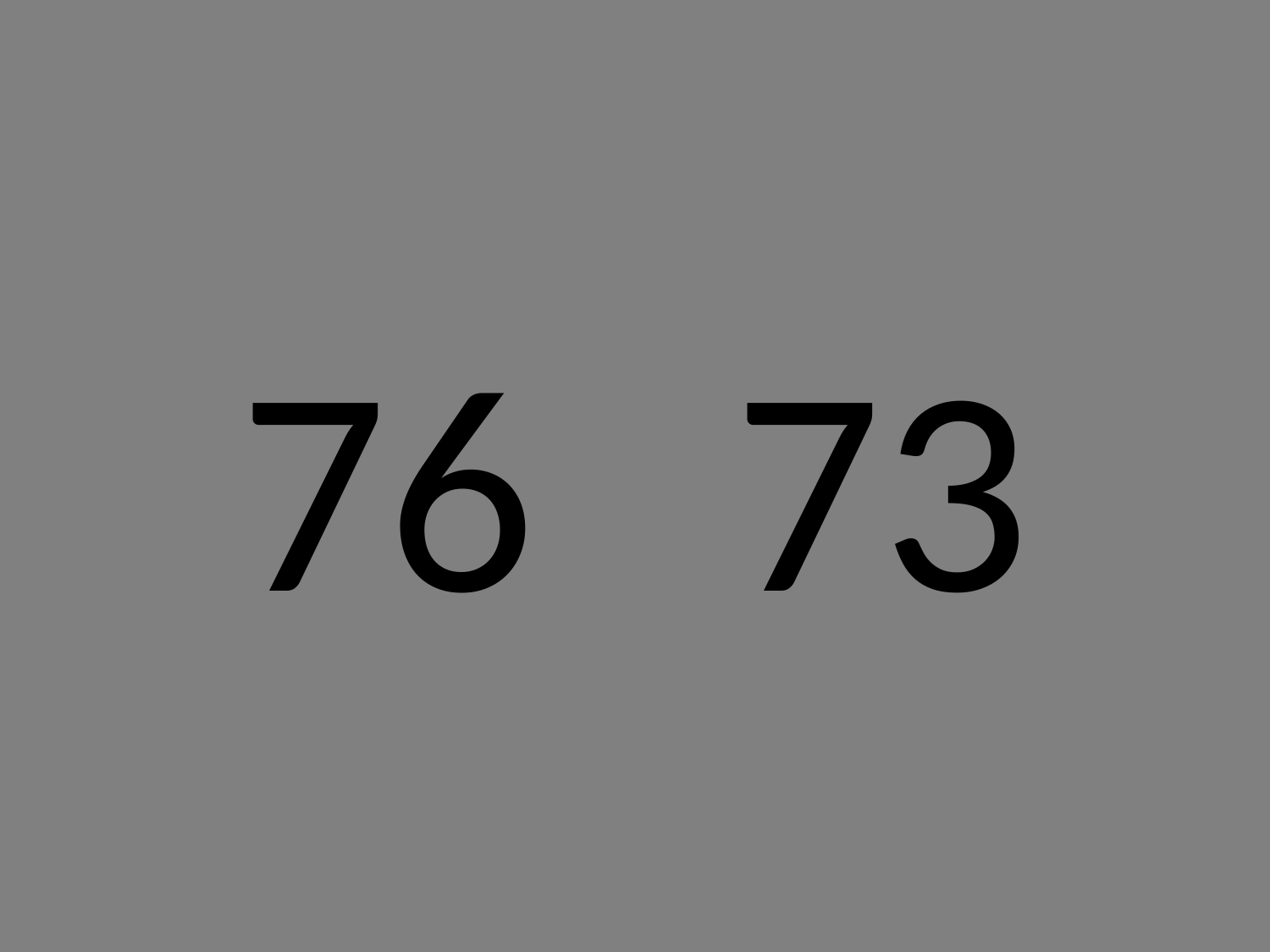

76 73

## Slide 53
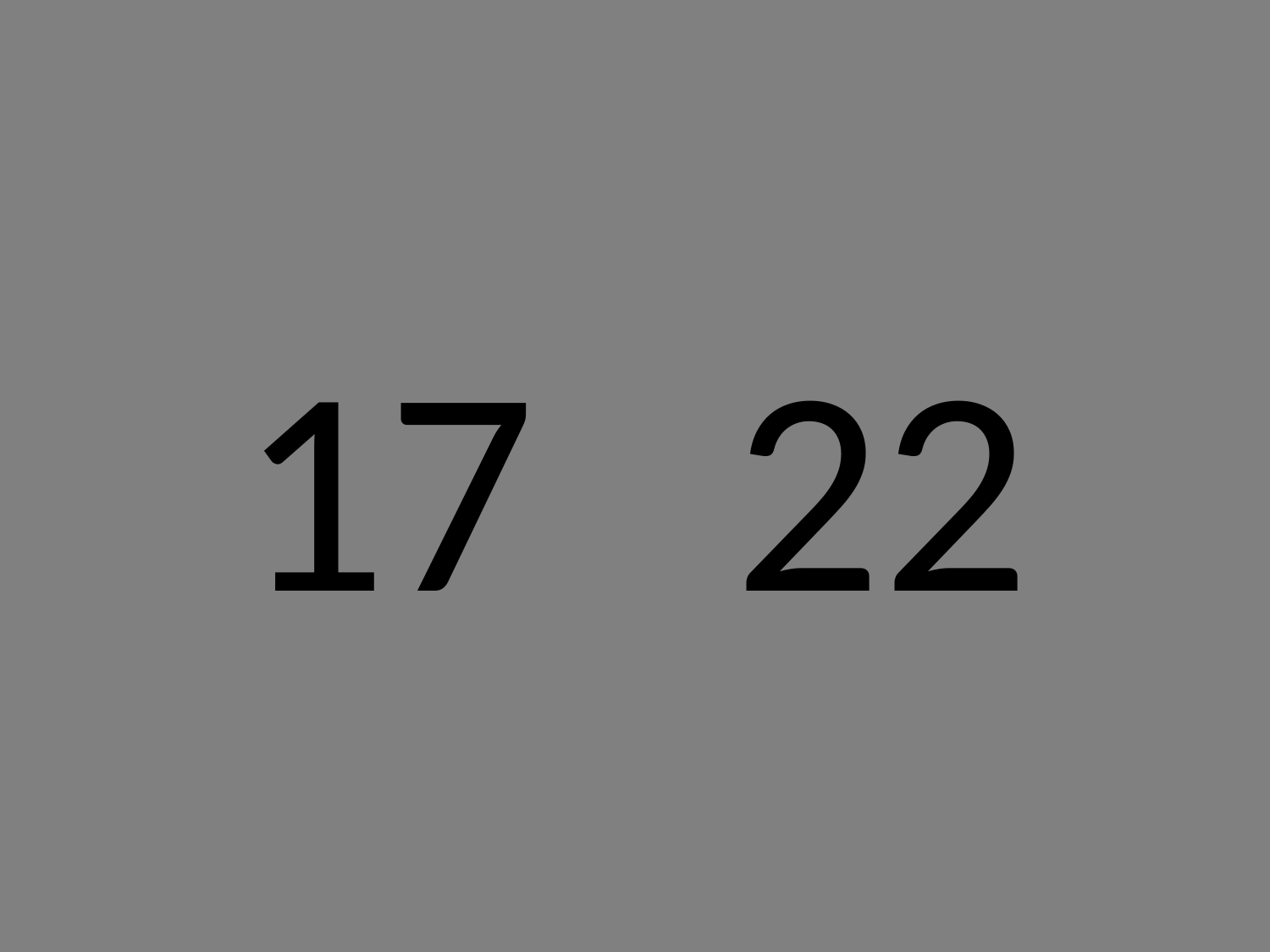

17 22

## Slide 54
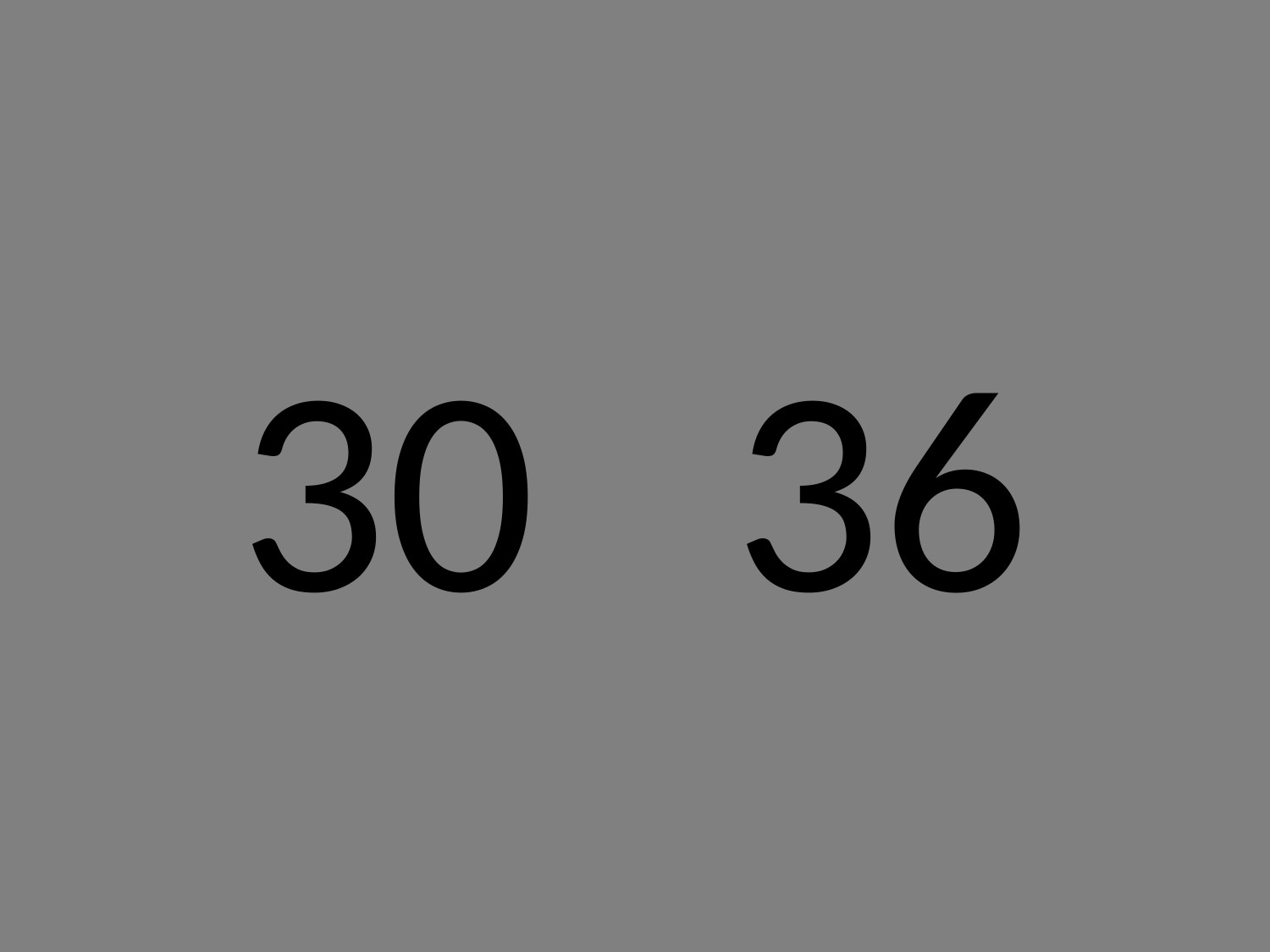

30 36

## Slide 55
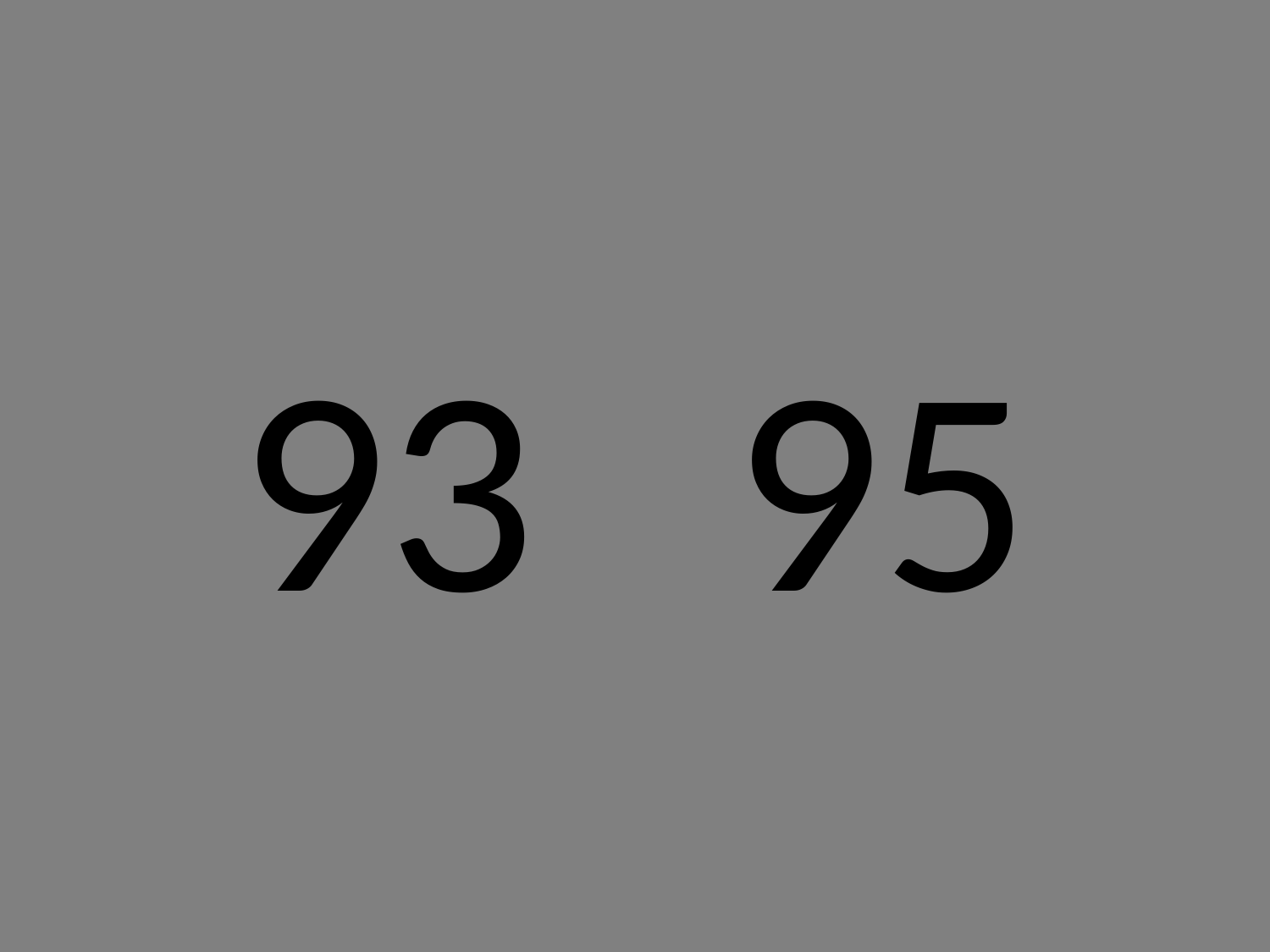

93 95

## Slide 56
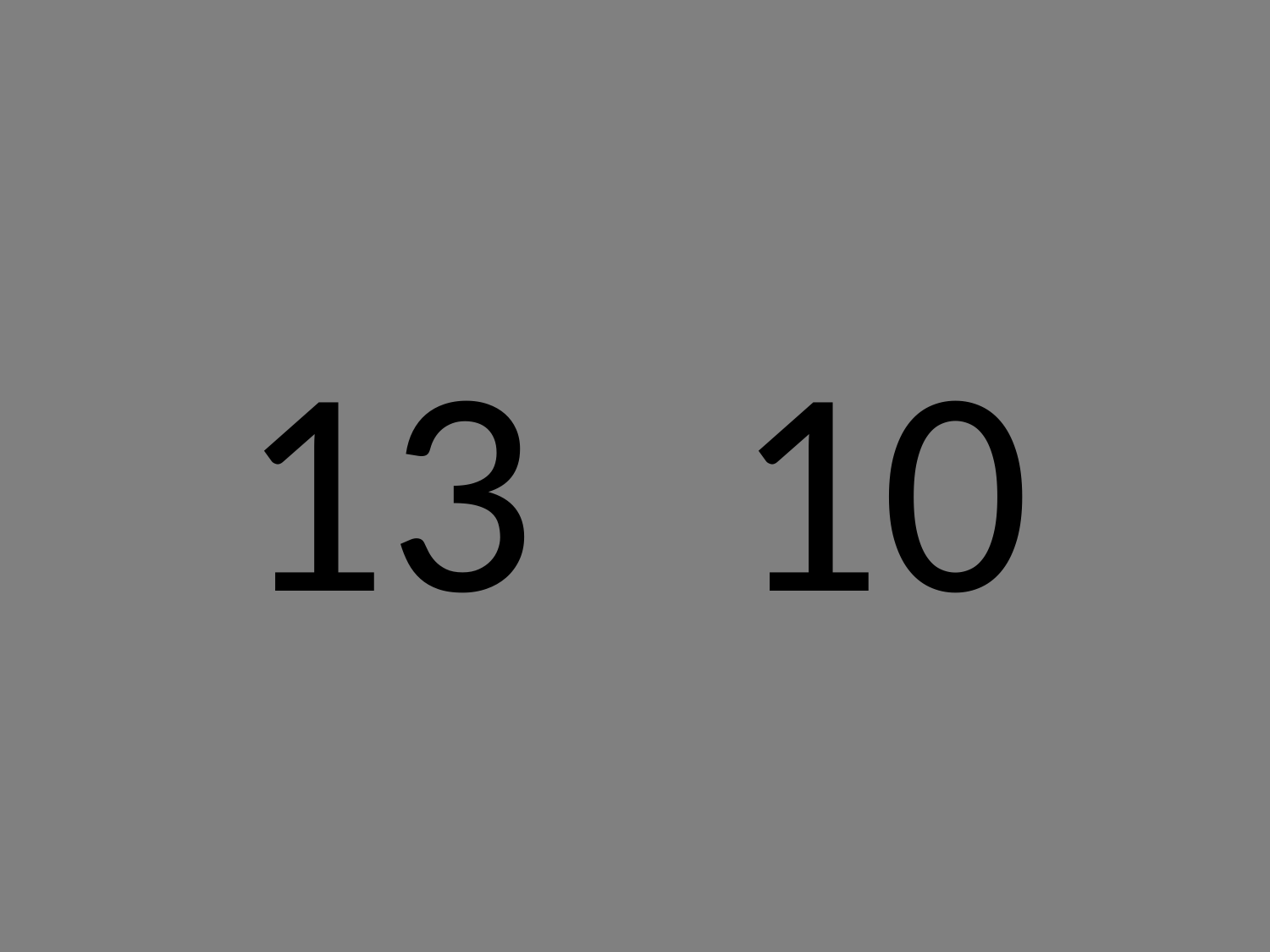

13 10

## Slide 57
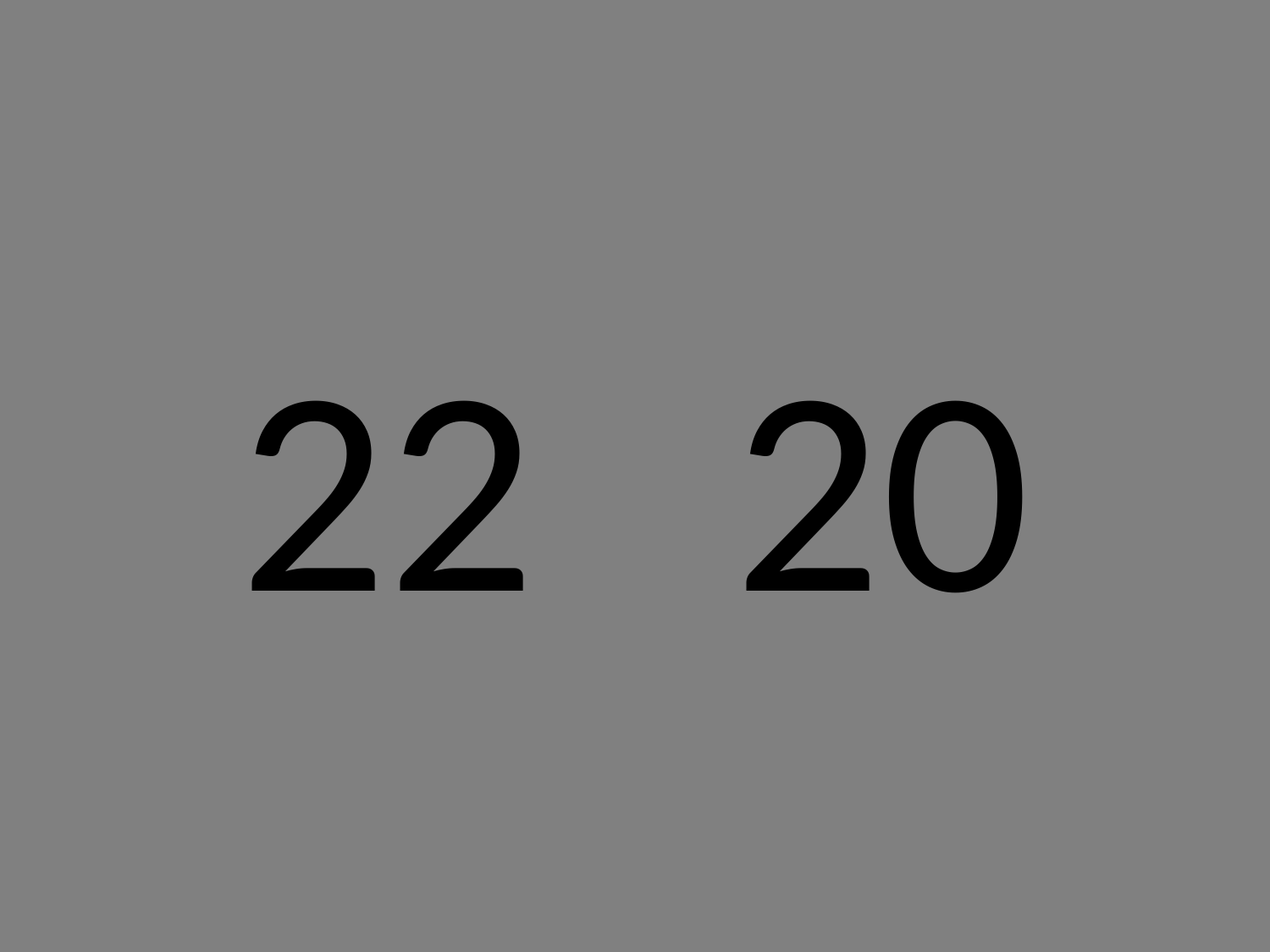

22 20

## Slide 58
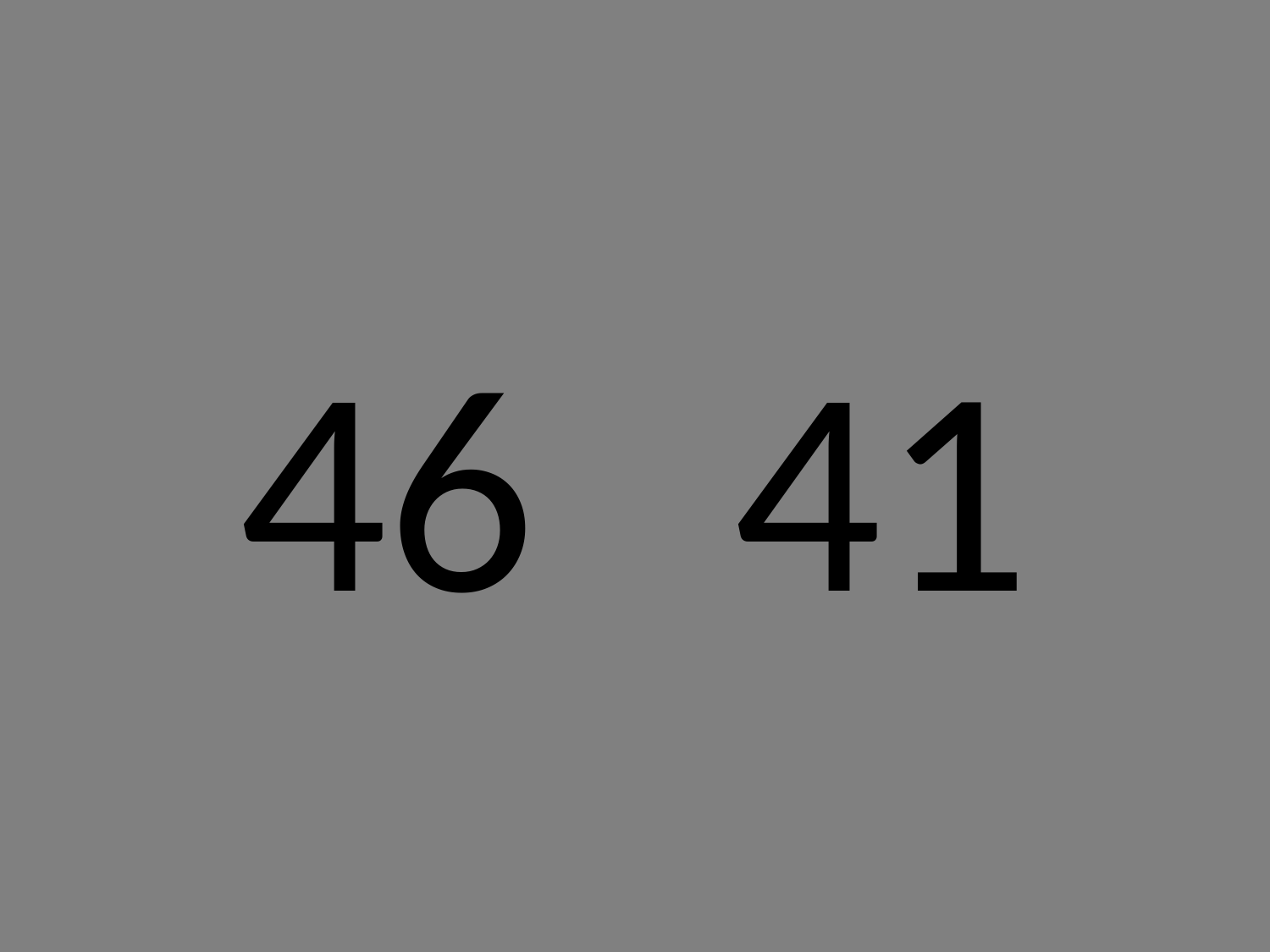

46 41

## Slide 59
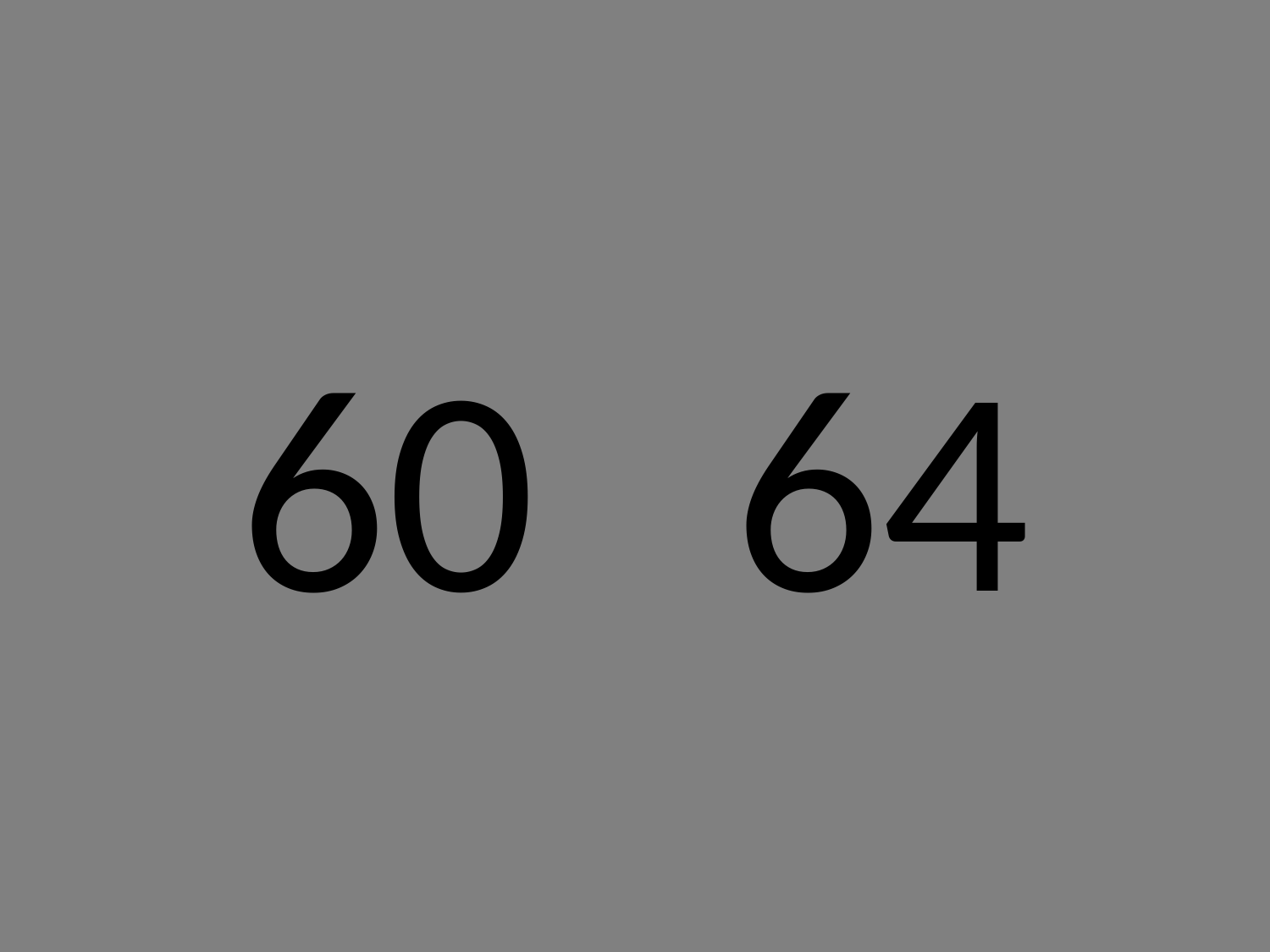

60 64

## Slide 60
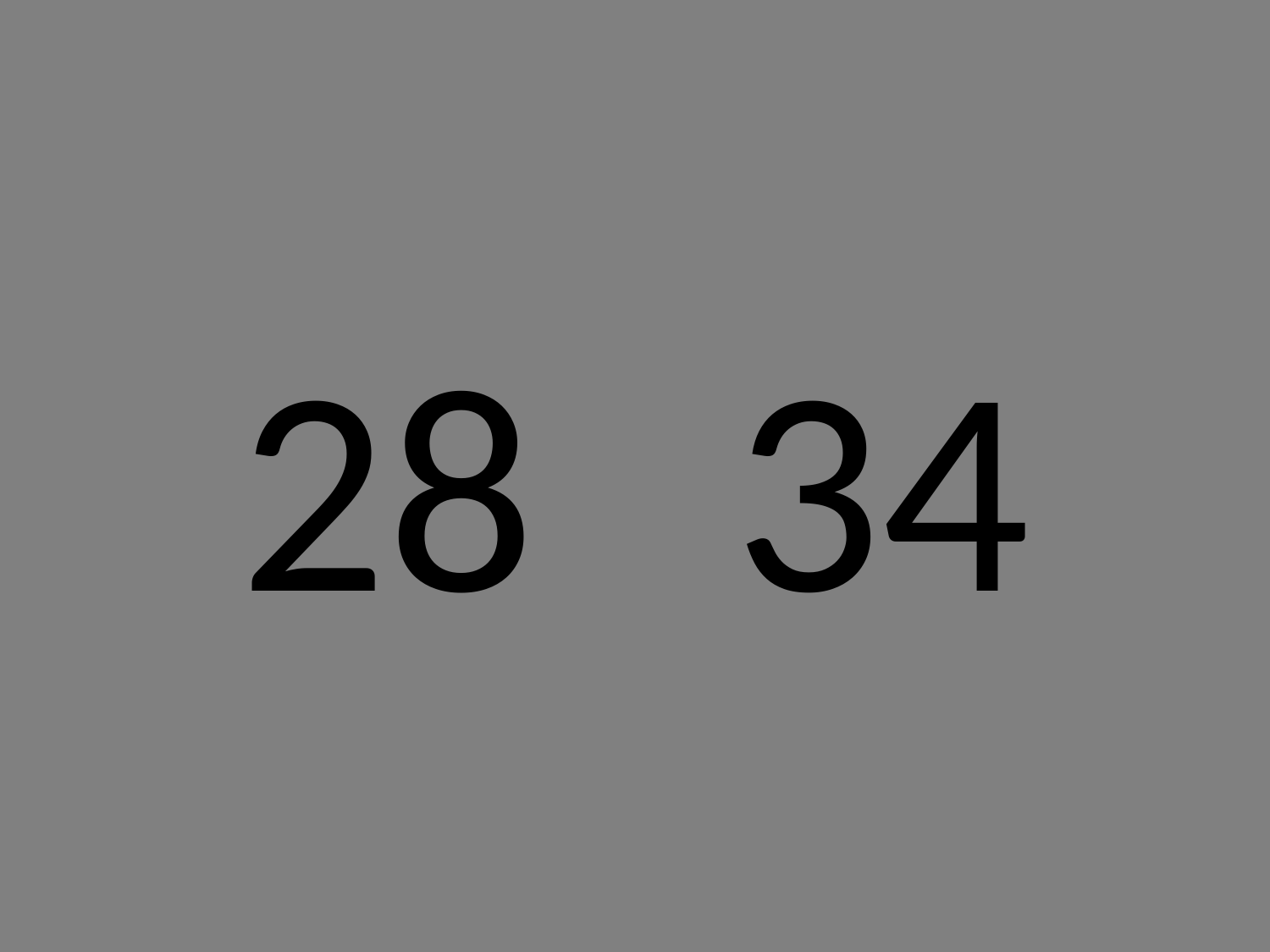

28 34

## Slide 61
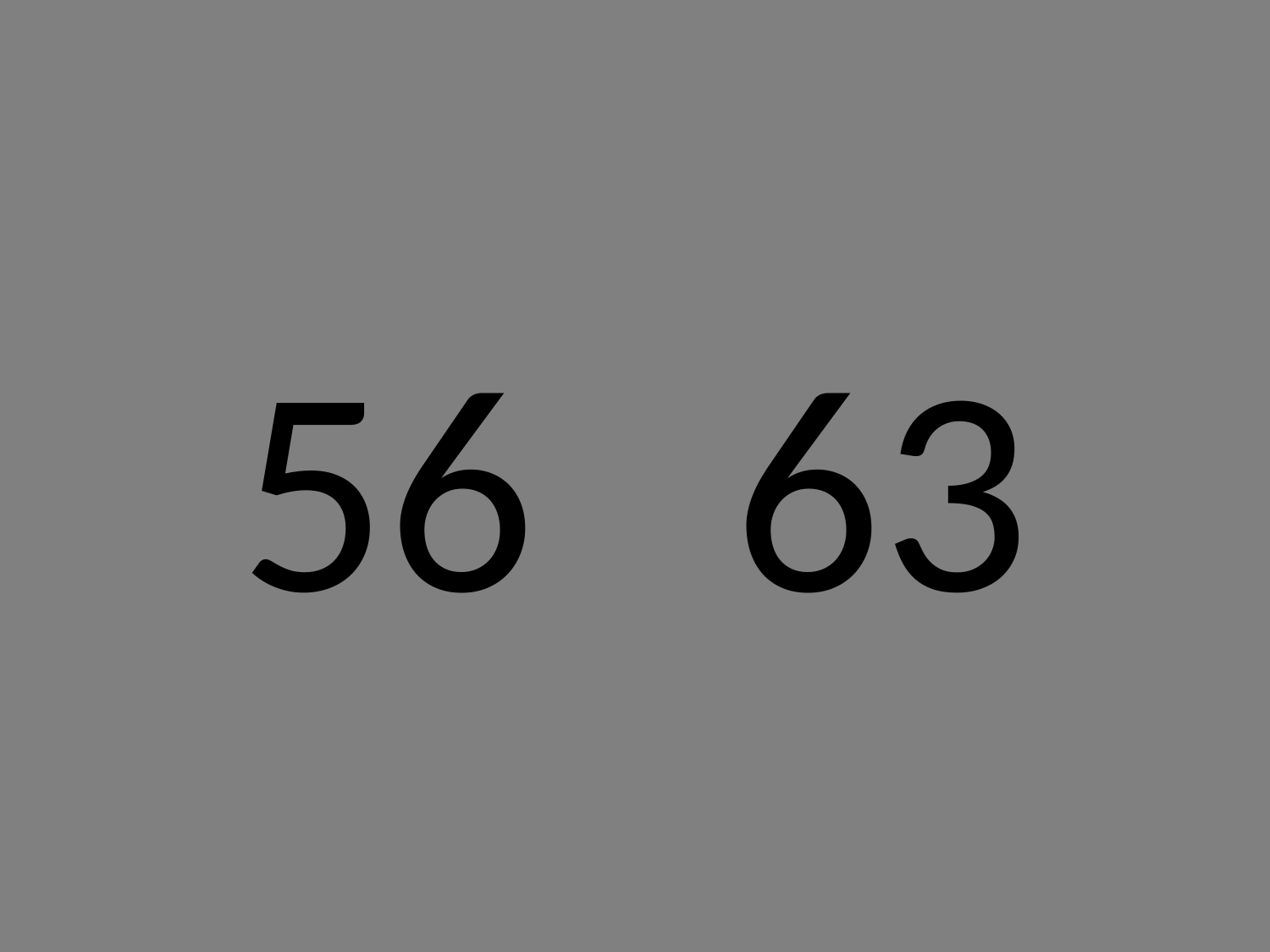

56 63

## Slide 62
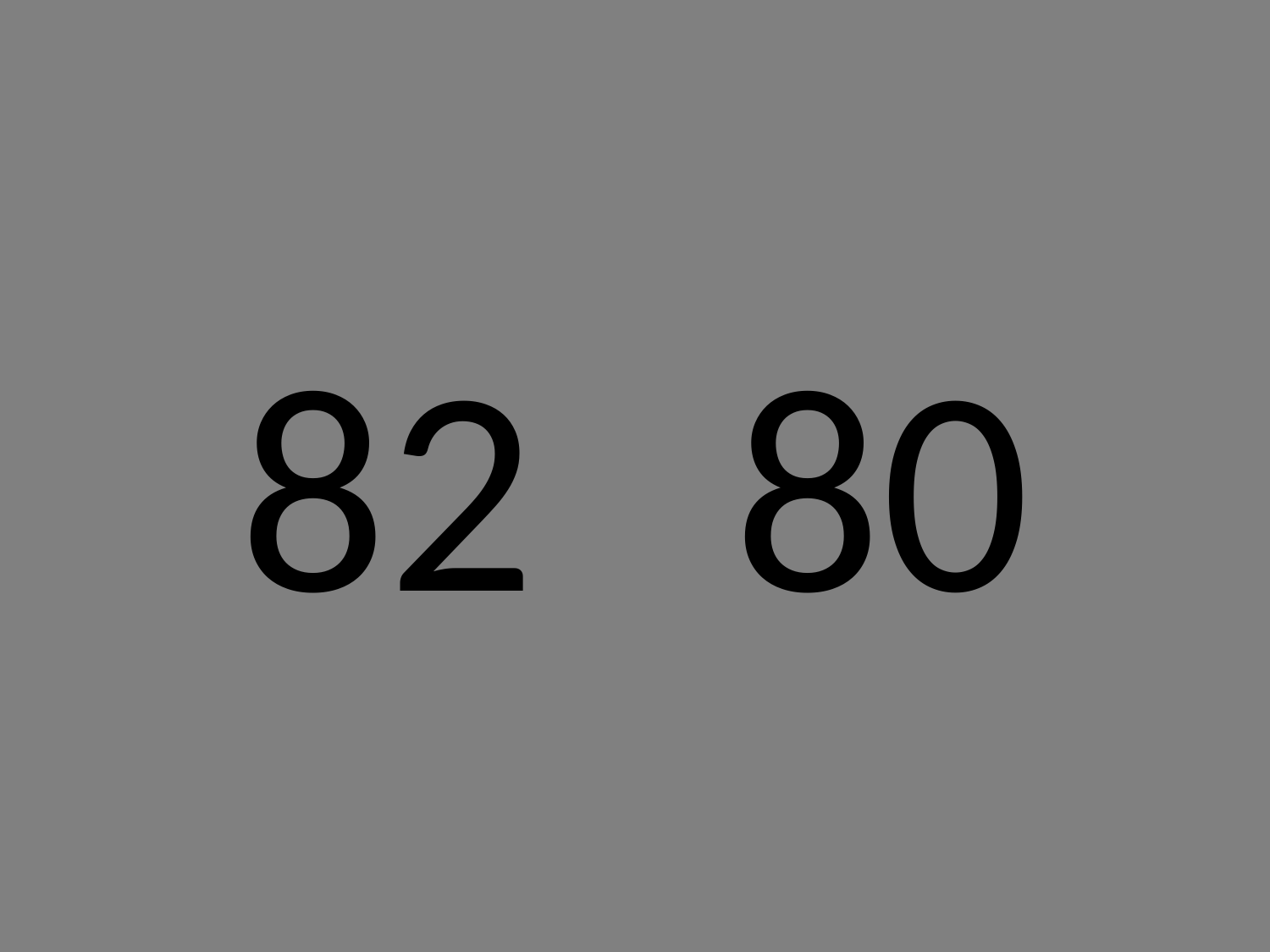

82 80

## Slide 63
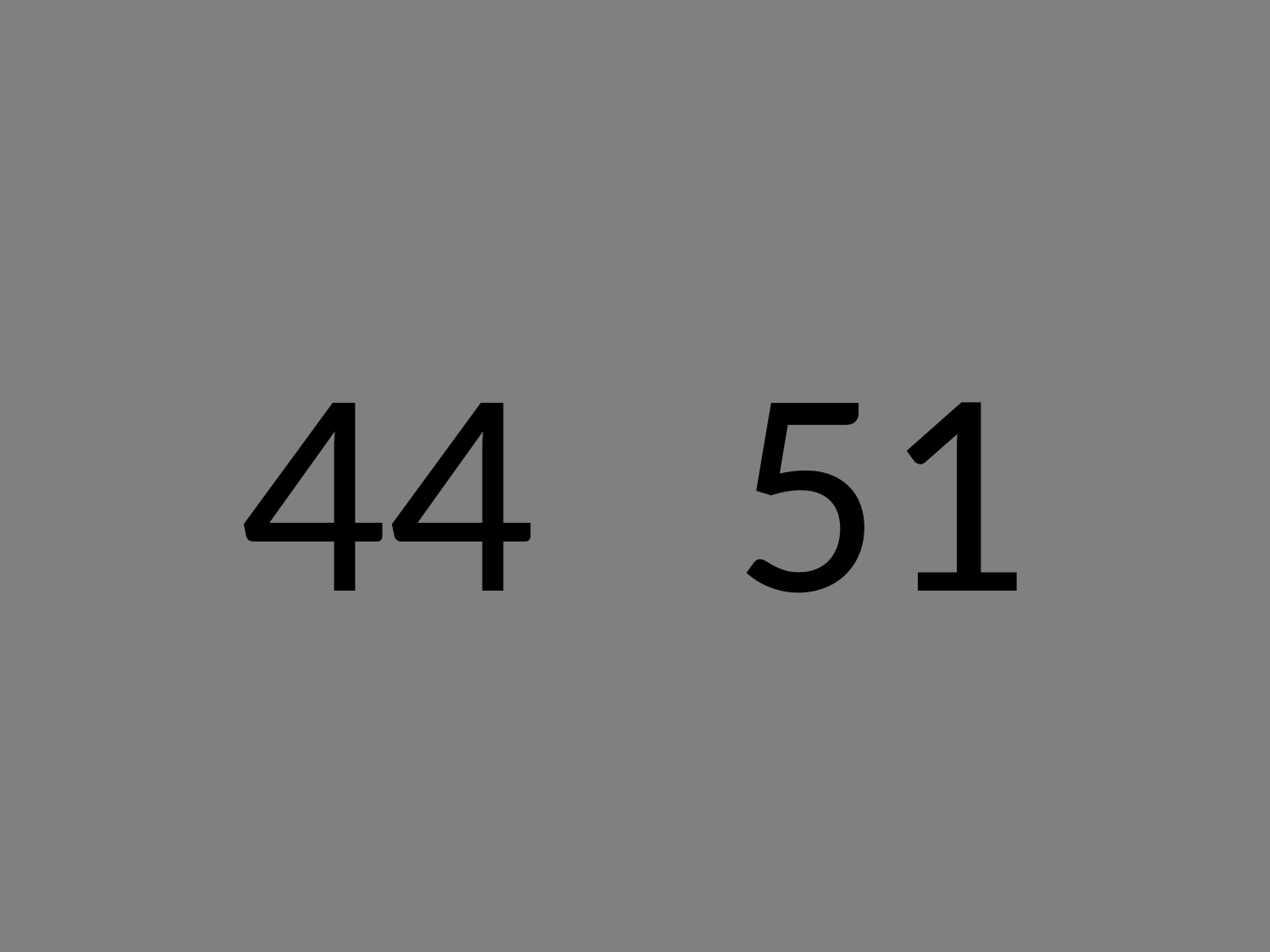

44 51

## Slide 64
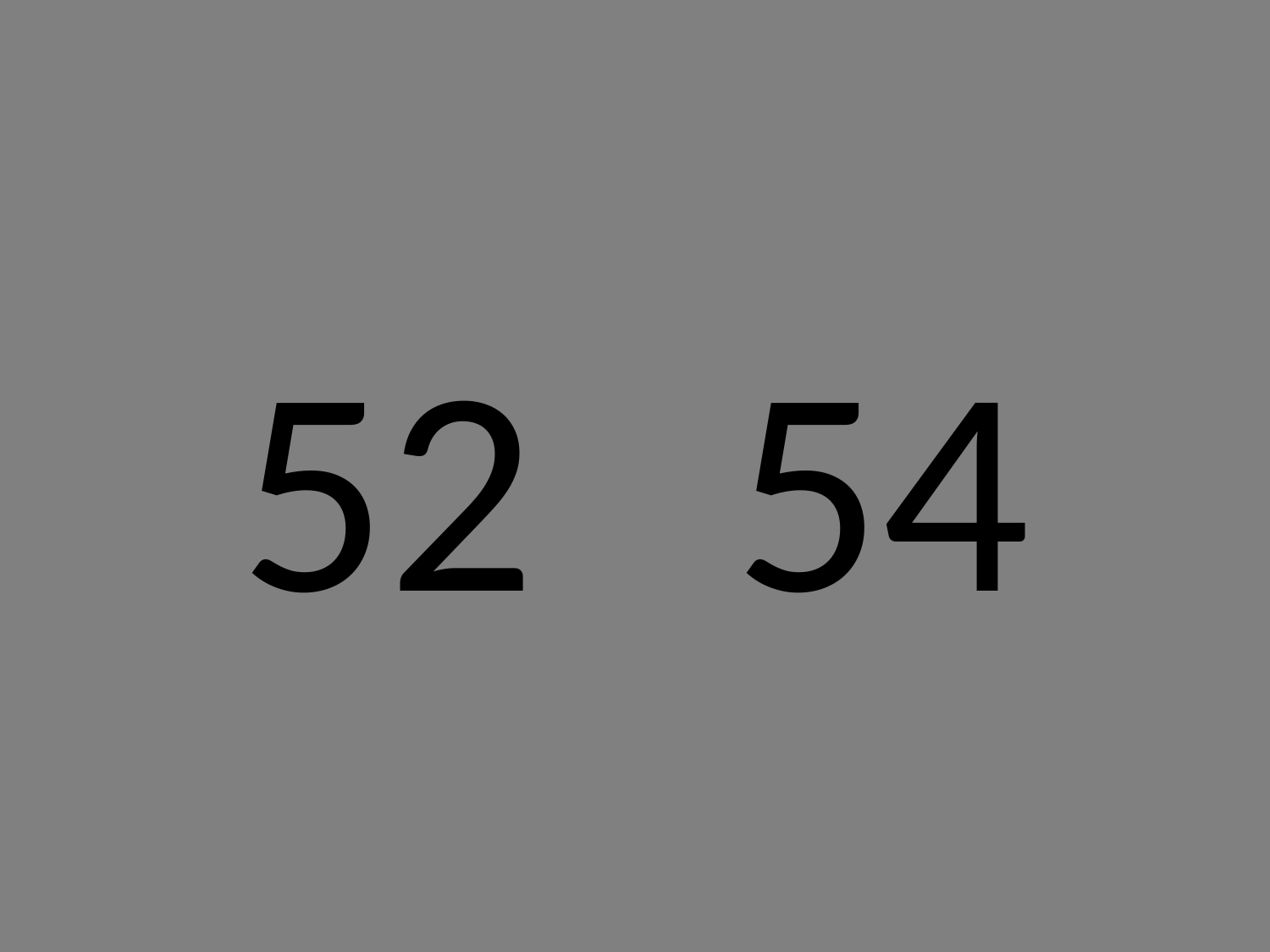

52 54

## Slide 65
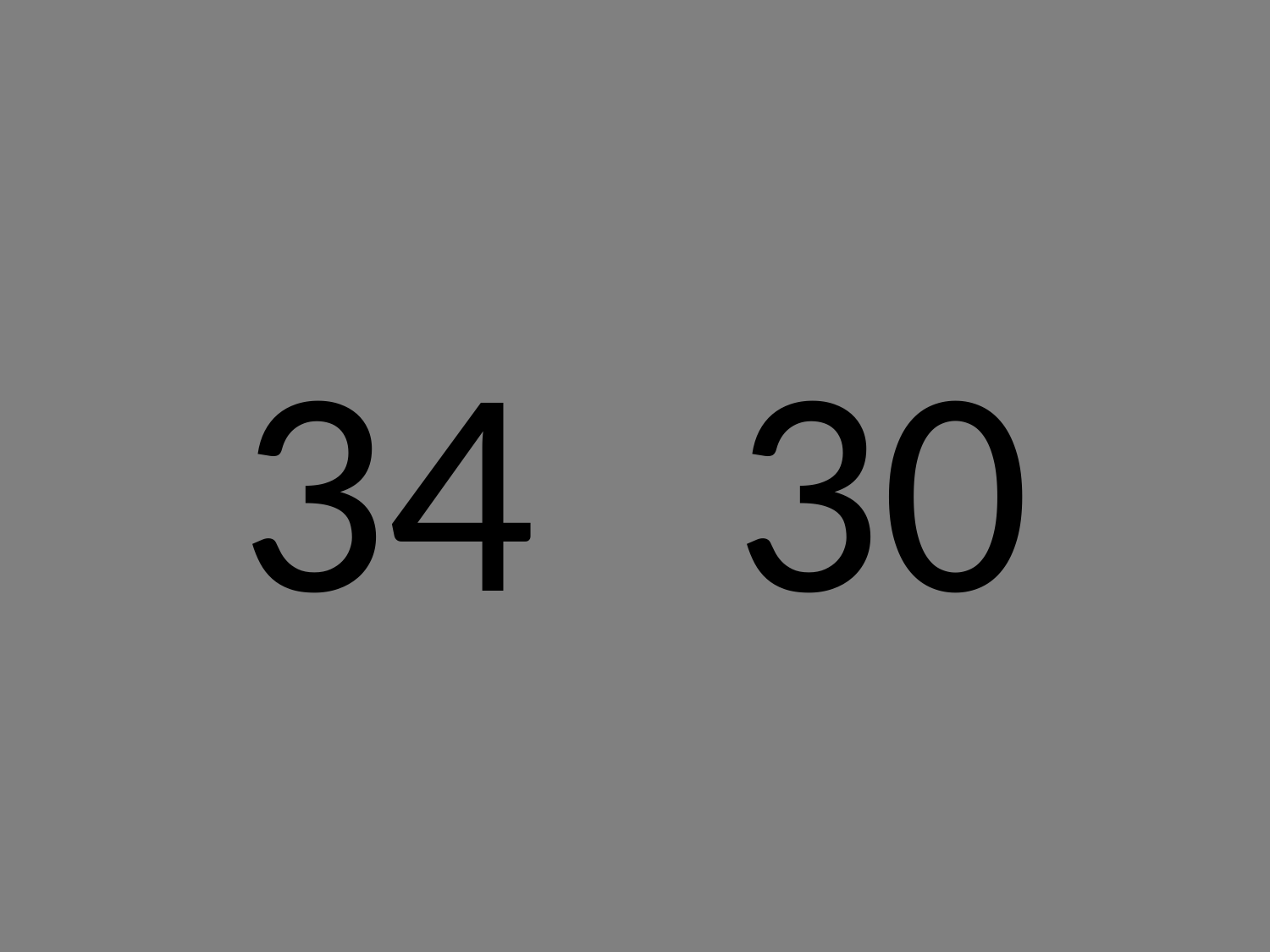

34 30

## Slide 66
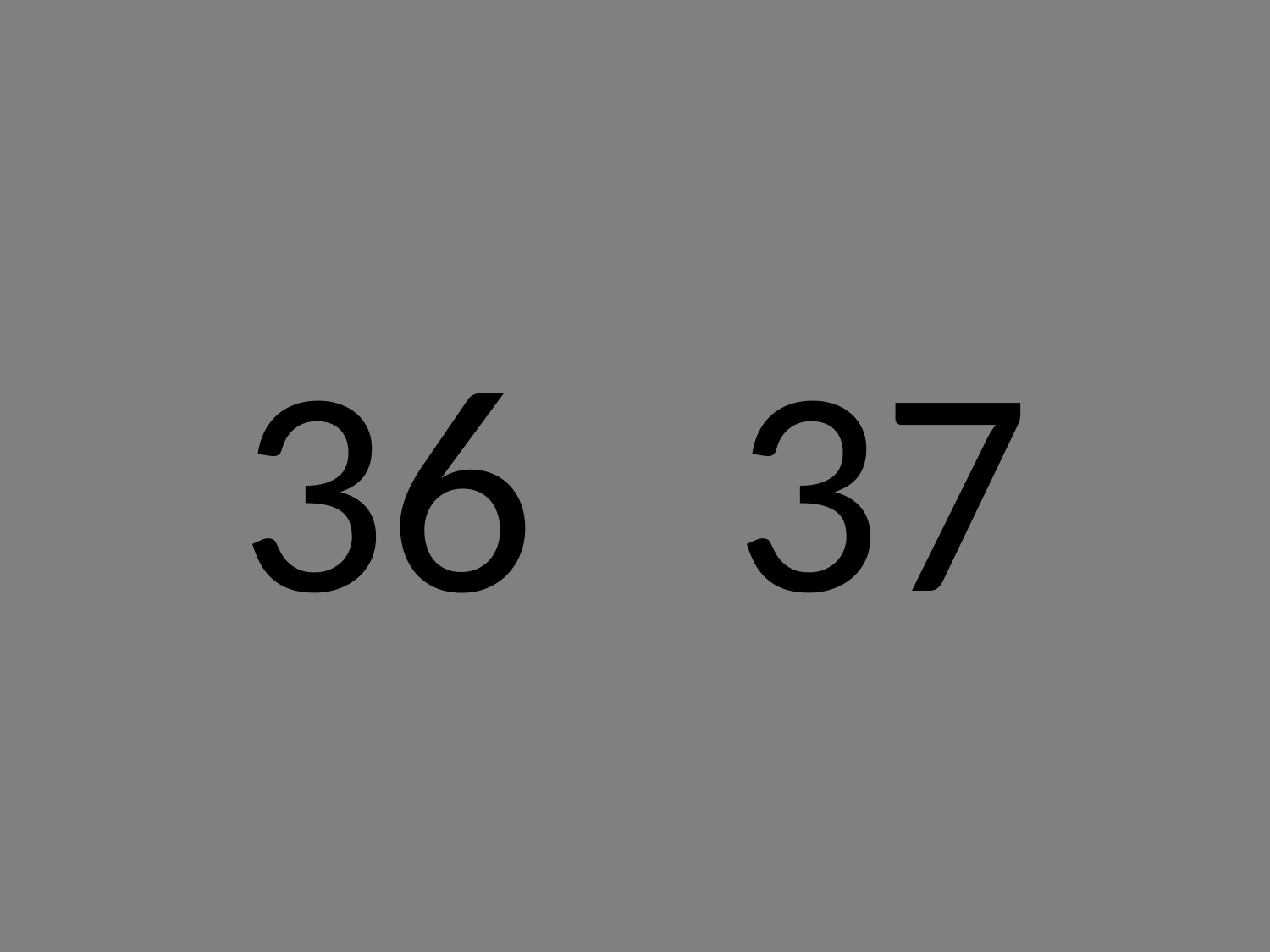

36 37

## Slide 67
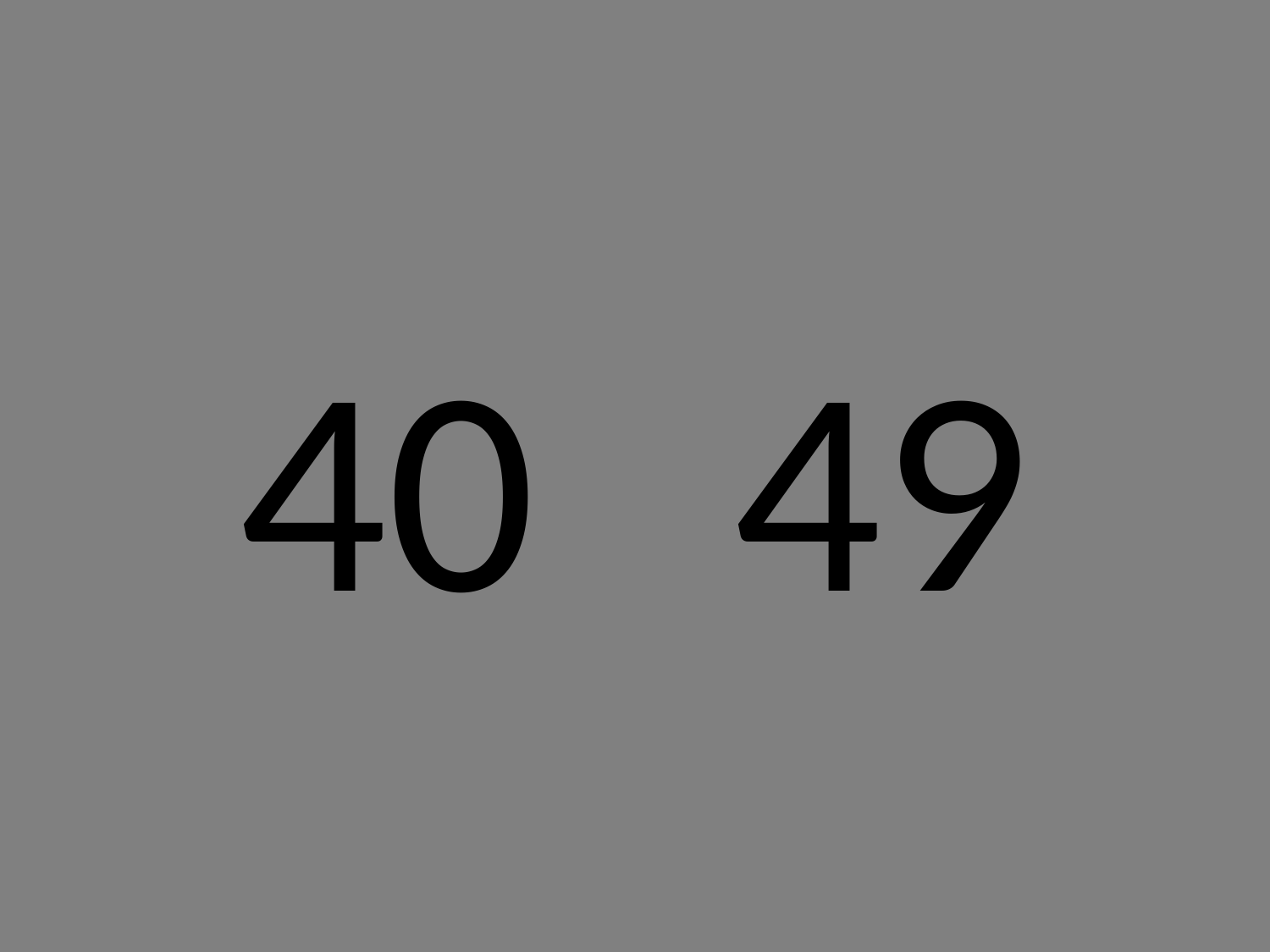

40 49

## Slide 68
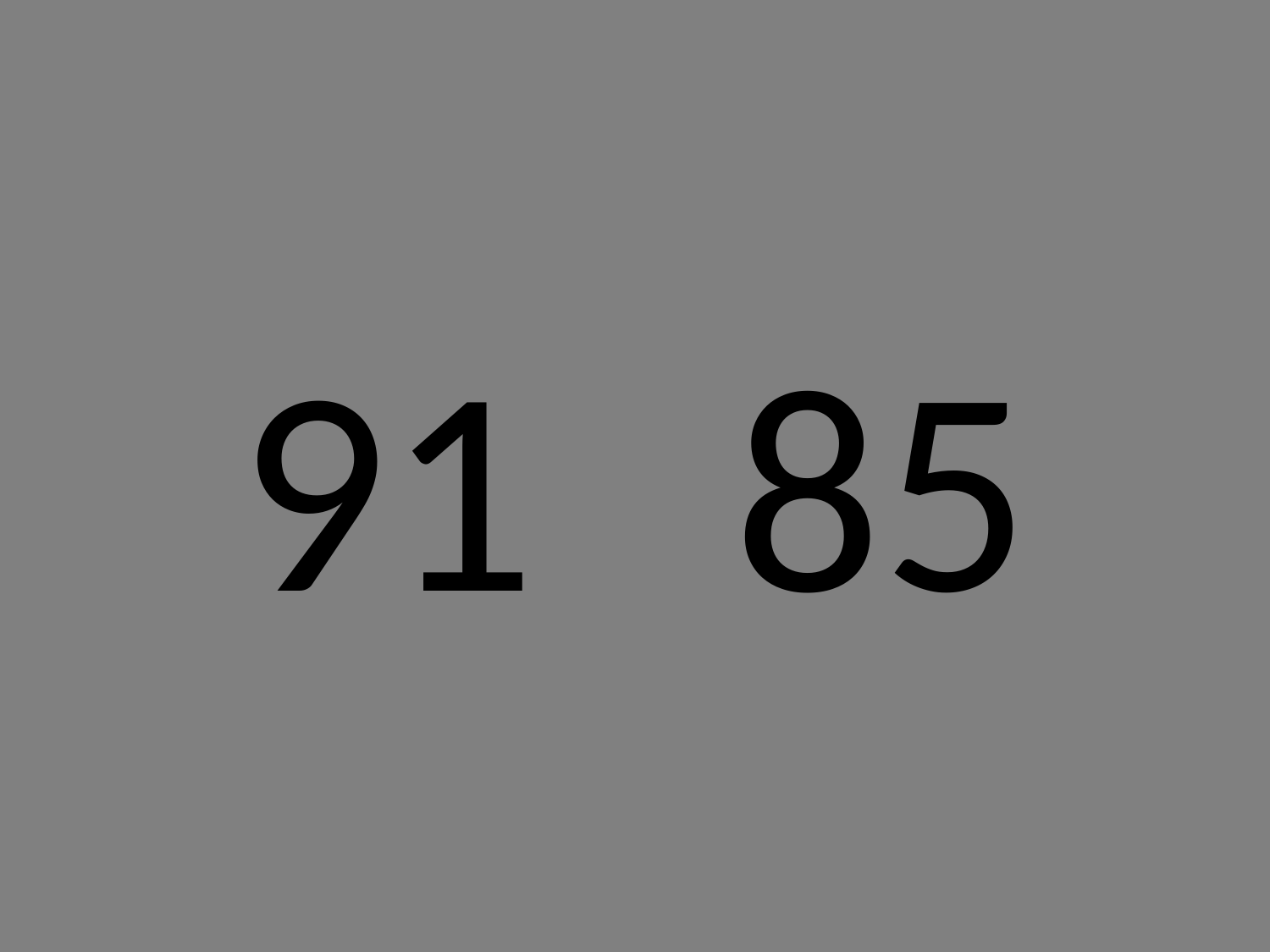

91 85

## Slide 69
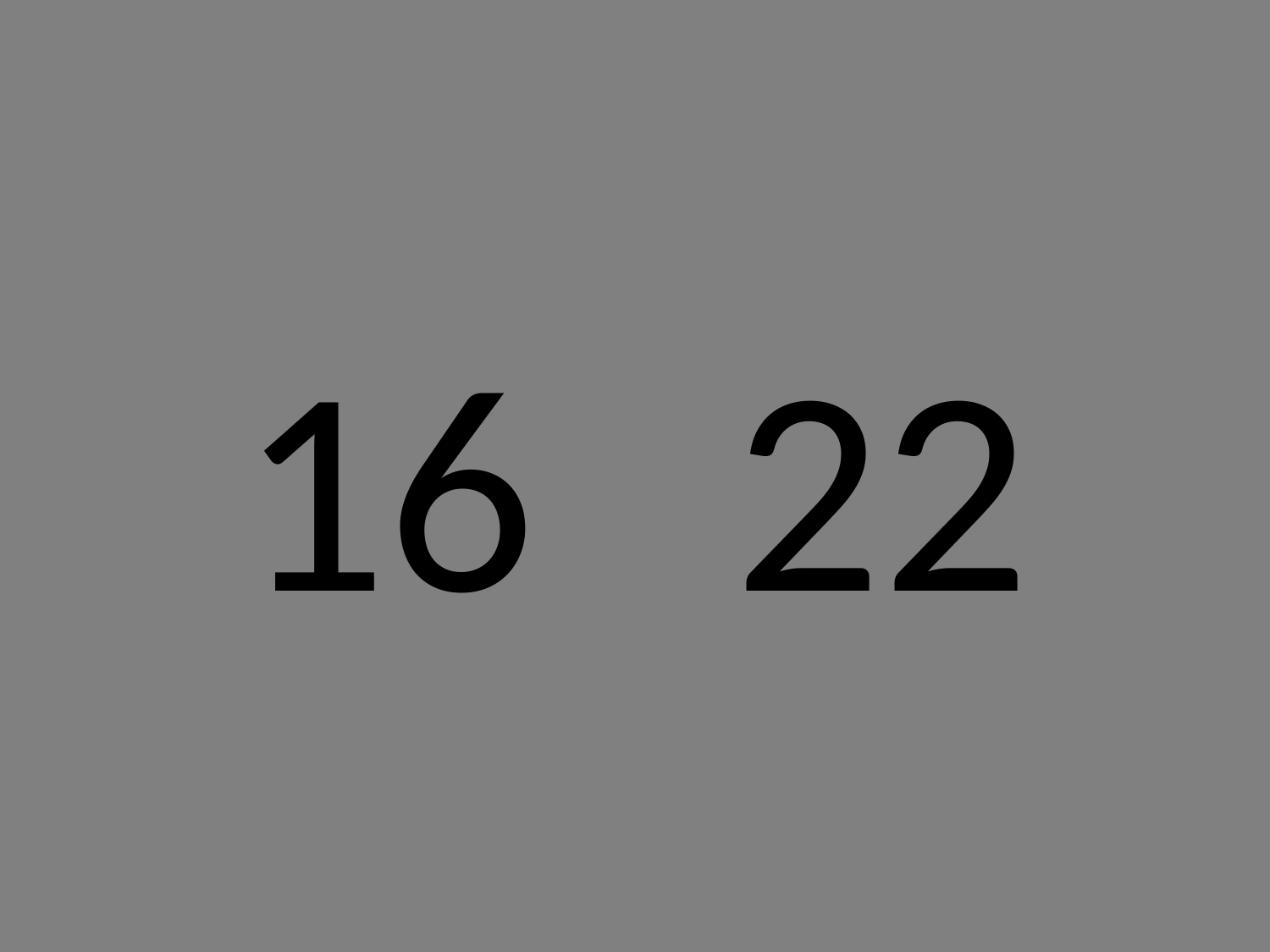

16 22

## Slide 70
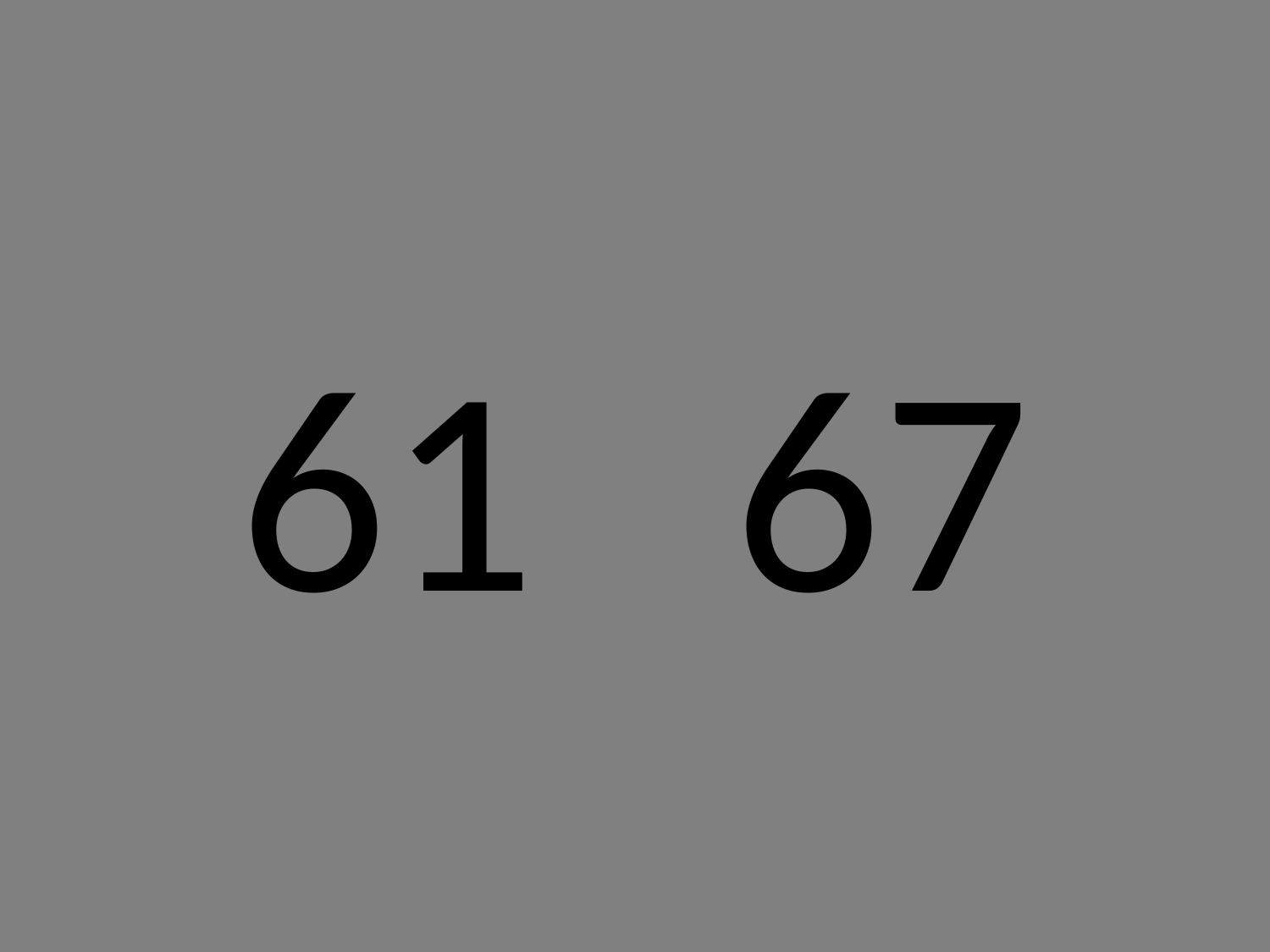

61 67

## Slide 71
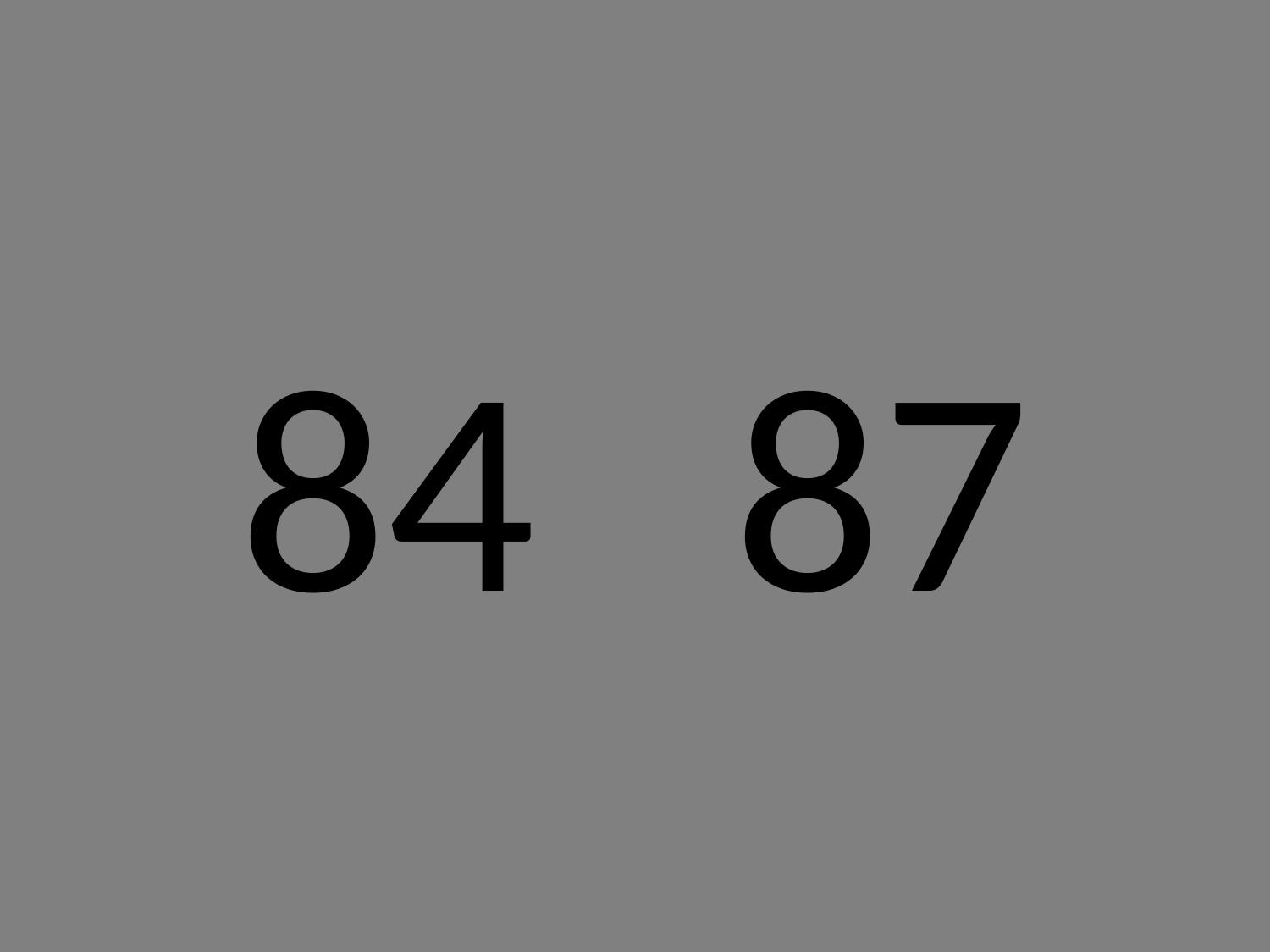

84 87

## Slide 72
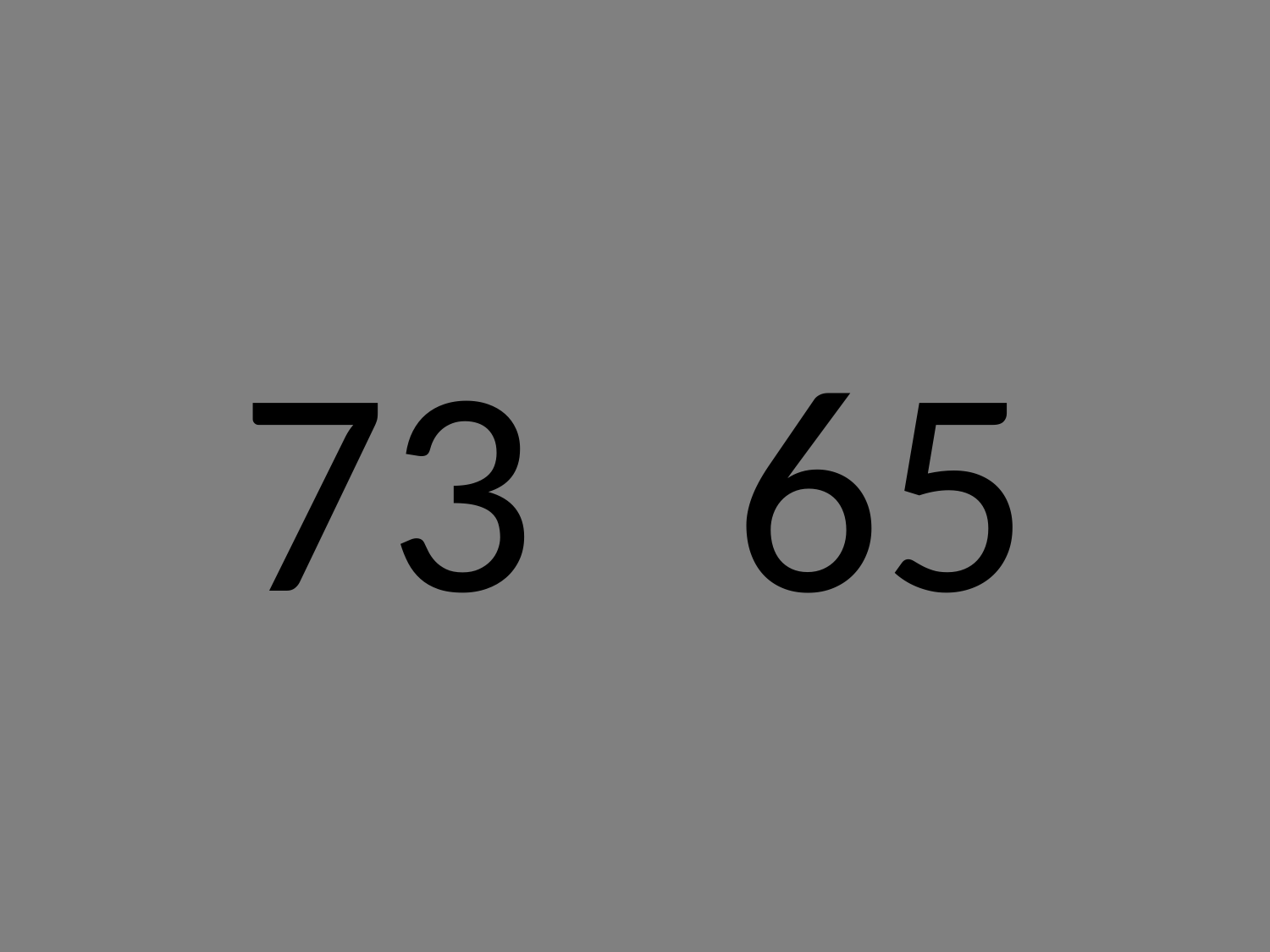

73 65

## Slide 73
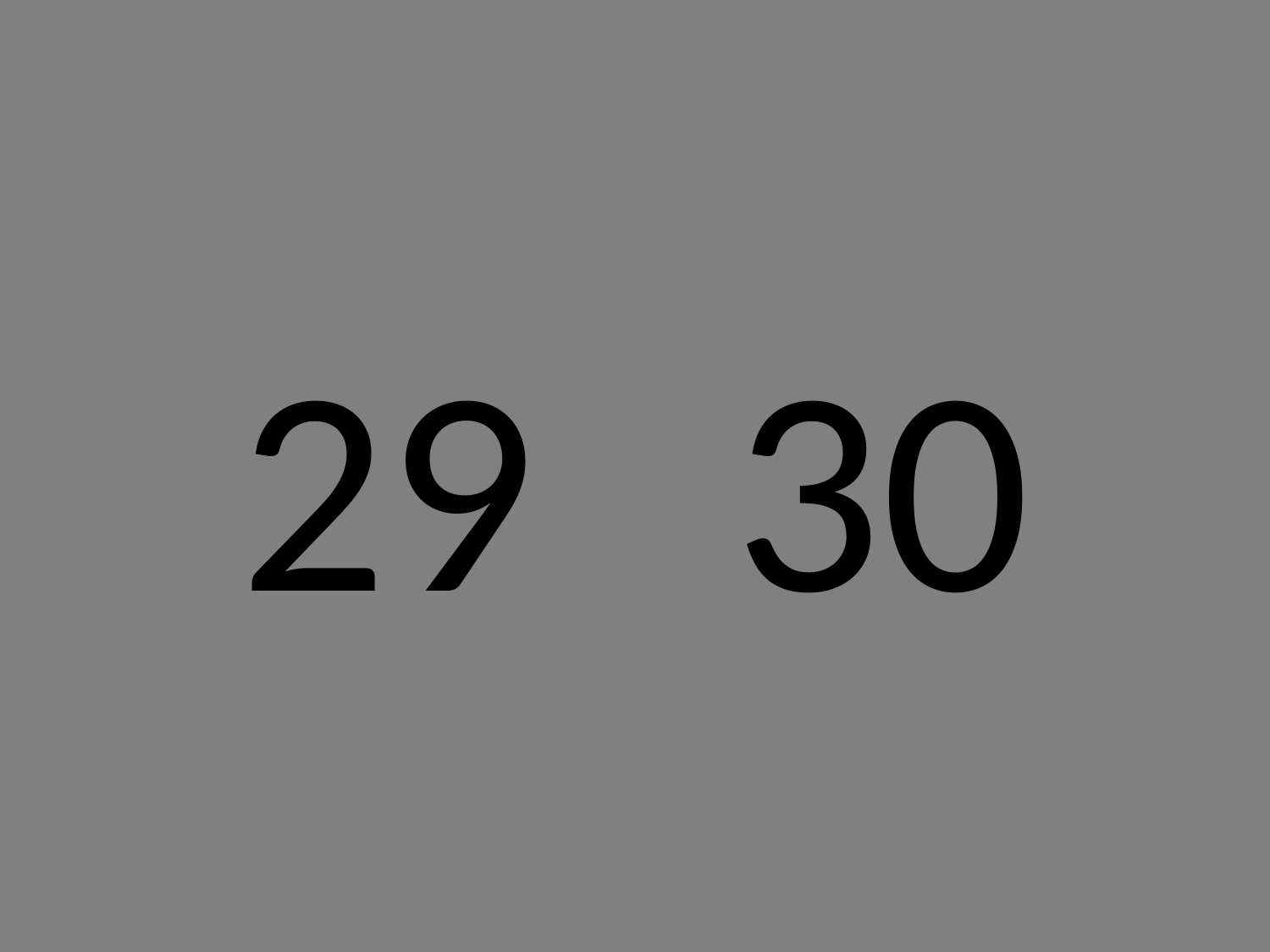

29 30

## Slide 74
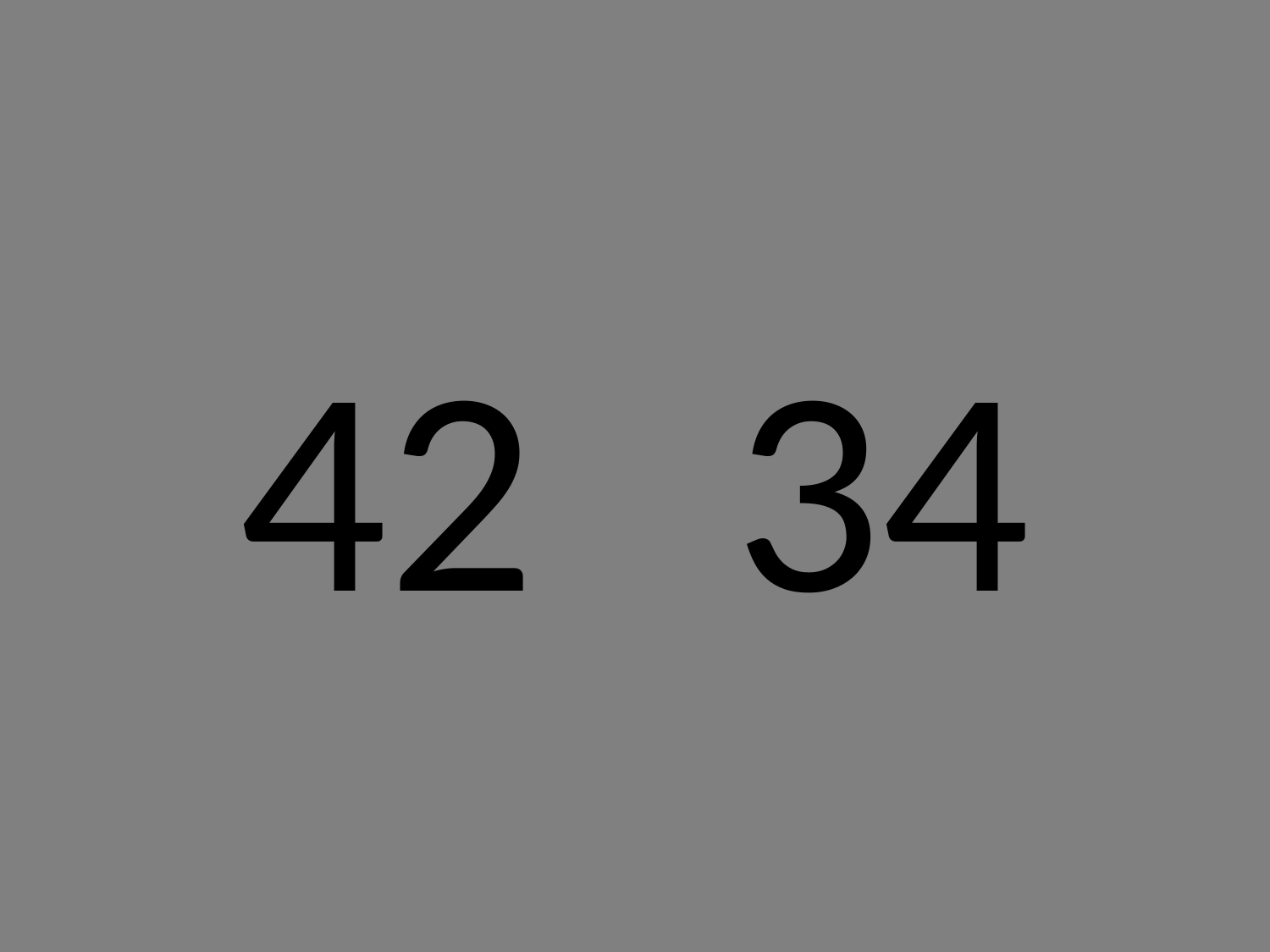

42 34

## Slide 75
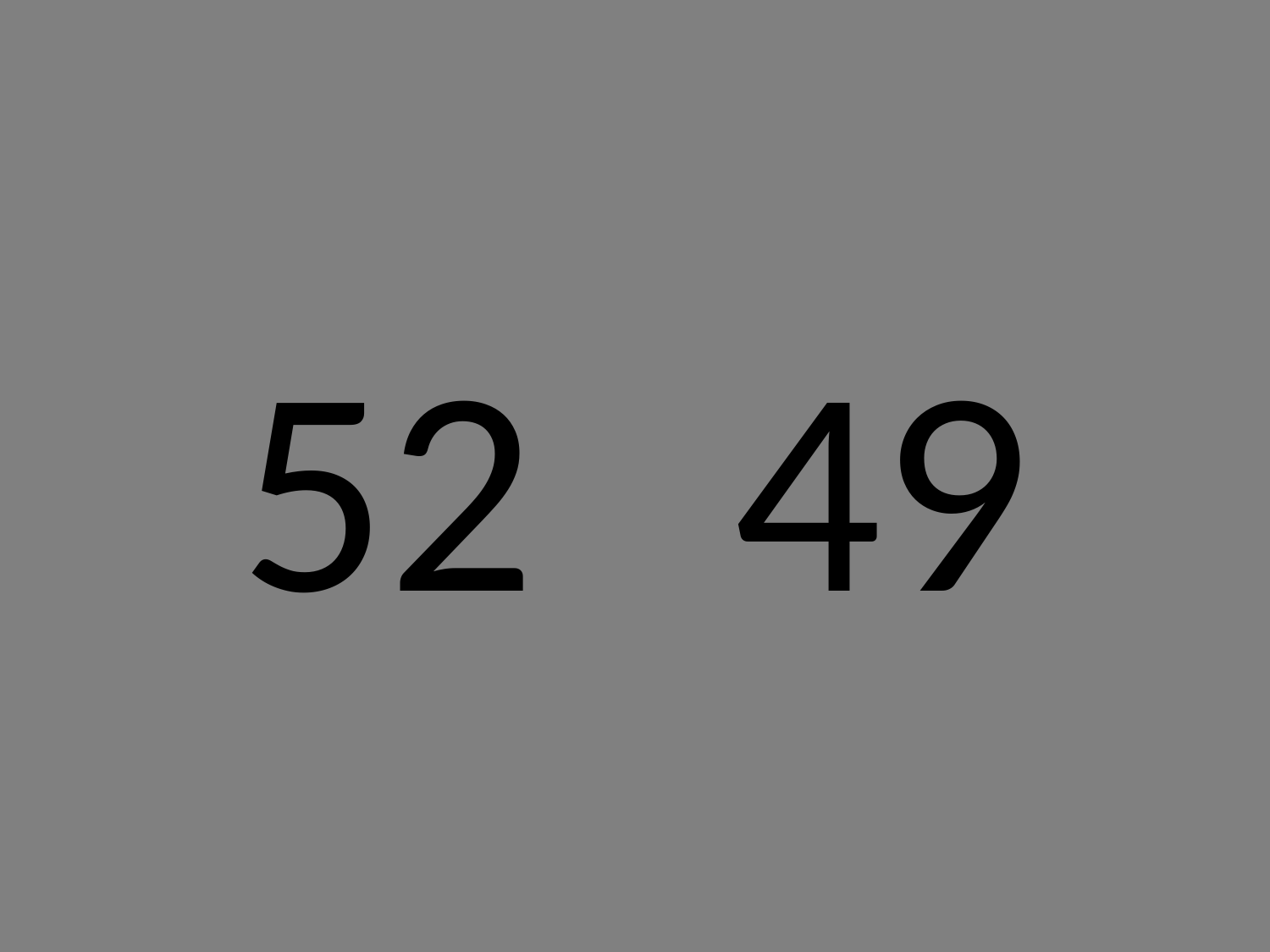

52 49

## Slide 76
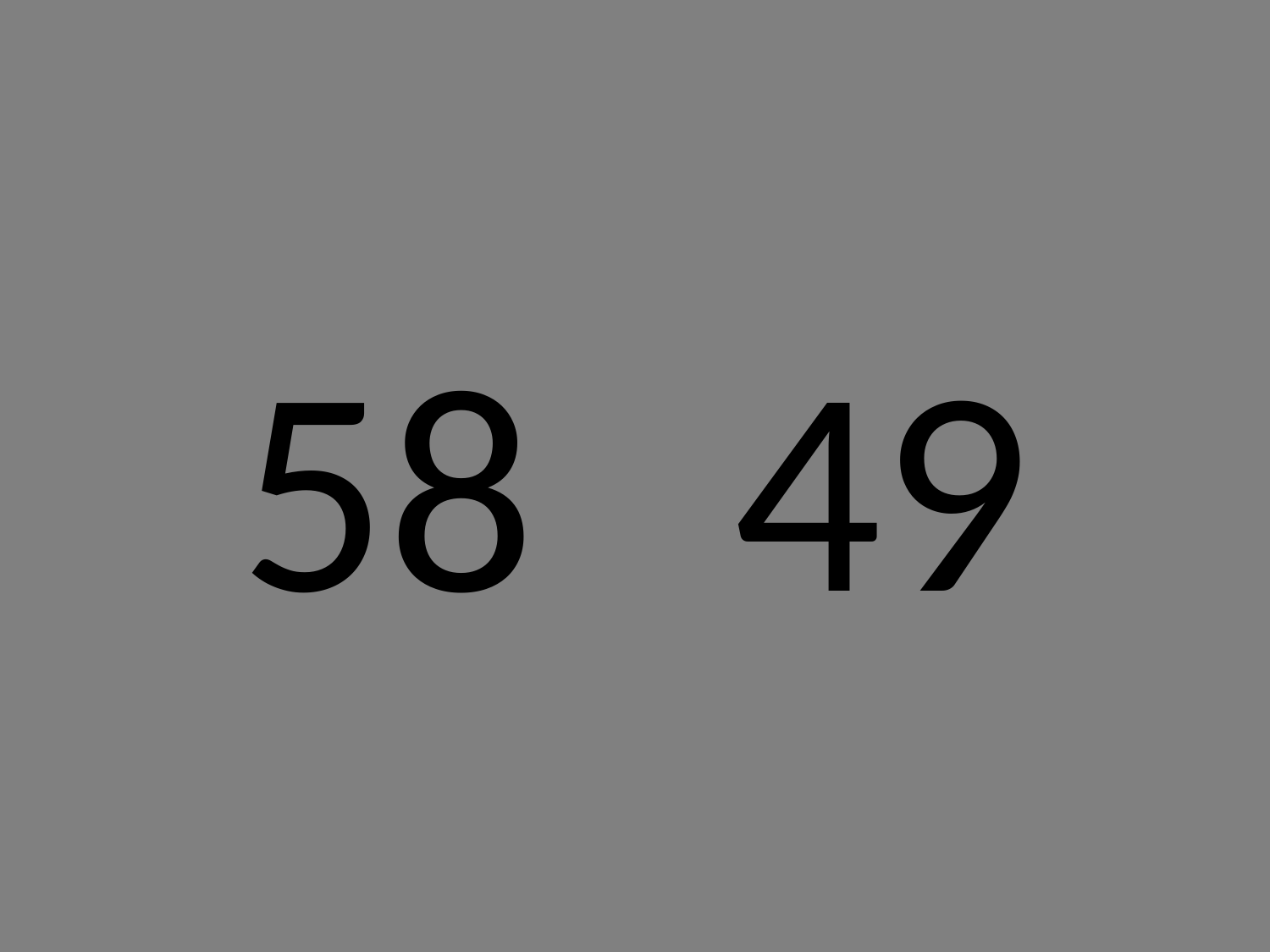

58 49

## Slide 77
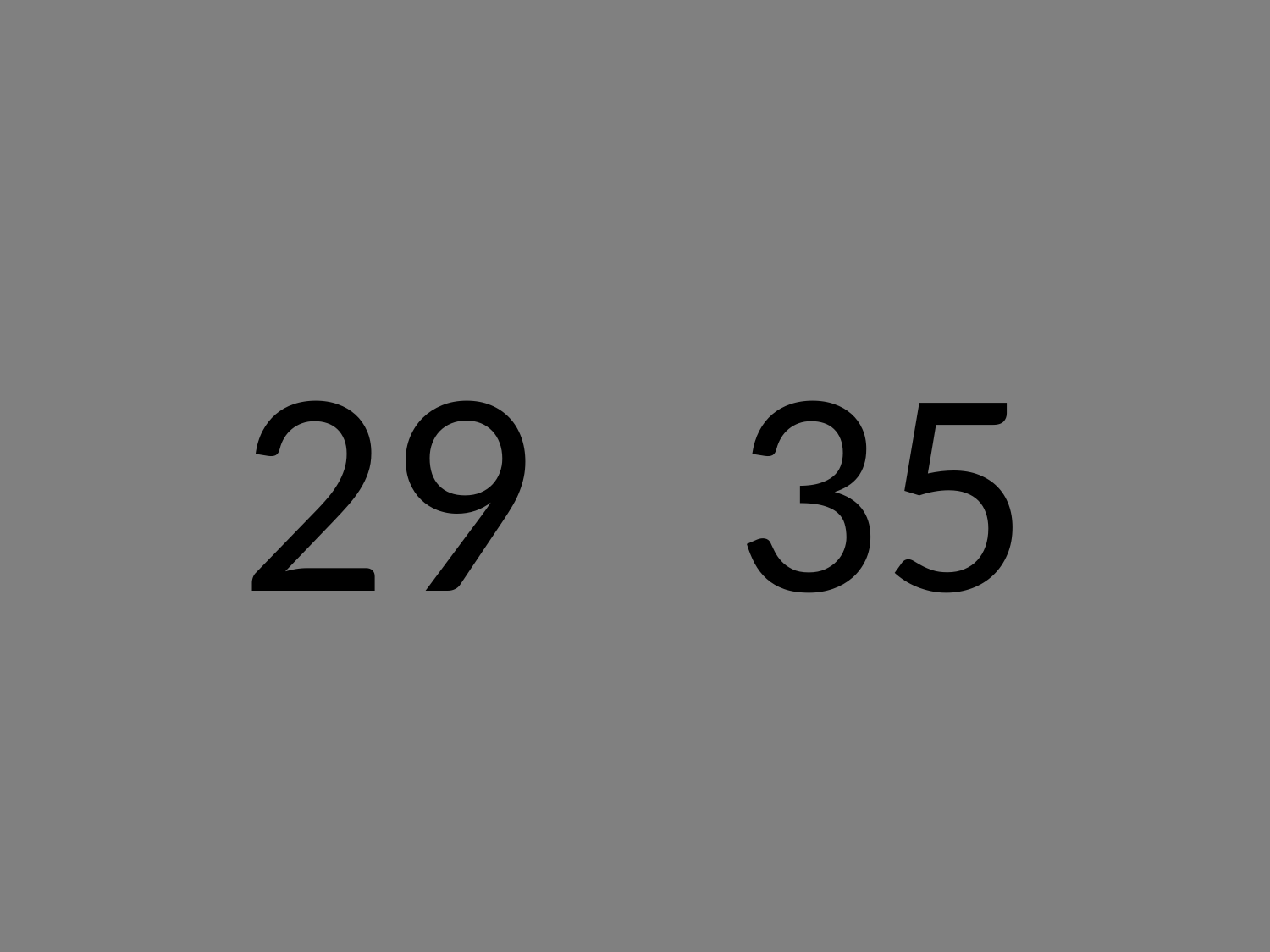

29 35

## Slide 78
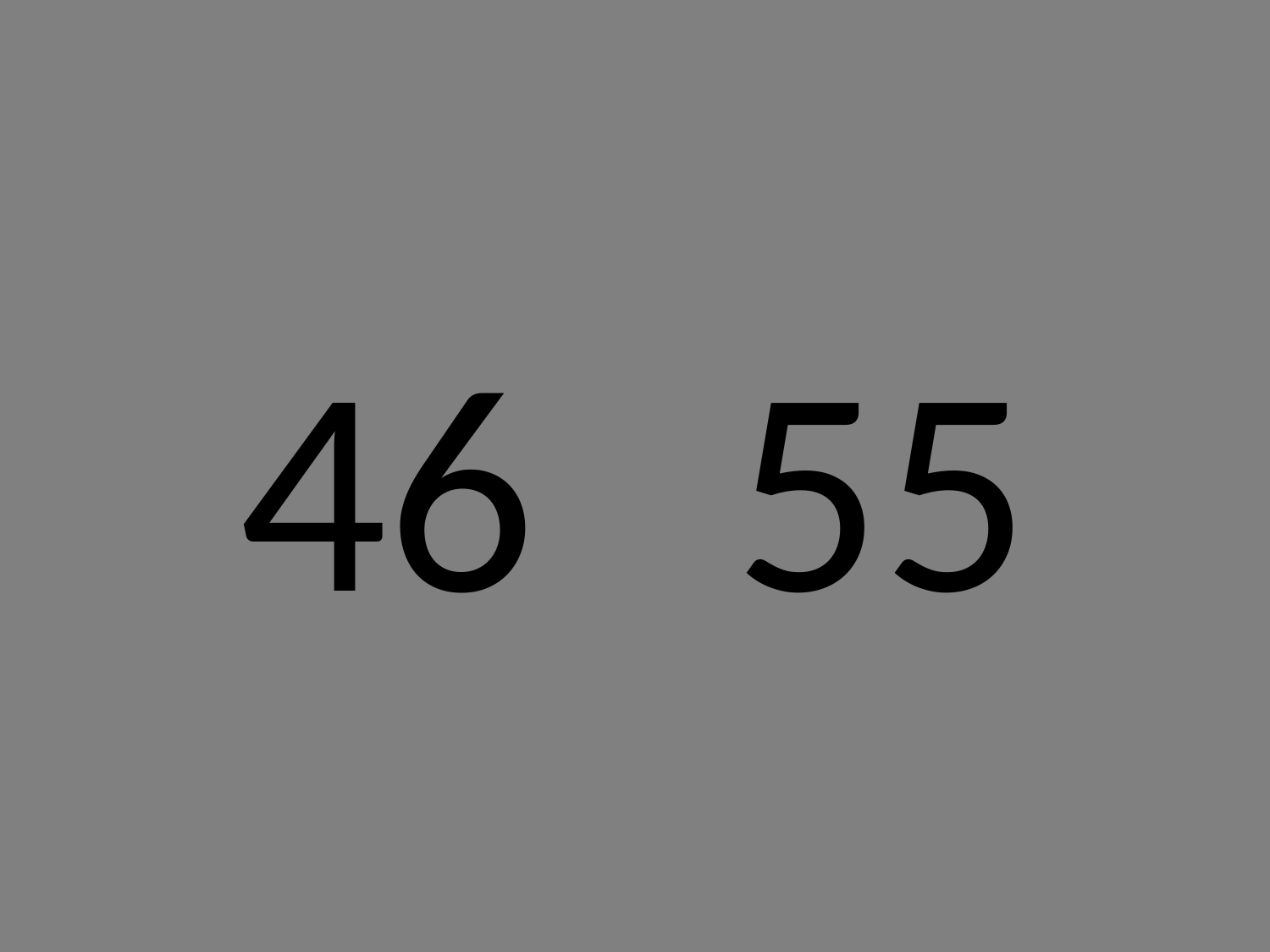

46 55

## Slide 79
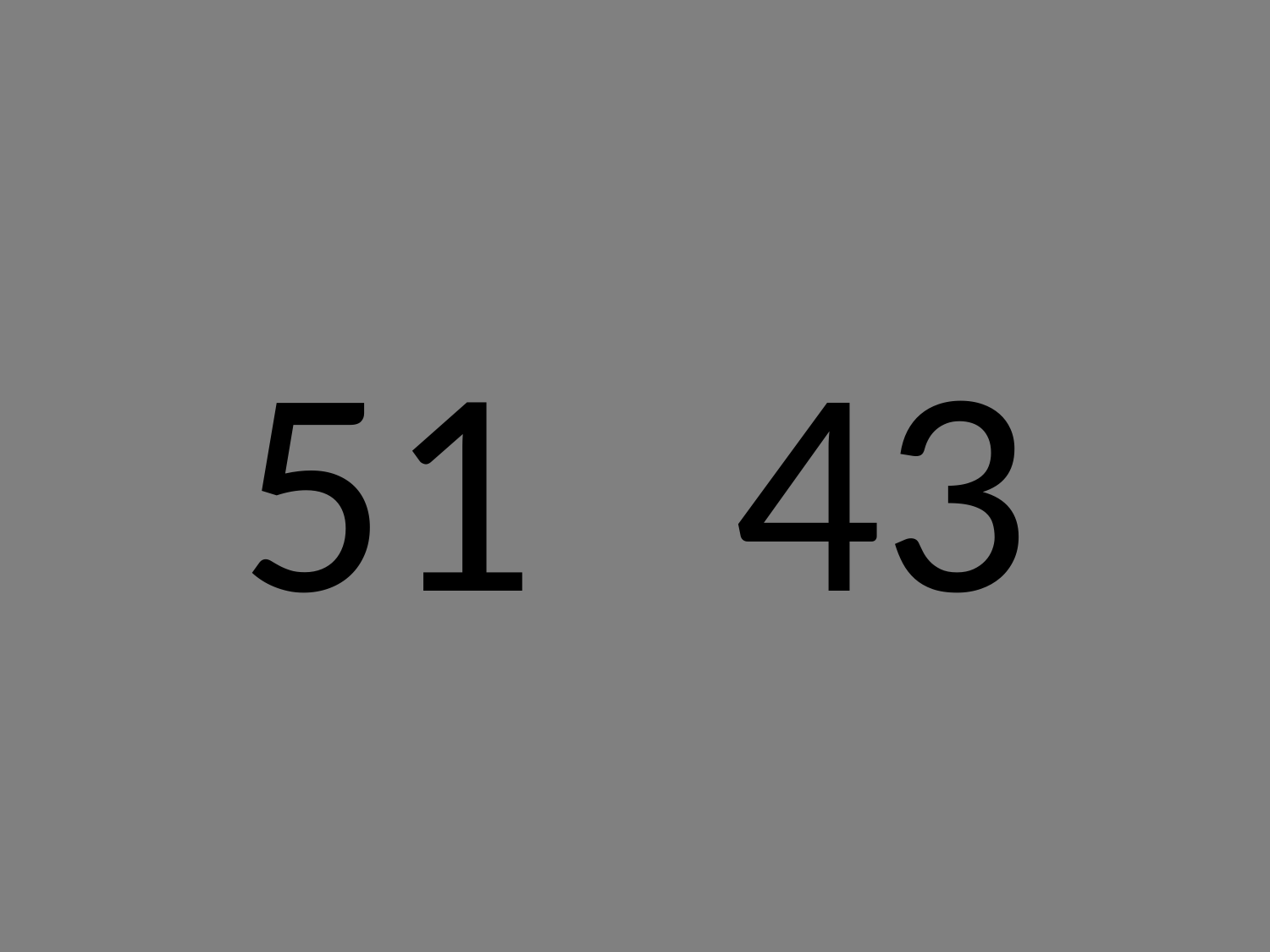

51 43

## Slide 80
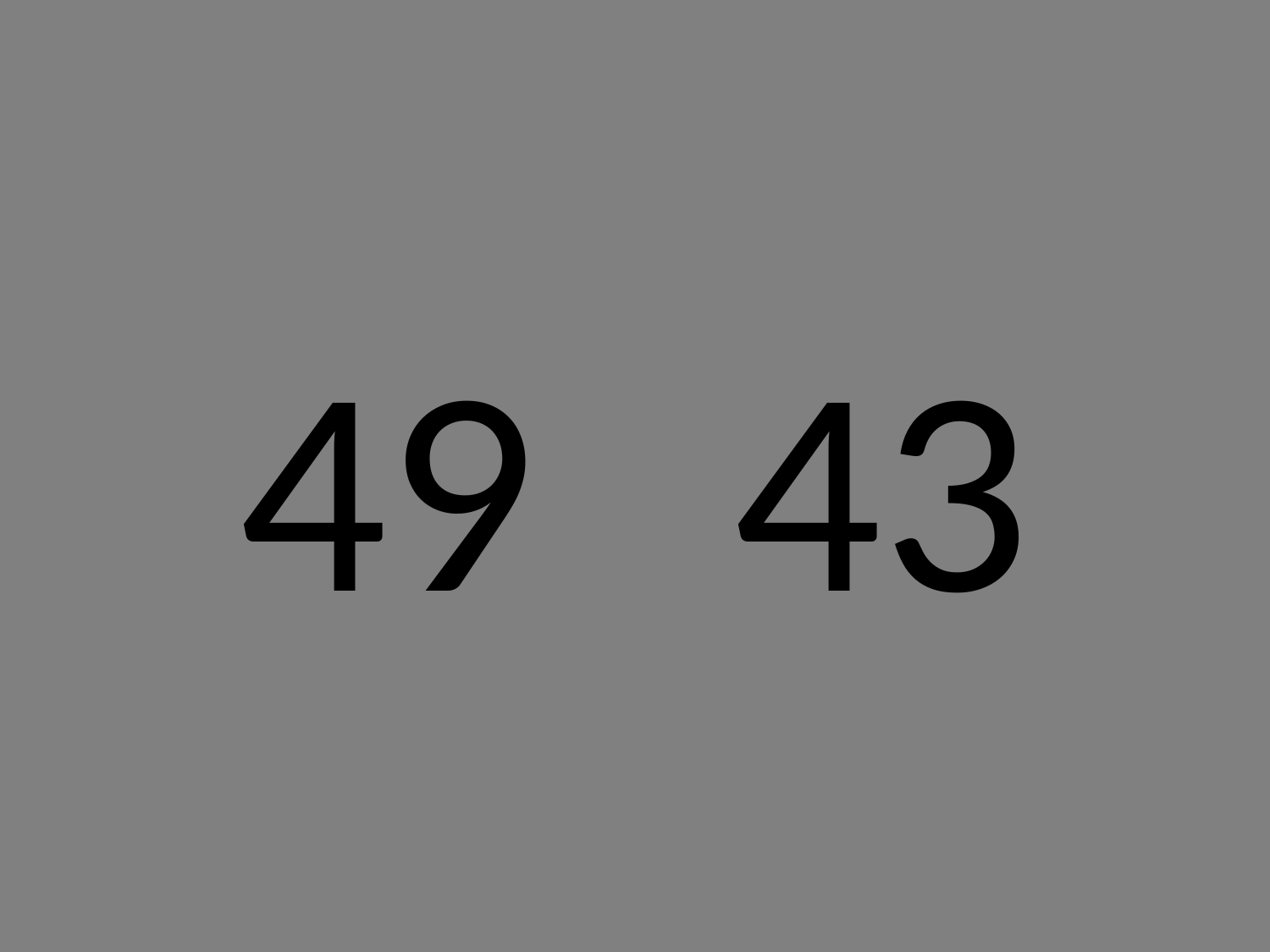

49 43

## Slide 81
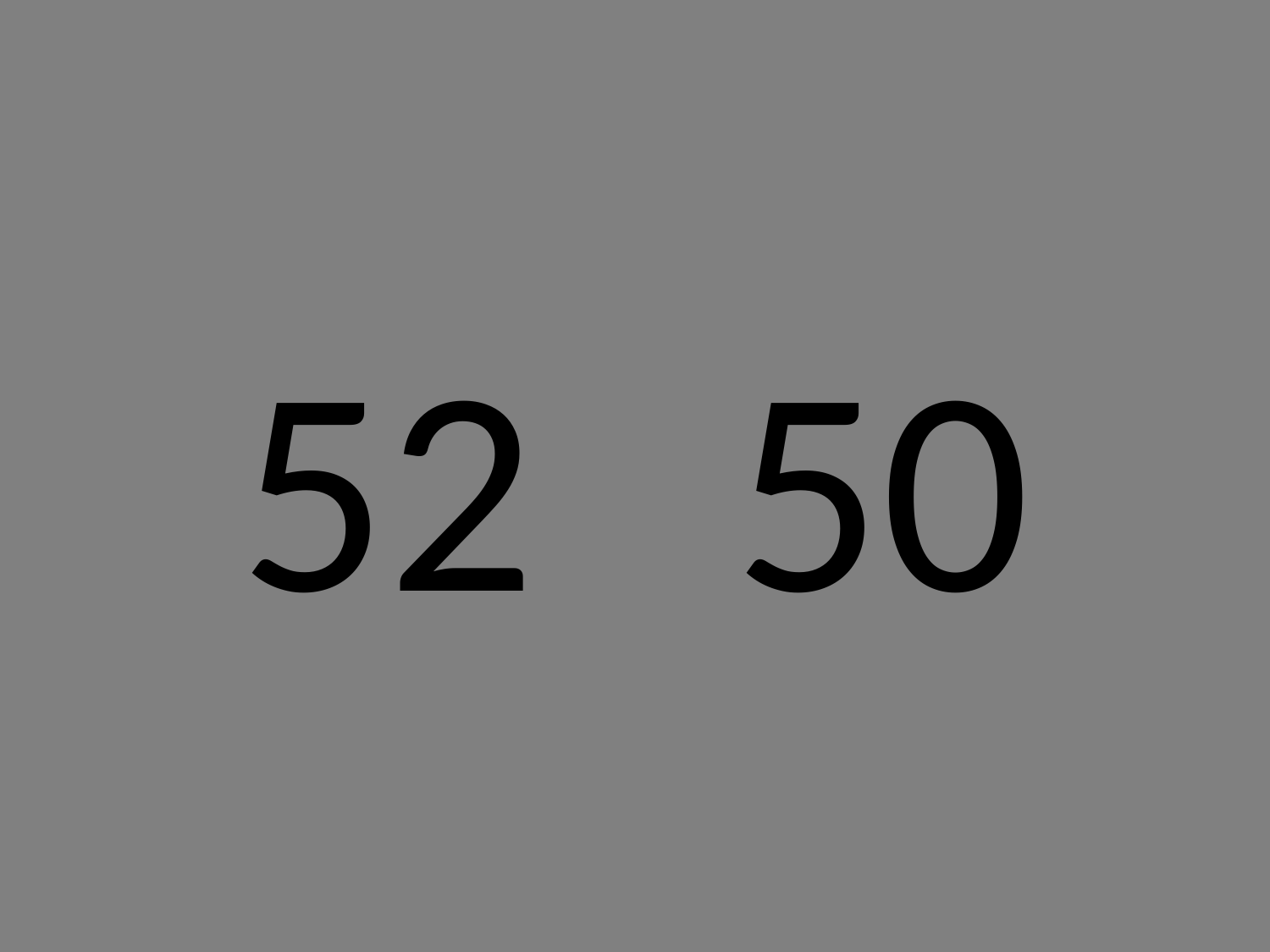

52 50

## Slide 82
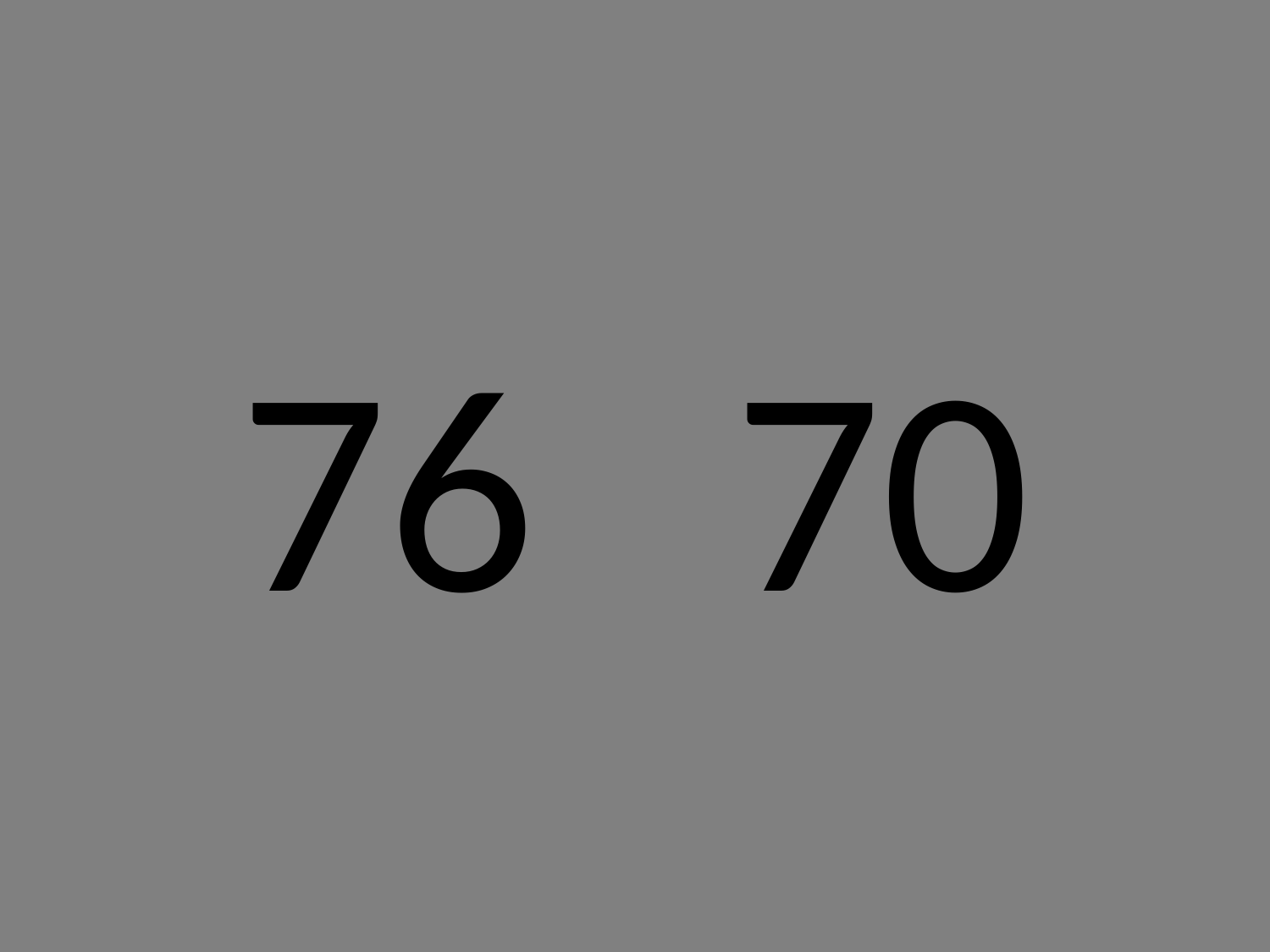

76 70

## Slide 83
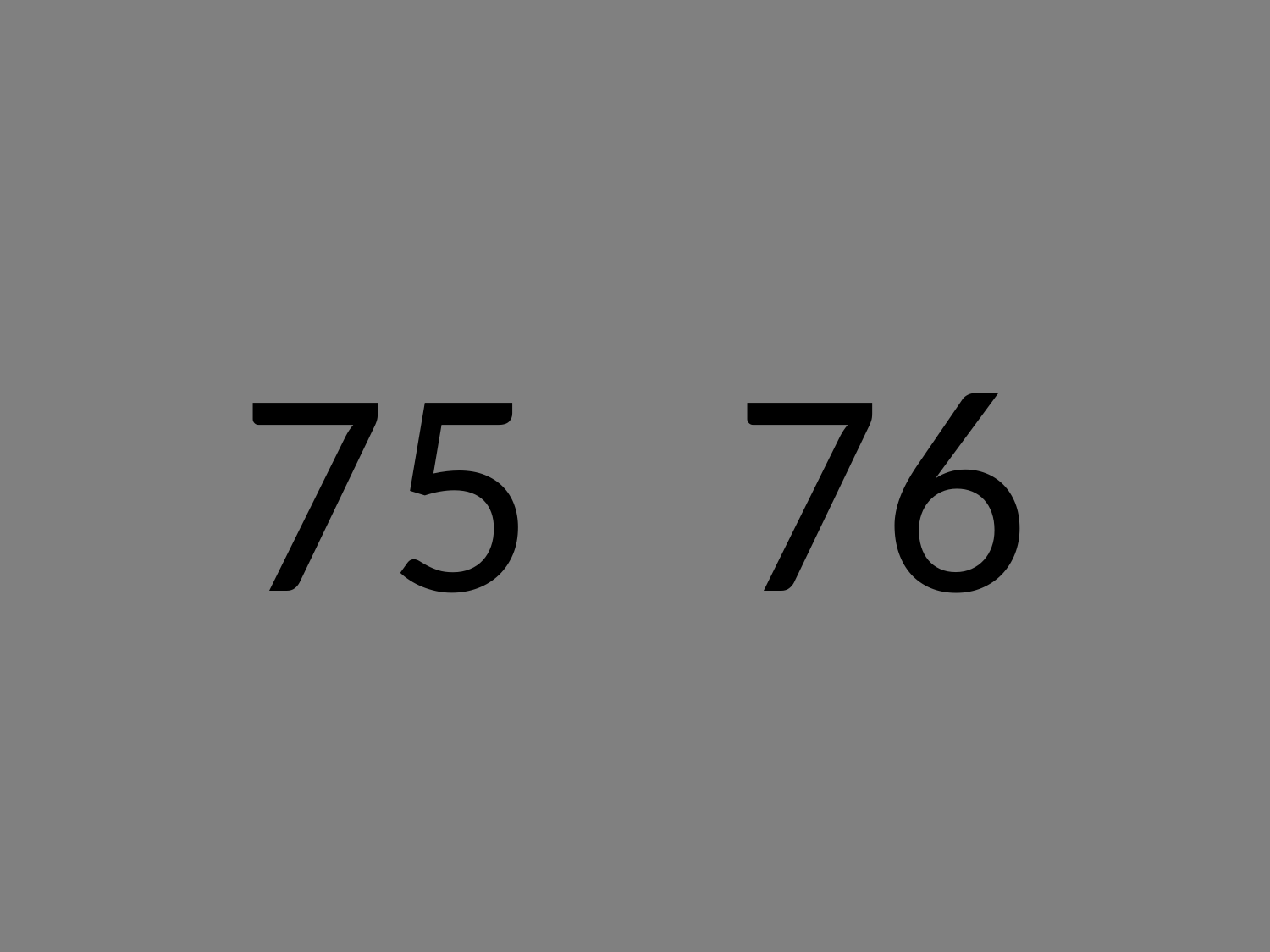

75 76

## Slide 84
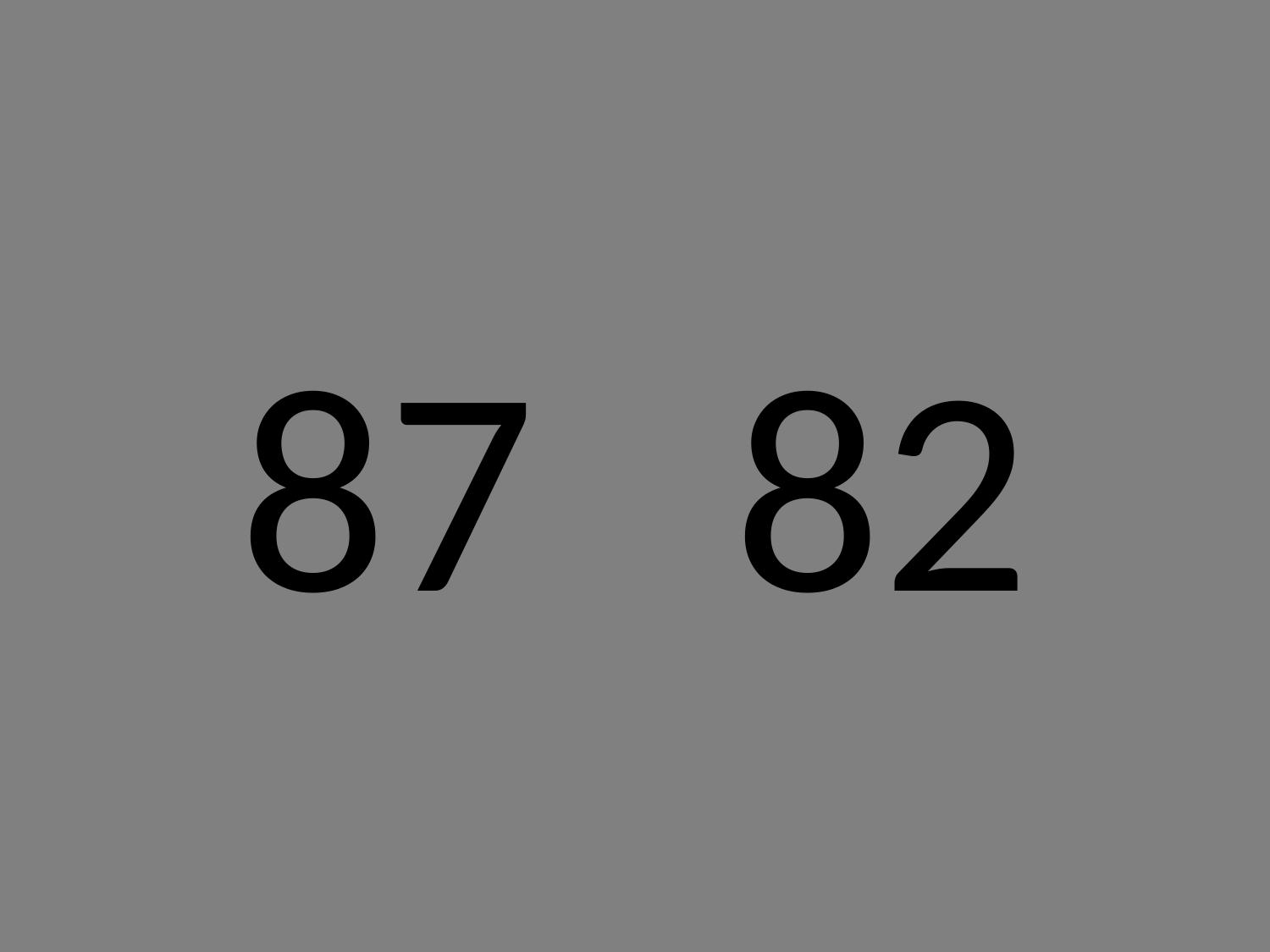

87 82

## Slide 85
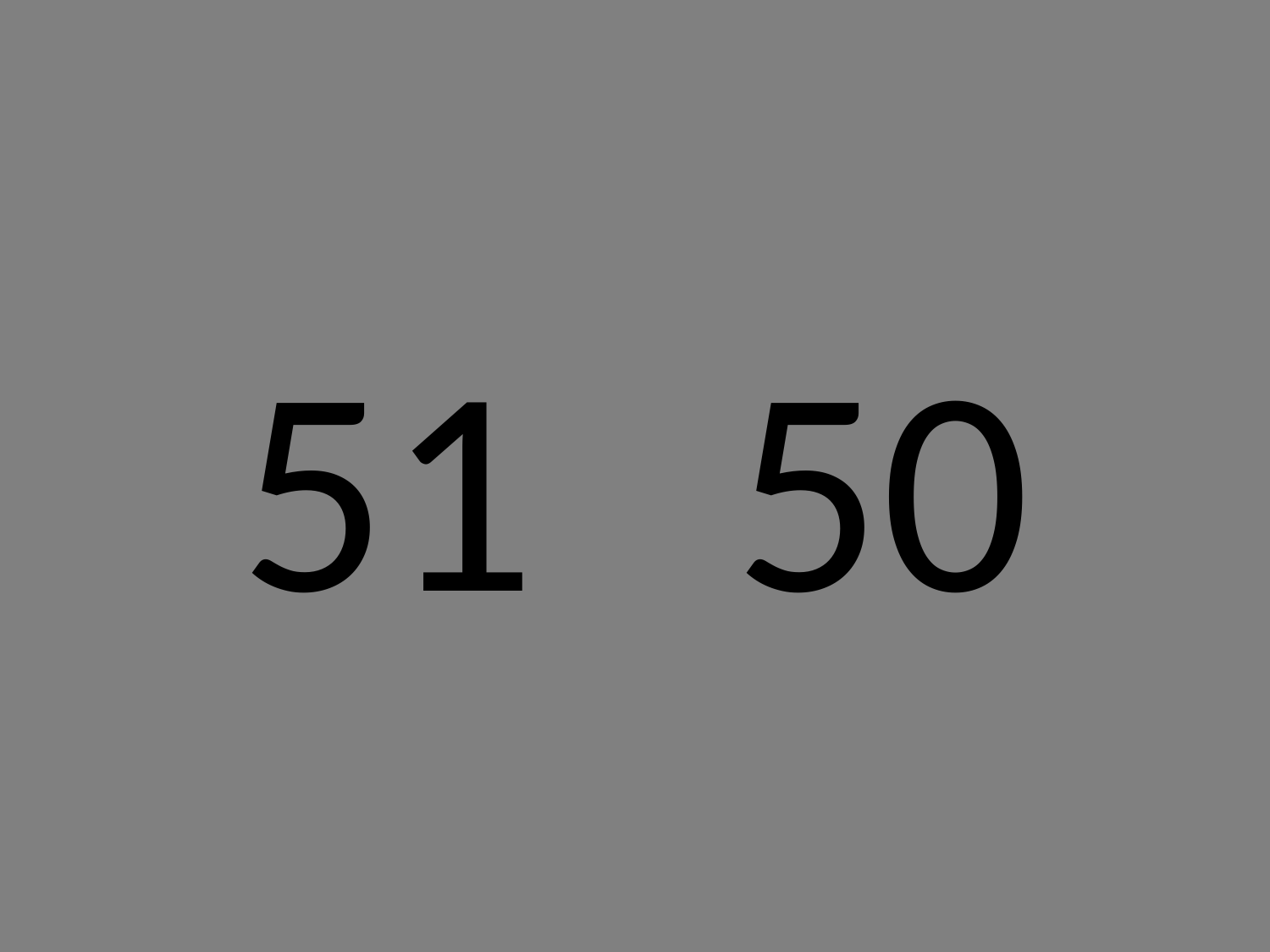

51 50

## Slide 86
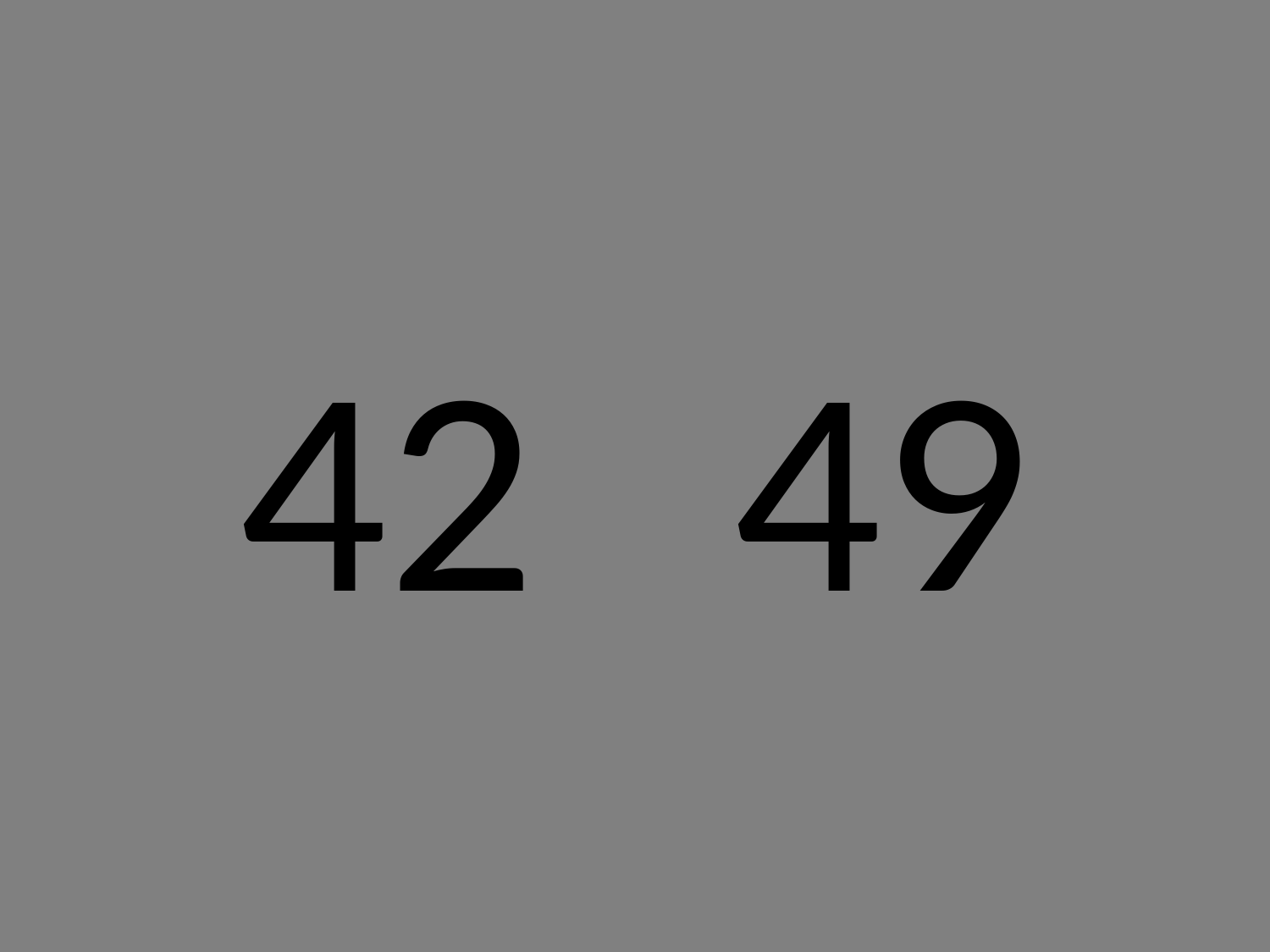

42 49

## Slide 87
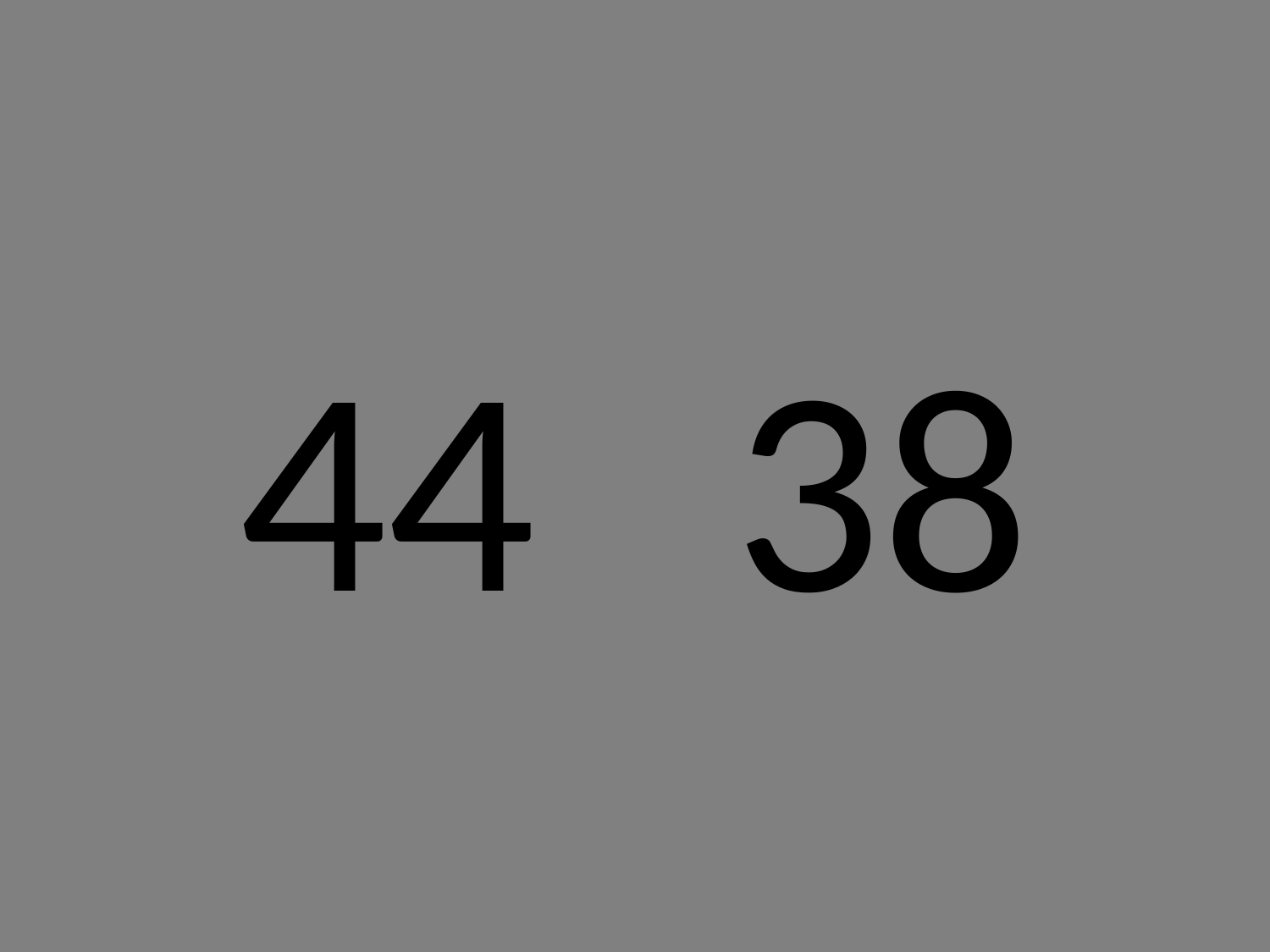

44 38

## Slide 88
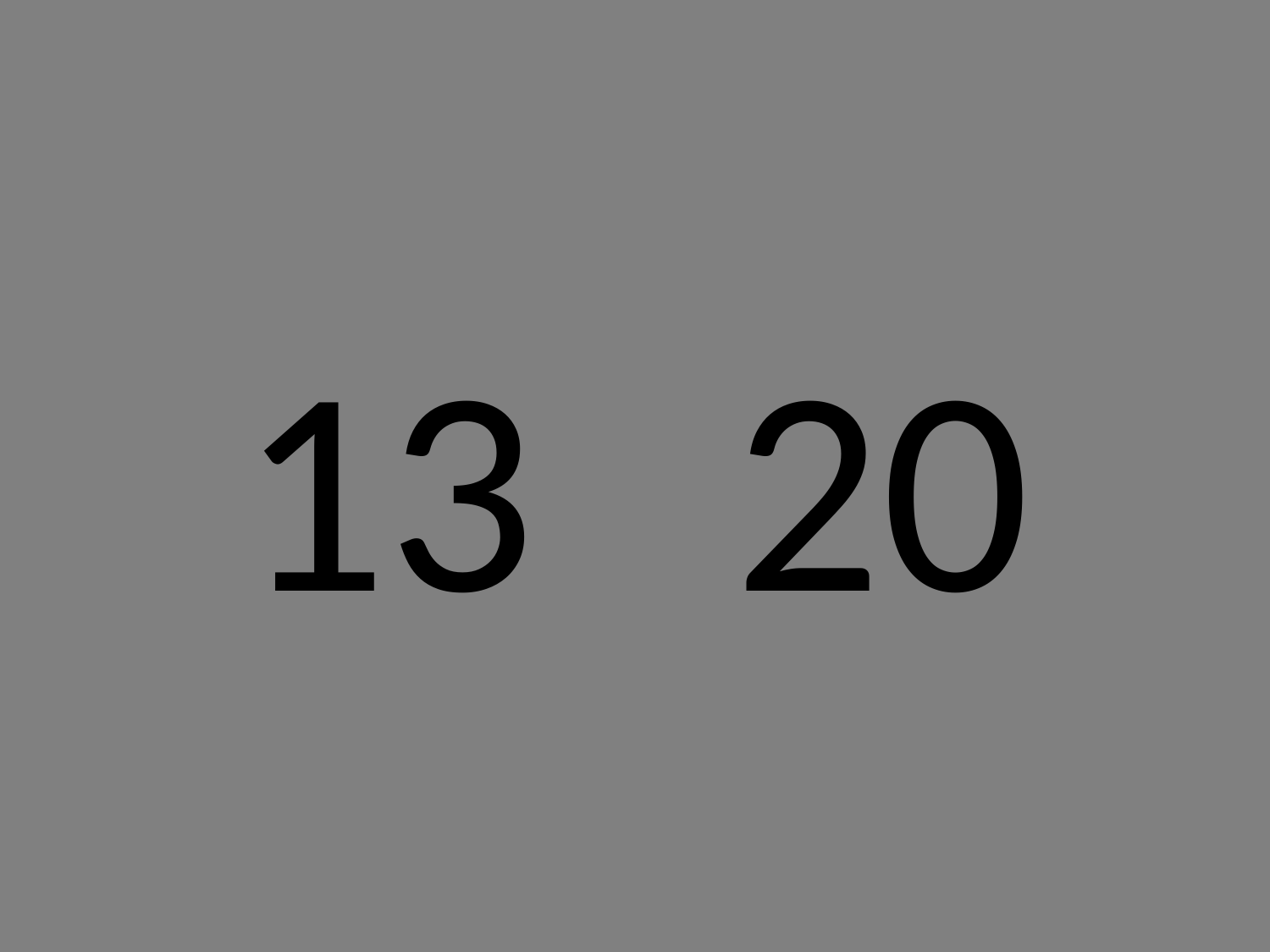

13 20

## Slide 89
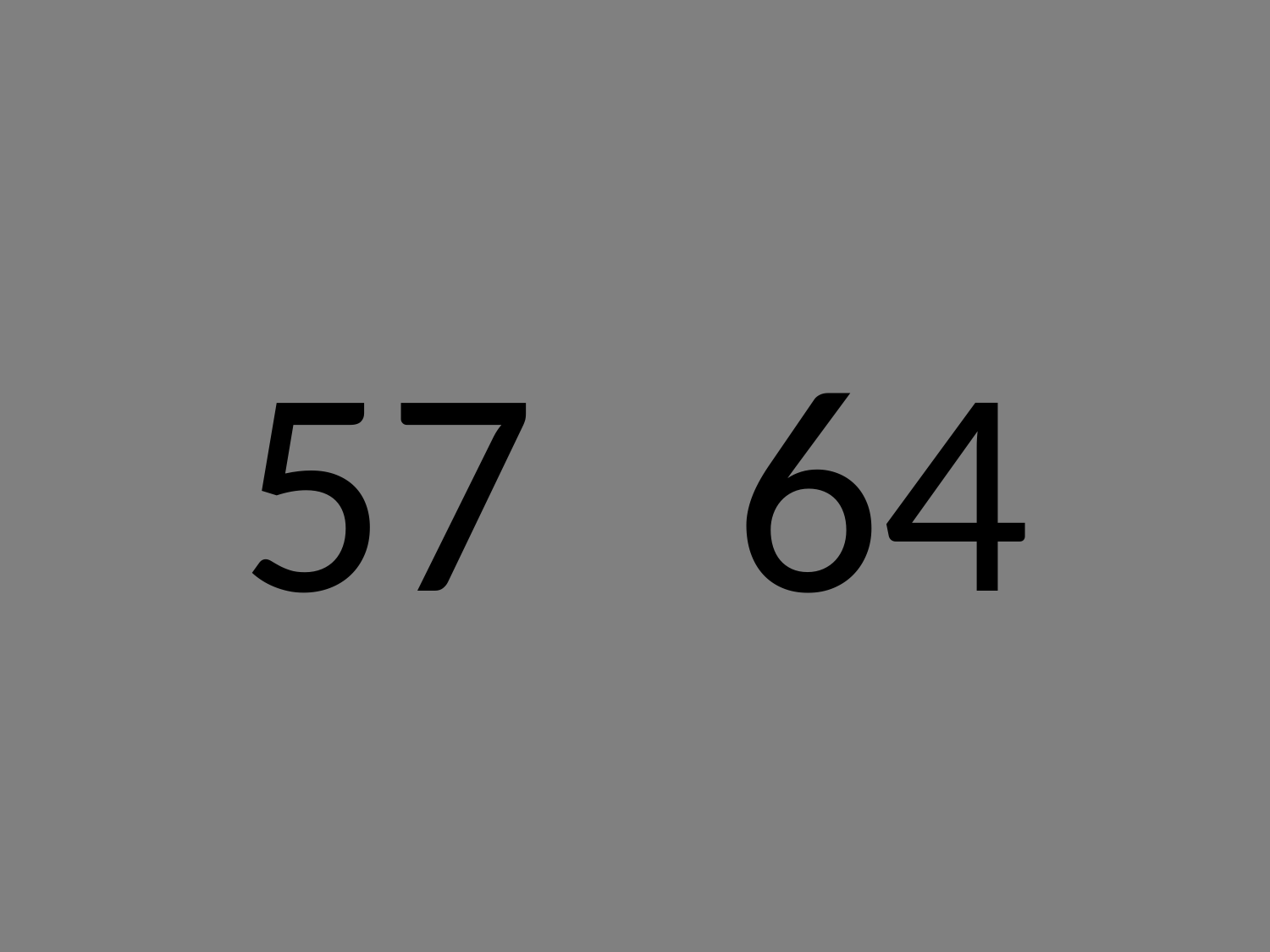

57 64

## Slide 90
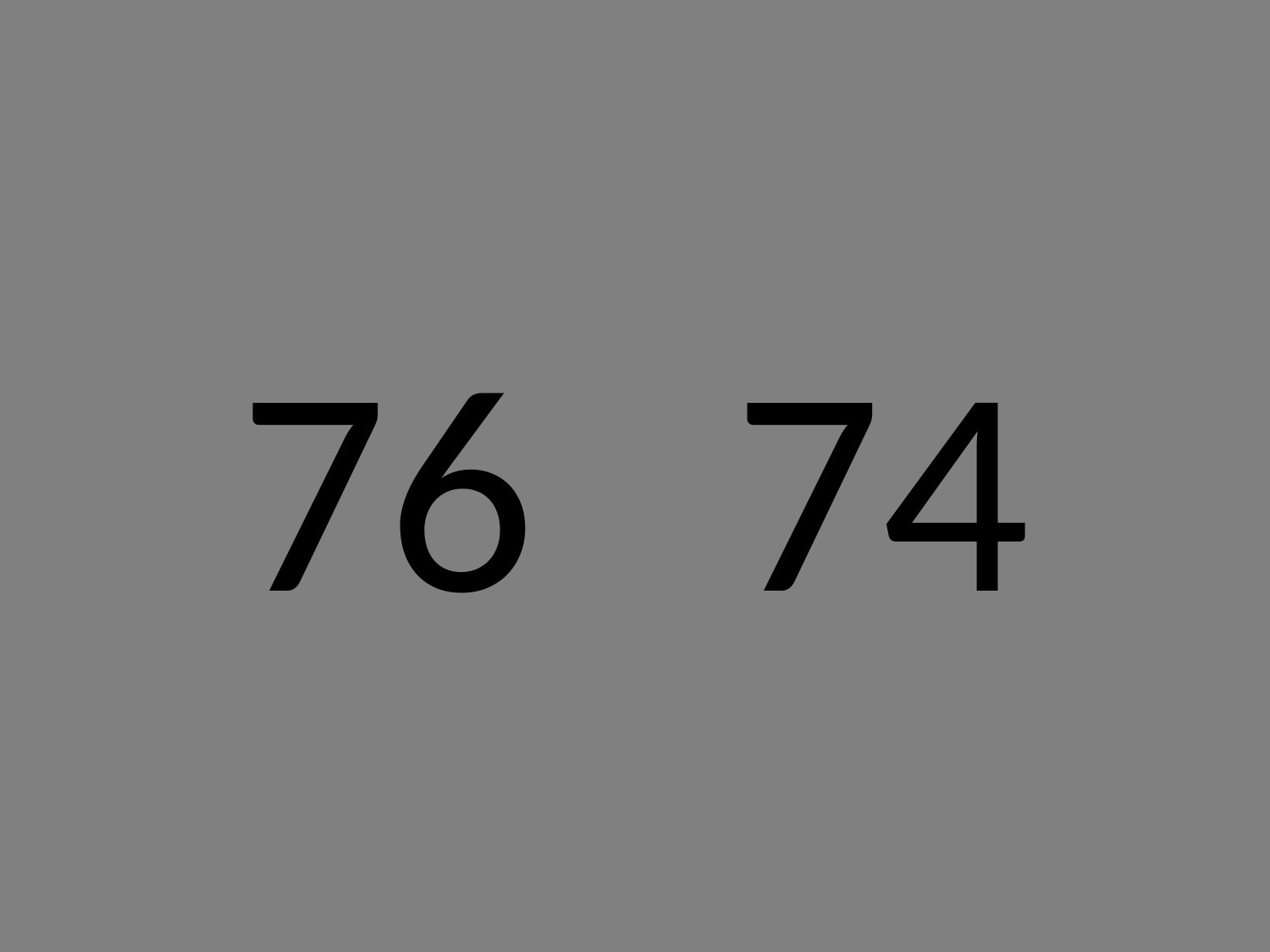

76 74

## Slide 91
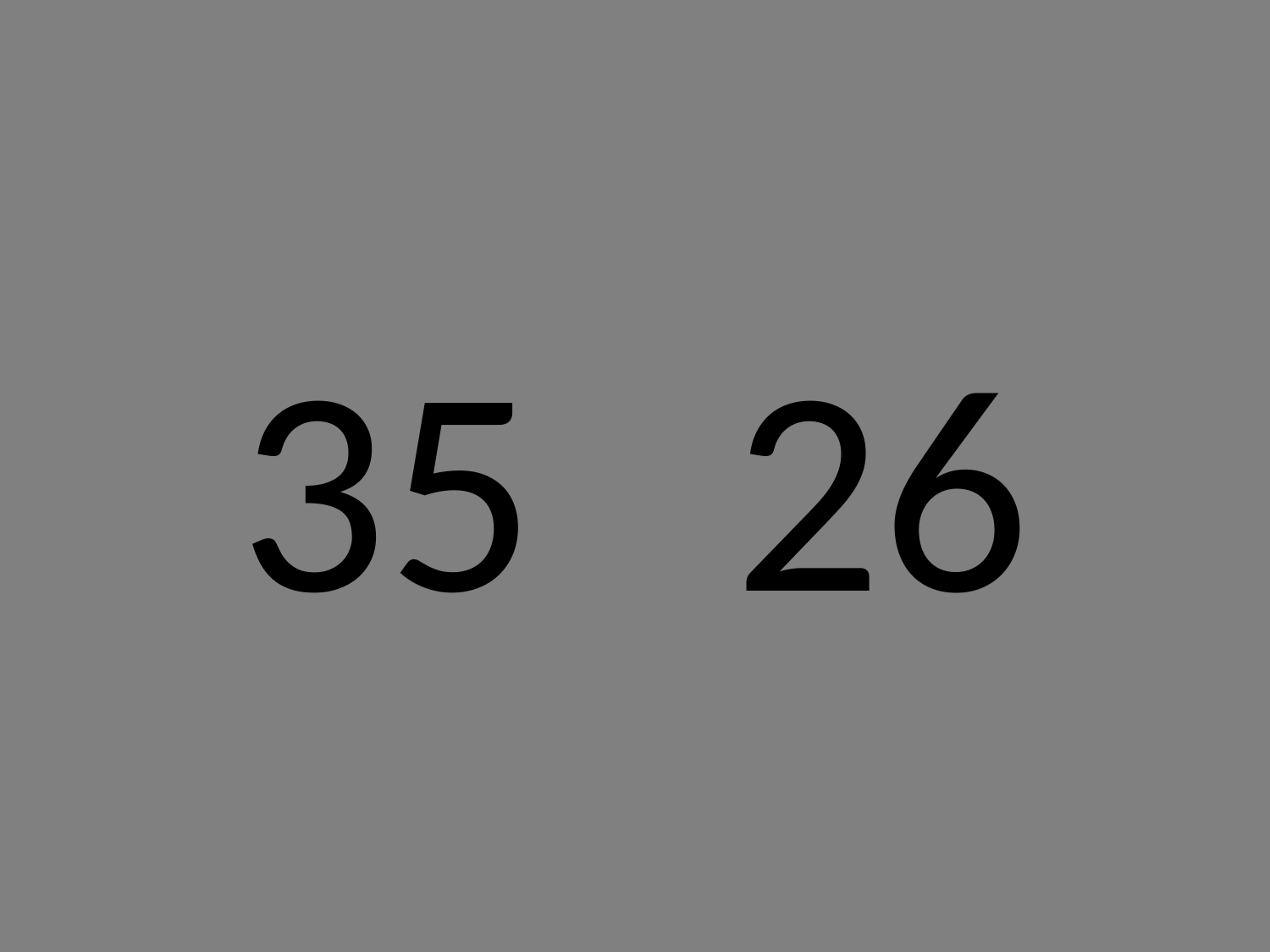

35 26

## Slide 92
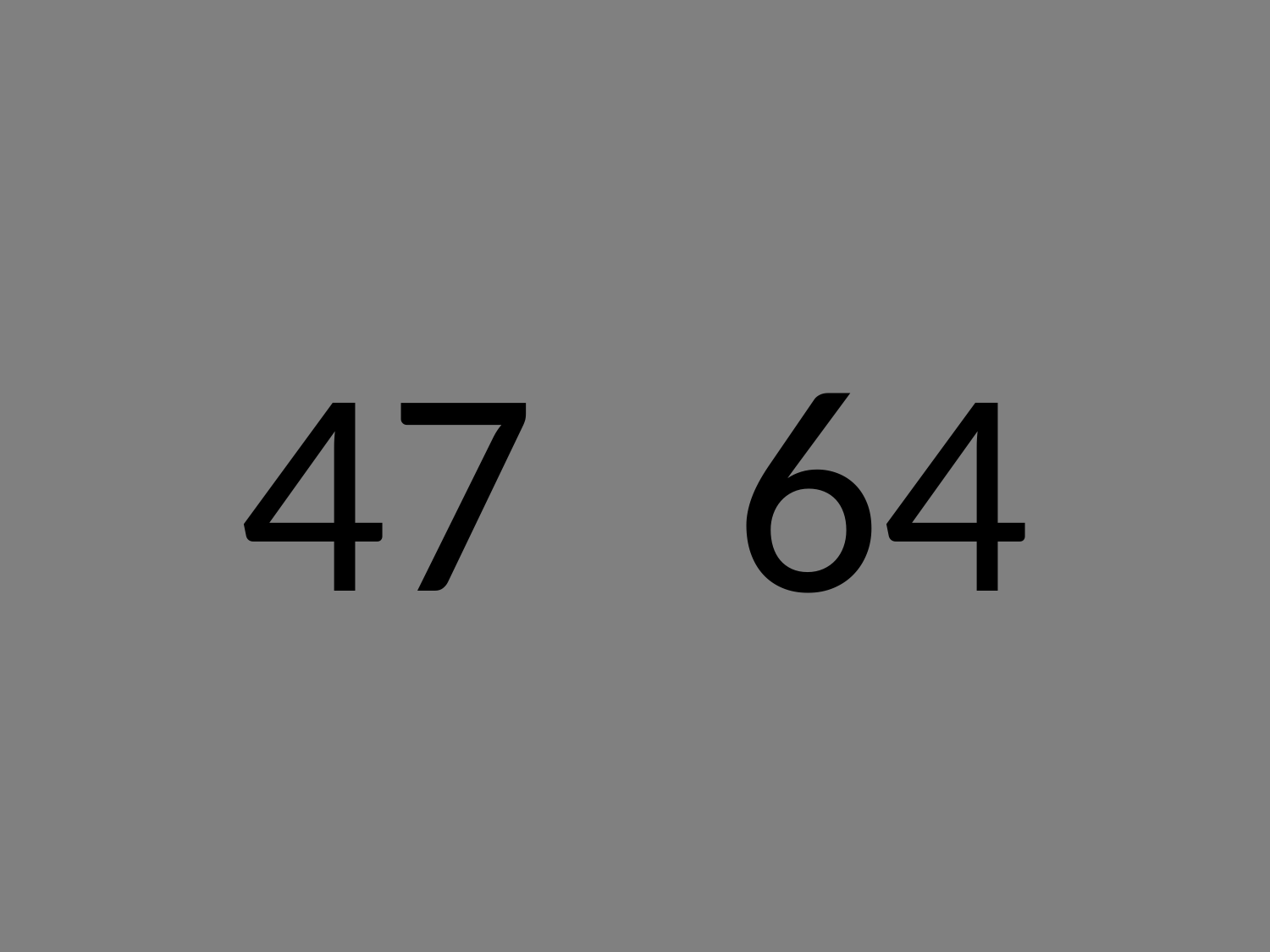

47 64

## Slide 93
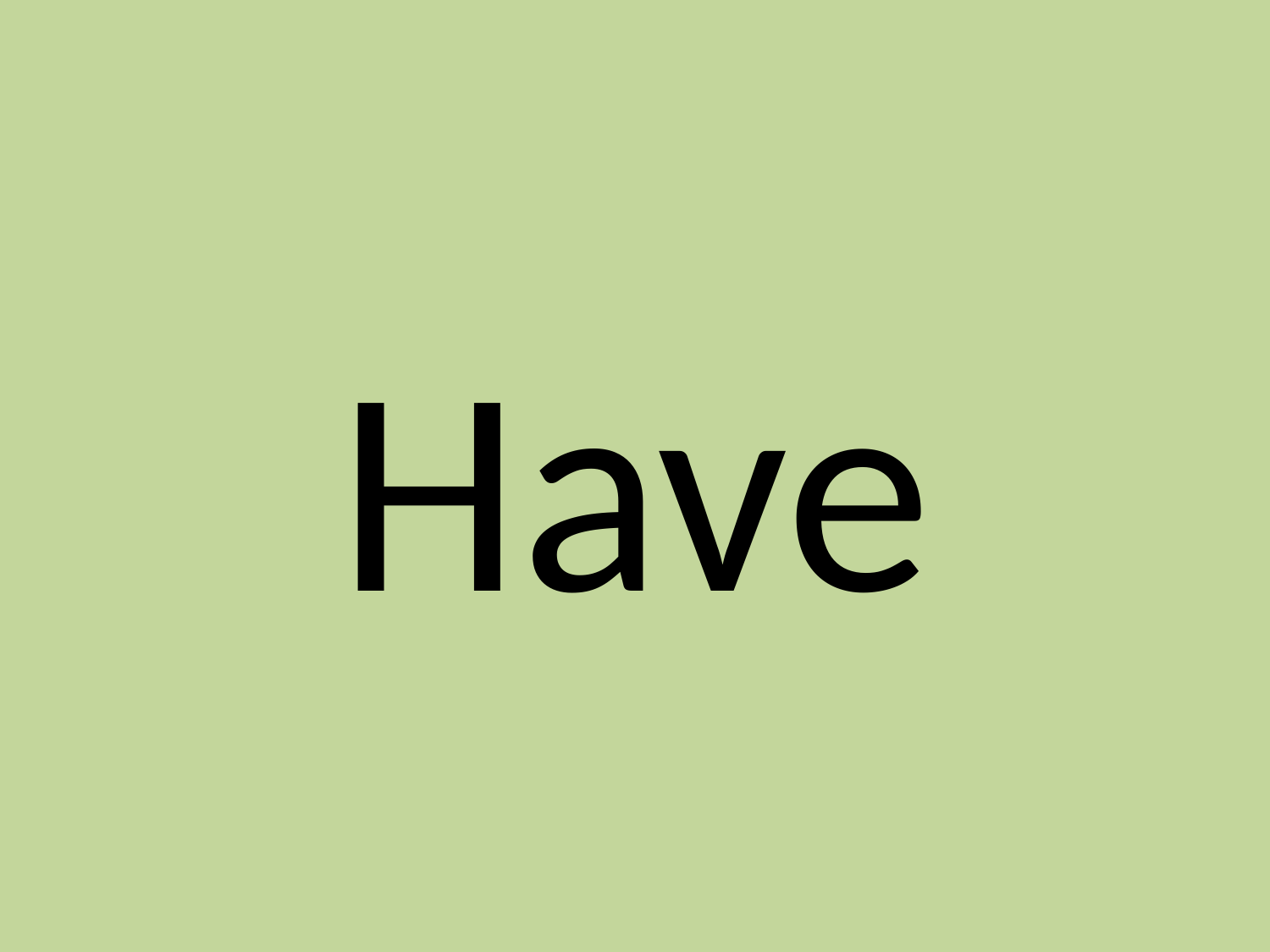

Have

## Slide 94
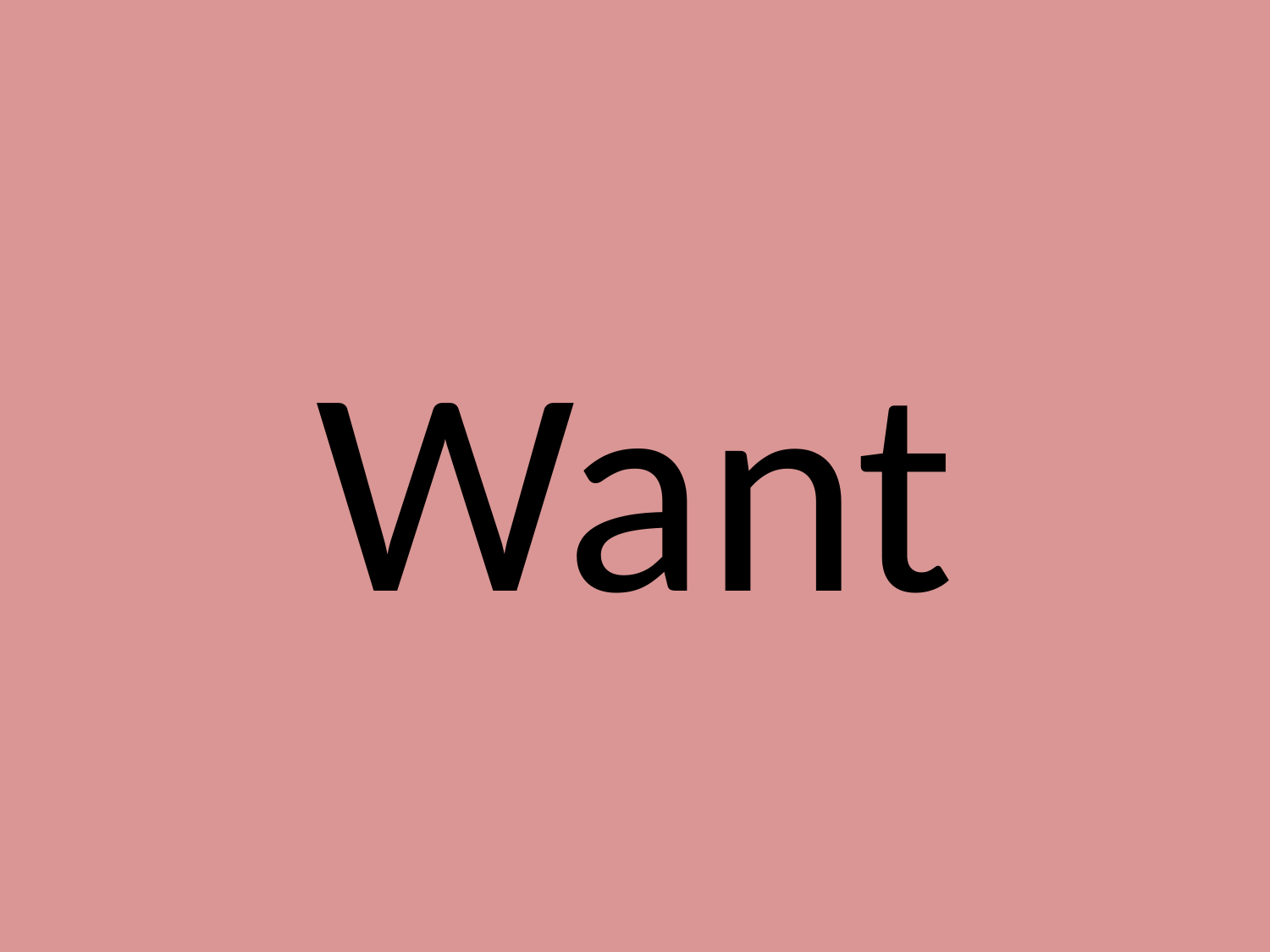

Want

## Slide 95
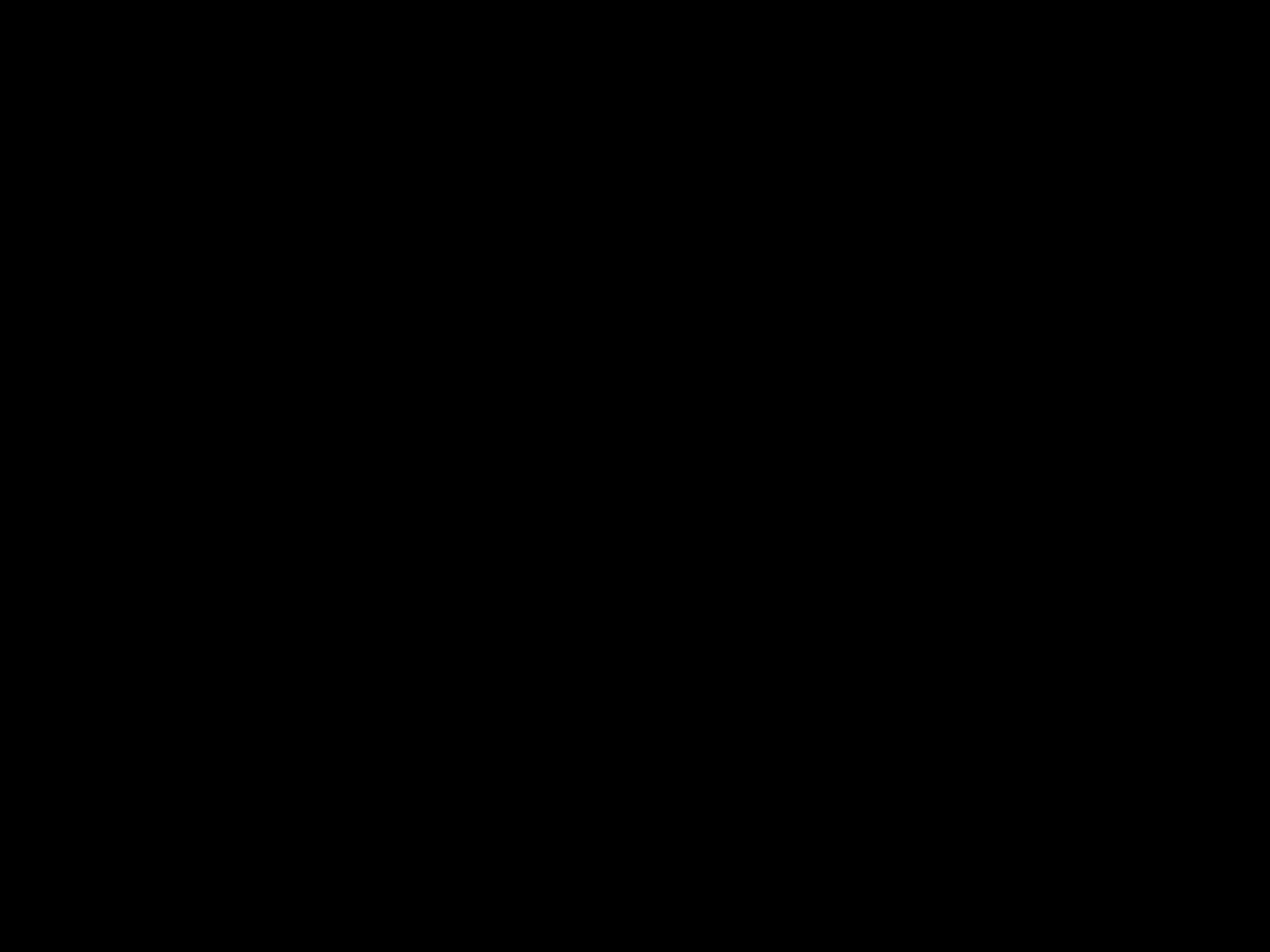

Supplement: S2 File — (ZIP) [file pone.0257717.s002.zip › software/material/TaskHaveTo.pptx]
