# Supplementary material for: Detours increase local knowledge—Exploring the hidden benefits of self-control failure
Source: PLoS One. 2021 Oct 1;16(10):e0257717. doi: 10.1371/journal.pone.0257717 (PMC8486128; doi:10.1371/journal.pone.0257717)
Supplement: S2 File — (ZIP) [file pone.0257717.s002.zip › software/Preparing the experimental software.pdf]

Preparing the experimental software

This tutorial describes the usage of the experimental software described in the article “Detours increase local knowledge - Exploring the hidden benefits of self-control failure” by Wiesner, Meyer & Lindner.

Quickstart: Downloading, Installing, Starting

Note, that copyright regulations prohibit us from providing the original distracting pictures and the original video used in the study. Therefore, we provide dummy pictures and a dummy video in the “stimuli” folder to enable you to test the software. However, the number comparison stimuli are free to use and the same as in the original study. Below, we provide detailed instructions for replication.

1. The first step is to download and install the latest version of PsychoPy on your computer. We have tested the software using PsychoPy v2021.1.2. You can download PsychoPy here: https://www.psychopy.org/index.html
2. Please unpack the zip file containing this document and the experimental software to a folder on your computer. PsychoPy will be unable to find the stimulus files in zipped folders.
3. You can start the experiment by double-clicking the file “HaveToWantTo_eng.psyexp“ and then hitting the green “play”-button in PsychoPy. The data will be saved in the folder “data”.

Warning! Calibration required!

If you want to replicate our study, it is important to keep in mind that every hardware configuration, every room and lighting, and every sample of participants is different. Therefore, you will have to calibrate the paradigm to obtain valid data.

The paradigm makes several assumptions that have to be taken care of by you.

1. Make sure all participants have normal or corrected to **normal vision**. Don’t forget to ask for color blindness and strabism.
2. Adjust the **monitor size and distance** of the participant from the monitor. The participant should be able to read the instructions comfortably. However, it is important to keep the vertical visual angle as small as possible so that both stimuli (top, bottom) can be seen without eye movement (vertical saccades).
3. Adjust the **difficulty of the number comparison task**. The aim is to keep all reaction times longer than 500 ms so that all pictures are presented for exactly 500 ms before the trial ends. To increase the difficulty of the task you could use longer numbers or different comparison criteria. A very hard variant would be to ask the participants to calculate and compare the checksums of both numbers. The least good option would be to decrease visibility (lower contrast, smaller font size).

What is what?

The zip-file contains the following files and folders:

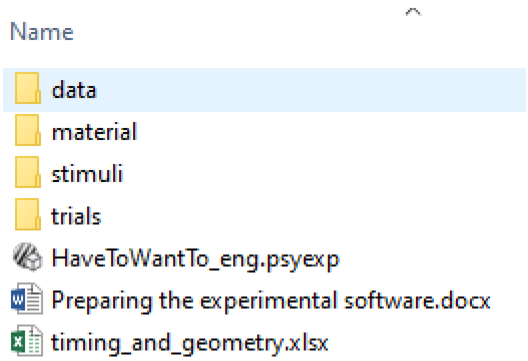

We used the following “terminology”: Pictures, instruction files, and Excel sheets related to the number comparison task have the prefix **HaveTo**. Pictures, instruction files, and Excel sheets related to the picture recognition task have the prefix **WantTo**.

- The file HaveToWantTo_eng.psyexp is the actual PsychoPy experiment, where you have to make all your adjustments to your hardware settings and picture stimuli.
- The folder “stimuli” contains “neutral” and “positive” dummy pictures, the number comparison stimuli, all instruction slides, and a dummy video. Please replace the dummy pictures and the dummy video with your own versions!
- The folder “trials” contains triallists that are read by PsychoPy at the beginning of the experiment. We do not recommend changing these lists.
- The folder “material” contains PowerPoint files that we used to produce the instruction slides. You can make changes to these files as needed and export the slides as jpg-files.
- In the directory "data" PsychoPy will write the results of an experimental run

Adjusting the software to your hardware

Why do you need to adjust the software to your hardware? Every hardware configuration and every operating system has specific requirements. The software will most likely run irrespective of the configuration you use. However, to obtain precise timing and sharp images, adjustments are strongly recommended. To adapt the software to your computer you will need basic knowledge of PsychoPy. Using the so-called "builder view" in PsychoPy will be sufficient. Python programming skills are useful but not required.

We conducted the experiment using notebooks with Intel i5 processors, 8GB RAM, nvidia graphics cards, and Full HD monitors running on 60 Hz refresh rate. The paradigm is not very resource-hungry. You should be

fine as long as you have a dedicated graphics card. On-board (integrated) graphics cards are not recommended. In the original software, we specified all presentation times and durations in frames. For example, a duration of 0.5 sec equals 30 frames presented for 1/60 sec each. We recommend that you adjust the timing in the PsychoPy experiment file to the frame rate of your computer to obtain the best precision. In this zip file, we provide an Excel sheet with all durations and times specified in frames and seconds.

The software has only been tested on computers running Windows 10 education. However, it should also run on macOS or Linux if you change all pathnames in the PsychoPy experiment file from e.g. “$'stimuli/'+FirstTop” to “$'stimuli\'+FirstTop”.

Inserting your pictures!

Due to copyright restrictions, we are not allowed to distribute the original pictures we have used in the experiment. However, the pictures are described in

> Wiesner CD, Lindner C. Weakening self-control biases the emotional evaluation of appetitive cues. PLoS ONE 2017;12(1):e0170245.

We created dummy pictures that you will find in the subfolder stimuli. We recommend that you replace the dummy files with your jpg-files but keep the same file names. The jpg-files should have a resolution of 800 by 600 pixels and all images should be corrected to the mean luminance of all images so that each image has the same mean luminance.

> Willenbockel, V., Sadr, J., Fiset, D., Horne, G. O., Gosselin, F., & Tanaka, J. W. (2010). Controlling low-level image properties: The SHINE toolbox. Behavior Research Methods, 42(3), 671–684. http://doi.org/10.3758/BRM.42.3.671

There are different matlab toolboxes available to correct luminance, e.g. https://osf.io/auzjy/wiki/home/.

In PsychoPy you can specify in two ways how large an image should be displayed on the monitor: first, you can specify the size in proportions of the monitor height and width, e.g. an image height of 0.5 corresponds to a display at half the monitor height. Second, you can specify the image size in pixels, e.g. an image height of 600 pixels. Depending on the resolution of your monitor, the image will then be displayed larger at low resolutions or smaller at high resolutions. However, we recommend the second method, because PsychoPy then displays the images in original resolution and does not need to rescale. This way you get the sharpest image display on the monitor. In the zip file containing this document, we provide an Excel sheet with all sizes in pixels and all positions on the screen.

Good luck!
