# Supplementary material for: Detours increase local knowledge—Exploring the hidden benefits of self-control failure
Source: PLoS One. 2021 Oct 1;16(10):e0257717. doi: 10.1371/journal.pone.0257717 (PMC8486128; doi:10.1371/journal.pone.0257717)
Supplement: S2 File — (ZIP) [file pone.0257717.s002.zip › software/stimuli/stimuli_neutral.pptx]

## Slide 1
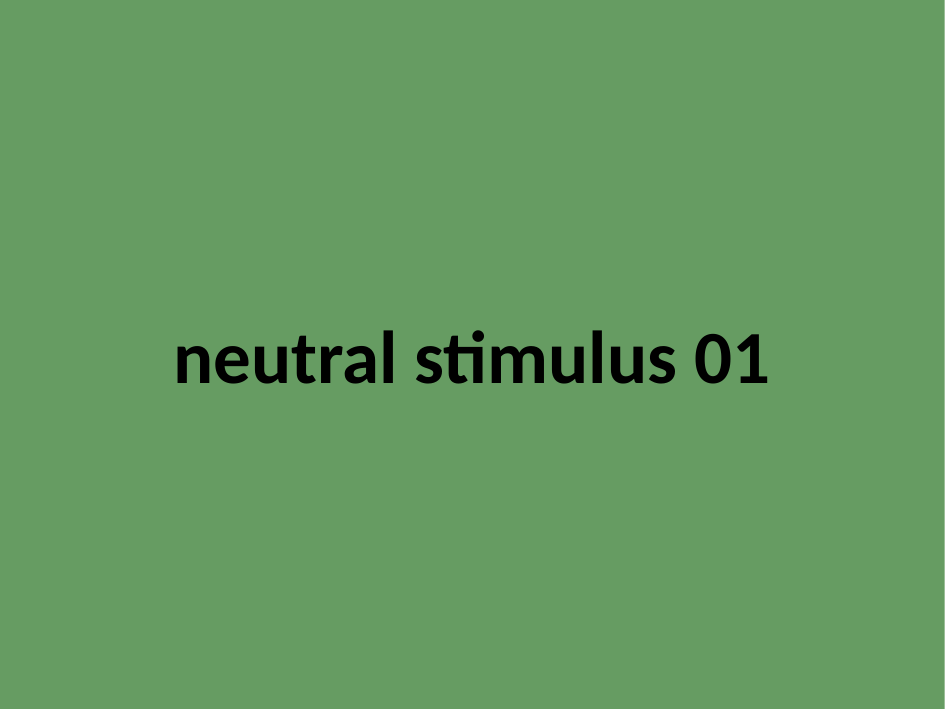

neutral stimulus 01

## Slide 2
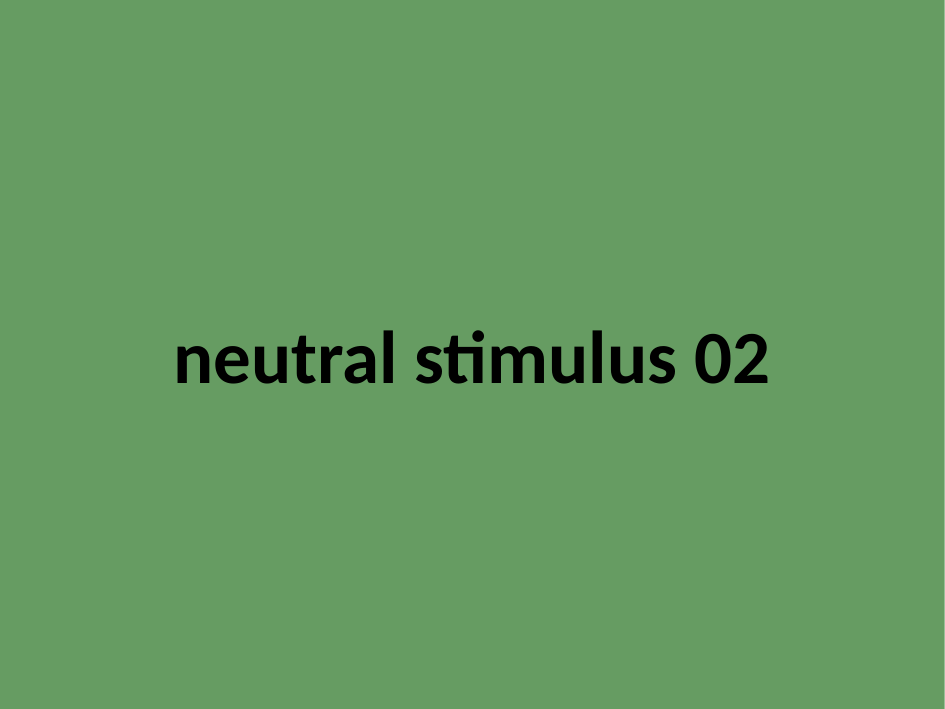

neutral stimulus 02

## Slide 3
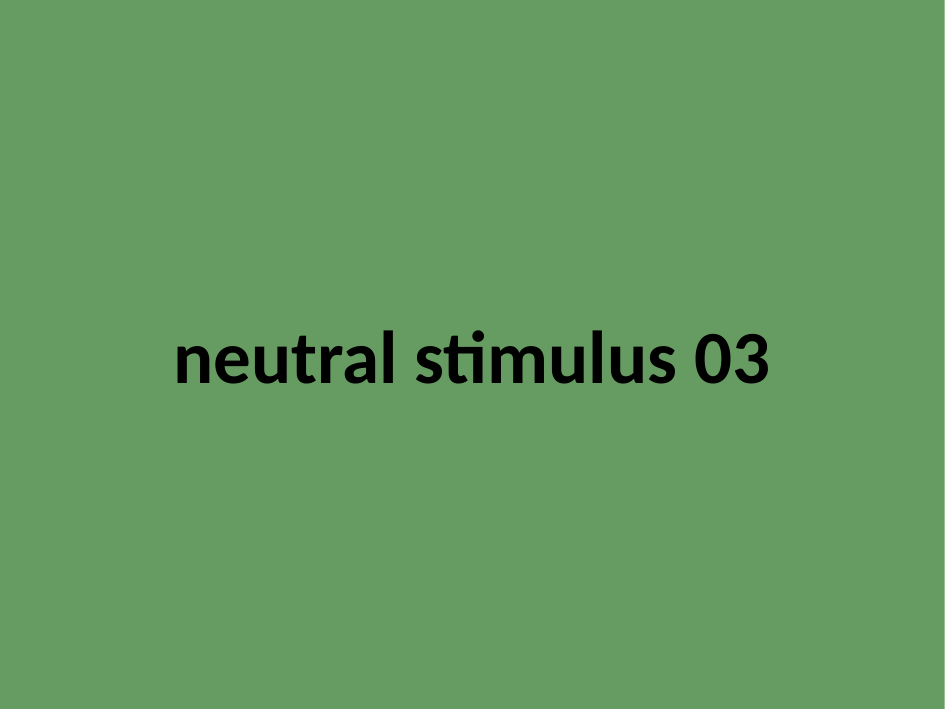

neutral stimulus 03

## Slide 4
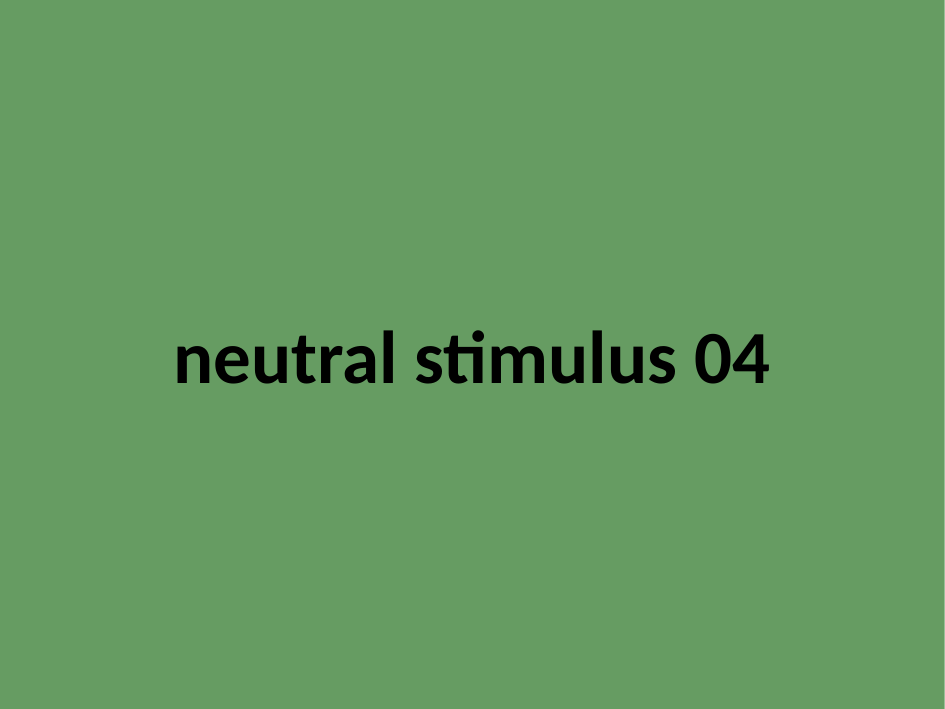

neutral stimulus 04

## Slide 5
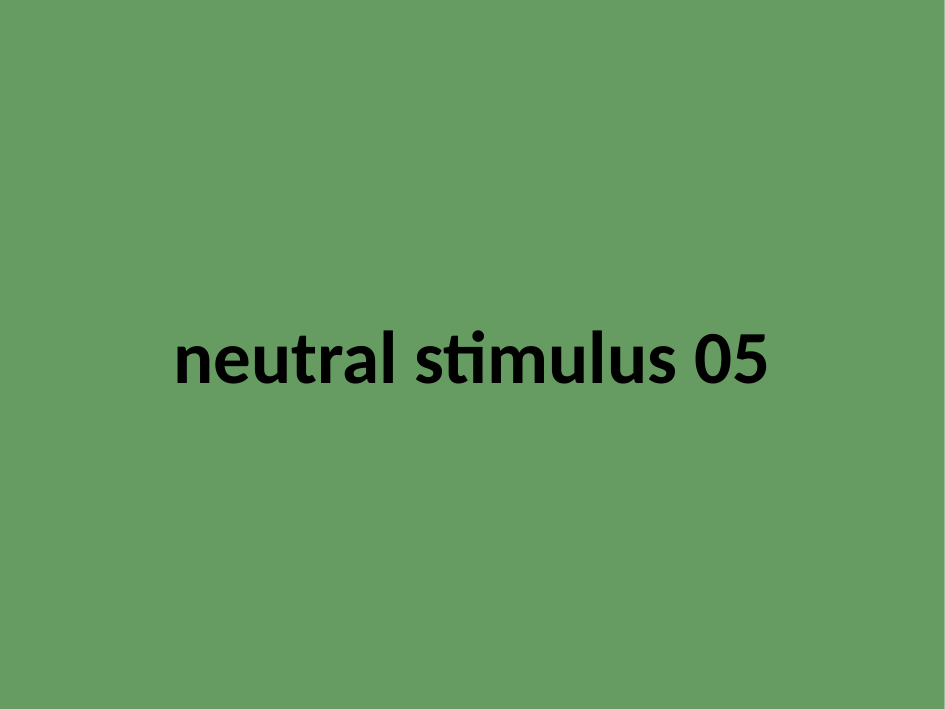

neutral stimulus 05

## Slide 6
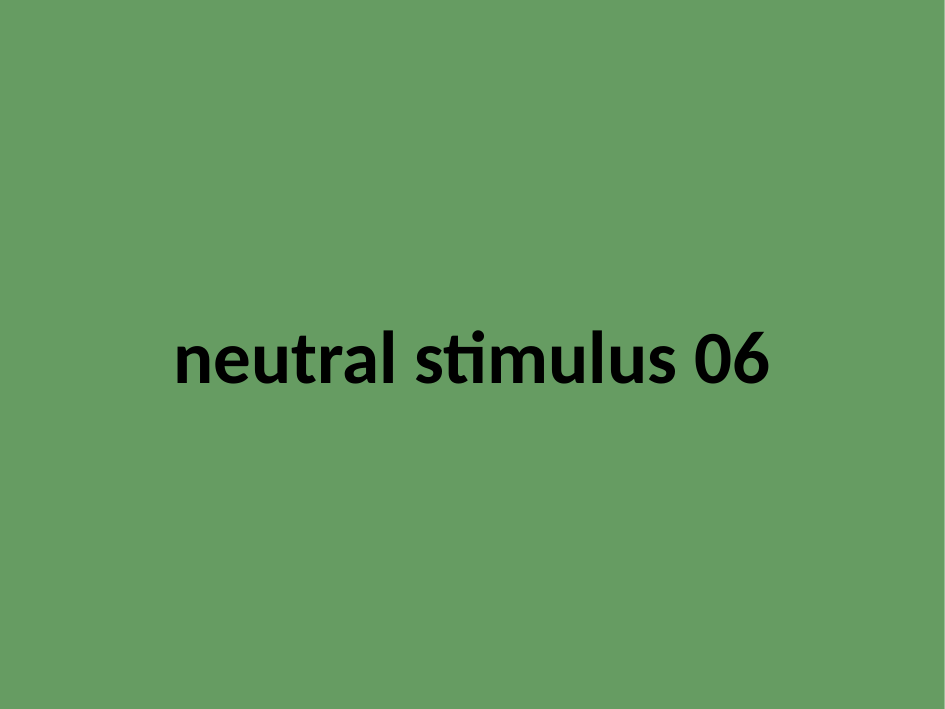

neutral stimulus 06

## Slide 7
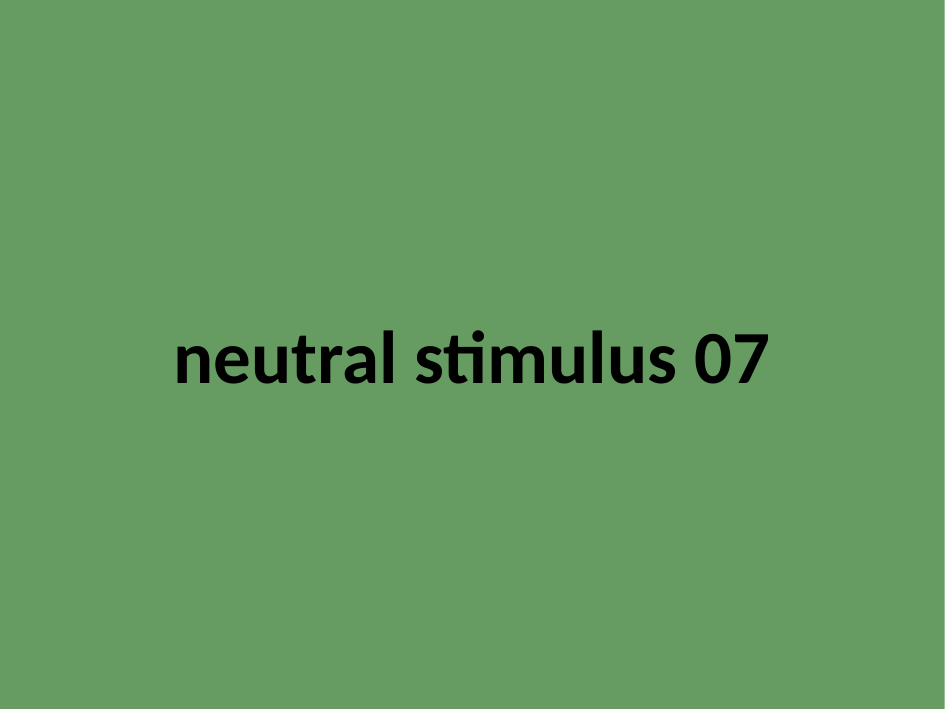

neutral stimulus 07

## Slide 8
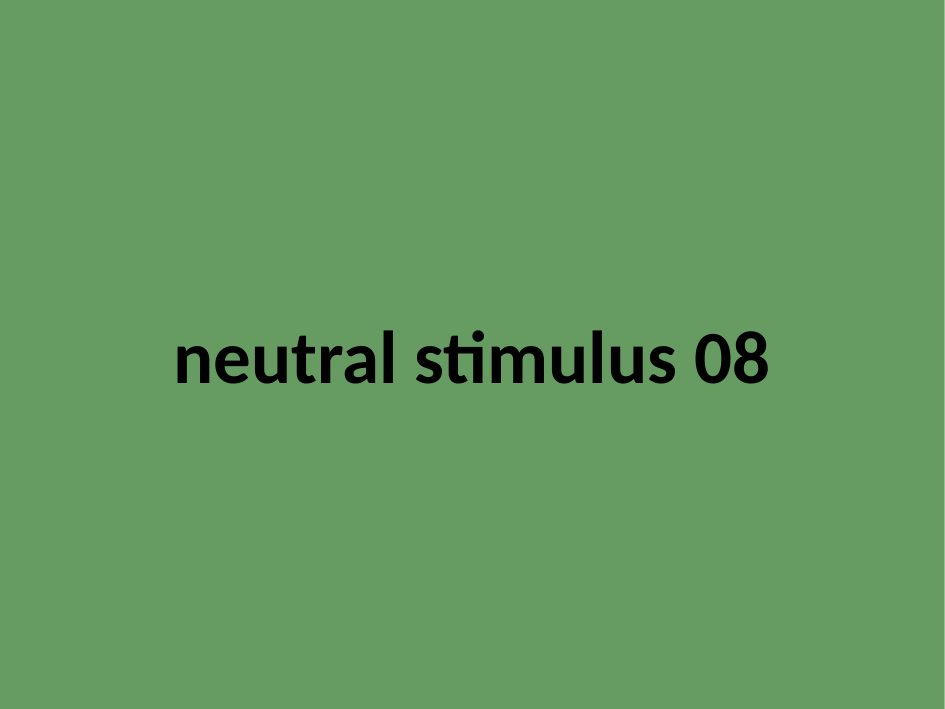

neutral stimulus 08

## Slide 9
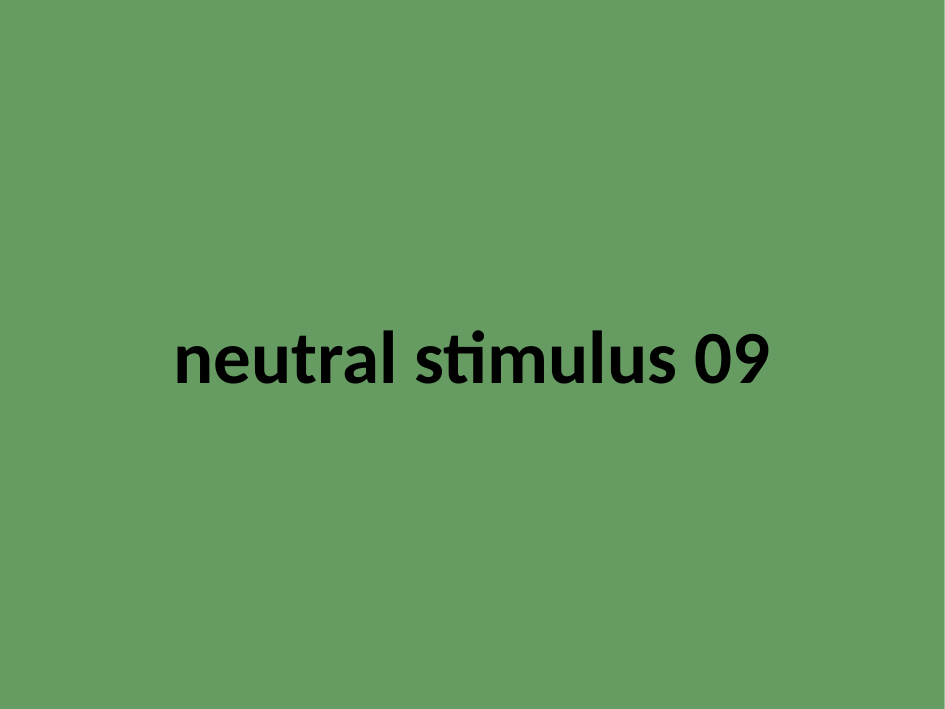

neutral stimulus 09

## Slide 10
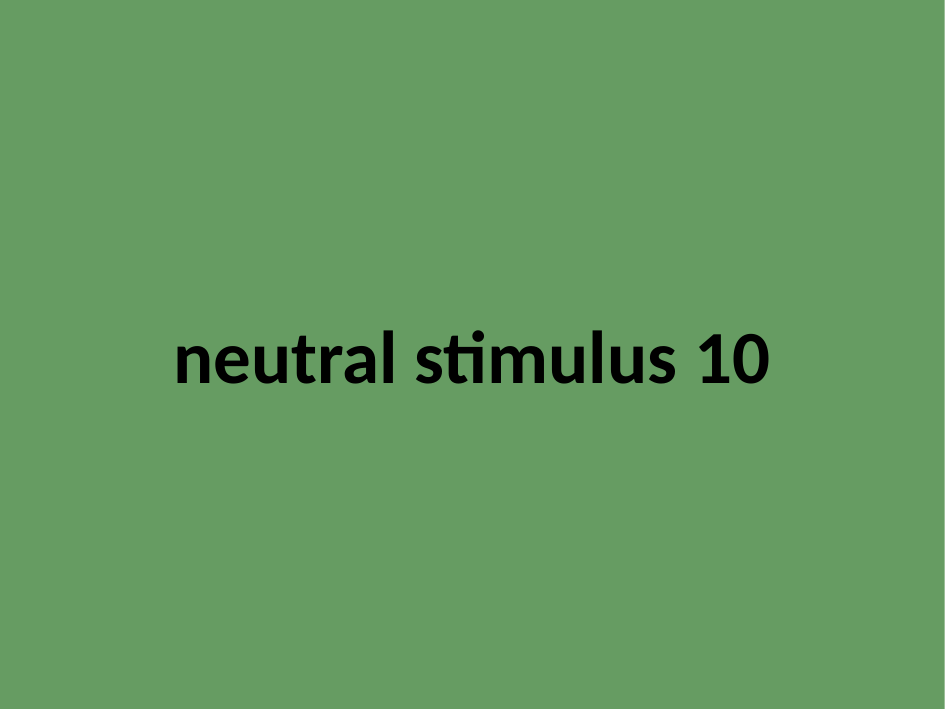

neutral stimulus 10

## Slide 11
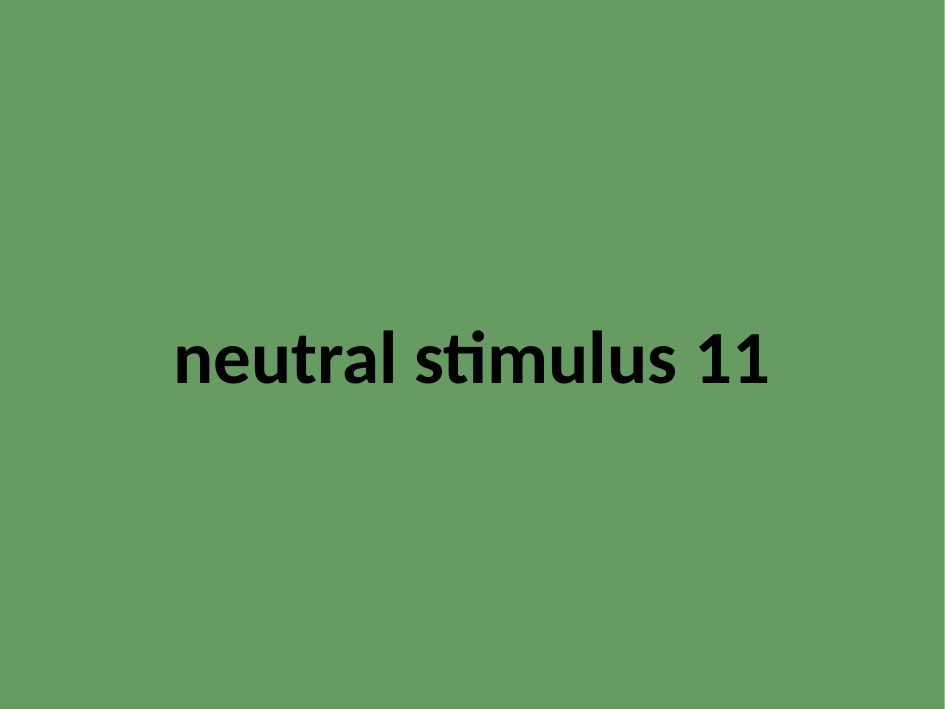

neutral stimulus 11

## Slide 12
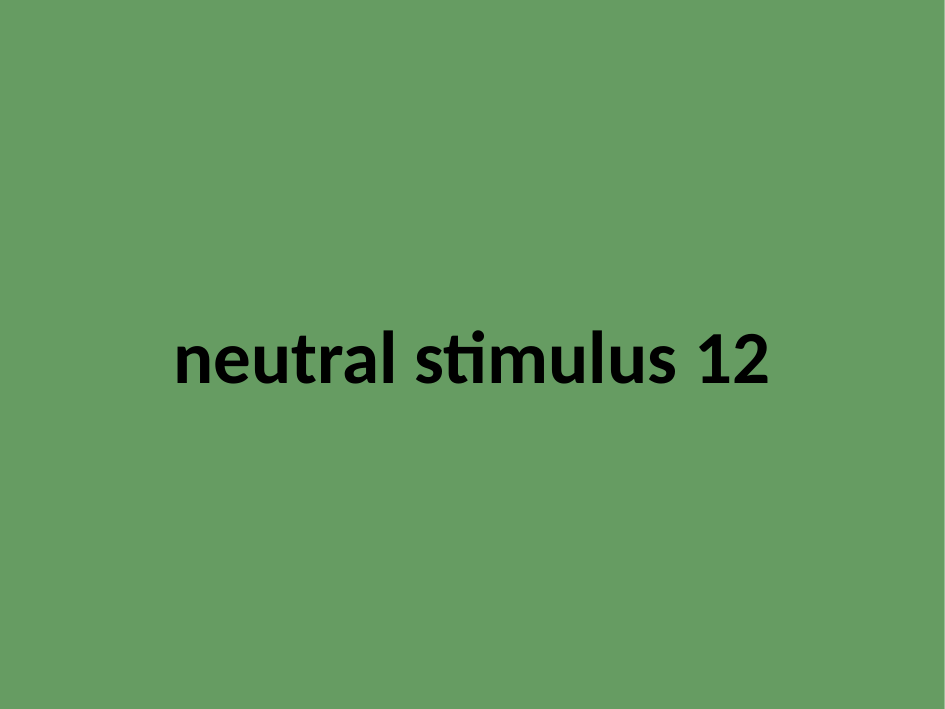

neutral stimulus 12

## Slide 13
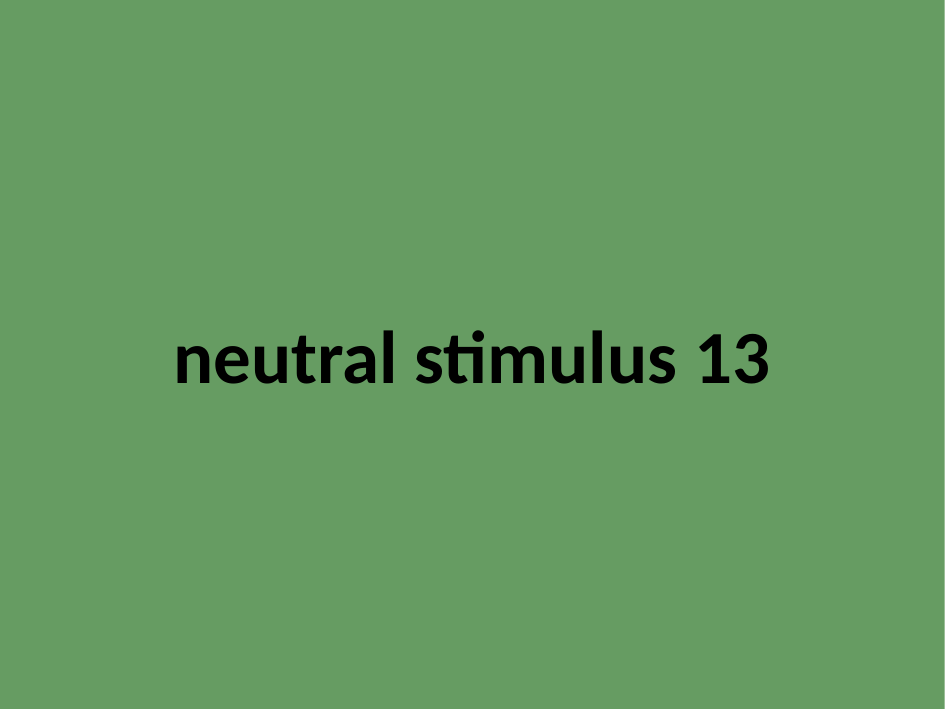

neutral stimulus 13

## Slide 14
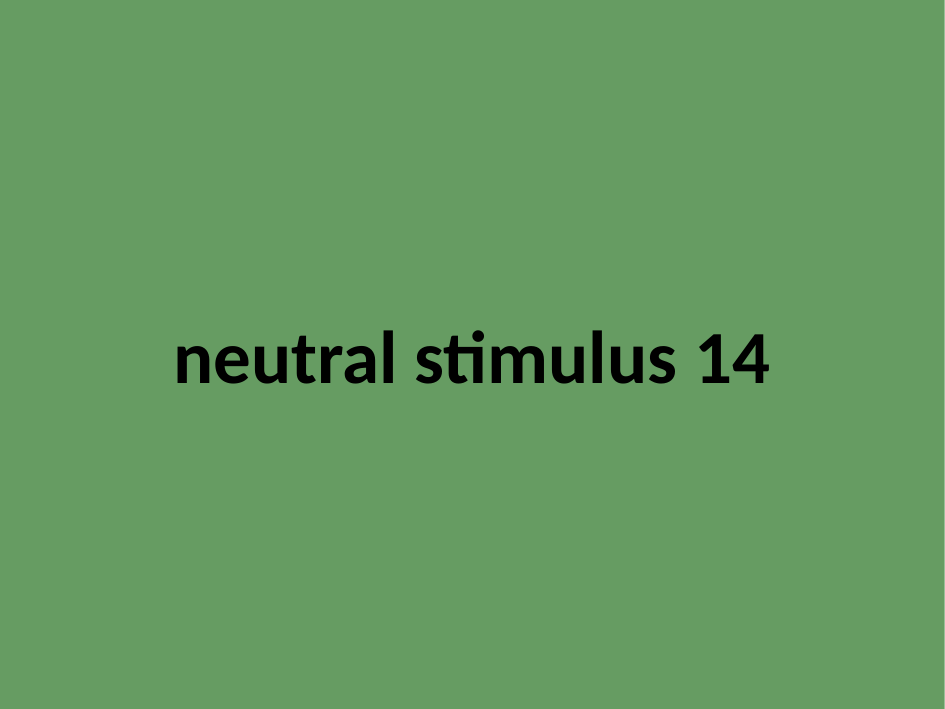

neutral stimulus 14

## Slide 15
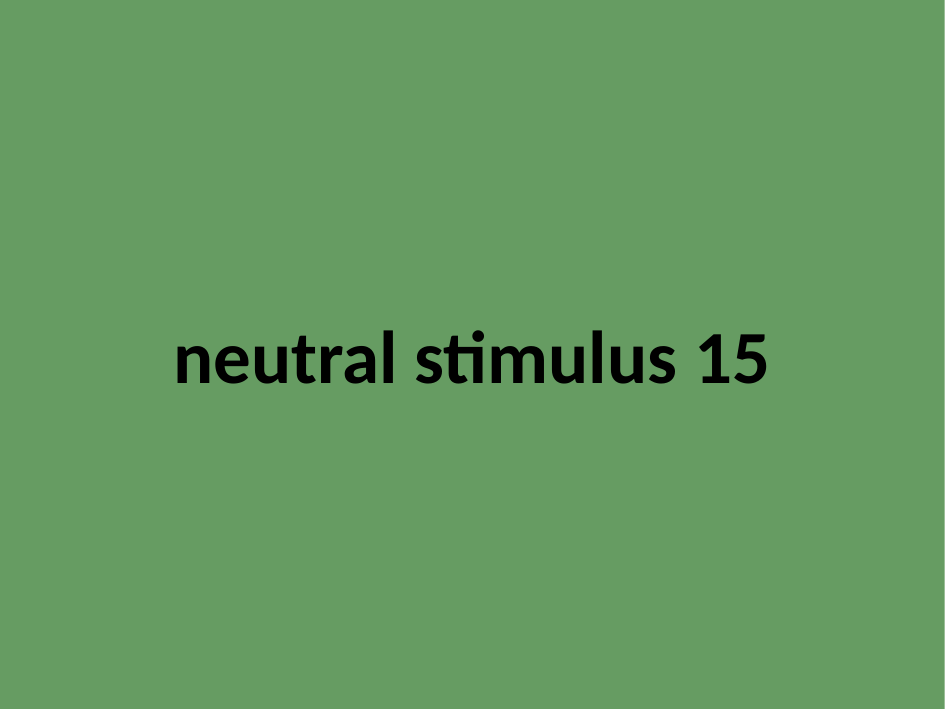

neutral stimulus 15

## Slide 16
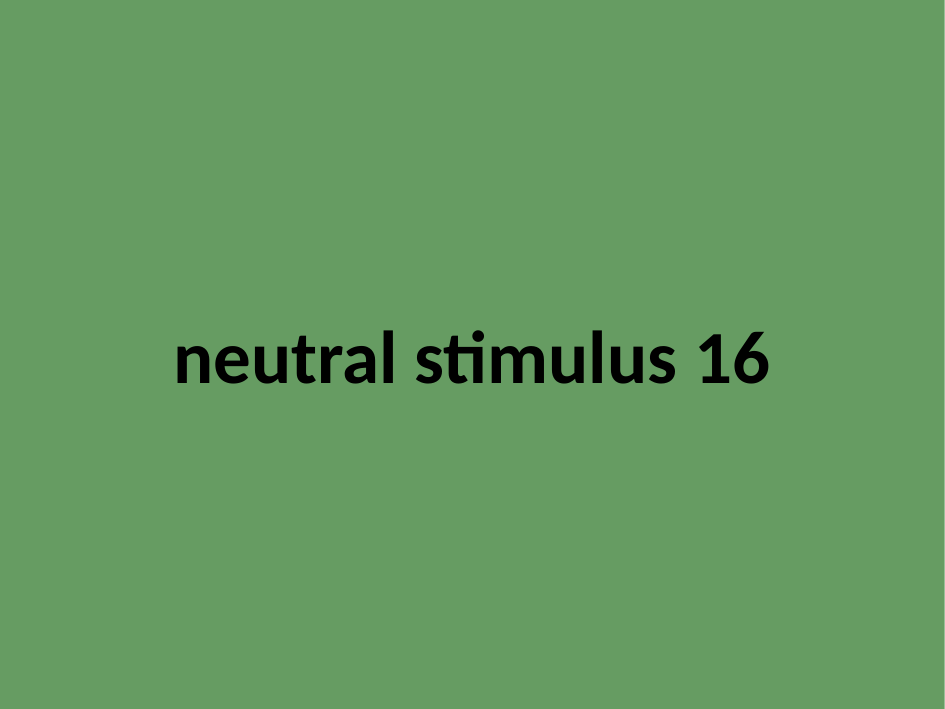

neutral stimulus 16

## Slide 17
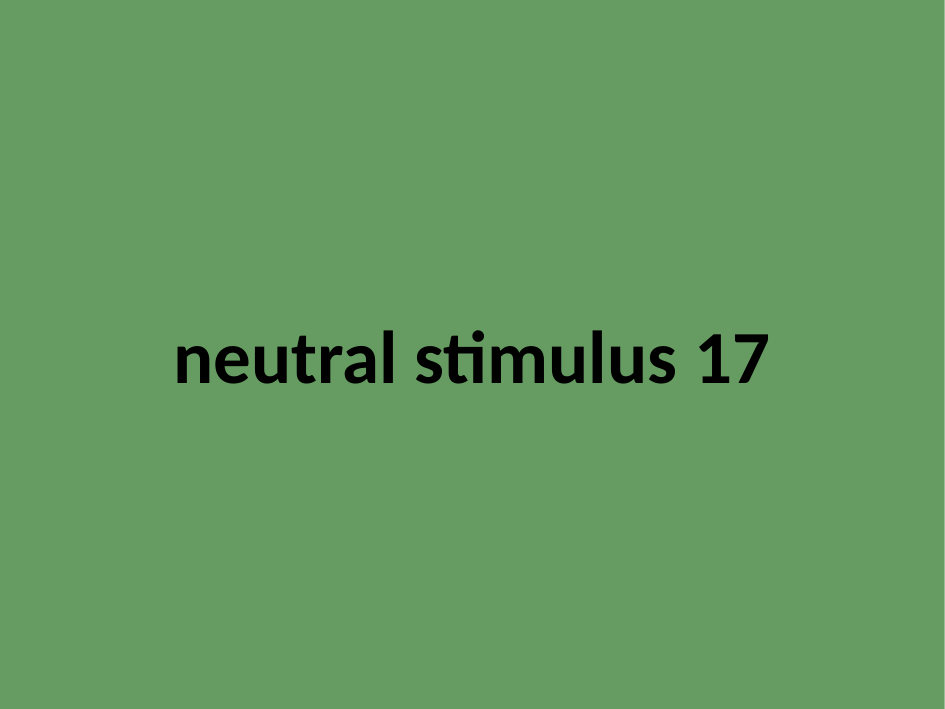

neutral stimulus 17

## Slide 18
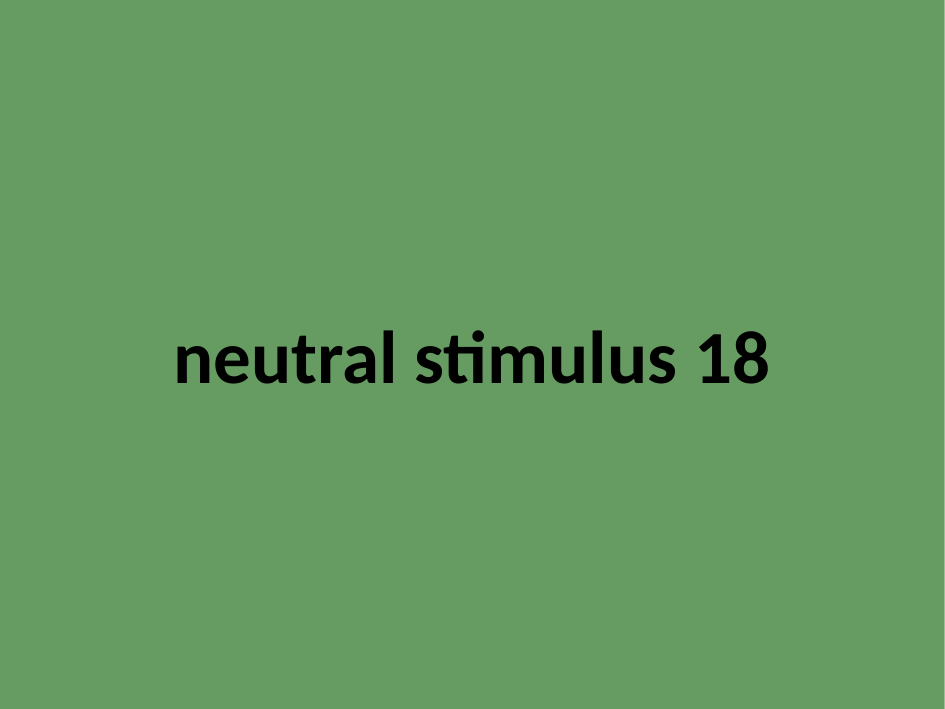

neutral stimulus 18

## Slide 19
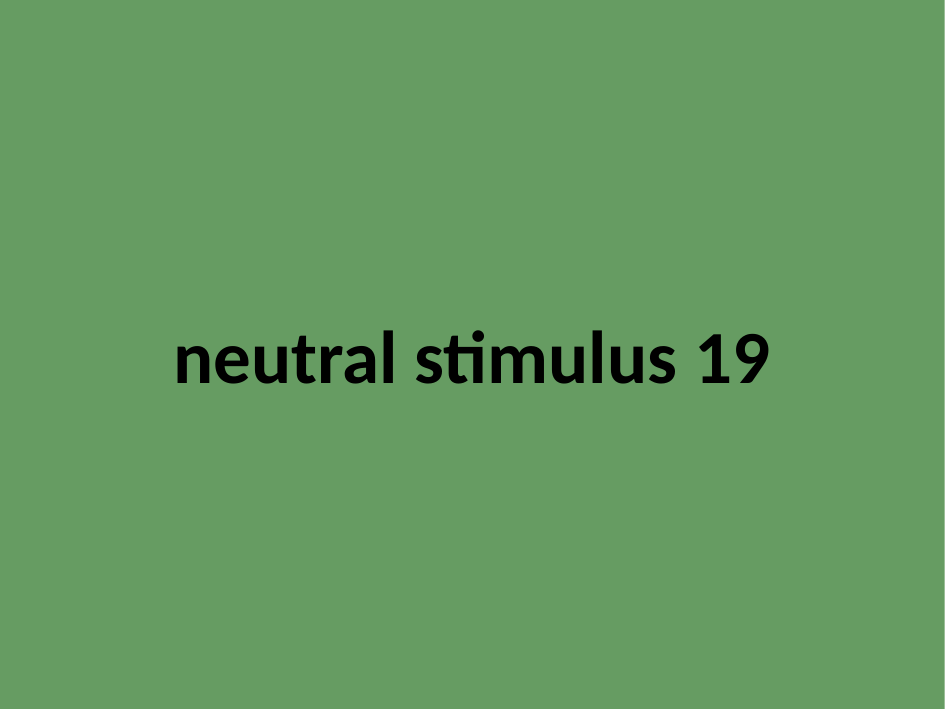

neutral stimulus 19

## Slide 20
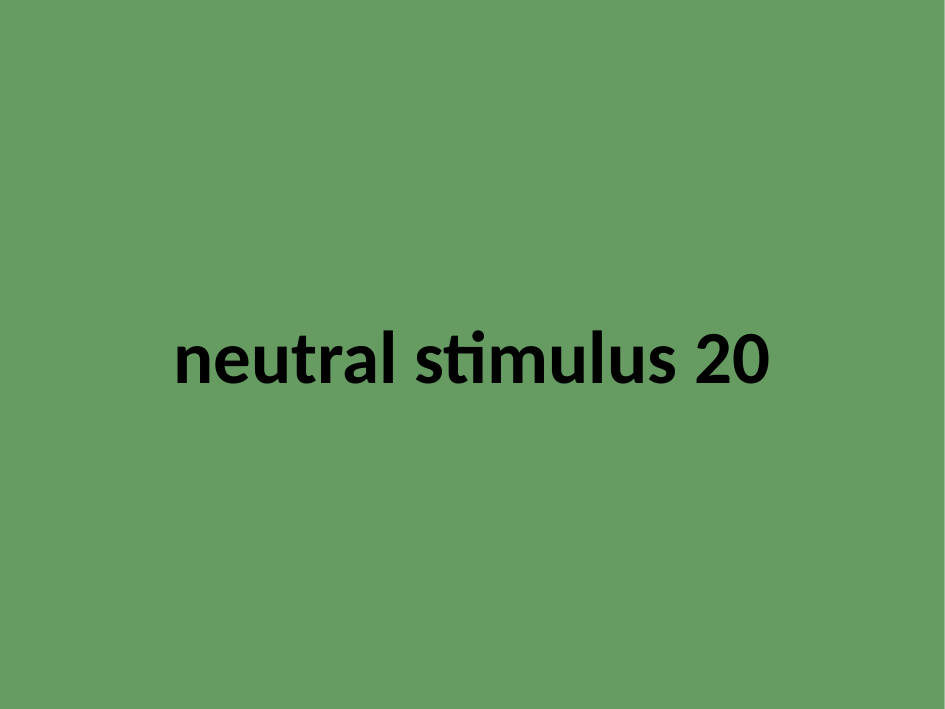

neutral stimulus 20

## Slide 21
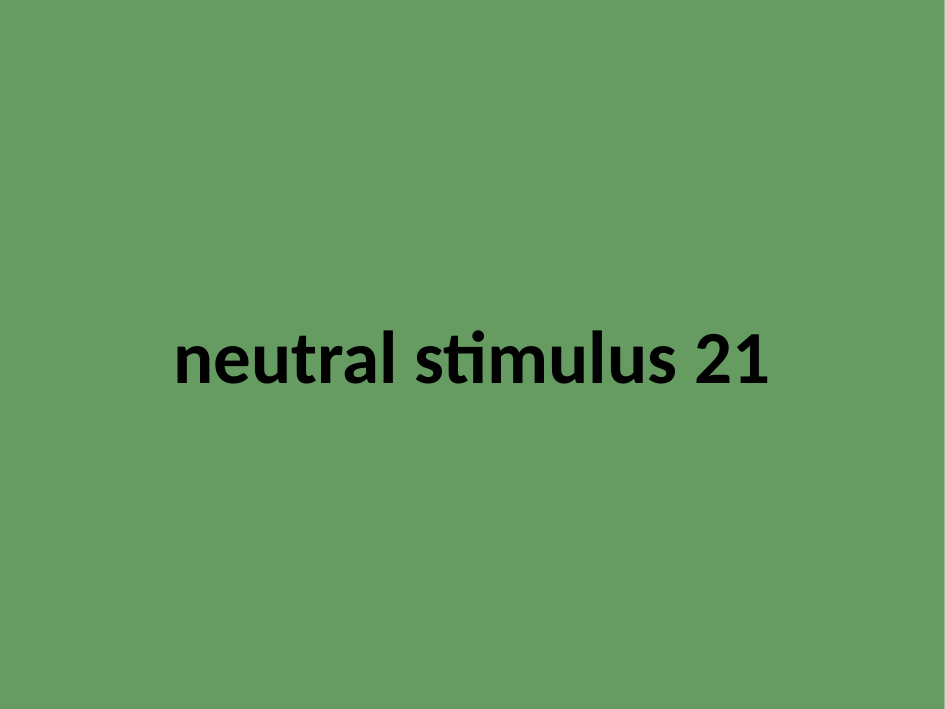

neutral stimulus 21

## Slide 22
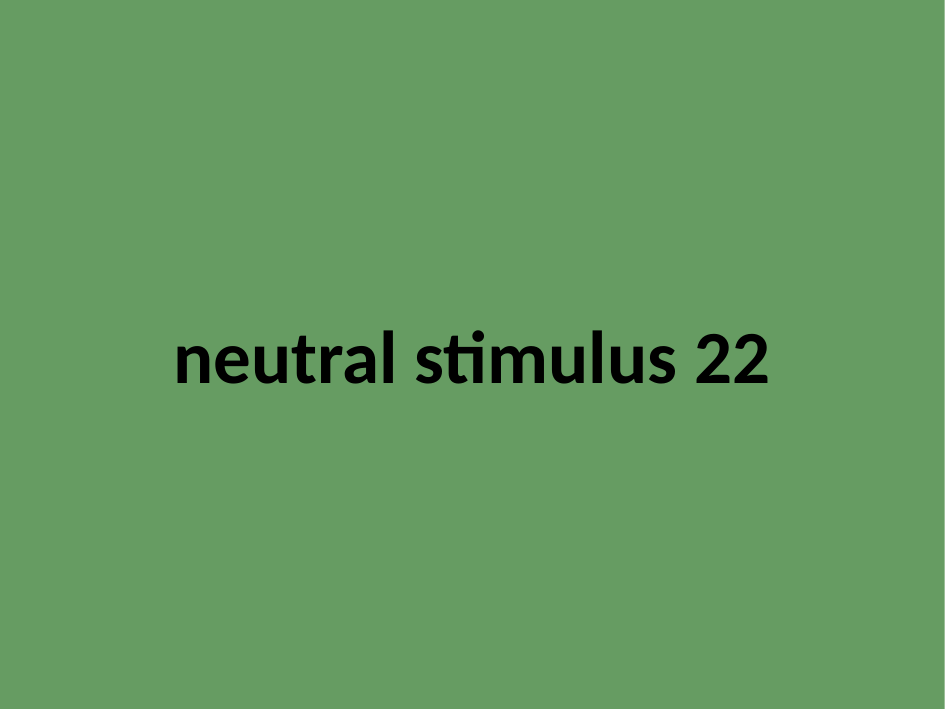

neutral stimulus 22

## Slide 23
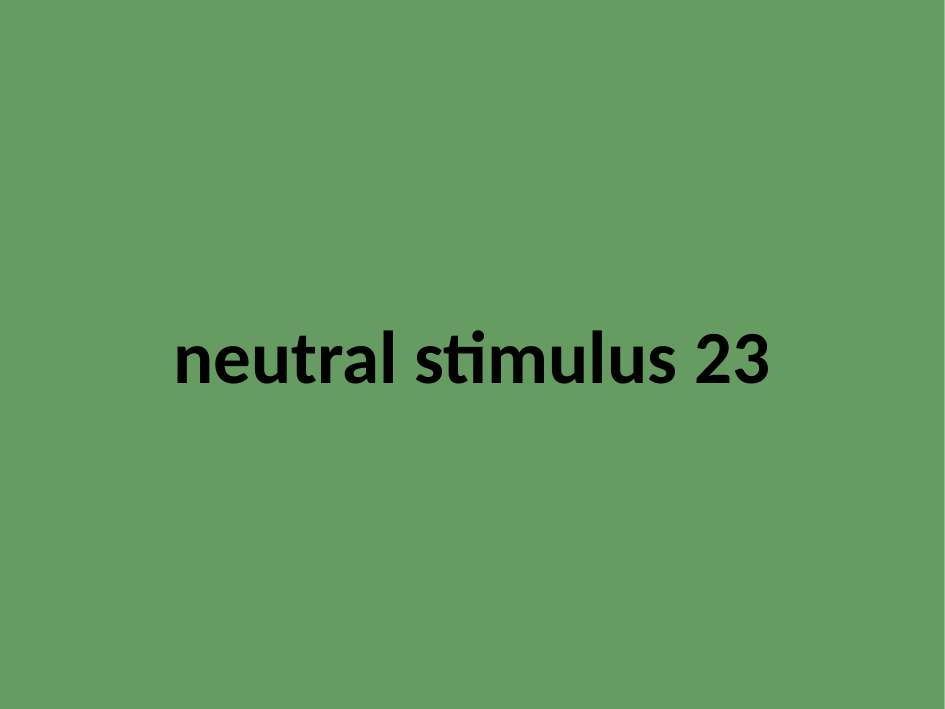

neutral stimulus 23

## Slide 24
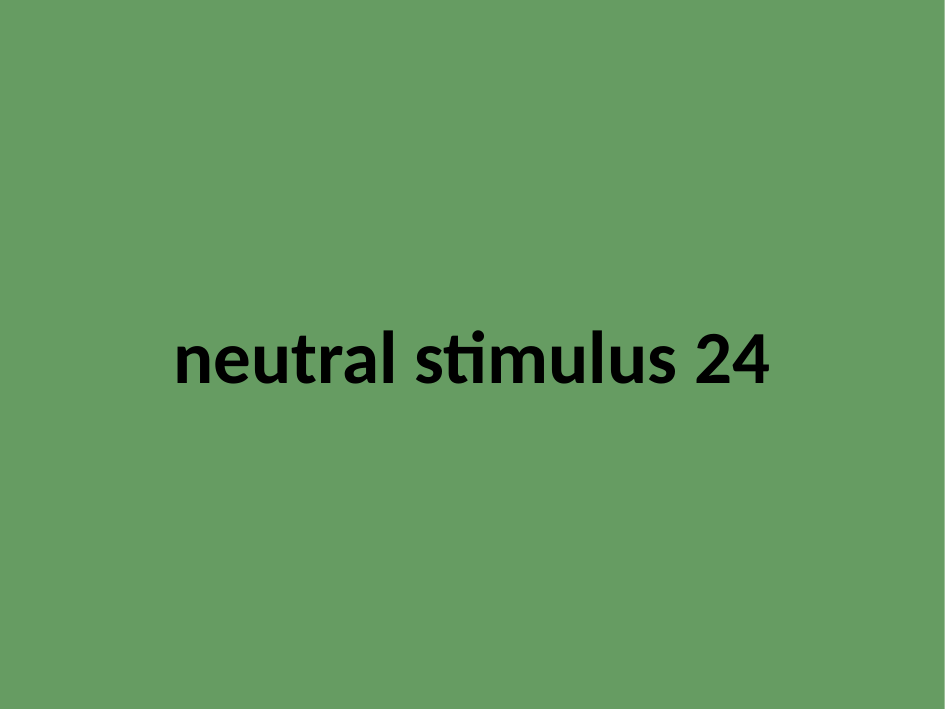

neutral stimulus 24

## Slide 25
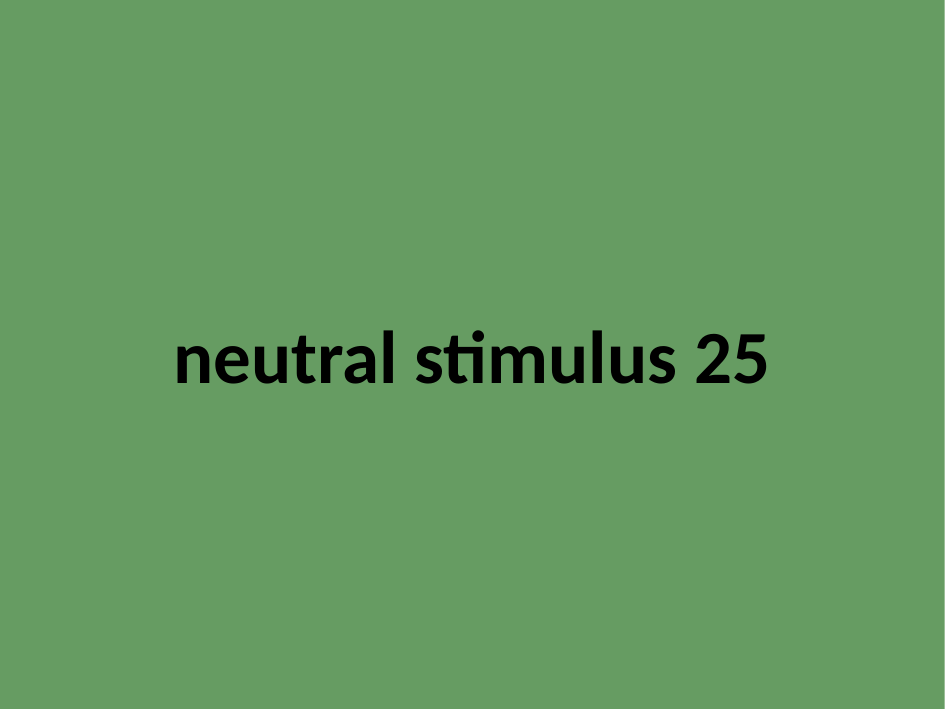

neutral stimulus 25

## Slide 26
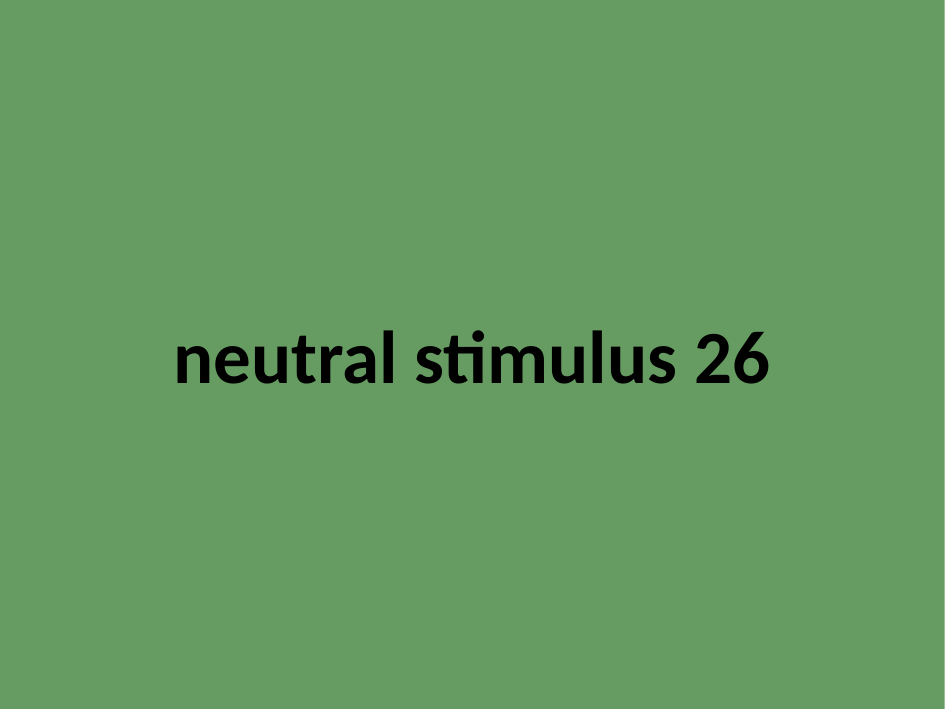

neutral stimulus 26

## Slide 27
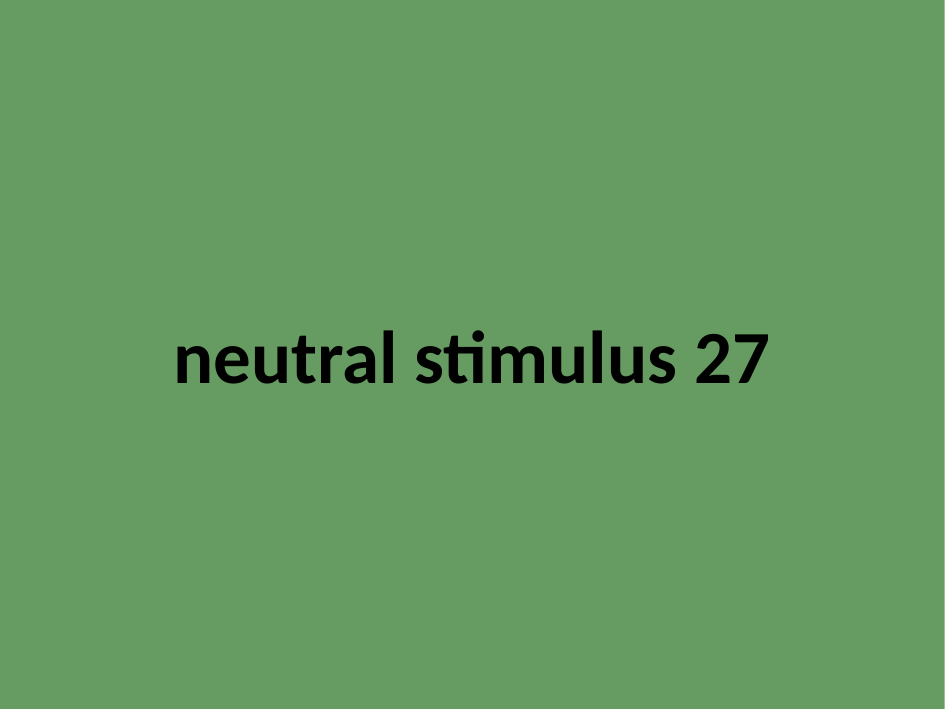

neutral stimulus 27

## Slide 28
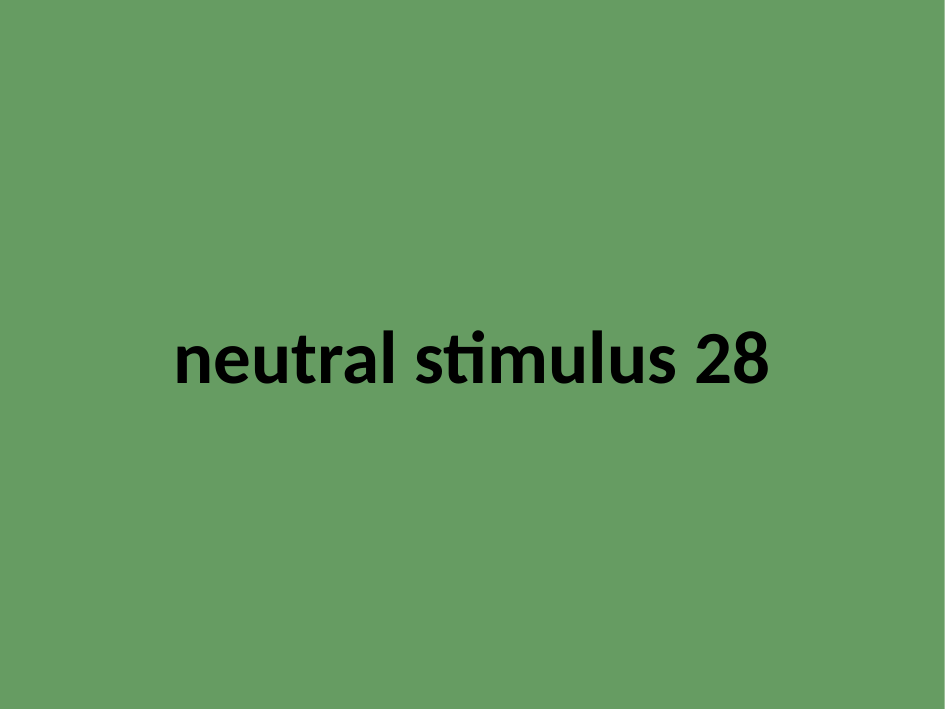

neutral stimulus 28

## Slide 29
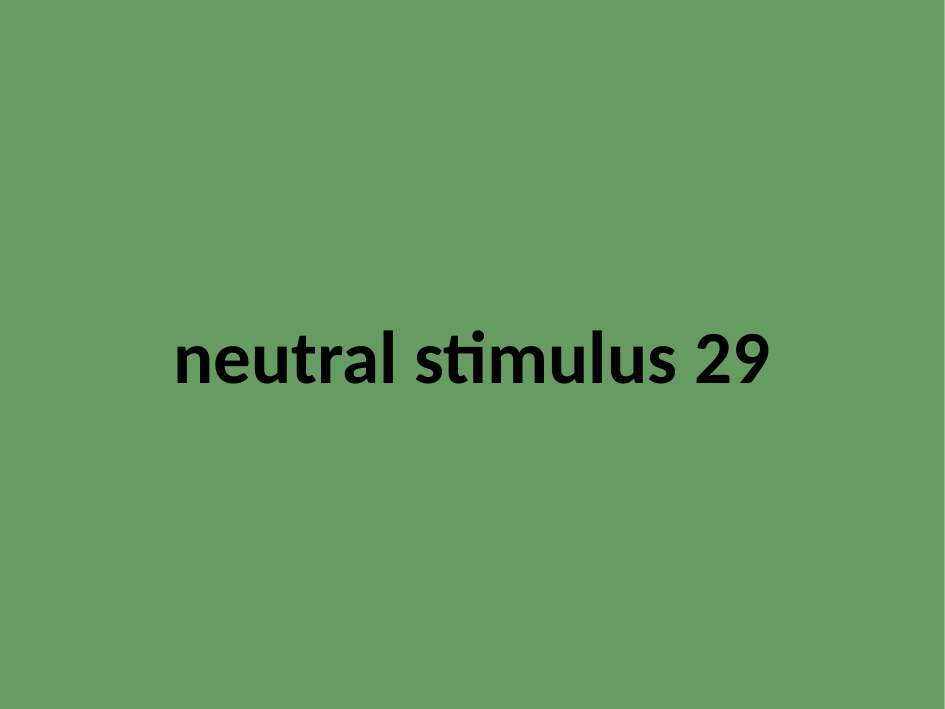

neutral stimulus 29

## Slide 30
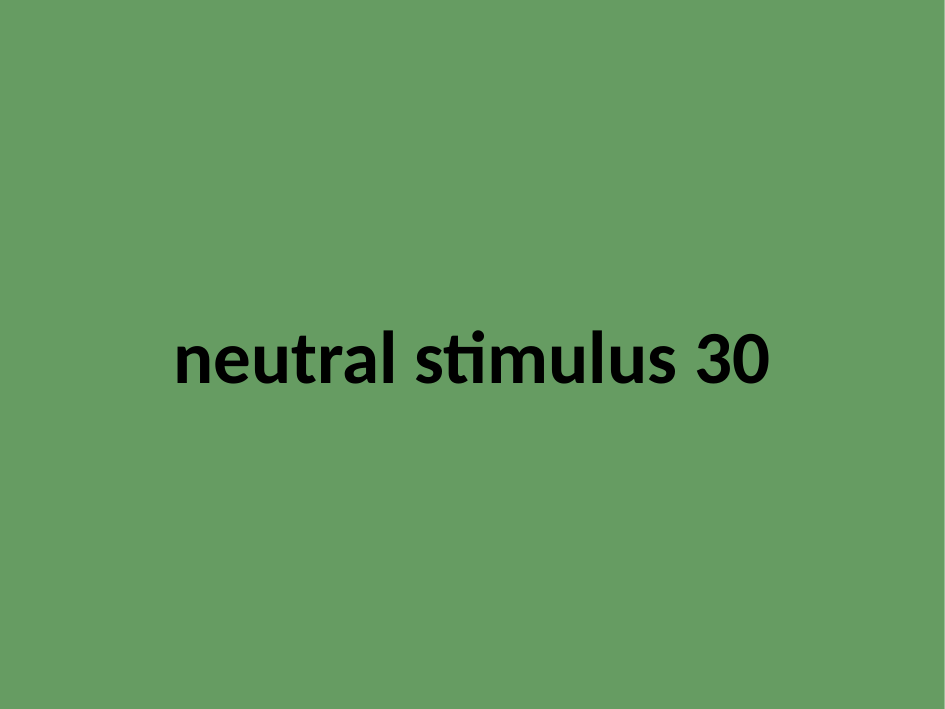

neutral stimulus 30

## Slide 31
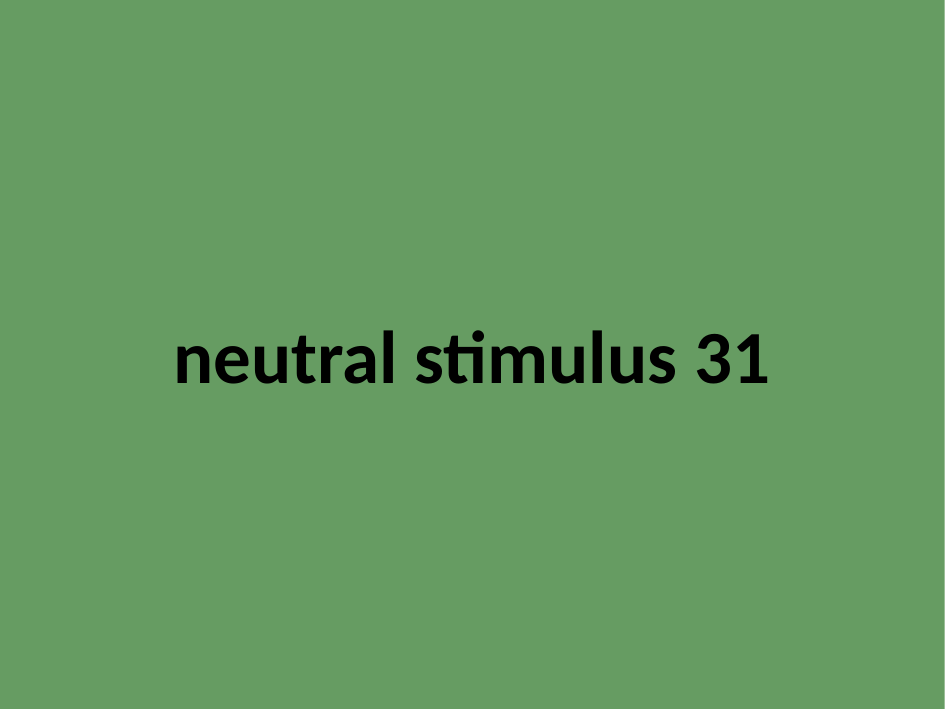

neutral stimulus 31

## Slide 32
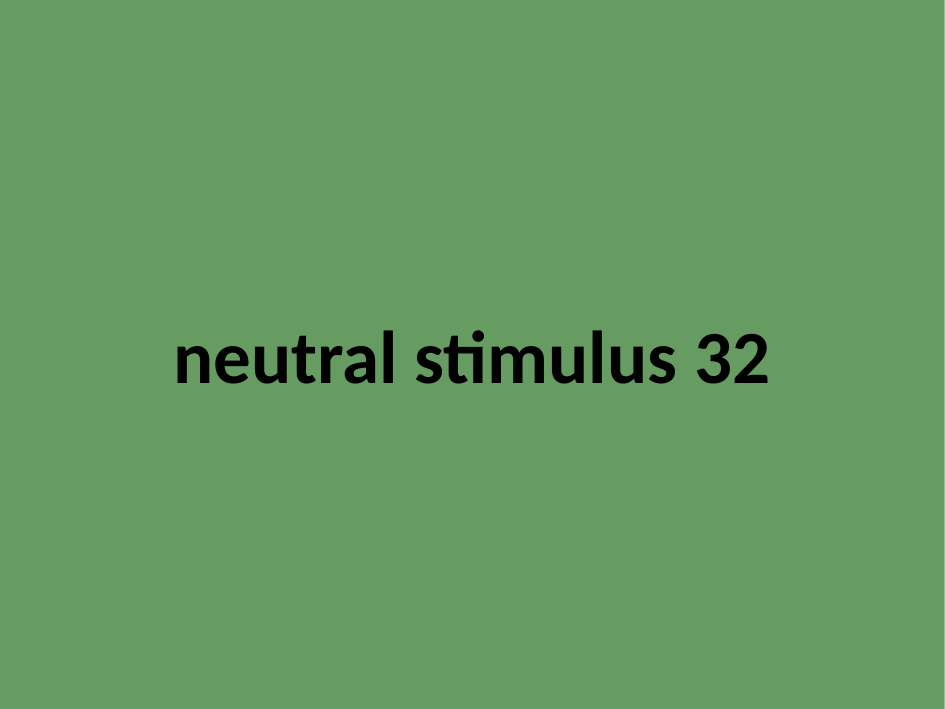

neutral stimulus 32

## Slide 33
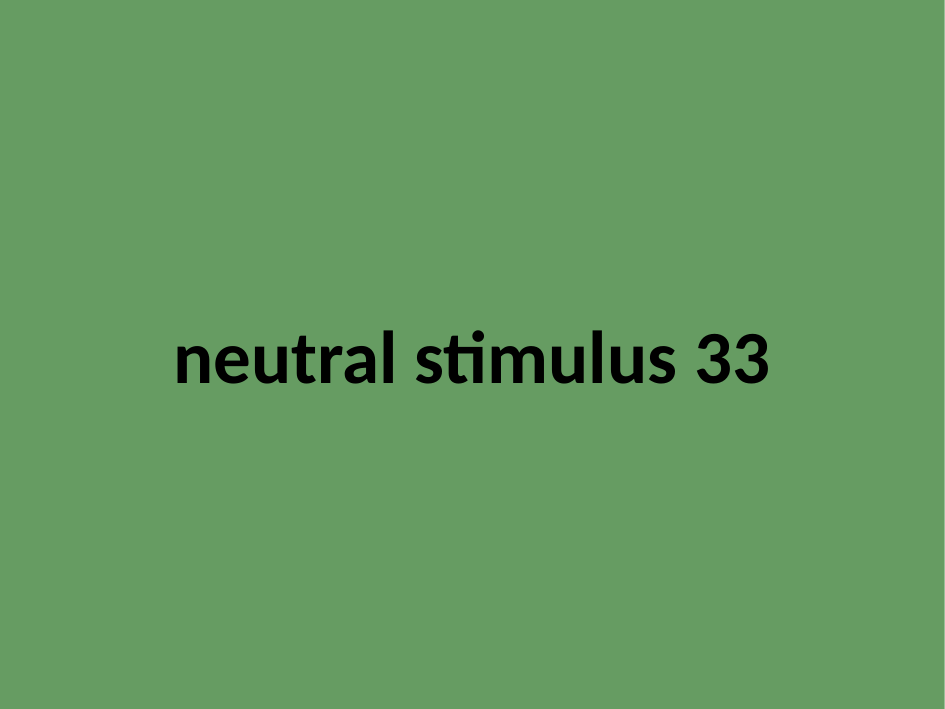

neutral stimulus 33

## Slide 34
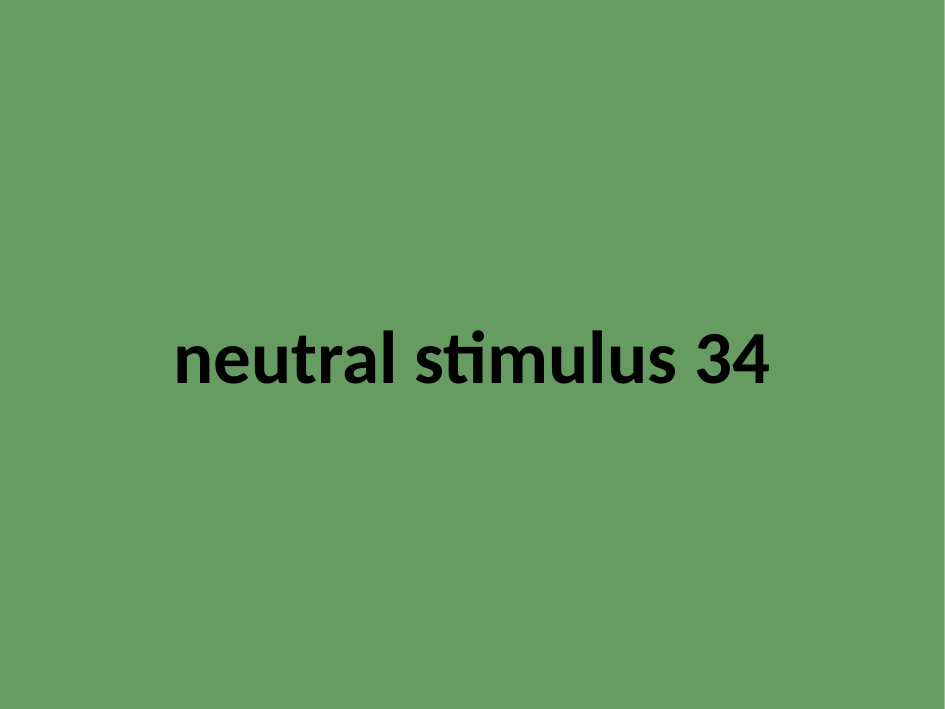

neutral stimulus 34

## Slide 35
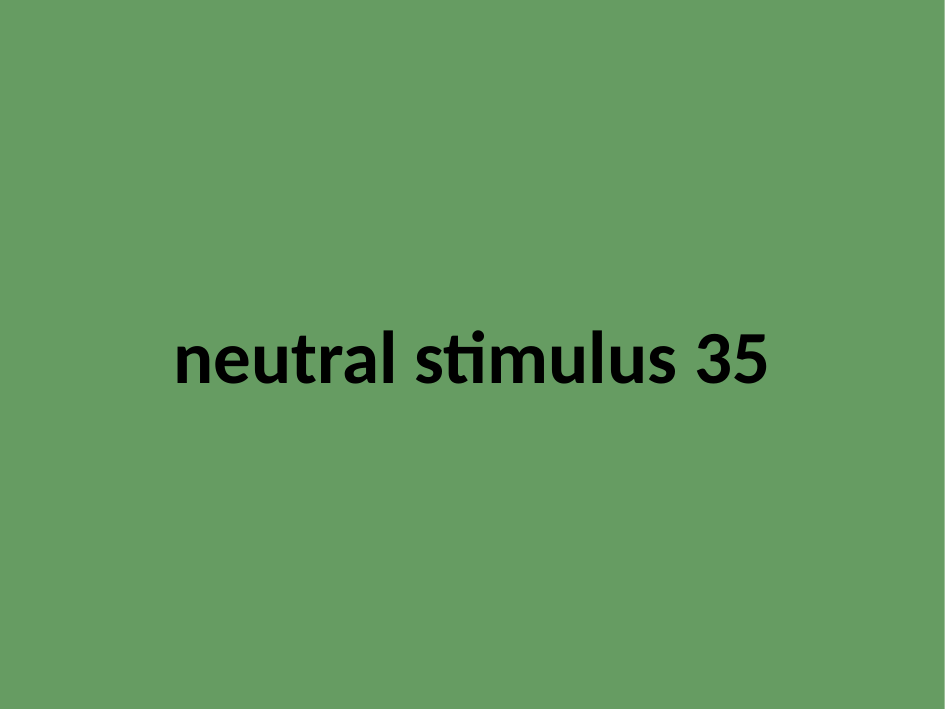

neutral stimulus 35

## Slide 36
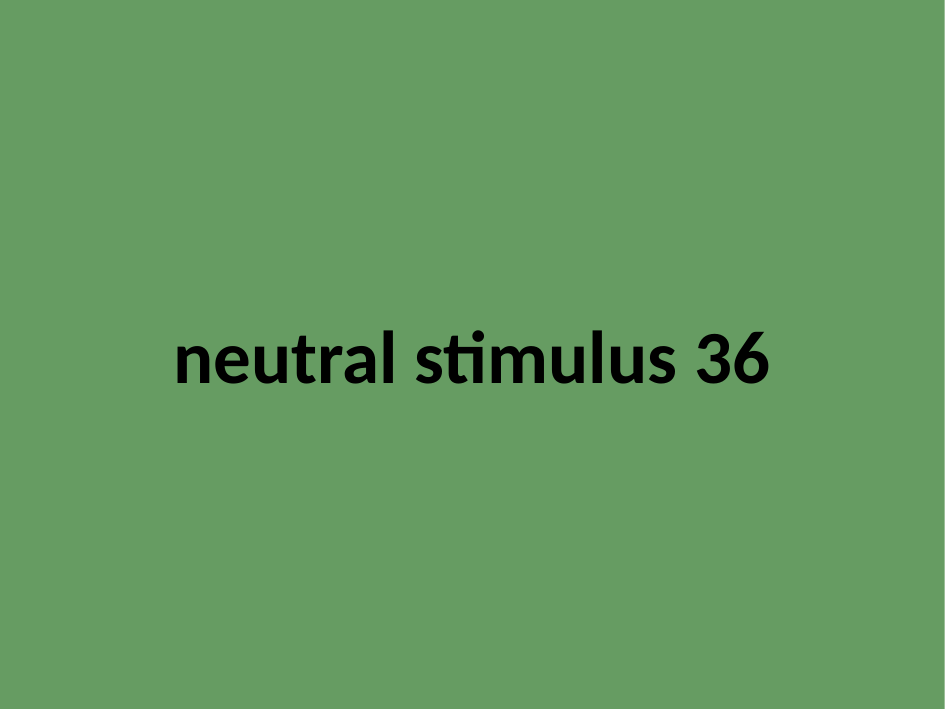

neutral stimulus 36

## Slide 37
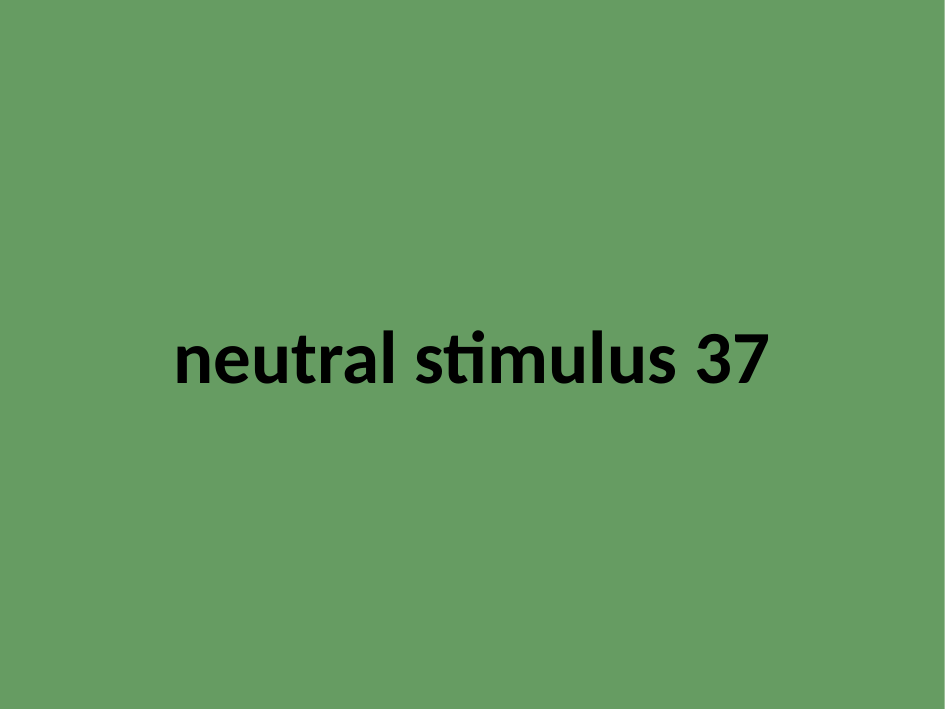

neutral stimulus 37

## Slide 38
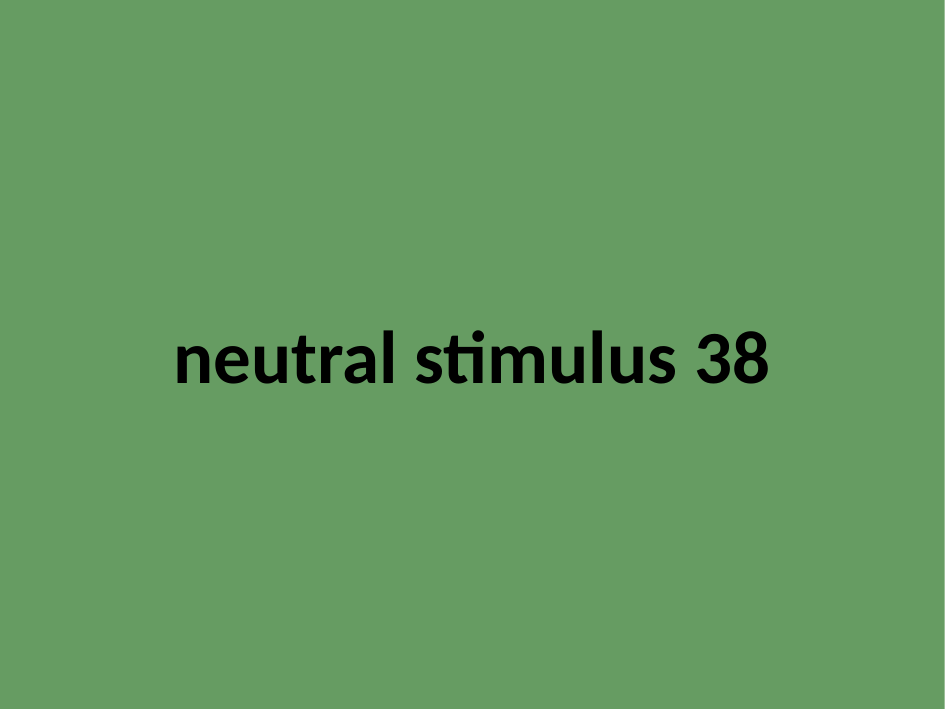

neutral stimulus 38

## Slide 39
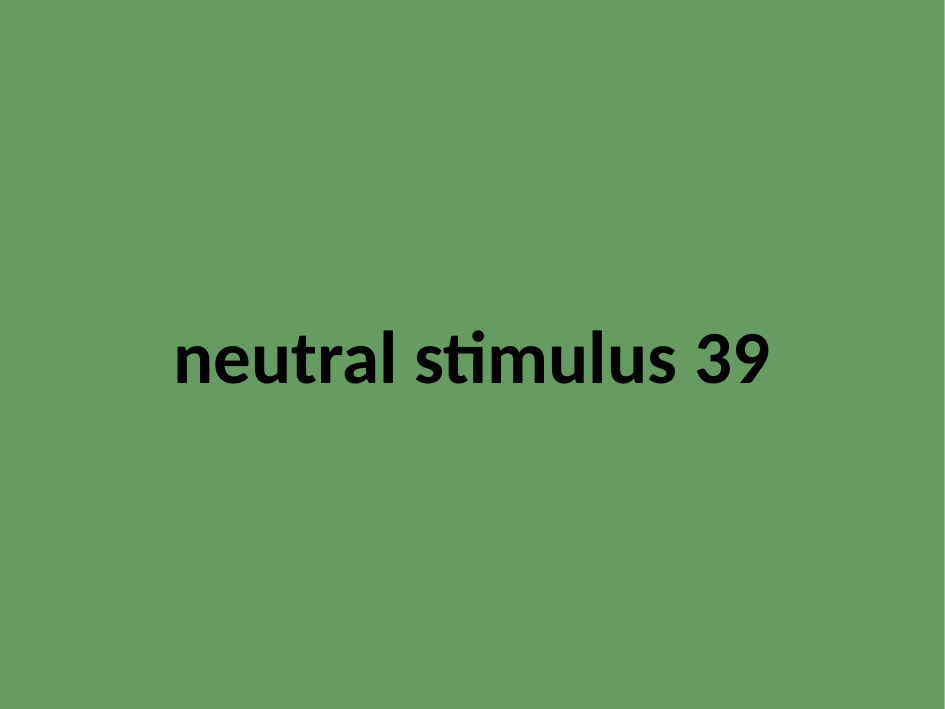

neutral stimulus 39

## Slide 40
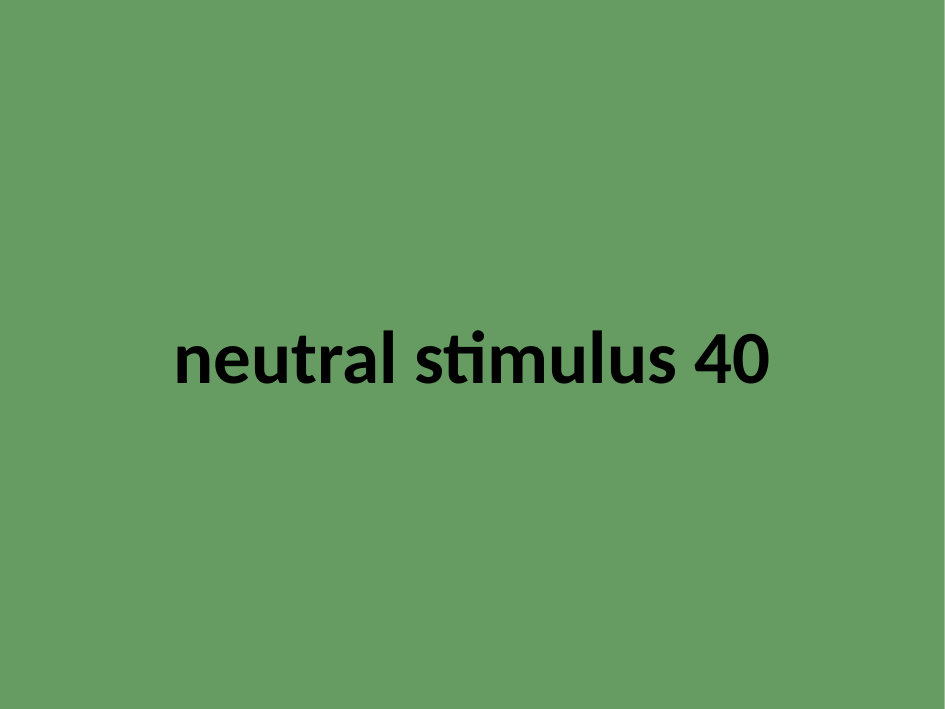

neutral stimulus 40

## Slide 41
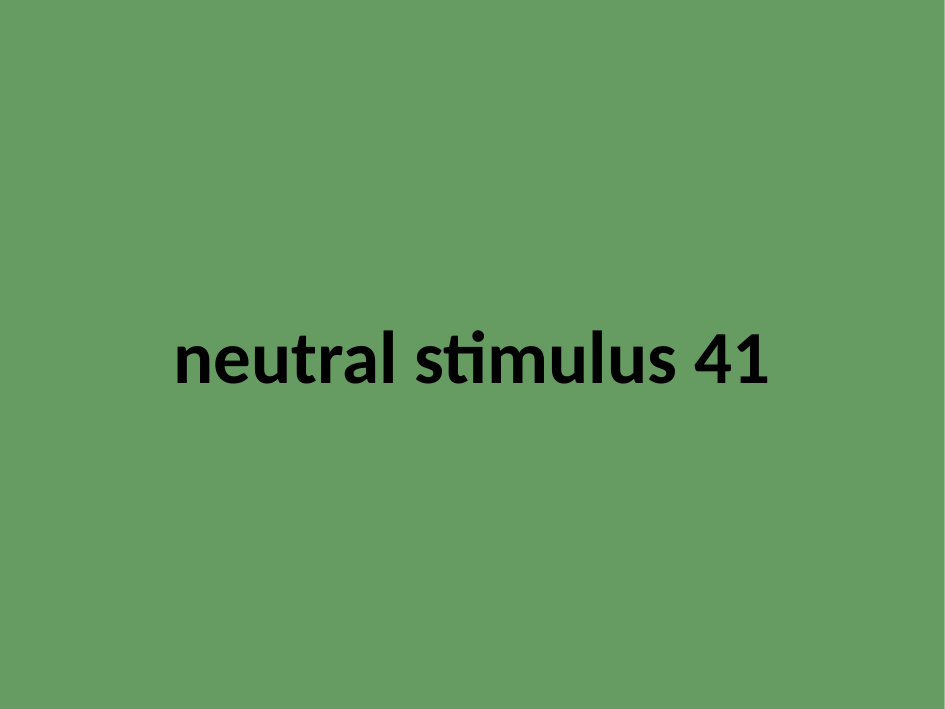

neutral stimulus 41

## Slide 42
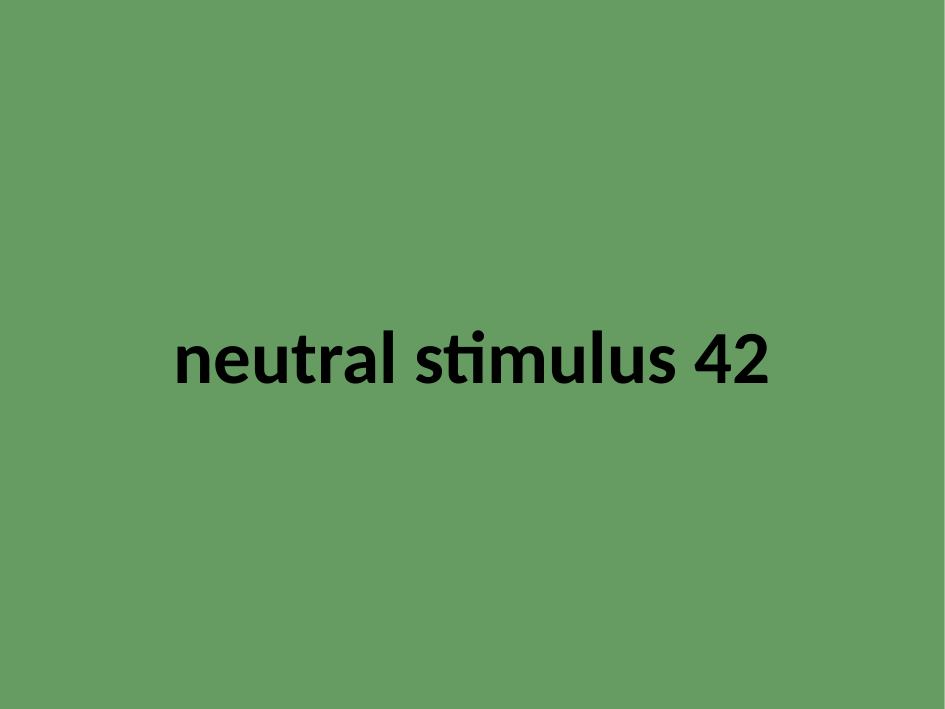

neutral stimulus 42

## Slide 43
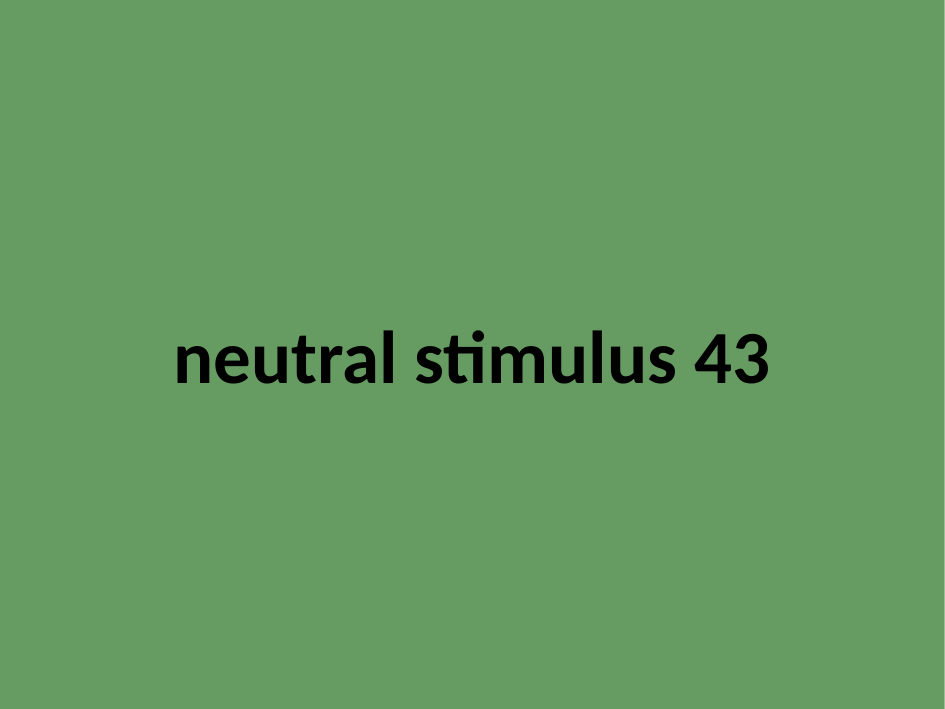

neutral stimulus 43

## Slide 44
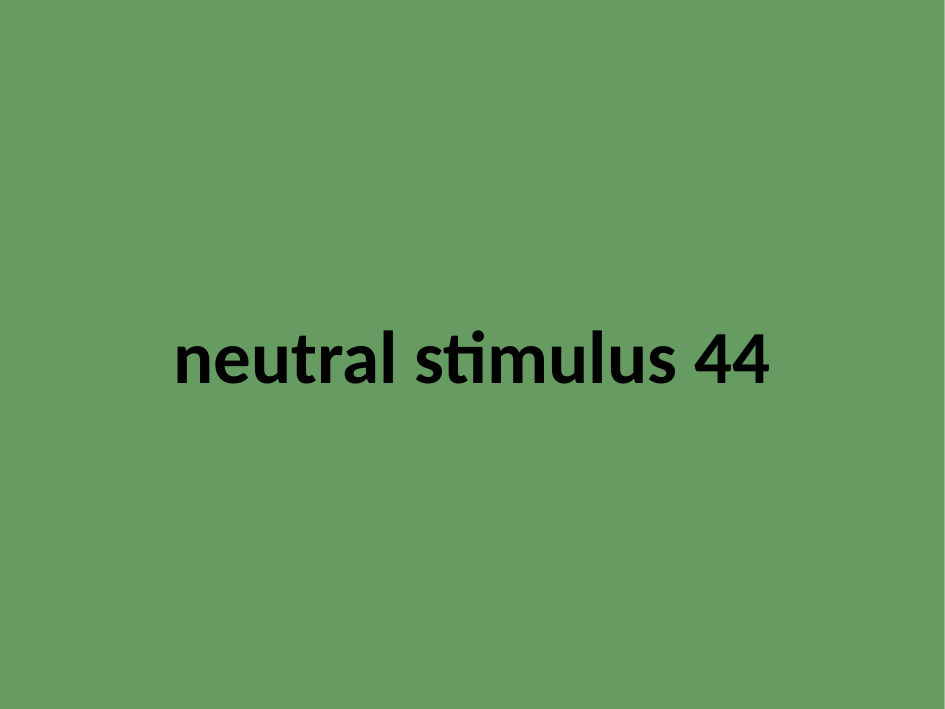

neutral stimulus 44

## Slide 45
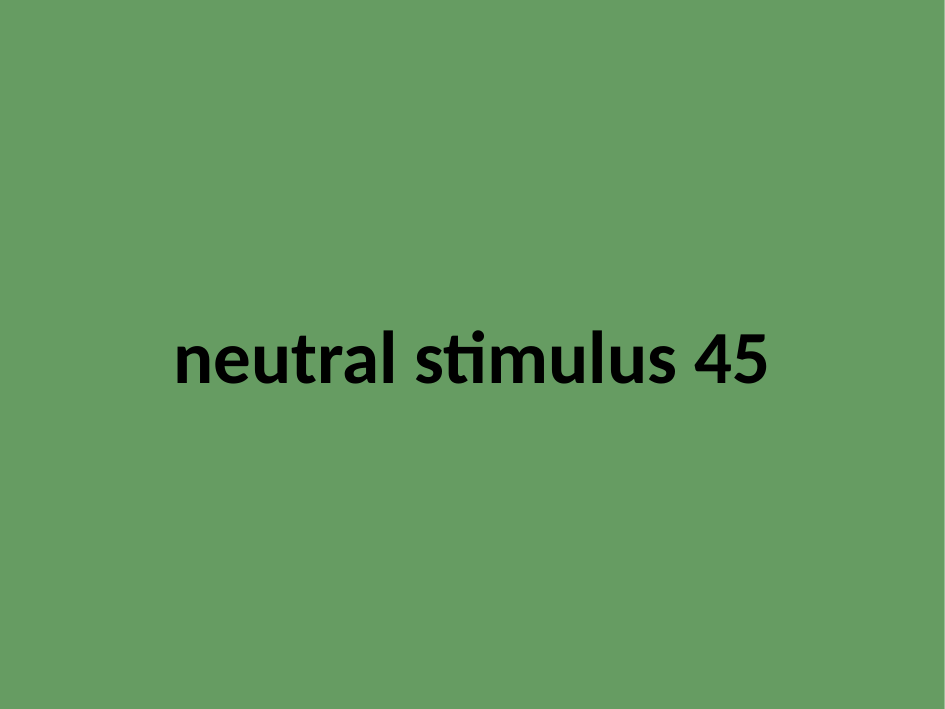

neutral stimulus 45

## Slide 46
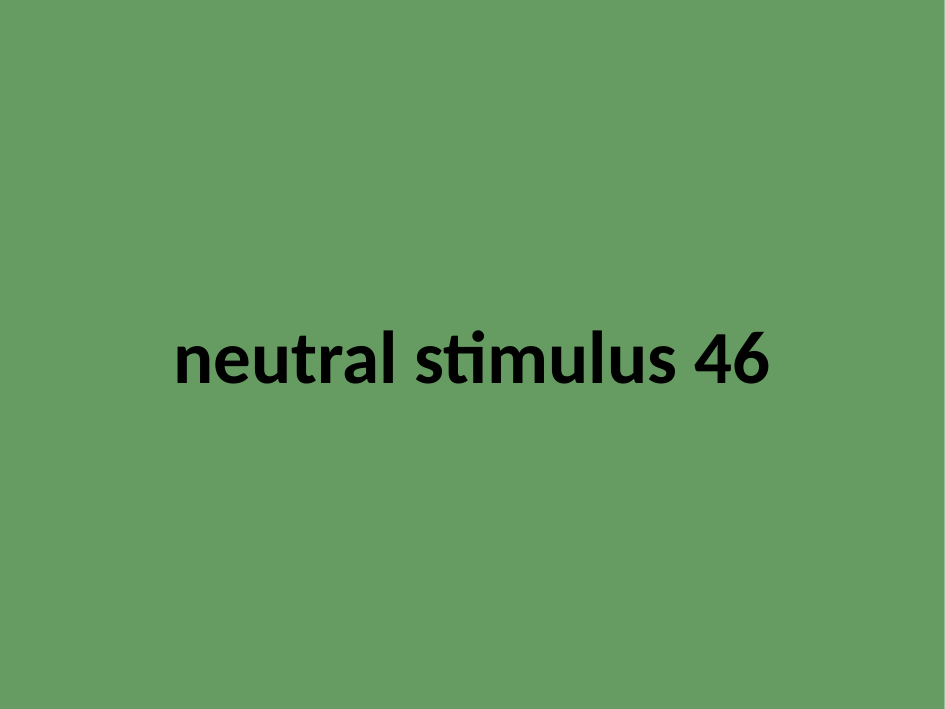

neutral stimulus 46

## Slide 47
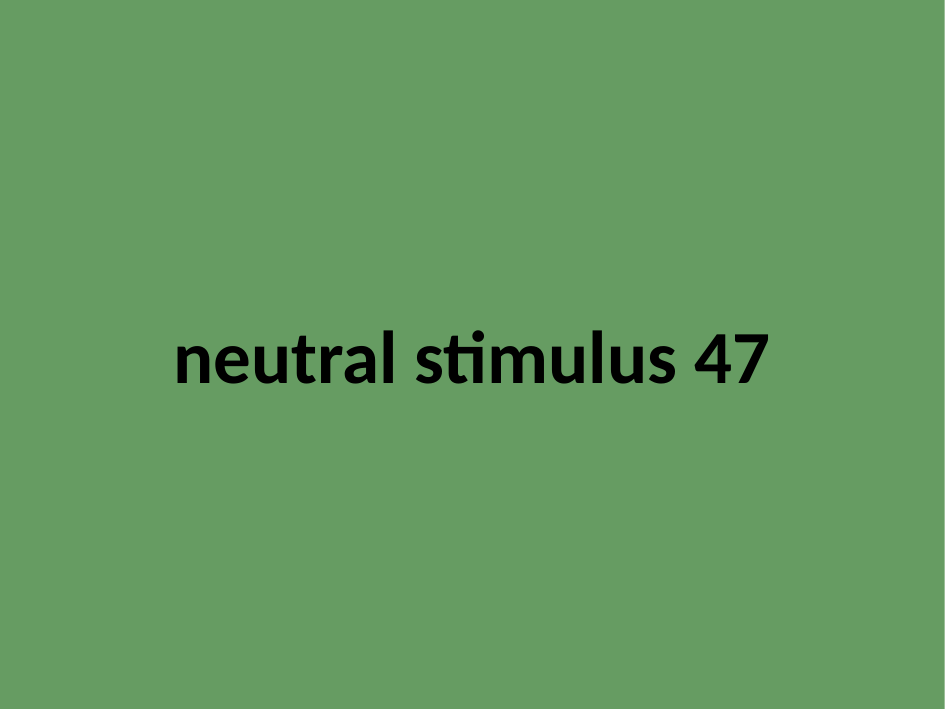

neutral stimulus 47

## Slide 48
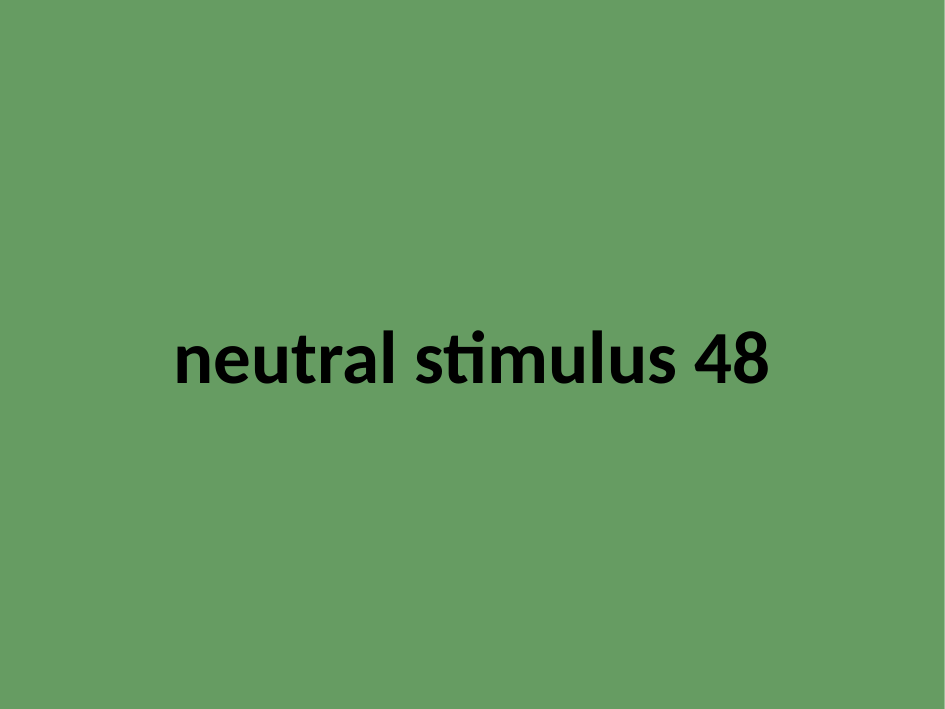

neutral stimulus 48

## Slide 49
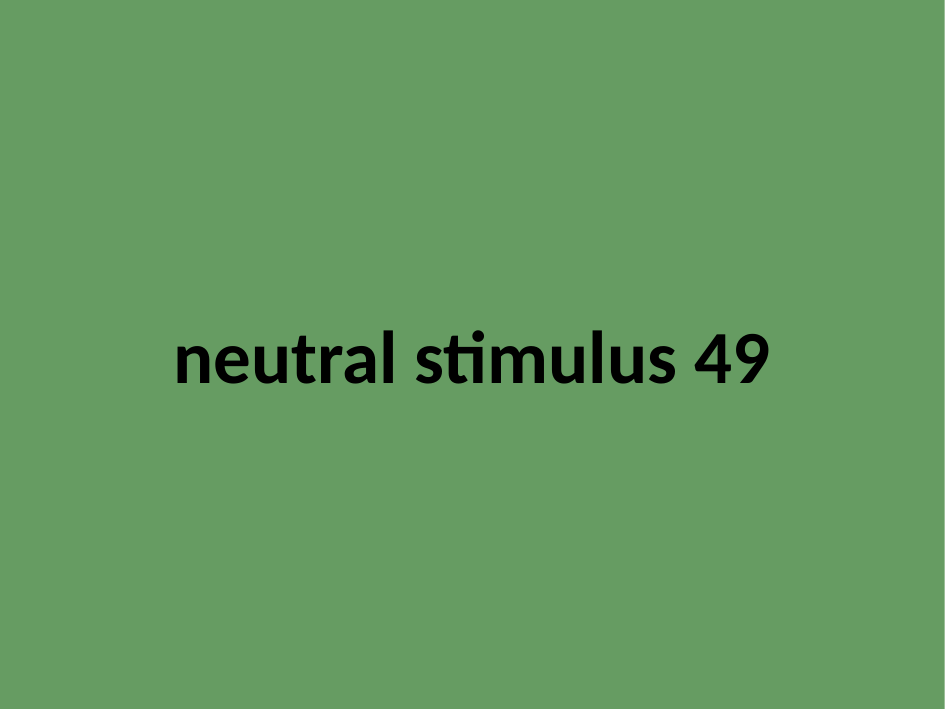

neutral stimulus 49

## Slide 50
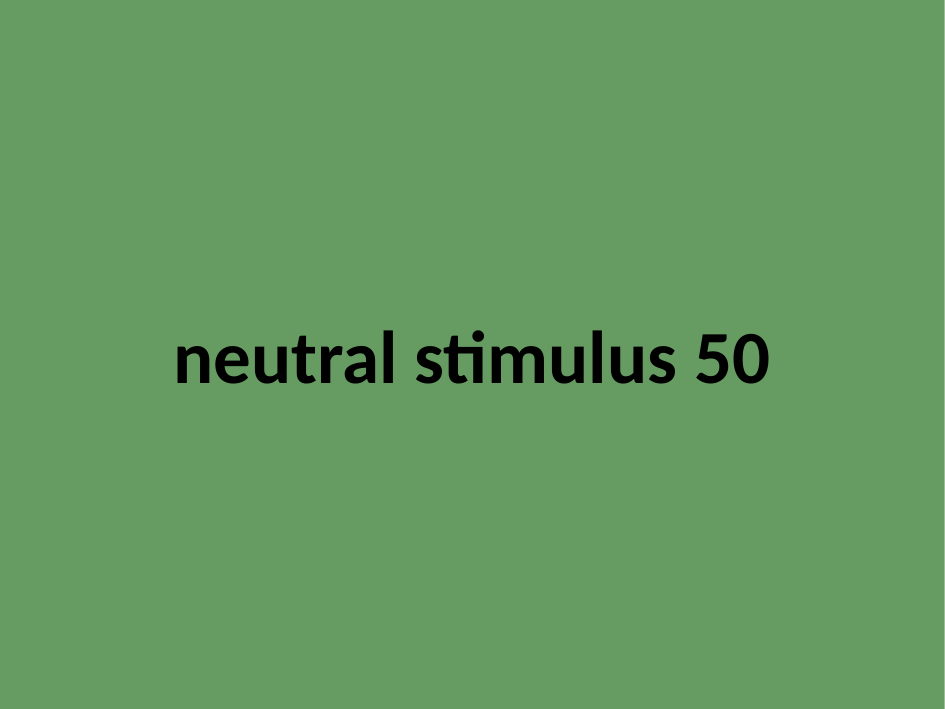

neutral stimulus 50

## Slide 51
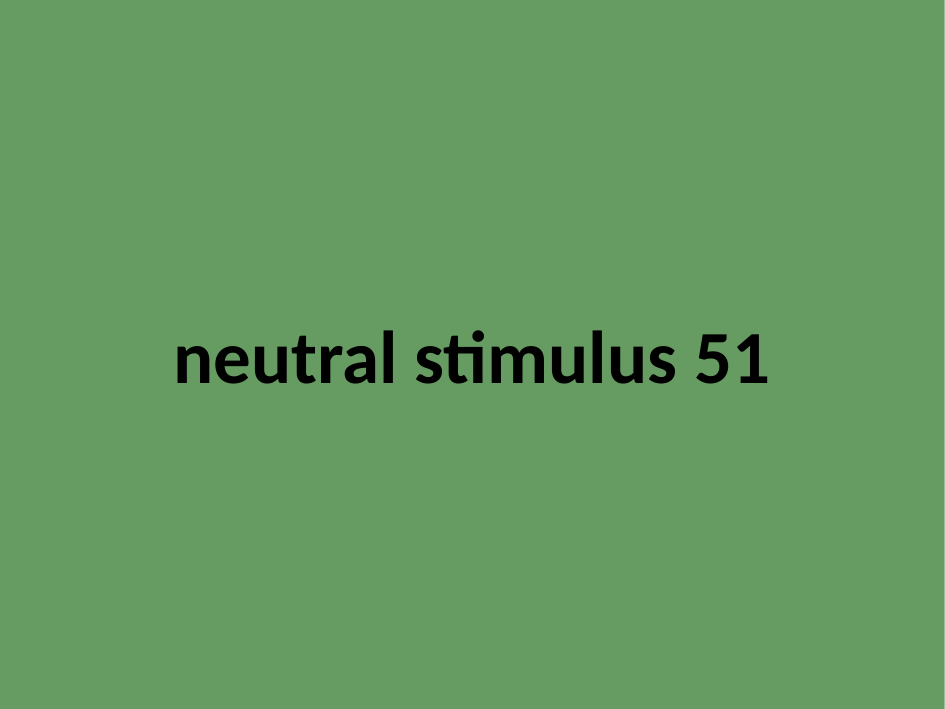

neutral stimulus 51

## Slide 52
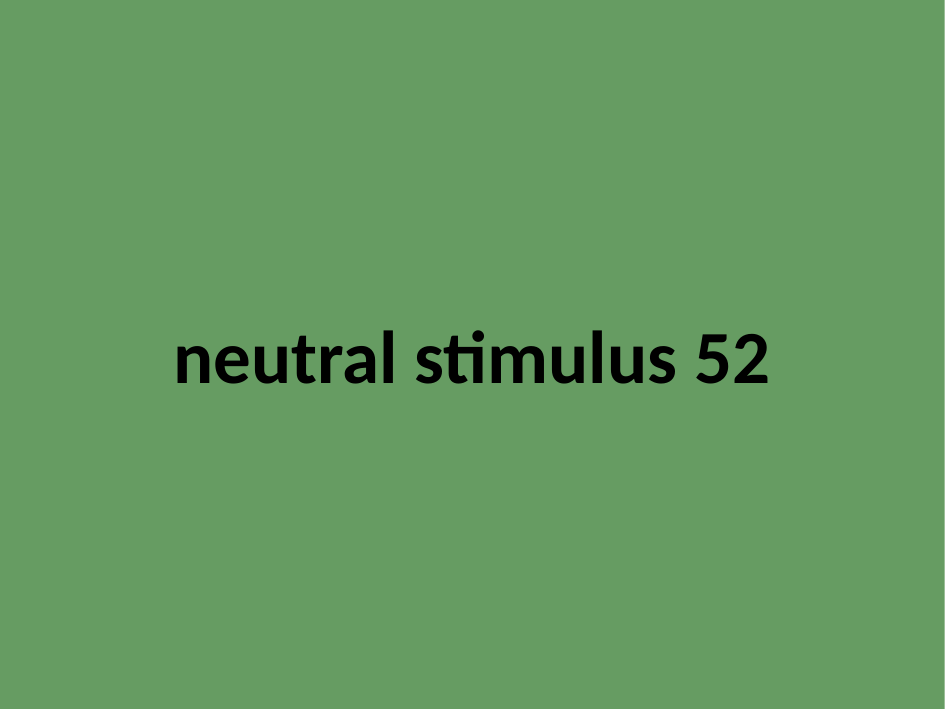

neutral stimulus 52

## Slide 53
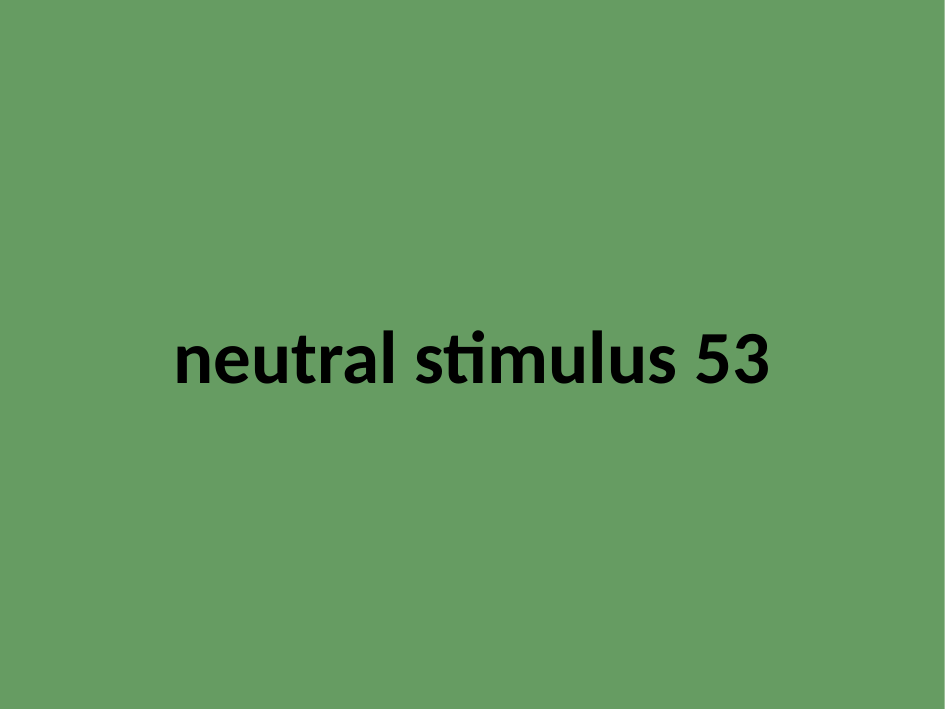

neutral stimulus 53

## Slide 54
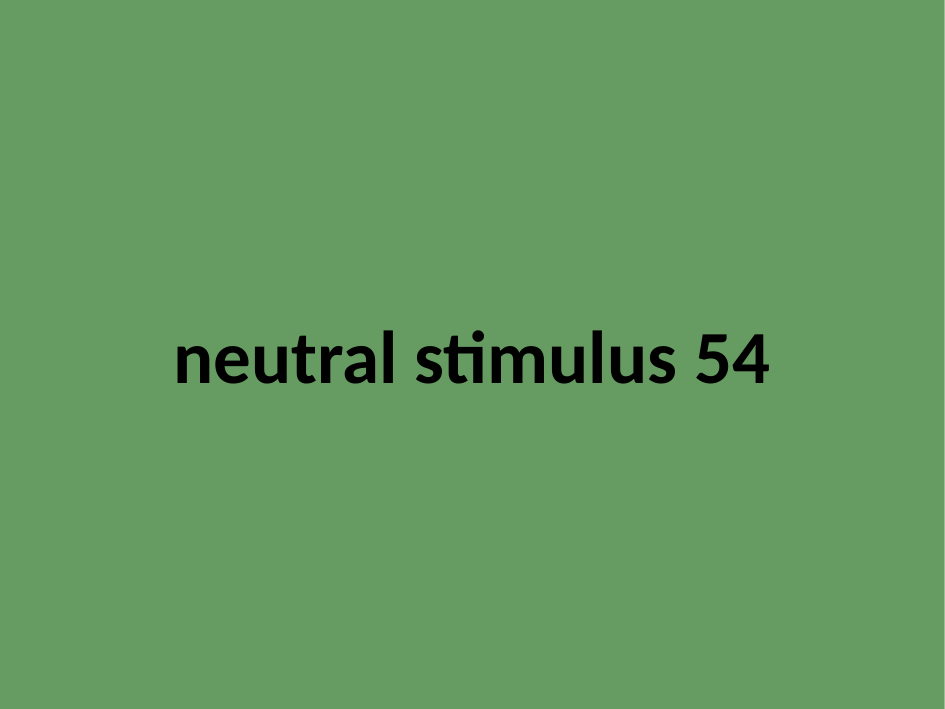

neutral stimulus 54

## Slide 55
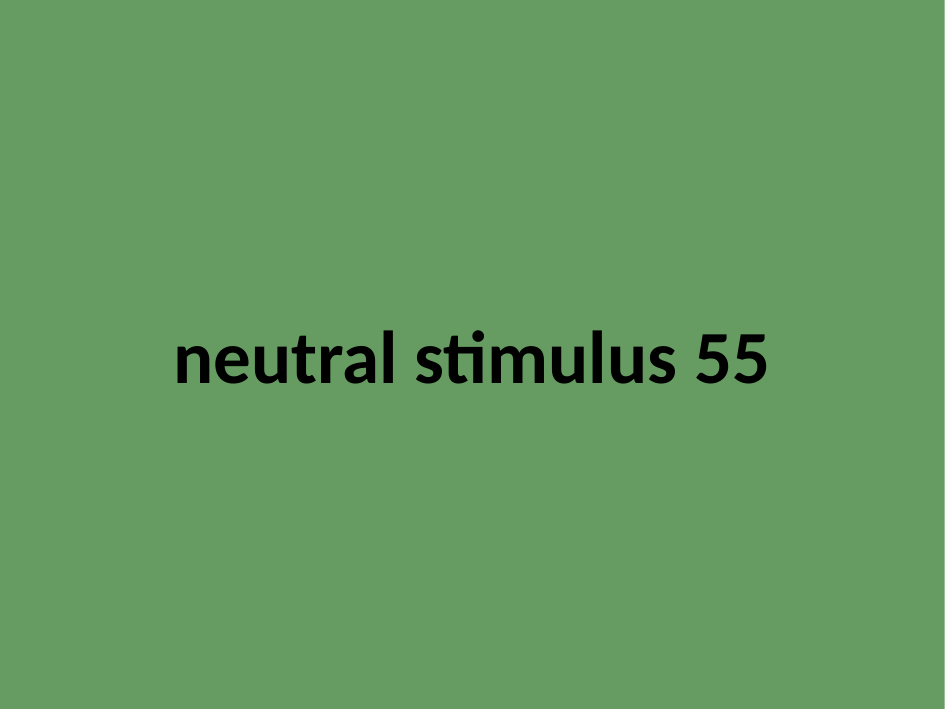

neutral stimulus 55

## Slide 56
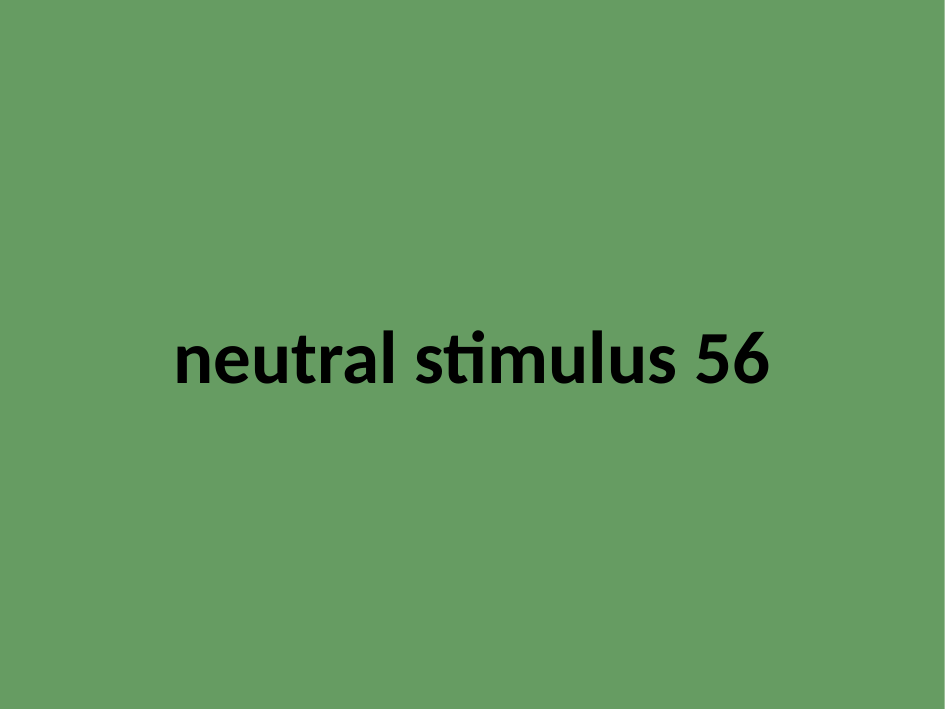

neutral stimulus 56

## Slide 57
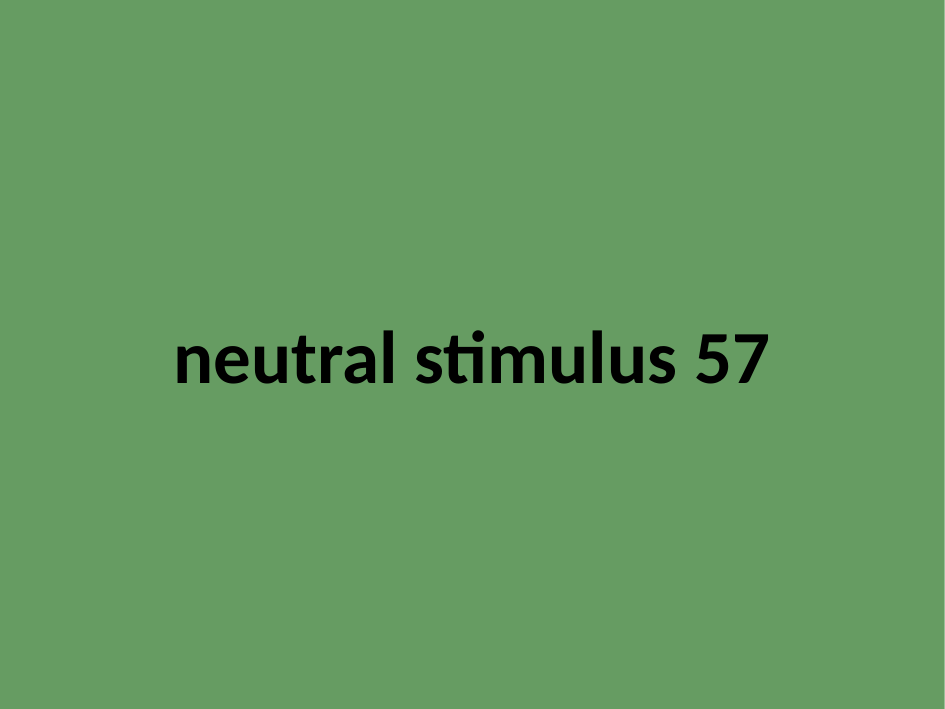

neutral stimulus 57

## Slide 58
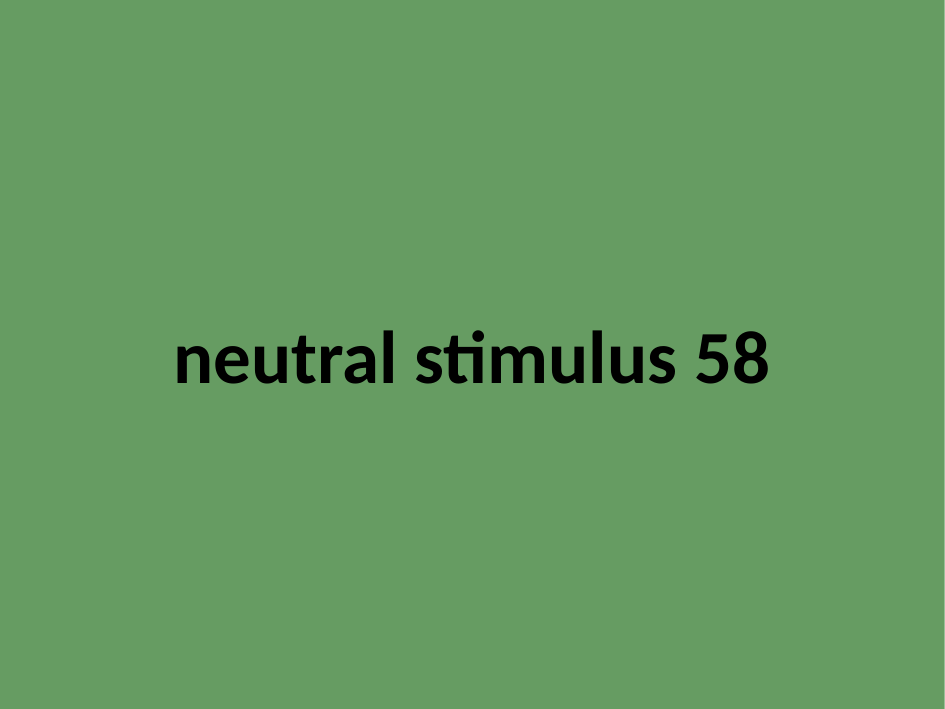

neutral stimulus 58

## Slide 59
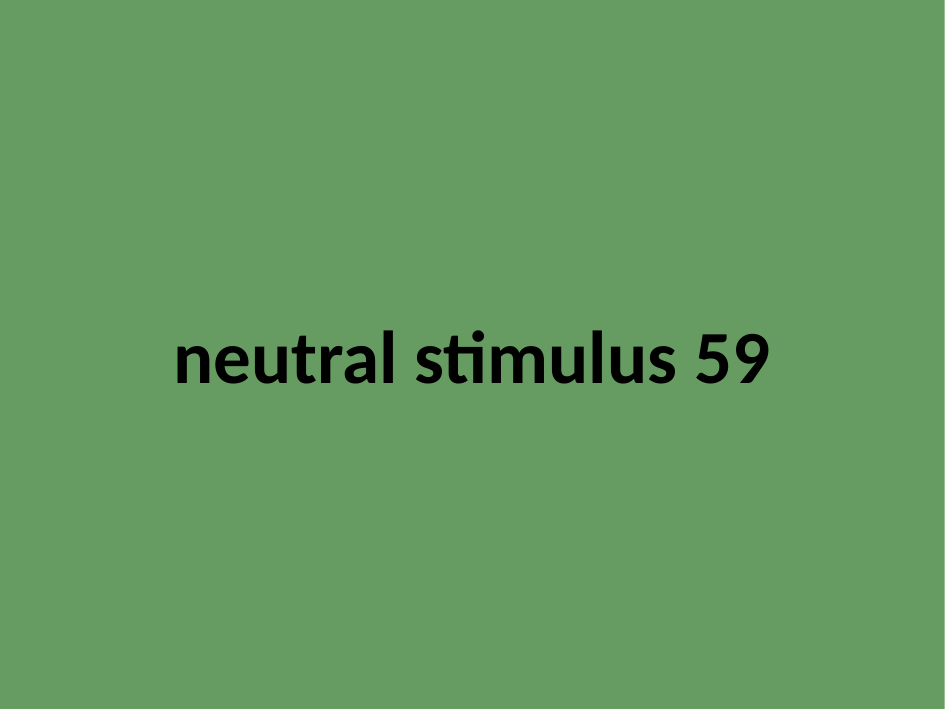

neutral stimulus 59

## Slide 60
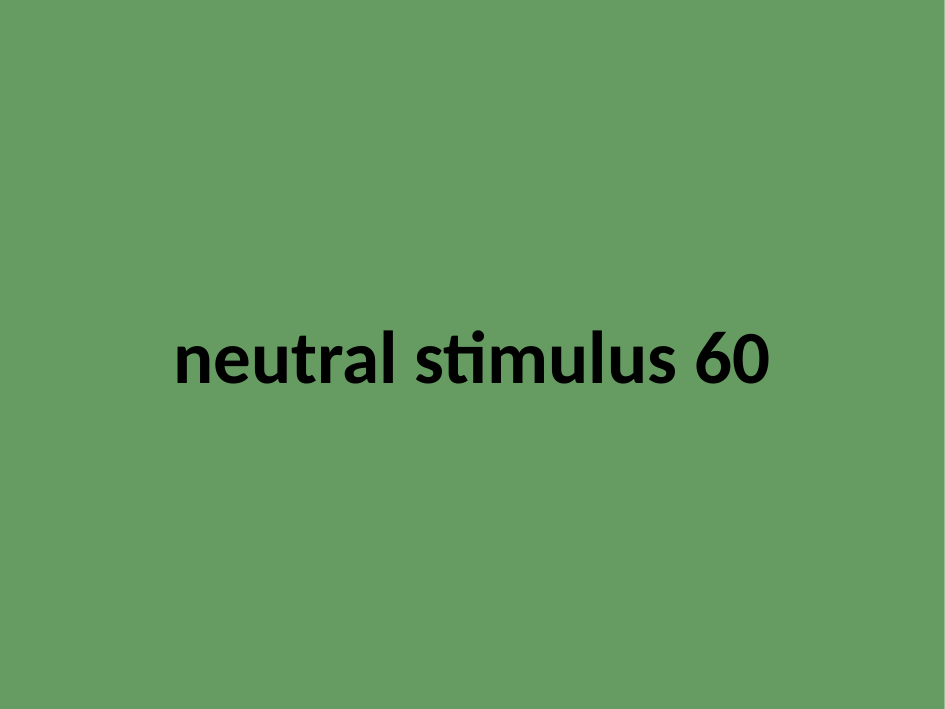

neutral stimulus 60

## Slide 61
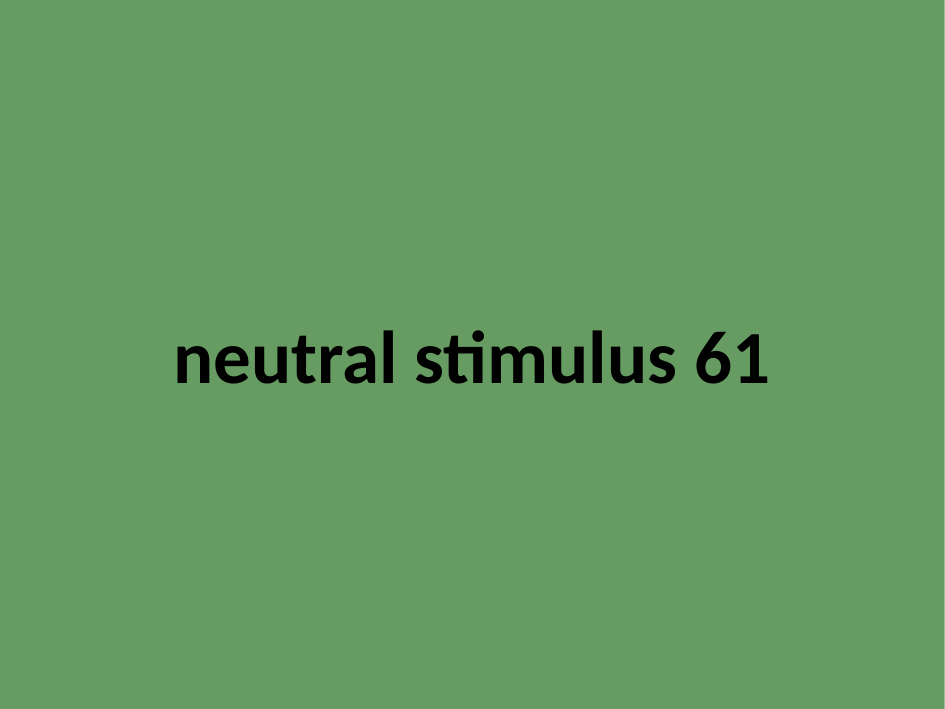

neutral stimulus 61

## Slide 62
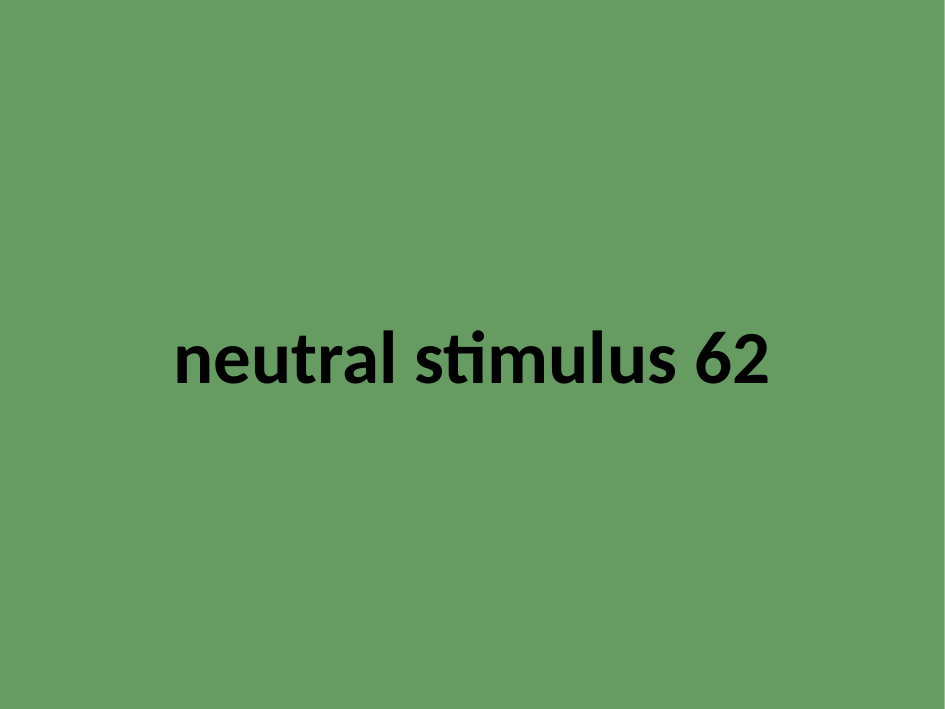

neutral stimulus 62

## Slide 63
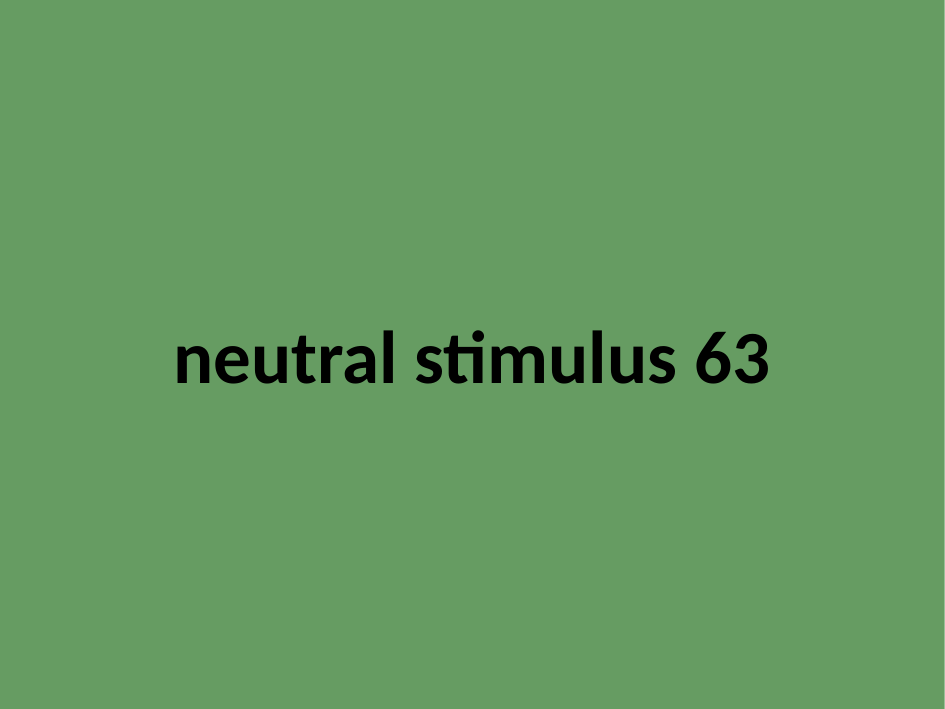

neutral stimulus 63

## Slide 64
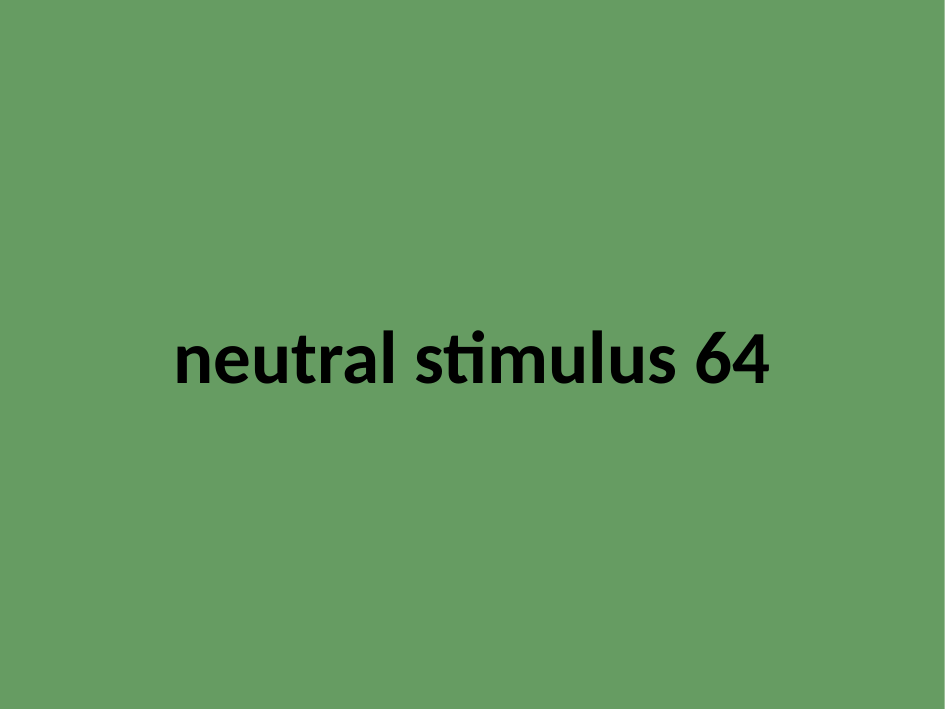

neutral stimulus 64

## Slide 65
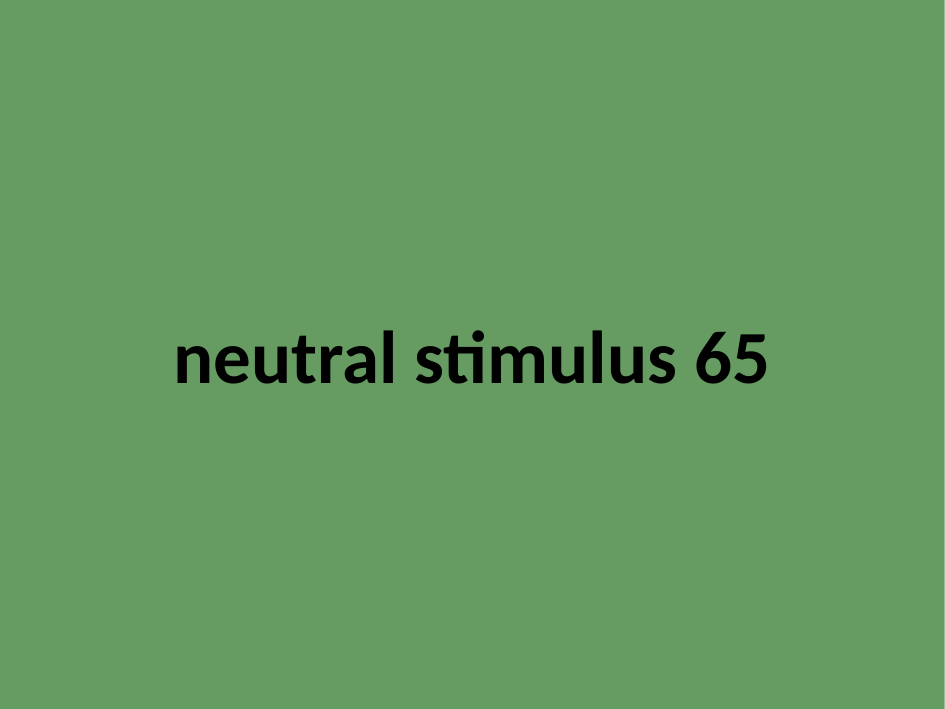

neutral stimulus 65

## Slide 66
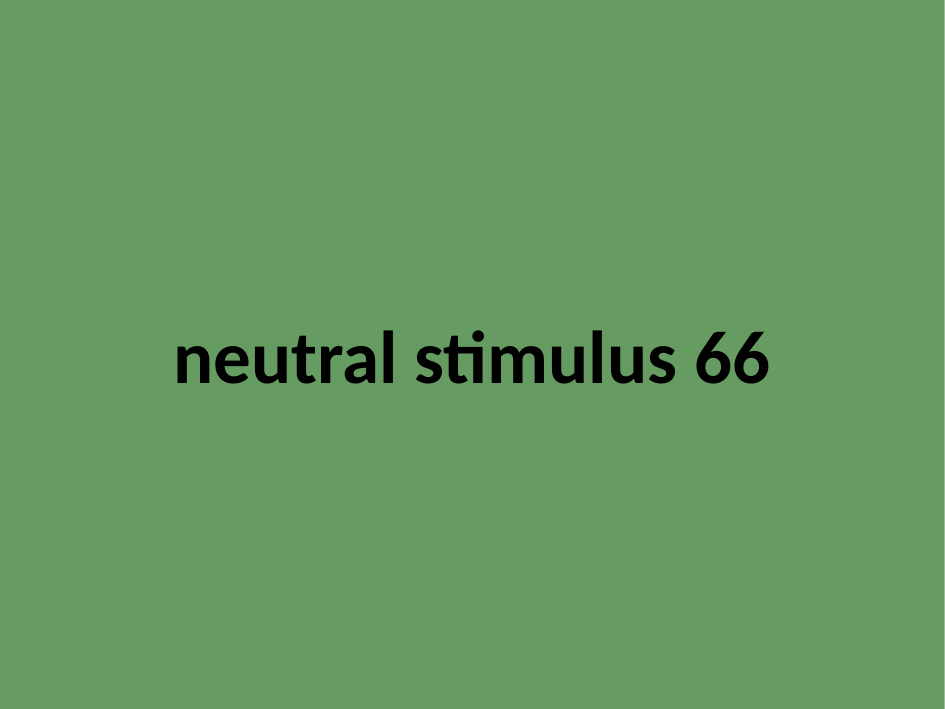

neutral stimulus 66

## Slide 67
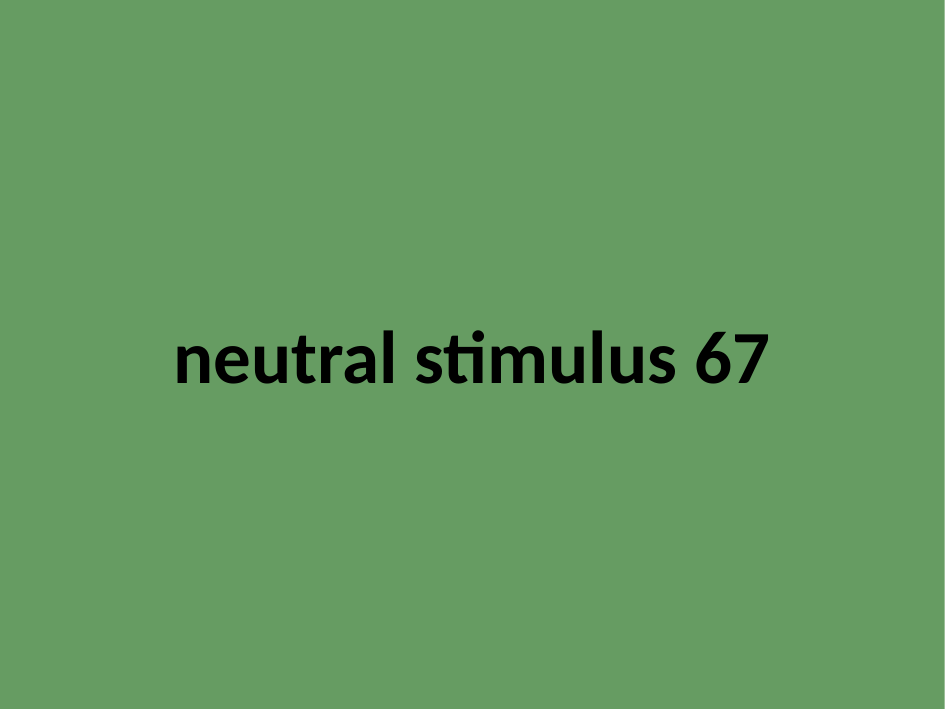

neutral stimulus 67

## Slide 68
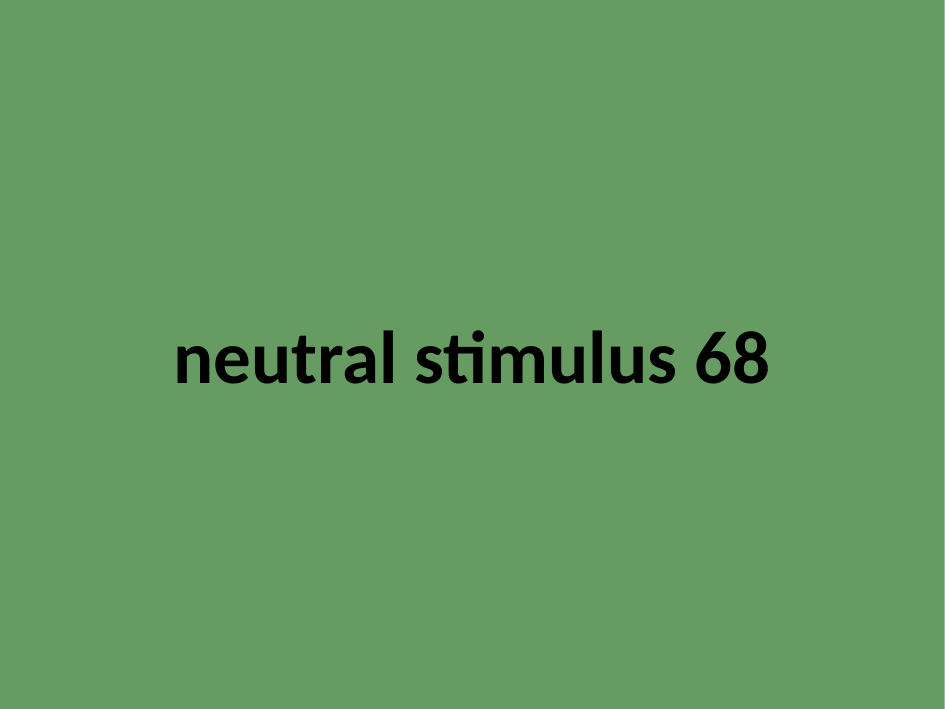

neutral stimulus 68

## Slide 69
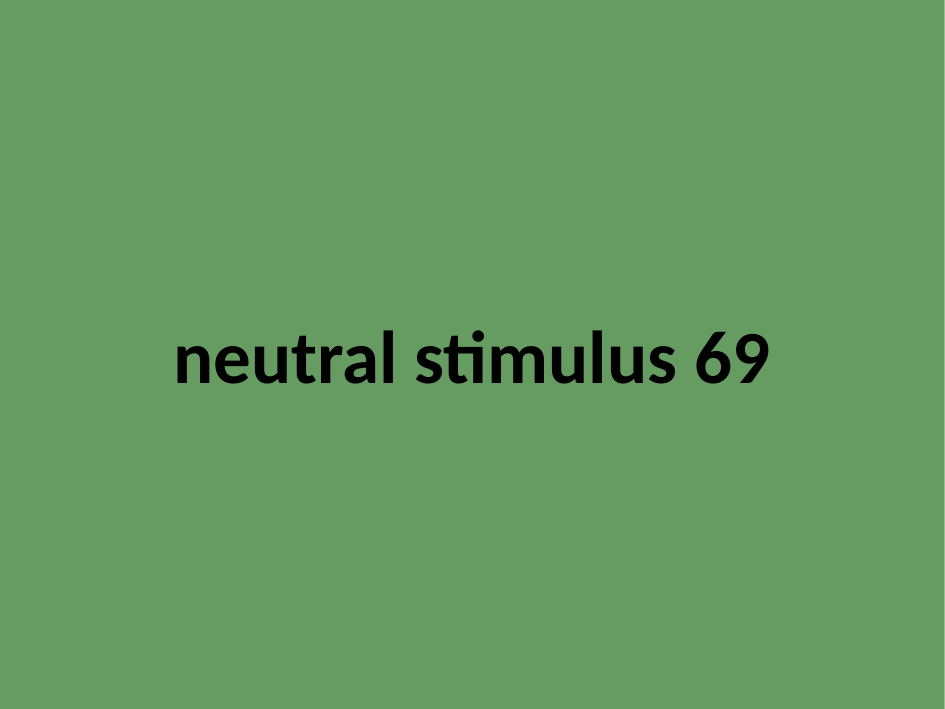

neutral stimulus 69

## Slide 70
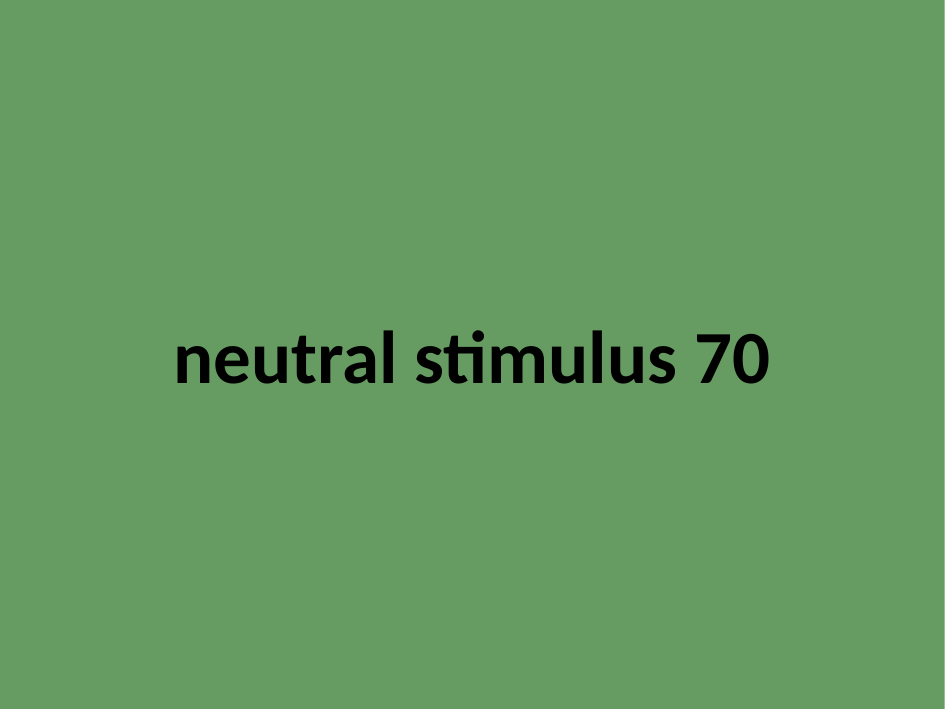

neutral stimulus 70
