# Supplementary material for: Detours increase local knowledge—Exploring the hidden benefits of self-control failure
Source: PLoS One. 2021 Oct 1;16(10):e0257717. doi: 10.1371/journal.pone.0257717 (PMC8486128; doi:10.1371/journal.pone.0257717)
Supplement: S2 File — (ZIP) [file pone.0257717.s002.zip › software/stimuli/stimuli_positive.pptx]

## Slide 1
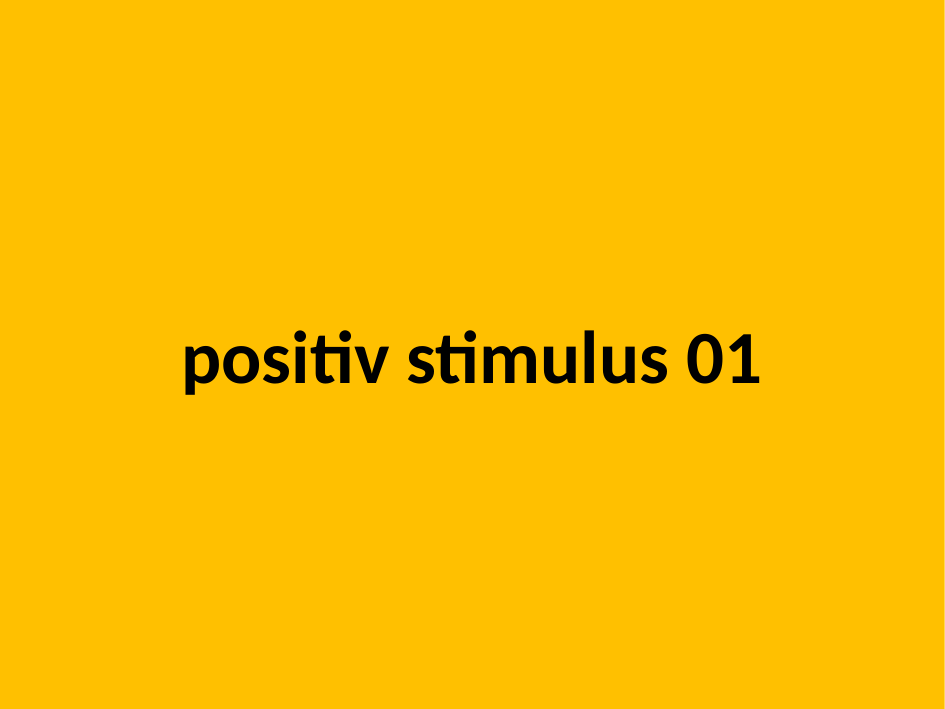

positiv stimulus 01

## Slide 2
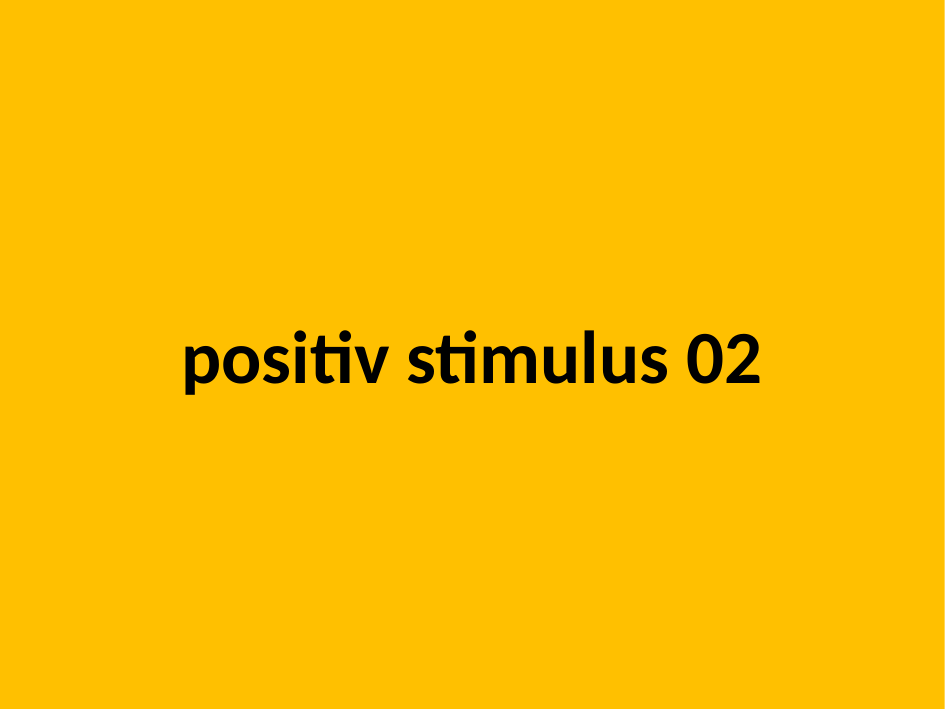

positiv stimulus 02

## Slide 3
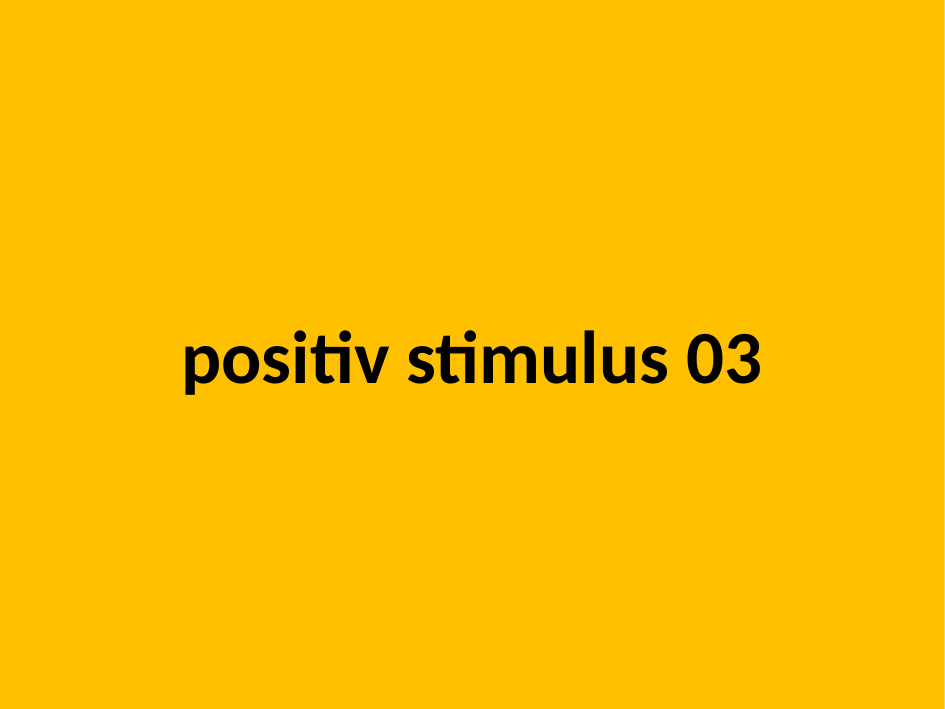

positiv stimulus 03

## Slide 4
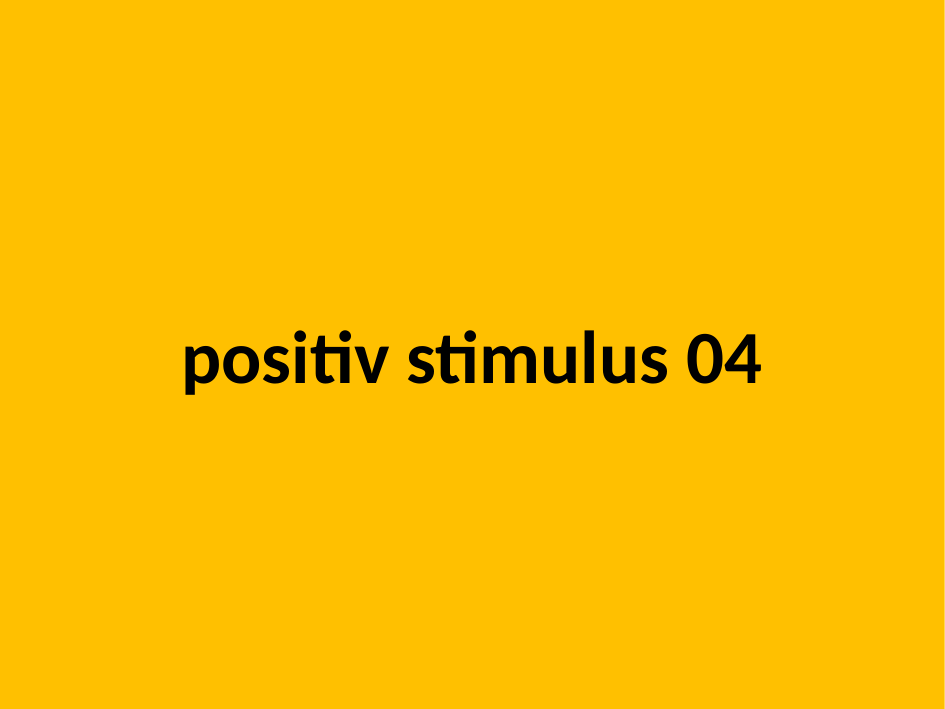

positiv stimulus 04

## Slide 5
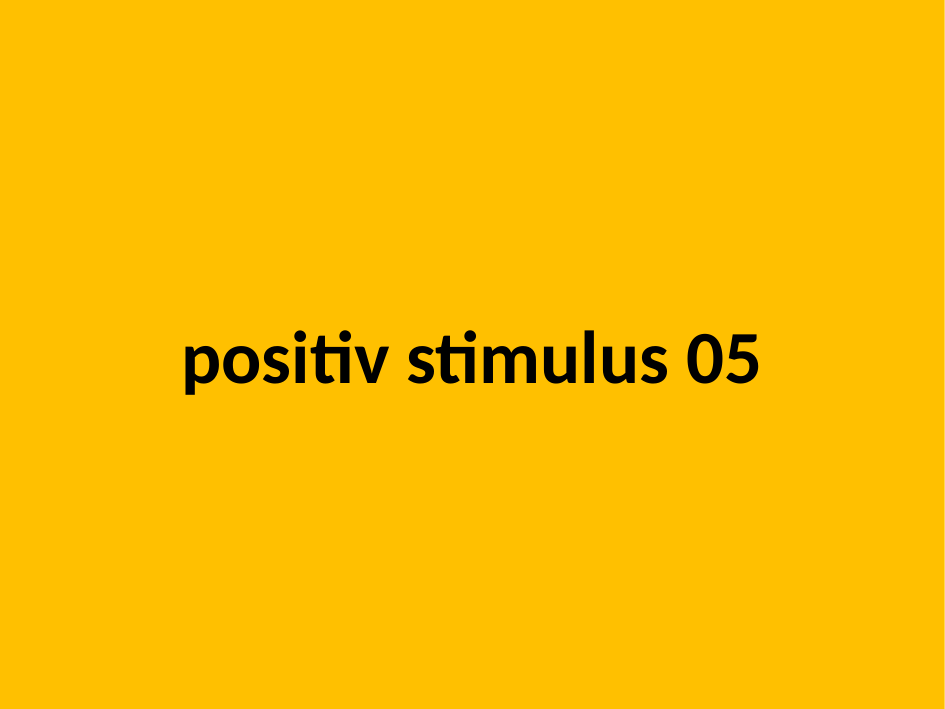

positiv stimulus 05

## Slide 6
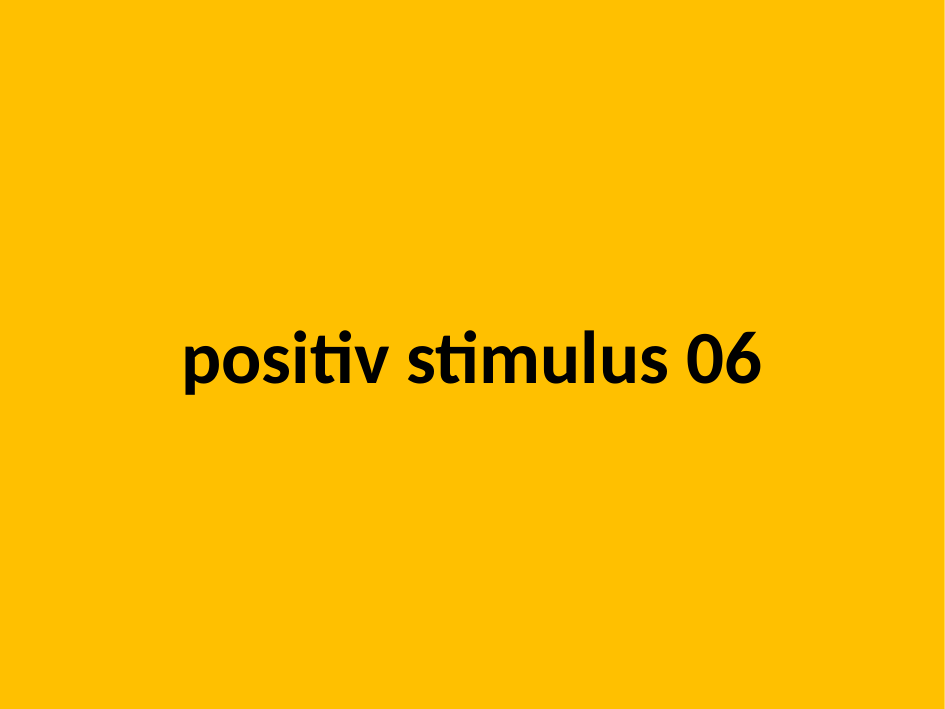

positiv stimulus 06

## Slide 7
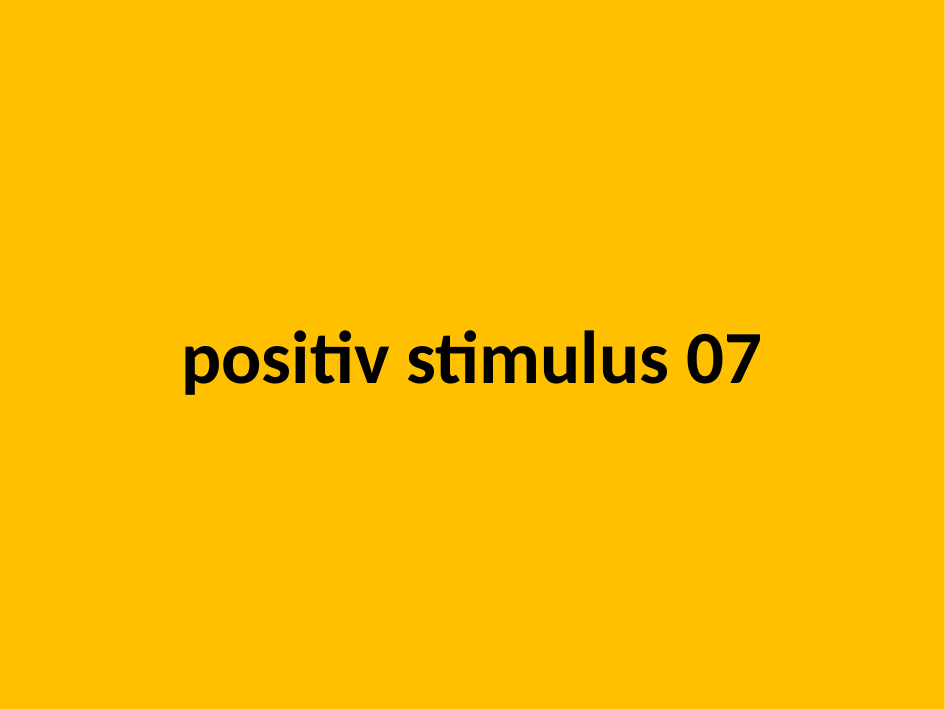

positiv stimulus 07

## Slide 8
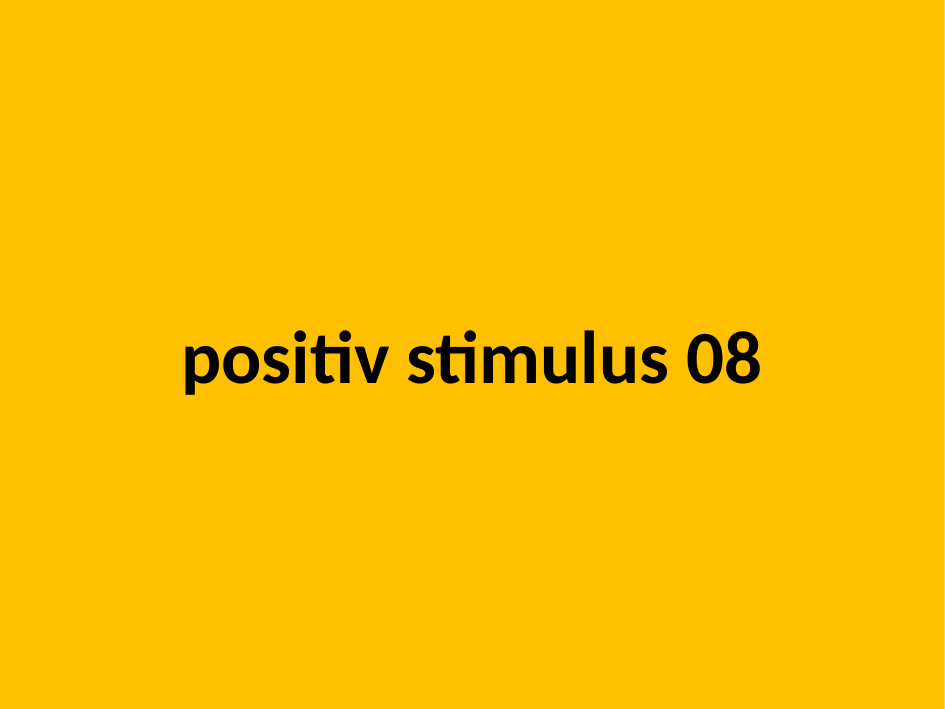

positiv stimulus 08

## Slide 9
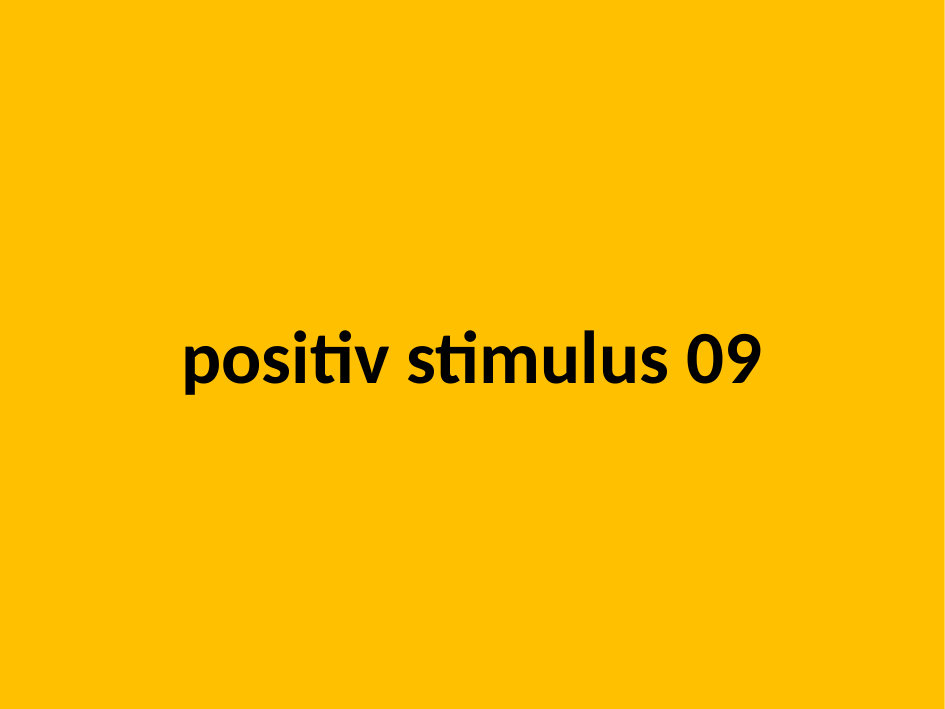

positiv stimulus 09

## Slide 10
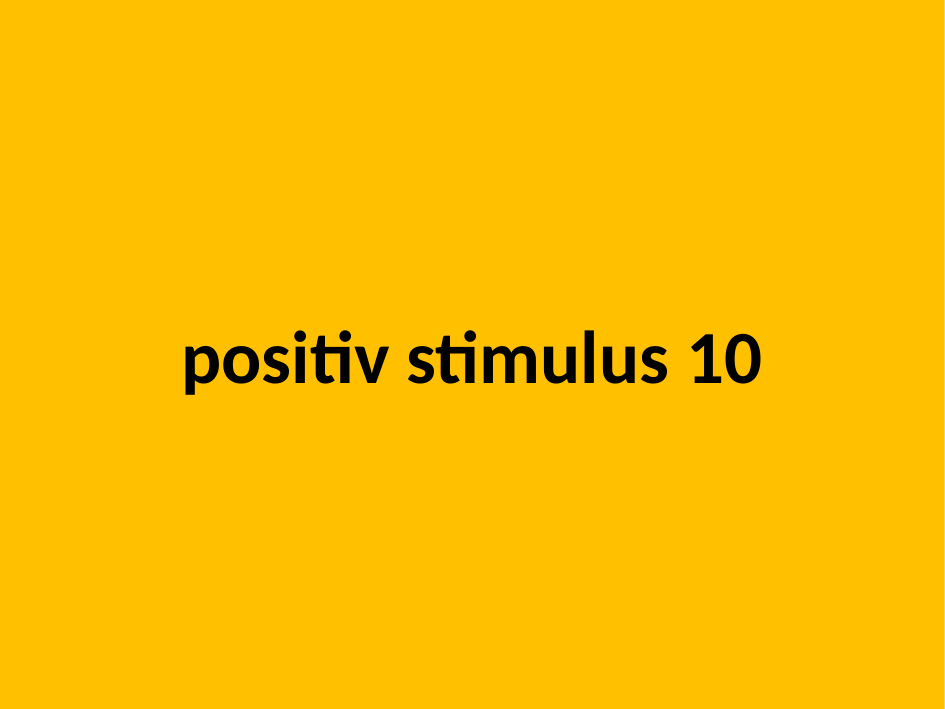

positiv stimulus 10

## Slide 11
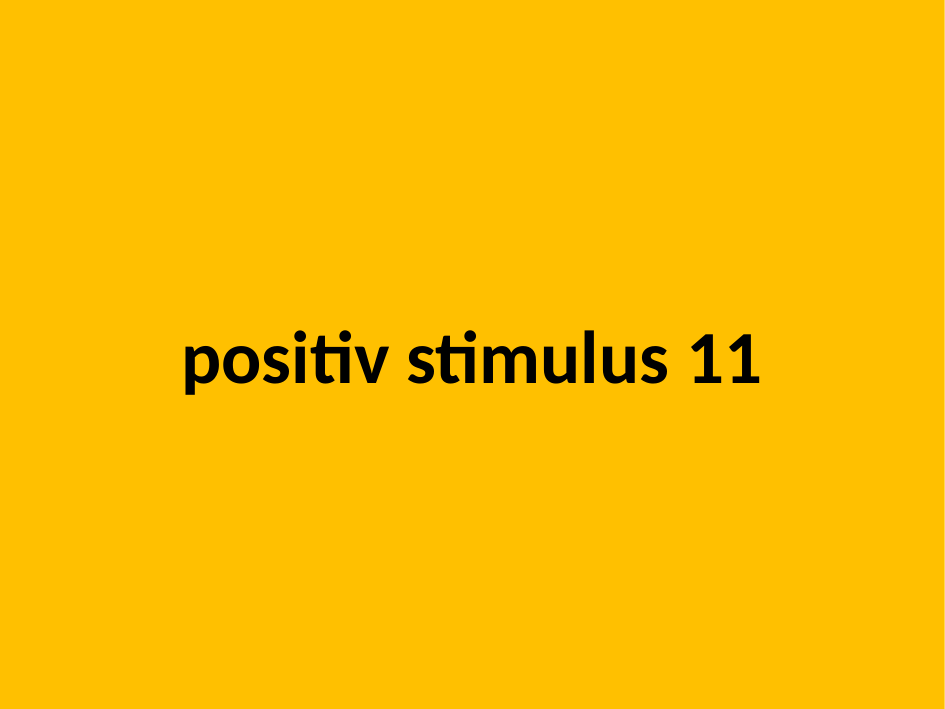

positiv stimulus 11

## Slide 12
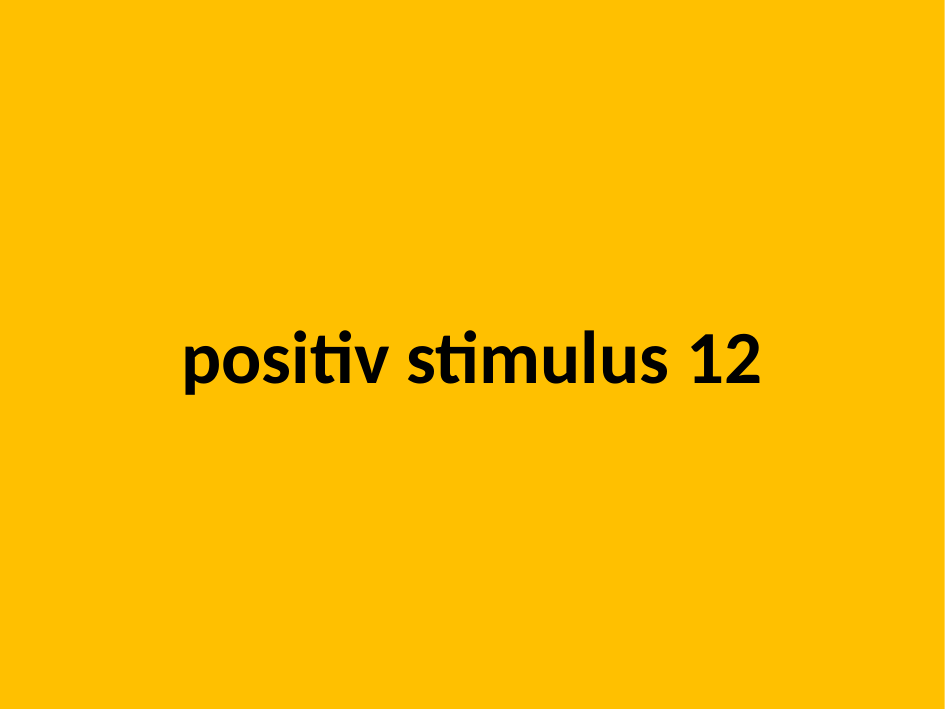

positiv stimulus 12

## Slide 13
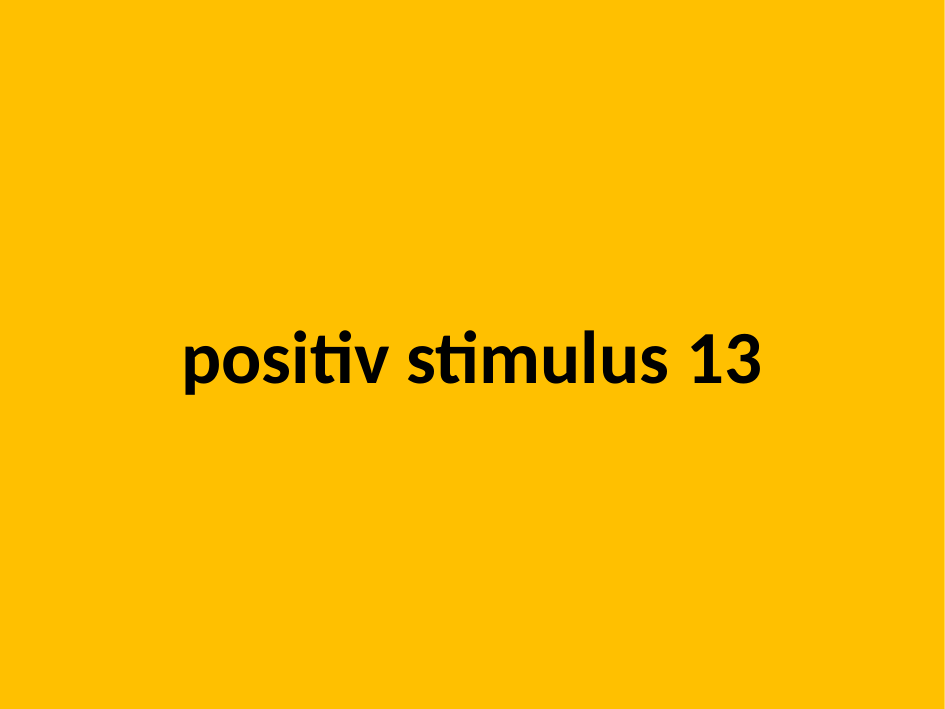

positiv stimulus 13

## Slide 14
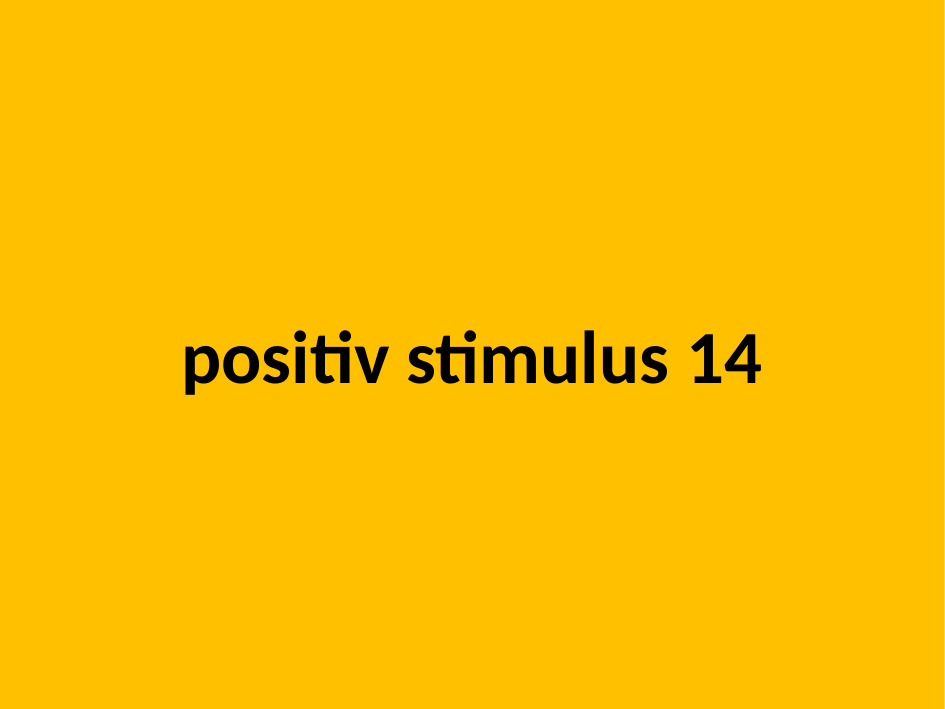

positiv stimulus 14

## Slide 15
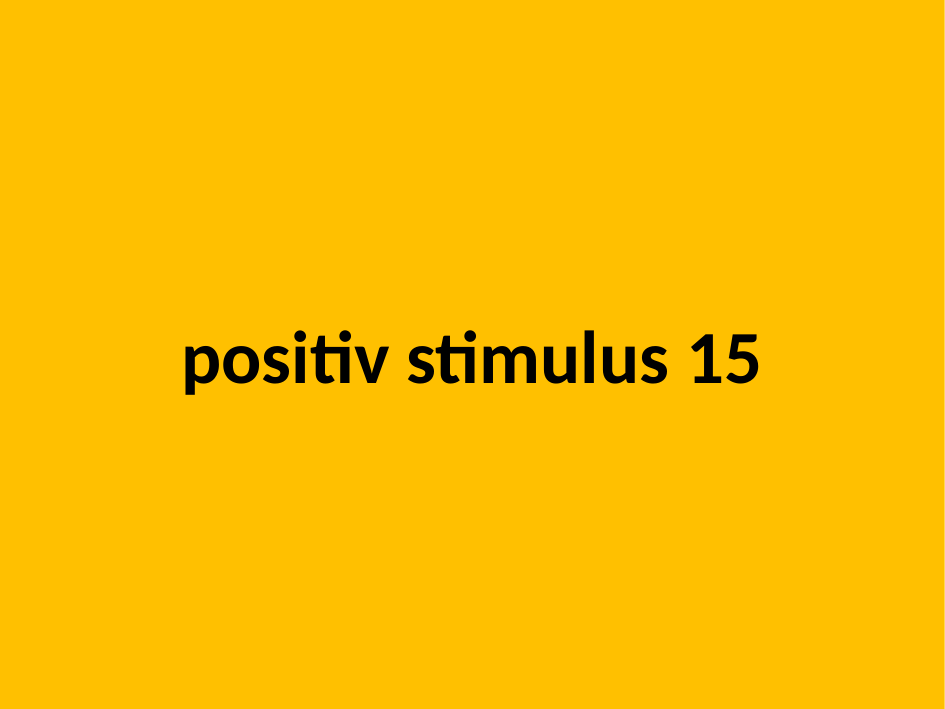

positiv stimulus 15

## Slide 16
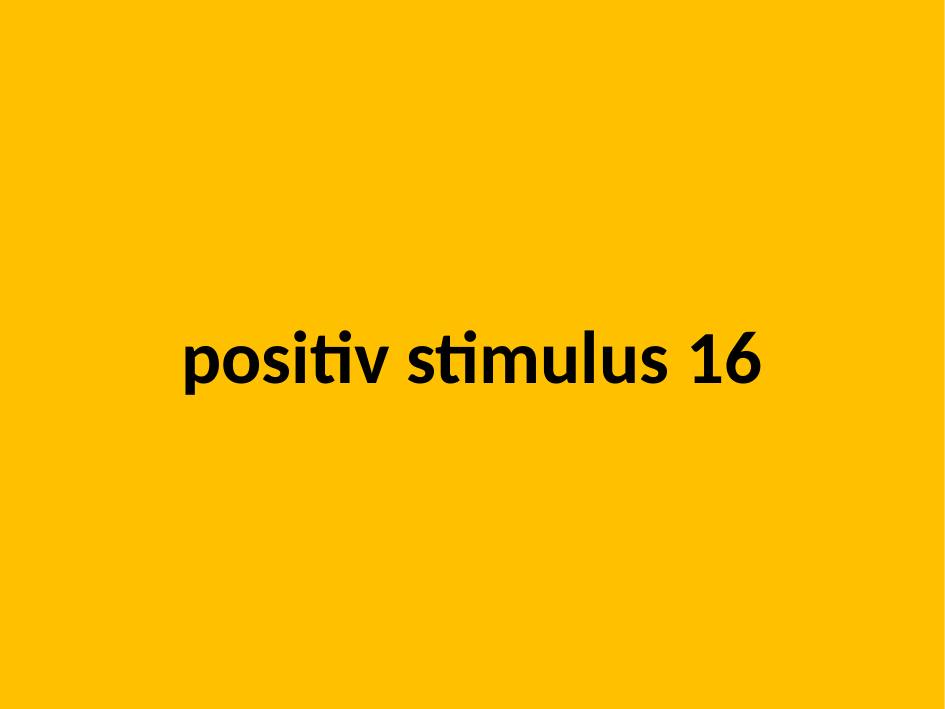

positiv stimulus 16

## Slide 17
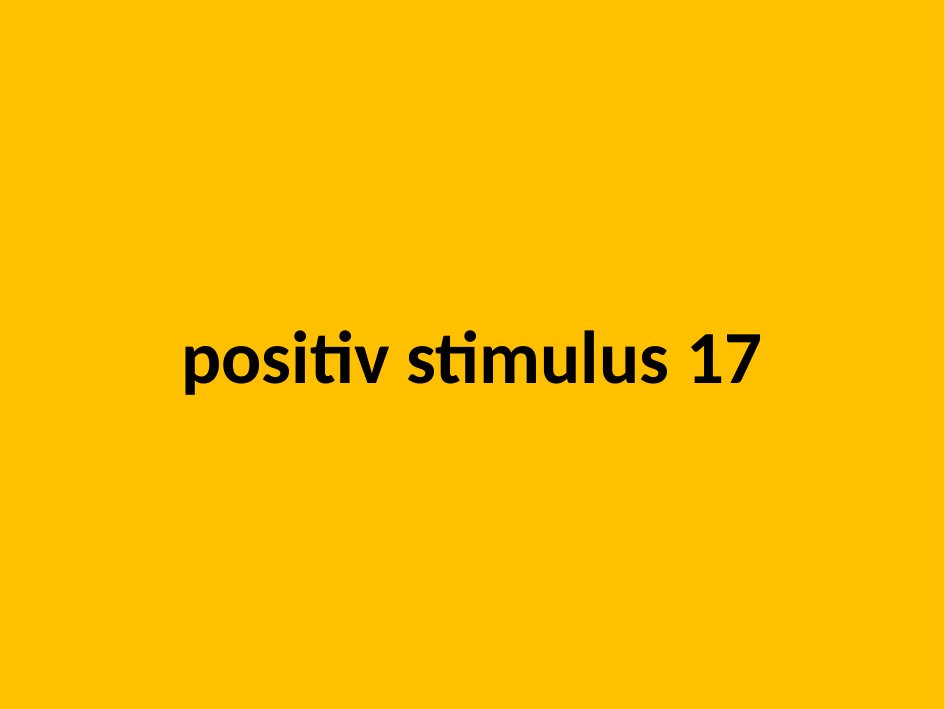

positiv stimulus 17

## Slide 18
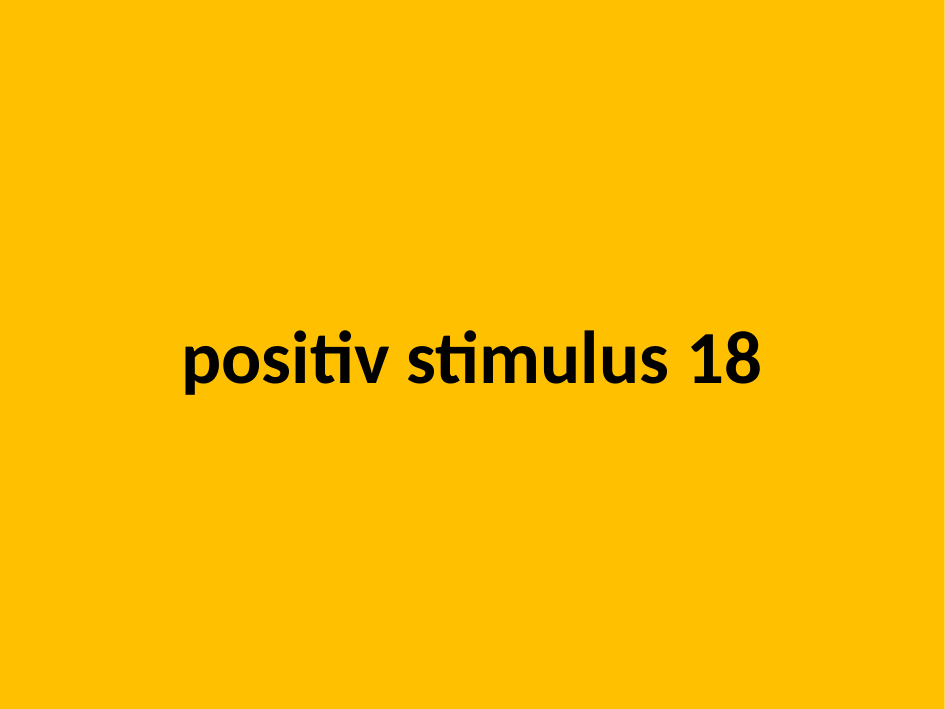

positiv stimulus 18

## Slide 19
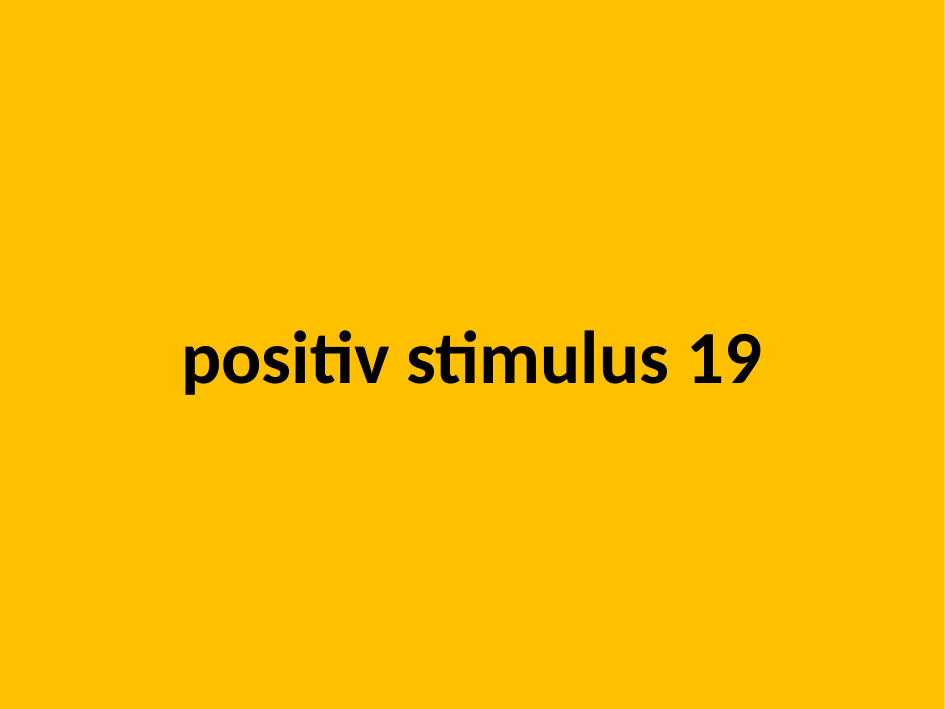

positiv stimulus 19

## Slide 20
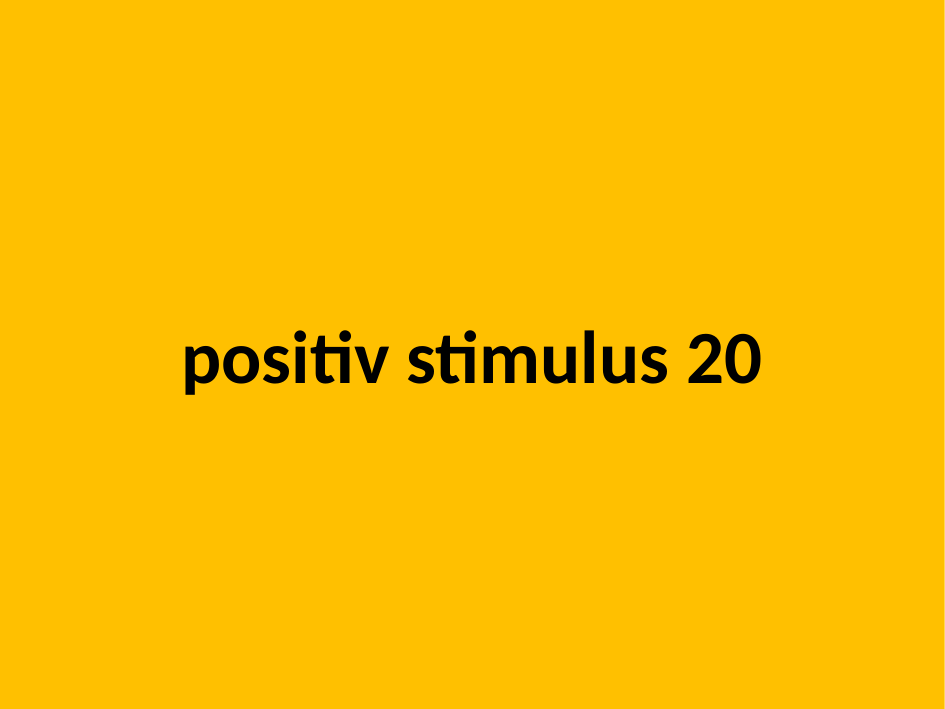

positiv stimulus 20

## Slide 21
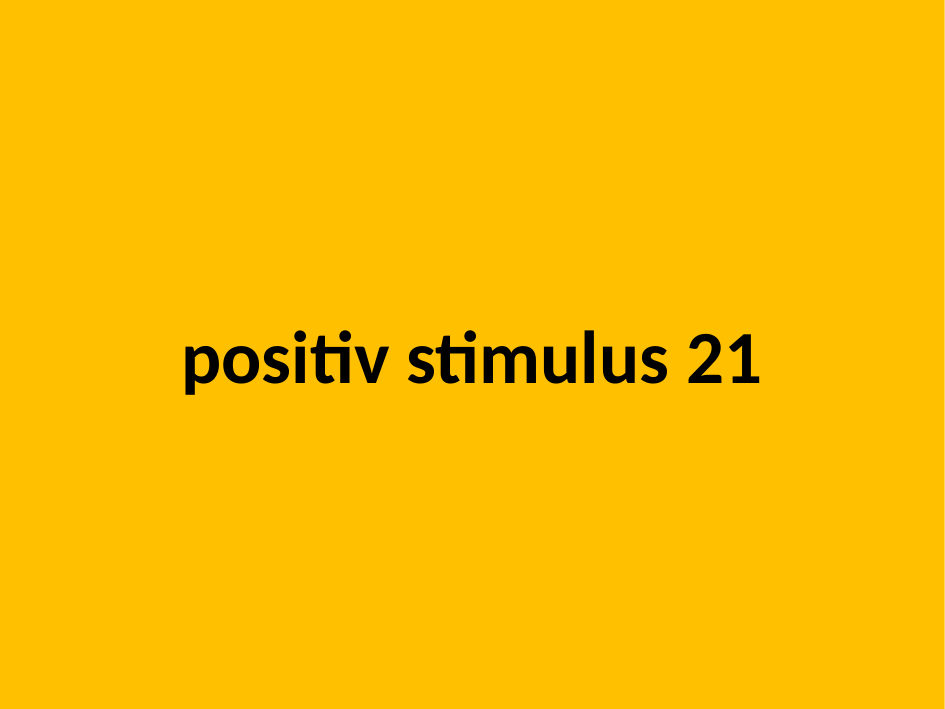

positiv stimulus 21

## Slide 22
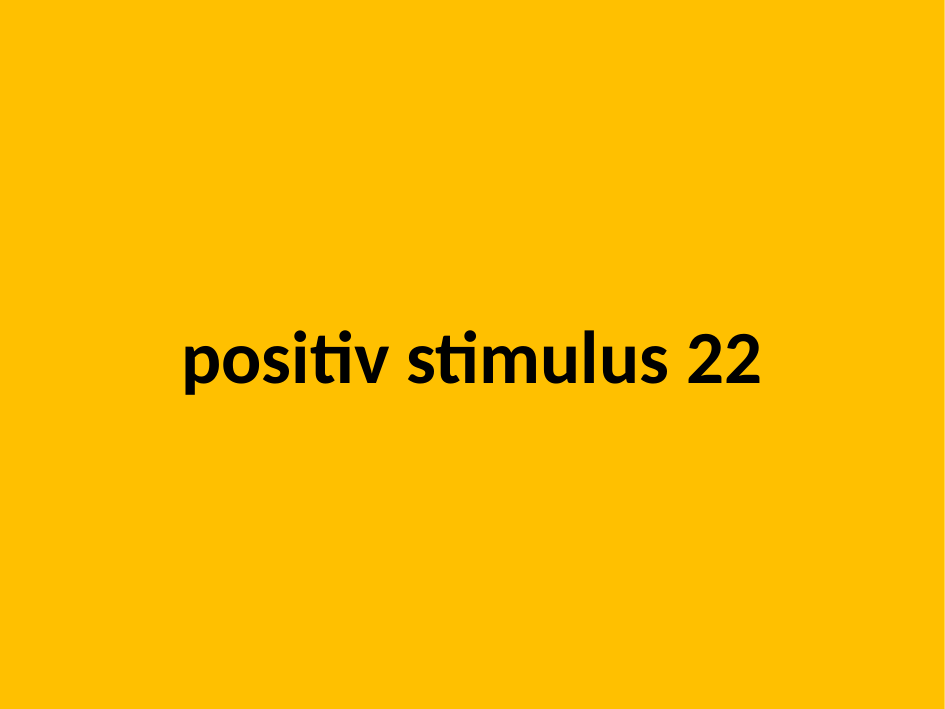

positiv stimulus 22

## Slide 23
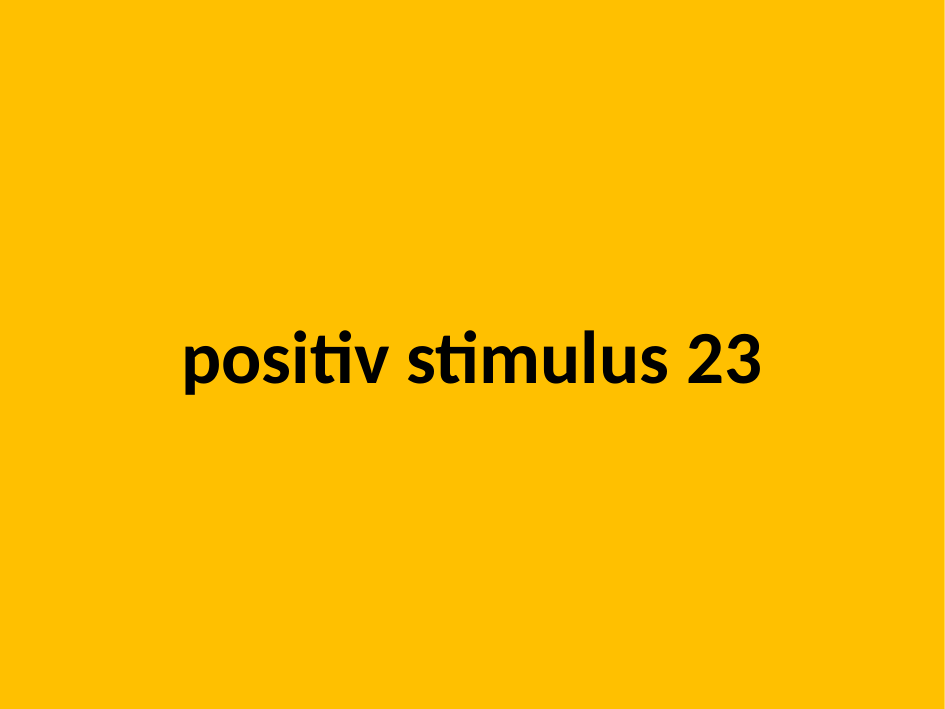

positiv stimulus 23

## Slide 24
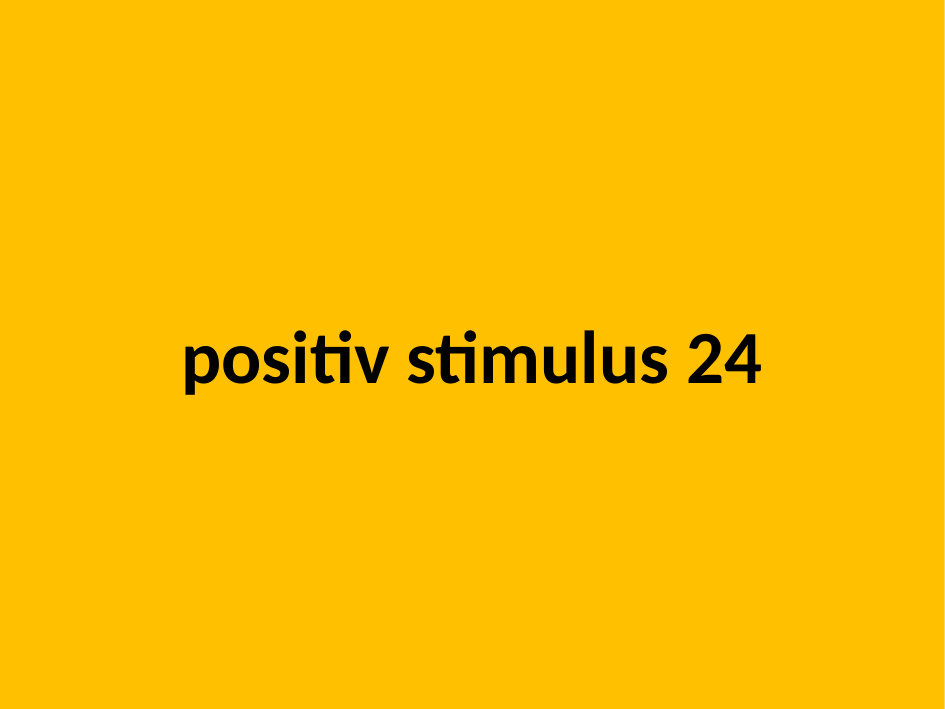

positiv stimulus 24

## Slide 25
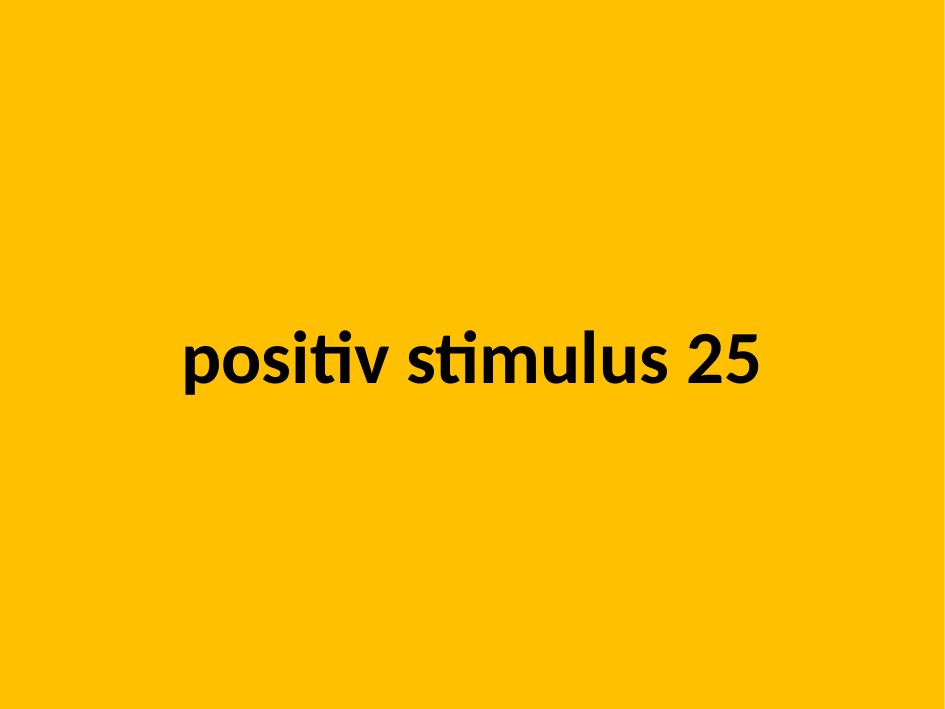

positiv stimulus 25

## Slide 26
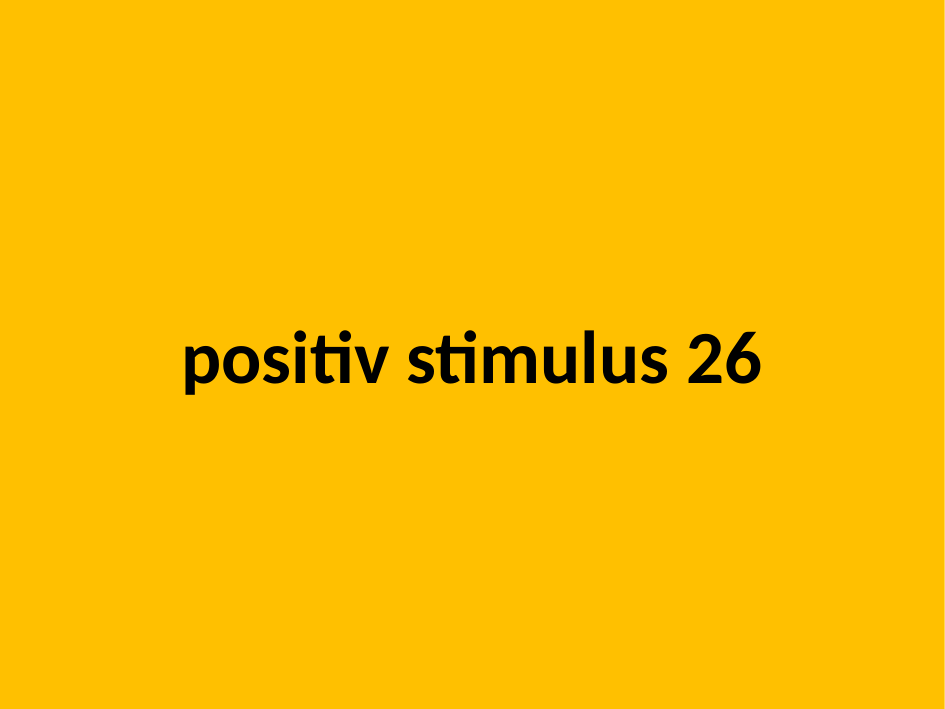

positiv stimulus 26

## Slide 27
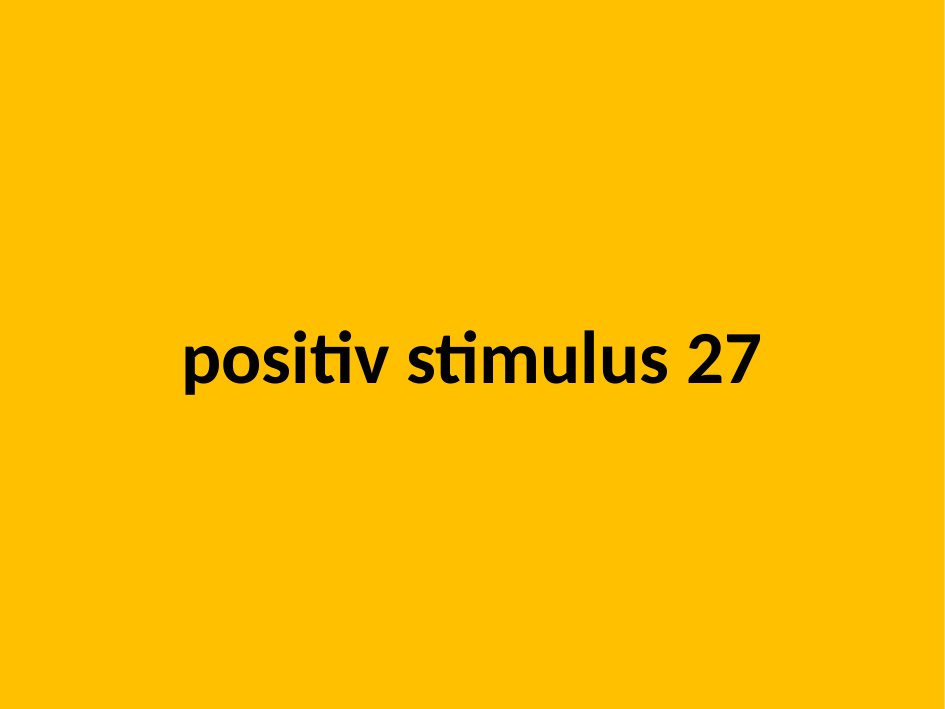

positiv stimulus 27

## Slide 28
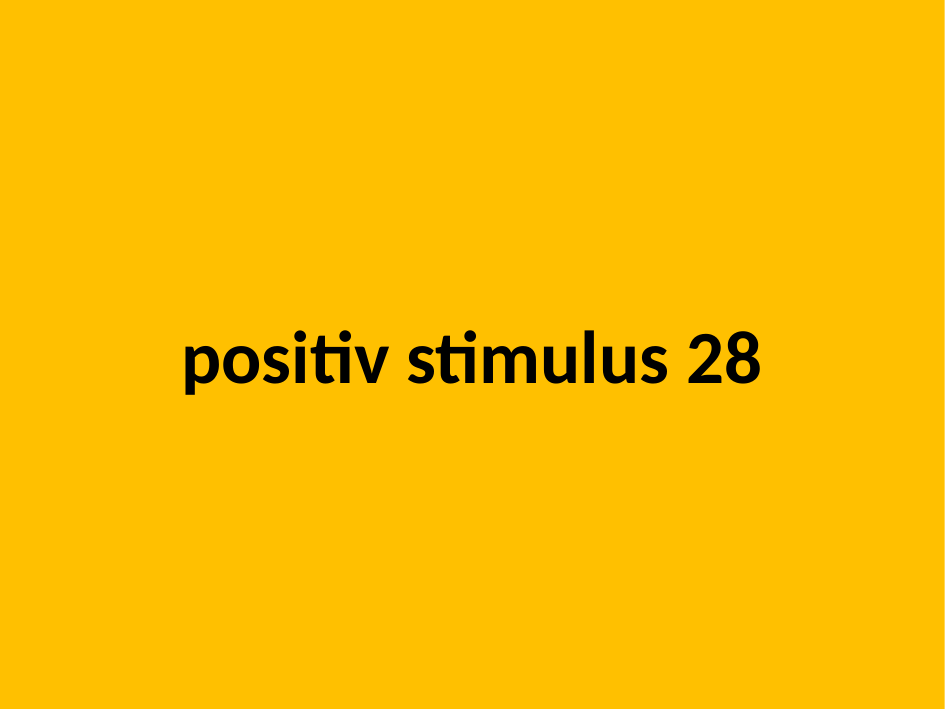

positiv stimulus 28

## Slide 29
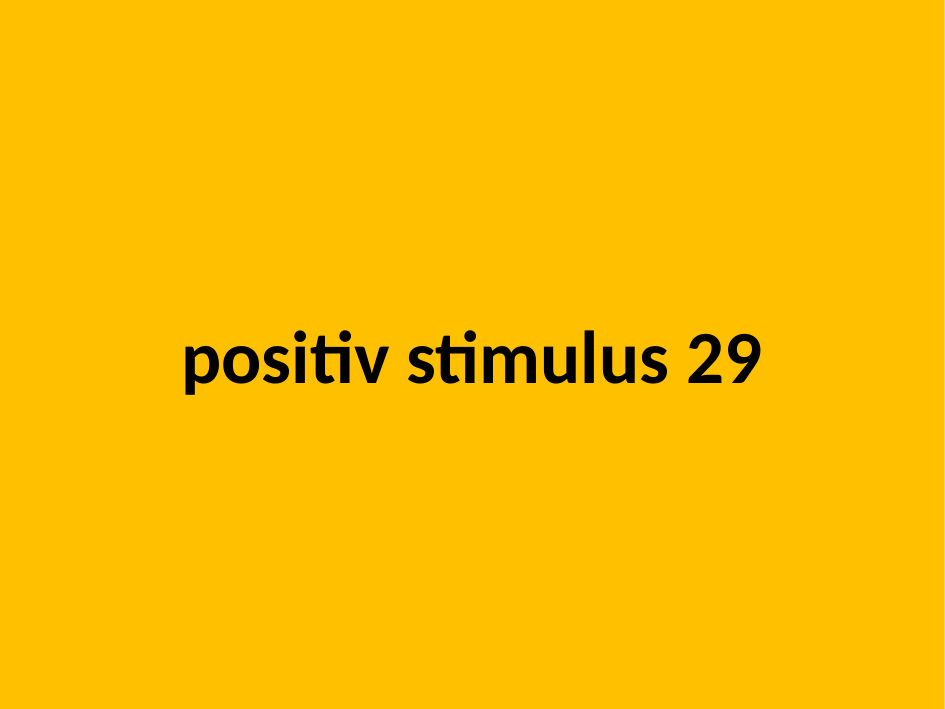

positiv stimulus 29

## Slide 30
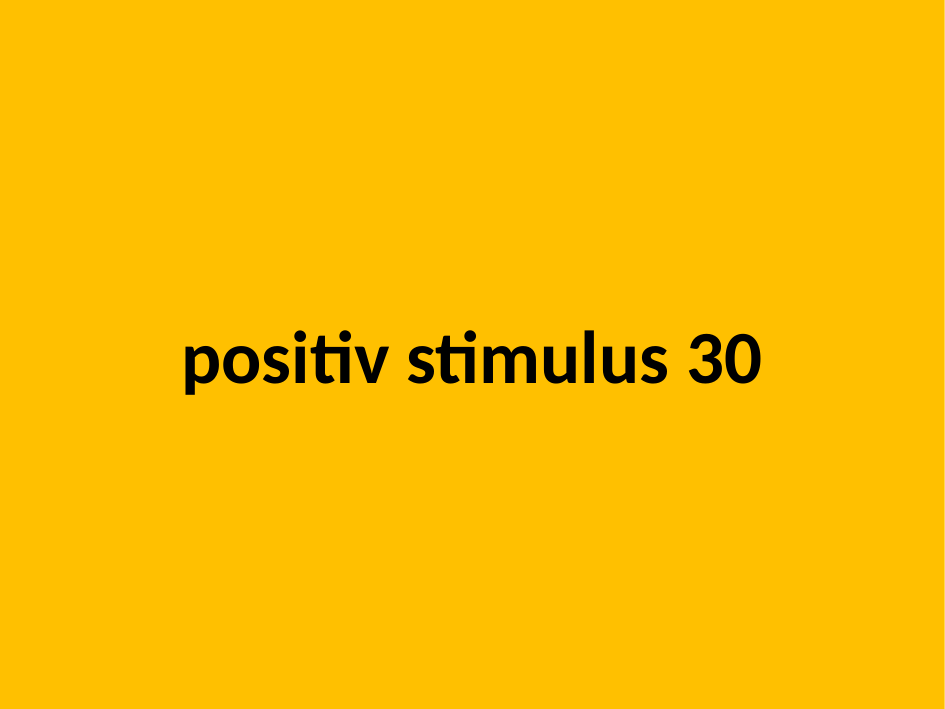

positiv stimulus 30

## Slide 31
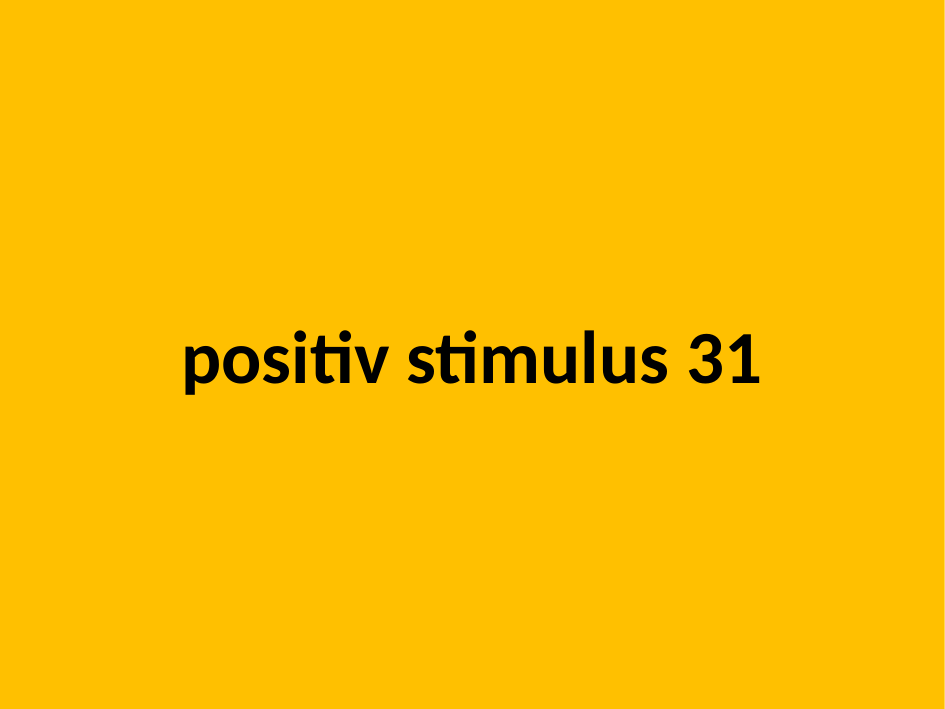

positiv stimulus 31

## Slide 32
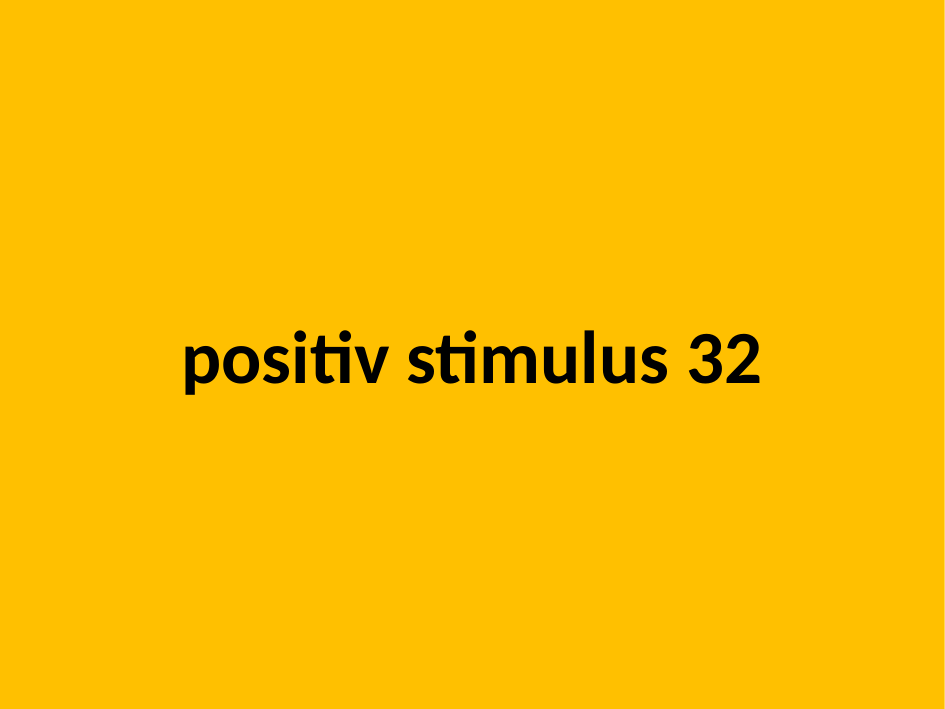

positiv stimulus 32

## Slide 33
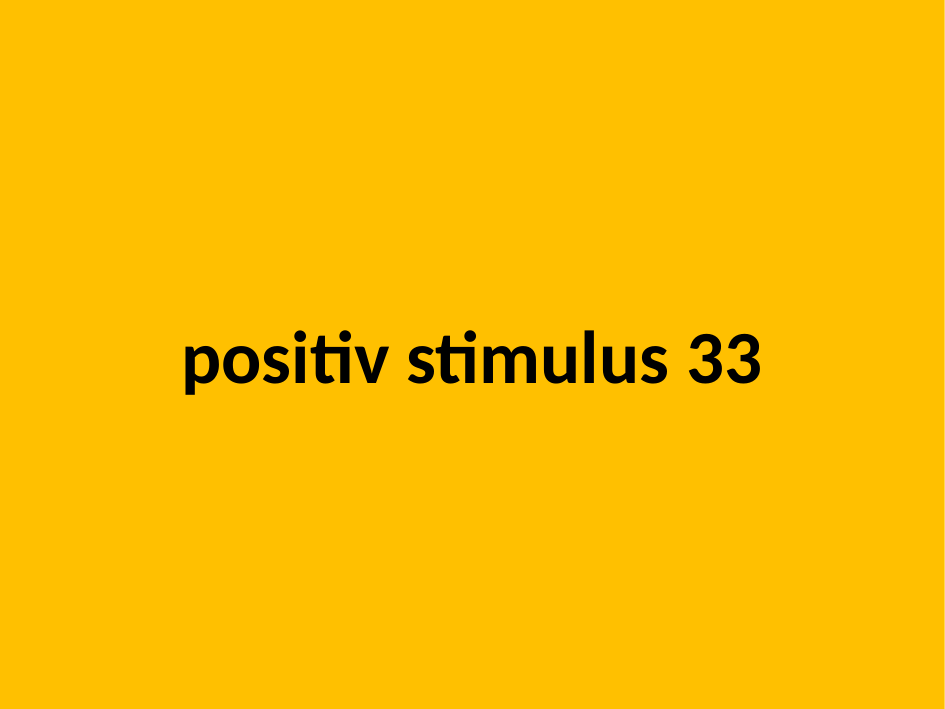

positiv stimulus 33

## Slide 34
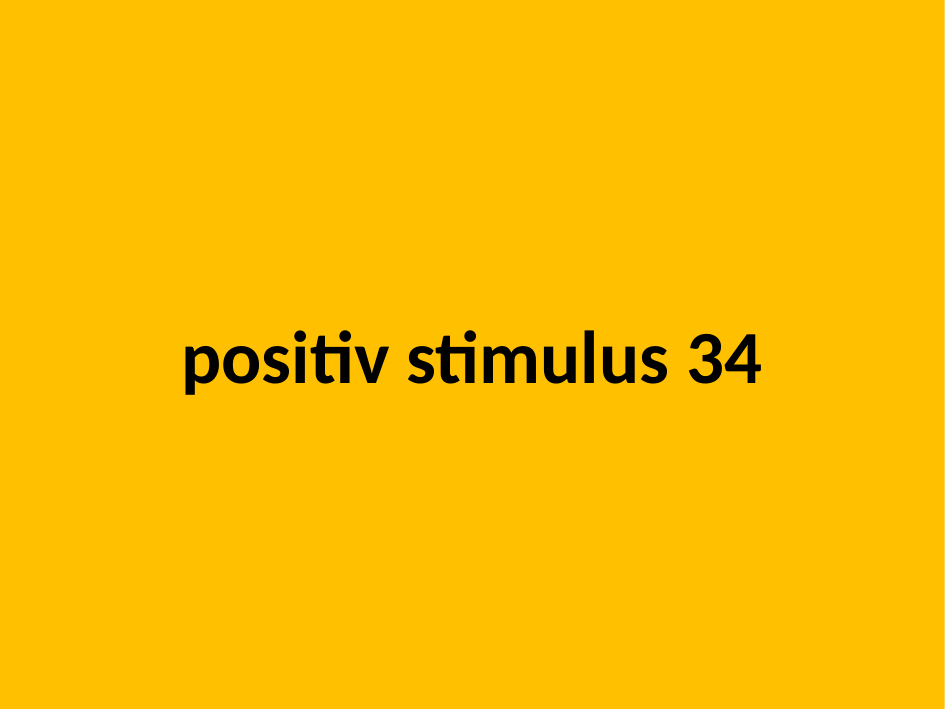

positiv stimulus 34

## Slide 35
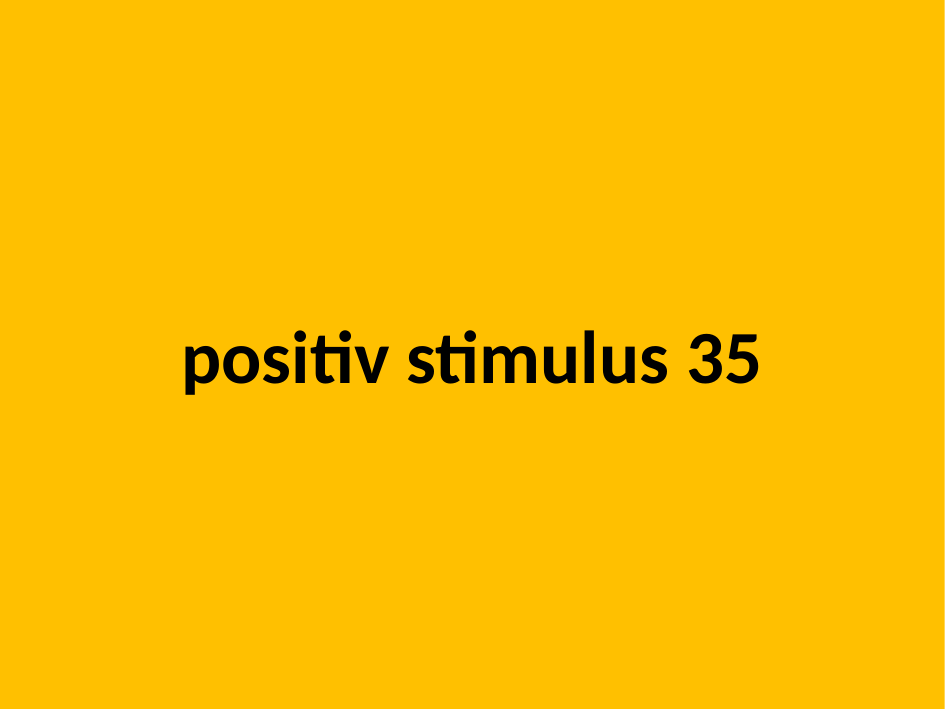

positiv stimulus 35

## Slide 36
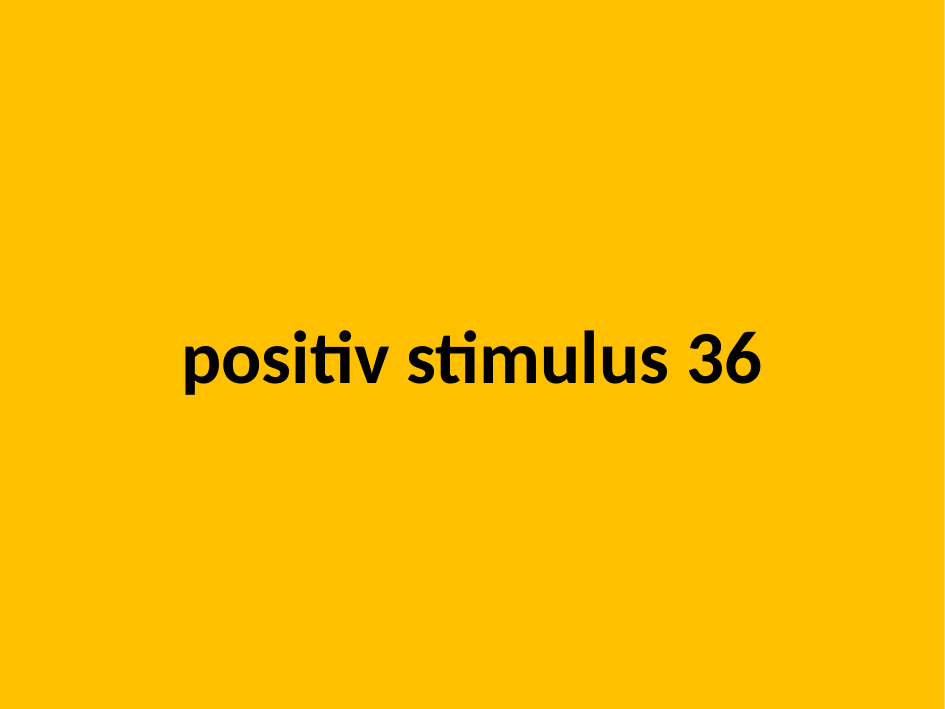

positiv stimulus 36

## Slide 37
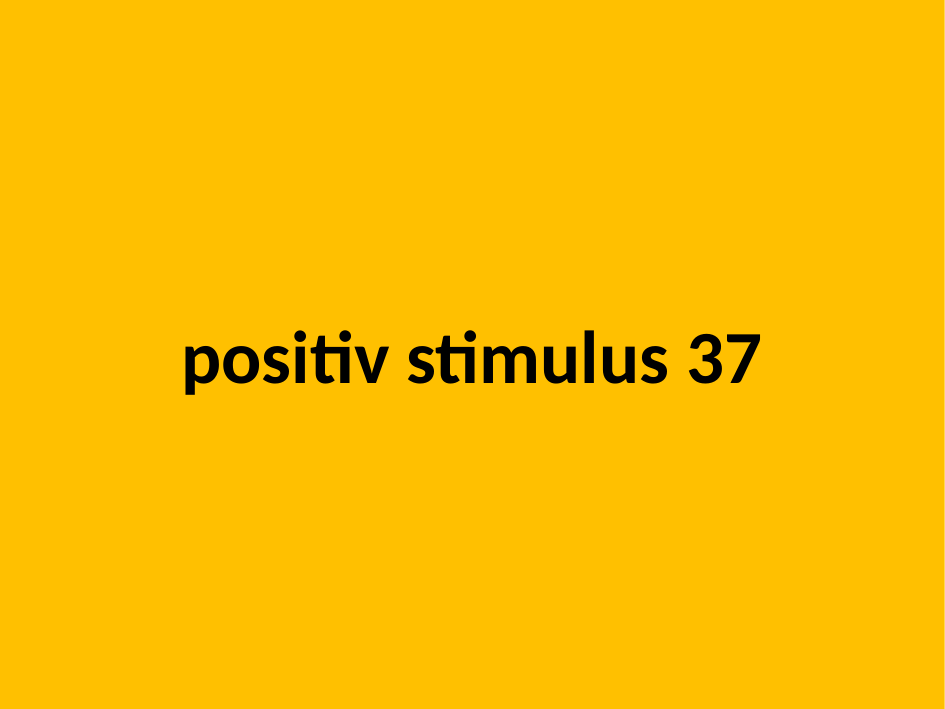

positiv stimulus 37

## Slide 38
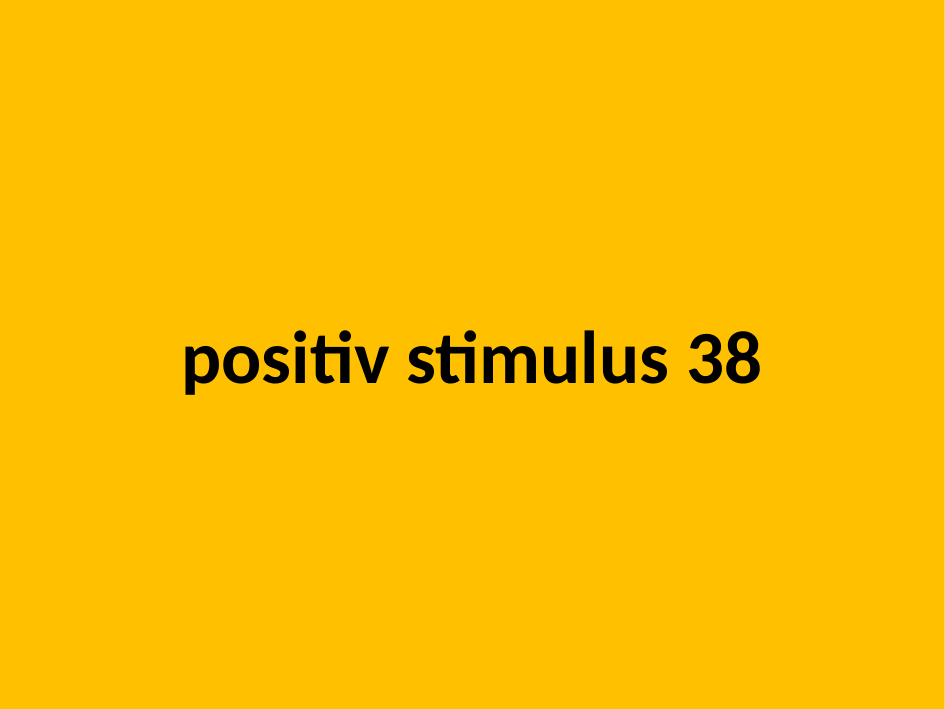

positiv stimulus 38

## Slide 39
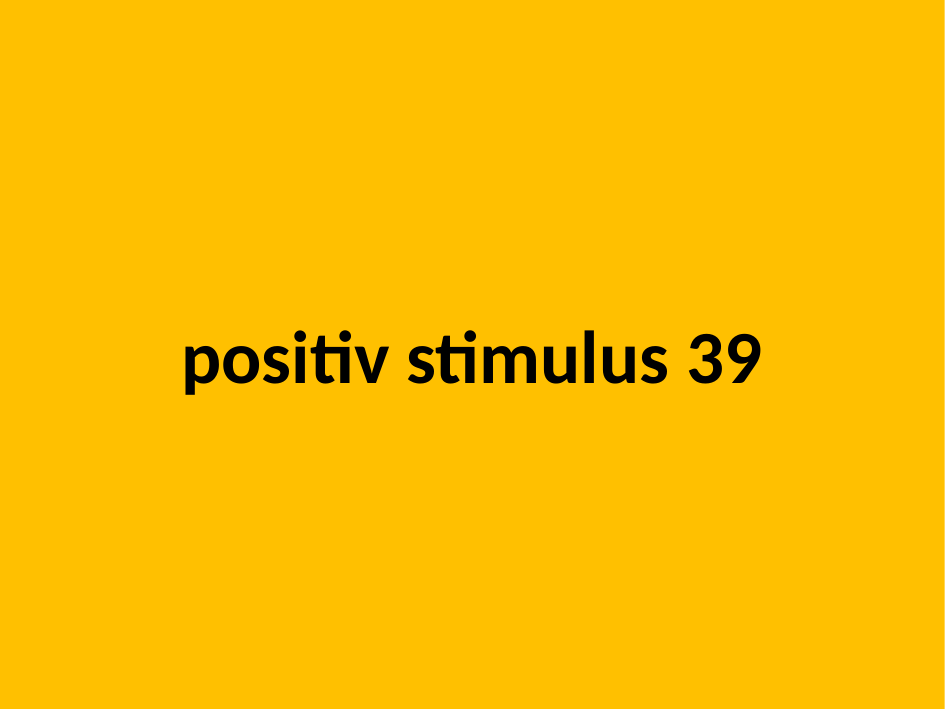

positiv stimulus 39

## Slide 40
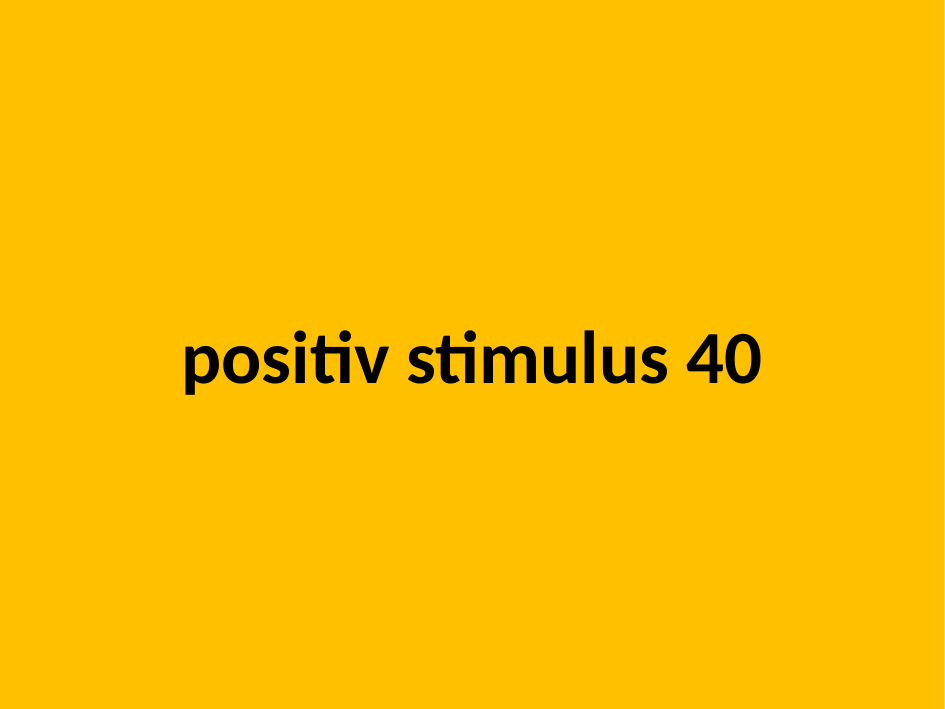

positiv stimulus 40

## Slide 41
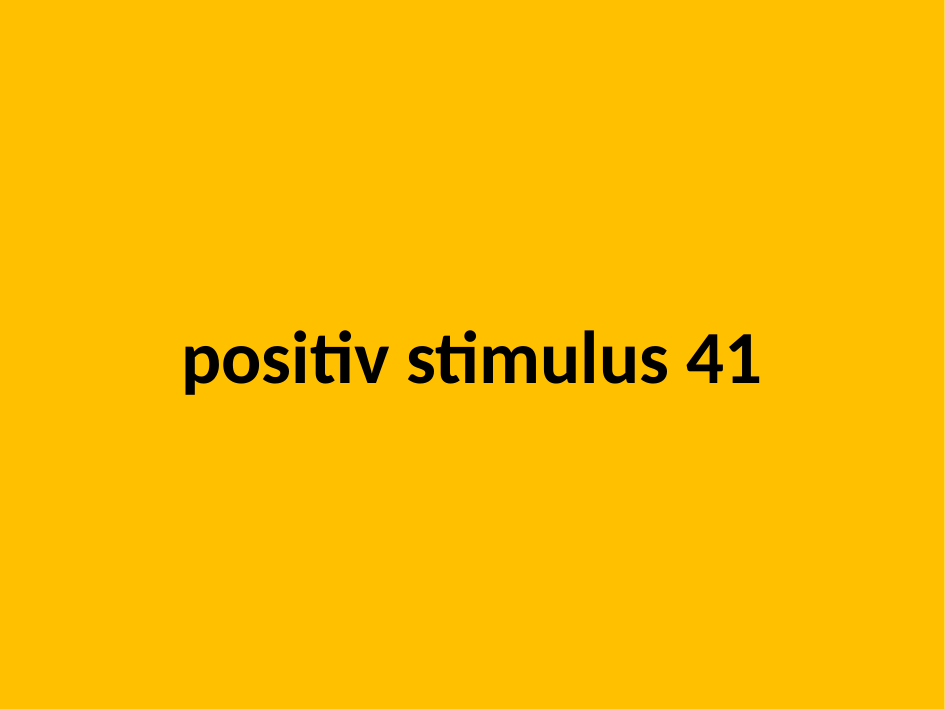

positiv stimulus 41

## Slide 42
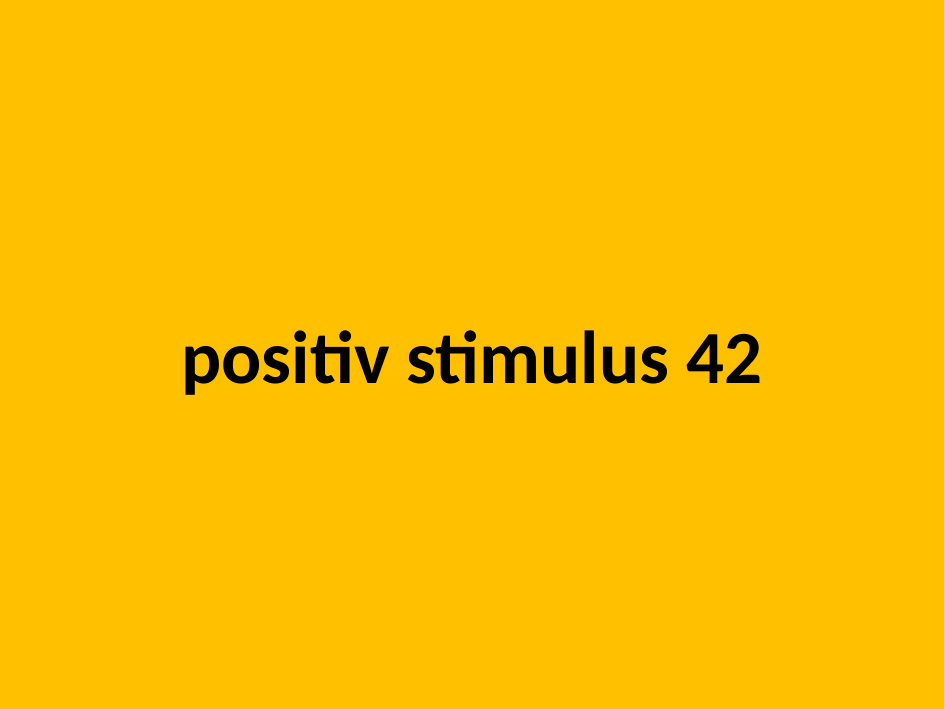

positiv stimulus 42

## Slide 43
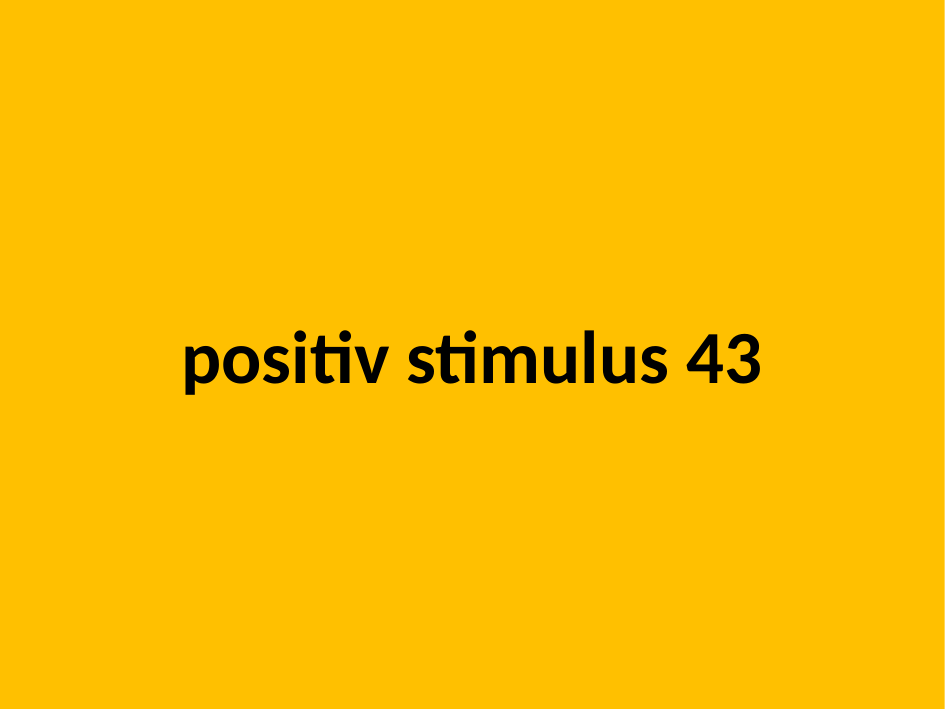

positiv stimulus 43

## Slide 44
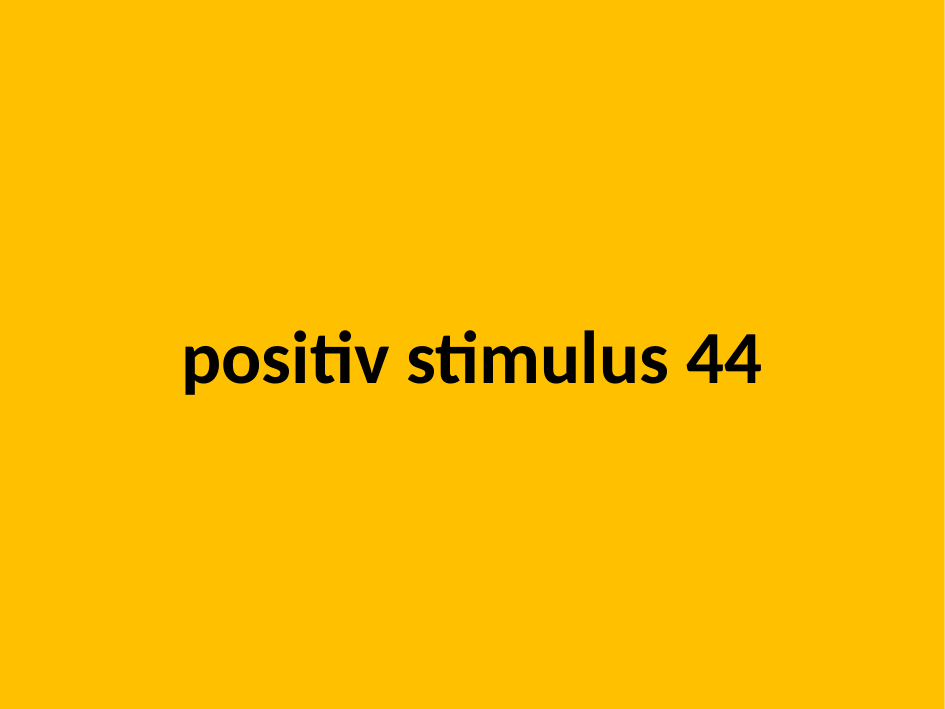

positiv stimulus 44

## Slide 45
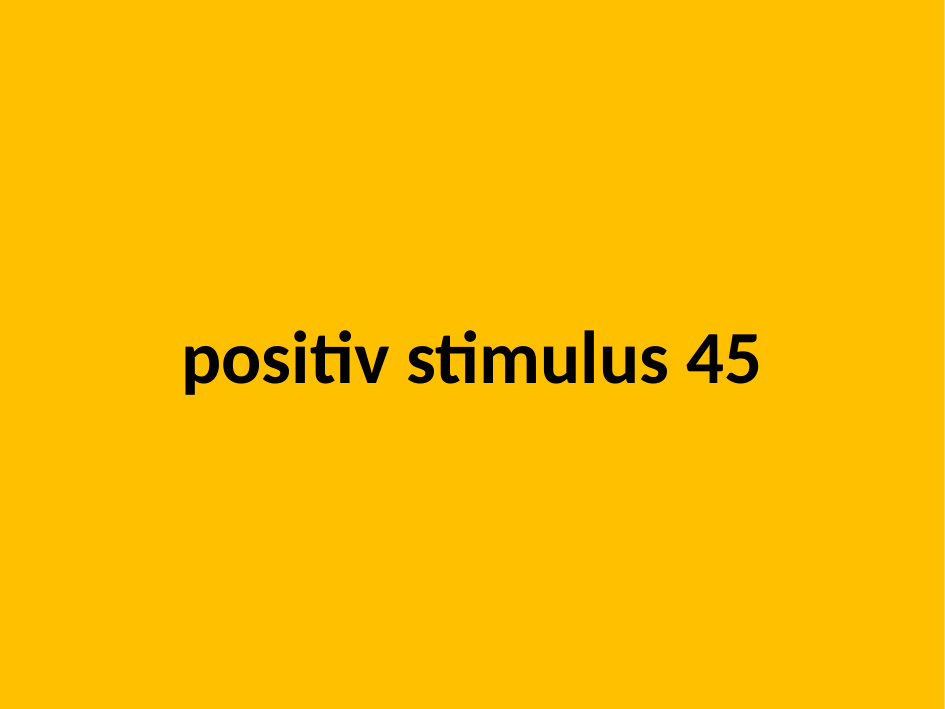

positiv stimulus 45

## Slide 46
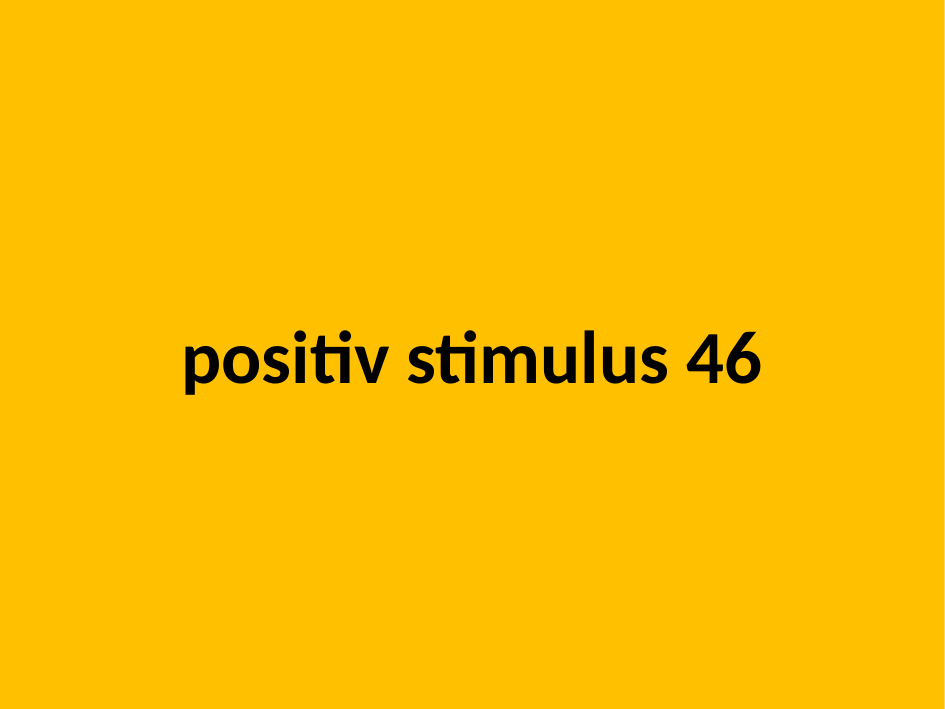

positiv stimulus 46

## Slide 47
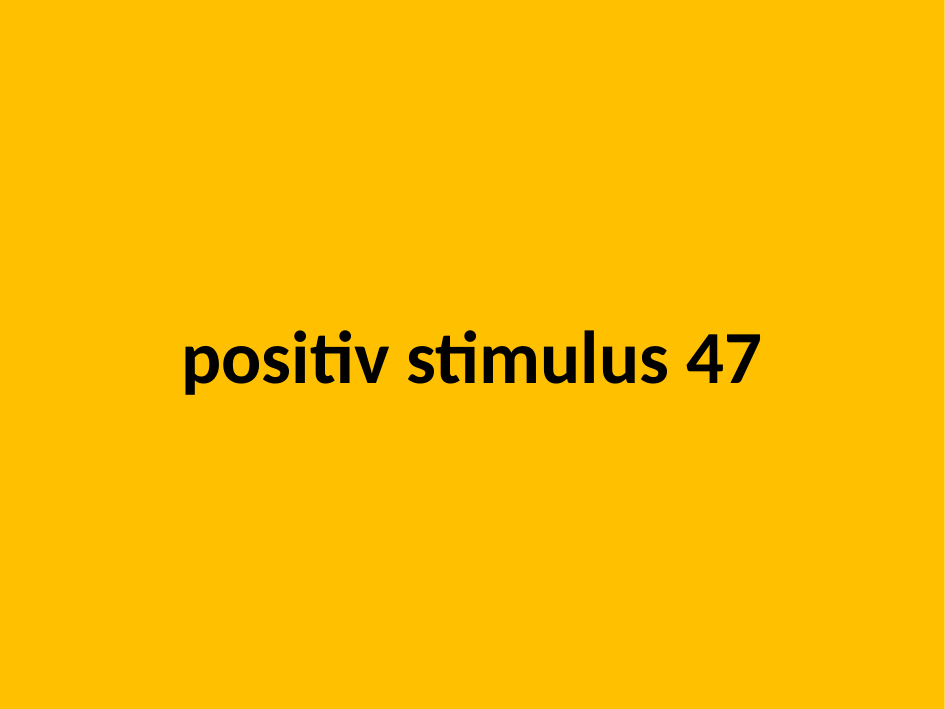

positiv stimulus 47

## Slide 48
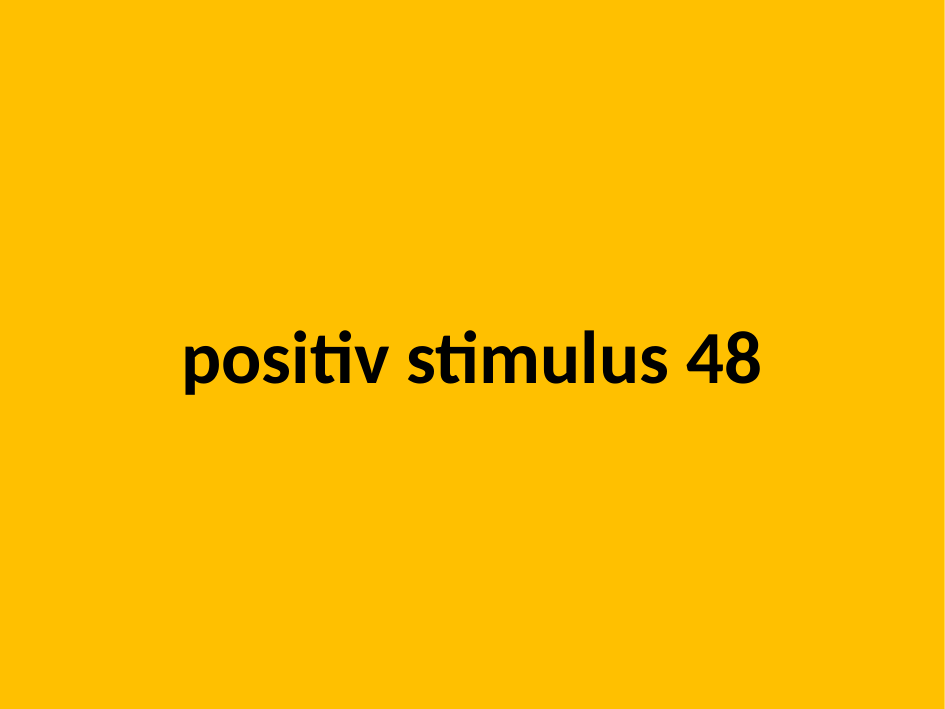

positiv stimulus 48

## Slide 49
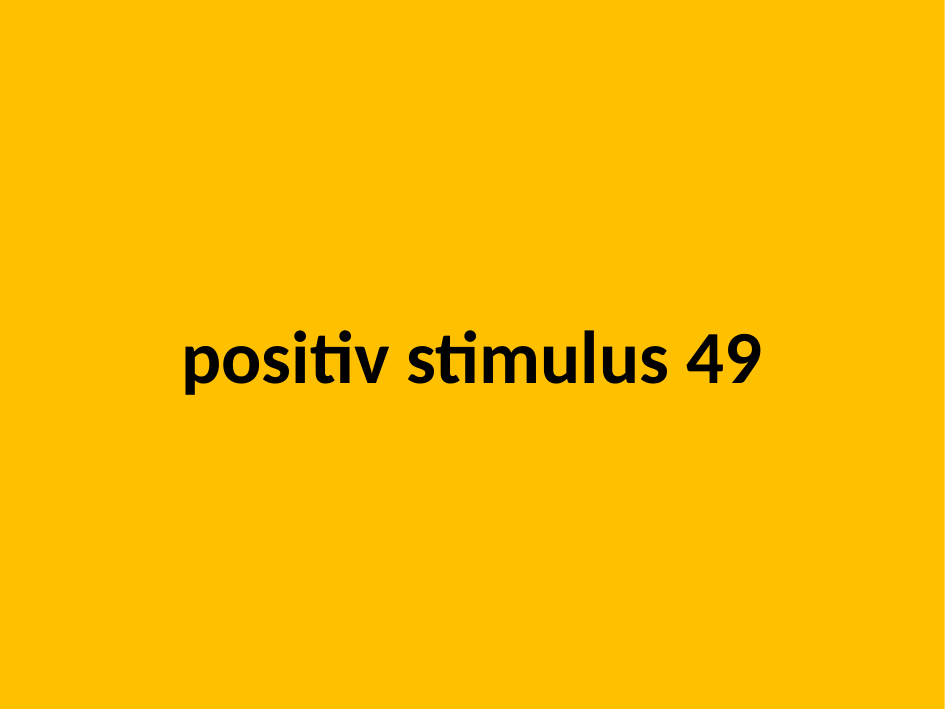

positiv stimulus 49

## Slide 50
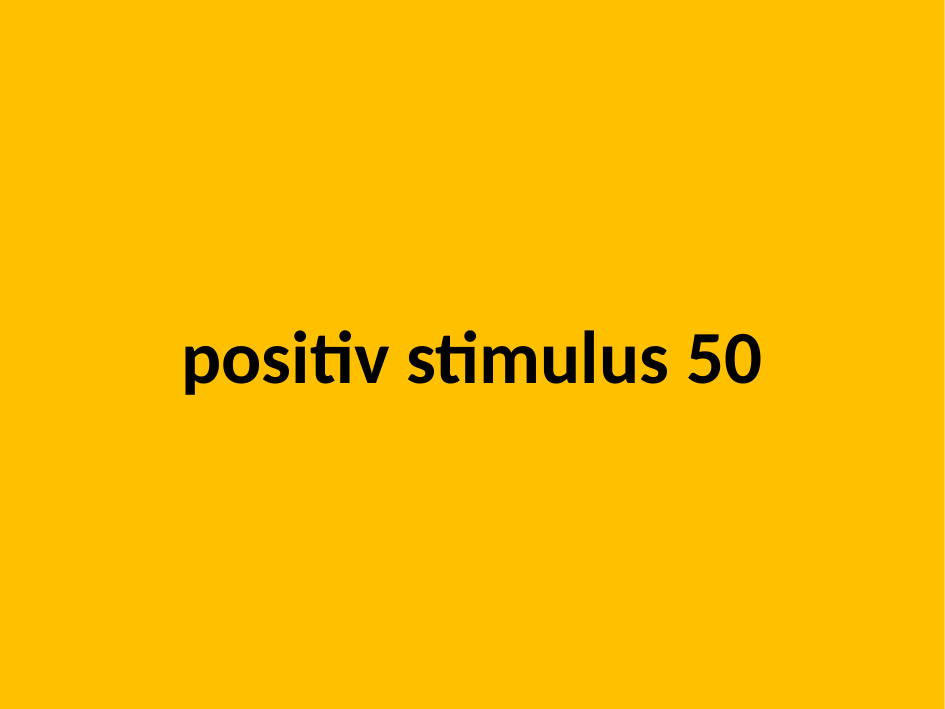

positiv stimulus 50

## Slide 51
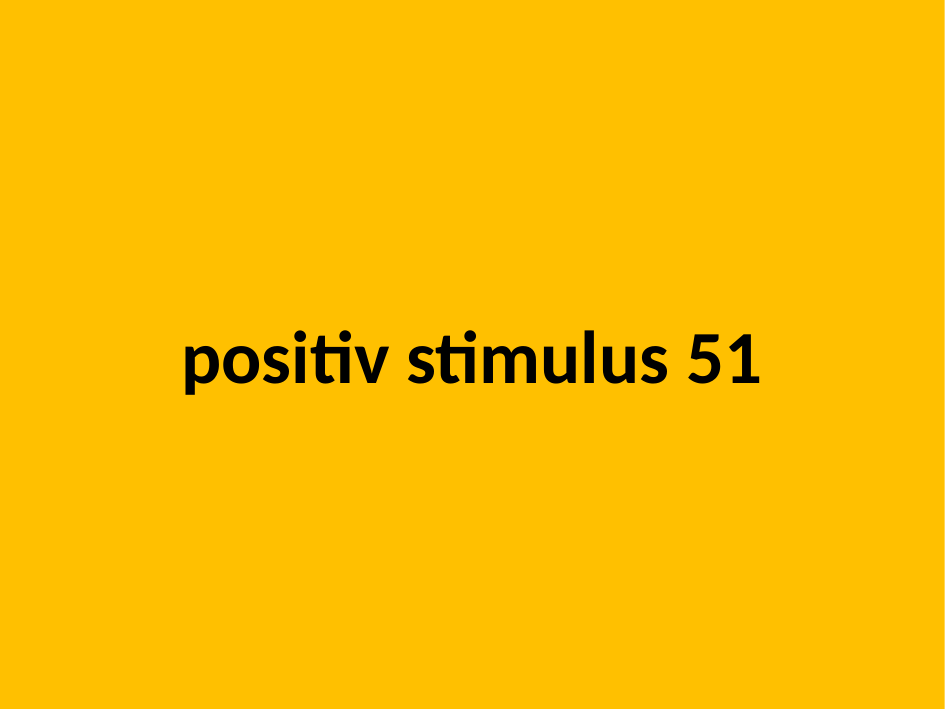

positiv stimulus 51

## Slide 52
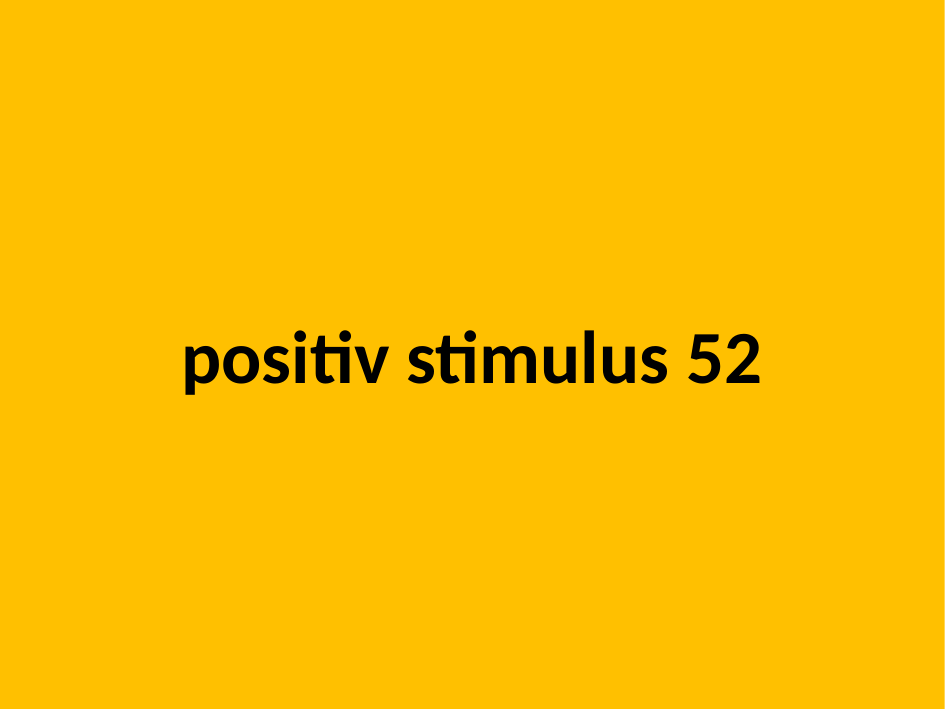

positiv stimulus 52

## Slide 53
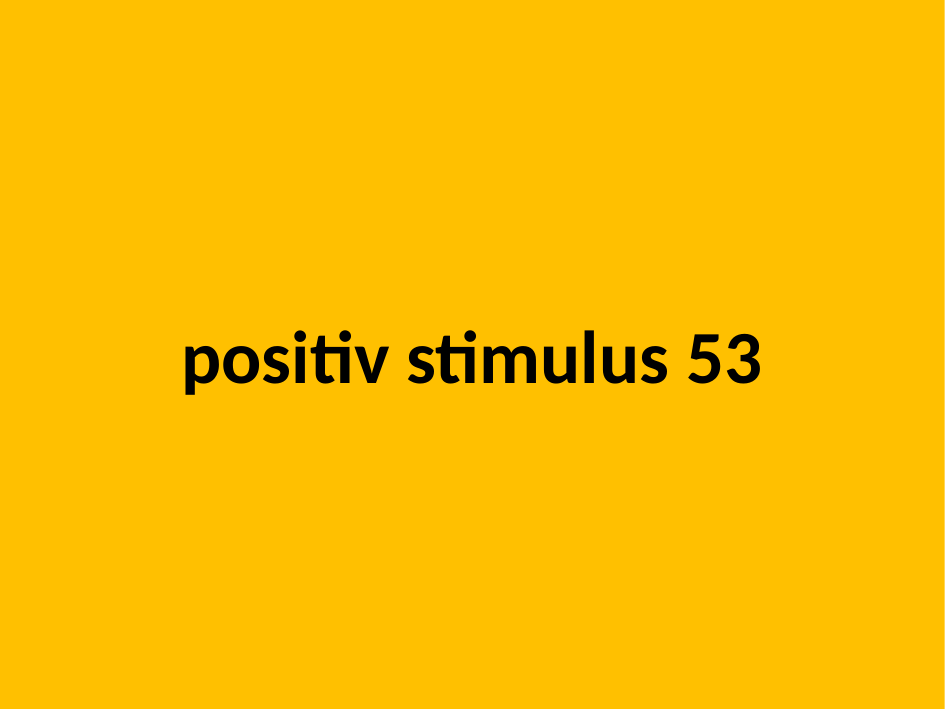

positiv stimulus 53

## Slide 54
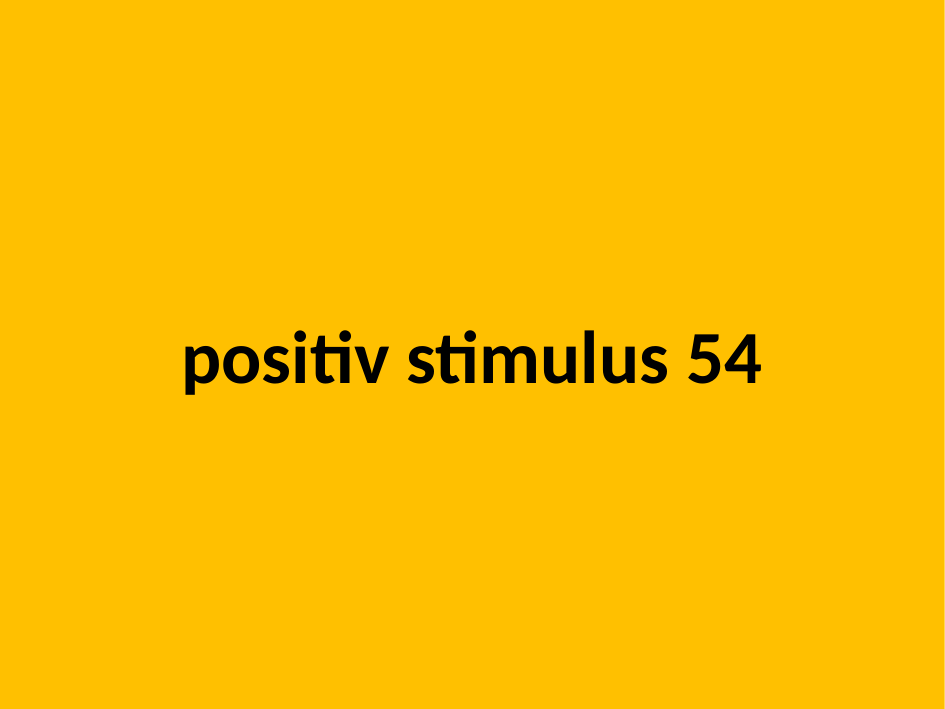

positiv stimulus 54

## Slide 55
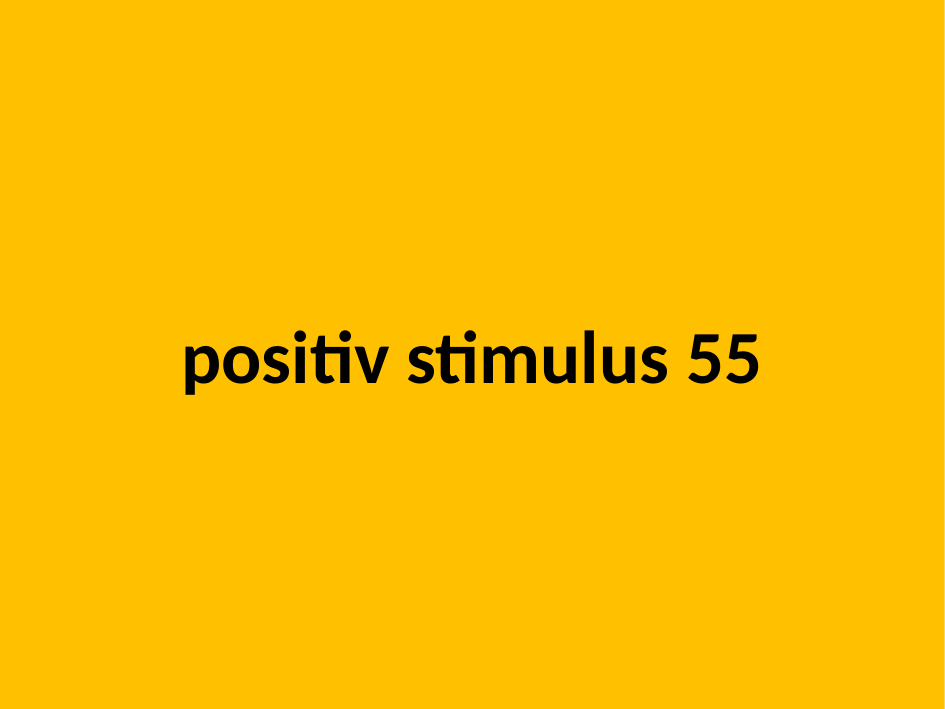

positiv stimulus 55

## Slide 56
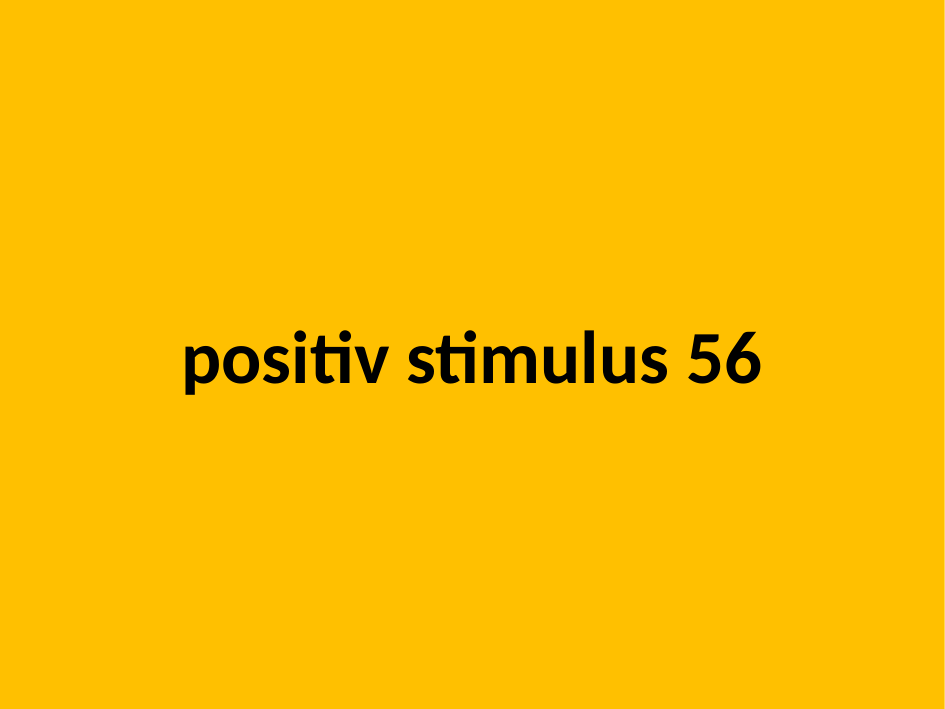

positiv stimulus 56

## Slide 57
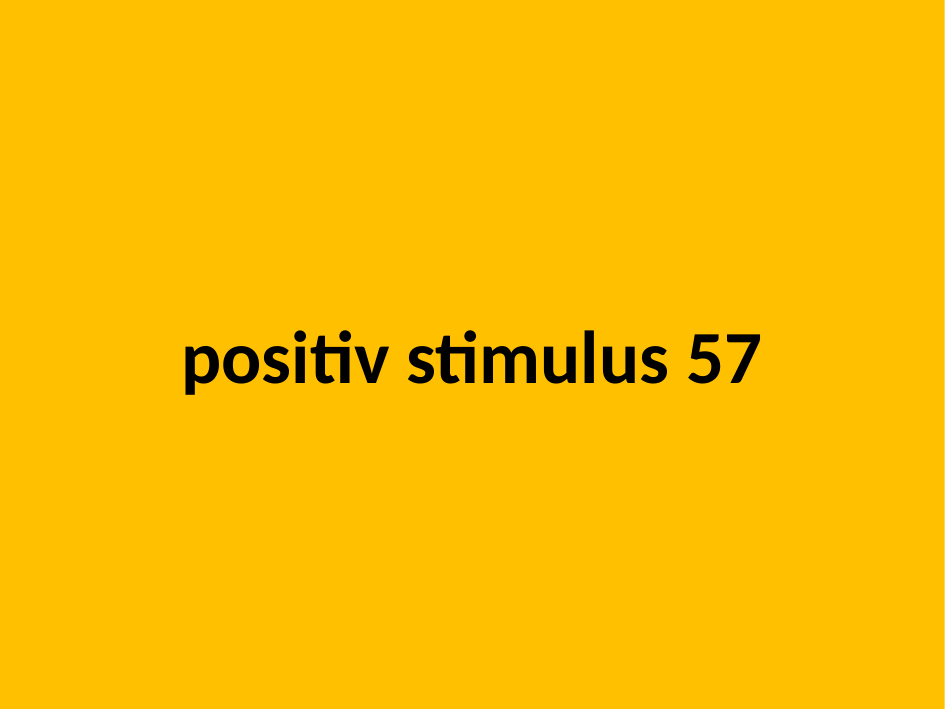

positiv stimulus 57

## Slide 58
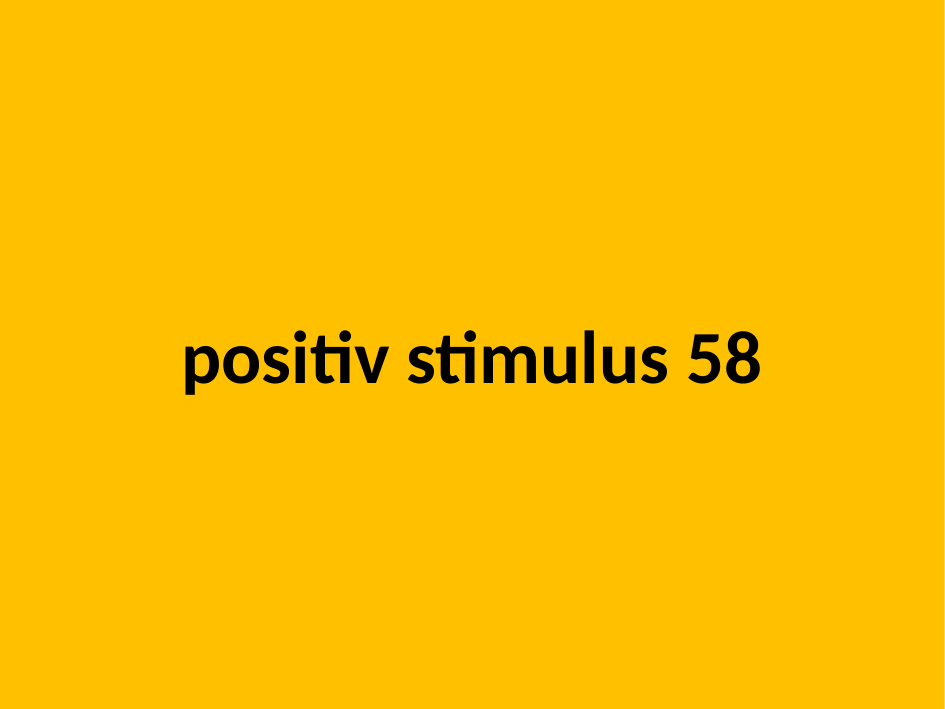

positiv stimulus 58

## Slide 59
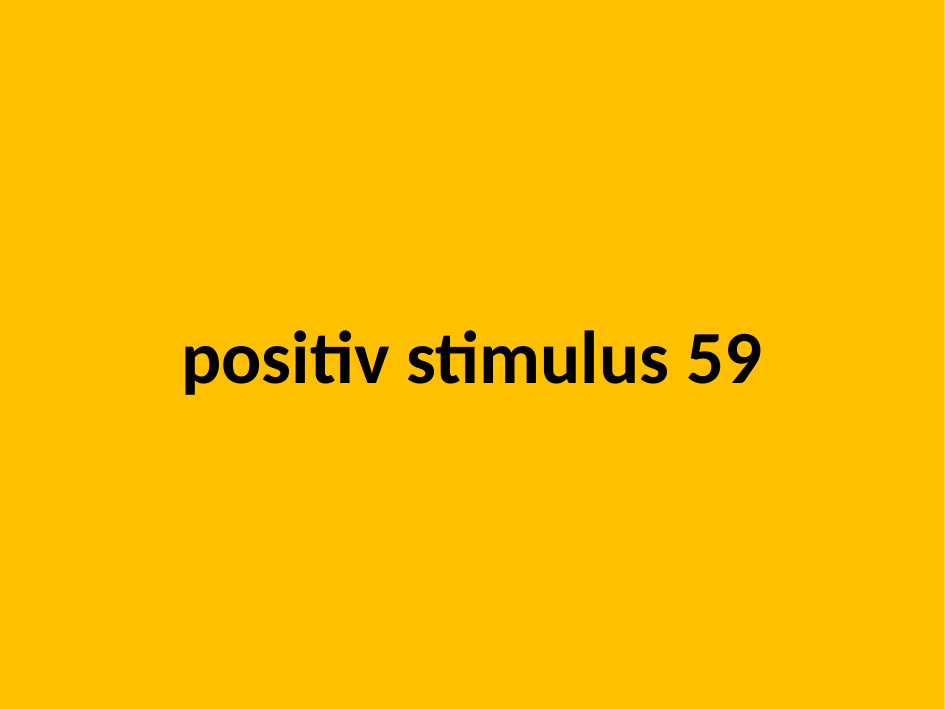

positiv stimulus 59

## Slide 60
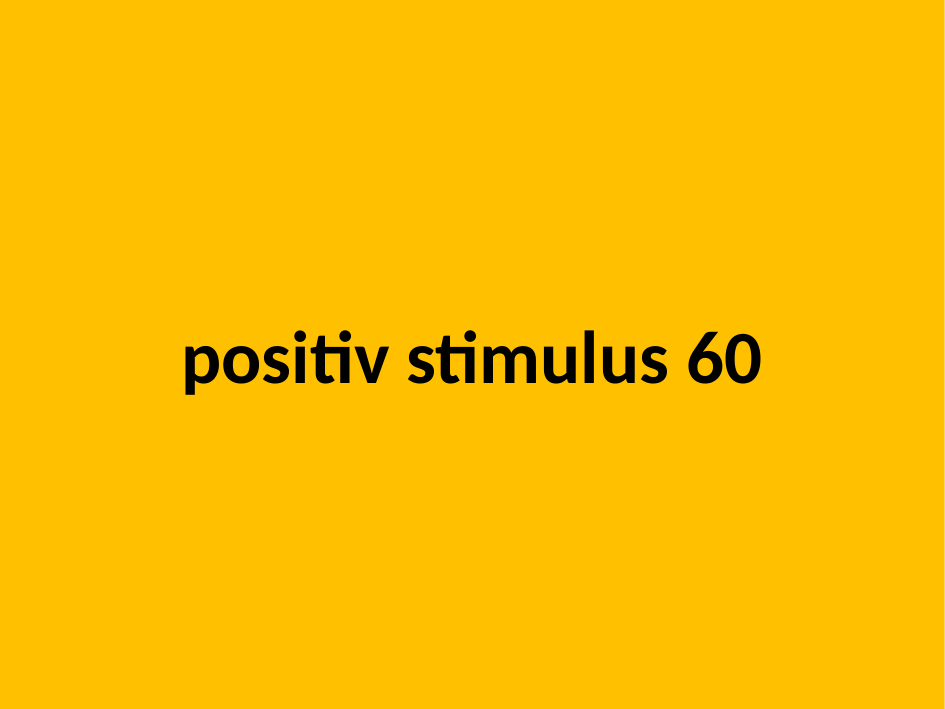

positiv stimulus 60

## Slide 61
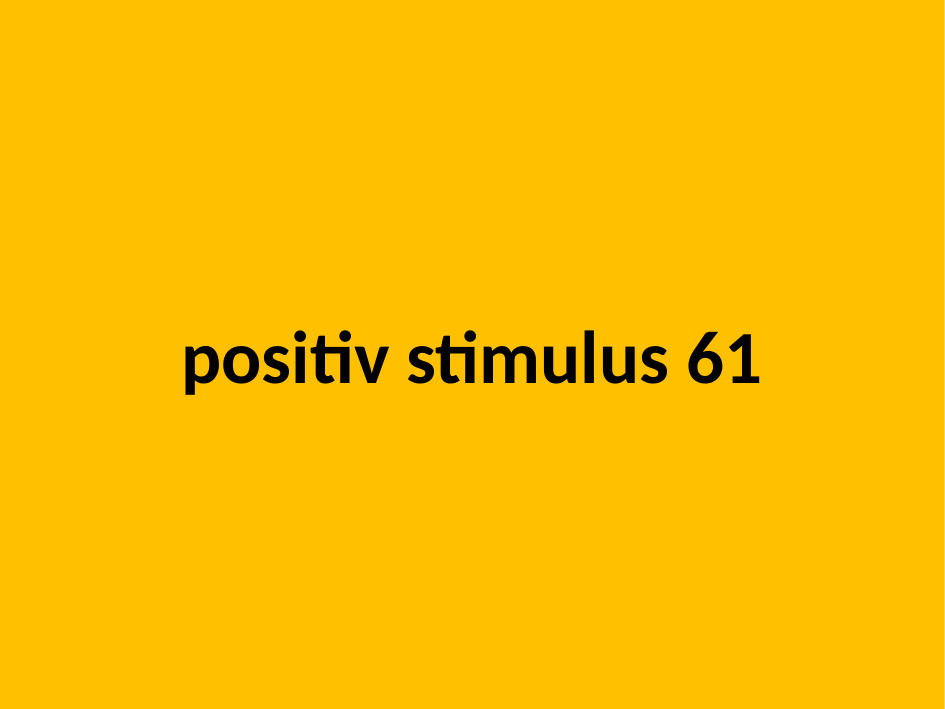

positiv stimulus 61

## Slide 62
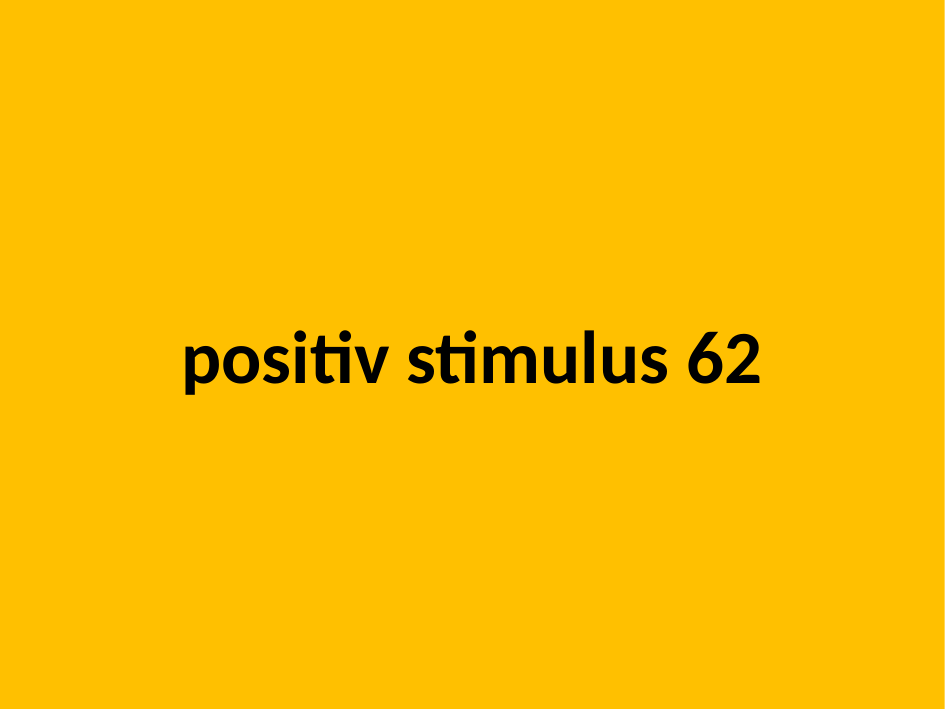

positiv stimulus 62

## Slide 63
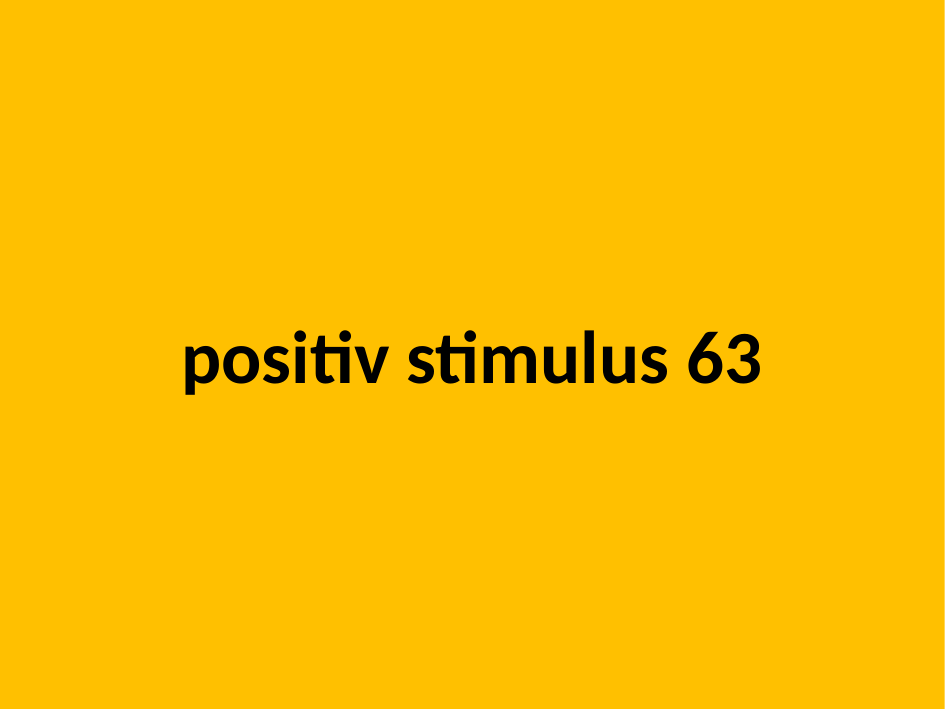

positiv stimulus 63

## Slide 64
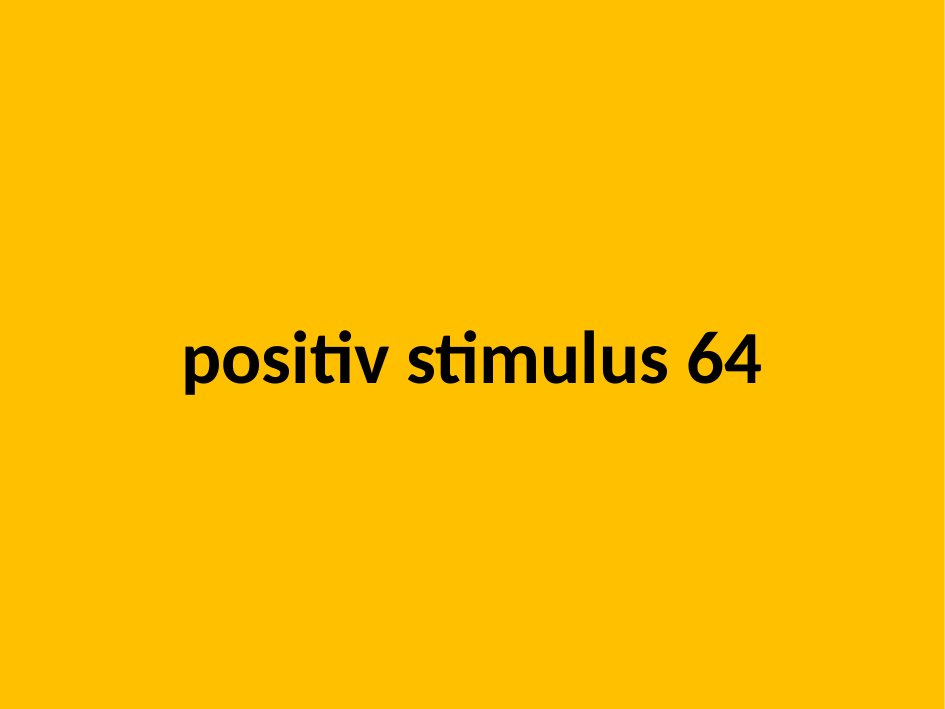

positiv stimulus 64

## Slide 65
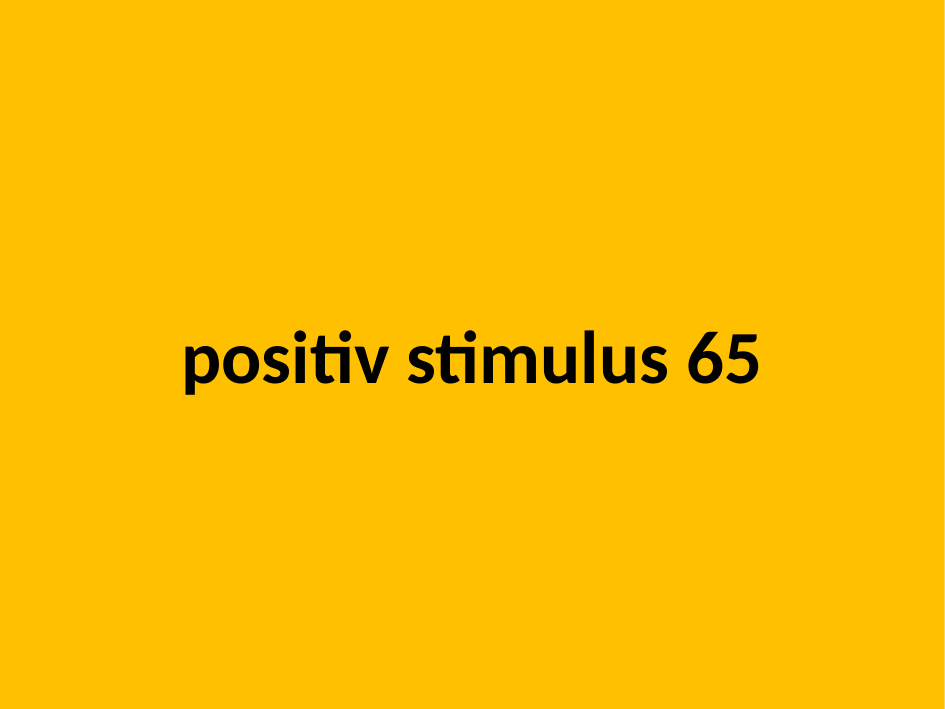

positiv stimulus 65

## Slide 66
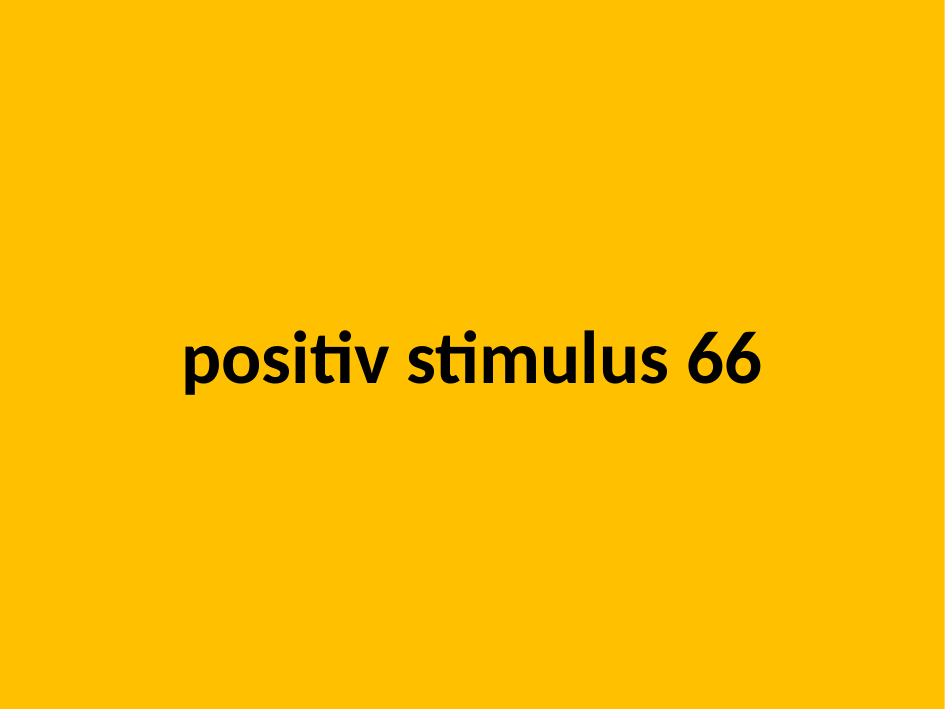

positiv stimulus 66

## Slide 67
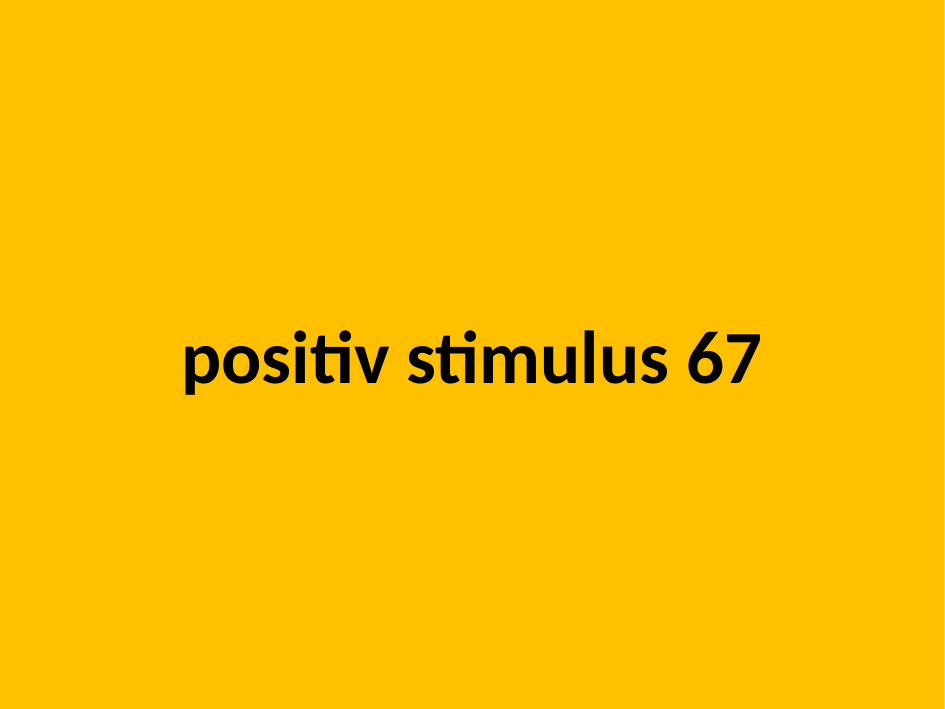

positiv stimulus 67

## Slide 68
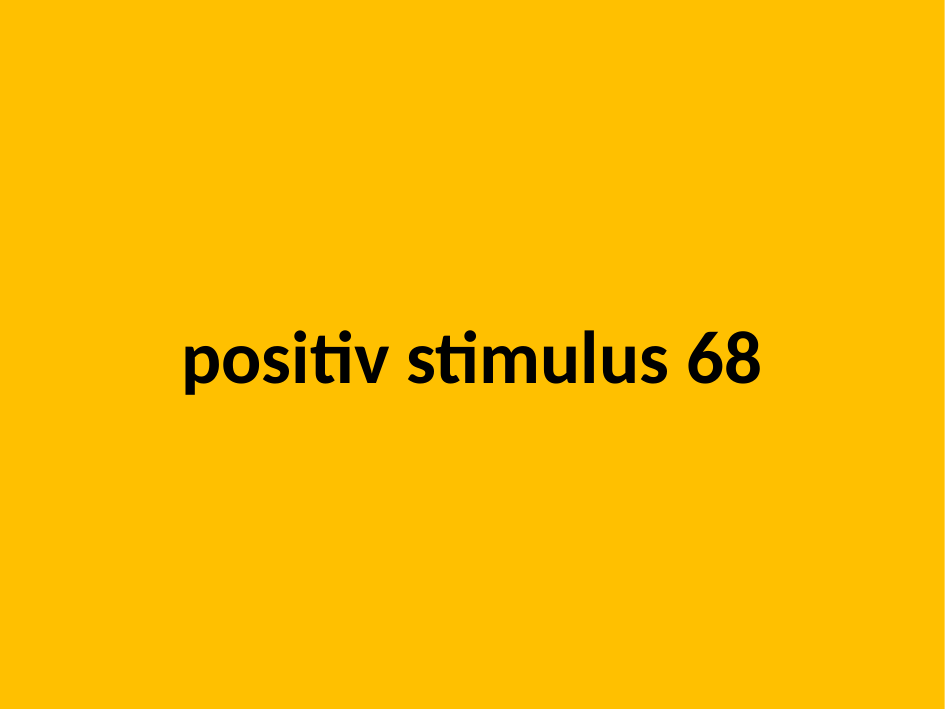

positiv stimulus 68

## Slide 69
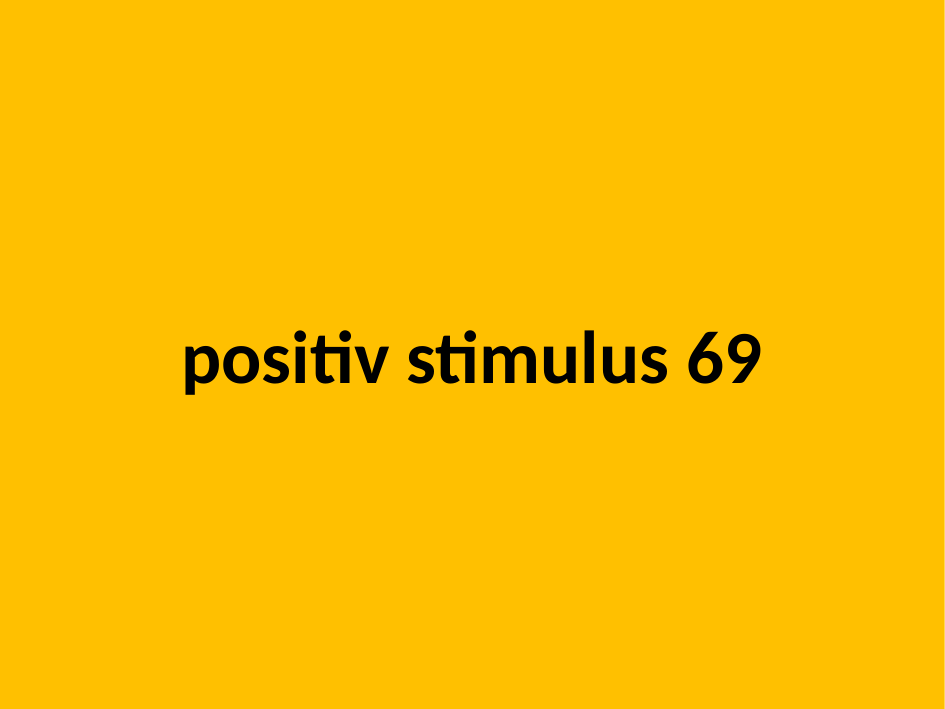

positiv stimulus 69

## Slide 70
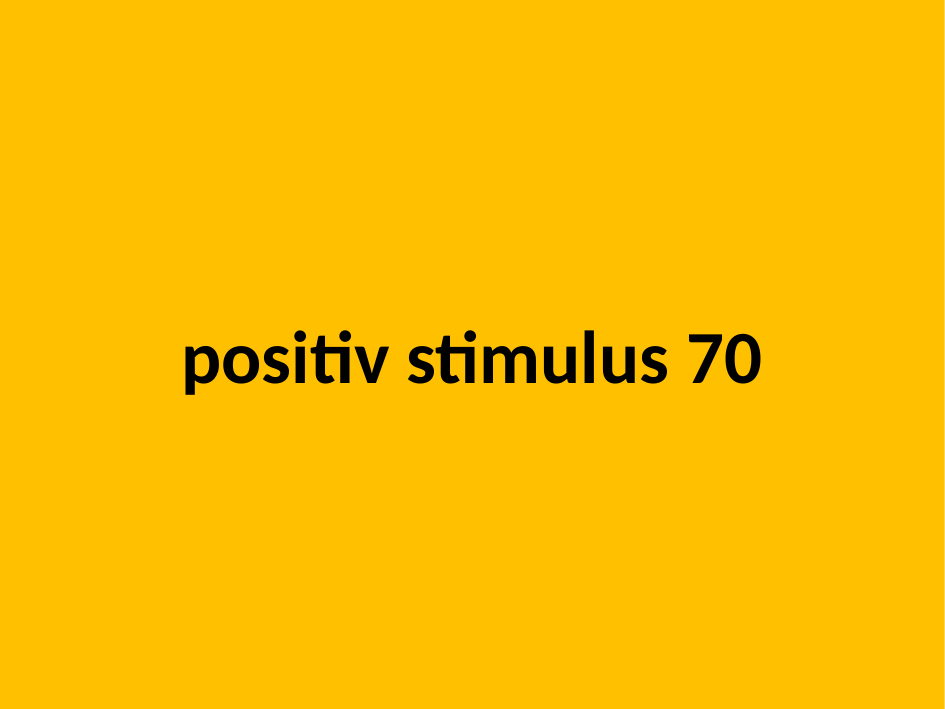

positiv stimulus 70
