# Supplementary figures and images for: Detours increase local knowledge—Exploring the hidden benefits of self-control failure (part 2 of 2)
Source: PLoS One. 2021 Oct 1;16(10):e0257717. doi: 10.1371/journal.pone.0257717 (PMC8486128; doi:10.1371/journal.pone.0257717)

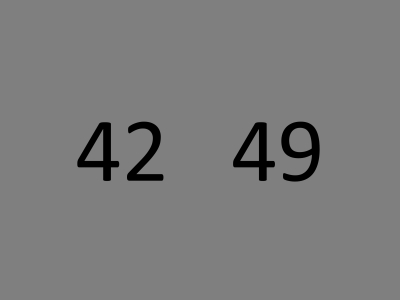

Supplement: S2 File — (ZIP) [file pone.0257717.s002.zip › software/stimuli/HaveToTask86.jpg]

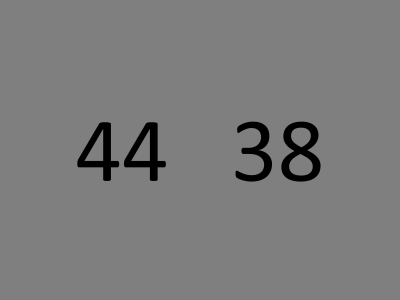

Supplement: S2 File — (ZIP) [file pone.0257717.s002.zip › software/stimuli/HaveToTask87.jpg]

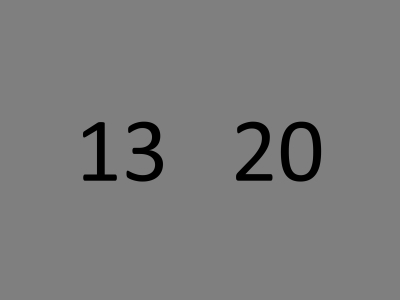

Supplement: S2 File — (ZIP) [file pone.0257717.s002.zip › software/stimuli/HaveToTask88.jpg]

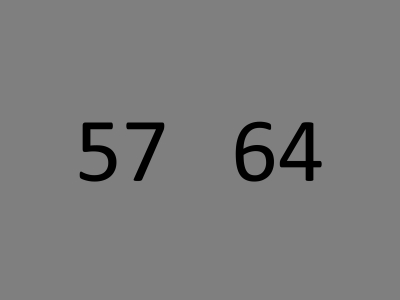

Supplement: S2 File — (ZIP) [file pone.0257717.s002.zip › software/stimuli/HaveToTask89.jpg]

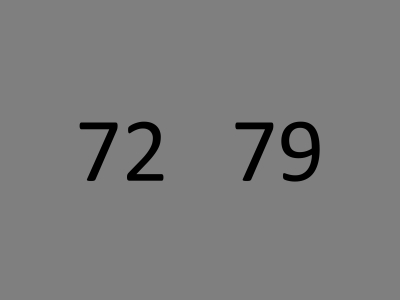

Supplement: S2 File — (ZIP) [file pone.0257717.s002.zip › software/stimuli/HaveToTask9.jpg]

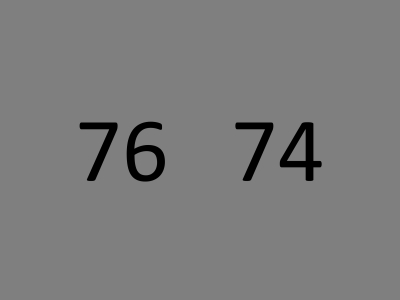

Supplement: S2 File — (ZIP) [file pone.0257717.s002.zip › software/stimuli/HaveToTask90.jpg]

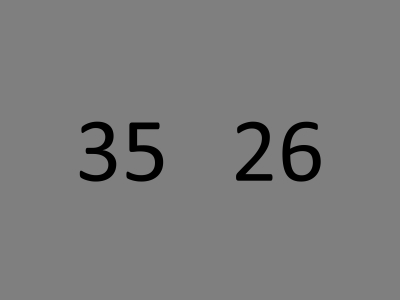

Supplement: S2 File — (ZIP) [file pone.0257717.s002.zip › software/stimuli/HaveToTask91.jpg]

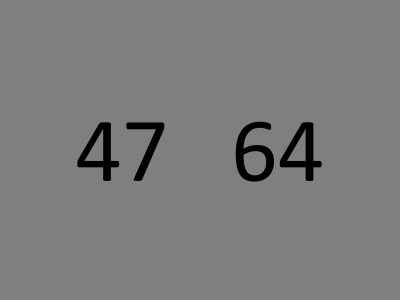

Supplement: S2 File — (ZIP) [file pone.0257717.s002.zip › software/stimuli/HaveToTask92.jpg]

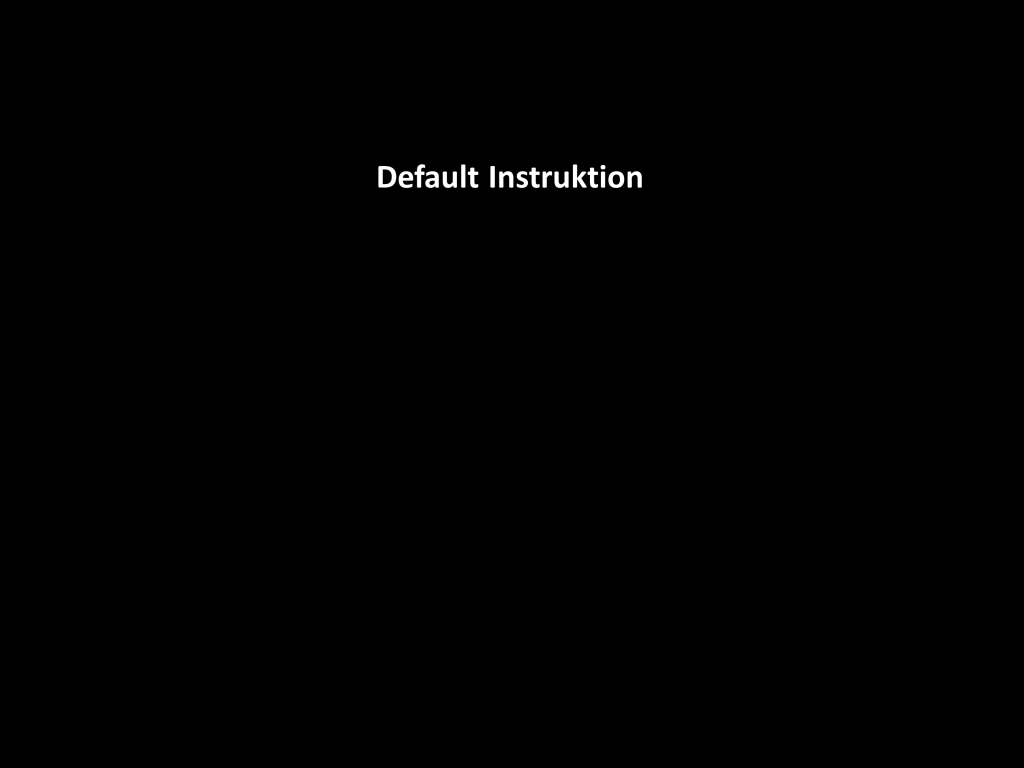

Supplement: S2 File — (ZIP) [file pone.0257717.s002.zip › software/stimuli/InstructionDefault.tif]

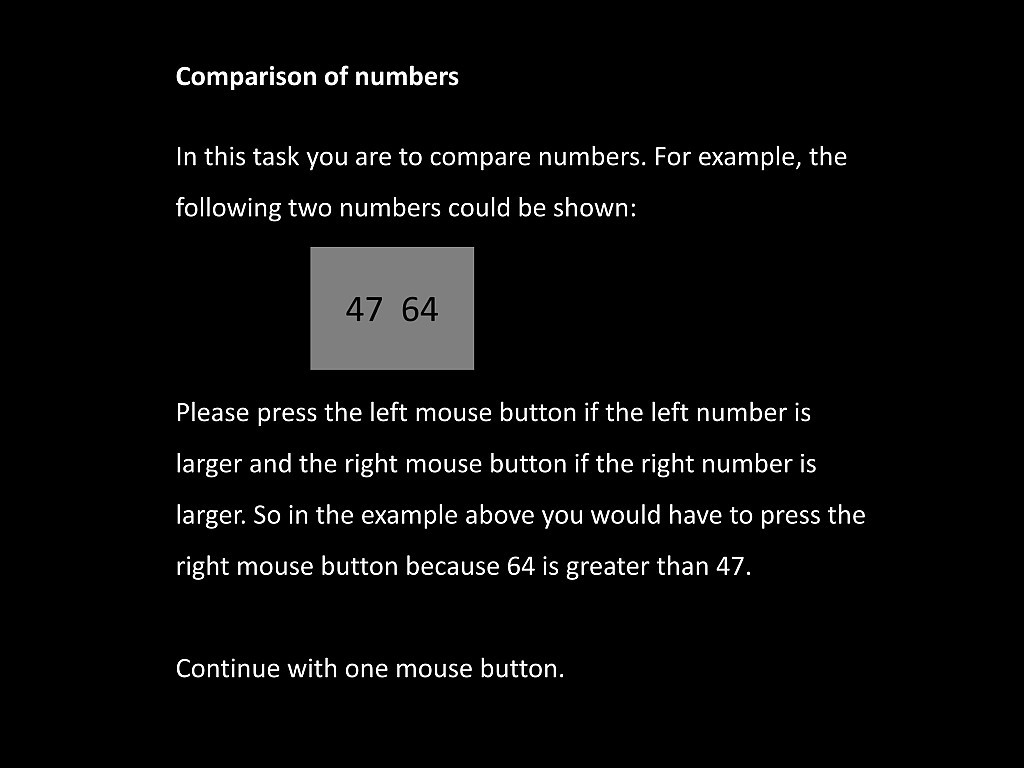

Supplement: S2 File — (ZIP) [file pone.0257717.s002.zip › software/stimuli/InstructionHaveTo1.jpg]

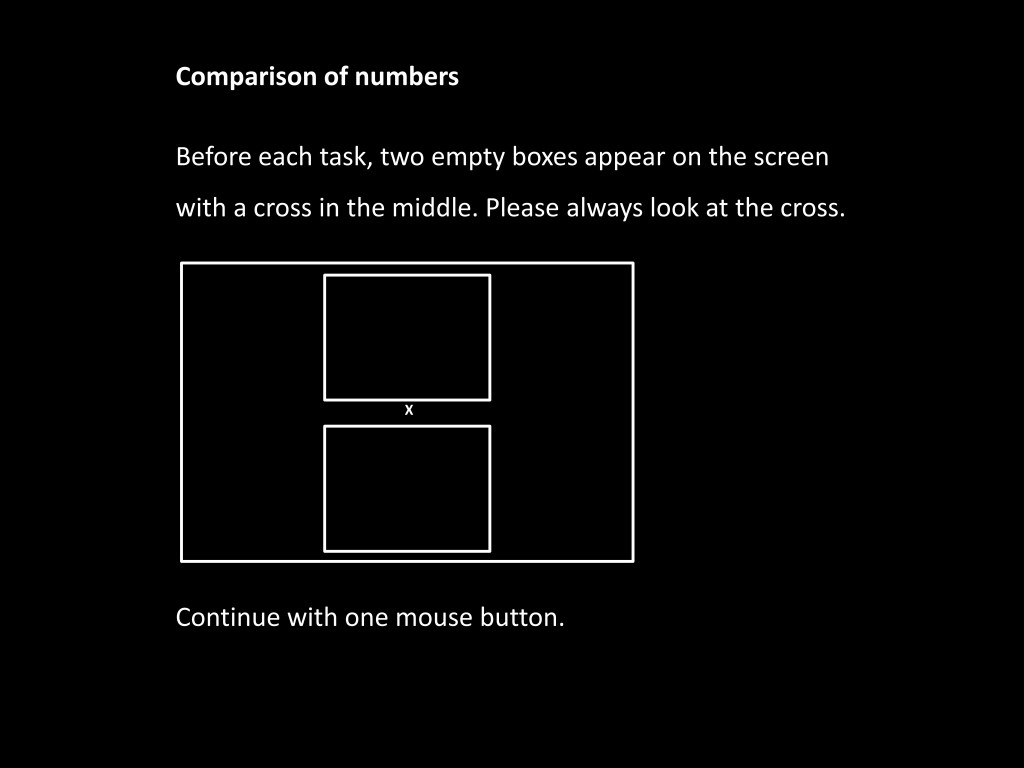

Supplement: S2 File — (ZIP) [file pone.0257717.s002.zip › software/stimuli/InstructionHaveTo2.jpg]

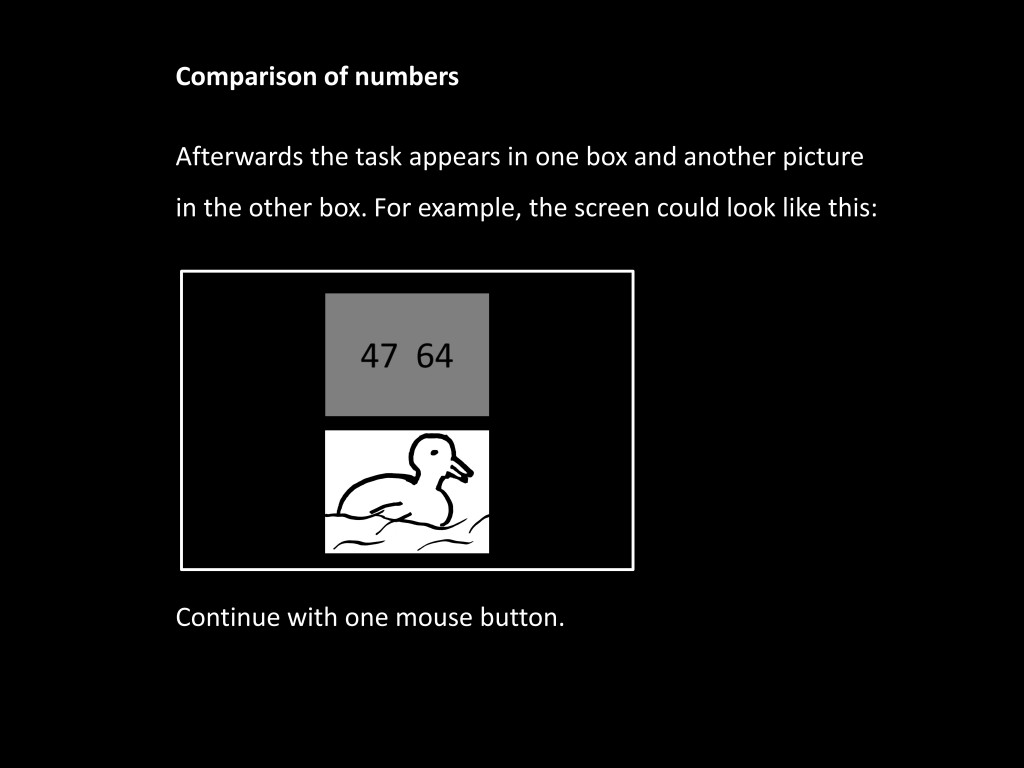

Supplement: S2 File — (ZIP) [file pone.0257717.s002.zip › software/stimuli/InstructionHaveTo3.jpg]

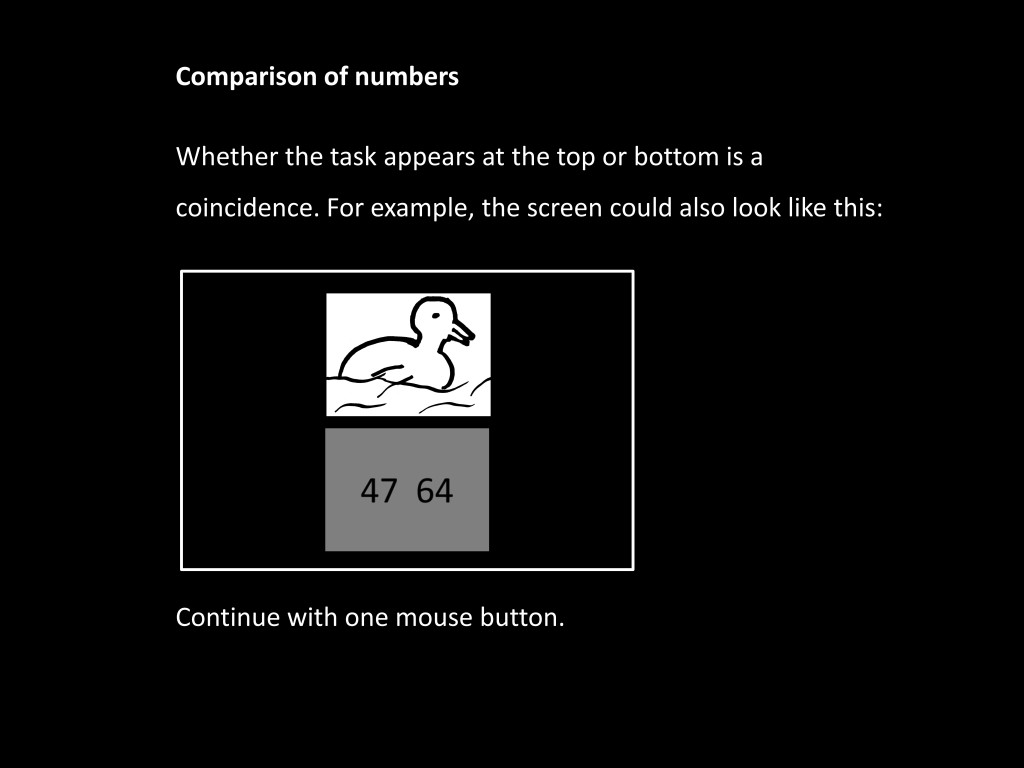

Supplement: S2 File — (ZIP) [file pone.0257717.s002.zip › software/stimuli/InstructionHaveTo4.jpg]

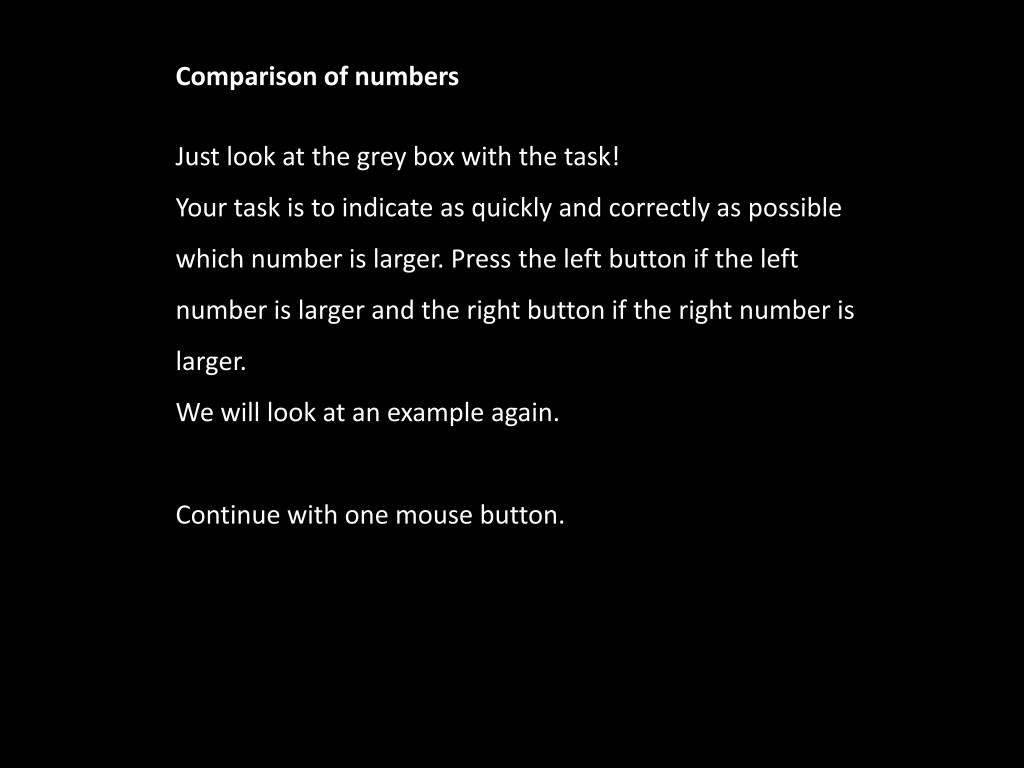

Supplement: S2 File — (ZIP) [file pone.0257717.s002.zip › software/stimuli/InstructionHaveTo5.jpg]

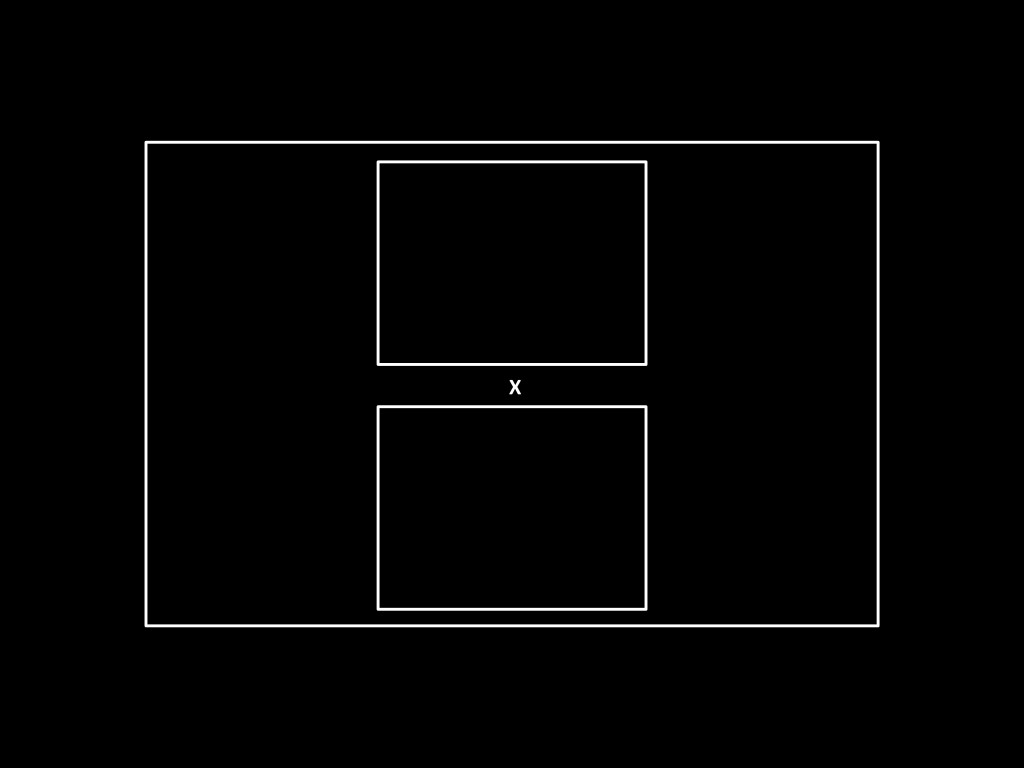

Supplement: S2 File — (ZIP) [file pone.0257717.s002.zip › software/stimuli/InstructionHaveTo6.jpg]

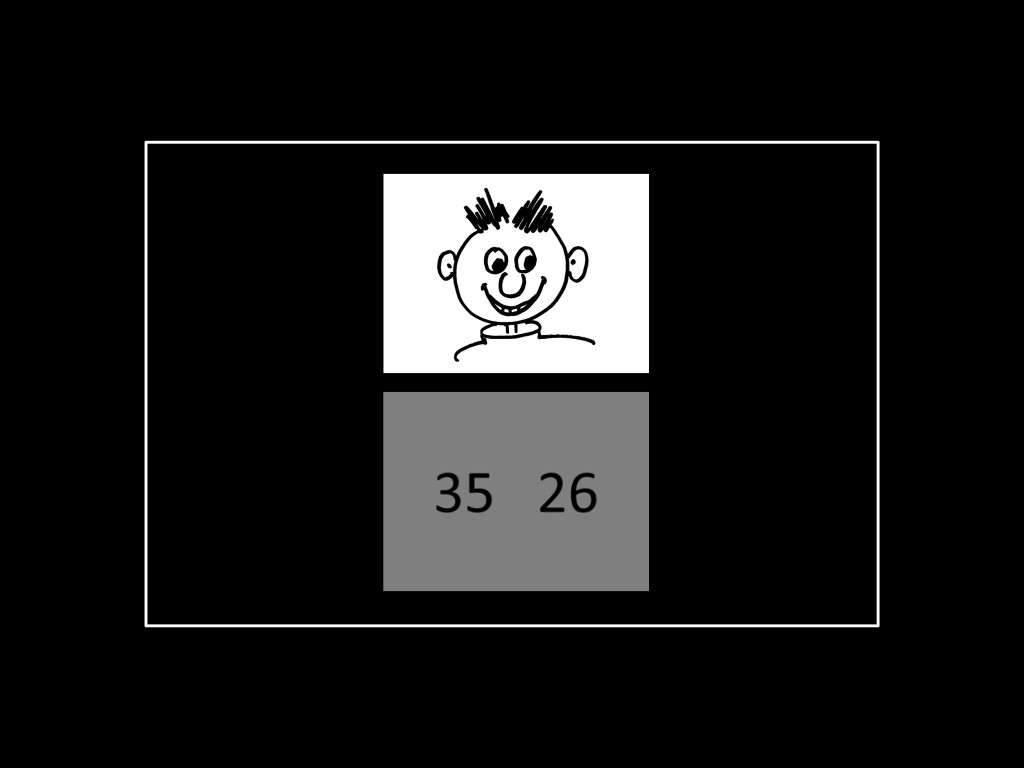

Supplement: S2 File — (ZIP) [file pone.0257717.s002.zip › software/stimuli/InstructionHaveTo7.jpg]

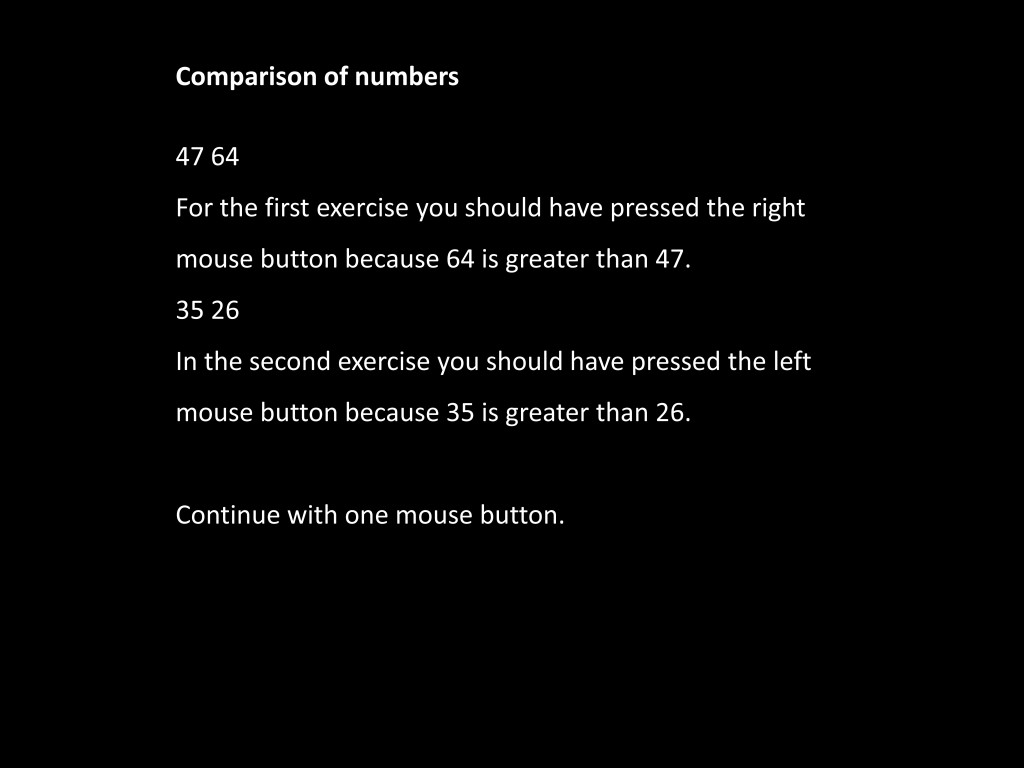

Supplement: S2 File — (ZIP) [file pone.0257717.s002.zip › software/stimuli/InstructionHaveTo8.jpg]

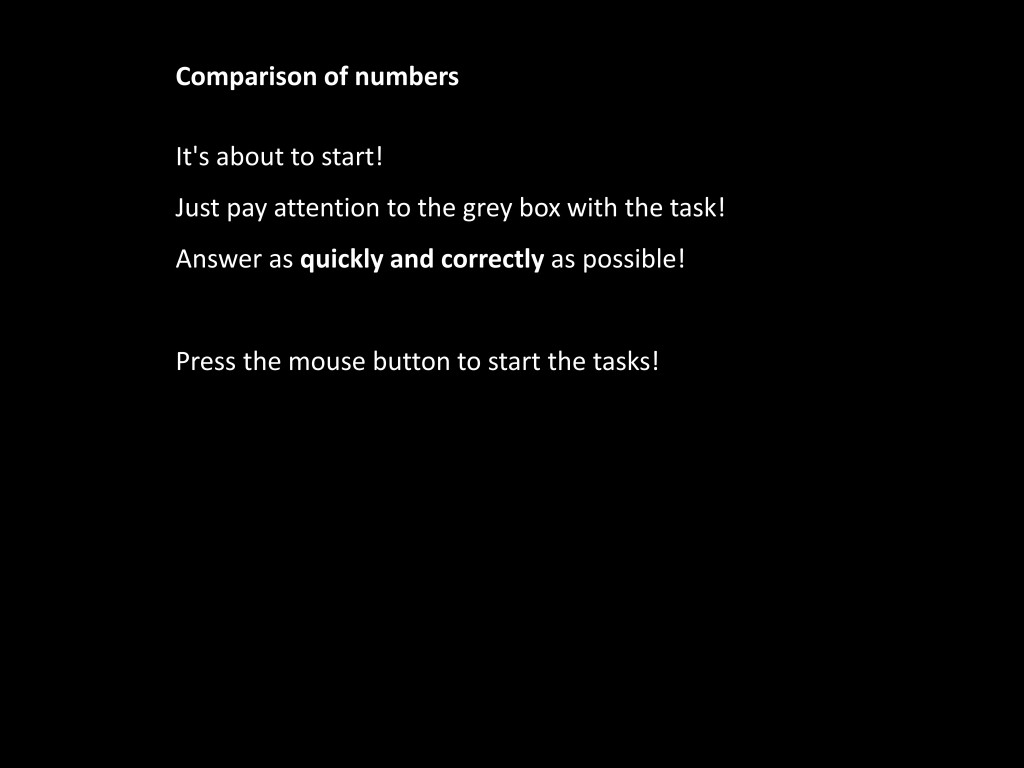

Supplement: S2 File — (ZIP) [file pone.0257717.s002.zip › software/stimuli/InstructionHaveTo9.jpg]

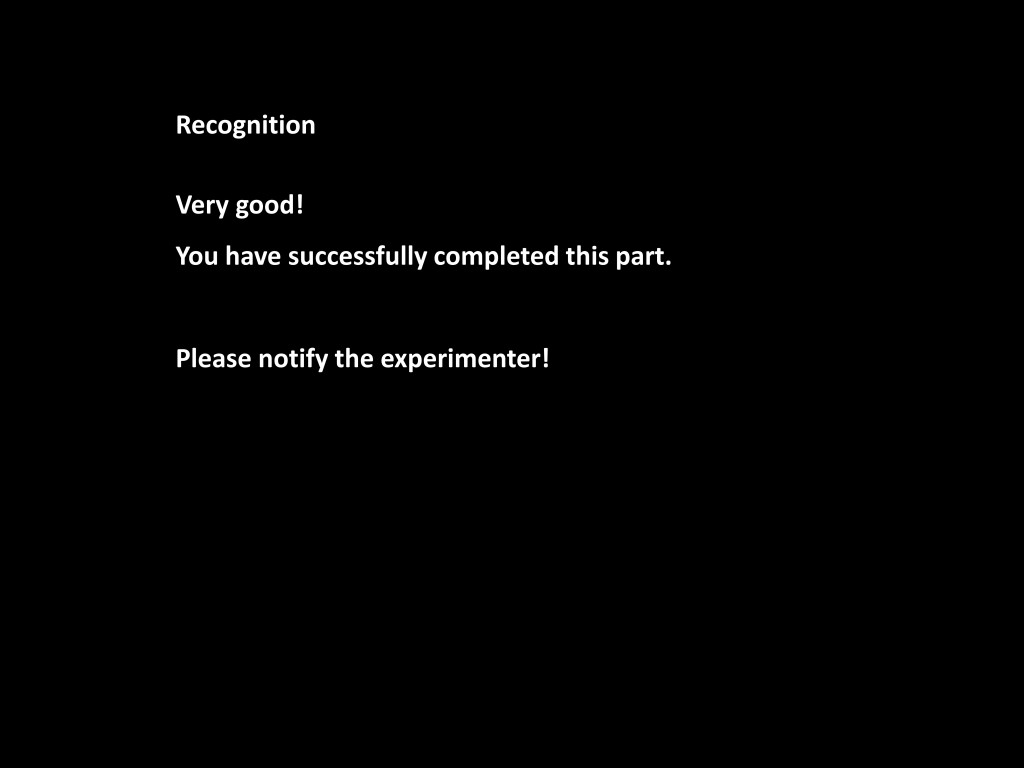

Supplement: S2 File — (ZIP) [file pone.0257717.s002.zip › software/stimuli/InstructionPause.jpg]

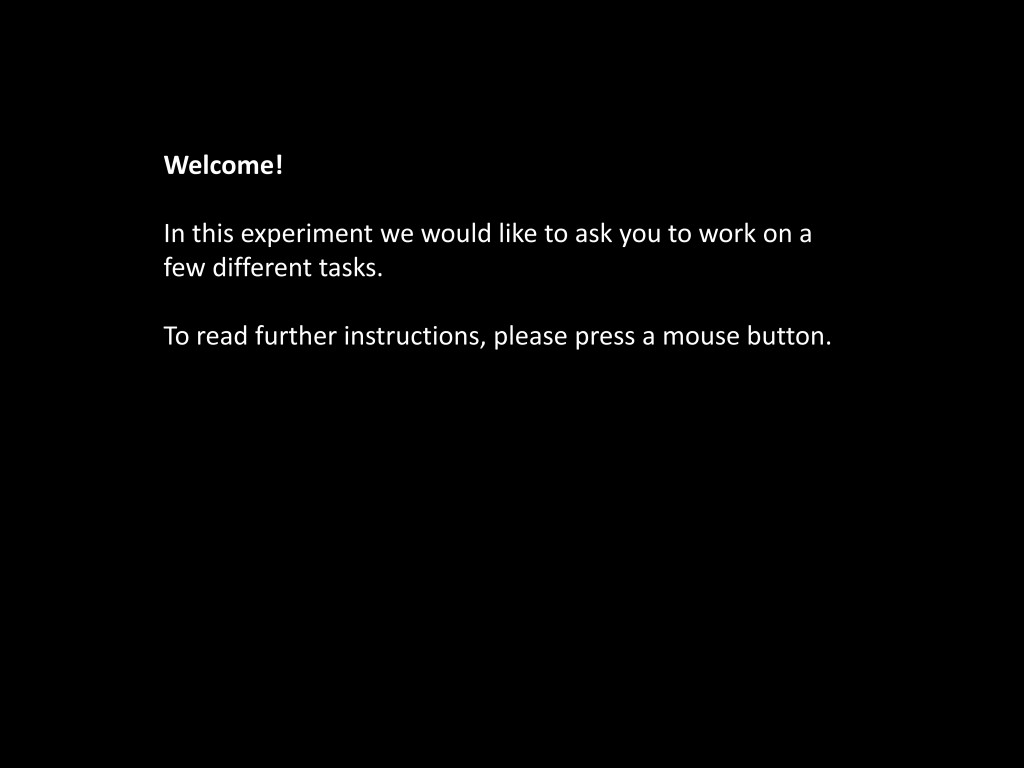

Supplement: S2 File — (ZIP) [file pone.0257717.s002.zip › software/stimuli/InstructionStart.jpg]

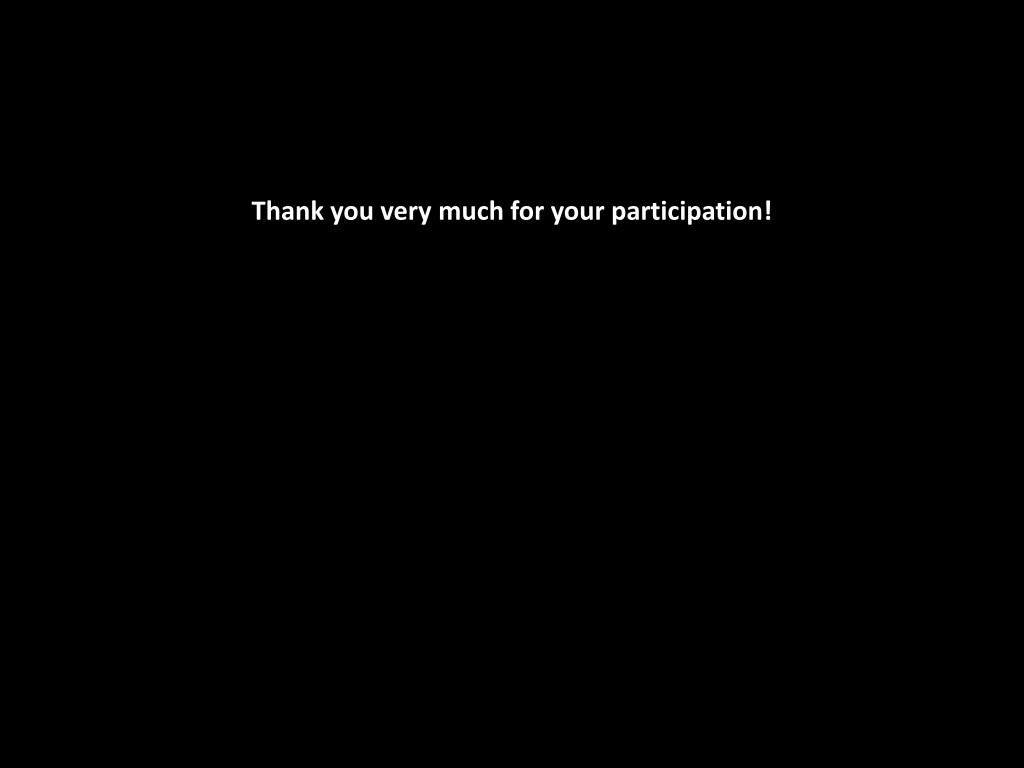

Supplement: S2 File — (ZIP) [file pone.0257717.s002.zip › software/stimuli/InstructionStop.jpg]

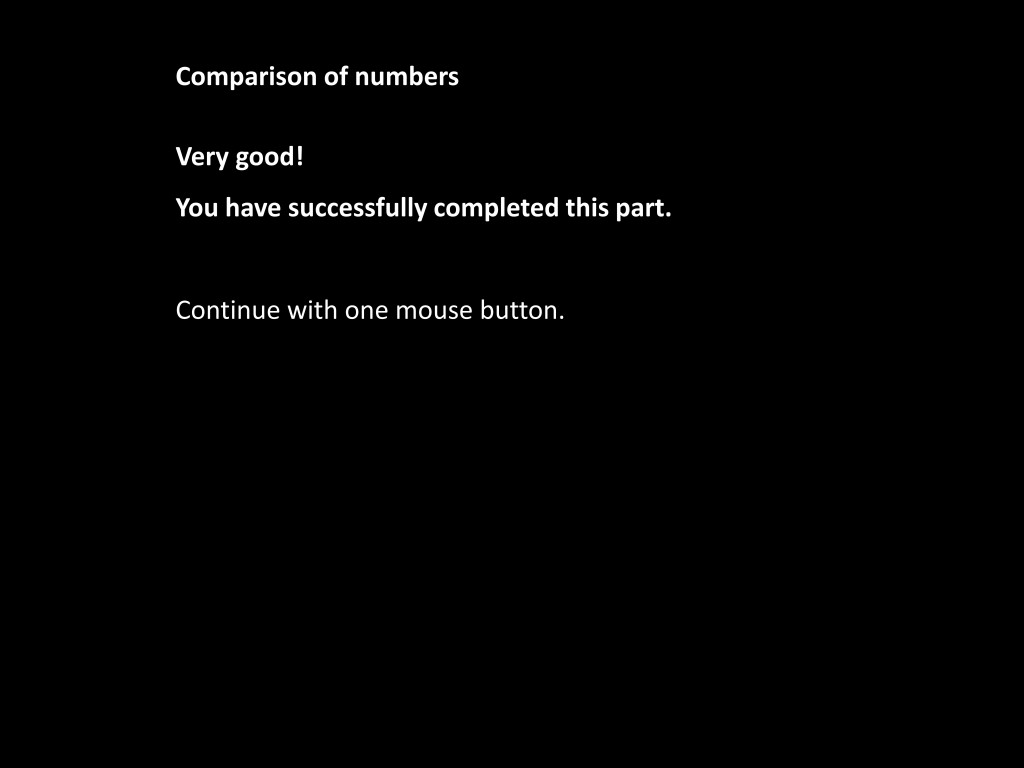

Supplement: S2 File — (ZIP) [file pone.0257717.s002.zip › software/stimuli/InstructionVideo1.jpg]

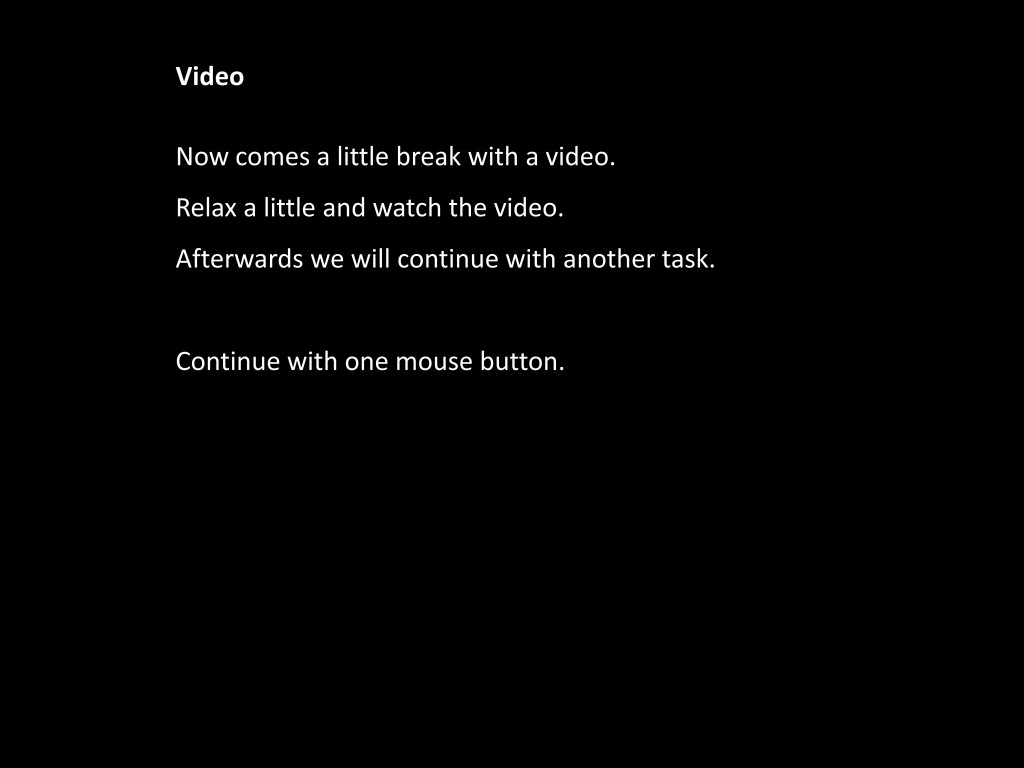

Supplement: S2 File — (ZIP) [file pone.0257717.s002.zip › software/stimuli/InstructionVideo2.jpg]

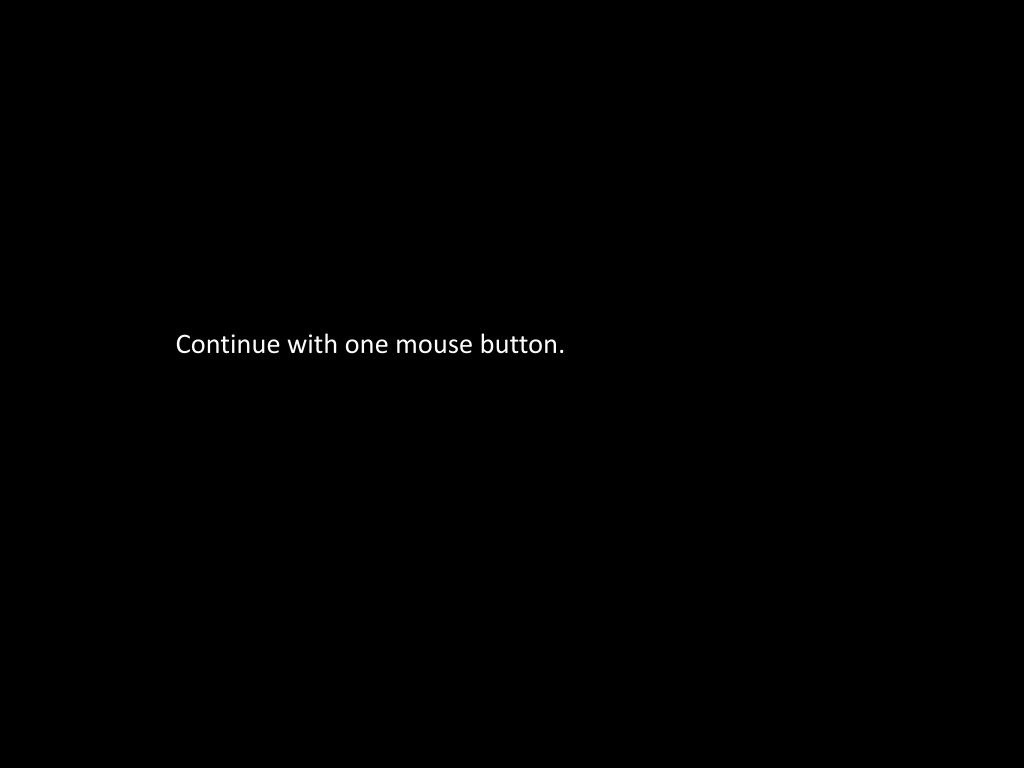

Supplement: S2 File — (ZIP) [file pone.0257717.s002.zip › software/stimuli/InstructionVideo3.jpg]

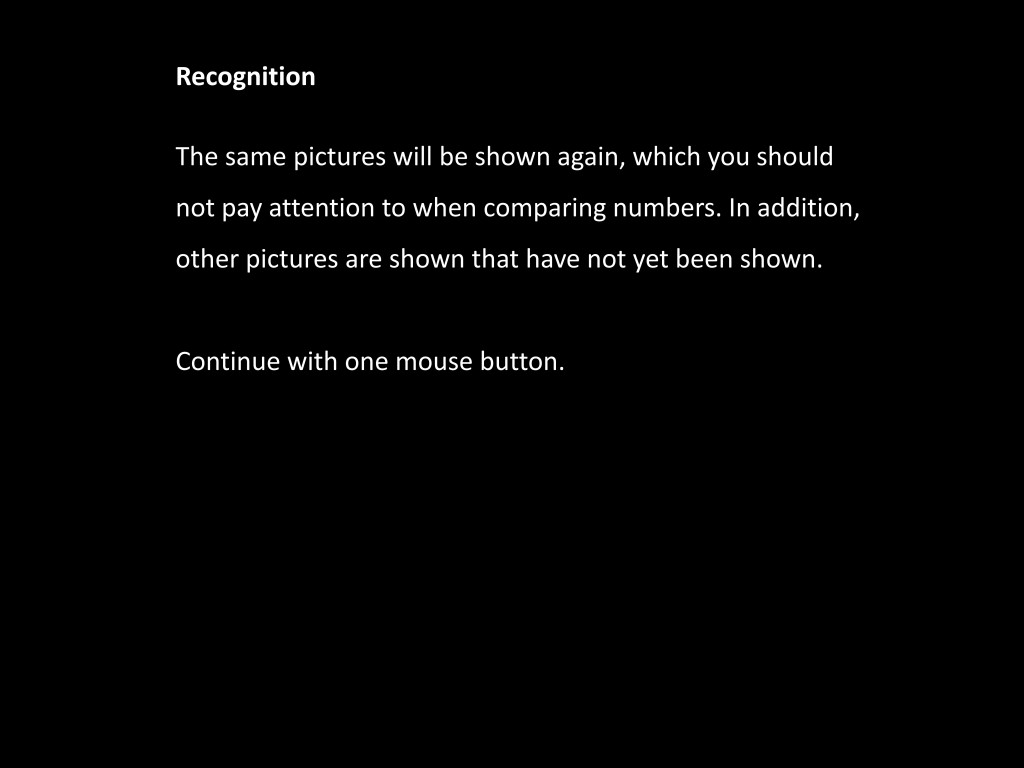

Supplement: S2 File — (ZIP) [file pone.0257717.s002.zip › software/stimuli/InstructionWantTo1.jpg]

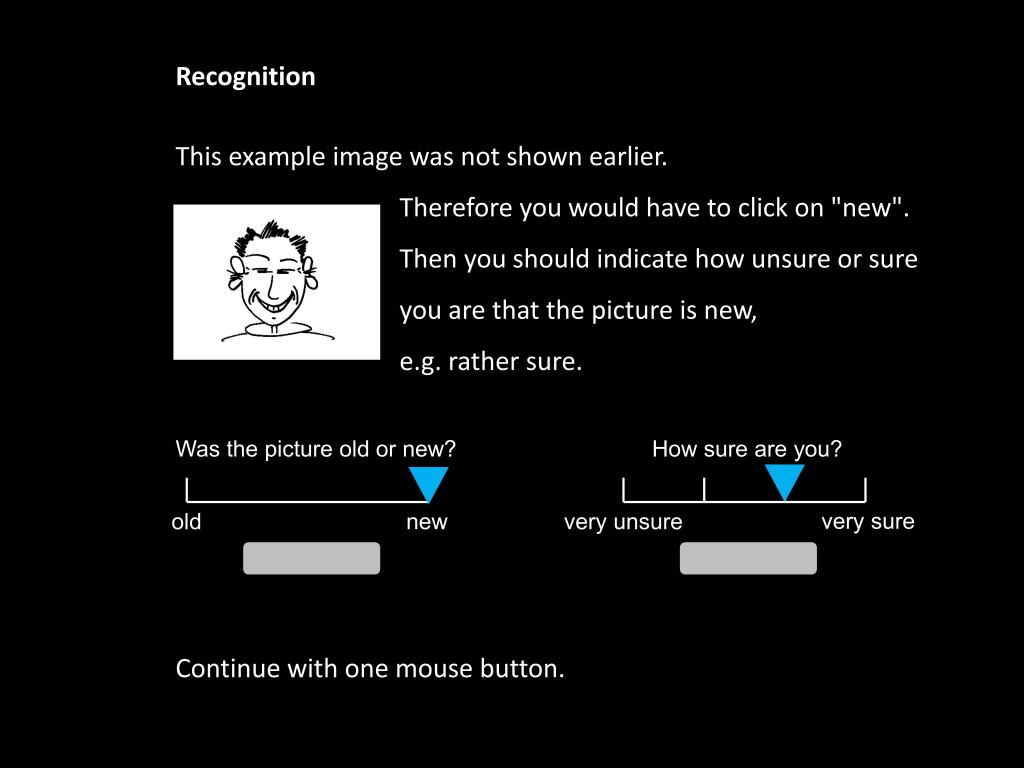

Supplement: S2 File — (ZIP) [file pone.0257717.s002.zip › software/stimuli/InstructionWantTo10.jpg]

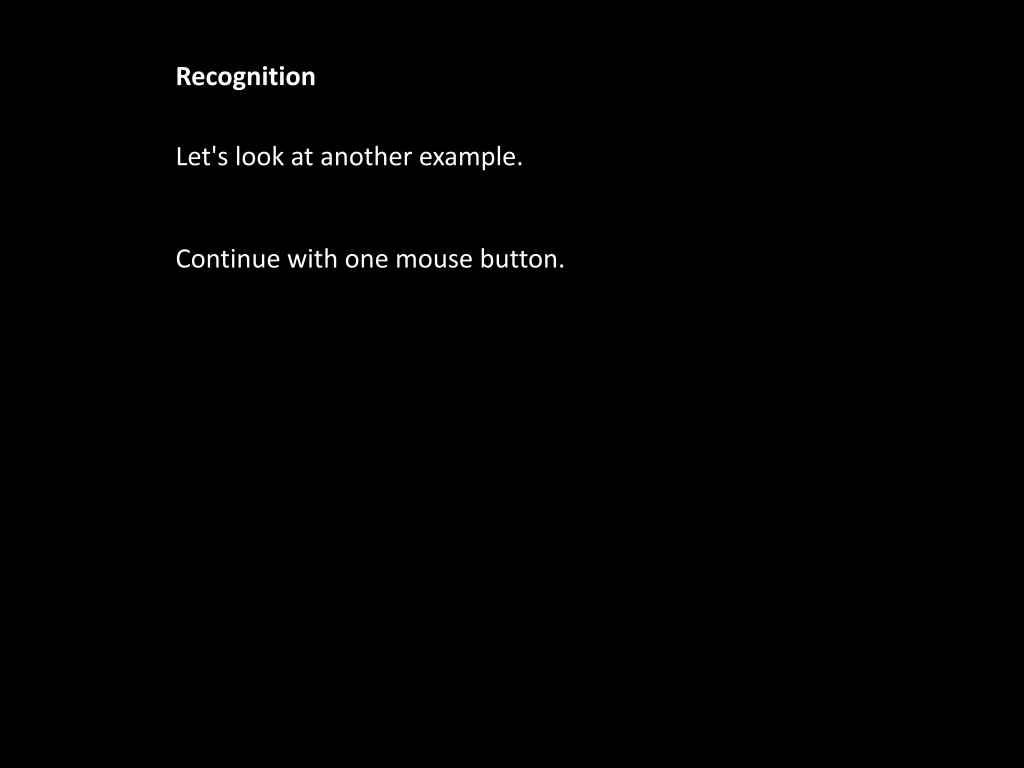

Supplement: S2 File — (ZIP) [file pone.0257717.s002.zip › software/stimuli/InstructionWantTo11.jpg]

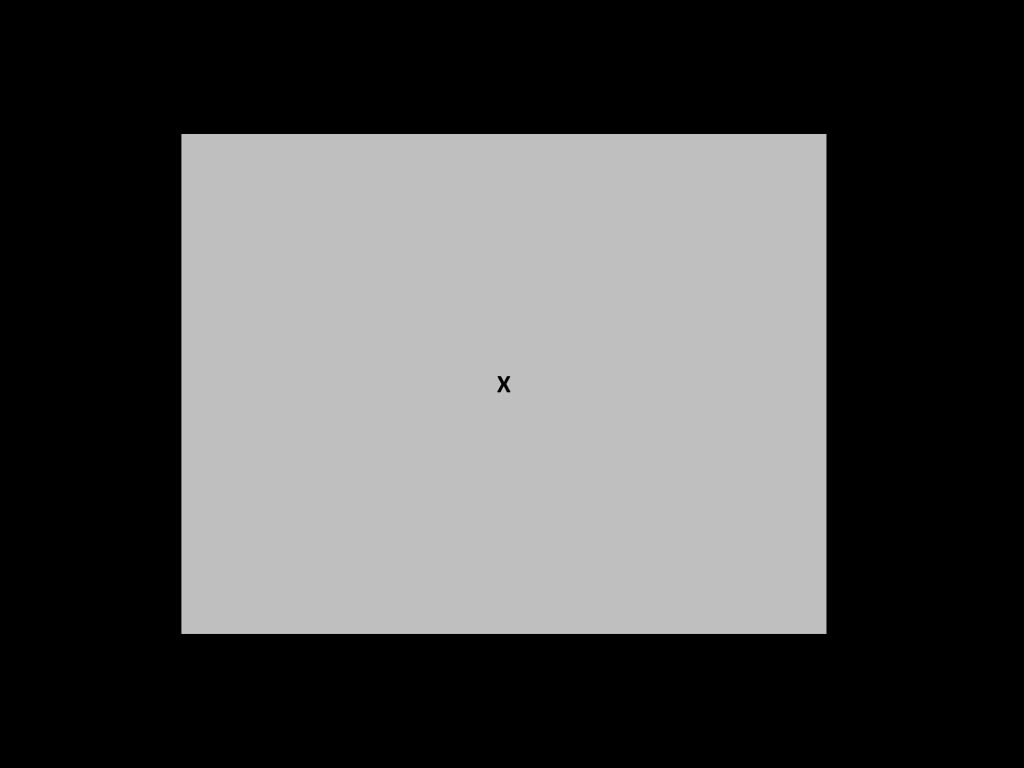

Supplement: S2 File — (ZIP) [file pone.0257717.s002.zip › software/stimuli/InstructionWantTo12.jpg]

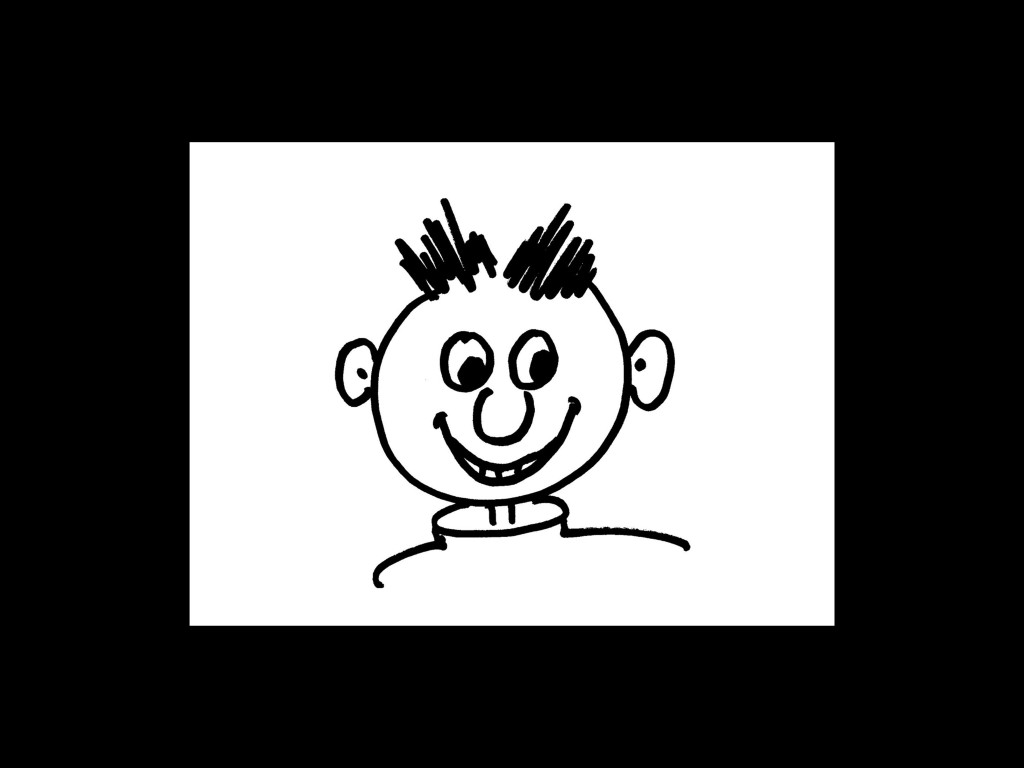

Supplement: S2 File — (ZIP) [file pone.0257717.s002.zip › software/stimuli/InstructionWantTo13.jpg]

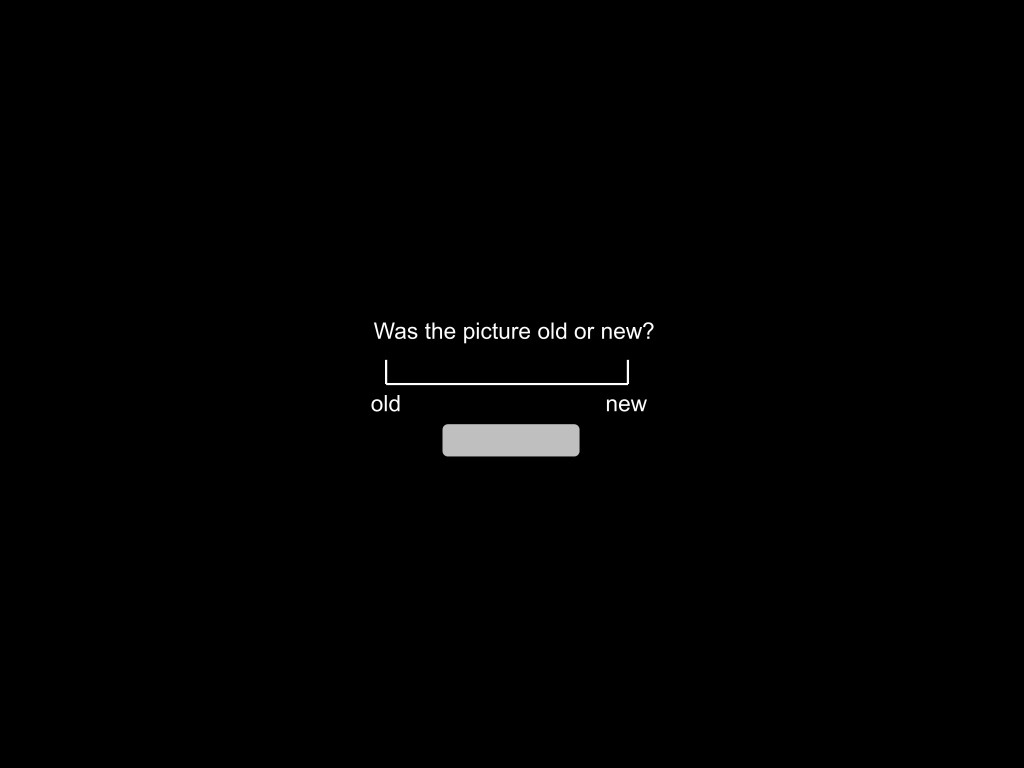

Supplement: S2 File — (ZIP) [file pone.0257717.s002.zip › software/stimuli/InstructionWantTo14.jpg]

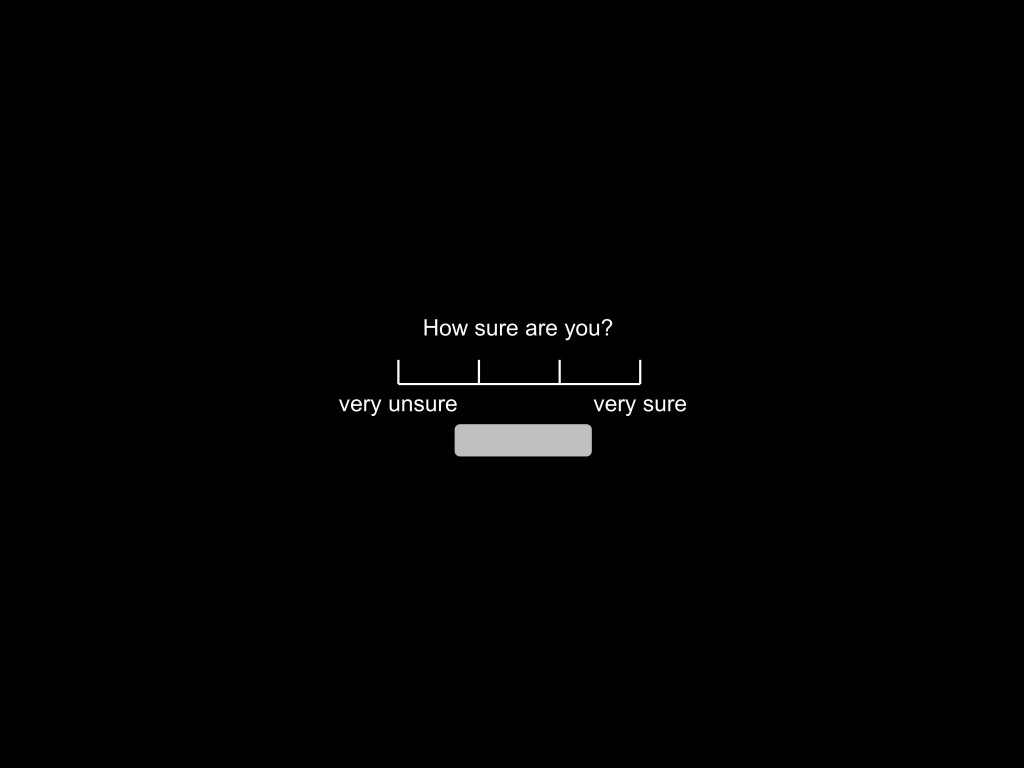

Supplement: S2 File — (ZIP) [file pone.0257717.s002.zip › software/stimuli/InstructionWantTo15.jpg]

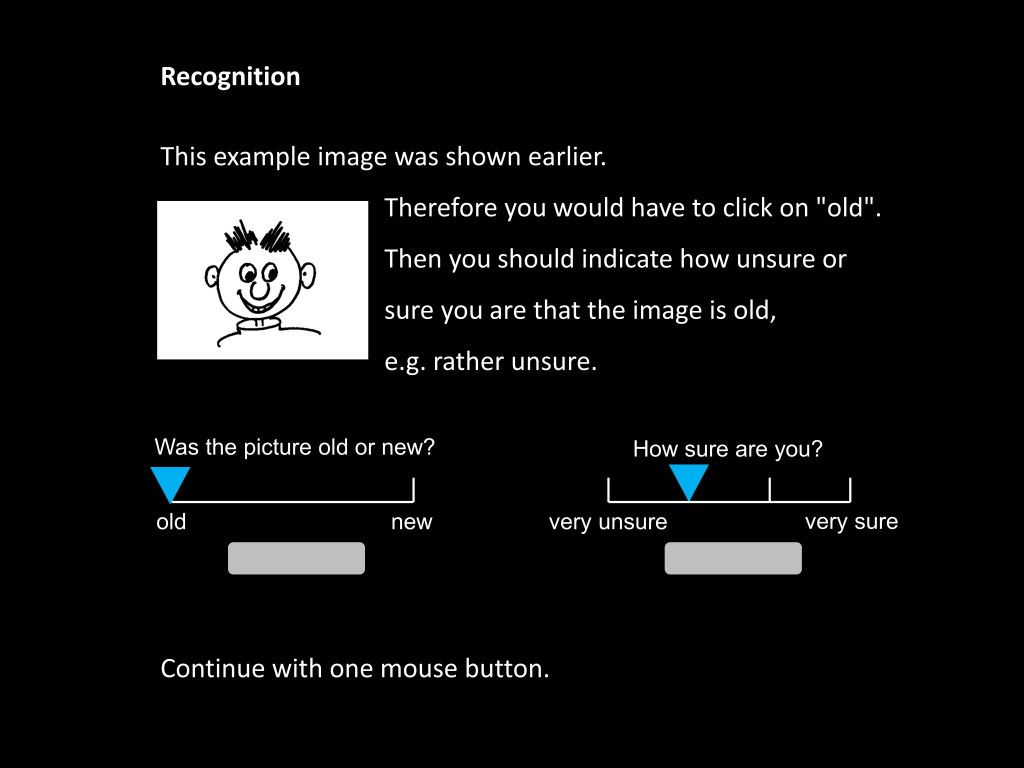

Supplement: S2 File — (ZIP) [file pone.0257717.s002.zip › software/stimuli/InstructionWantTo16.jpg]

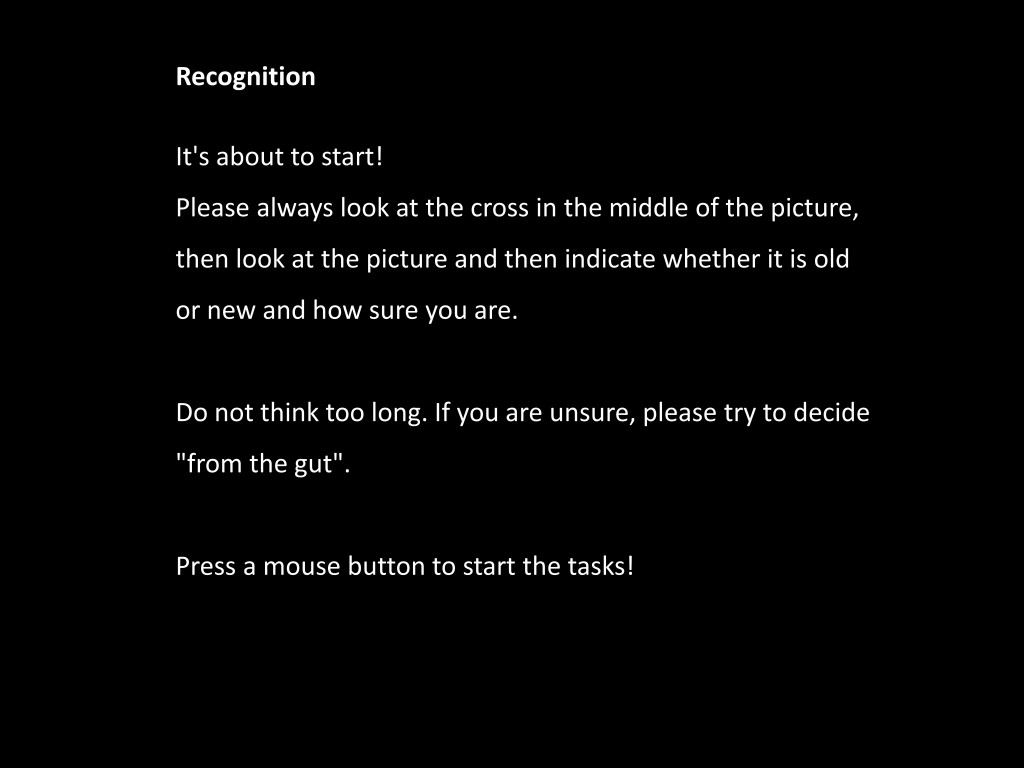

Supplement: S2 File — (ZIP) [file pone.0257717.s002.zip › software/stimuli/InstructionWantTo17.jpg]

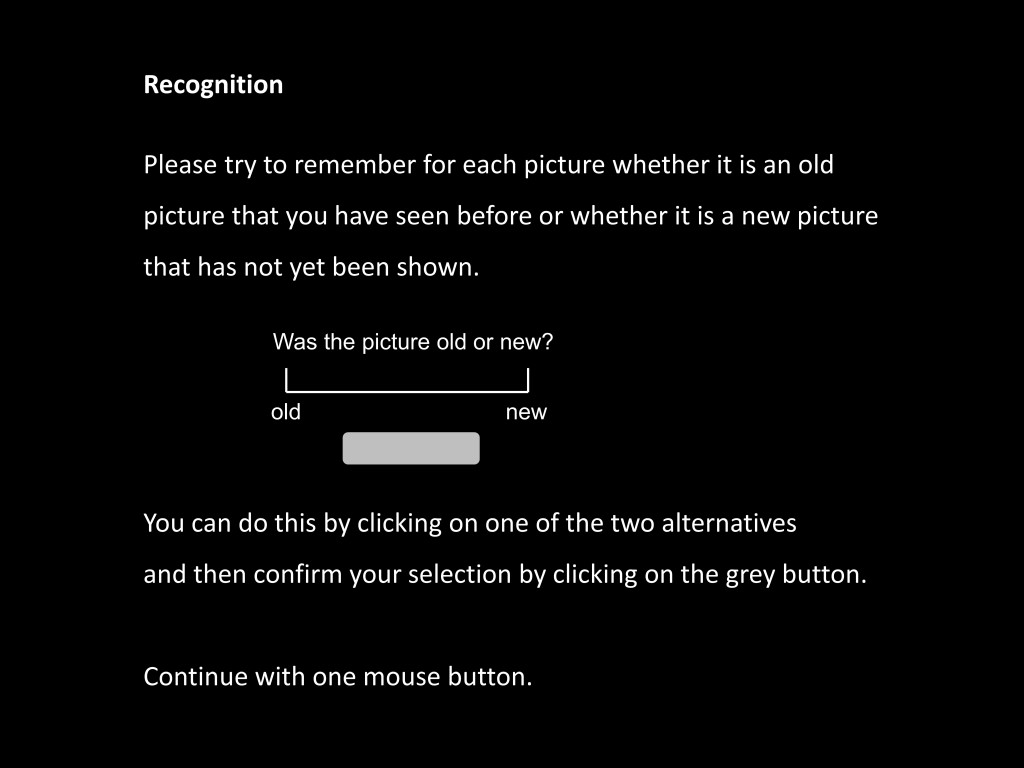

Supplement: S2 File — (ZIP) [file pone.0257717.s002.zip › software/stimuli/InstructionWantTo2.jpg]

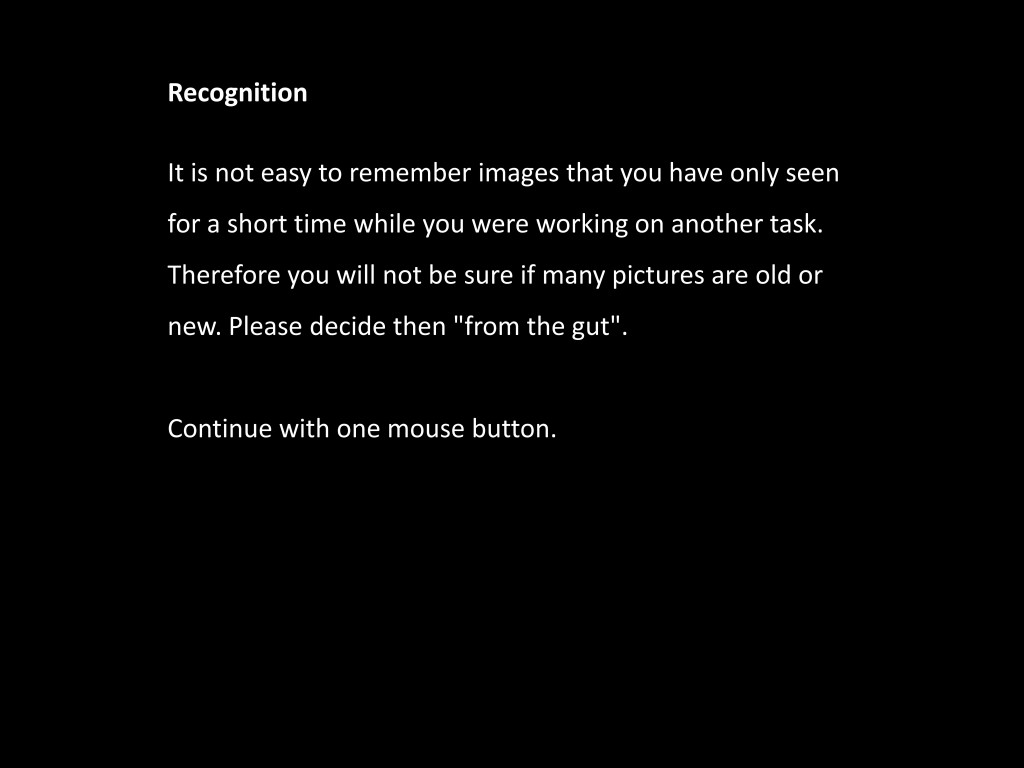

Supplement: S2 File — (ZIP) [file pone.0257717.s002.zip › software/stimuli/InstructionWantTo3.jpg]

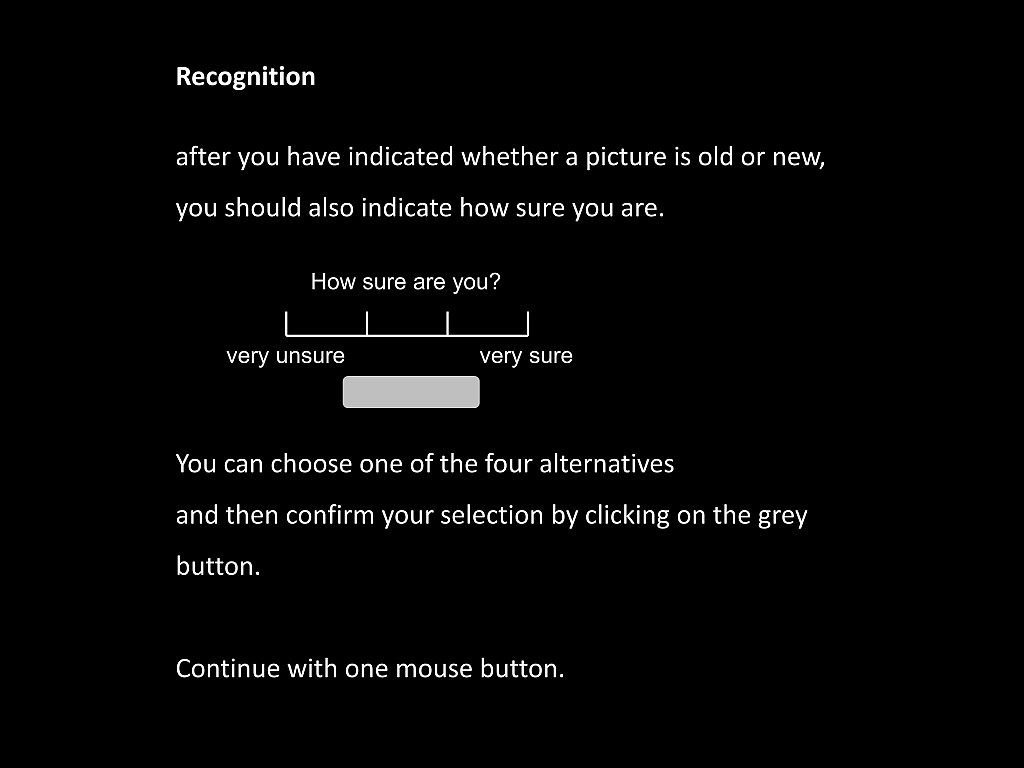

Supplement: S2 File — (ZIP) [file pone.0257717.s002.zip › software/stimuli/InstructionWantTo4.jpg]

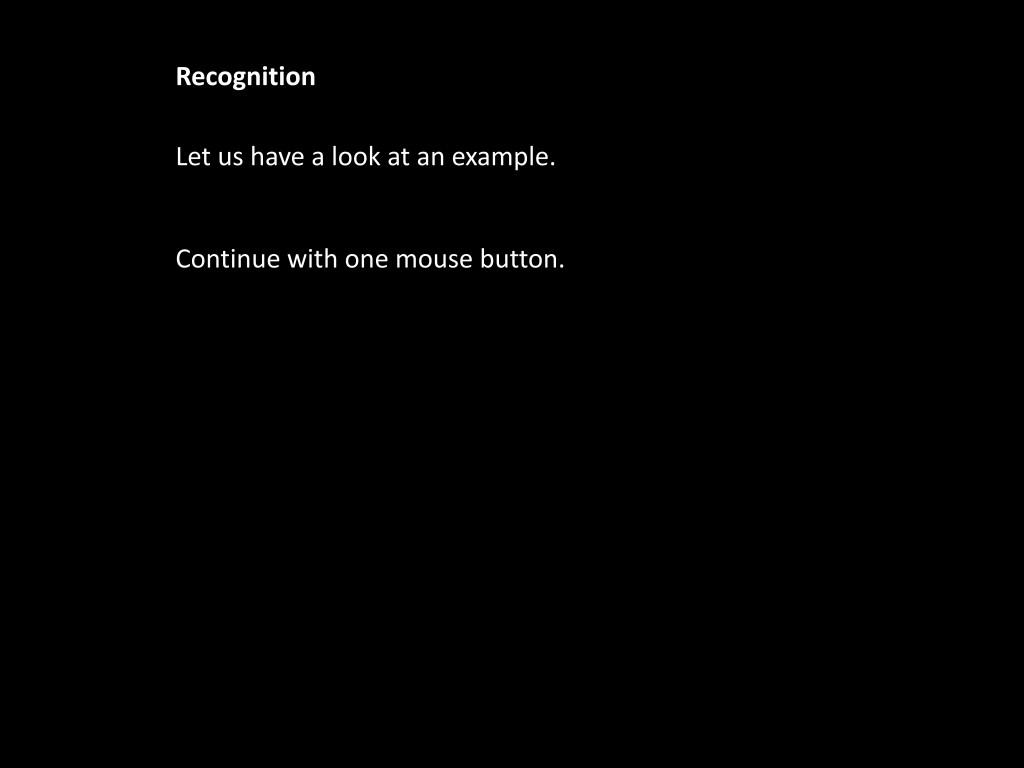

Supplement: S2 File — (ZIP) [file pone.0257717.s002.zip › software/stimuli/InstructionWantTo5.jpg]

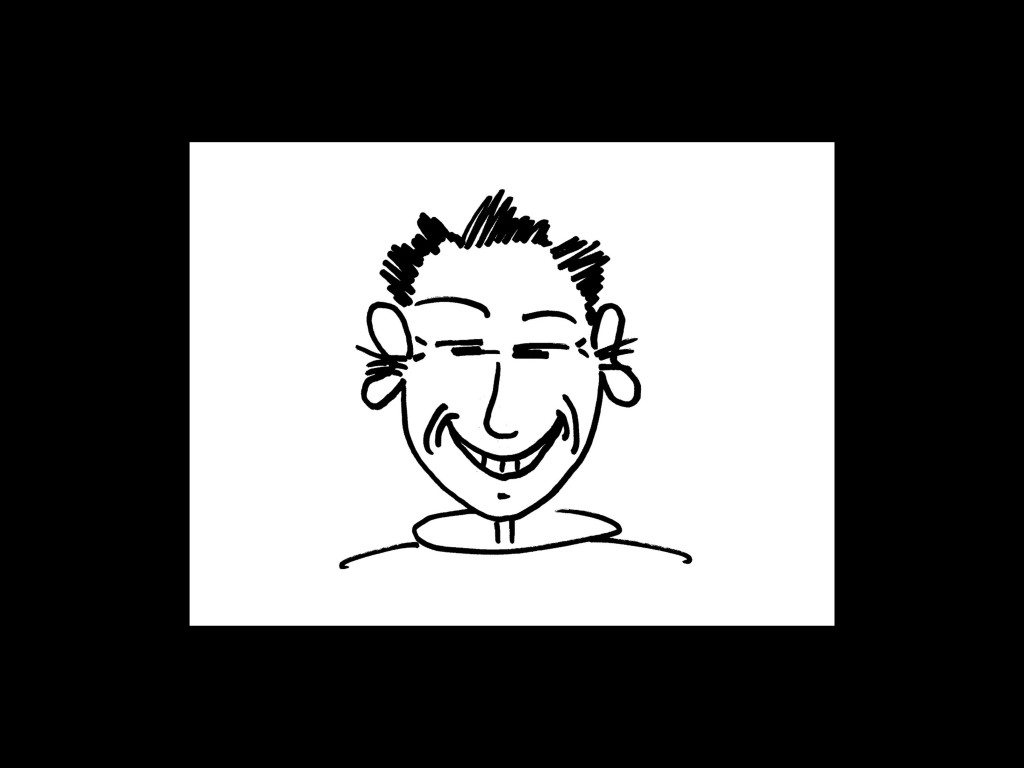

Supplement: S2 File — (ZIP) [file pone.0257717.s002.zip › software/stimuli/InstructionWantTo7.jpg]

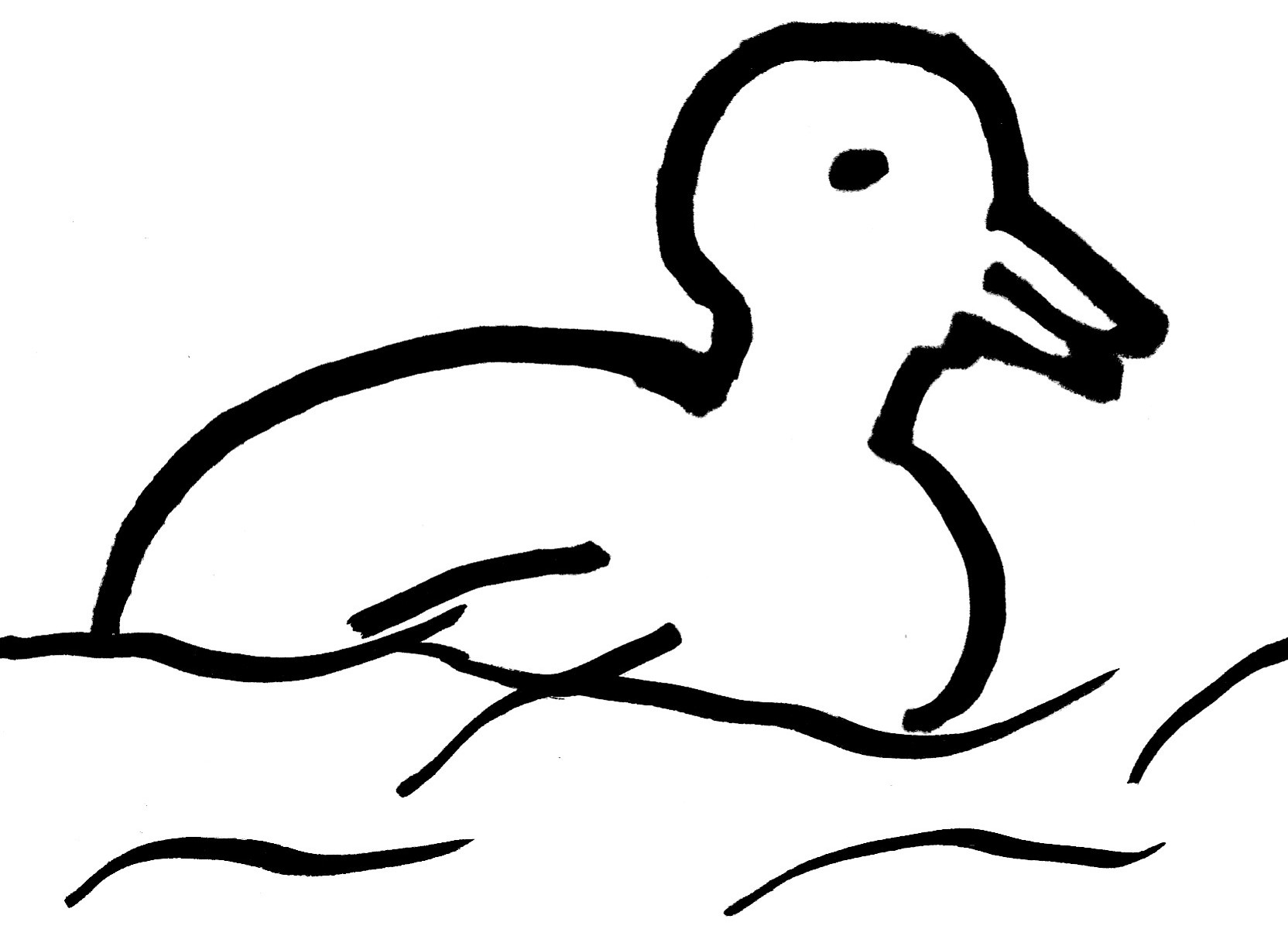

Supplement: S2 File — (ZIP) [file pone.0257717.s002.zip › software/stimuli/sample_pic_duck.jpg]

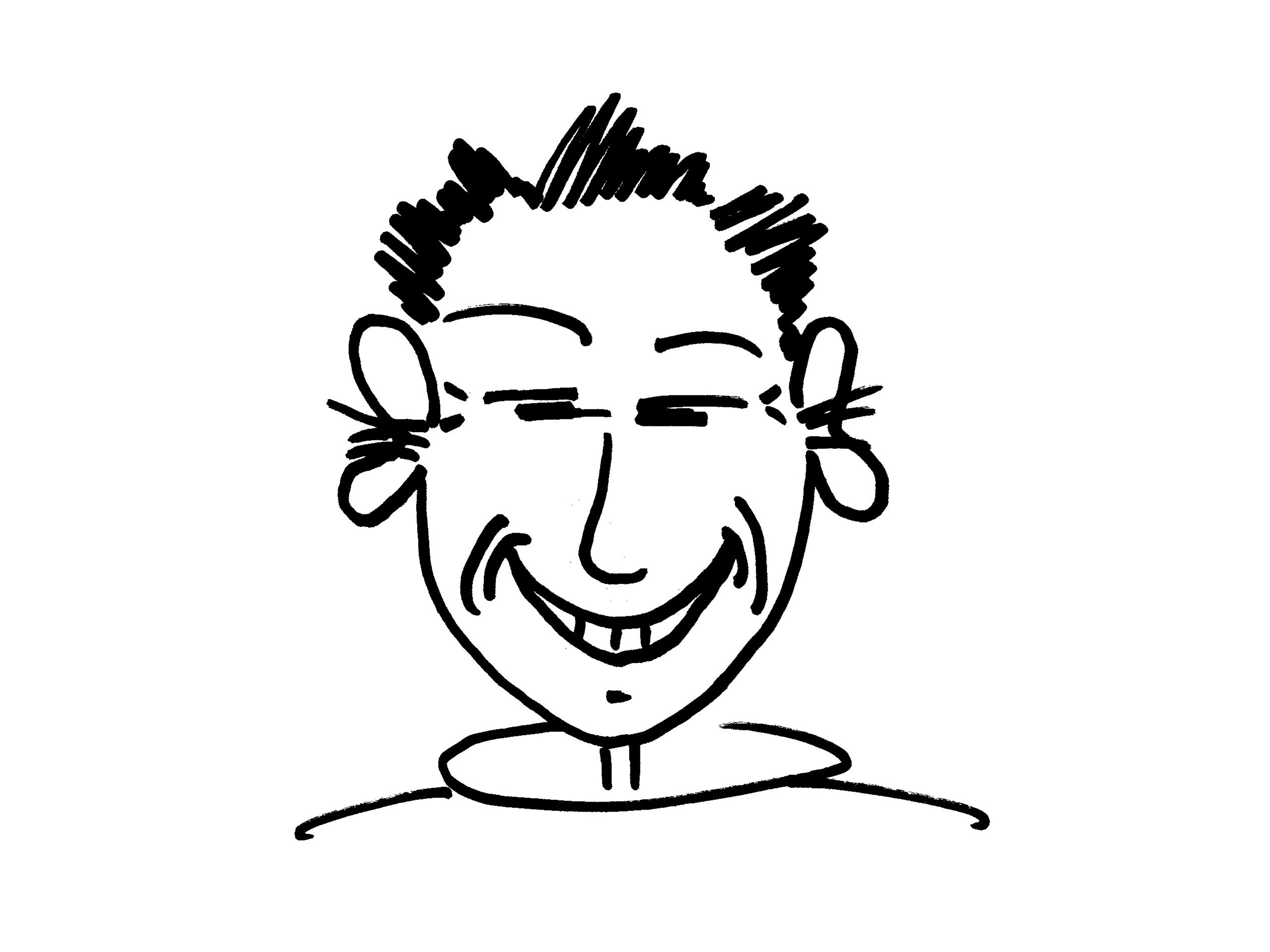

Supplement: S2 File — (ZIP) [file pone.0257717.s002.zip › software/stimuli/sample_pic_face_new.jpg]

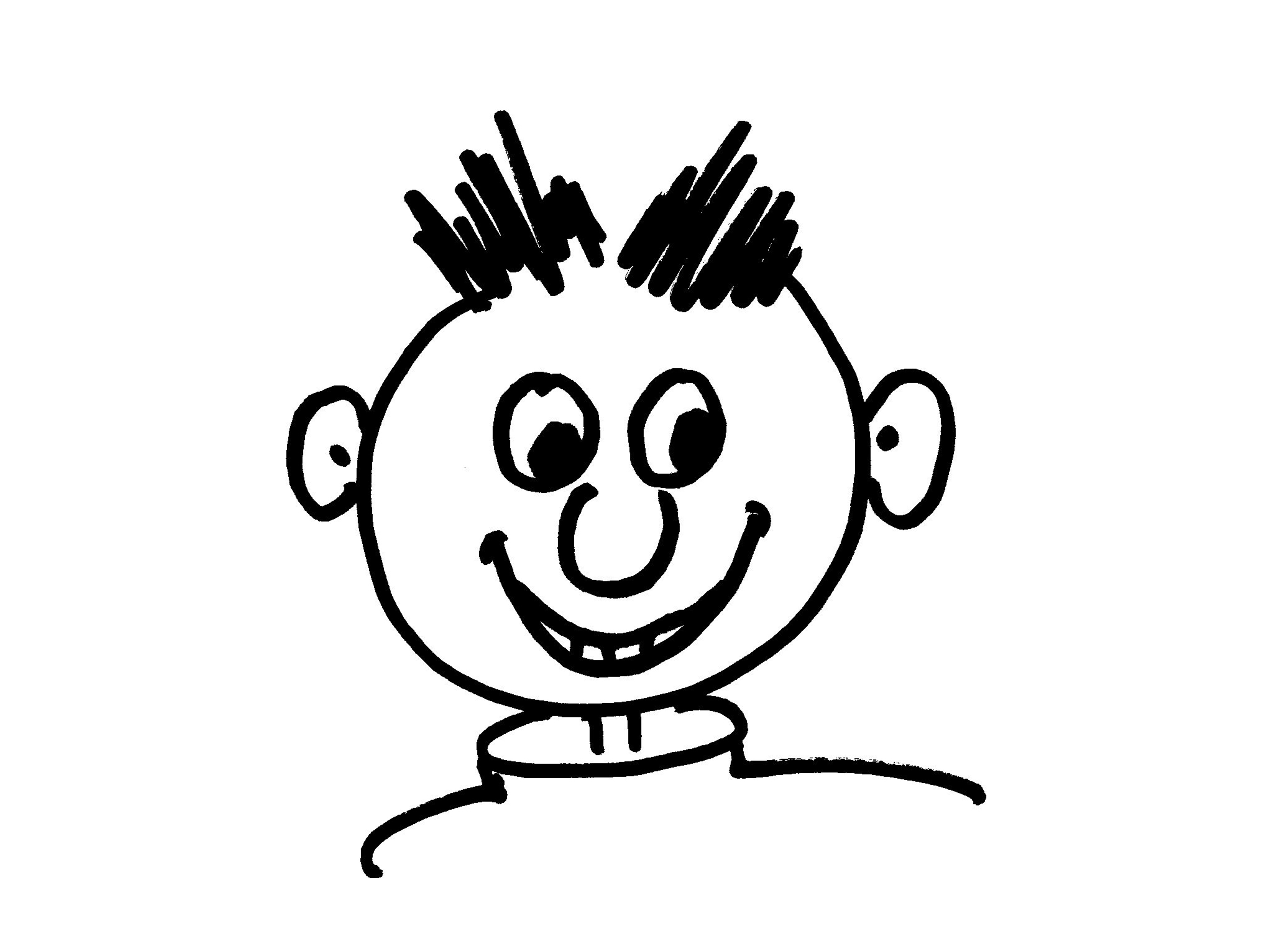

Supplement: S2 File — (ZIP) [file pone.0257717.s002.zip › software/stimuli/sample_pic_face_old.jpg]

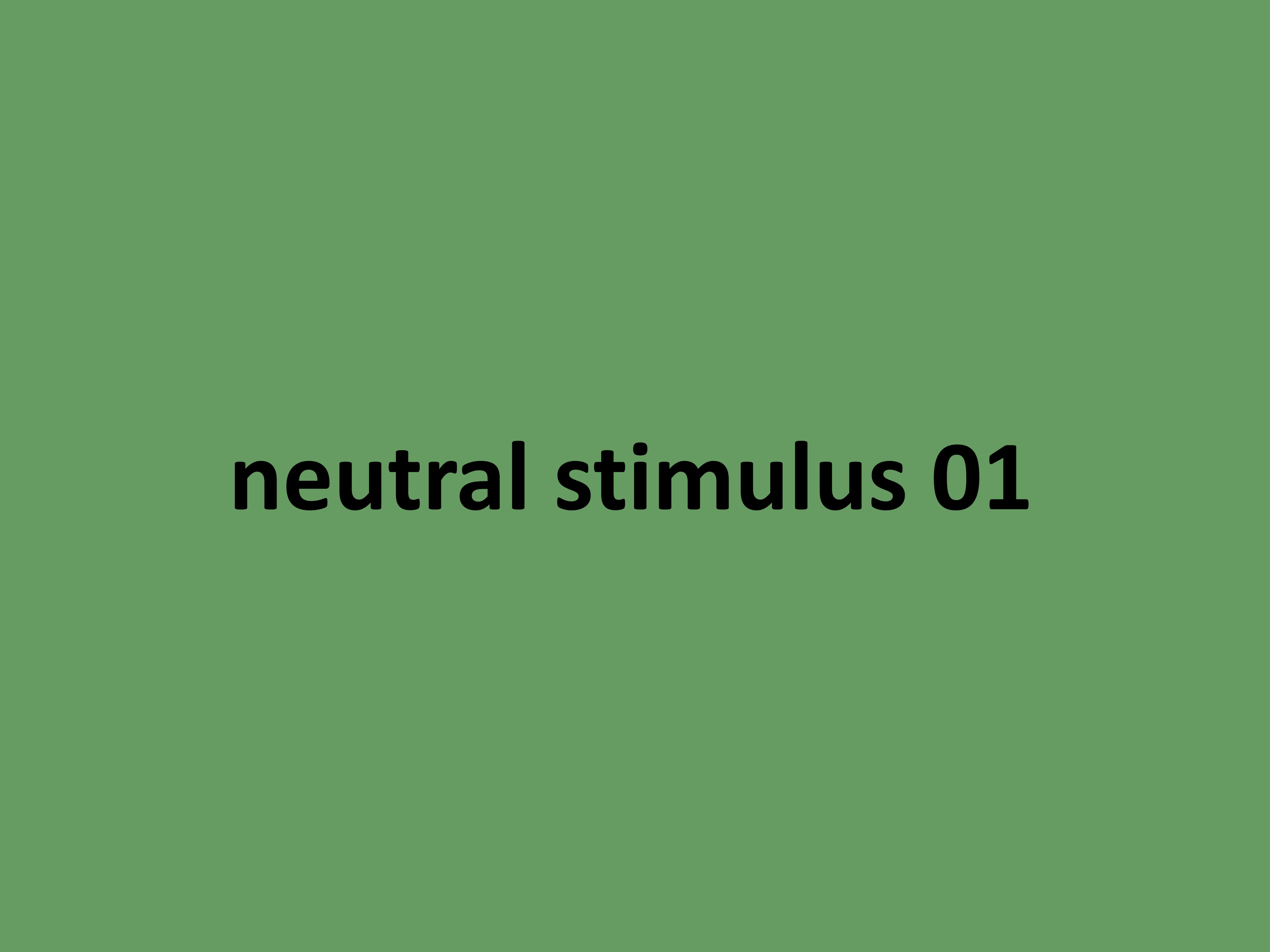

Supplement: S2 File — (ZIP) [file pone.0257717.s002.zip › software/stimuli/stimulus_neutral_01.jpg]

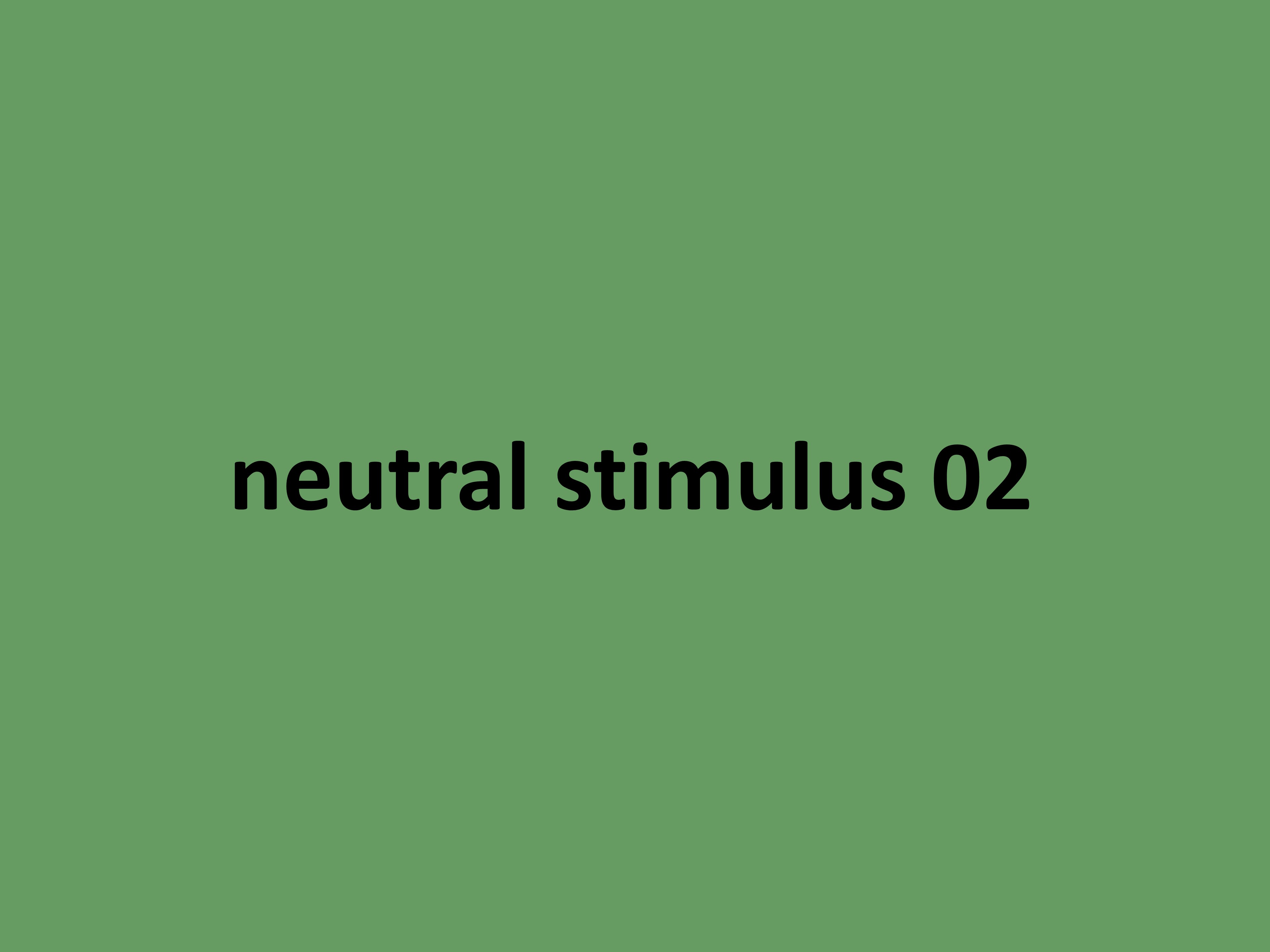

Supplement: S2 File — (ZIP) [file pone.0257717.s002.zip › software/stimuli/stimulus_neutral_02.jpg]

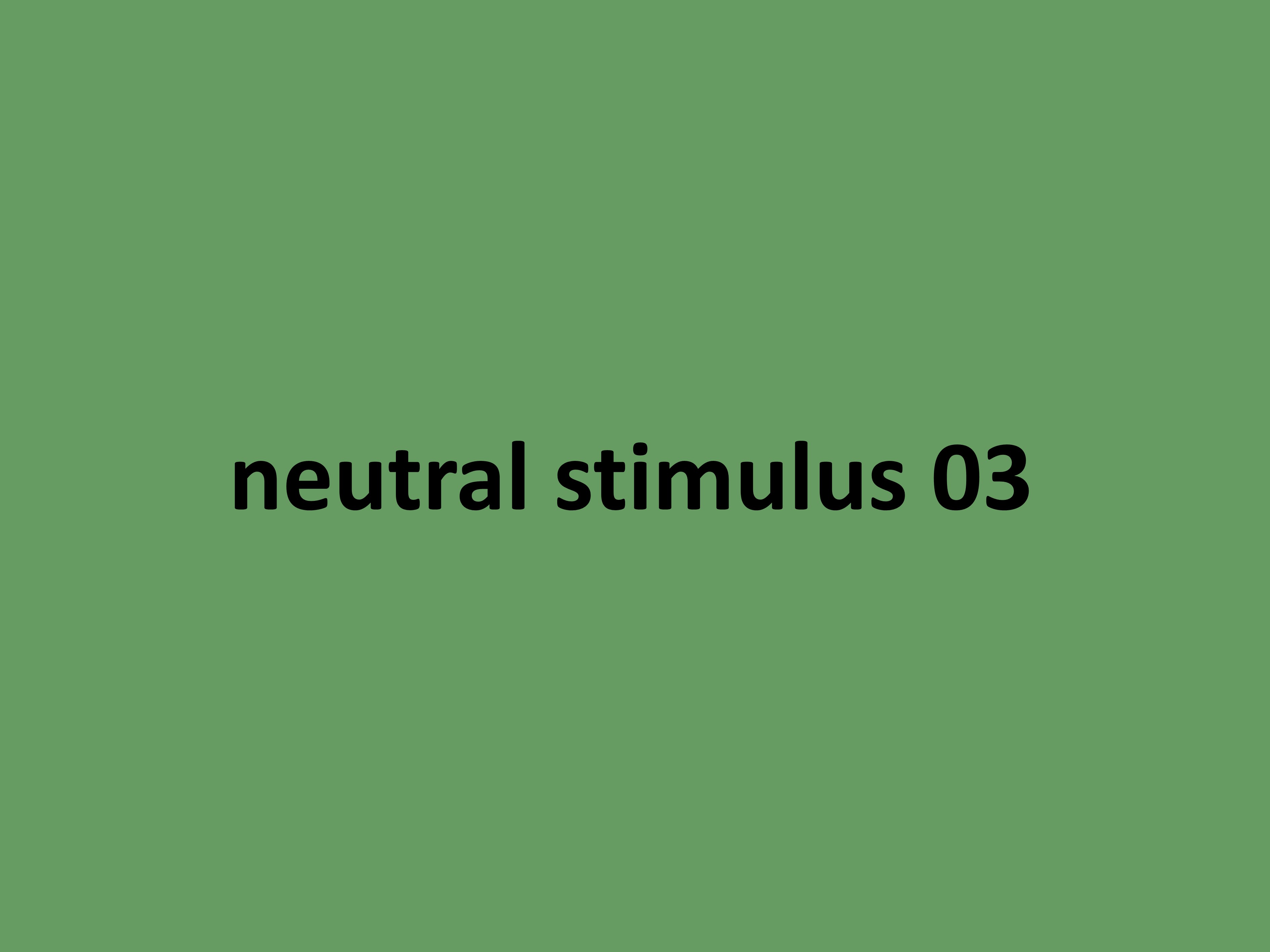

Supplement: S2 File — (ZIP) [file pone.0257717.s002.zip › software/stimuli/stimulus_neutral_03.jpg]

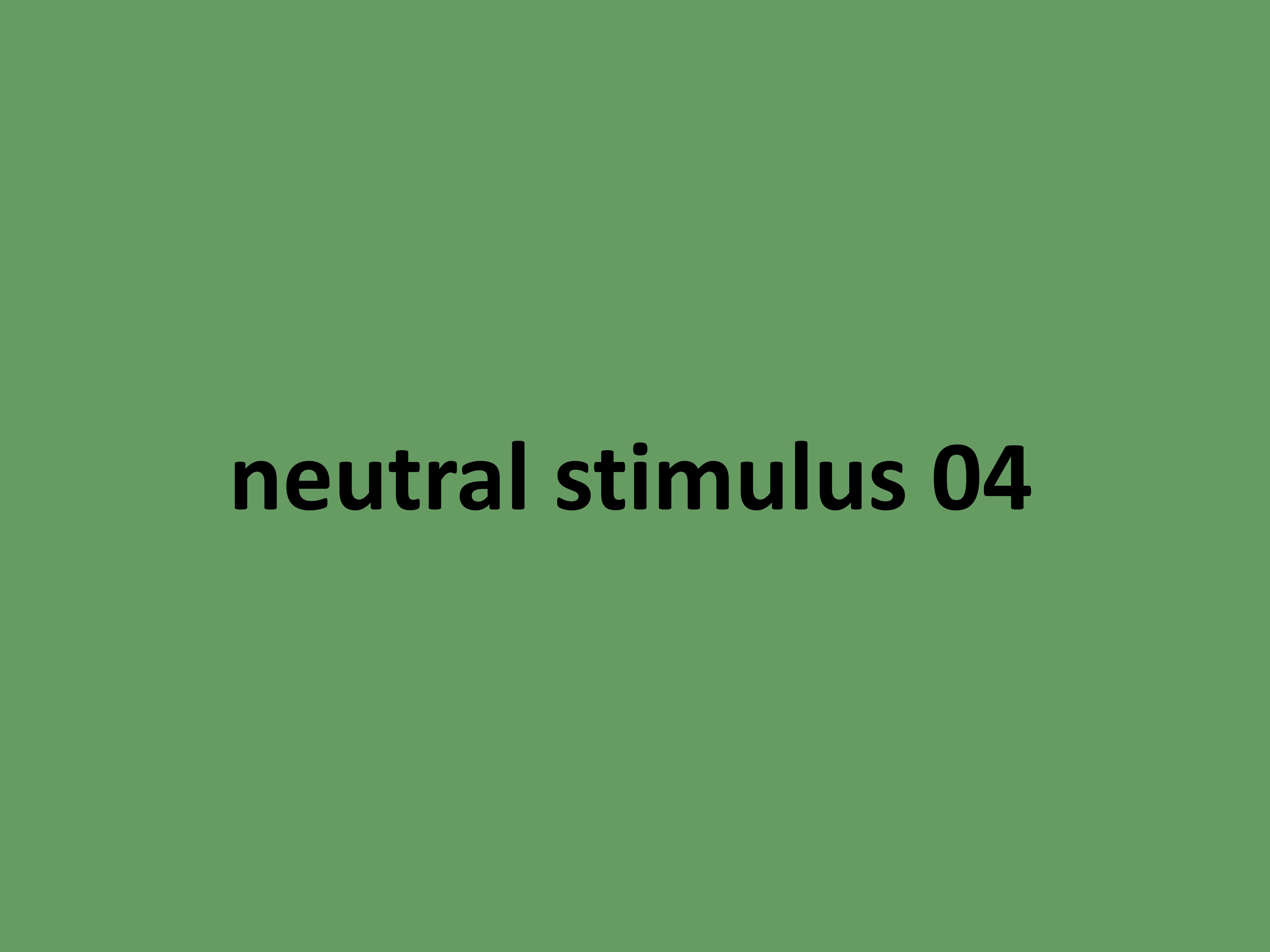

Supplement: S2 File — (ZIP) [file pone.0257717.s002.zip › software/stimuli/stimulus_neutral_04.jpg]

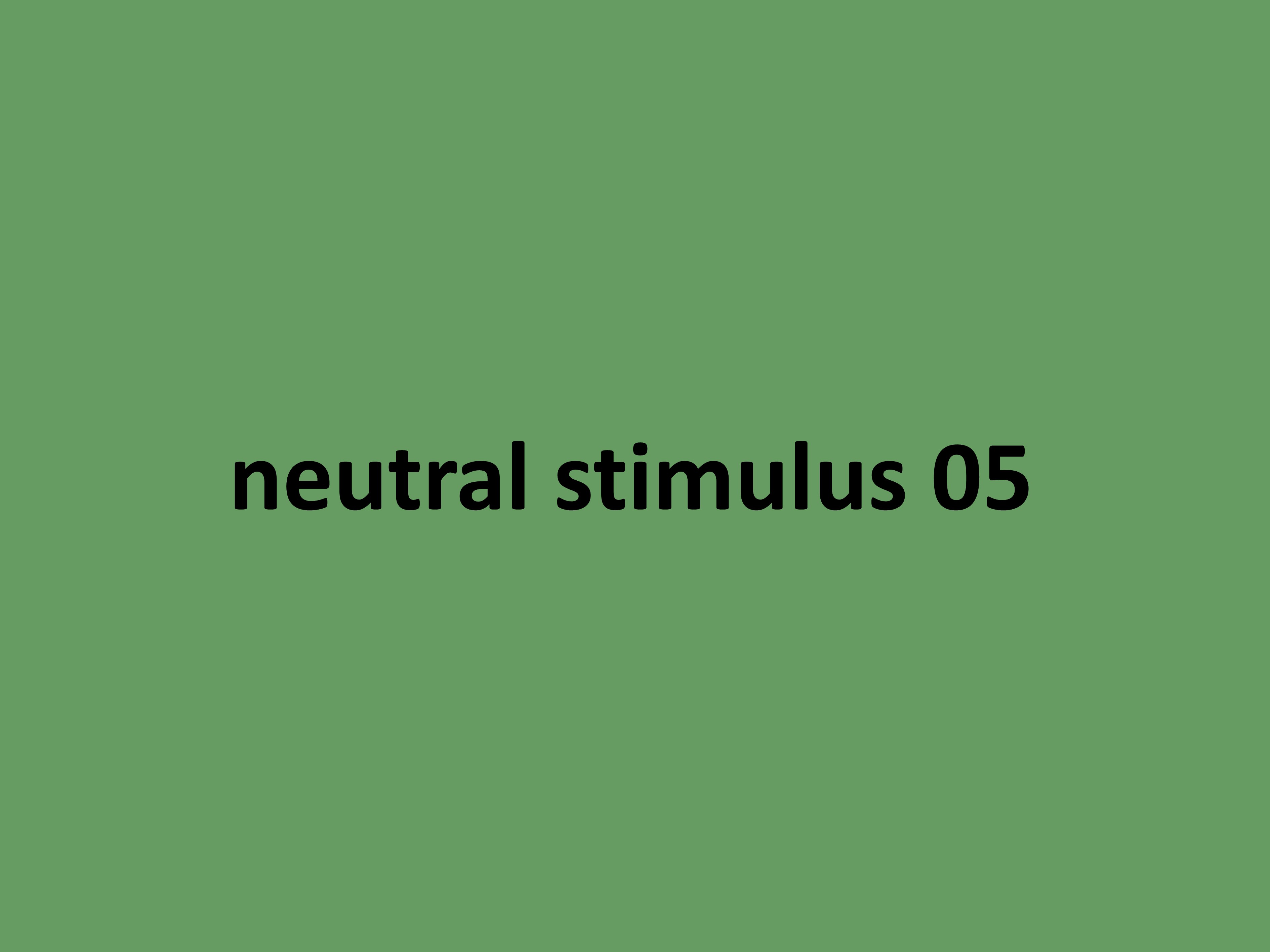

Supplement: S2 File — (ZIP) [file pone.0257717.s002.zip › software/stimuli/stimulus_neutral_05.jpg]

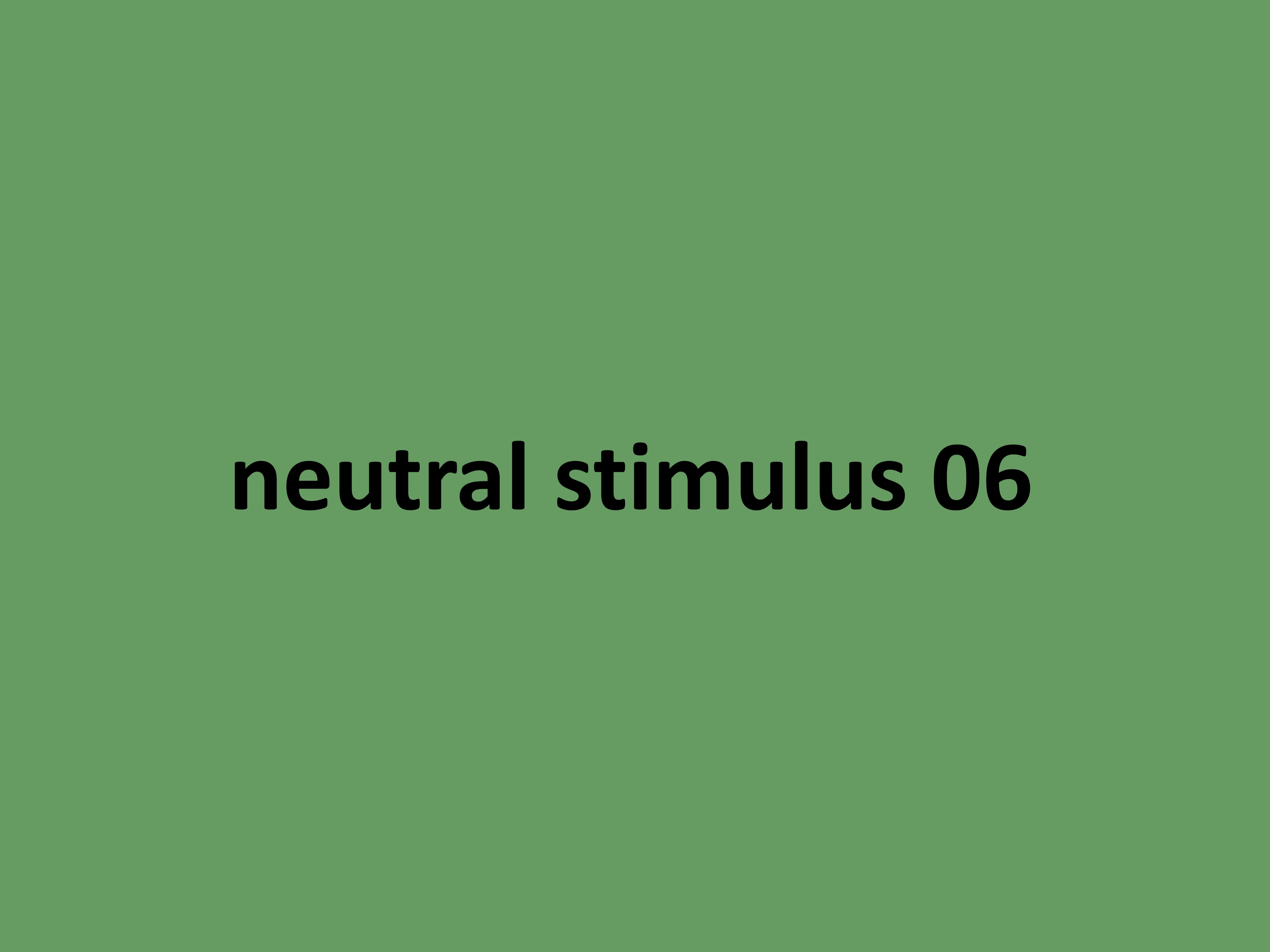

Supplement: S2 File — (ZIP) [file pone.0257717.s002.zip › software/stimuli/stimulus_neutral_06.jpg]

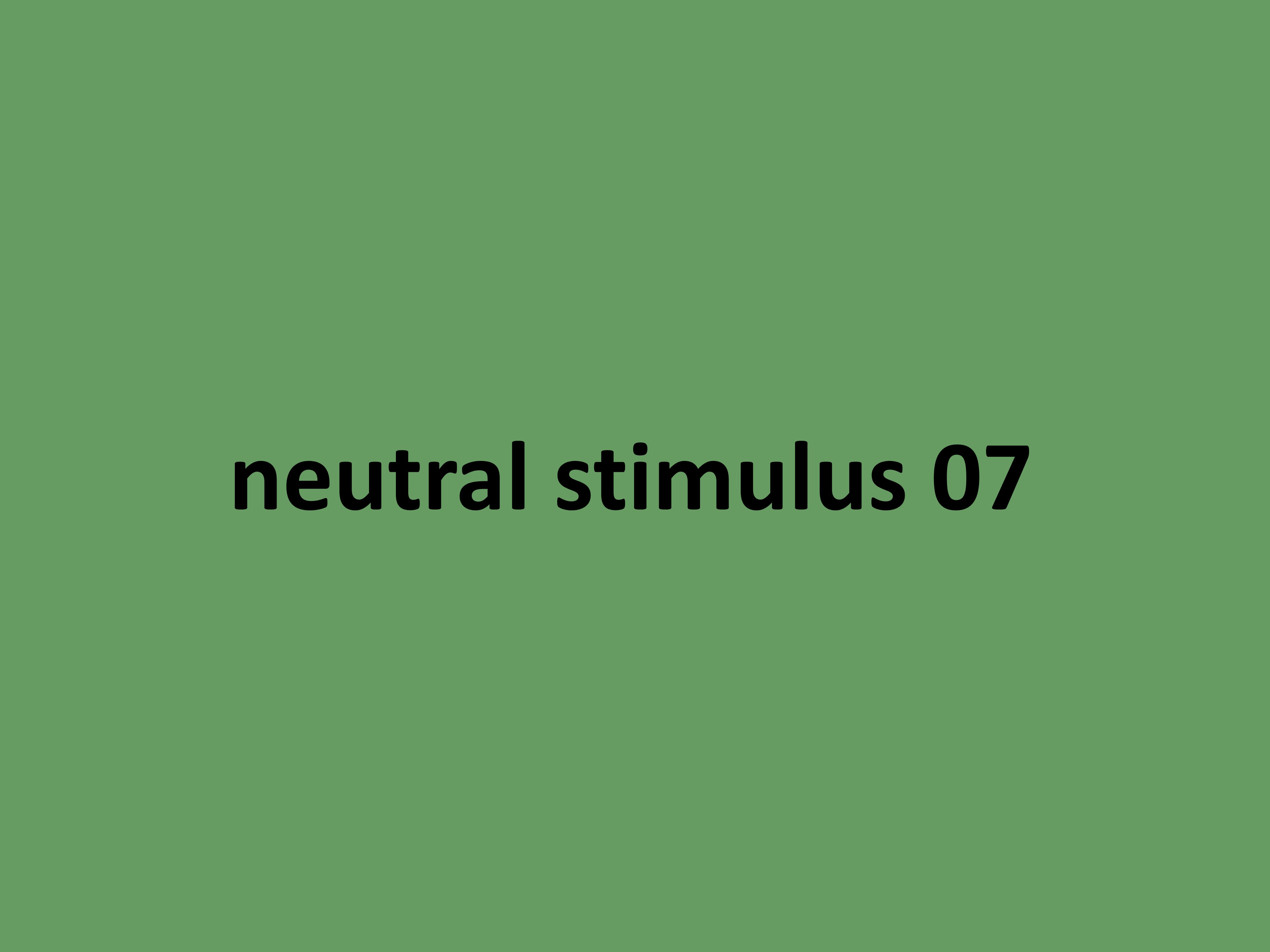

Supplement: S2 File — (ZIP) [file pone.0257717.s002.zip › software/stimuli/stimulus_neutral_07.jpg]

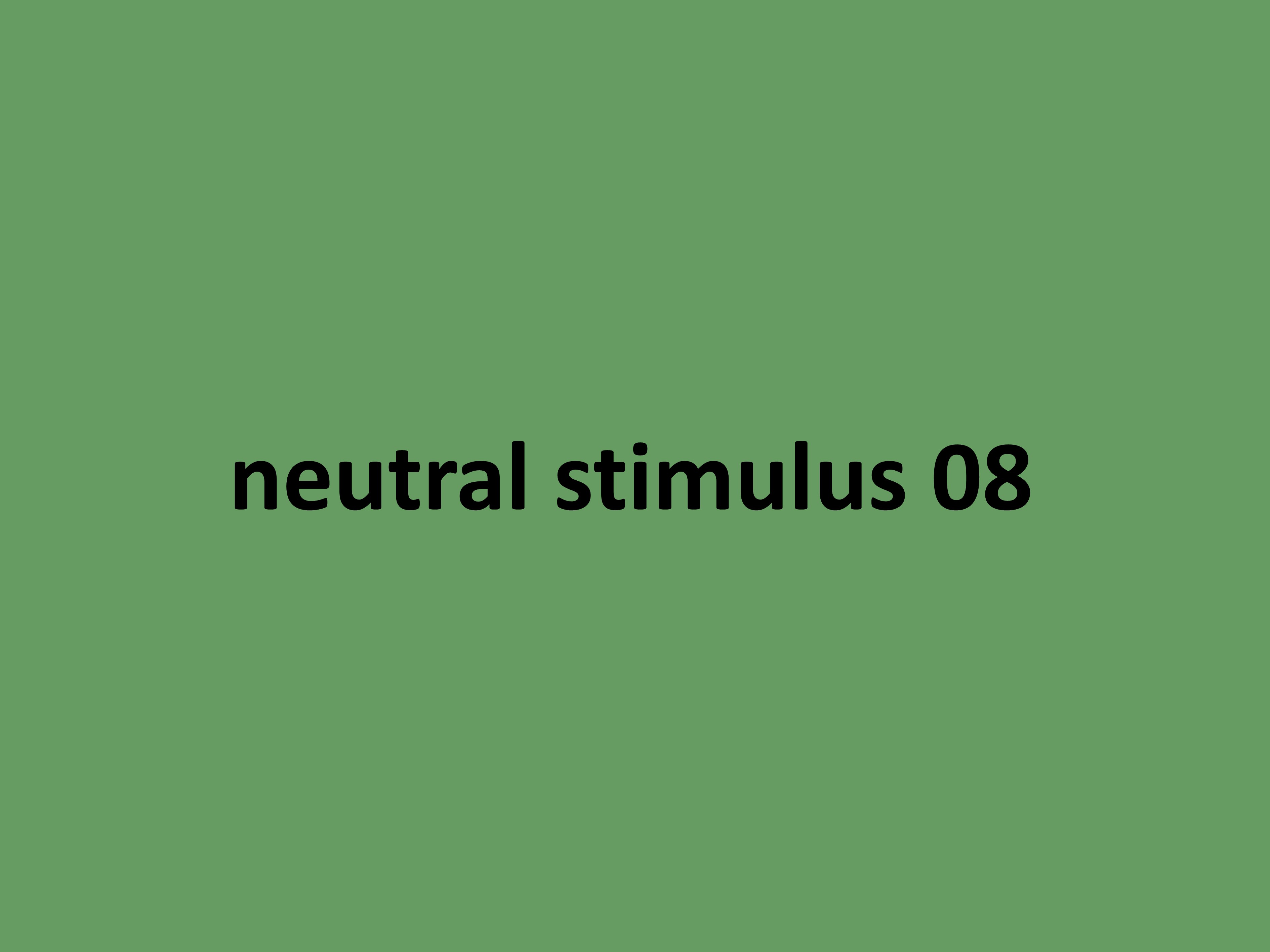

Supplement: S2 File — (ZIP) [file pone.0257717.s002.zip › software/stimuli/stimulus_neutral_08.jpg]

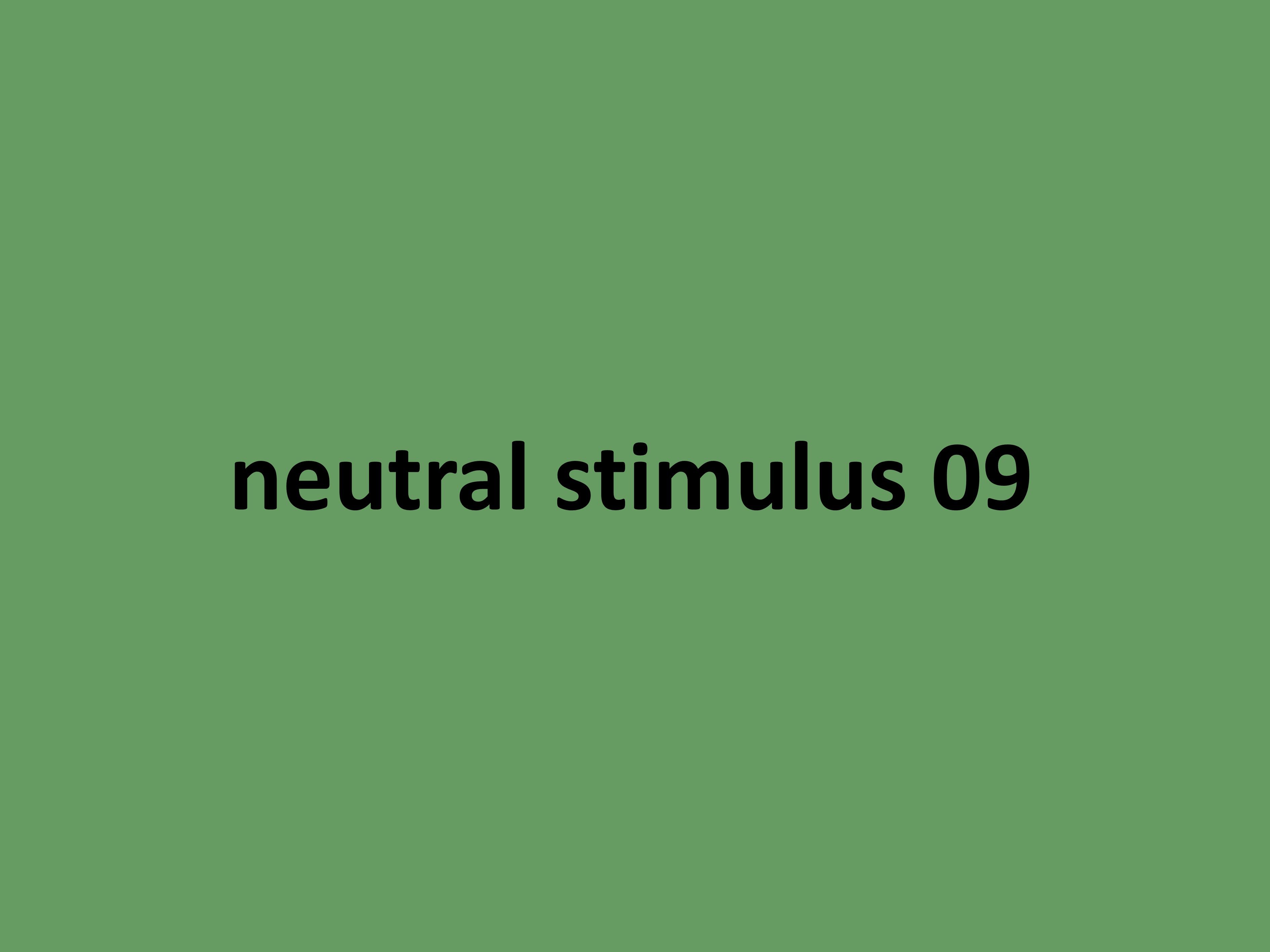

Supplement: S2 File — (ZIP) [file pone.0257717.s002.zip › software/stimuli/stimulus_neutral_09.jpg]

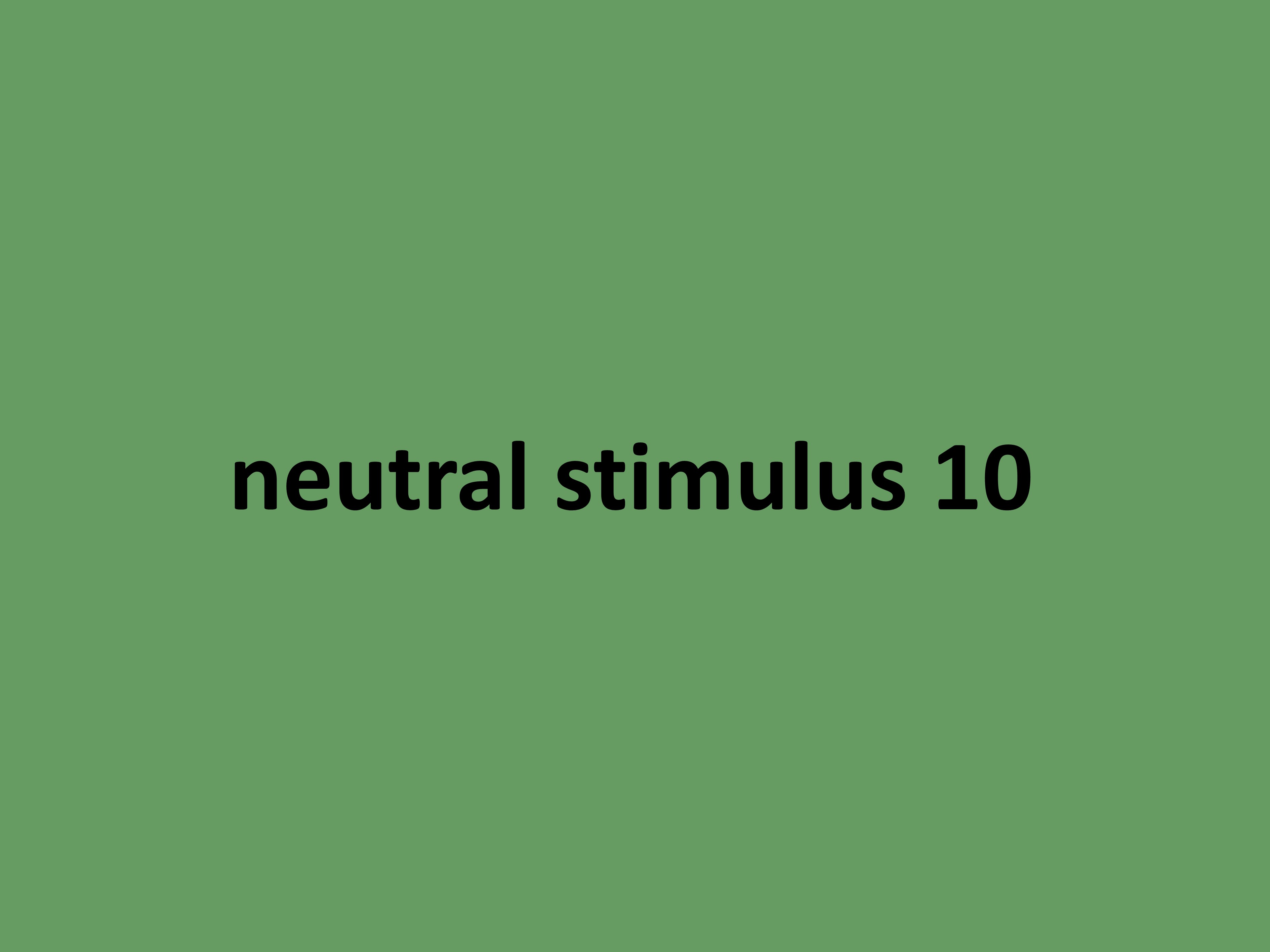

Supplement: S2 File — (ZIP) [file pone.0257717.s002.zip › software/stimuli/stimulus_neutral_10.jpg]

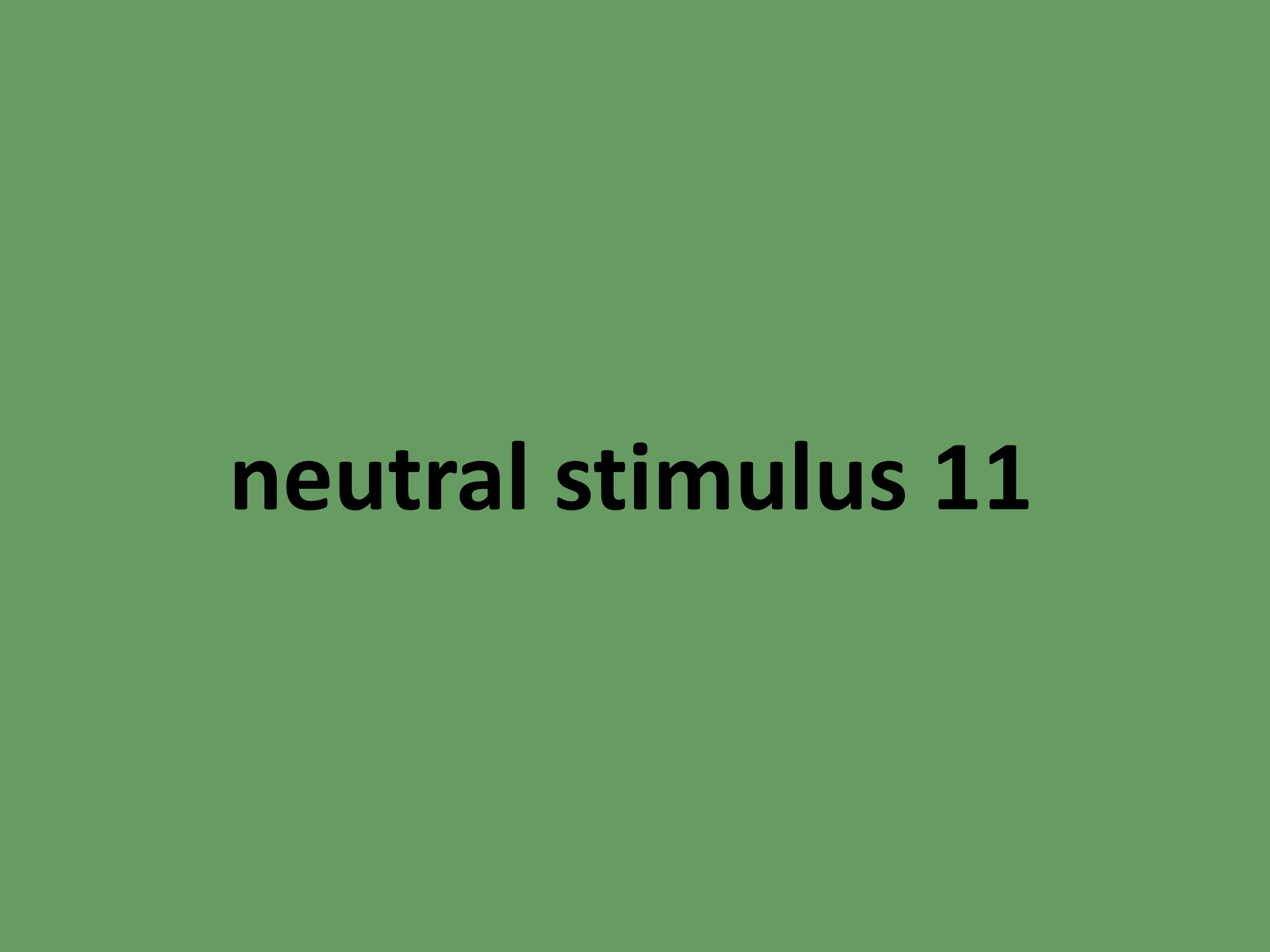

Supplement: S2 File — (ZIP) [file pone.0257717.s002.zip › software/stimuli/stimulus_neutral_11.jpg]

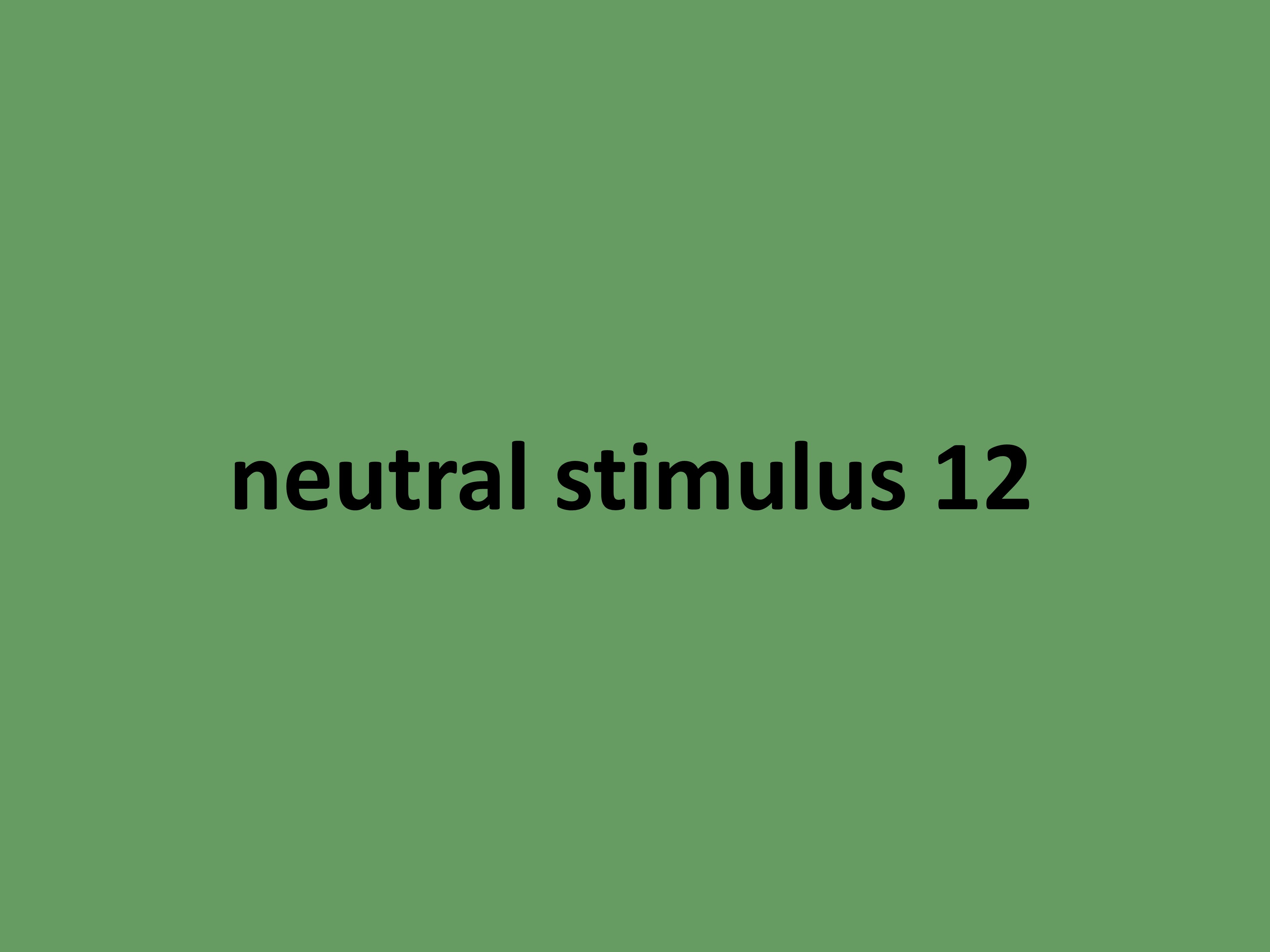

Supplement: S2 File — (ZIP) [file pone.0257717.s002.zip › software/stimuli/stimulus_neutral_12.jpg]

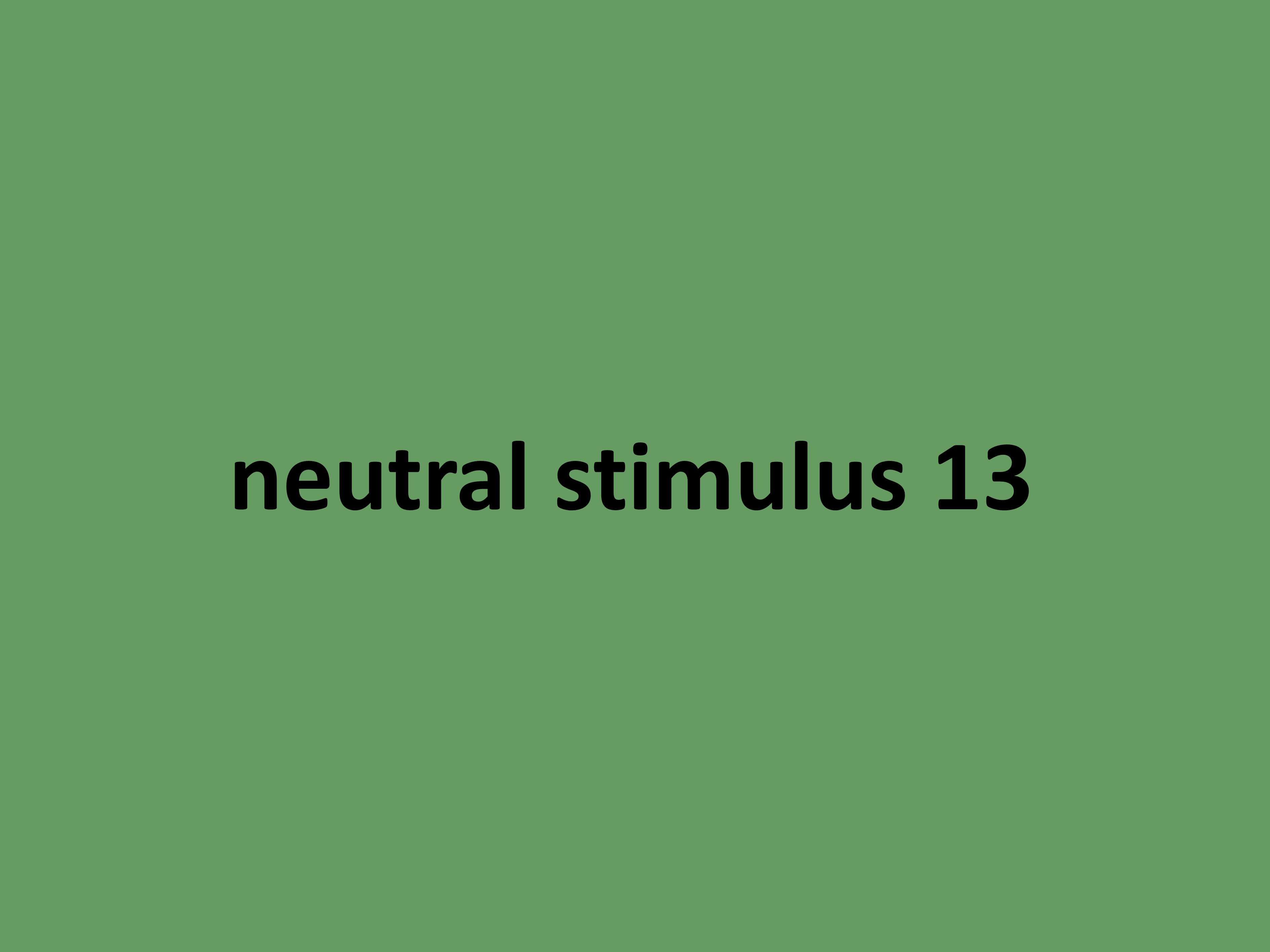

Supplement: S2 File — (ZIP) [file pone.0257717.s002.zip › software/stimuli/stimulus_neutral_13.jpg]

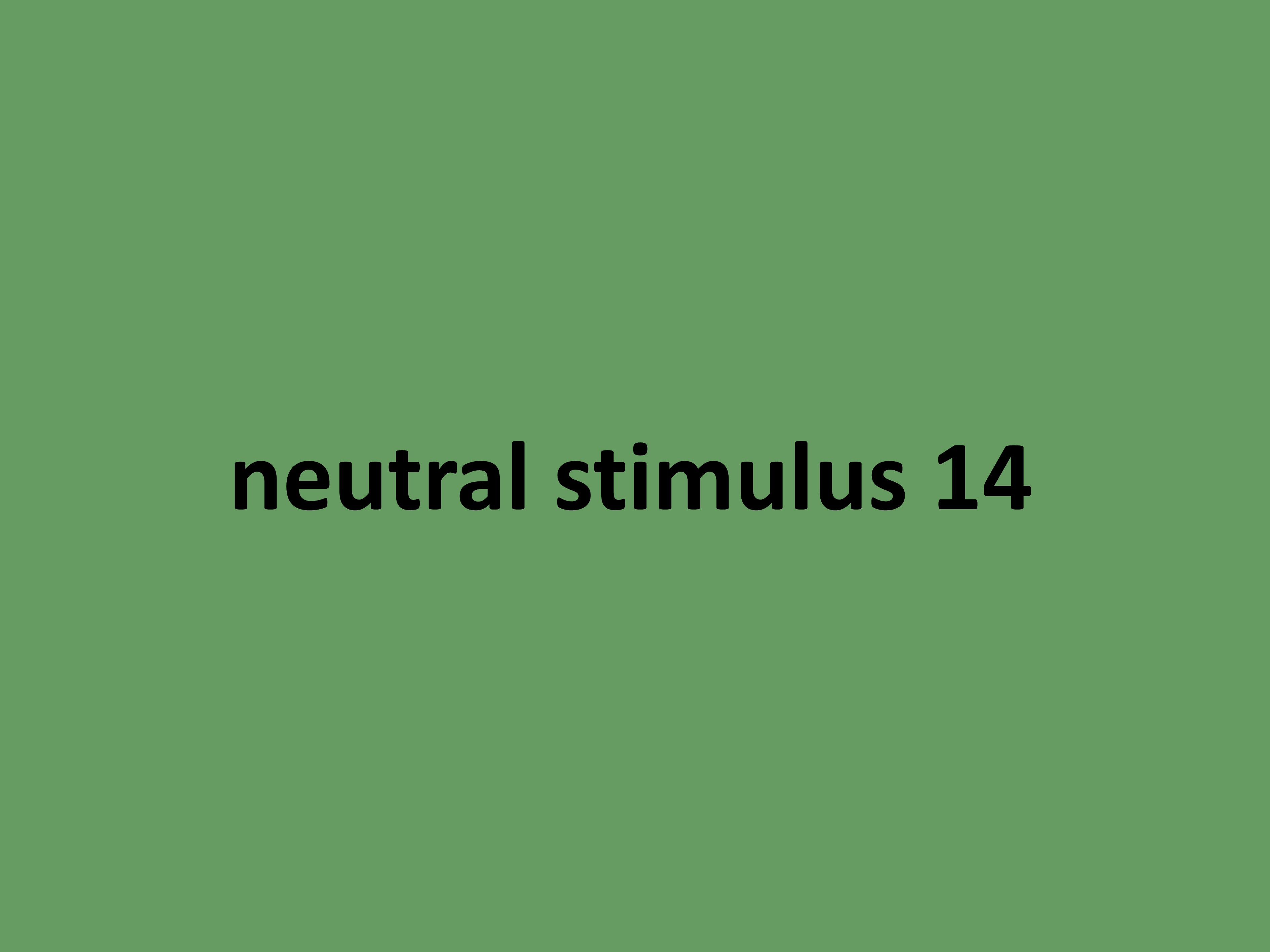

Supplement: S2 File — (ZIP) [file pone.0257717.s002.zip › software/stimuli/stimulus_neutral_14.jpg]

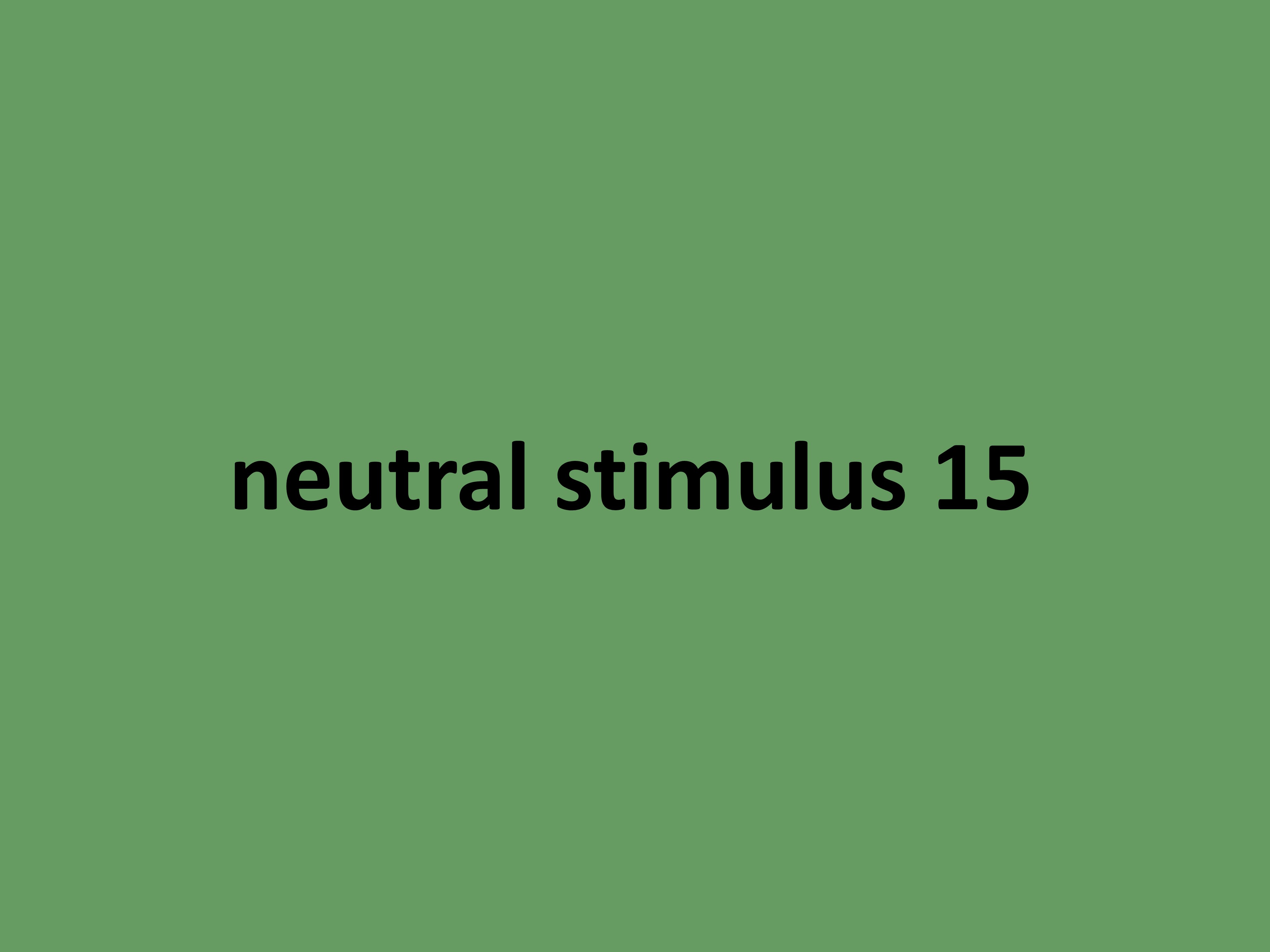

Supplement: S2 File — (ZIP) [file pone.0257717.s002.zip › software/stimuli/stimulus_neutral_15.jpg]

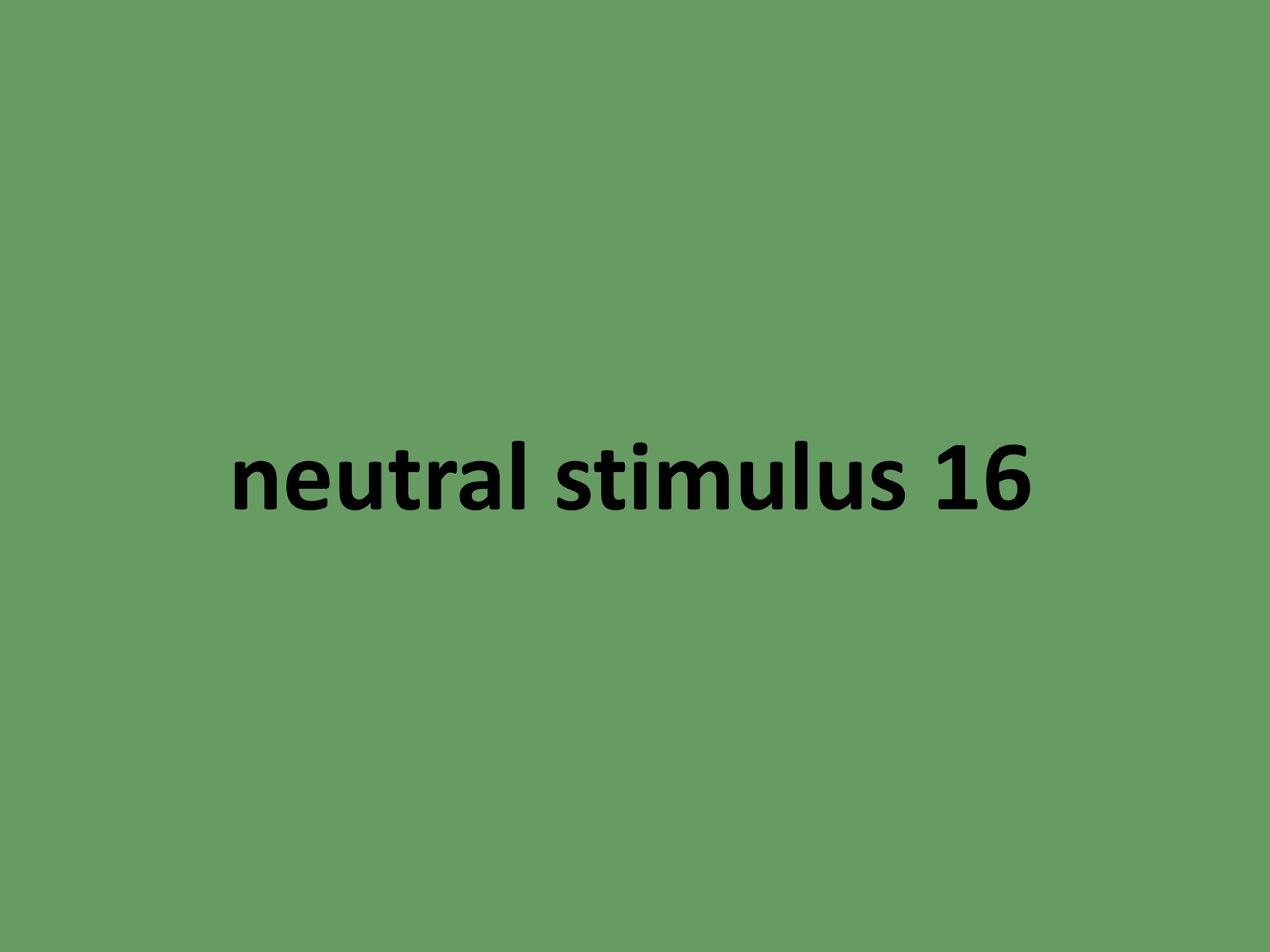

Supplement: S2 File — (ZIP) [file pone.0257717.s002.zip › software/stimuli/stimulus_neutral_16.jpg]

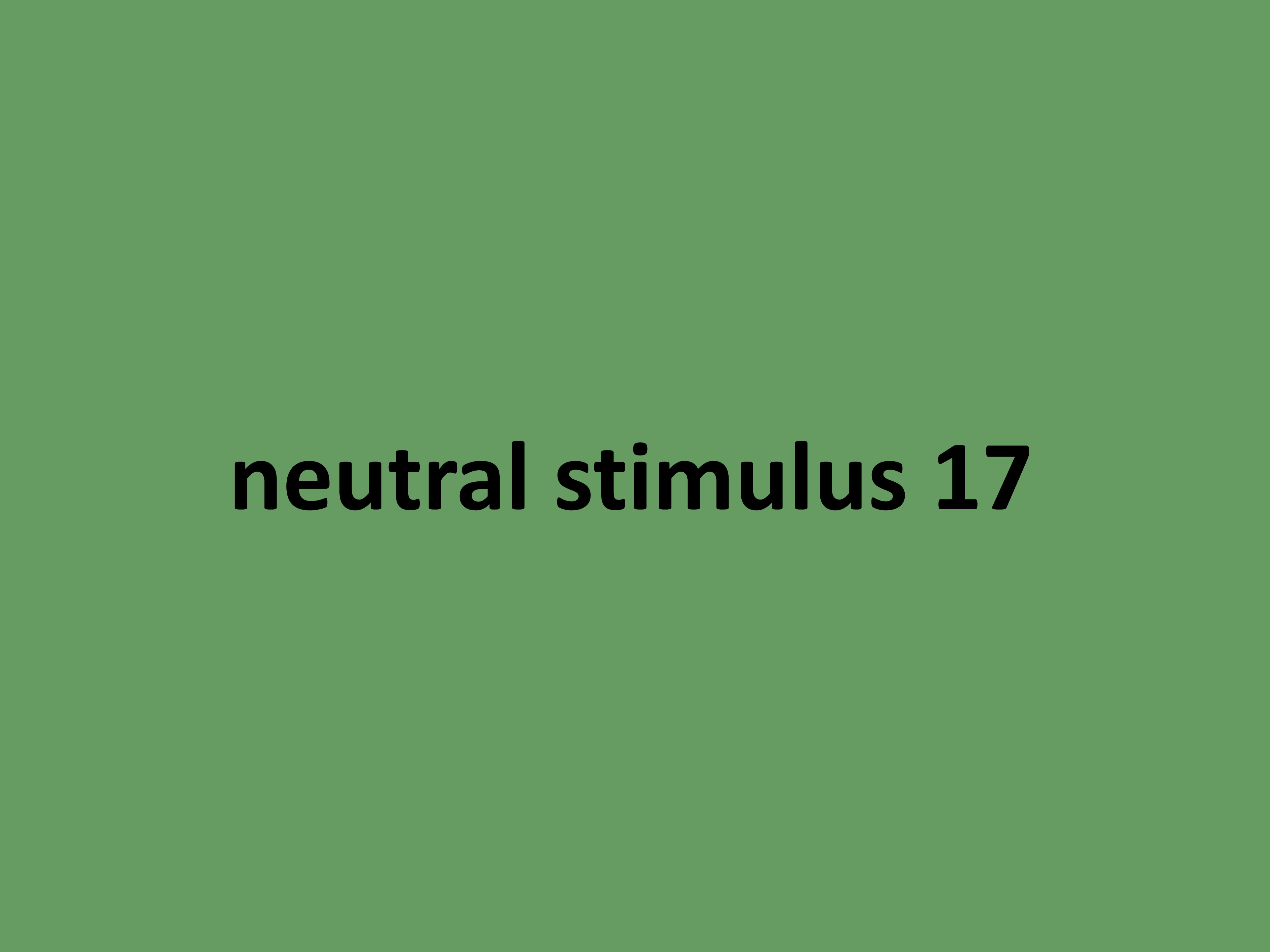

Supplement: S2 File — (ZIP) [file pone.0257717.s002.zip › software/stimuli/stimulus_neutral_17.jpg]

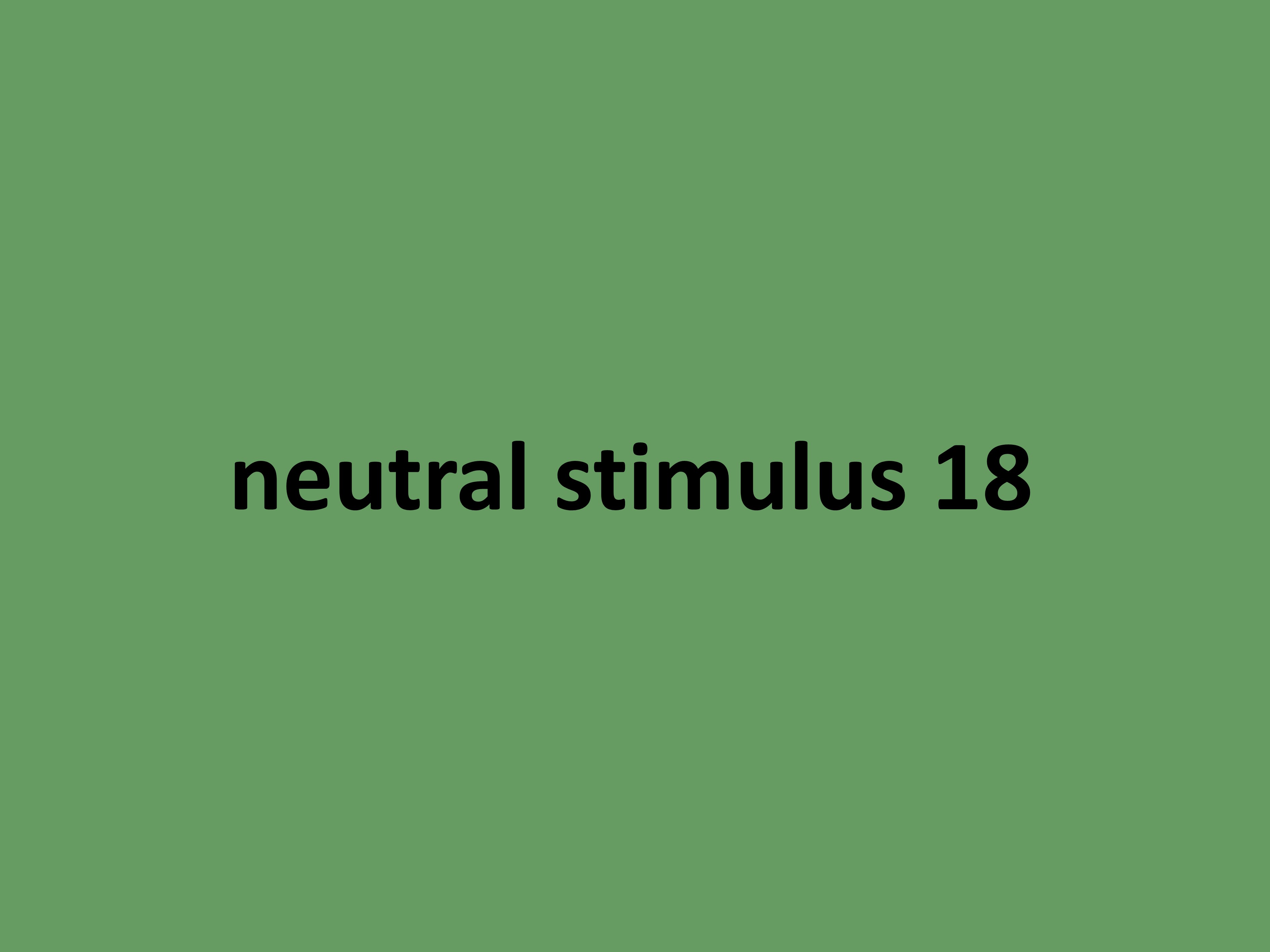

Supplement: S2 File — (ZIP) [file pone.0257717.s002.zip › software/stimuli/stimulus_neutral_18.jpg]

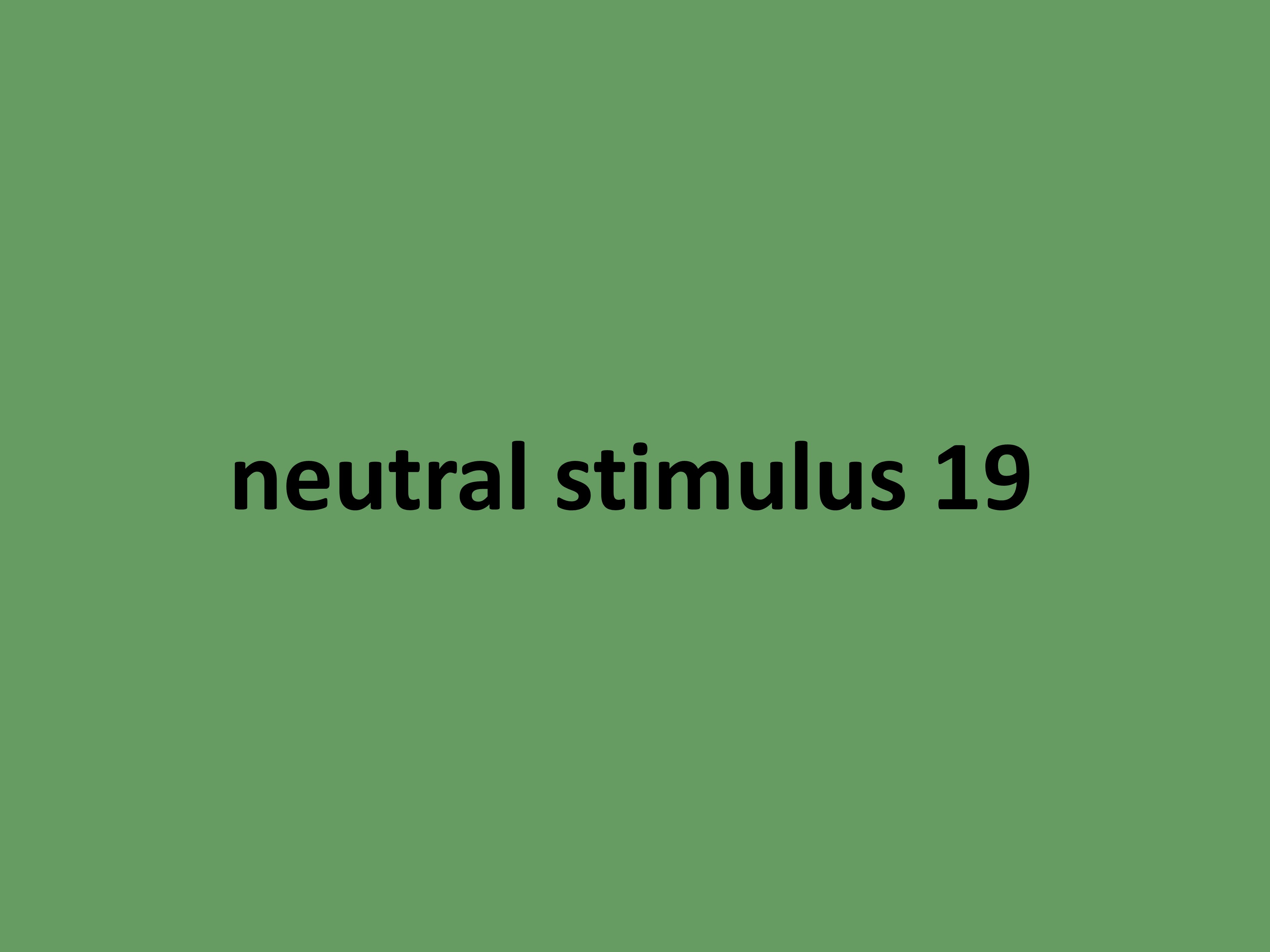

Supplement: S2 File — (ZIP) [file pone.0257717.s002.zip › software/stimuli/stimulus_neutral_19.jpg]

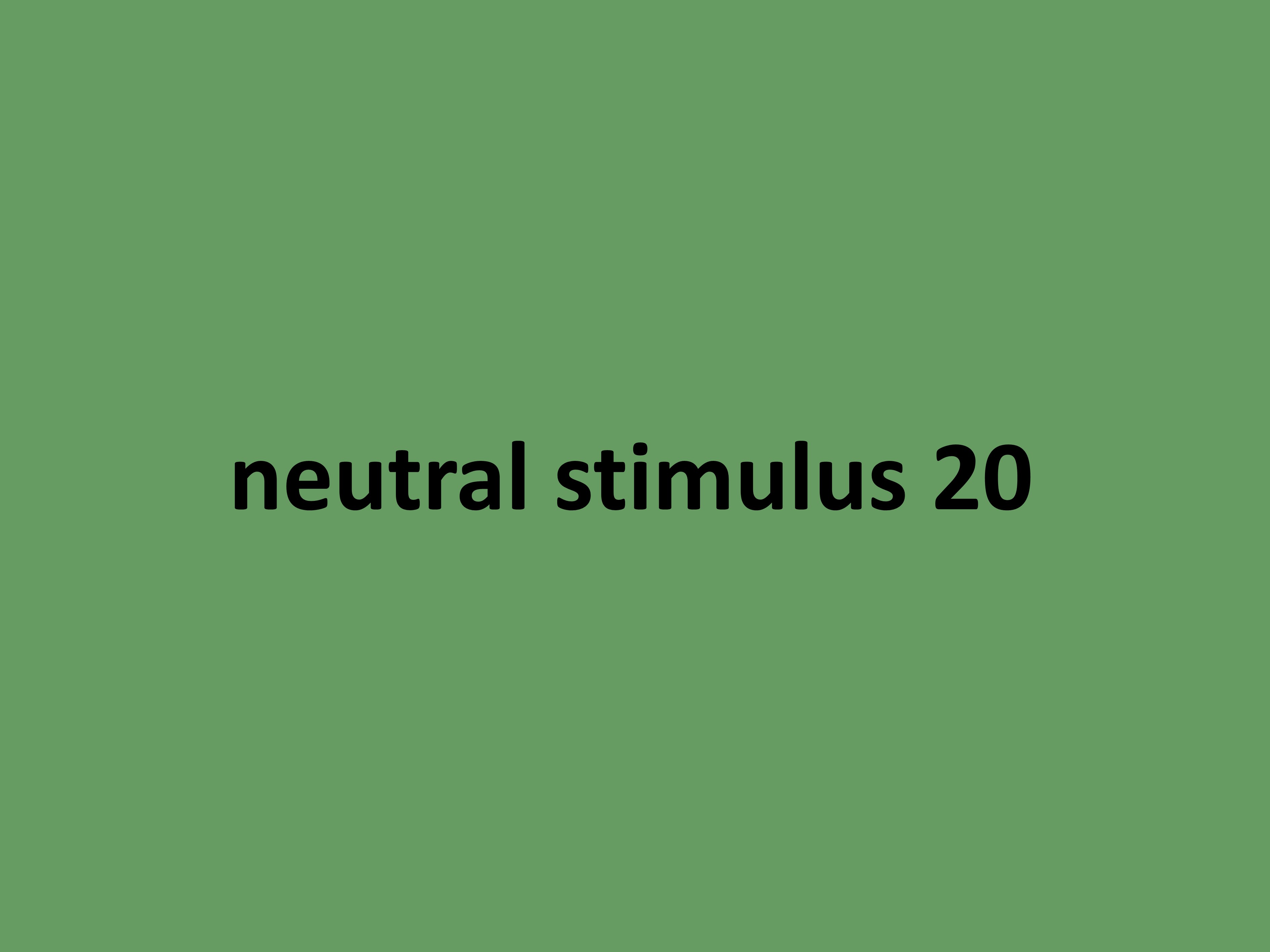

Supplement: S2 File — (ZIP) [file pone.0257717.s002.zip › software/stimuli/stimulus_neutral_20.jpg]

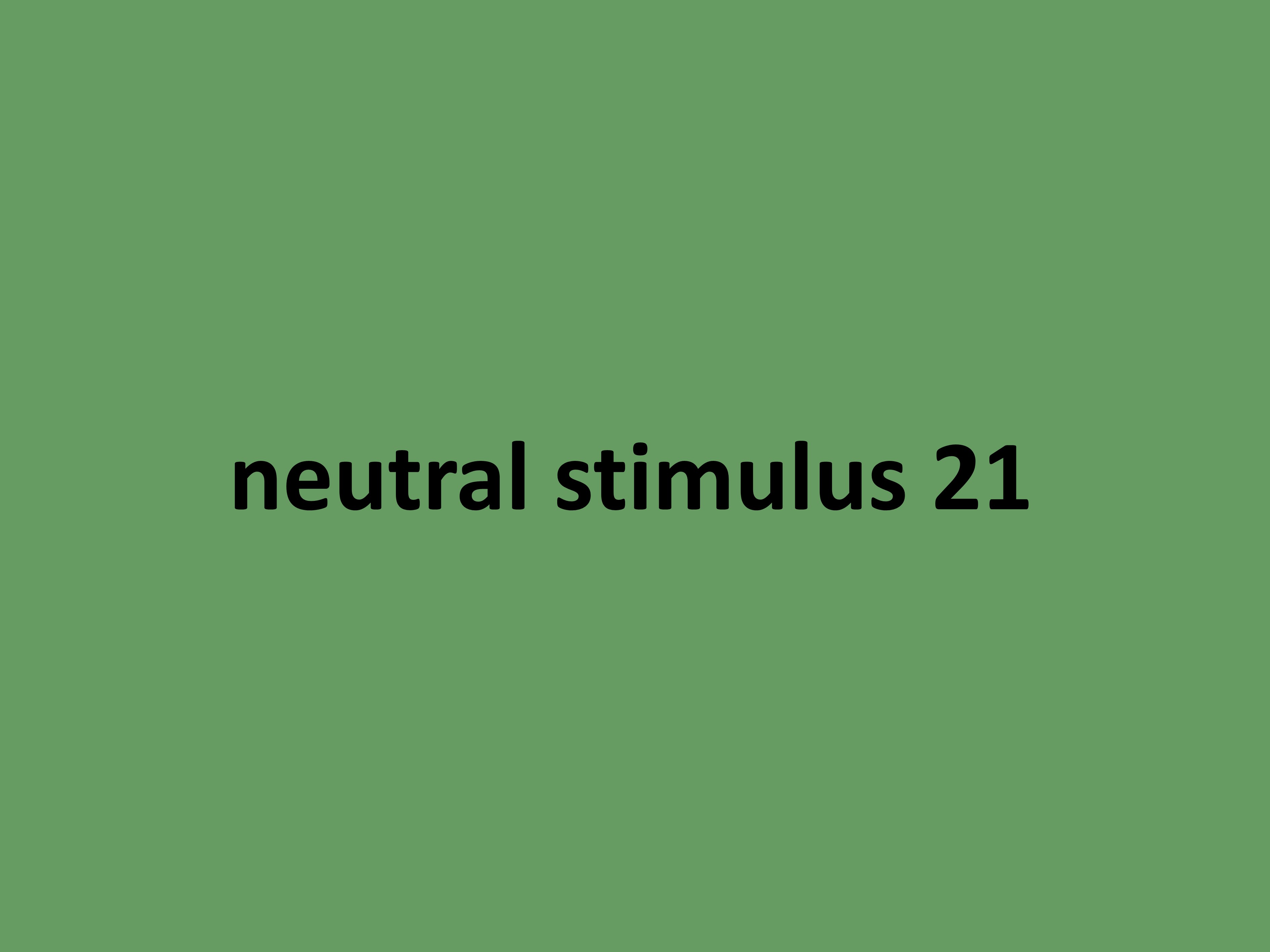

Supplement: S2 File — (ZIP) [file pone.0257717.s002.zip › software/stimuli/stimulus_neutral_21.jpg]

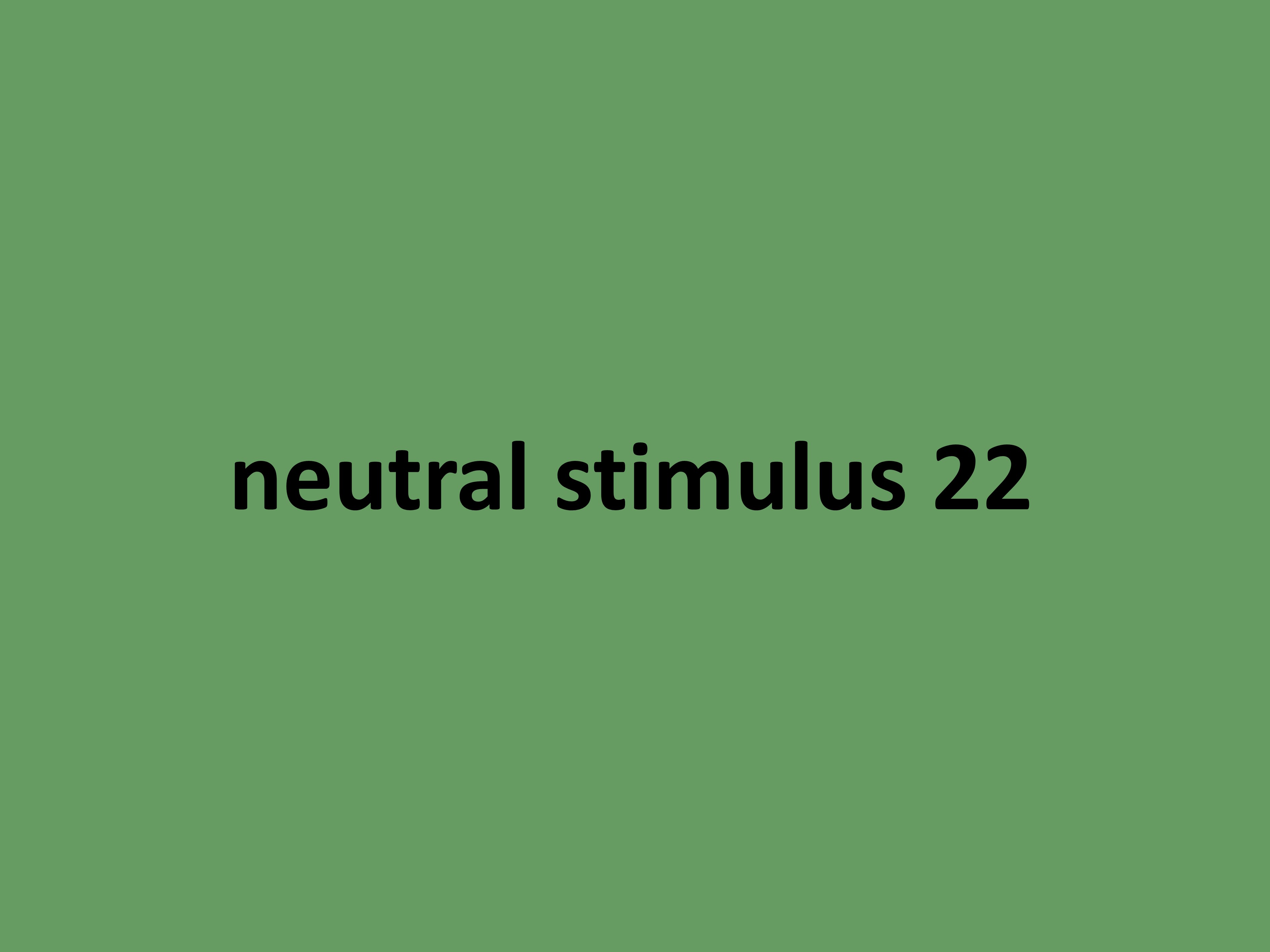

Supplement: S2 File — (ZIP) [file pone.0257717.s002.zip › software/stimuli/stimulus_neutral_22.jpg]

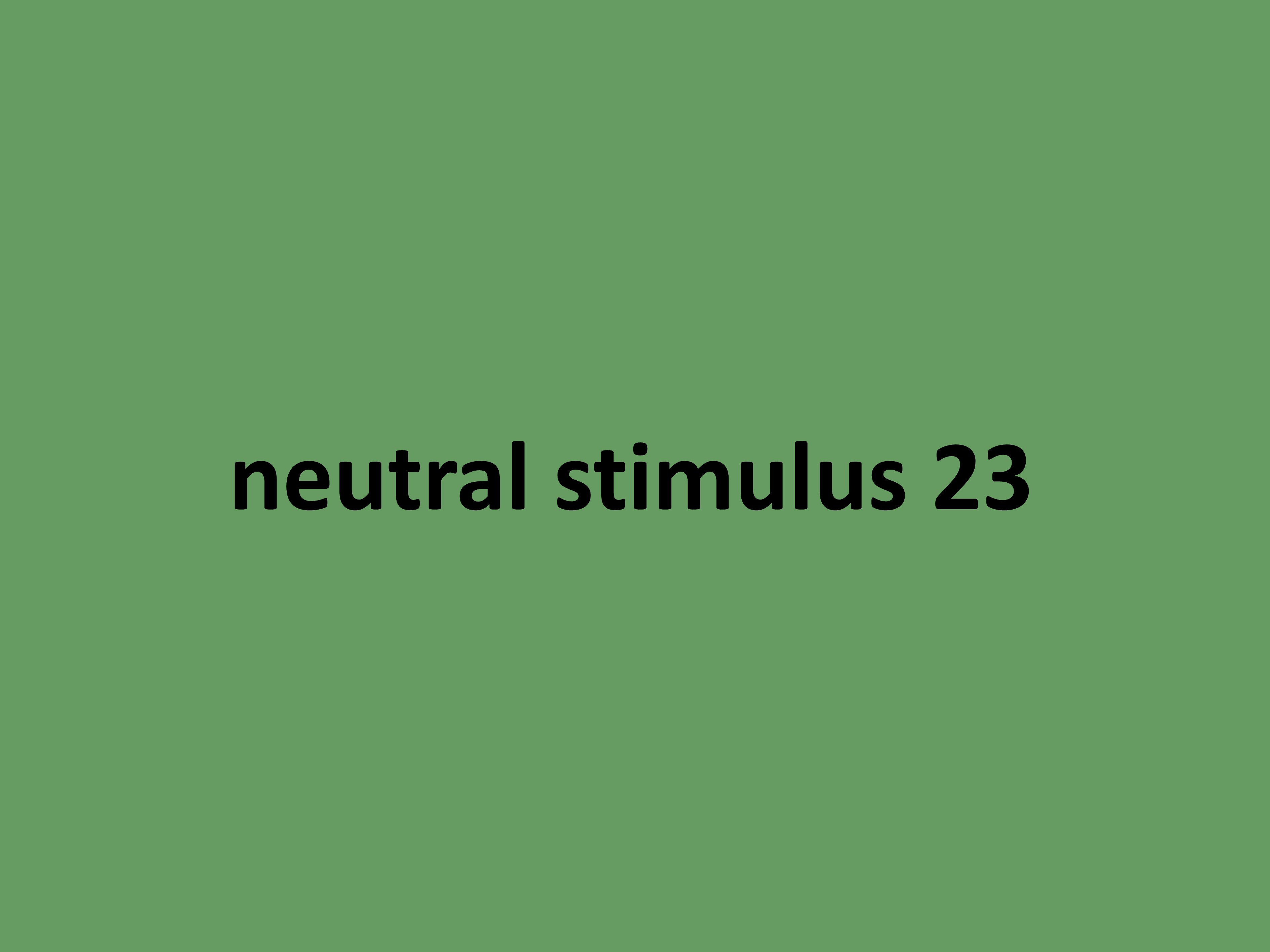

Supplement: S2 File — (ZIP) [file pone.0257717.s002.zip › software/stimuli/stimulus_neutral_23.jpg]

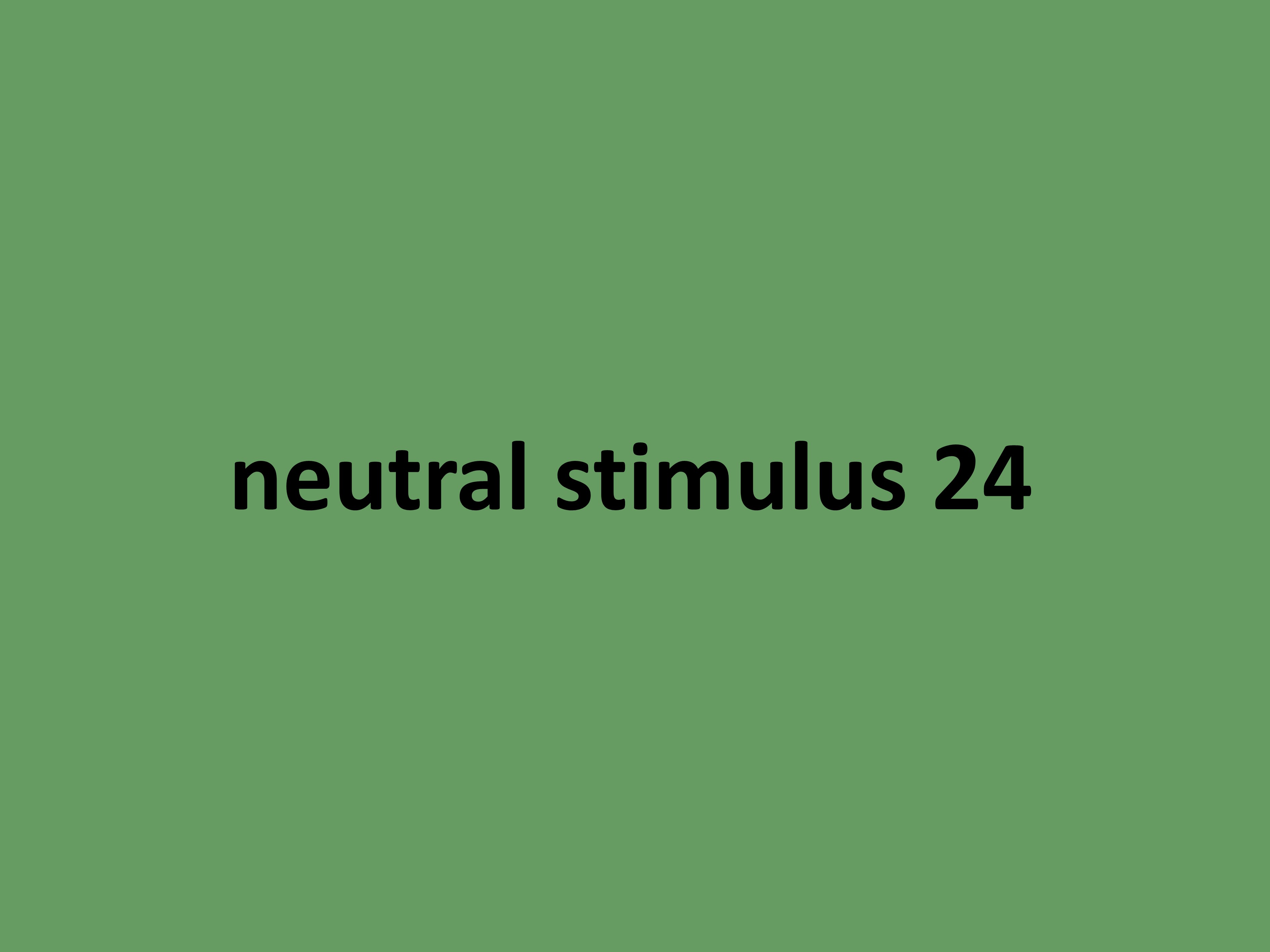

Supplement: S2 File — (ZIP) [file pone.0257717.s002.zip › software/stimuli/stimulus_neutral_24.jpg]

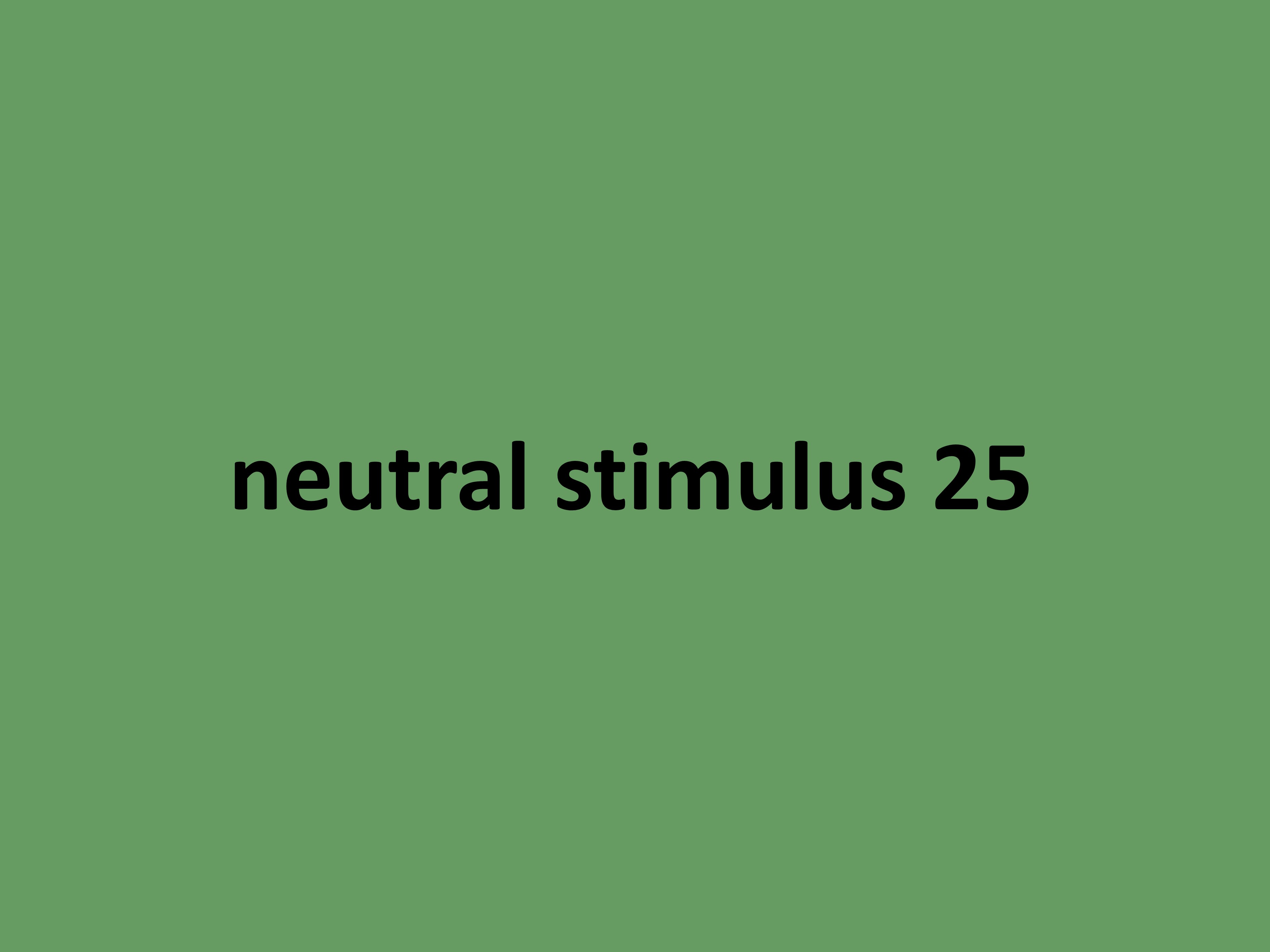

Supplement: S2 File — (ZIP) [file pone.0257717.s002.zip › software/stimuli/stimulus_neutral_25.jpg]

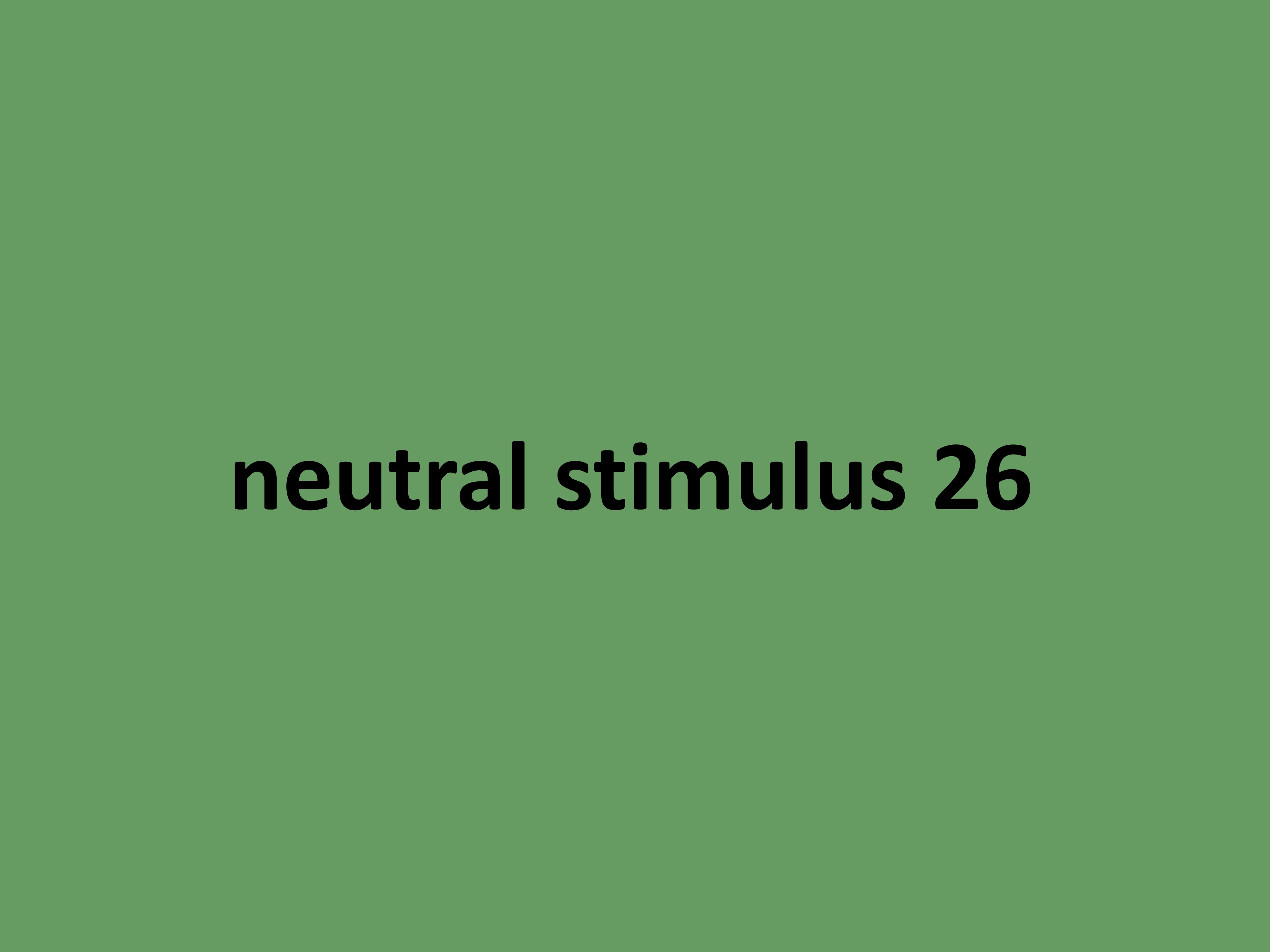

Supplement: S2 File — (ZIP) [file pone.0257717.s002.zip › software/stimuli/stimulus_neutral_26.jpg]

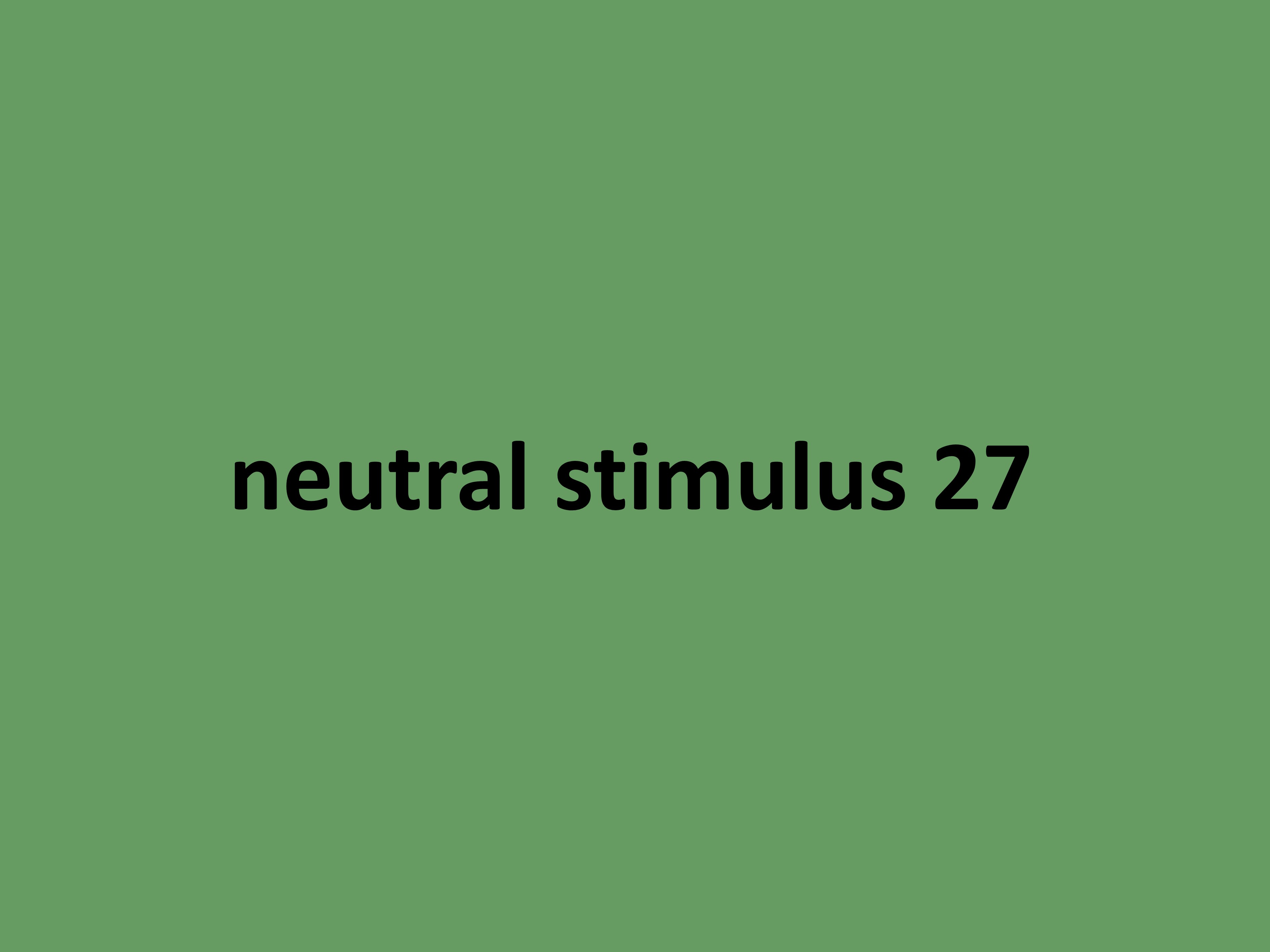

Supplement: S2 File — (ZIP) [file pone.0257717.s002.zip › software/stimuli/stimulus_neutral_27.jpg]

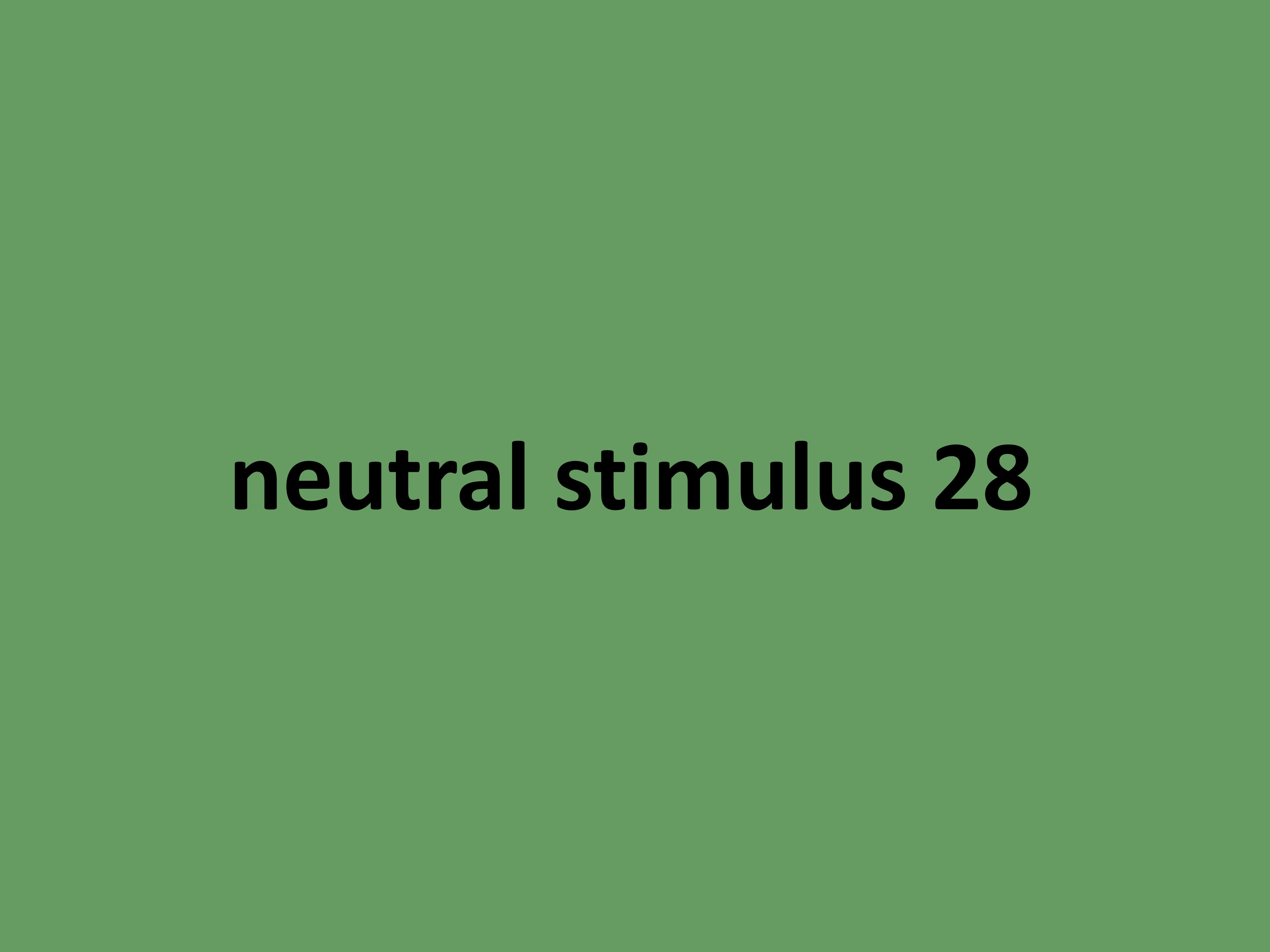

Supplement: S2 File — (ZIP) [file pone.0257717.s002.zip › software/stimuli/stimulus_neutral_28.jpg]

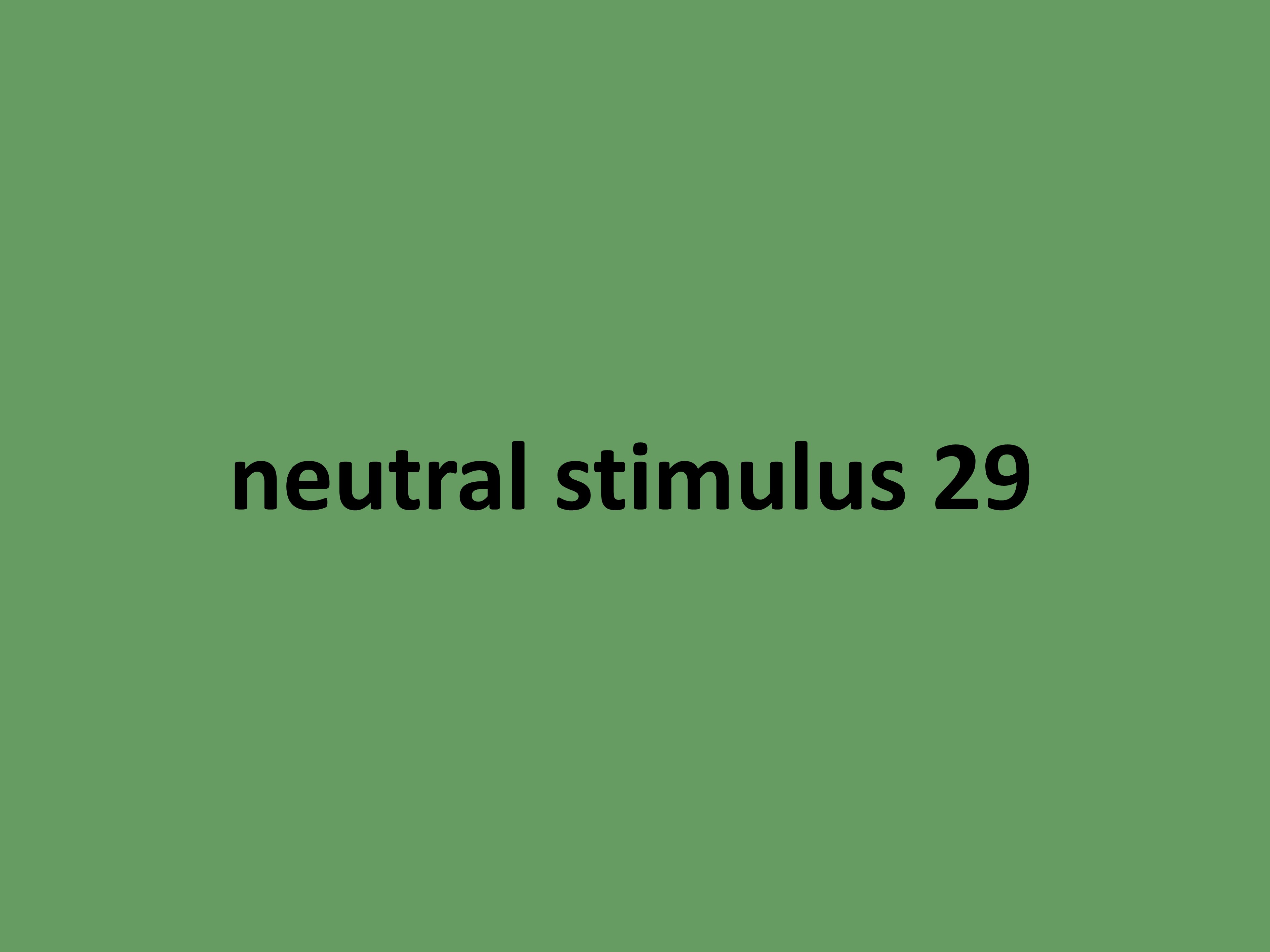

Supplement: S2 File — (ZIP) [file pone.0257717.s002.zip › software/stimuli/stimulus_neutral_29.jpg]

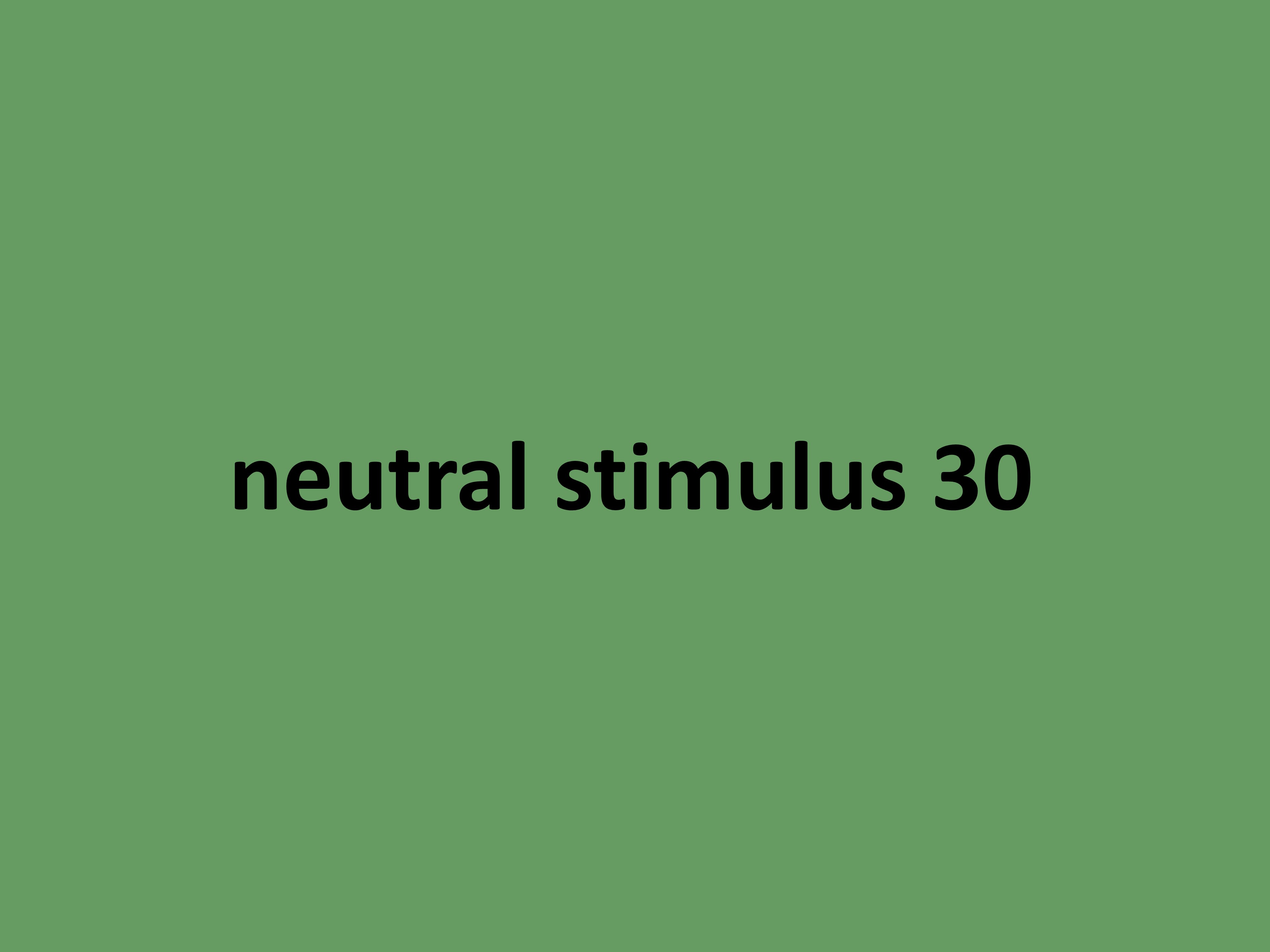

Supplement: S2 File — (ZIP) [file pone.0257717.s002.zip › software/stimuli/stimulus_neutral_30.jpg]

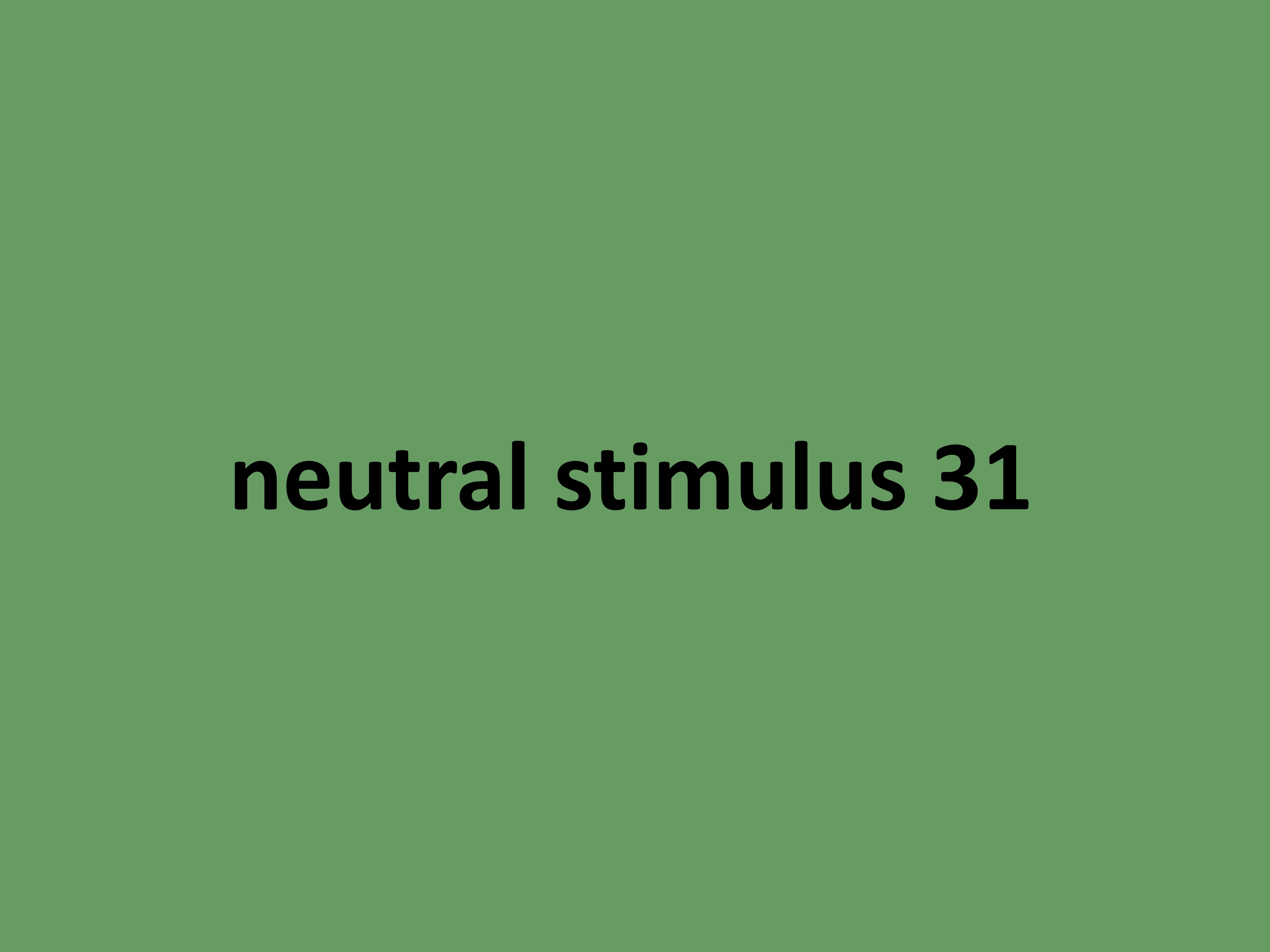

Supplement: S2 File — (ZIP) [file pone.0257717.s002.zip › software/stimuli/stimulus_neutral_31.jpg]

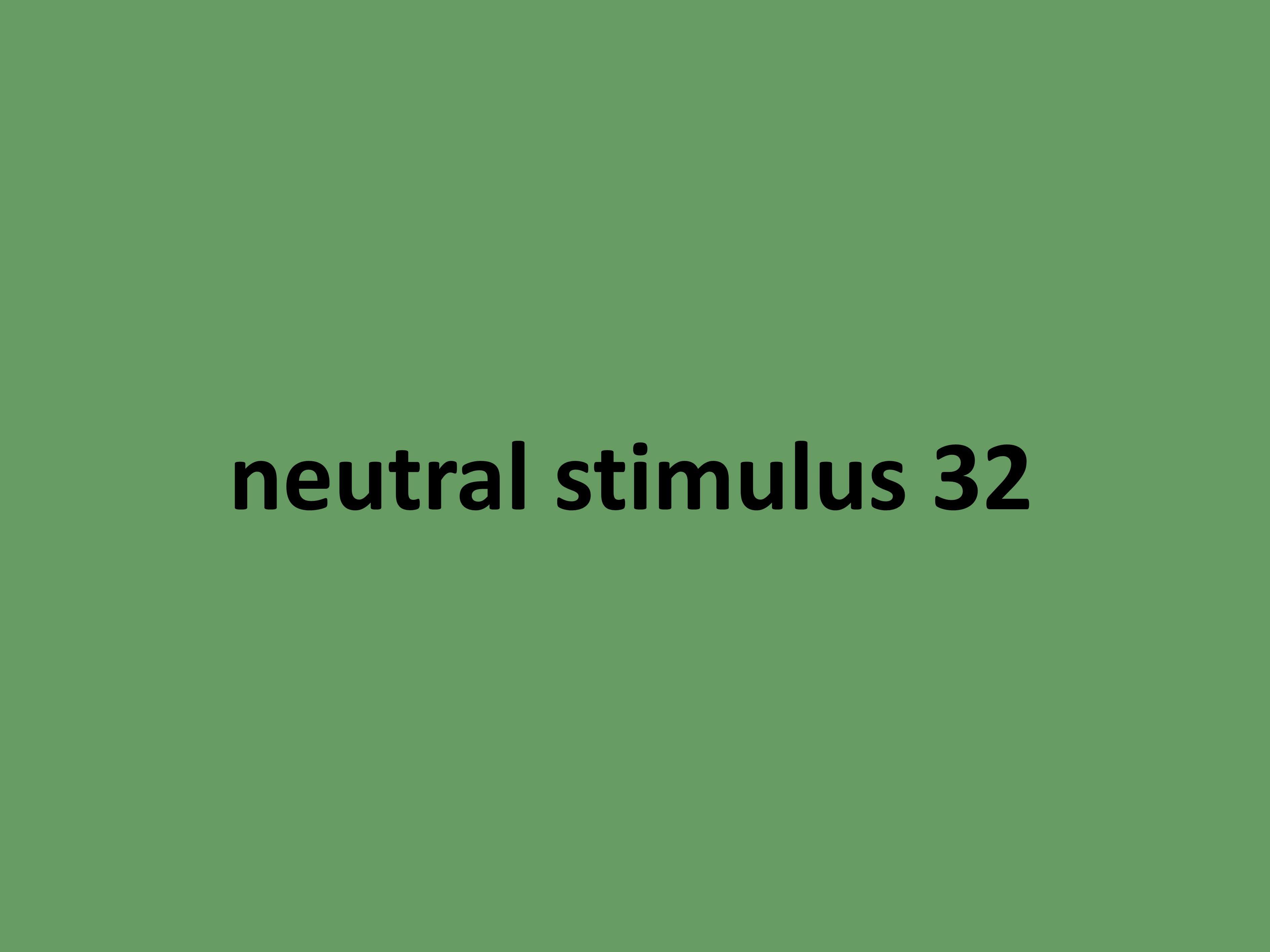

Supplement: S2 File — (ZIP) [file pone.0257717.s002.zip › software/stimuli/stimulus_neutral_32.jpg]

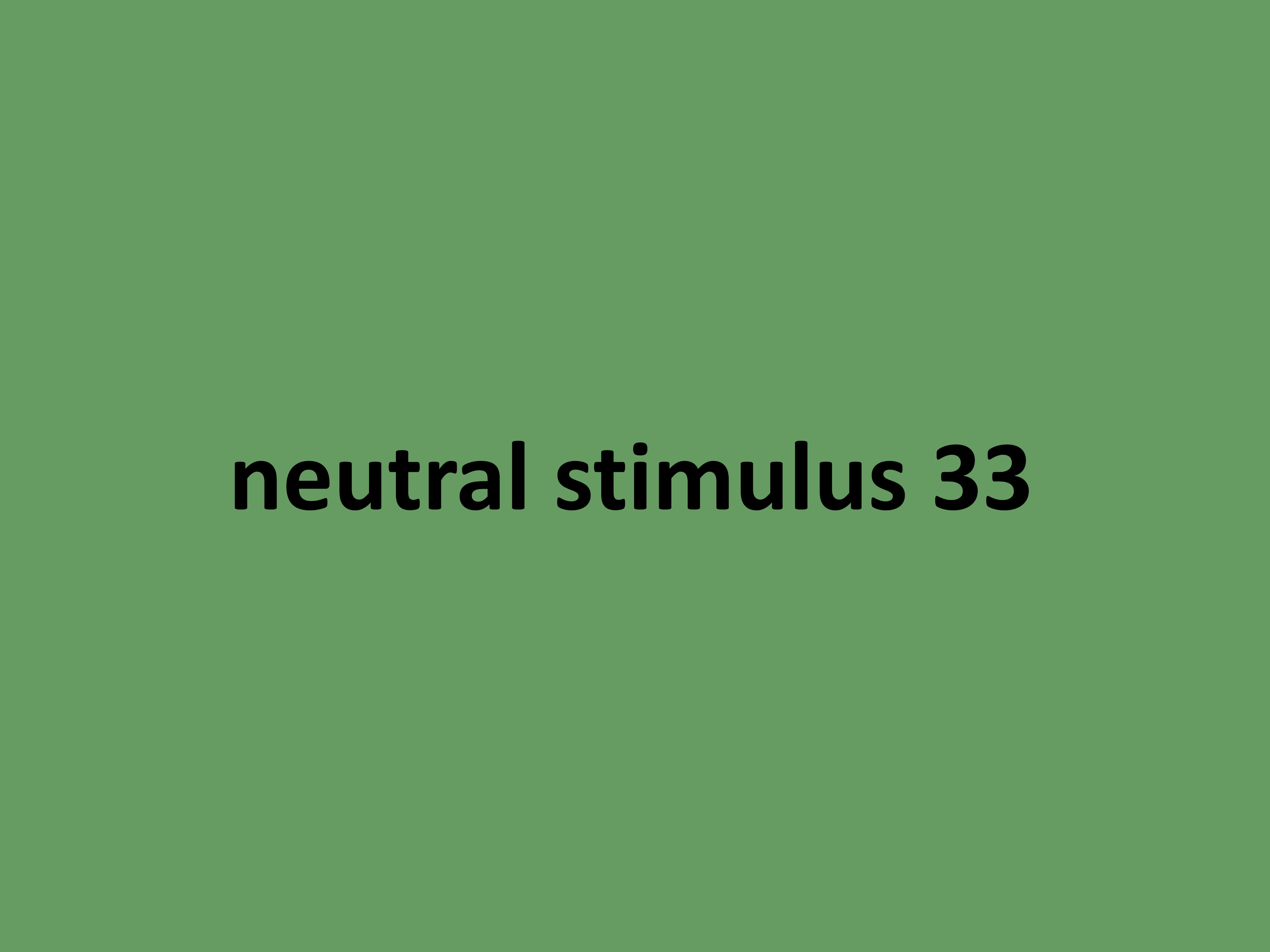

Supplement: S2 File — (ZIP) [file pone.0257717.s002.zip › software/stimuli/stimulus_neutral_33.jpg]

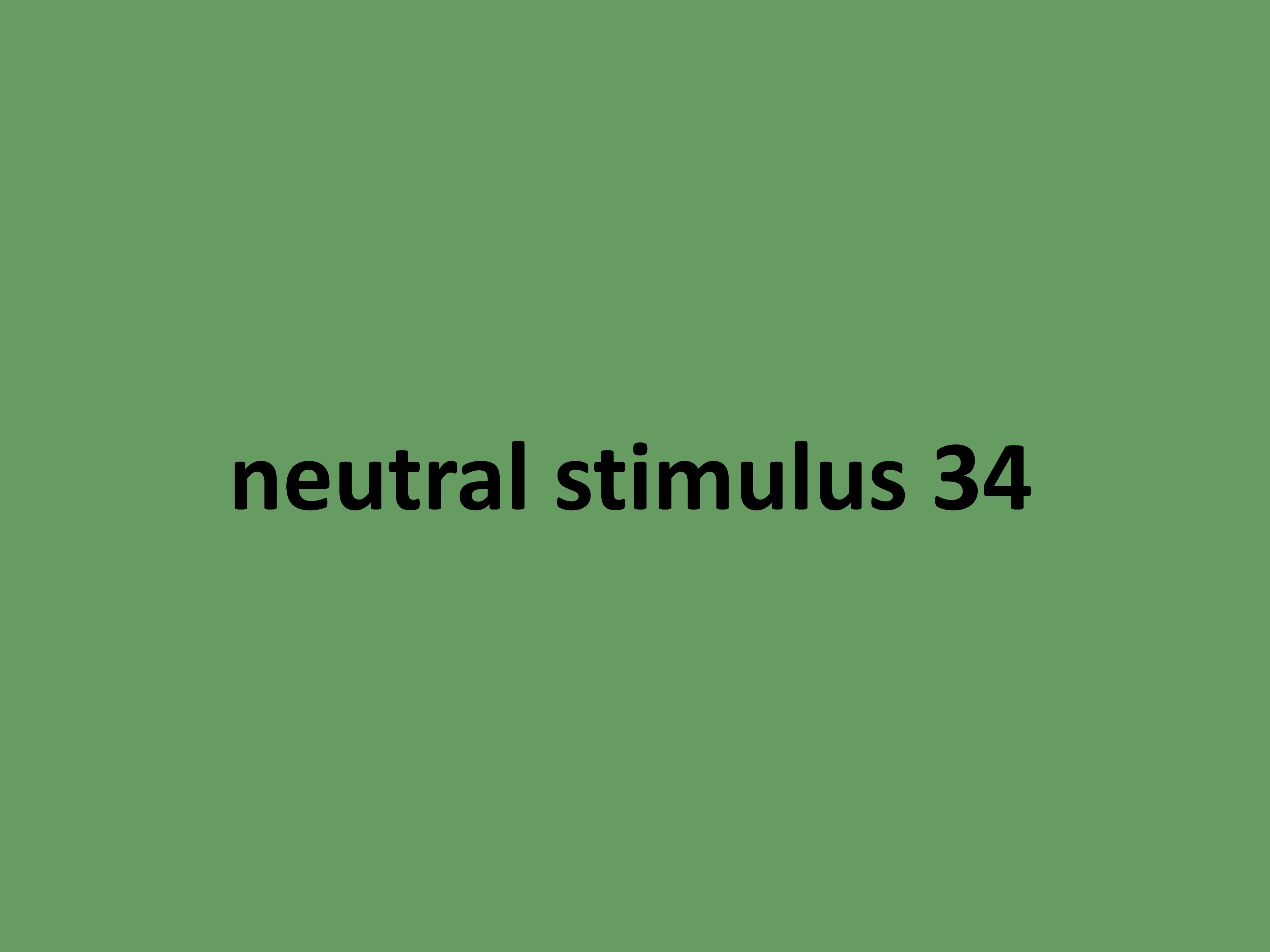

Supplement: S2 File — (ZIP) [file pone.0257717.s002.zip › software/stimuli/stimulus_neutral_34.jpg]

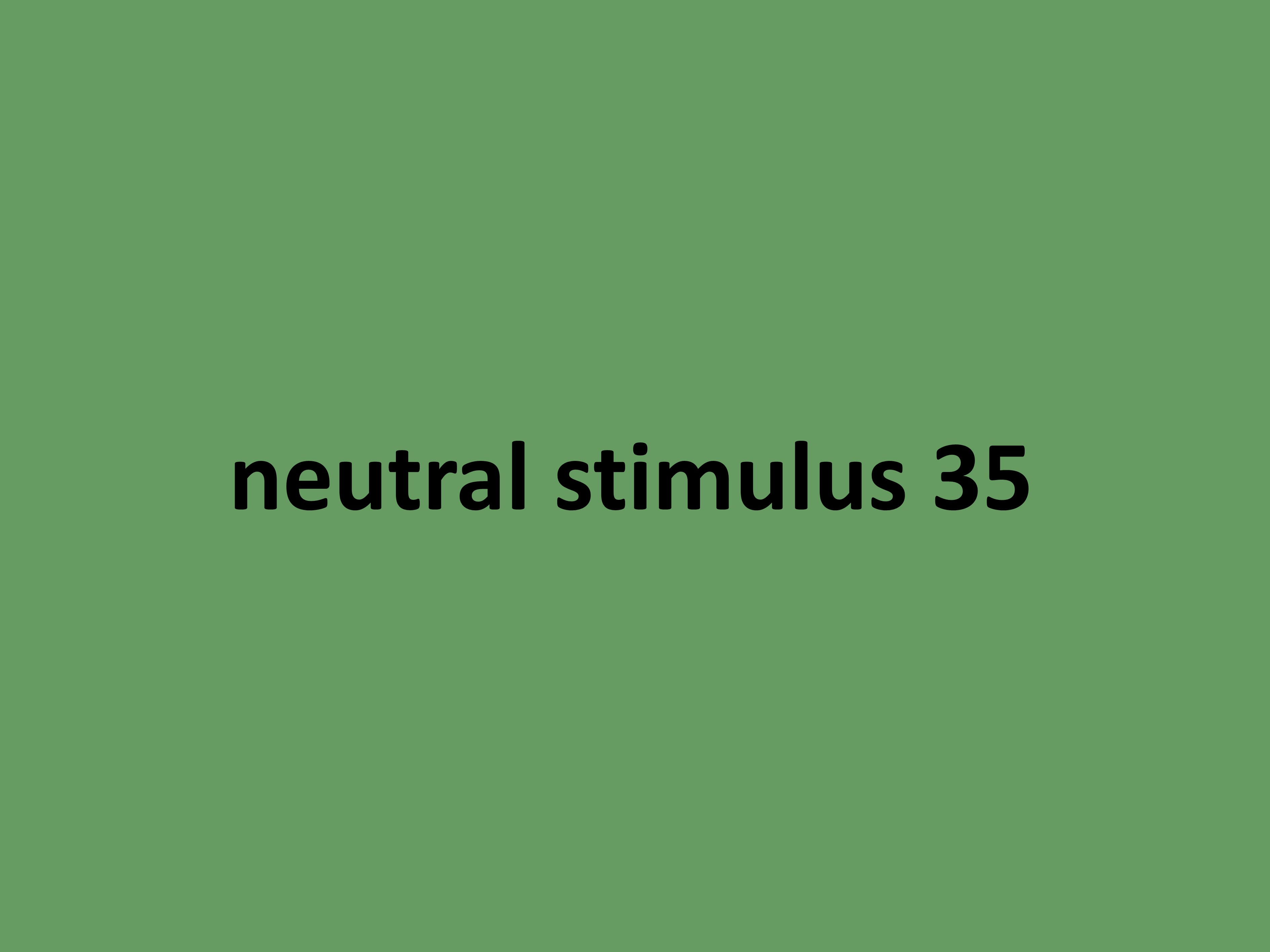

Supplement: S2 File — (ZIP) [file pone.0257717.s002.zip › software/stimuli/stimulus_neutral_35.jpg]

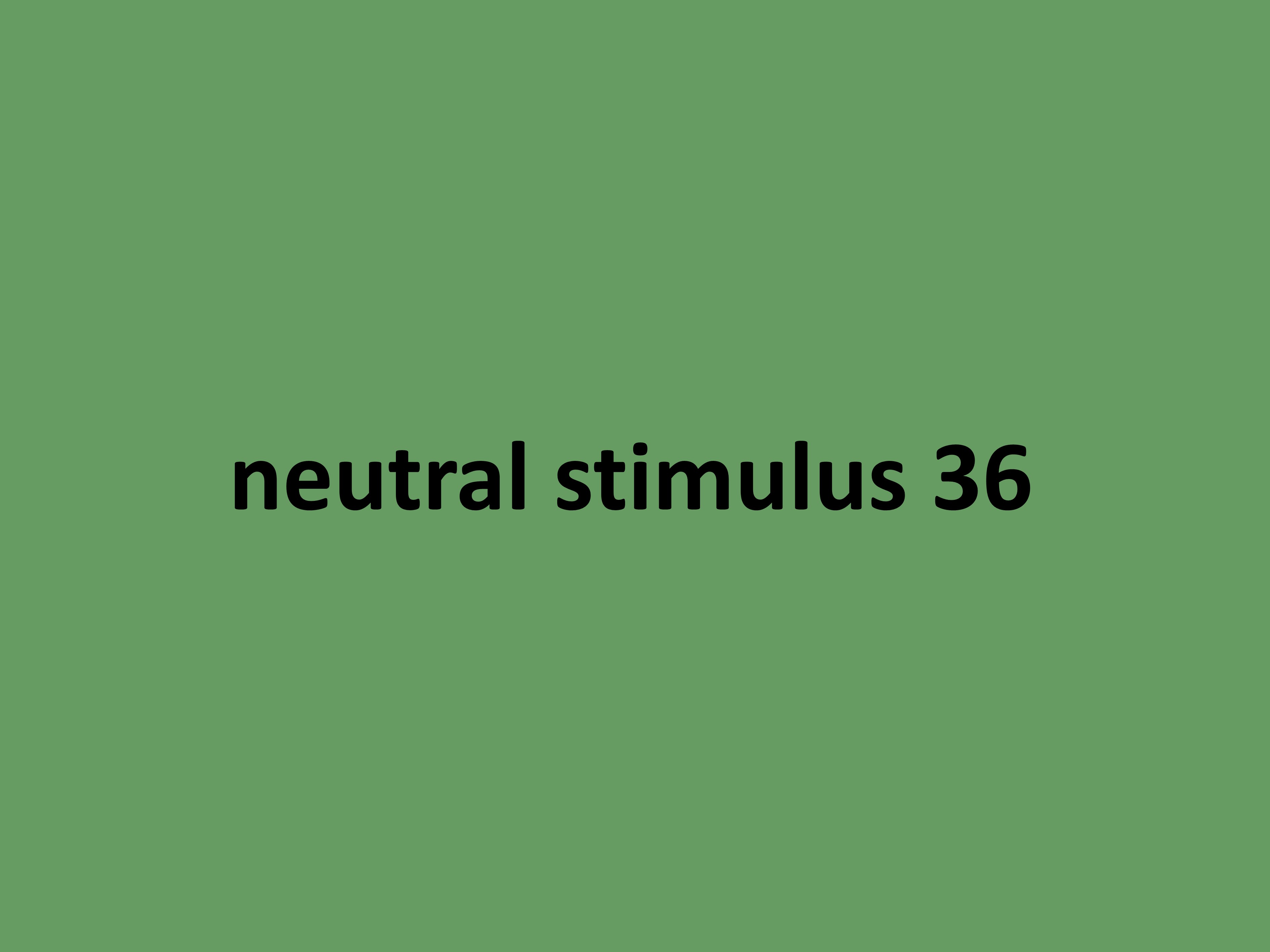

Supplement: S2 File — (ZIP) [file pone.0257717.s002.zip › software/stimuli/stimulus_neutral_36.jpg]

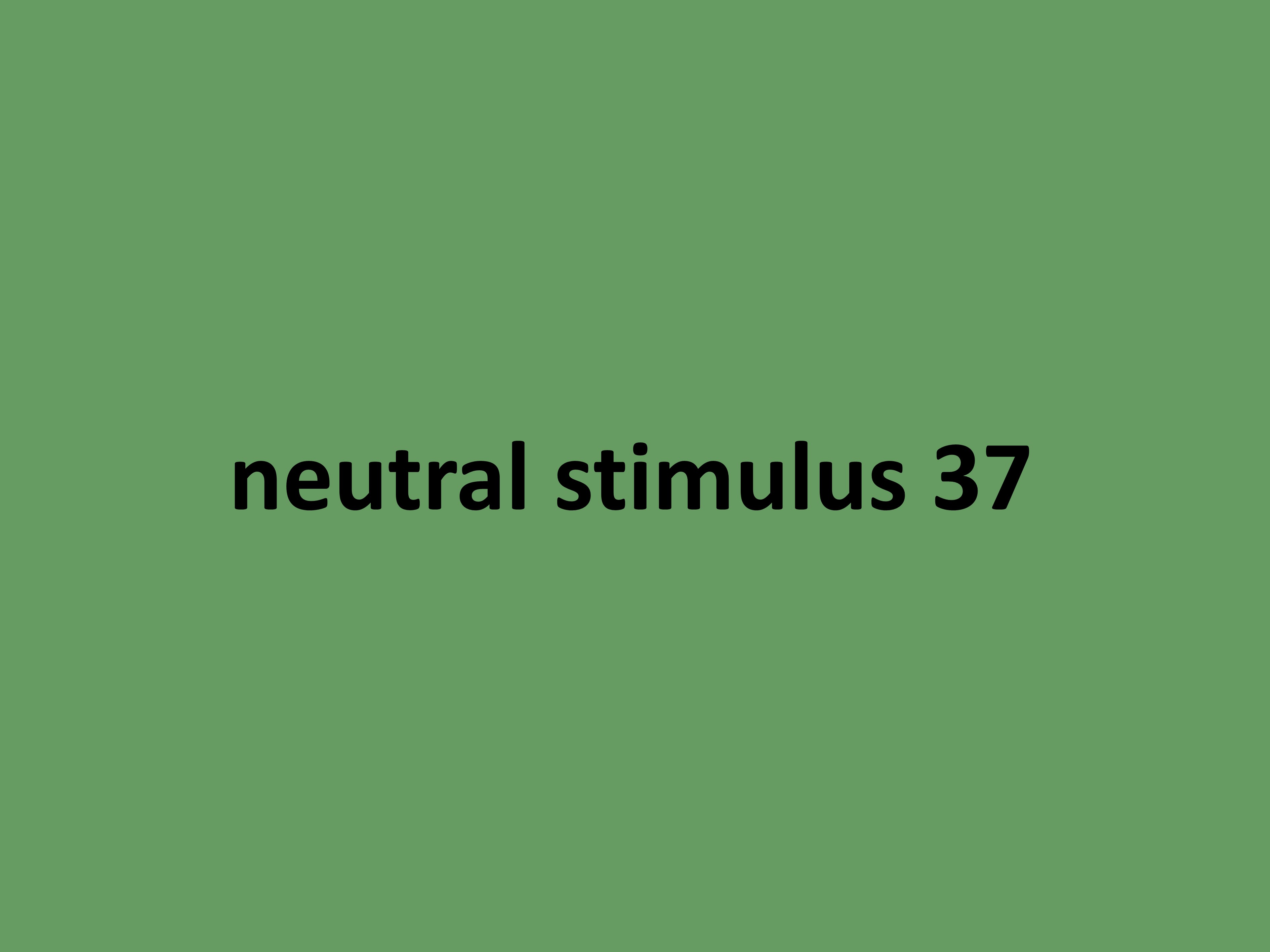

Supplement: S2 File — (ZIP) [file pone.0257717.s002.zip › software/stimuli/stimulus_neutral_37.jpg]

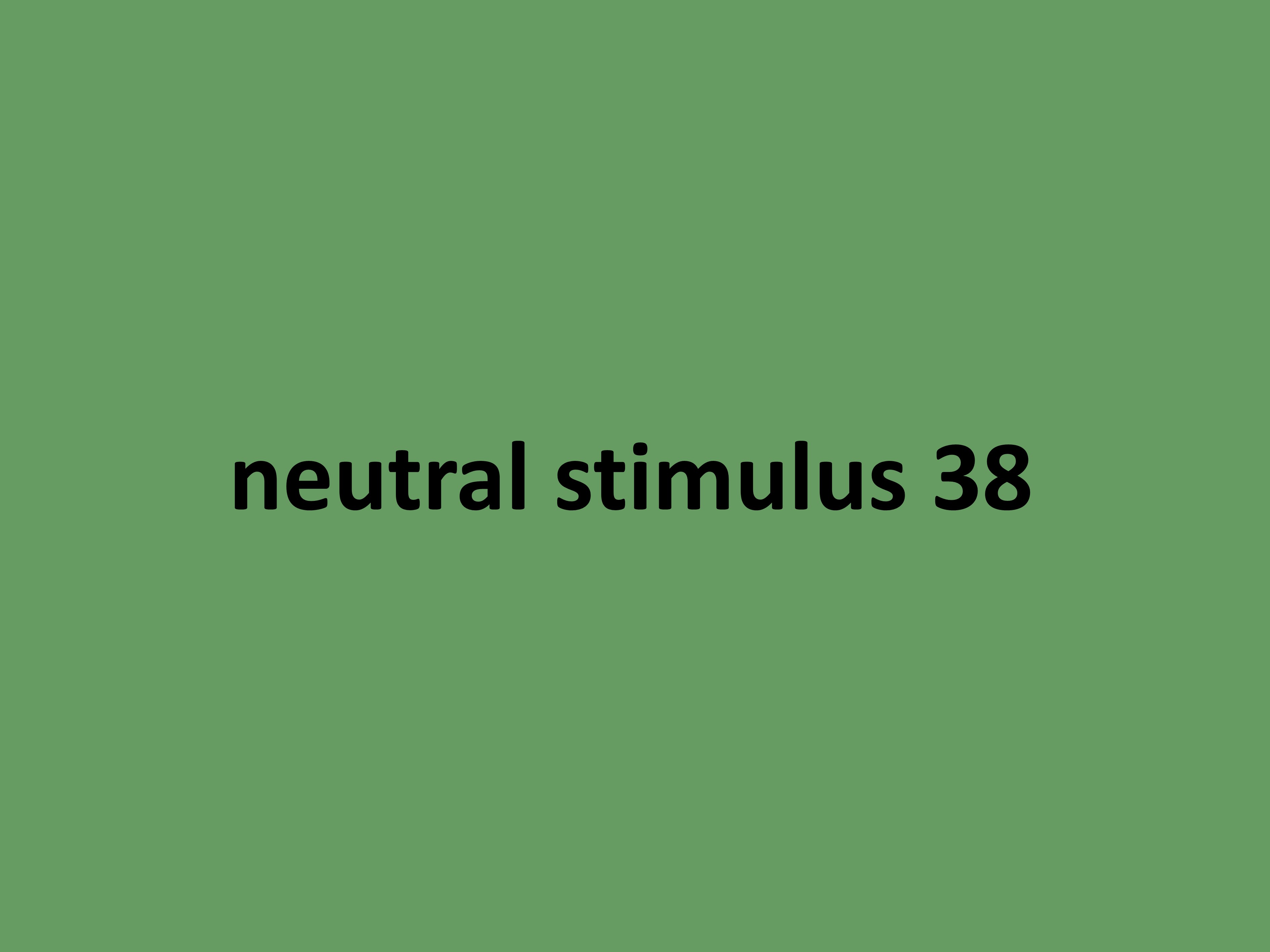

Supplement: S2 File — (ZIP) [file pone.0257717.s002.zip › software/stimuli/stimulus_neutral_38.jpg]

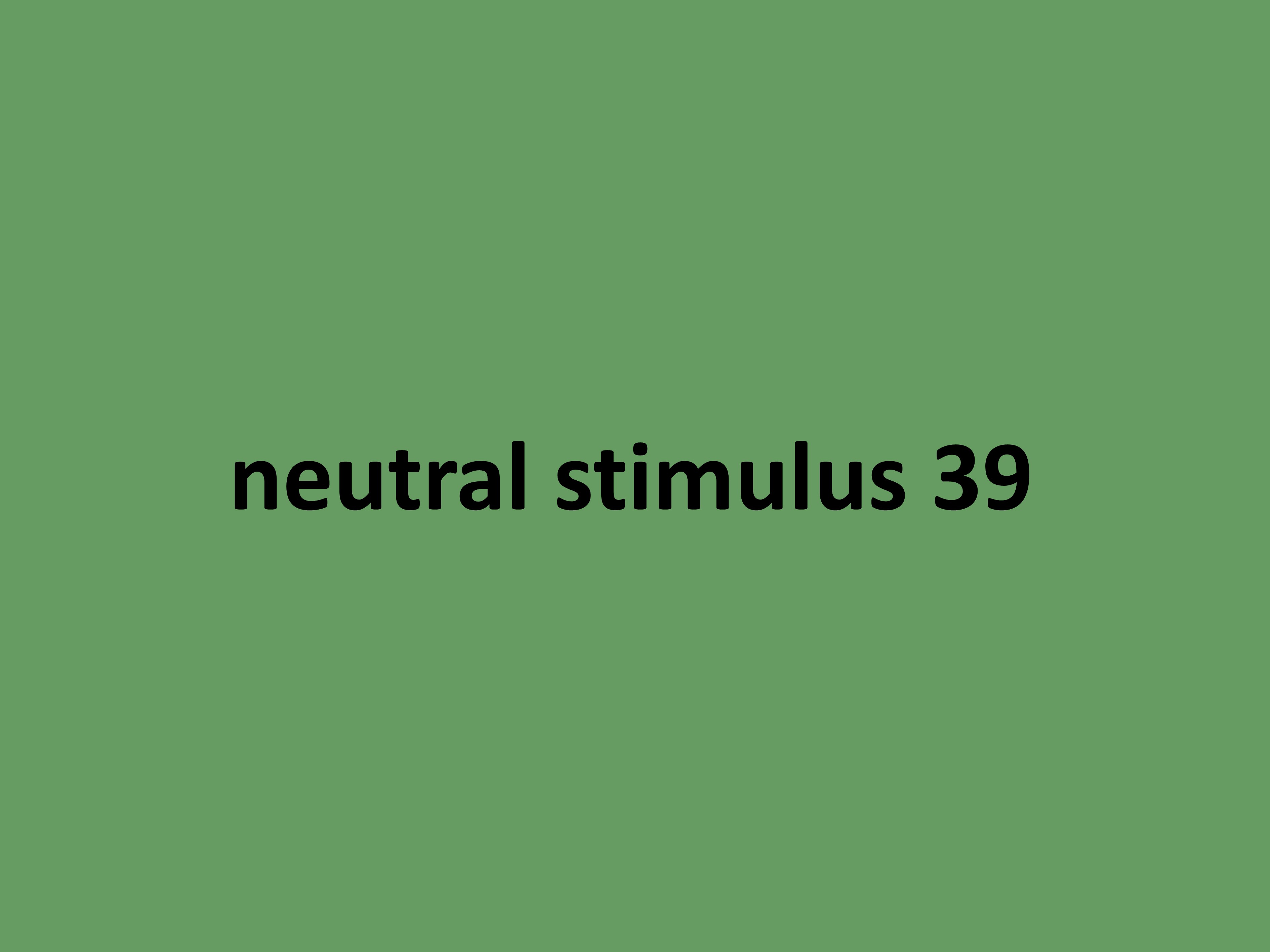

Supplement: S2 File — (ZIP) [file pone.0257717.s002.zip › software/stimuli/stimulus_neutral_39.jpg]

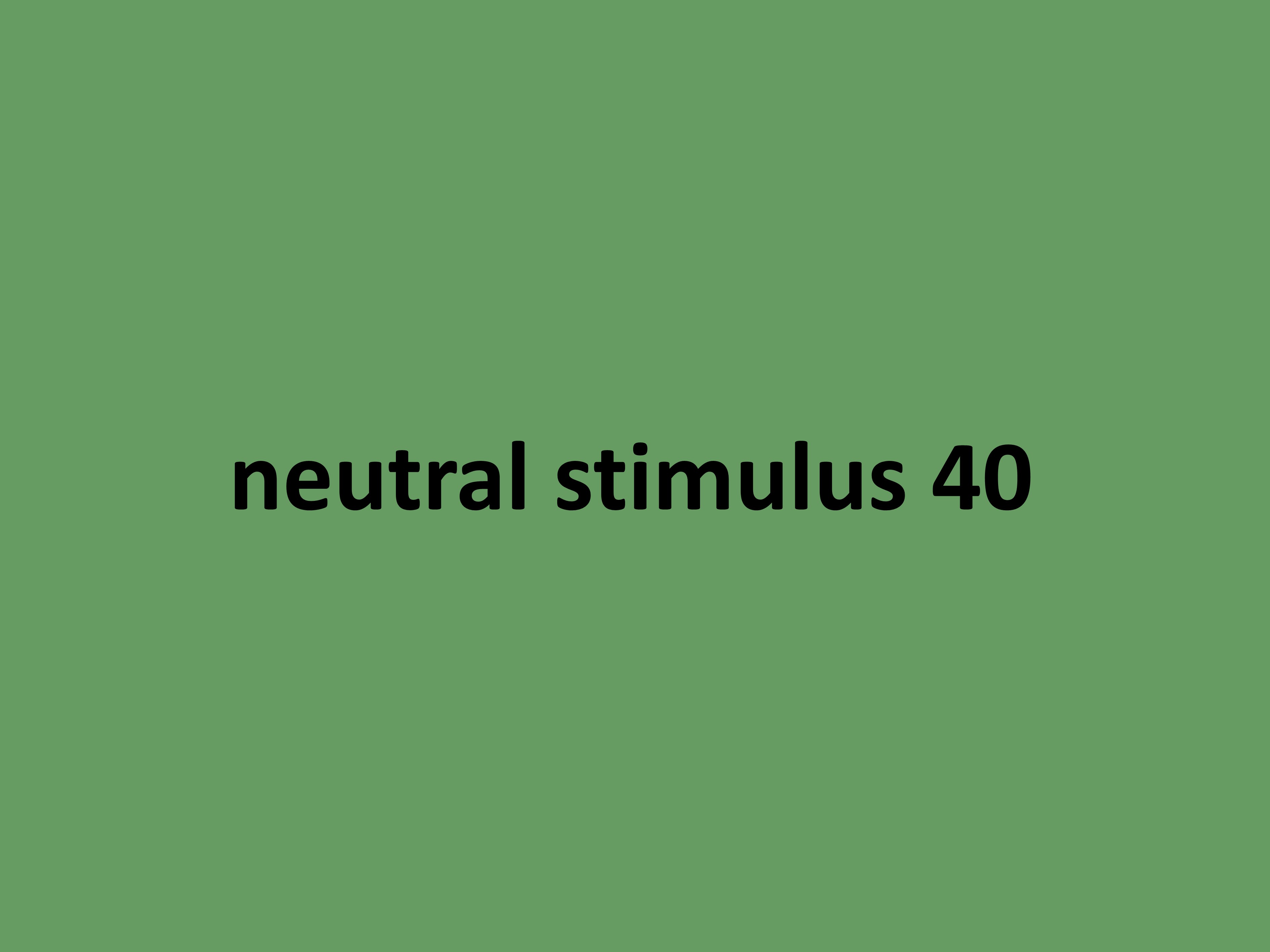

Supplement: S2 File — (ZIP) [file pone.0257717.s002.zip › software/stimuli/stimulus_neutral_40.jpg]

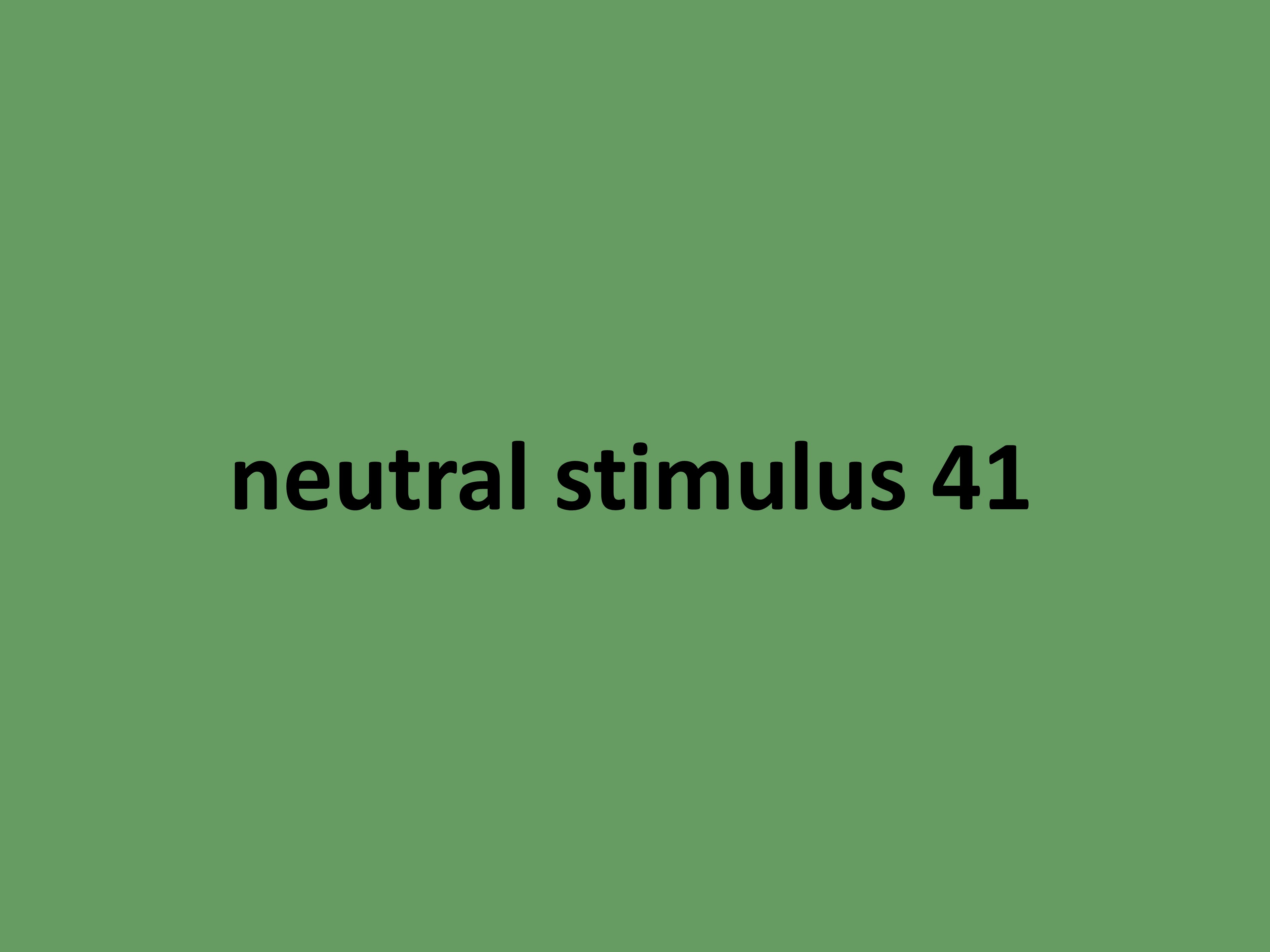

Supplement: S2 File — (ZIP) [file pone.0257717.s002.zip › software/stimuli/stimulus_neutral_41.jpg]
