# Supplementary figures and images for: Detours increase local knowledge—Exploring the hidden benefits of self-control failure (part 1 of 2)
Source: PLoS One. 2021 Oct 1;16(10):e0257717. doi: 10.1371/journal.pone.0257717 (PMC8486128; doi:10.1371/journal.pone.0257717)

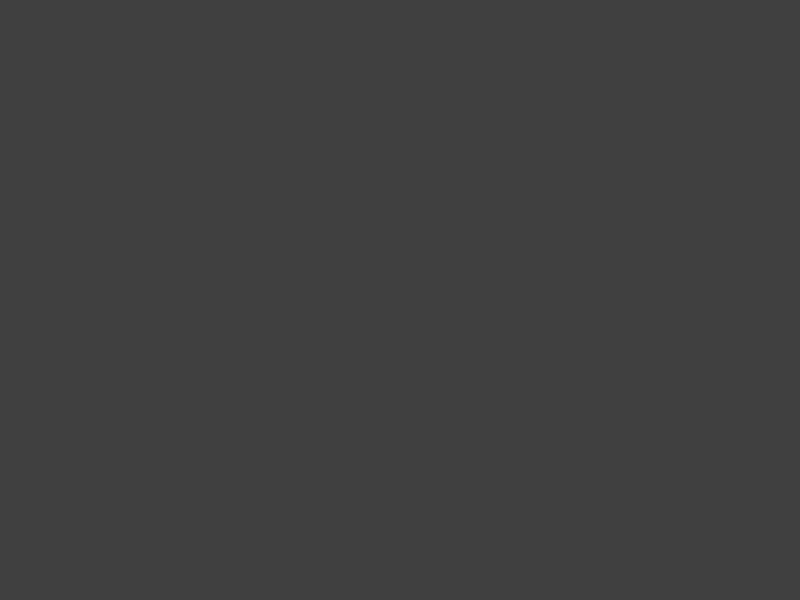

Supplement: S2 File — (ZIP) [file pone.0257717.s002.zip › software/stimuli/blank.jpg]

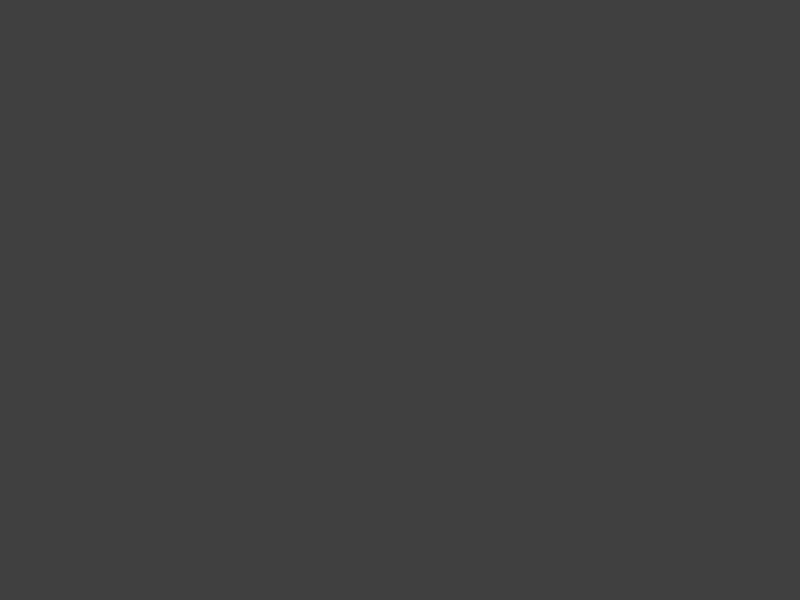

Supplement: S2 File — (ZIP) [file pone.0257717.s002.zip › software/stimuli/Blank.tif]

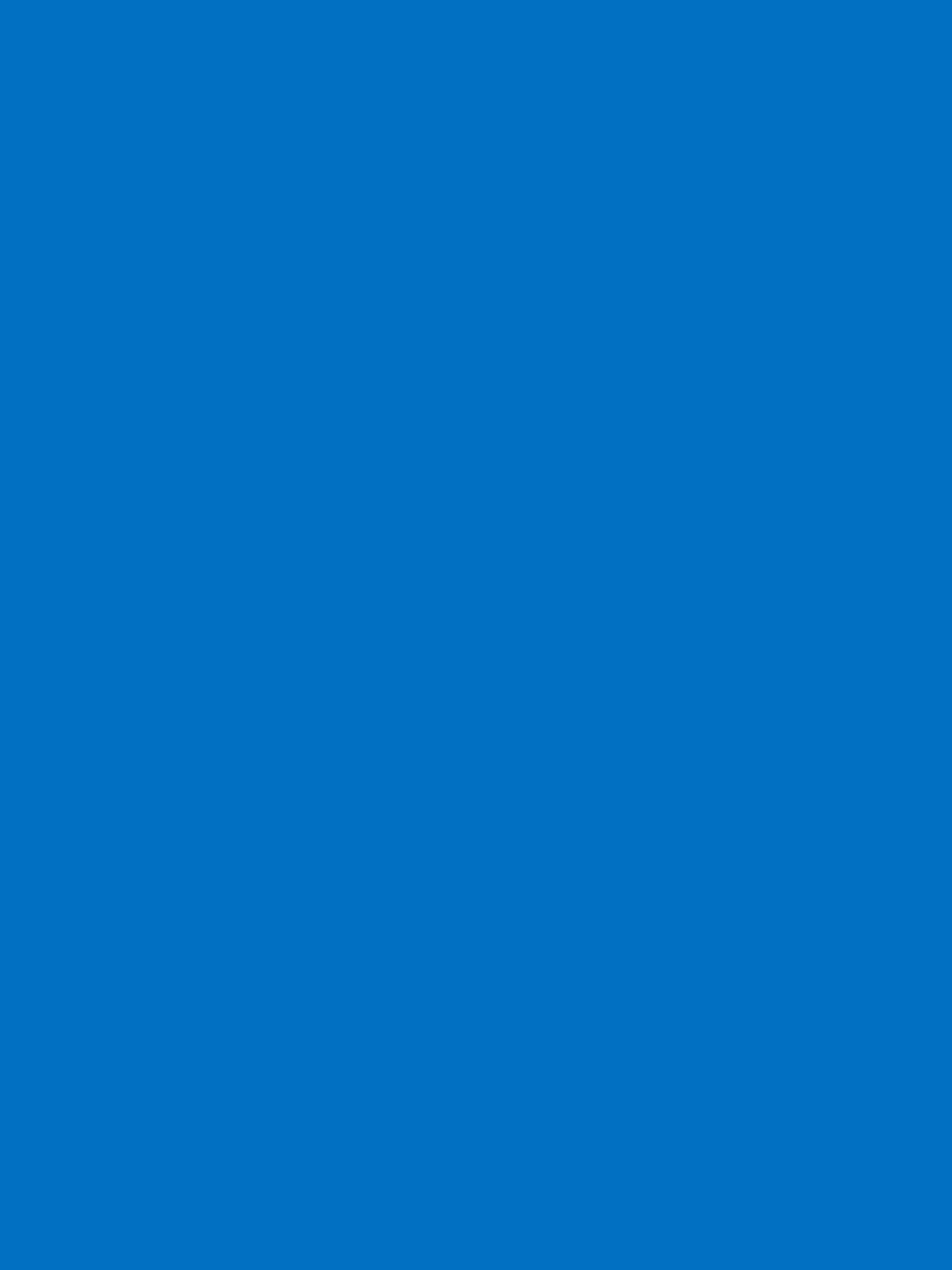

Supplement: S2 File — (ZIP) [file pone.0257717.s002.zip › software/stimuli/Blue.tif]

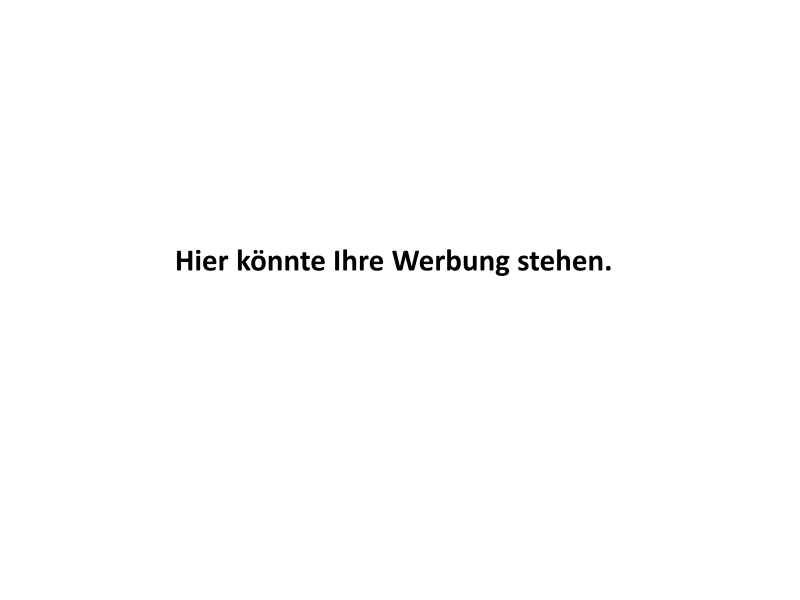

Supplement: S2 File — (ZIP) [file pone.0257717.s002.zip › software/stimuli/Default1.tif]

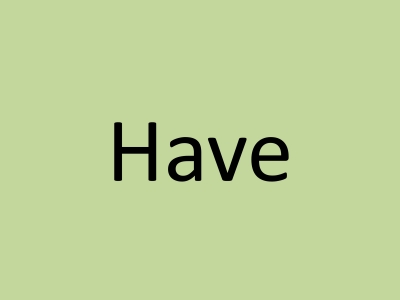

Supplement: S2 File — (ZIP) [file pone.0257717.s002.zip › software/stimuli/DefaultHave.jpg]

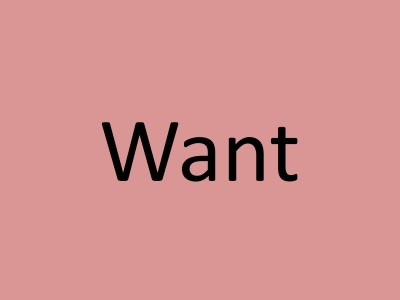

Supplement: S2 File — (ZIP) [file pone.0257717.s002.zip › software/stimuli/DefaultWant.jpg]

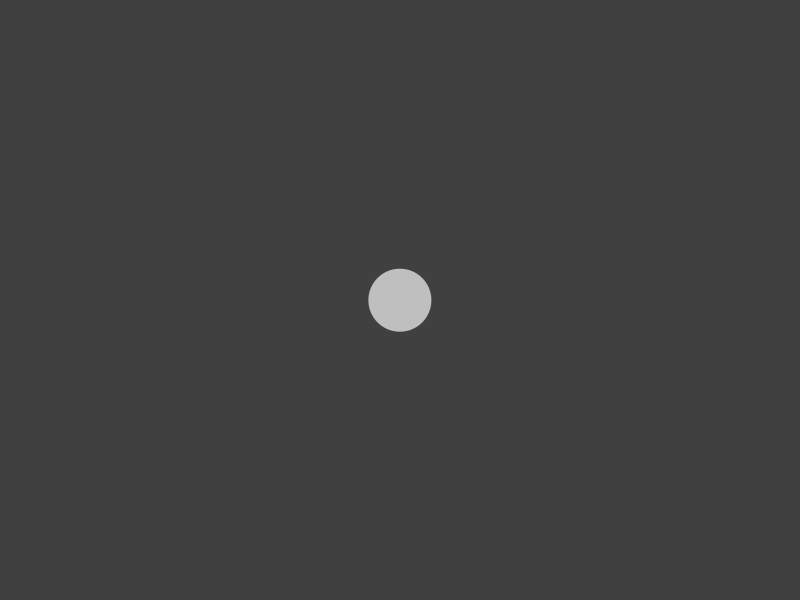

Supplement: S2 File — (ZIP) [file pone.0257717.s002.zip › software/stimuli/Fix.tif]

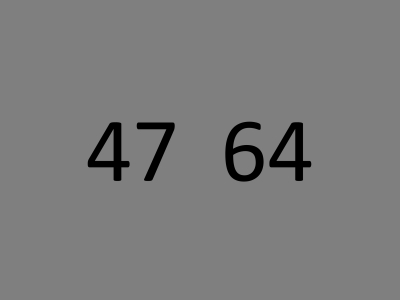

Supplement: S2 File — (ZIP) [file pone.0257717.s002.zip › software/stimuli/HaveToB1.jpg]

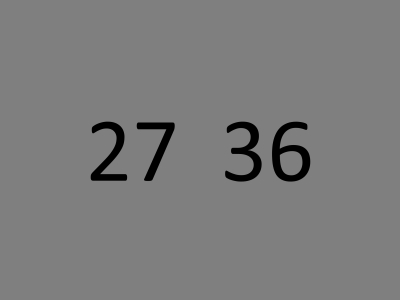

Supplement: S2 File — (ZIP) [file pone.0257717.s002.zip › software/stimuli/HaveToB2.jpg]

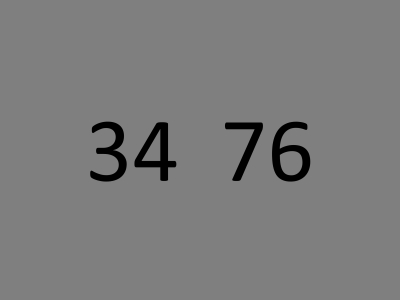

Supplement: S2 File — (ZIP) [file pone.0257717.s002.zip › software/stimuli/HaveToB3.jpg]

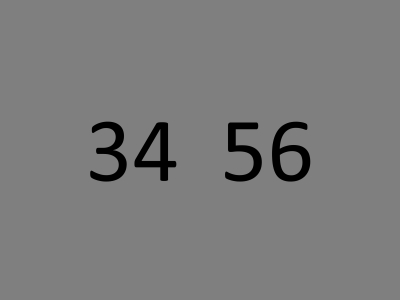

Supplement: S2 File — (ZIP) [file pone.0257717.s002.zip › software/stimuli/HaveToB4.jpg]

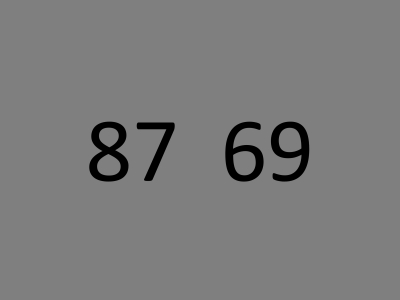

Supplement: S2 File — (ZIP) [file pone.0257717.s002.zip › software/stimuli/HaveToB5.jpg]

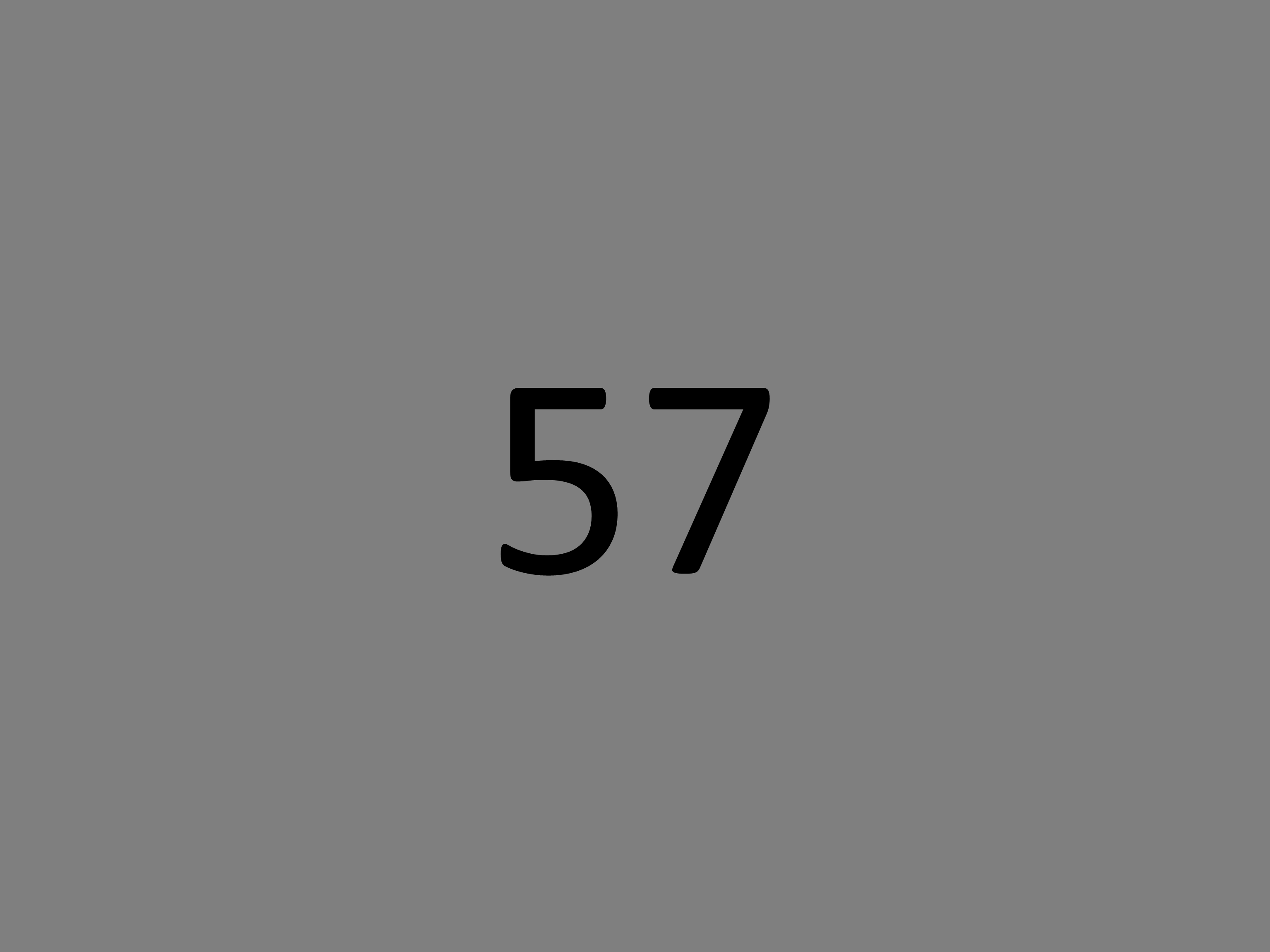

Supplement: S2 File — (ZIP) [file pone.0257717.s002.zip › software/stimuli/HaveToC1.tif]

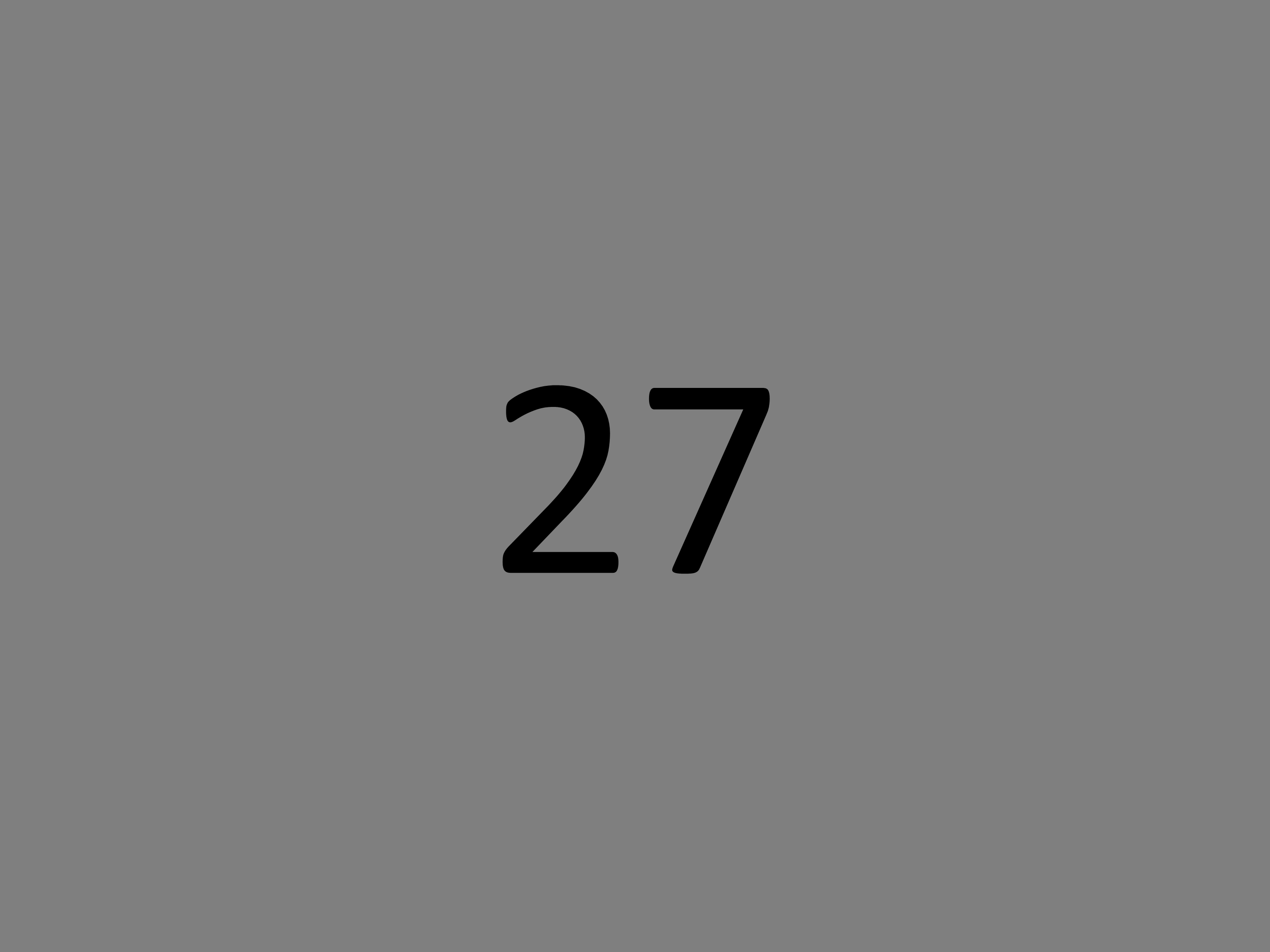

Supplement: S2 File — (ZIP) [file pone.0257717.s002.zip › software/stimuli/HaveToC2.tif]

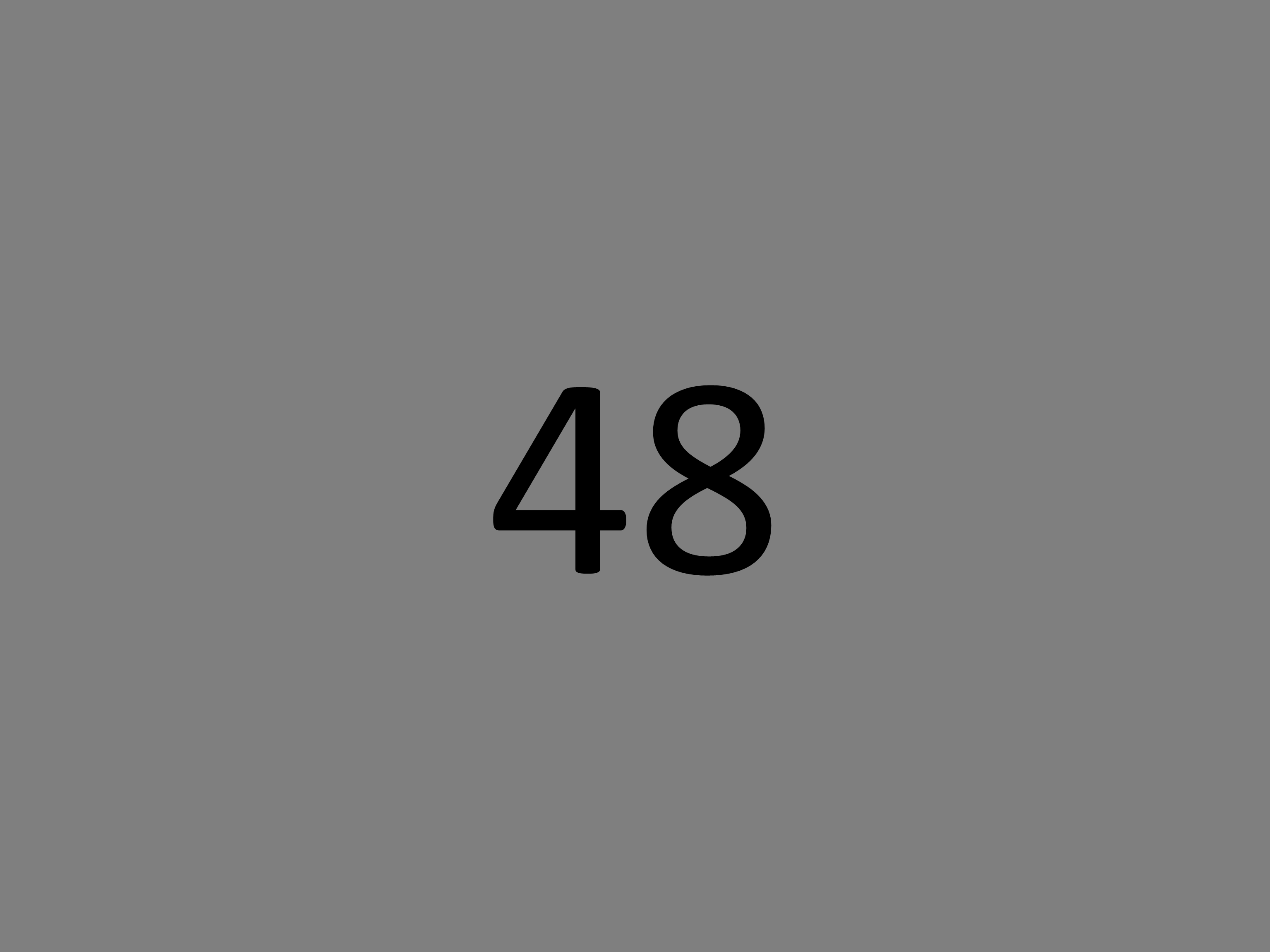

Supplement: S2 File — (ZIP) [file pone.0257717.s002.zip › software/stimuli/HaveToC3.tif]

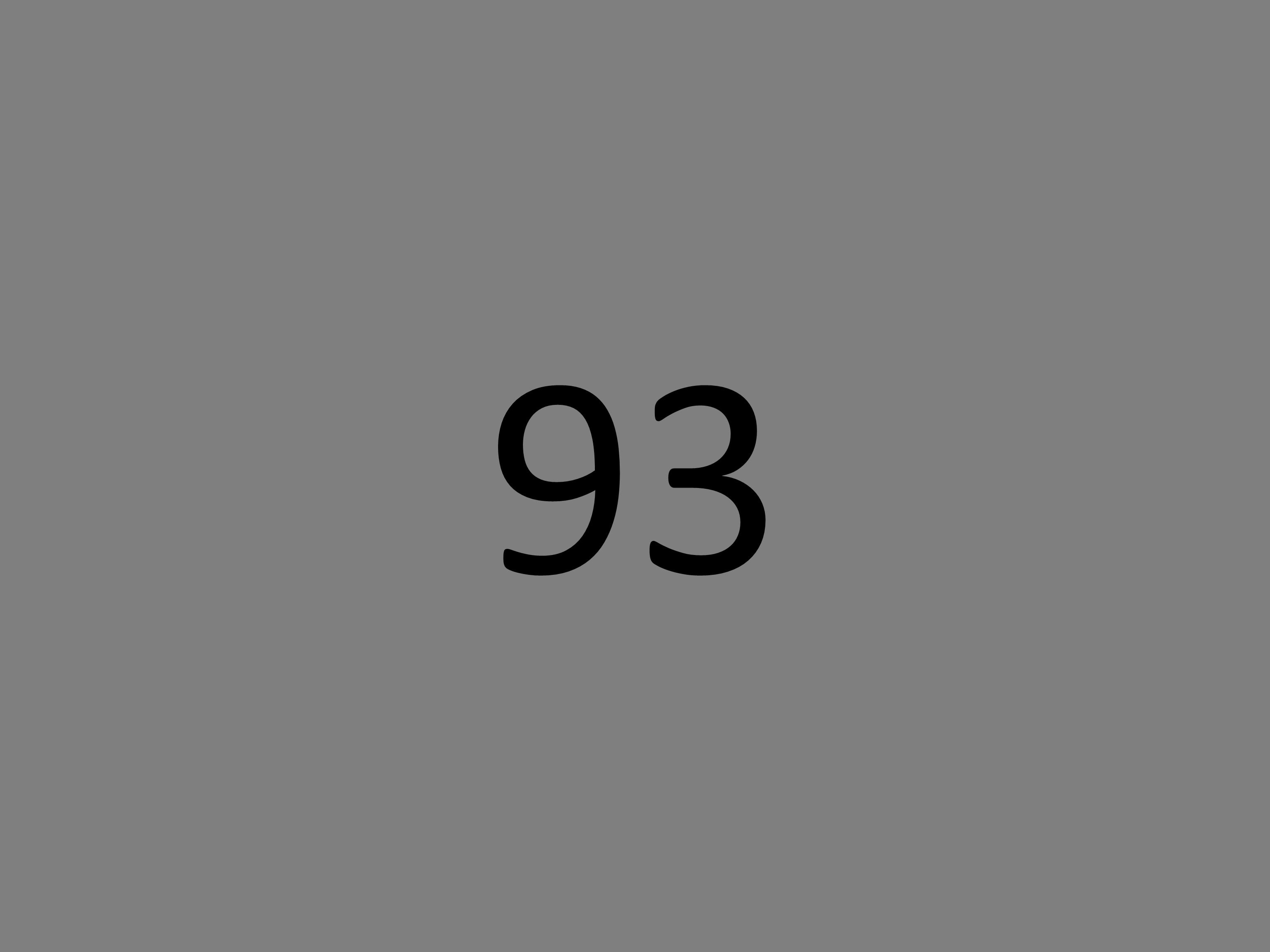

Supplement: S2 File — (ZIP) [file pone.0257717.s002.zip › software/stimuli/HaveToC4.tif]

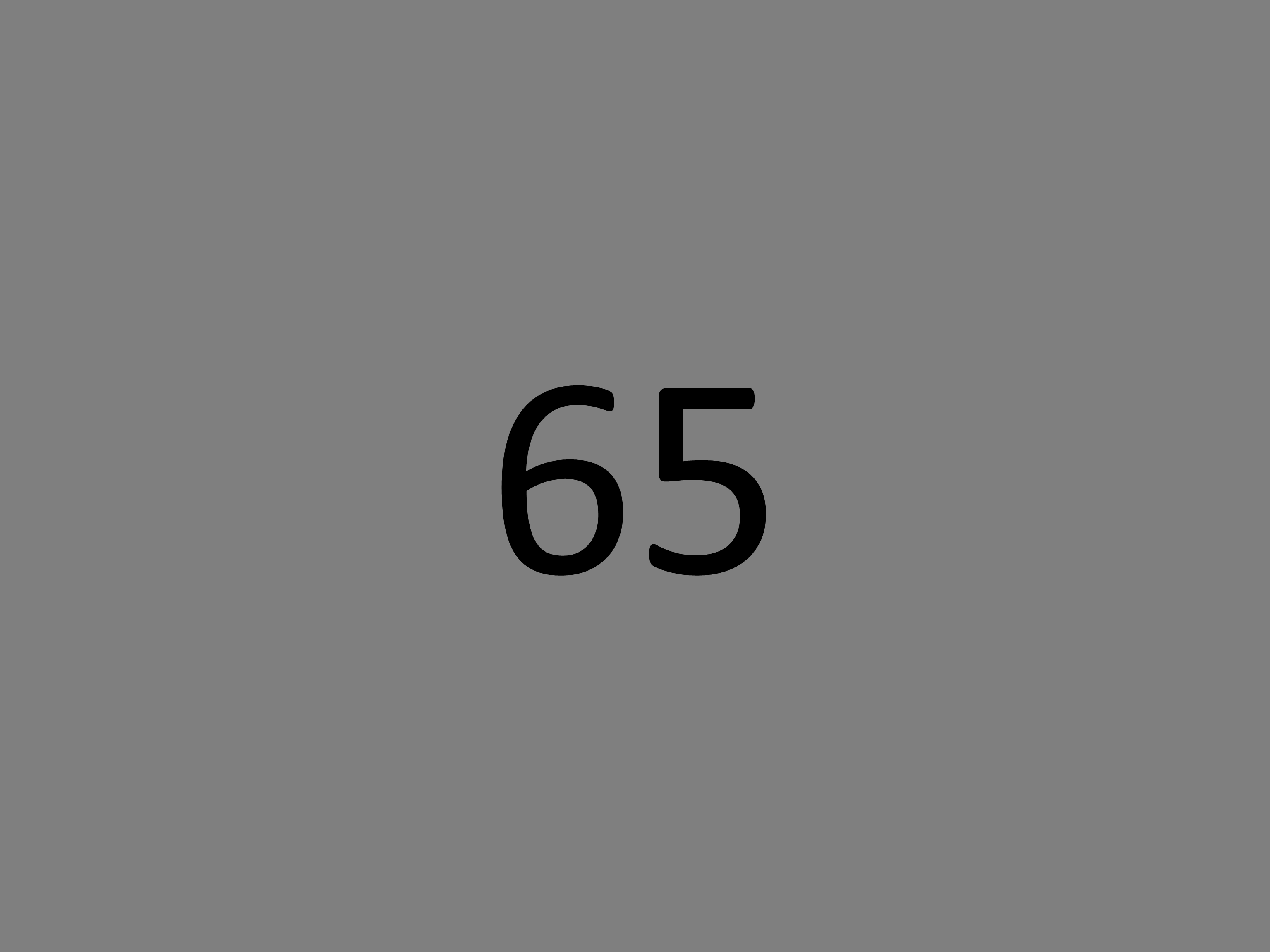

Supplement: S2 File — (ZIP) [file pone.0257717.s002.zip › software/stimuli/HaveToC5.tif]

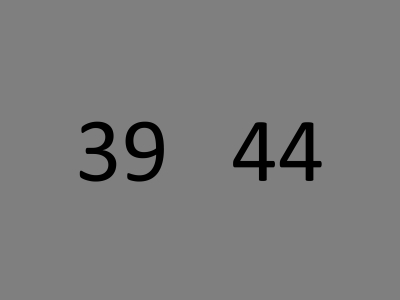

Supplement: S2 File — (ZIP) [file pone.0257717.s002.zip › software/stimuli/HaveToTask1.jpg]

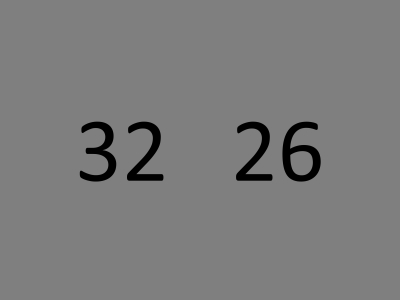

Supplement: S2 File — (ZIP) [file pone.0257717.s002.zip › software/stimuli/HaveToTask10.jpg]

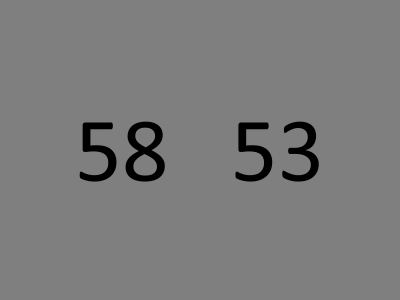

Supplement: S2 File — (ZIP) [file pone.0257717.s002.zip › software/stimuli/HaveToTask11.jpg]

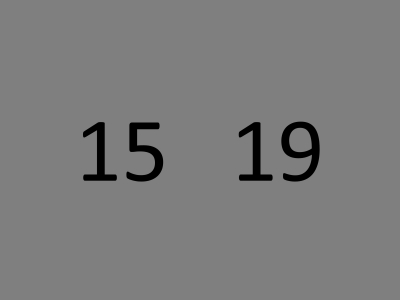

Supplement: S2 File — (ZIP) [file pone.0257717.s002.zip › software/stimuli/HaveToTask12.jpg]

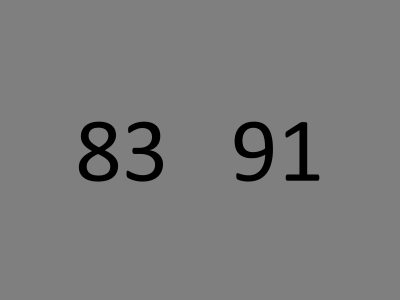

Supplement: S2 File — (ZIP) [file pone.0257717.s002.zip › software/stimuli/HaveToTask13.jpg]

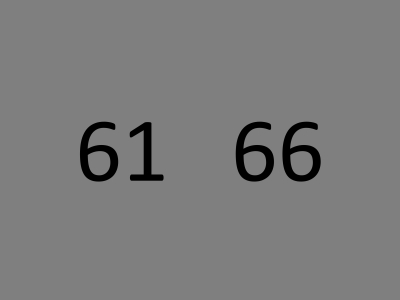

Supplement: S2 File — (ZIP) [file pone.0257717.s002.zip › software/stimuli/HaveToTask14.jpg]

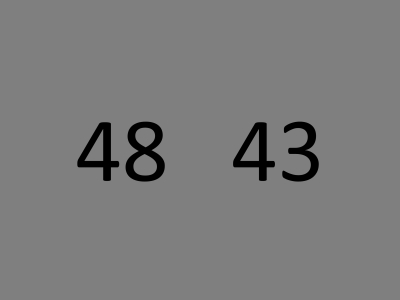

Supplement: S2 File — (ZIP) [file pone.0257717.s002.zip › software/stimuli/HaveToTask15.jpg]

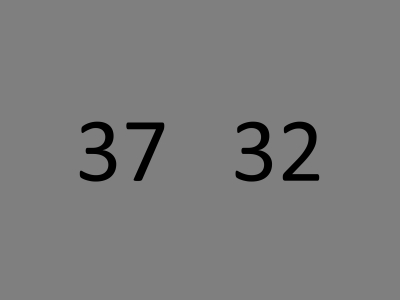

Supplement: S2 File — (ZIP) [file pone.0257717.s002.zip › software/stimuli/HaveToTask16.jpg]

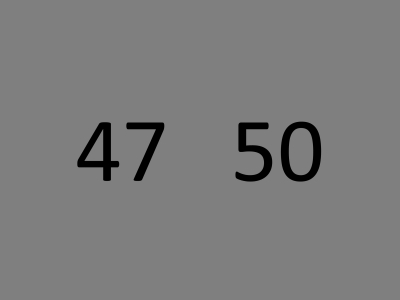

Supplement: S2 File — (ZIP) [file pone.0257717.s002.zip › software/stimuli/HaveToTask17.jpg]

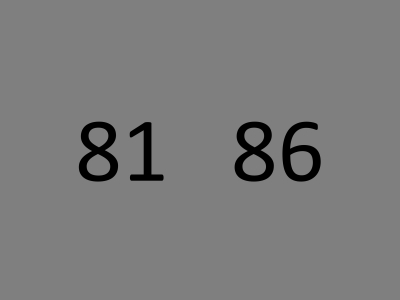

Supplement: S2 File — (ZIP) [file pone.0257717.s002.zip › software/stimuli/HaveToTask18.jpg]

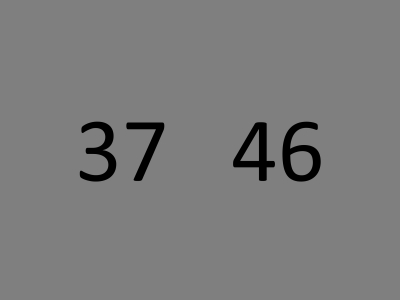

Supplement: S2 File — (ZIP) [file pone.0257717.s002.zip › software/stimuli/HaveToTask19.jpg]

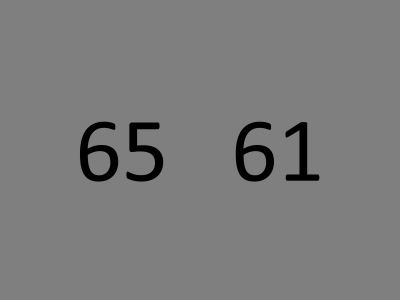

Supplement: S2 File — (ZIP) [file pone.0257717.s002.zip › software/stimuli/HaveToTask2.jpg]

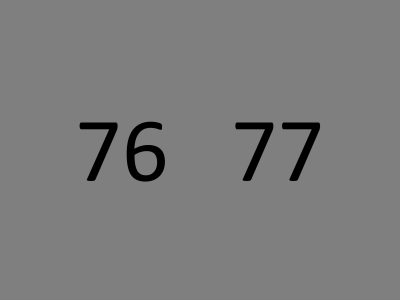

Supplement: S2 File — (ZIP) [file pone.0257717.s002.zip › software/stimuli/HaveToTask20.jpg]

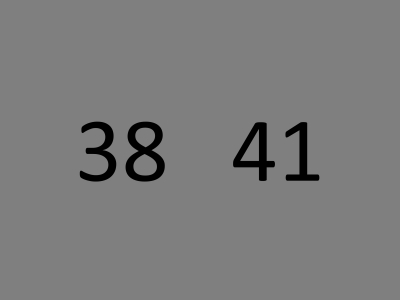

Supplement: S2 File — (ZIP) [file pone.0257717.s002.zip › software/stimuli/HaveToTask21.jpg]

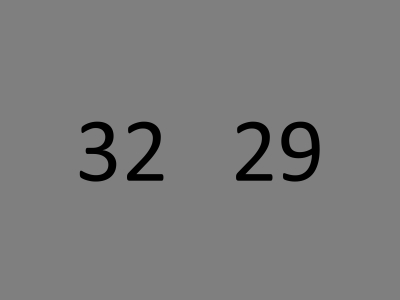

Supplement: S2 File — (ZIP) [file pone.0257717.s002.zip › software/stimuli/HaveToTask22.jpg]

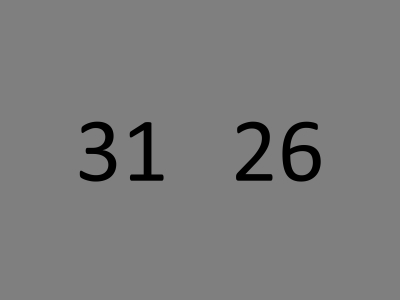

Supplement: S2 File — (ZIP) [file pone.0257717.s002.zip › software/stimuli/HaveToTask23.jpg]

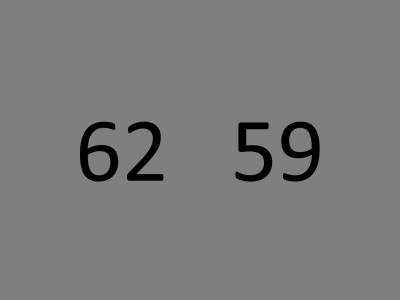

Supplement: S2 File — (ZIP) [file pone.0257717.s002.zip › software/stimuli/HaveToTask24.jpg]

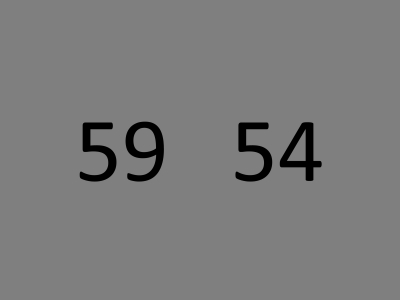

Supplement: S2 File — (ZIP) [file pone.0257717.s002.zip › software/stimuli/HaveToTask25.jpg]

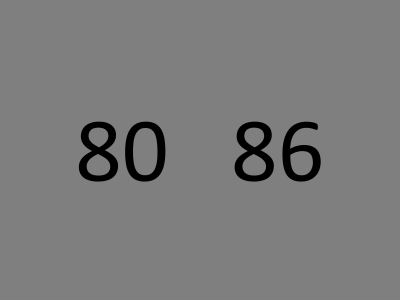

Supplement: S2 File — (ZIP) [file pone.0257717.s002.zip › software/stimuli/HaveToTask26.jpg]

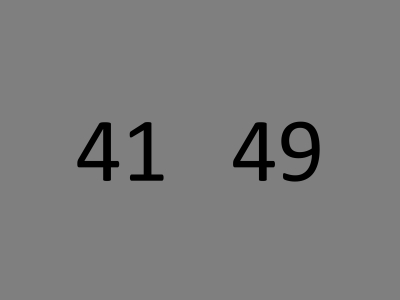

Supplement: S2 File — (ZIP) [file pone.0257717.s002.zip › software/stimuli/HaveToTask27.jpg]

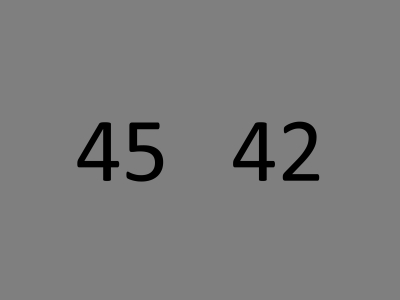

Supplement: S2 File — (ZIP) [file pone.0257717.s002.zip › software/stimuli/HaveToTask28.jpg]

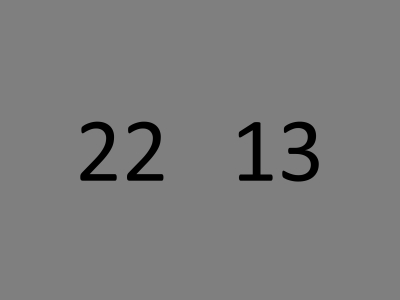

Supplement: S2 File — (ZIP) [file pone.0257717.s002.zip › software/stimuli/HaveToTask29.jpg]

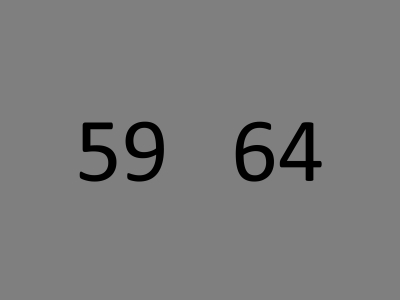

Supplement: S2 File — (ZIP) [file pone.0257717.s002.zip › software/stimuli/HaveToTask3.jpg]

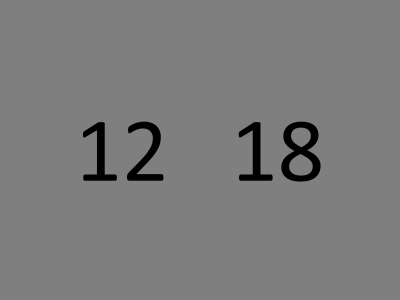

Supplement: S2 File — (ZIP) [file pone.0257717.s002.zip › software/stimuli/HaveToTask30.jpg]

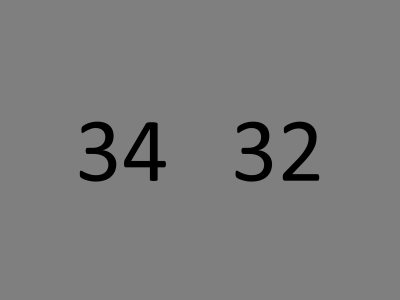

Supplement: S2 File — (ZIP) [file pone.0257717.s002.zip › software/stimuli/HaveToTask31.jpg]

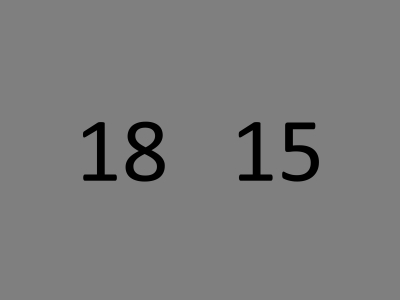

Supplement: S2 File — (ZIP) [file pone.0257717.s002.zip › software/stimuli/HaveToTask32.jpg]

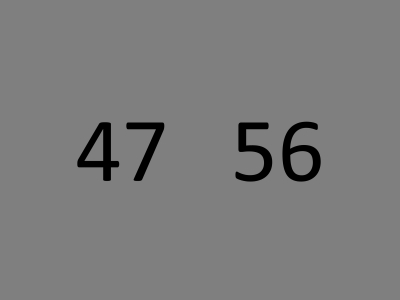

Supplement: S2 File — (ZIP) [file pone.0257717.s002.zip › software/stimuli/HaveToTask33.jpg]

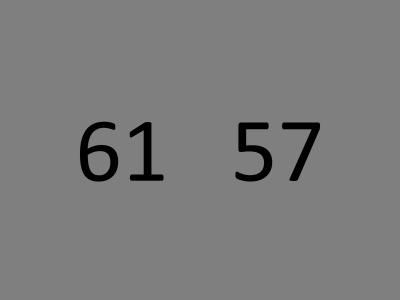

Supplement: S2 File — (ZIP) [file pone.0257717.s002.zip › software/stimuli/HaveToTask34.jpg]

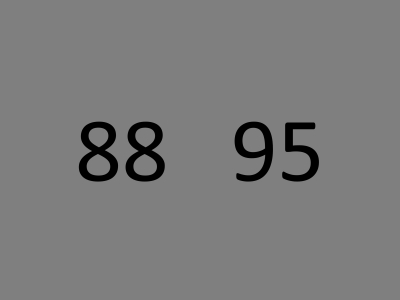

Supplement: S2 File — (ZIP) [file pone.0257717.s002.zip › software/stimuli/HaveToTask35.jpg]

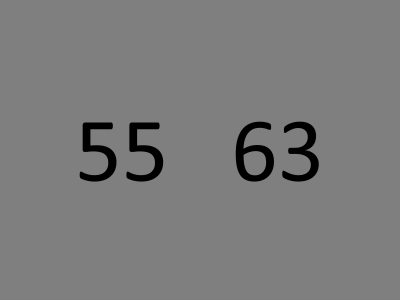

Supplement: S2 File — (ZIP) [file pone.0257717.s002.zip › software/stimuli/HaveToTask36.jpg]

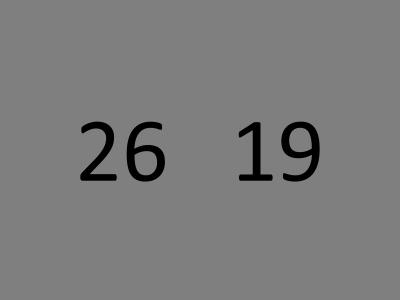

Supplement: S2 File — (ZIP) [file pone.0257717.s002.zip › software/stimuli/HaveToTask37.jpg]

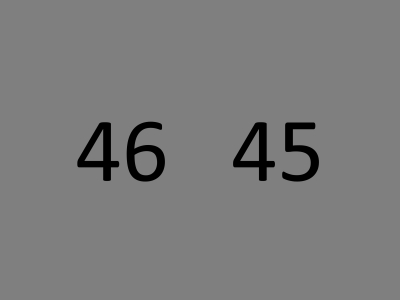

Supplement: S2 File — (ZIP) [file pone.0257717.s002.zip › software/stimuli/HaveToTask38.jpg]

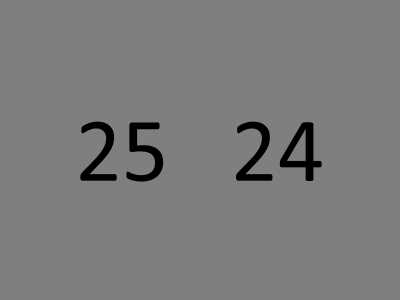

Supplement: S2 File — (ZIP) [file pone.0257717.s002.zip › software/stimuli/HaveToTask39.jpg]

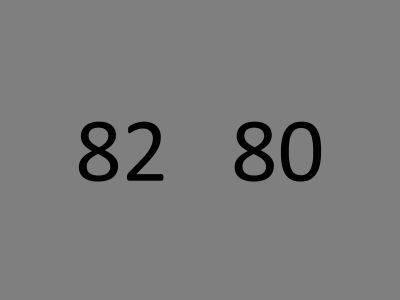

Supplement: S2 File — (ZIP) [file pone.0257717.s002.zip › software/stimuli/HaveToTask4.jpg]

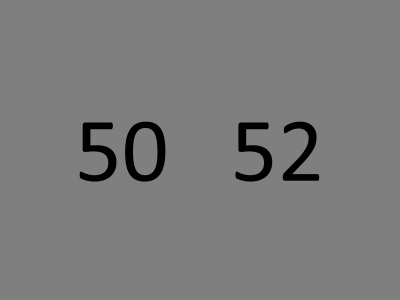

Supplement: S2 File — (ZIP) [file pone.0257717.s002.zip › software/stimuli/HaveToTask40.jpg]

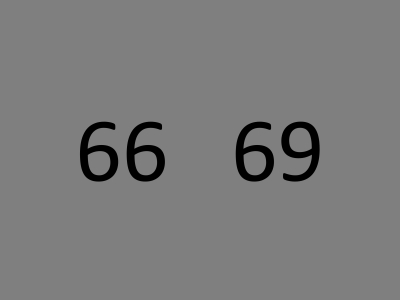

Supplement: S2 File — (ZIP) [file pone.0257717.s002.zip › software/stimuli/HaveToTask41.jpg]

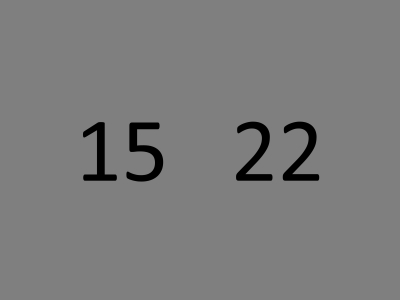

Supplement: S2 File — (ZIP) [file pone.0257717.s002.zip › software/stimuli/HaveToTask42.jpg]

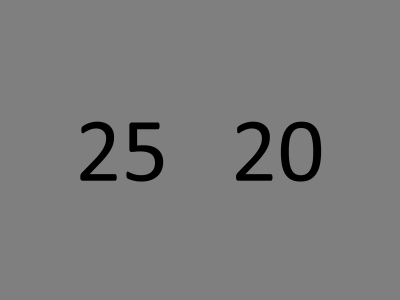

Supplement: S2 File — (ZIP) [file pone.0257717.s002.zip › software/stimuli/HaveToTask43.jpg]

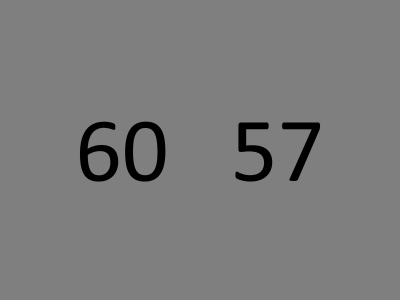

Supplement: S2 File — (ZIP) [file pone.0257717.s002.zip › software/stimuli/HaveToTask44.jpg]

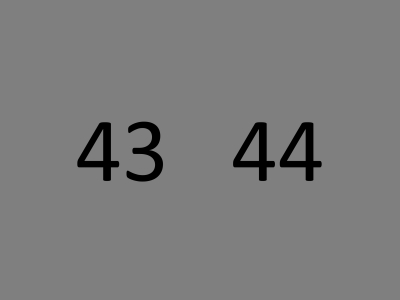

Supplement: S2 File — (ZIP) [file pone.0257717.s002.zip › software/stimuli/HaveToTask45.jpg]

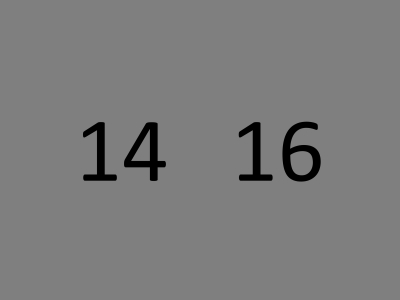

Supplement: S2 File — (ZIP) [file pone.0257717.s002.zip › software/stimuli/HaveToTask46.jpg]

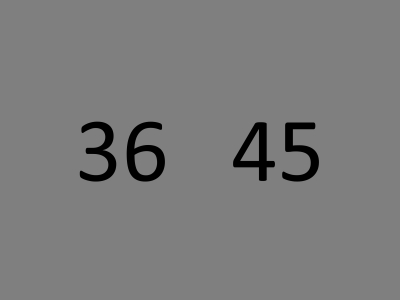

Supplement: S2 File — (ZIP) [file pone.0257717.s002.zip › software/stimuli/HaveToTask47.jpg]

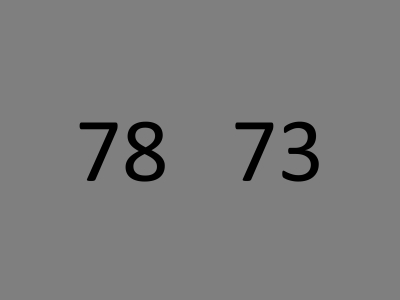

Supplement: S2 File — (ZIP) [file pone.0257717.s002.zip › software/stimuli/HaveToTask48.jpg]

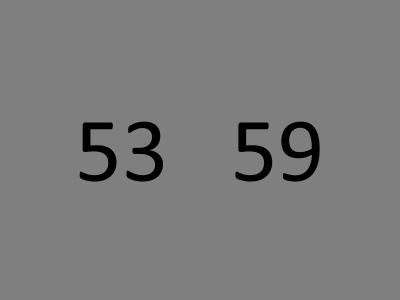

Supplement: S2 File — (ZIP) [file pone.0257717.s002.zip › software/stimuli/HaveToTask49.jpg]

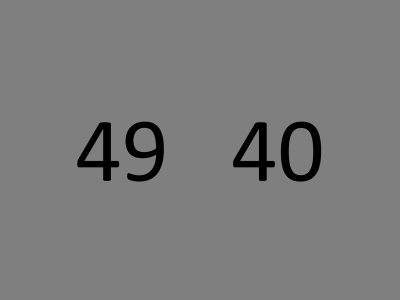

Supplement: S2 File — (ZIP) [file pone.0257717.s002.zip › software/stimuli/HaveToTask5.jpg]

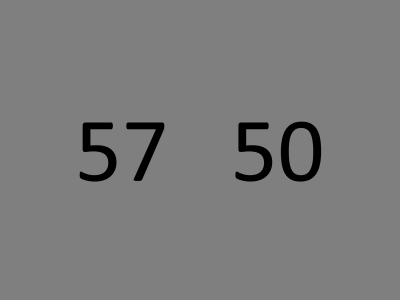

Supplement: S2 File — (ZIP) [file pone.0257717.s002.zip › software/stimuli/HaveToTask50.jpg]

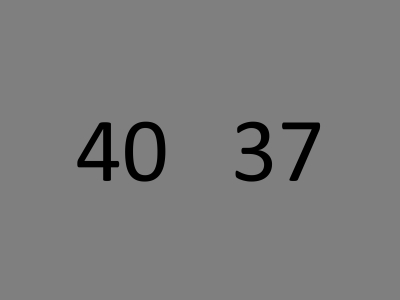

Supplement: S2 File — (ZIP) [file pone.0257717.s002.zip › software/stimuli/HaveToTask51.jpg]

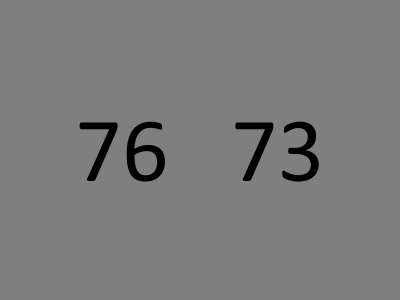

Supplement: S2 File — (ZIP) [file pone.0257717.s002.zip › software/stimuli/HaveToTask52.jpg]

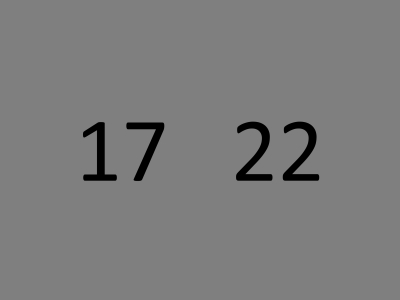

Supplement: S2 File — (ZIP) [file pone.0257717.s002.zip › software/stimuli/HaveToTask53.jpg]

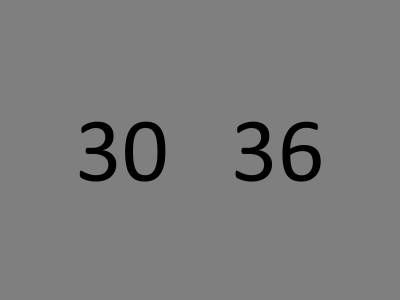

Supplement: S2 File — (ZIP) [file pone.0257717.s002.zip › software/stimuli/HaveToTask54.jpg]

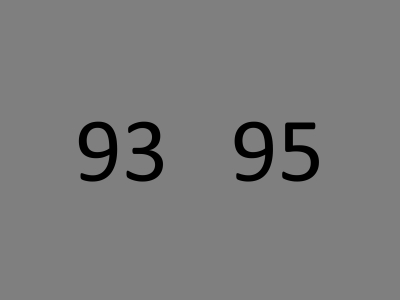

Supplement: S2 File — (ZIP) [file pone.0257717.s002.zip › software/stimuli/HaveToTask55.jpg]

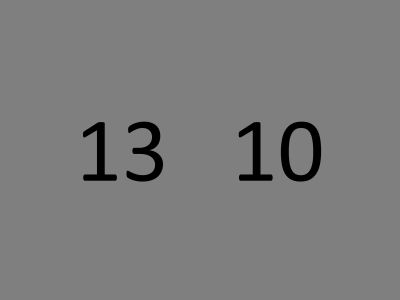

Supplement: S2 File — (ZIP) [file pone.0257717.s002.zip › software/stimuli/HaveToTask56.jpg]

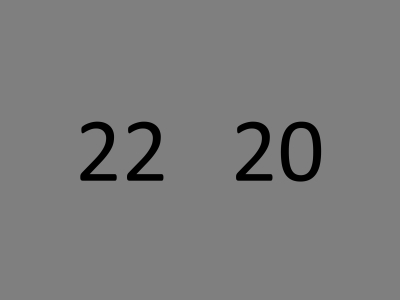

Supplement: S2 File — (ZIP) [file pone.0257717.s002.zip › software/stimuli/HaveToTask57.jpg]

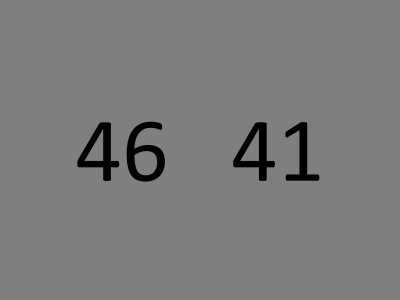

Supplement: S2 File — (ZIP) [file pone.0257717.s002.zip › software/stimuli/HaveToTask58.jpg]

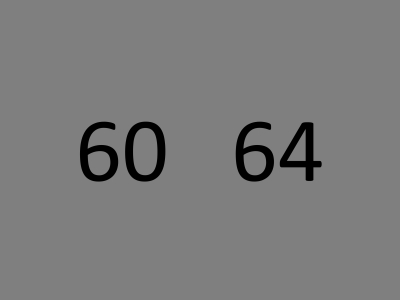

Supplement: S2 File — (ZIP) [file pone.0257717.s002.zip › software/stimuli/HaveToTask59.jpg]

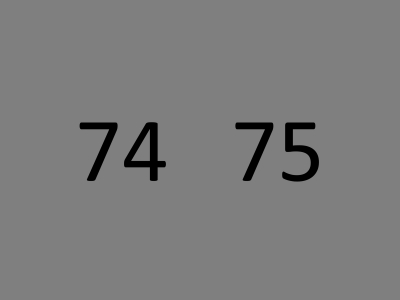

Supplement: S2 File — (ZIP) [file pone.0257717.s002.zip › software/stimuli/HaveToTask6.jpg]

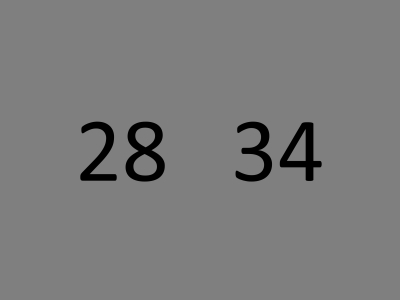

Supplement: S2 File — (ZIP) [file pone.0257717.s002.zip › software/stimuli/HaveToTask60.jpg]

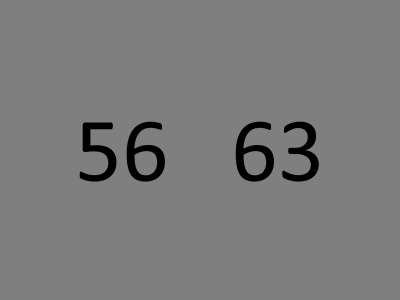

Supplement: S2 File — (ZIP) [file pone.0257717.s002.zip › software/stimuli/HaveToTask61.jpg]

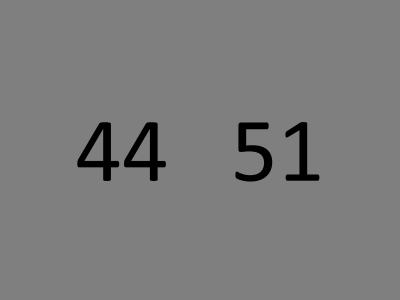

Supplement: S2 File — (ZIP) [file pone.0257717.s002.zip › software/stimuli/HaveToTask63.jpg]

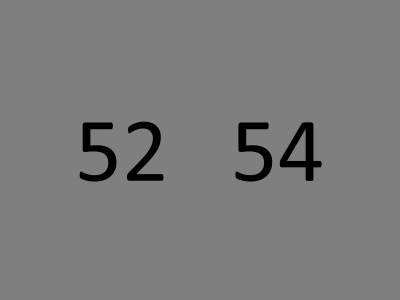

Supplement: S2 File — (ZIP) [file pone.0257717.s002.zip › software/stimuli/HaveToTask64.jpg]

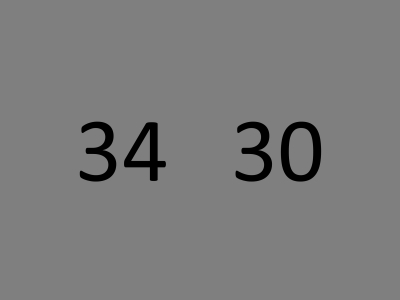

Supplement: S2 File — (ZIP) [file pone.0257717.s002.zip › software/stimuli/HaveToTask65.jpg]

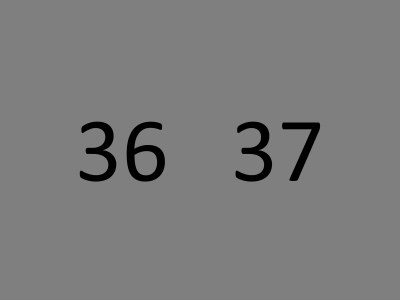

Supplement: S2 File — (ZIP) [file pone.0257717.s002.zip › software/stimuli/HaveToTask66.jpg]

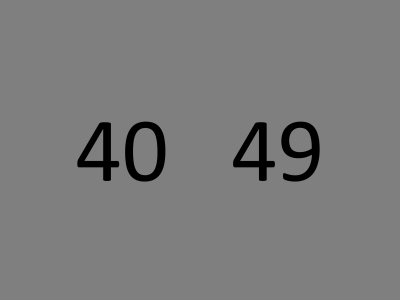

Supplement: S2 File — (ZIP) [file pone.0257717.s002.zip › software/stimuli/HaveToTask67.jpg]

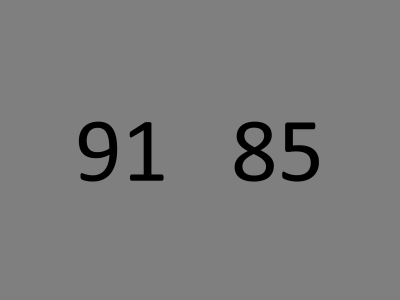

Supplement: S2 File — (ZIP) [file pone.0257717.s002.zip › software/stimuli/HaveToTask68.jpg]

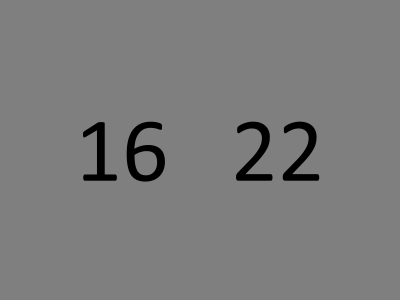

Supplement: S2 File — (ZIP) [file pone.0257717.s002.zip › software/stimuli/HaveToTask69.jpg]

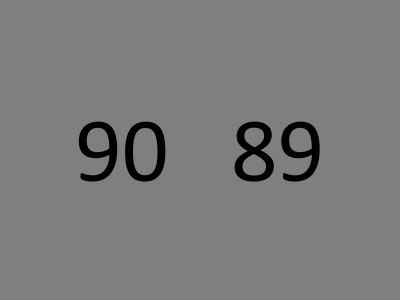

Supplement: S2 File — (ZIP) [file pone.0257717.s002.zip › software/stimuli/HaveToTask7.jpg]

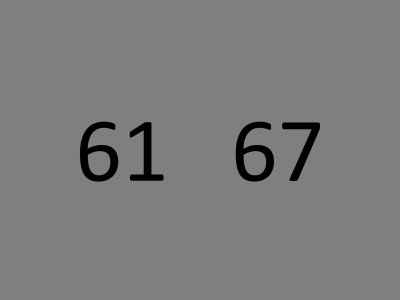

Supplement: S2 File — (ZIP) [file pone.0257717.s002.zip › software/stimuli/HaveToTask70.jpg]

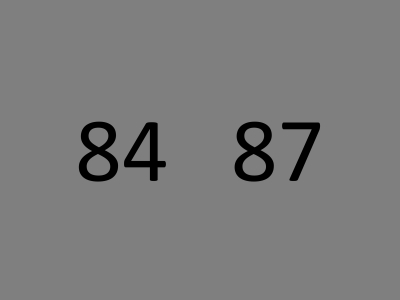

Supplement: S2 File — (ZIP) [file pone.0257717.s002.zip › software/stimuli/HaveToTask71.jpg]

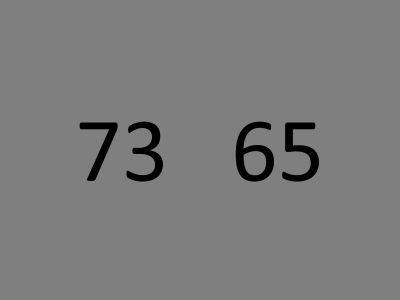

Supplement: S2 File — (ZIP) [file pone.0257717.s002.zip › software/stimuli/HaveToTask72.jpg]

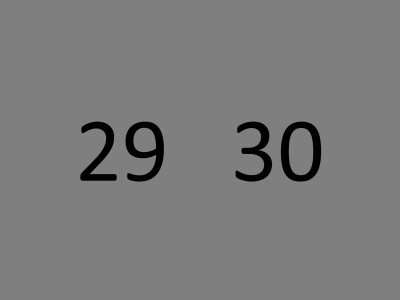

Supplement: S2 File — (ZIP) [file pone.0257717.s002.zip › software/stimuli/HaveToTask73.jpg]

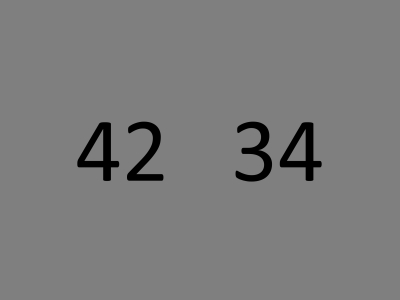

Supplement: S2 File — (ZIP) [file pone.0257717.s002.zip › software/stimuli/HaveToTask74.jpg]

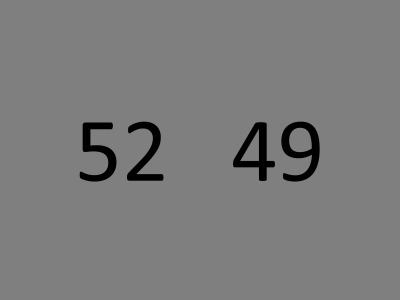

Supplement: S2 File — (ZIP) [file pone.0257717.s002.zip › software/stimuli/HaveToTask75.jpg]

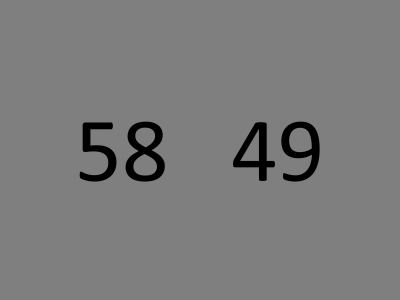

Supplement: S2 File — (ZIP) [file pone.0257717.s002.zip › software/stimuli/HaveToTask76.jpg]

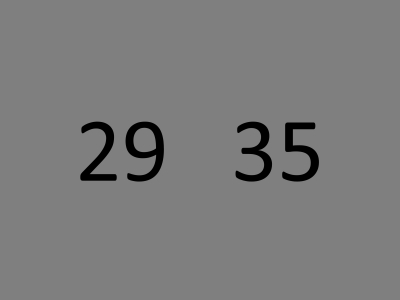

Supplement: S2 File — (ZIP) [file pone.0257717.s002.zip › software/stimuli/HaveToTask77.jpg]

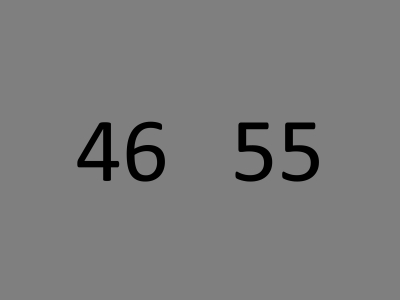

Supplement: S2 File — (ZIP) [file pone.0257717.s002.zip › software/stimuli/HaveToTask78.jpg]

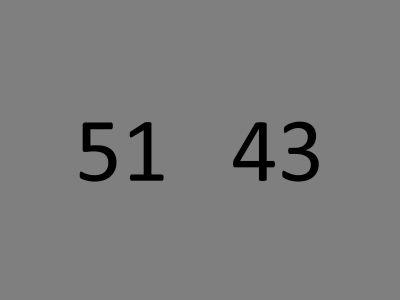

Supplement: S2 File — (ZIP) [file pone.0257717.s002.zip › software/stimuli/HaveToTask79.jpg]

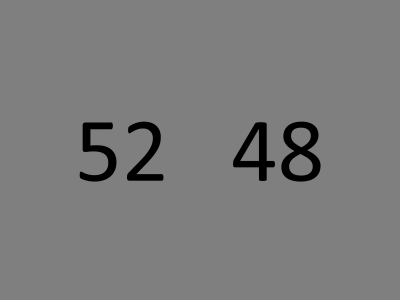

Supplement: S2 File — (ZIP) [file pone.0257717.s002.zip › software/stimuli/HaveToTask8.jpg]

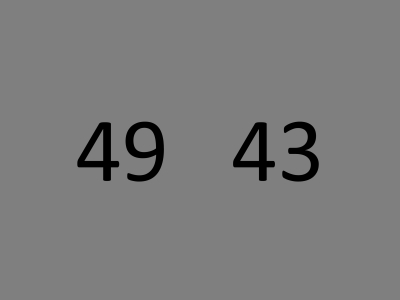

Supplement: S2 File — (ZIP) [file pone.0257717.s002.zip › software/stimuli/HaveToTask80.jpg]

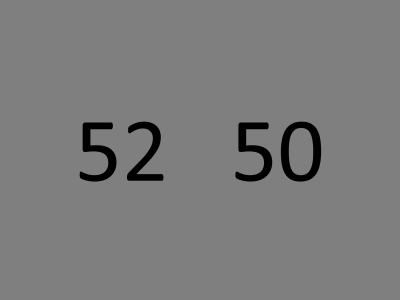

Supplement: S2 File — (ZIP) [file pone.0257717.s002.zip › software/stimuli/HaveToTask81.jpg]

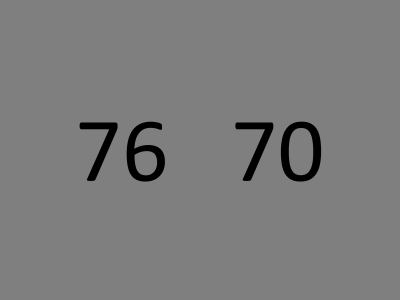

Supplement: S2 File — (ZIP) [file pone.0257717.s002.zip › software/stimuli/HaveToTask82.jpg]

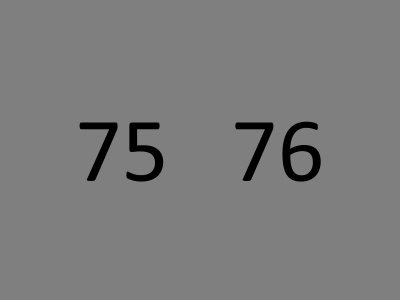

Supplement: S2 File — (ZIP) [file pone.0257717.s002.zip › software/stimuli/HaveToTask83.jpg]

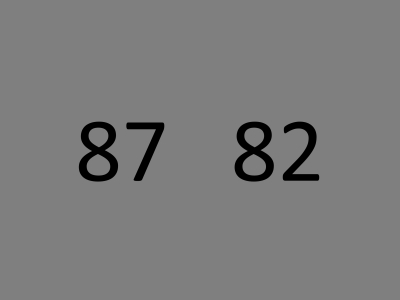

Supplement: S2 File — (ZIP) [file pone.0257717.s002.zip › software/stimuli/HaveToTask84.jpg]

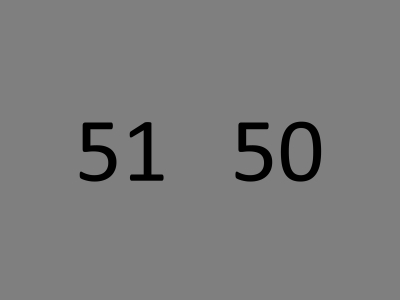

Supplement: S2 File — (ZIP) [file pone.0257717.s002.zip › software/stimuli/HaveToTask85.jpg]
